# Ni/Cu Dual‐Catalyzed Propargylation for the Stereodivergent Synthesis of Methohexital

**DOI:** 10.1002/advs.202406764

**Published:** 2024-07-25

**Authors:** Xihao Chang, Jiayin Zhang, Xiang Cheng, Xianhai Lv, Chang Guo

**Affiliations:** ^1^ College of Materials and Chemistry & School of Plant Protection Anhui Agricultural University Hefei 230036 China; ^2^ Hefei National Research Center for Physical Sciences at the Microscale and Department of Chemistry University of Science and Technology of China Hefei 230026 China

**Keywords:** benzoxazole ester, methohexital, propargylic carbonate, quaternary stereocenter, stereodivergent propargylation, synergistic catalysis

## Abstract

The development of efficient methodologies for the controlled manufacture of specific stereoisomers bearing quaternary stereocenters has prompted advances in a variety of scientific disciplines including pharmaceutical chemistry, materials science, and chemical biology. However, complete control of the absolute and relative stereochemical configurations of alkyne derivatives remains an unmet synthetic challenge. Herein, a Ni/Cu dual‐catalyzed asymmetric propargylic substitution reaction is presented to produce propargylated products with all‐carbon quaternary stereocenters in high yields with significant diastereo‐ and enantioselectivities (up to >20:1 dr, >99% ee). The synthesis of all stereochemical variants of methohexital, a widely used sedative‐hypnotic drug, exemplifies the efficacy of dual‐catalyzed stereodivergent propargylation.

## Graphical abstract

A highly efficient Ni/Cu dual‐catalyzed stereodivergent propargylation reaction is successfully devised, offering a novel approach for the asymmetric synthesis with significant diastereo‐ and enantioselectivities (up to >20:1 dr, >99% ee). This efficient process offers a novel pathway for the stereodivergent total synthesis of Methohexital.

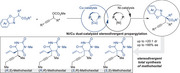

## 1 Introduction

**Figure 1:**
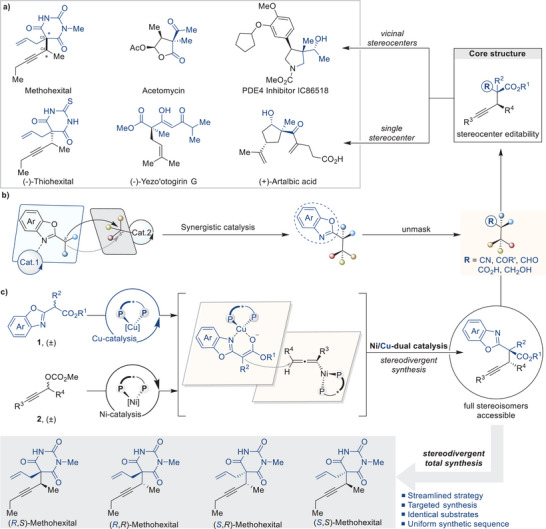
Stereodivergent total synthesis of methohexital. a) Representative biological compounds. b) Synergistic catalysis with benzoxazole esters as surrogates of unsymmetrical 1,3‐dicarbonyl nucleophiles. c) Ni/Cu dual‐catalyzed stereodivergent propargylation for the stereoisomeric synthesis of methohexital.

The chirality of bioactive molecules plays a crucial role in determining their physiological and therapeutic properties, due to the distinctive chiral environments in biological enzymes and receptors.^[^ ^1^, ^2^ ^]^ Naturally occurring compounds and pharmaceutical agents often contain a substantial number of vicinal stereocenters,^[^ ^3^, ^4^ ^]^ and the stereoselective establishment of these structural motifs is of long‐standing interest in the field of synthetic chemistry.^[^ ^5^ ^]^ During the drug discovery and development process, the candidate molecules with multiple stereocenters may have enantiomers that exhibit divergent biological effects.^[^ ^6^ ^]^ This emphasizes the importance of establishing efficient methodologies for the stereodivergent synthesis of all stereoisomers of potential pharmaceutical candidates.^[^ ^7^ ^]^ Methohexital, a barbiturate with contiguous stereocenters and an alkyne moiety, is widely utilized in dentistry as an ultrashort‐acting sedative‐hypnotic agent due to its rapid onset, predictable effects, and brief duration of action.^[^ ^8^, ^9^ ^]^ The selective access of different stereoisomers of methohexital allows for the investigation of structure–activity relationships and presents opportunities for fine‐tuning its pharmaceutical properties.^[^ ^10^ ^]^ However, the lack of versatility of current methods poses a challenge in accessing methohexital with diverse structural and stereochemical variations.^[^ ^11^ ^]^ As part of our ongoing program in developing catalytic stereodivergent total synthesis, we were attracted to the stereodivergent synthesis of methohexital and open avenues for the synthesis of a wide range of stereodiverse propargylated compounds (**Figure** **1**a).^[^ ^12^, ^13^, ^14^, ^15^ ^]^

The dual‐catalyzed asymmetric reaction^[^ ^16^, ^17^, ^18^, ^19^, ^20^ ^]^ has emerged as a powerful strategy for the stereodivergent synthesis of complex molecules, involving the simultaneous control of relative and absolute configurations during the key C*─*C bond‐forming event.^[^ ^21^, ^22^, ^23^, ^24^, ^25^, ^26^ ^]^ Since Carreira and co‐workers reported the groundbreaking stereodivergent α‐allylic alkylation of branched aldehydes by employing the orthogonal permutation of chiral iridium and amine catalysts,^[^ ^27^ ^]^ considerable advancements have been achieved in the synthesis of such reactions in recent years.^[^ ^28^, ^29^, ^30^, ^31^, ^32^, ^33^, ^34^, ^35^, ^36^, ^37^, ^38^, ^39^, ^40^, ^41^, ^42^ ^]^ The stereoselective formation of C*─*C and C*─*heteroatom bonds at the propargylic position plays a crucial role in enhancing skeletal diversity.^[^ ^43^, ^44^, ^45^, ^46^ ^]^ The resulting products are highly amenable to modification, offering significant potential for advancements in total synthesis, materials science, and pharmaceutical development. The nickel‐catalyzed asymmetric propargylation was discovered to be an effective pathway for introducing an internal alkyne group, allowing for a plethora of C*─*C and C*─*heteroatom bond‐forming events.^[^ ^47^, ^48^, ^49^, ^50^, ^51^ ^]^ Following our continuous investigations on nickel‐catalyzed asymmetric propargylation, we further developed a synergistic Ni/Cu catalysis for the stereodivergent coupling of aldimine esters and propargylic carbonates.^[^ ^52^, ^53^ ^]^ However, stereodivergent synthesis involving unsymmetrical 1,3‐dicarbonyl nucleophiles have seldom been achieved to access vicinal quaternary/tertiary propargylic stereocenters and forge the required C4–C5 linkage in methohexital.^[^ ^12^ ^]^ It has been demonstrated that chiral copper(I) complexes act as Lewis acids, efficiently catalyzing the asymmetric functionalization of strategically designed benzoxazole esters via a two‐point binding mechanism,^[^ ^54^, ^55^, ^56^, ^57^ ^]^ and our group has successfully developed the first stereodivergent radical‐polar homocoupling of benzoxazolyl acetate by merging electrosynthesis with synergistic Ni/Cu catalysis.^[^ ^58^ ^]^ We hypothesized that a synergistic catalytic strategy, which involved the asymmetric substitution of primary or secondary propargylic electrophiles with nucleophiles bearing the benzoxazole moiety as a masked acid equivalent, could yield a range of structurally diverse propargylic derivatives with high enantiomeric purity. Through subsequent unmasking of the benzoxazole functionality, this strategy would enable efficient synthesis of a series of α‐quaternary carboxylic acid analogs, including acids, aldehydes, ketones, cyanides, and alcohols, as well as methohexital, with different stereochemical configurations (Figure 1b).^[^ ^59^ ^]^

We developed a synergistic catalytic system that activates nucleophilic benzoxazole esters with chiral copper catalysts^[^ ^54^, ^55^, ^56^, ^57^ ^]^ and propargylic carbonates with chiral nickel catalysts (Figure 1c).^[^ ^47^, ^48^, ^49^, ^50^, ^51^ ^]^ In this catalytic strategy, each catalyst operates on a distinct site, enabling simultaneous control of both the relative and absolute configurations during the C*─*C bond formation, and allows for the feasible construction of stereocenters with high accuracy.^[^ ^60^, ^61^, ^62^, ^63^, ^64^, ^65^, ^66^, ^67^, ^68^, ^69^, ^70^, ^71^, ^72^ ^]^ This strategy has multiple merits. First, the independent control model enables versatile transformation of both primary and secondary propargylic carbonates as substrates, paving the way for accessing all potential stereoisomers of the propargylated products using a unified approach.^[^ ^23^ ^]^ Second, in the dual‐catalyzed propargylic substitution reactions, benzoxazole esters could viably serve as surrogates of 1,3‐dicarbonyl nucleophiles via a logically distinct synthesis design. A wide range of structurally diverse architectures can be readily obtained through the facile interconversion of functional groups on the benzoxazole and alkyne moieties. Third, the ability to synthesize stereodivergent natural products containing contiguous stereocenters can be enhanced by choosing appropriate catalysts under catalytically relevant reaction conditions.^[^ ^53^, ^60^ ^]^ Finally, in contrast to previously reported bimetallic synergistic catalysis, which predominantly relied on utilizing distinct chiral ligands,^[^ ^18^, ^19^, ^20^ ^]^ we have presented another successful case demonstrating the efficacy of employing chiral bidentate phosphine ligands for stereocontrol across different metal catalysts.^[^ ^56^ ^]^ Herein, we showcase the enantioselective α‐propargylation of benzoxazole esters through synergistic metal catalysis, thereby enabling the synthesis of various chiral propargylated building blocks with high levels of chemo‐, regio‐, diastereo‐, and enantioselectivity. By judicious selection of the enantiomeric ligand, the dual‐catalyzed enantioselective propargylation process demonstrated high efficacy in achieving stereodivergent synthesis of all four stereoisomers of methohexital.

## 2 Results and Discussion

### 2.1 Optimization Studies

According to our design plan, the model reaction of racemic benzoxazolyl acetate **1a** with secondary propargylic carbonate **2a** was conducted to investigate the stereodivergent propargylic substitution reaction (**Table** **1**). With a Ni/Cu bimetallic catalytic system consisting of a chiral nickel complex derived from the bidentate phosphine ligand (*R*)−**4a** and a chiral copper complex modified with the Walphos ligand (*S*,*S*_p_)−**5a**, we were able to obtain the desired propargylated product **3a** featuring two adjacent stereocenters in a yield of 93% with a dr of 10:1 and >99% ee (entry 1). The diastereoselectivity was further improved by employing different diphosphine ligands in conjunction with the nickel catalyst (entries 2–4), and (*R*)‐difluorphos **4c** was identified as the privileged one to produce **3a** in 95% yield, >20:1 dr, and 99% ee (entry 3). Subsequently, a series of phosphine ligands **5** for copper catalyst were further evaluated in conjunction with chiral nickel complexes Ni/(*R*)−**4c** (entries 5–7). However, no improvement in reaction efficiency or enantioselectivity was observed. Control experiments validated the necessity of each catalyst component and demonstrated the functional independence and ligand specificity of the chiral nickel catalyst and the chiral copper catalyst (entries 8–11). No reaction occurred in the absence of either Ni(COD)_2_ or the ligand (*R*)−**4c** (entries 8 and 9). In the absence of Cu(MeCN)_4_BF_4_, **3a** was generated in low yield and with inadequate stereoselectivity (entry 10 vs 3). Furthermore, the absence of (*S*,*S*_p_)−**5a** led to a substantial decrease in diastereoselectivity (entry 11 vs 3). The thorough kinetic study, monitored by gas chromatography‐mass spectrometry, demonstrated that the utilization of the Ni/Cu bimetallic catalytic system is more efficient compared to single nickel catalysis in the propargylation reaction. This study highlights the synergistic effect of copper and nickel catalysts in achieving the requisite stereochemistry when appropriate ligands are employed.

**Table 1:** Survey on the model reaction conditions.

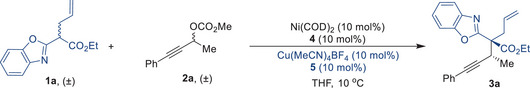							
Entry^a)^	[Ni]	[Cu]	4	5	yield [%]^b)^	dr^c)^	ee [%]^d)^
1	Ni(COD)_2_	Cu(MeCN)_4_BF_4_	(*R*)−**4a**	(*S*,*S*_p_)−**5a**	93	10:1	>99
2	Ni(COD)_2_	Cu(MeCN)_4_BF_4_	(*R*)−**4b**	(*S*,*S*_p_)−**5a**	92	7:1	>99
3	Ni(COD)_2_	Cu(MeCN)_4_BF_4_	(*R*)−**4c**	(*S*,*S*_p_)−**5a**	95	>20:1	>99
4	Ni(COD)_2_	Cu(MeCN)_4_BF_4_	(*R*)−**4d**	(*S*,*S*_p_)−**5a**	91	18:1	>99
5	Ni(COD)_2_	Cu(MeCN)_4_BF_4_	(*R*)−**4c**	(*S*,*S*_p_)−**5b**	91	5:1	>99
6	Ni(COD)_2_	Cu(MeCN)_4_BF_4_	(*R*)−**4c**	(*S*,*R*_p_)−**5c**	89	1.3:1	98
7	Ni(COD)_2_	Cu(MeCN)_4_BF_4_	(*R*)−**4c**	(*R*,*R*)−**5d**	93	6:1	>99
8	−	Cu(MeCN)_4_BF_4_	(*R*)−**4c**	(*S*,*S*_p_)−**5a**	nr	−	−
9	Ni(COD)_2_	Cu(MeCN)_4_BF_4_	−	(*S*,*S*_p_)−**5a**	nr	−	−
10	Ni(COD)_2_	−	(*R*)−**4c**	(*S*,*S*_p_)−**5a**	45	1.4:1	89
11	Ni(COD)_2_	Cu(MeCN)_4_BF_4_	(*R*)−**4c**	−	89	1.6:1	99
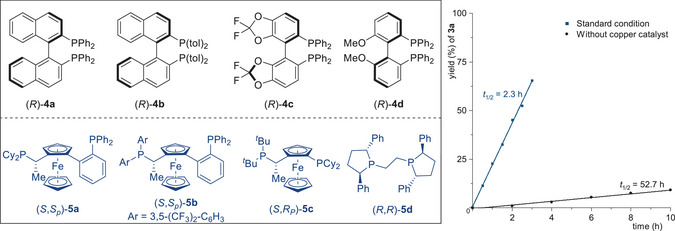							

^a)^ Reactions in this table were conducted with **1a** (0.15 mmol), **2a** (0.3 mmol), Ni(COD)_2_ (10 mol%), **4** (10 mol%), Cu(MeCN)_4_BF_4_ (10 mol%), and **5** (10 mol%) in tetrahydrofuran (THF) at 10 °C for 72 h;

^b)^ Isolated yields after chromatography;

^c)^ Determined by 1H NMR spectroscopy of the crude reaction mixture;

^d)^ ee values were determined by high‐performance liquid chromatography analysis. nr = no reaction.

### 2.2 Substrate Scope

After establishing the optimal reaction conditions, we proceeded to conduct a methodical investigation of the generality of our methodology (**Table** **2**). Initial validation demonstrated that variations in the ester group of benzoxazole acetates **1** were well‐tolerated, resulting in the corresponding products with high yields and great stereoselectivities (**3a**‐**3e**). Moreover, the consistent achievement of high yields and stereoselectivities in an upscaled reaction for the synthesis of compound **3a** provides compelling evidence of the scalability of our methodology. Furthermore, the introduction of different substituents at the α‐position of the carbonyl group in compound **1** facilitated a successful reaction with racemic propargylic carbonate **2a**. The propargylic substitution adducts **3** were obtained with dr values ranging from 12:1 to >20:1, and ee values all >99% (**3f‐3m**), thereby demonstrating the robustness and practicality of the present reaction. It is worth noting that nitrogen‐containing aromatic moieties, such as benzothiazolyl and pyridinyl, can also facilitate the synthesis of asymmetric propargylic substitution products (**3n** and **3o**). Additionally, a diverse range of racemic propargylic carbonates **1** with electron‐withdrawing or electron‐donating substituents on the *para* position of the benzene ring performed well in this synergistic reaction (**3p**‐**3u**). Furthermore, substituents at either the *meta*‐ or *ortho*‐positions on the benzene ring had no significant impact on the reaction outcomes (**3v**‐**3x**). Notably, the propargylic carbonate bearing a thiophene substituent was also tolerated in the reaction, affording the desired product **3y** in 90% yield with >20:1 dr and >99% ee. This approach was additionally demonstrated to be compatible with dialkyl‐substituted propargylic carbonates, which produced the desired products without affecting the reaction efficiency or enantiocontrol (**3z**‐**3ac**). Propargylic carbonates with heteroatomic alkyl (**3ad**‐**3af**), cycloalkyl (**3ag**), or cycloalkenyl (**3ah**) substituents, as well as those bearing different groups on the propargylic carbon center (**3ai‐3al**), all exhibit good tolerance in the process.

**Table 2:** Substrate scope of Ni/Cu dual‐catalyzed enantioselective propargylation of secondary propargylic carbonates.

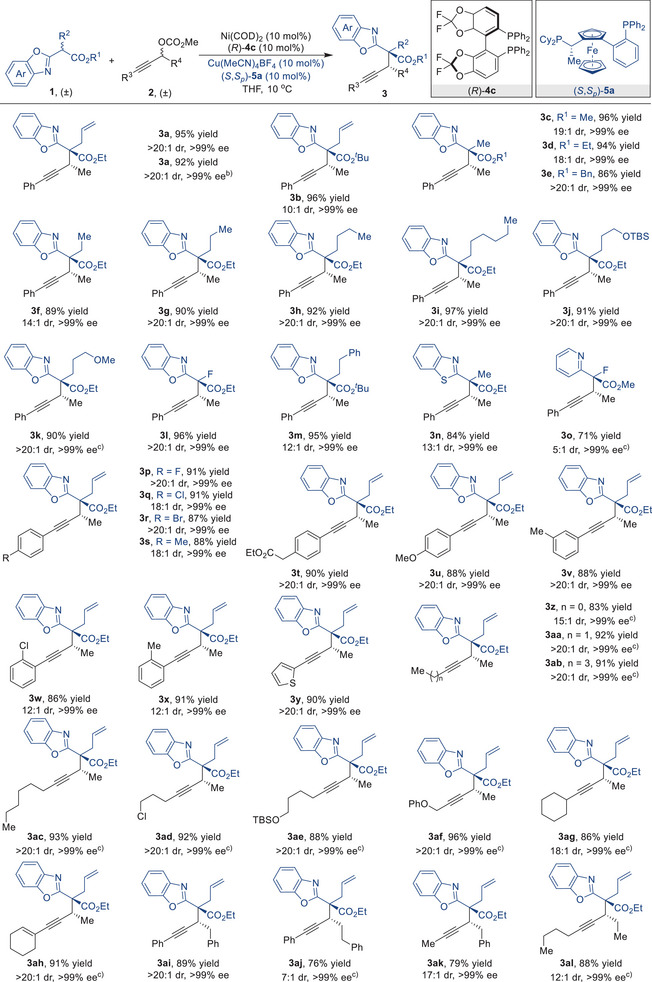

^a)^ Unless otherwise specified, all the reactions were carried out by using Ni(COD)_2_ (10 mol%), (*R*)−**4c** (10 mol%), Cu(MeCN)_4_BF_4_ (10 mol%), (*S*,*S*_p_)−**5a** (10 mol%), **1** (0.15 mmol, 1.0 equiv), and **2** (0.3 mmol, 2.0 equiv) in tetrahydrofuran (THF) at 10 °C for 72 h. b) 3.0 mmol scale reaction. c) The reaction was performed at 20 °C.

**Figure 2:**
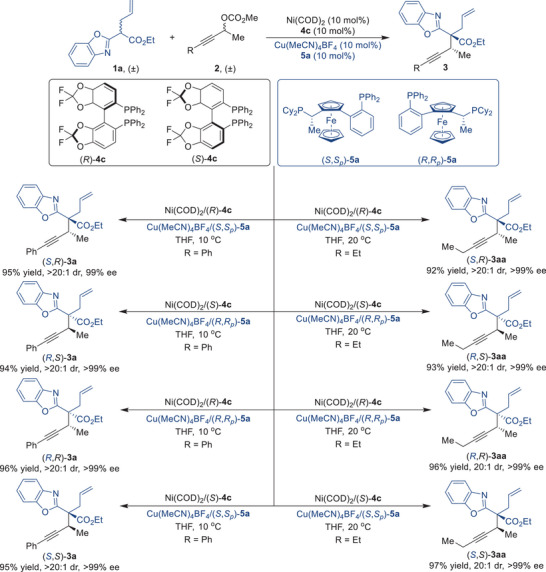
Ni/Cu dual‐catalyzed stereodivergent propargylic substitution reactions.

To highlight the stereodivergence of the Ni/Cu synergistic catalytic system, benzoxazolyl acetate **1a** and propargylic carbonate **2** were subjected to stereoselective propargylation reactions with four different pairs of enantiomers of the nickel catalyst and the copper catalyst under the same conditions (**Figure** **2**). Remarkably, all four stereoisomers of desired products **3a** and **3aa** were produced in 92%–97% yields with dr values of 20:1 to >20:1 and ee values of >99%, showing an exceptional capacity to perform stereodivergent synthesis.

After establishing a robust and modular method for the stereodivergent synthesis of continuous chiral centers using Ni/Cu dual catalysis based on secondary propargylic carbonates, our focus shifted to the synthesis of propargylated products with a single chirality center to demonstrate the universality and broad applicability of this synergistic catalytic system. Using the Ni/Cu synergistic catalytic approach, the generality of the reaction between benzoxazole esters **1** and primary propargylic carbonates **6** was investigated (**Table** **3**). It was found that a range of benzoxazole esters could be employed to produce the desired products **7a**‐**7j** with yields of 79%–97% and enantiomeric excesses (ee) of 92%–97%. The absolute configuration of **7c** was assigned by single‐crystal X‐ray diffraction analysis. The incorporation of various ester groups into benzoxazole esters **1** shows good compatibility in forming the target compounds with notable yields and high enantioselectivity (**7k**‐**7m**). The presence of electron‐donating and ‐withdrawing substituents on the benzene ring of propargylic carbonates **6** was nicely tolerated, thereby furnishing the corresponding products with up to 96% yield and 99% ee (**7n**‐**7v**). This method also works well with propargyl carbonate with a thiophene substituent and produces the desired product **7w** in 94% yield and 98% ee. Aliphatic propargyl carbonates also underwent the desired reaction to give the alkylation products with satisfying results (**7x**‐**7ac**).

**Table 3:** Substrate scope of Ni/Cu dual‐catalyzed enantioselective propargylation of primary propargylic carbonates.

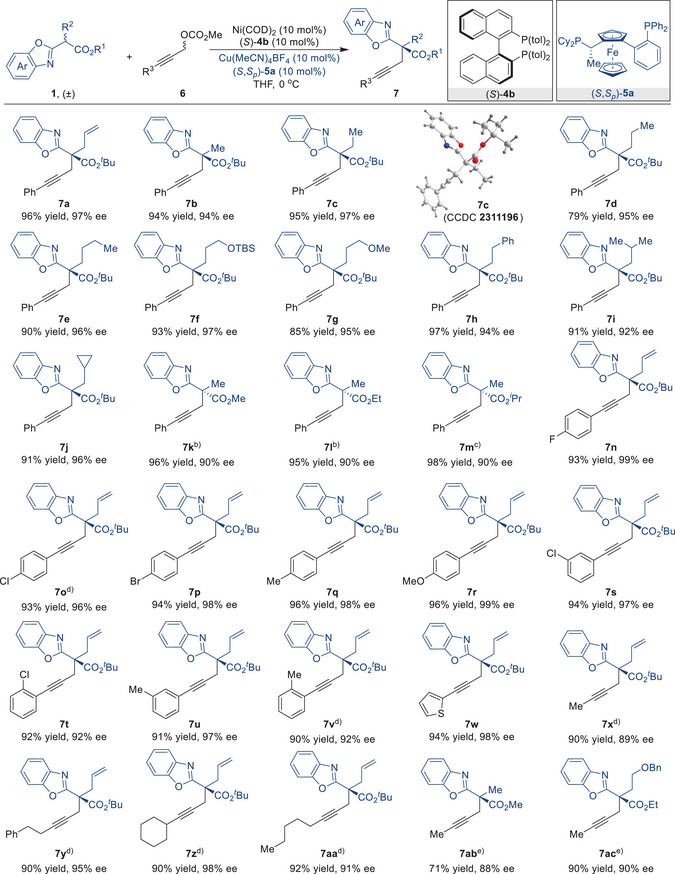

^a)^ Unless otherwise specified, all the reactions were carried out by using Ni(COD)_2_ (10 mol%), (*S*)−**4b** (10 mol%), Cu(MeCN)_4_BF_4_ (10 mol%), (*S*,*S*_p_)−**5a** (10 mol%), **1** (0.15 mmol, 1.0 equiv), and **6** (0.3 mmol, 2.0 equiv) in tetrahydrofuran (THF) at 0 °C for 72 h. b) (*S,S*)‐BPE [(*S,S*)−**5d**] (10 mol%) was used instead of (*S*,*S*_p_)−**5a**. c) The reaction was performed by using Ni(COD)_2_ (10 mol%), (*S*)‐Difluorphos [(*S*)−**4c**] (10 mol%), Cu(MeCN)_4_BF_4_ (10 mol%), and (*S,S*)−**5d** (10 mol%) in 1,4‐dioxane at 10 °C for 72 h. d) The reaction was performed at 20 °C. e) (*R*)‐Difluorphos [(*R*)−**4c**] (10 mol%) was used instead of (*S*)−**4b**, and the reaction was performed at 20 °C.

### 2.3 Synthetic Application

**Figure 3:**
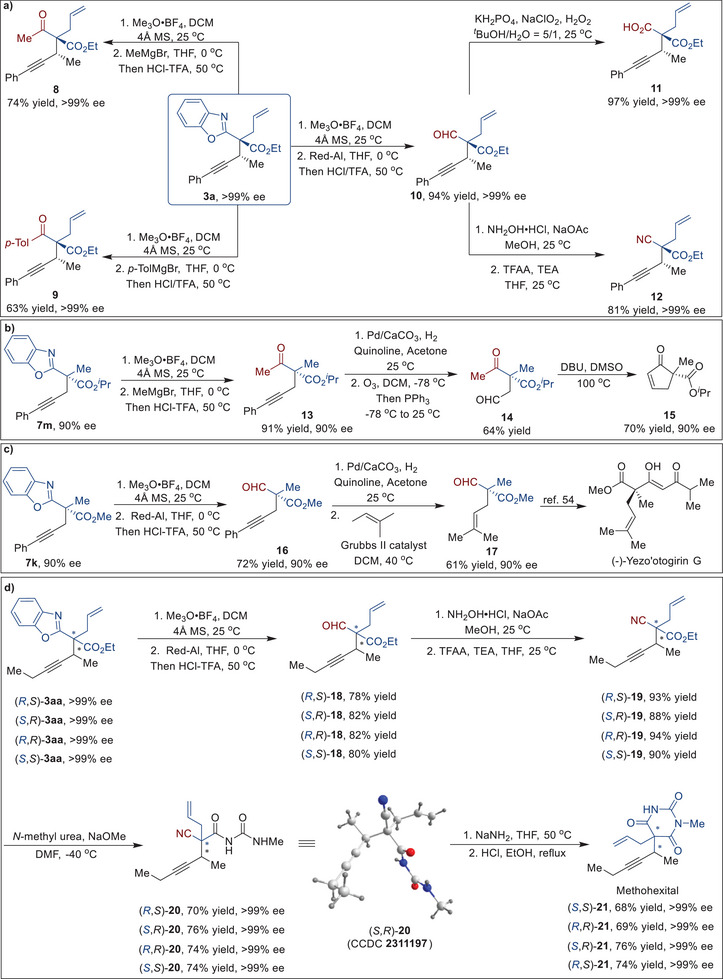
Synthetic utilities and stereodivergent transformations. a) Synthetic transformations of propargylated product 3a. b) Concise asymmetric synthesis of cyclic unsaturated ketone 15. c) Concise asymmetric formal synthesis of (−)‐Yezo'otogirin G. d) Stereodivergent total synthesis of all four stereoisomers of methohexital.

To highlight the applicability of the current synthetic process, the benzoxazole group of enantioenriched propargylation product **3a** was further functionalized into multiple types of valuable building blocks (**Figure** **3**a). By utilizing trimethyloxonium tetrafluoroborate as the methylation agent, the benzoxazole portion of **3a** was then reacted with different Grignard reagents, and achieved good yields of ketones **8** and **9** without any loss of enantiopurity. The benzoxazole group can also undergo sequential methylation, reduction, and hydrolysis to produce aldehyde **10**, which can be efficiently transformed into carboxylic acid **11** and cyanide **12**. These efficient methods enable the selective conversion of the benzoxazole moiety without compromising the required stereochemistry.

The utility of this asymmetric propargylation reaction was further demonstrated by the stereoselective collective synthesis of essential intermediates, bioactive compounds, and natural products (Figure 3b). The enantioenriched propargylated product **7m**, obtained through the synergistic catalytic strategy, the benzoxazole group was removed successfully to afford the ketone **13** in 91% yield. The alkyne group in ketone **13** was then reduced to a *cis*‐alkene moiety using Lindlar's catalyst, which was then oxidized with ozone to generate aldehyde **14**. The intermolecular cyclization of aldehyde **14** resulted in the formation of cyclic unsaturated ketone **15** with a yield of 74%, which can be further applied in the synthesis of artalbic acid.^[^ ^13^ ^]^ We then employed the dual‐catalyzed approach for the asymmetric formal synthesis of Yezo'otogirin G (Figure 3c). The propargylated product **7k** can undergo functional conversion of the benzoxazole group, followed by reduction and olefin metathesis reactions, to yield the essential intermediate **17** required for the subsequent synthesis of Yezo'otogirin G.^[^ ^73^ ^]^

Despite the significant impact of methohexital in the field of clinical medicine, the current absence of asymmetric catalytic methods for synthesizing all stereoisomers of methohexital hinders the investigation of the relationship between stereochemical structure and anesthetic activity.^[^ ^8^, ^9^, ^10^ ^]^ We initiated the stereodivergent total synthesis of methohexital by starting with access to four stereoisomers of propargylated product **3aa** (Figure 3d). The benzoxazole group of compound **3aa** underwent a facile conversion to form aldehyde **18**. Subsequently, the transformation of aldehyde **18** into cyanide **19** took place, followed by the reaction with *N*‐methylurea under alkaline conditions to generate compound **20**. The absolute configuration of (*S*,*R*)−**20** was confirmed by X‐ray diffraction analysis. A final cyclization of compound **20** afforded methohexital **21** in 68% yield with an enantiomeric excess of >99% ee, demonstrating the effectiveness of the current stereodivergent propargylation methods. The concise synthesis of all stereoisomers of methohexital **21** was achieved in a uniform synthetic sequence. Furthermore, this synthetic approach would offer new opportunities for investigating the relationship between the stereostructure and anesthetic activity of methohexital **21**.^[^ ^10^ ^]^

### 2.4 Mechanistic Investigation

**Figure 4:**
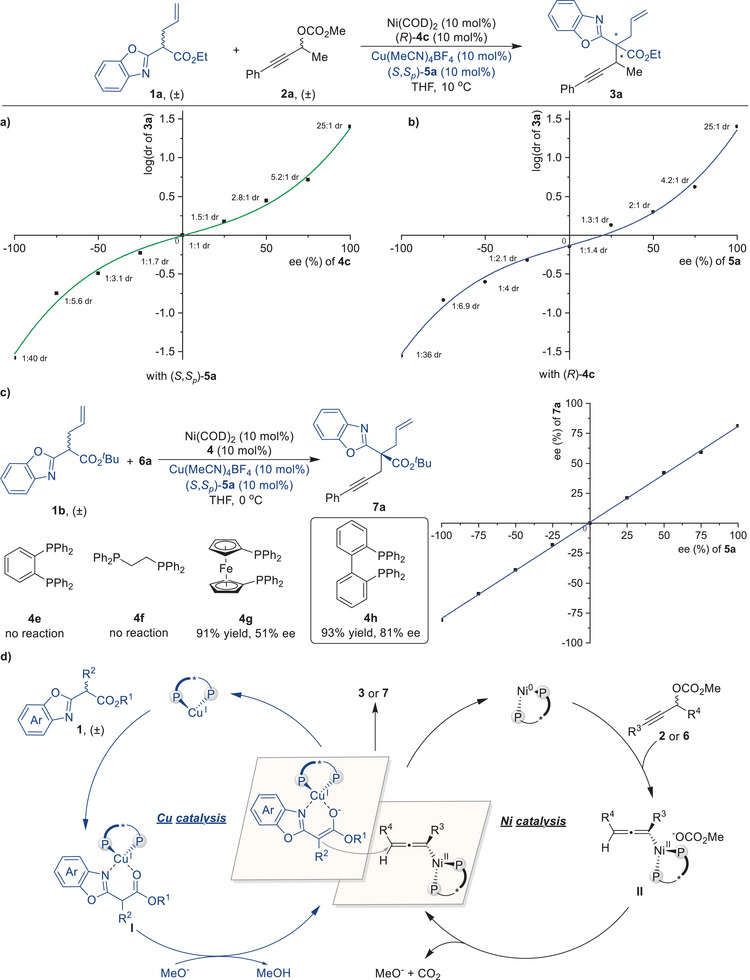
Mechanistic studies. a) Investigation of the relationship between the enantioselectivity of **4c** and the diastereoselectivity of **3a**. b) Investigation of the relationship between the enantioselectivity of **5a** and the diastereoselectivity of **3a**. c) Nonlinear effect investigation with **4h** as an achiral ligand of nickel catalyst. d) Proposed mechanism.

A series of preliminary mechanistic experiments were carried out to get insight into the mechanism of this Ni/Cu‐catalyzed asymmetric propargylic substitution reaction (**Figure** **4**). We observed a correlation between the enantiomeric purity of ligand **4c** and the diastereomeric ratio of product **3a** (Figure 4a), highlighting the crucial role of the chiral nickel complex in controlling the stereoselectivity. Further investigation revealed a similar relationship between the enantiomeric purity of ligand **5a** and the diastereomeric ratio of **3a** (Figure 4b), also indicating the importance of chiral copper complexes in achieving stereoselective control. The combined findings indicate that both the chiral nickel and copper catalysts work cooperatively during the bimetallic synergistic catalytic process. Various achiral phosphine ligands (**4e**‐**4h**) were evaluated, and the use of BIPHEP (**4h**) for the nickel complex resulted in the desired propargylated product **7a** in 93% yield with 81% ee (Figure 4c). To explore the nonlinear effects of this reaction, a nickel complex with an achiral ligand **4h** and a copper complex with an ee‐varied chiral ligand **5a** were utilized in subsequent experiments. Remarkably, a linear relationship was observed between the ee of **7a** and the enantiopurity of **5a**, suggesting that one molecule of copper catalyst was involved in the enantio‐determining step.

Based on our mechanistic investigations, we propose a plausible synergistic catalytic cycle as depicted in Figure 4d. The catalytic cycles initiate with the binding of a copper catalyst to benzoxazole ester **1**, resulting in the formation of a nucleophilic copper‐bound enolate **I**. Concurrently, Ni‐mediated decarboxylation of propargylic carbonate **2** or **6** generates an electrophilic allenyl‐nickel complex **II**. Specifically, the methoxy group generated from the Ni‐mediated decarboxylation of propargylic carbonate **2** or **6** serves as a base, facilitating the deprotonation in the copper cycle, thereby assisting in the formation of nucleophilic copper‐bound enolate **I**. Subsequently, copper‐bound enolate **I** undergoes nucleophilic substitution of the allenyl‐nickel complex **II**, leading to the production of the final propargylated product (**3** or **7**) and simultaneous regeneration of both catalysts.^[^ ^74^ ^]^ The chiral nickel catalyst and the copper complex work cooperatively in the propargylic alkylation process and allow independent control of each chiral center, providing a powerful tool for stereodivergent synthesis.

## 3 Conclusion

In summary, a Ni/Cu dual‐catalyzed stereodivergent propargylic substitution reaction was developed using racemic propargyl carbonate and benzoxazole ester as starting materials. The propargylation reaction exhibited high enantioselectivity and stereodivergence, enabling the synthesis of propargylated products with privileged frameworks commonly found in biological molecules and natural products. Moreover, a concise and unified stereodivergent total synthesis of methohexital has been successfully accomplished by employing a straightforward combination of enantiomeric nickel and copper complexes within identical starting materials. The combination of nickel and copper catalysts shows great promise as a versatile platform for advancing various stereodivergent reactions with extensive applicability.

## 4 Experimental Section

### General Procedure for the Synthesis of Product 3

In a nitrogen‐filled glove box, an oven‐dried 10 mL screw‐cap reaction tube equipped with a stir bar was charged with Ni(COD)_2_ (4.1 mg, 0.015 mmol, 10 mol%), and (*R*)−**4c** (10.2 mg, 0.015 mmol, 10 mol%) in THF (1 mL) at rt for ≈20 min. Meanwhile, Cu(MeCN)_4_BF_4_ (4.7 mg, 0.015 mmol, 10 mol%) and (*S,S*_p_)−**5a** (10.1 mg, 0.015 mmol, 10 mol%) were stirred in THF (1 mL) in a Schlenk flask under a nitrogen atmosphere for 30 min. Benzoxazole ester **1** (0.15 mmol, 1.0 equiv.) was added to the Schlenk flask containing copper complex and stirred for ≈5 min. Propargylic carbonate **2** (0.3 mmol, 2.0 equiv.) was then transformed into the Screw‐cap reaction tube containing a nickel complex and stirred for an additional 5 min. The nickel complex solution was then combined with the copper complex solution, and the resulting solution was stirred for ≈72 h at 10 °C until substrate **1** was completely consumed (monitored by TLC). The reaction mixture was subsequently concentrated under vacuum and purified by flash column chromatography on silica gel to afford the desired product **3**.

### General Procedure for the Synthesis of Product 7

In a nitrogen‐filled glove box, an oven‐dried 10 mL screw‐cap reaction tube equipped with a stir bar was charged with Ni(COD)_2_ (4.1 mg, 0.015 mmol, 10 mol%) and (*S*)−**4b** (10.2 mg, 0.015 mmol, 10 mol%) in THF (1 mL) at rt for ≈20 min. Meanwhile, Cu(MeCN)_4_BF_4_ (4.7 mg, 0.015 mmol, 10 mol%) and (*S,S*_p_)−**5a** (10.1 mg, 0.015 mmol, 10 mol%) were stirred in THF (1 mL) in a Schlenk flask under a nitrogen atmosphere for 30 min. Benzoxazole ester **1** (0.15 mmol, 1.0 equiv.) was added to the Schlenk flask containing the copper complex and stirred for ≈5 min. Propargylic carbonate **6** (0.3 mmol, 2.0 equiv.) was then transformed into the Screw‐cap reaction tube containing the nickel complex and stirred for an additional 5 min. The nickel complex solution was then combined with the copper complex solution and the resulting solution was stirred for ≈72 h at 0 °C until substrate **1** was completely consumed (monitored by TLC). The reaction mixture was subsequently concentrated under vacuum and purified by flash column chromatography on silica gel to afford the desired product **7**.

## Conflict of Interest

The authors declare no conflict of interest.

## Supporting information

Supporting Information

## Data Availability

The data that support the findings of this study are available in the Supporting Information of this article.
