# Supplementary material for: Ni/Cu Dual‐Catalyzed Propargylation for the Stereodivergent Synthesis of Methohexital
Source: Adv Sci (Weinh). 2024 Jul 25;11(36):2406764. doi: 10.1002/advs.202406764 (PMC11423103; doi:10.1002/advs.202406764)
Supplement: Supplementary file 1 — Supporting Information [file ADVS-11-2406764-s001.pdf]

ADVANCED SCIENCE
Open Access

Supporting Information

for *Adv. Sci.*, DOI 10.1002/advs.202406764

Ni/Cu Dual-Catalyzed Propargylation for the Stereodivergent Synthesis of Methohexital

Xihao Chang, Jiayin Zhang, Xiang Cheng, Xianhai Lv and Chang Guo**

Ni/Cu Dual-Catalyzed Propargylation for the Stereodivergent Synthesis of Methohexital

Xihao Chang[1]✉, Jiayin Zhang[2], Xiang Cheng[1], Xianhai Lv[1], Chang Guo[2]✉

[1]College of Materials and Chemistry & School of Plant Protection, Anhui Agricultural University, Hefei, 230036, China.
[2]Hefei National Research Center for Physical Sciences at the Microscale and Department of Chemistry, University of Science and Technology of China, Hefei, 230026, China.
✉Email: guochang@ustc.edu.cn
✉Email: changxihao@ahau.edu.cn

Table of contents

1. General Information S2

2. Synthesis and characterization of products S3

3. Synthetic utilities and stereodivergent transformations S36

4. Mechanistic Studies S54

5. X-ray Crystallography Data S62

6. NMR Spectra S64

7. HPLC Traces S169

8. References S262

This PDF file includes:

Supplementary Text
Figs. S1 to S6
Tables S1 to S3

1. General Information

Unless otherwise noted, all catalysts or reagents were purchased from commercial suppliers and used without further purification. Benzoxazole esters **1**,[1-3] and propargylic carbonates **2** and **6**[4] were synthesized following the procedures in the literature. All reactions were carried out in flame-dried glassware under a dry nitrogen atmosphere. ^{1}H NMR and ^{13}C NMR spectra were recorded at 25 ºC on Bruker Advance 400 M or 500 M NMR spectrometers ($CDCl_3$ as the solvent). Chemical shifts for ^{1}H NMR spectra are reported as δ in units of parts per million (ppm) downfield from $SiMe_4$ (δ 0.00) and relative to the signal of chloroform-*d* (δ 7.26, singlet). Multiplicities were given as: s (singlet); d (doublet); t (triplet); q (quartet); dd (doublet of doublets); dt (doublet of triplets); m (multiplets), etc. Coupling constants are reported as a *J* values in Hz. ^{13}C NMR spectra are reported as δ in units of parts per million (ppm) downfield from $SiMe_4$ (δ 0.00) and relative to the signal of chloroform-*d* (δ 77.16, triplet). High-resolution mass spectral analysis (HRMS) was performed on a Waters XEVO G2 Q-TOF. Optical rotations were measured at 589 nm (sodium D line) by using a Perkin-Elmer-343 polarimeter. The configurations of **7c** and (*S,R*)**-20** were assigned by the X-ray analysis. The measurement of enantiomeric excesses was performed on a Waters-Alliance (2998, Photodiode Array Detector). CHIRALCEL OJ-H, CHIRALPAK IA, IC, IE, IF, IG, AS-H, and AD-H columns were purchased from Daicel Chemical Industries, LTD.

2. Synthesis and characterization of the products

2.1 Optimization studies of 3a

Table S1. Optimization studies of different metal catalysts[a]

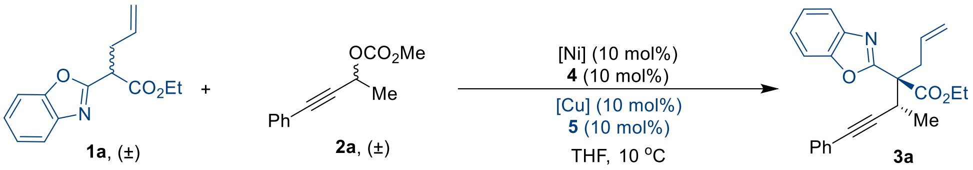

entry	[Ni]	[Cu]	4	5	yield (%)[b]	dr[c]	ee (%)[d]
1	$Ni(PPh_3)_4$	$Cu(MeCN)_4BF_4$	(*R*)-**4c**	(*S*,*S*$_p$)-**5a**	nr	—	—
2	Ni(COD)(DQ)	$Cu(MeCN)_4BF_4$	(*R*)-**4c**	(*S*,*S*$_p$)-**5a**	nr	—	—
3	$Ni(COD)_2$	$Cu(MeCN)_4PF_6$	(*R*)-**4c**	(*S*,*S*$_p$)-**5a**	89	>20:1	>99
4	$Ni(COD)_2$	$Cu(OTf)_2$	(*R*)-**4c**	(*S*,*S*$_p$)-**5a**	54	8:1	>99
5	$Ni(COD)_2$	CuI	(*R*)-**4c**	(*S*,*S*$_p$)-**5a**	23	1:1	98%/98%
6	$Ni(COD)_2$	CuCl	(*R*)-**4c**	(*S*,*S*$_p$)-**5a**	16	1:1	98%/97%

[a]Reactions in this table were conducted with **1a** (0.15 mmol), **2a** (0.3 mmol), [Ni] (10 mol%), **4** (10 mol%), [Cu] (10 mol%), and **5** (10 mol%) in tetrahydrofuran (THF) at 10 °C for 72 h. [b]Isolated yields after chromatography. [c]Determined by ^{1}H NMR spectroscopy of the crude reaction mixture. [d]ee values were determined by high-performance liquid chromatography analysis. nr = no reaction.

Table S2. Survey on the catalytic loading of model reaction[a]

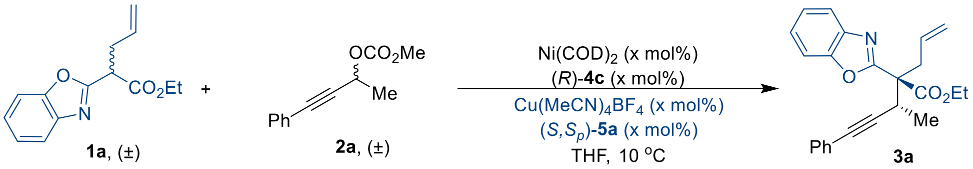

entry	Loading of $Ni(COD)_2$	Loading of $Cu(MeCN)_4BF_4$	yield (%)[b]	dr[c]	ee (%)[d]
1	8 mol%	8 mol%	93	>20:1	>99
2	6 mol%	6 mol%	89	>20:1	>99
3	4 mol%	4 mol%	75	16:1	>99
4	2 mol%	2 mol%	14	15:1	>99

[a]Reactions in this table were conducted with **1a** (0.15 mmol), **2a** (0.3 mmol), $Ni(COD)_2$ (x mol%), (*R*)-**4c** (x mol%), $Cu(MeCN)_4BF_4$ (x mol%), and (*S*,*S*$_p$)-**5a** (x mol%) in tetrahydrofuran (THF) at 10 °C for 72 h. [b]Isolated yields after chromatography. [c]Determined by ^{1}H NMR spectroscopy of the crude reaction mixture. [d]ee values were determined by high-performance liquid chromatography analysis.

2.2 Scale-up synthesis procedure of product 3a

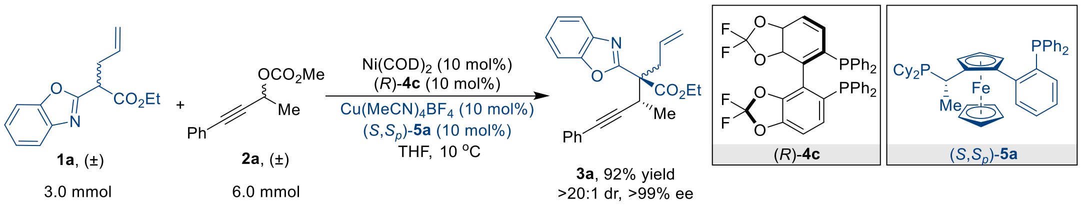

In a nitrogen-filled glove box, an oven-dried 100 mL screw-cap reaction tube equipped with a stir bar was charged with Ni(COD)$_2$ (82.5 mg, 0.3 mmol, 10 mol%) and (*R*)-**4c** (204.8 mg, 0.3 mmol, 10 mol%) in THF (20 mL) at rt for about 20 min; Meanwhile, Cu(MeCN)$_4$BF$_4$ (94.4 mg, 0.3 mmol, 10 mol%) and (*S,S$_p$*)-**5a** (201.2 mg, 0.3 mmol, 10 mol%) were stirred in THF (20 mL) in a Schlenk flask under a nitrogen atmosphere at rt for 30 min. Benzoxazole ester **1a** (3.0 mmol, 1.0 equiv.) was added to the Schlenk flask containing copper complex and stirred for approximately 5 min. Propargylic carbonate **2a** (6.0 mmol, 2.0 equiv.) was then transformed into a Screw-cap reaction tube containing a nickel complex and stirred for an additional 5 min. The nickel complex solution was then combined with the copper complex solution, and the resulting solution was stirred for approximately 72 hours at 10 °C until substrate **1a** was completely consumed (monitored by TLC). The reaction mixture was subsequently concentrated under vacuum and purified by flash column chromatography on silica gel to afford the desired product **3a** (1031.0 mg, 92% yield, >20:1 dr, >99% ee).

2.3 General procedure for the synthesis of product 3

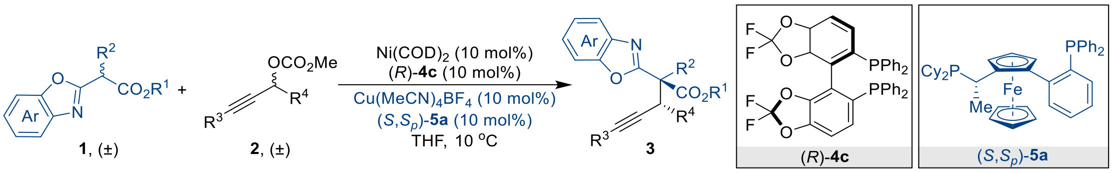

In a nitrogen-filled glove box, an oven-dried 10 mL screw-cap reaction tube equipped with a stir bar was charged with Ni(COD)$_2$ (4.1 mg, 0.015 mmol, 10 mol%) and (*R*)-**4c** (10.2 mg, 0.015 mmol, 10 mol%) in THF (1 mL) at rt for about 20 min; Meanwhile, Cu(MeCN)$_4$BF$_4$ (4.7 mg, 0.015 mmol, 10 mol%) and (*S,S$_p$*)-**5a** (10.1 mg, 0.015 mmol, 10 mol%) were stirred in THF (1 mL) in a Schlenk flask under a nitrogen atmosphere at rt for 30 min. Benzoxazole ester **1** (0.15 mmol, 1.0 equiv.) was added to the Schlenk flask containing copper complex and stirred for approximately 5 min. Propargylic carbonate **2** (0.3 mmol, 2.0 equiv.) was then transformed into a Screw-cap reaction tube containing a nickel complex and stirred for an additional 5 min. The nickel complex solution was then combined with the copper complex solution, and the resulting solution was stirred for approximately 72 hours at 10 °C until substrate **1** was completely consumed (monitored by TLC). The reaction mixture was subsequently concentrated under vacuum and purified by flash column chromatography on silica gel to afford the desired product **3**.

Ethyl (*S*)-2-(benzo[d]oxazol-2-yl)-2-((*R*)-4-phenylbut-3-yn-2-yl)pent-4-enoate (3a)

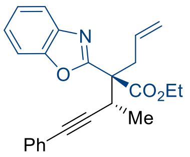

Colorless oil (53.2 mg, 95% yield, >20:1 dr). R$_f$ = 0.40 (Hexane/EtOAc = 19/1). It was prepared according to the general procedure described above. **^{1}H NMR (500 MHz, CDCl$_3$)** δ 7.78 – 7.74 (m, 1H), 7.56 – 7.51 (m, 1H), 7.41 – 7.37 (m, 2H), 7.36 – 7.32 (m, 2H), 7.31 – 7.27 (m, 3H), 5.86 – 5.76 (m, 1H), 5.17 – 5.03 (m, 2H), 4.34 – 4.26 (m, 2H), 3.75 (q, *J* = 7.0 Hz, 1H), 3.28 – 3.19 (m, 1H), 3.12 – 3.05 (m, 1H), 1.41 (d, *J* = 7.0 Hz, 3H), 1.27 (t, *J* = 7.1 Hz, 3H). **^{13}C NMR (125 MHz, CDCl$_3$)** δ 169.84, 164.58, 150.95, 140.65, 132.54, 131.69, 128.30, 127.96, 125.18, 124.38, 123.67, 120.30, 119.48, 110.76, 90.11, 83.55, 61.82, 56.90, 39.80, 32.26, 17.17, 14.37.

ESI-MS: calculated $[C_{24}H_{23}NO_3 + Na]^+$: 396.1570, found: 396.1578. $[\alpha]^{20}_D$ = -83.3 (c = 0.66, CH_2Cl_2). The product was analyzed by HPLC to determine the enantiomeric excess: >99% ee (CHIRALPAK IC, hexane/*i*-PrOH = 99/1, detector: 254 nm, T = 25 °C, flow rate: 0.5 mL/min), t_1(minor) = 15.8 min, t_2(major) = 26.6 min.

Tert-butyl (*S*)-2-(benzo[d]oxazol-2-yl)-2-((*R*)-4-phenylbut-3-yn-2-yl)pent-4-enoate (3b)

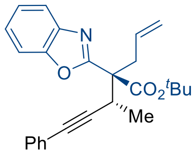

Colorless oil (57.7 mg, 96% yield, 10:1 dr). R_f = 0.50 (Hexane/EtOAc = 19/1). It was prepared according to the general procedure described above. **^{1}H NMR (500 MHz, $CDCl_3$)** δ 7.77 – 7.73 (m, 1H), 7.54 – 7.51 (m, 1H), 7.42 – 7.38 (m, 2H), 7.35 – 7.31 (m, 2H), 7.30 – 7.26 (m, 3H), 5.91 – 5.79 (m, 1H), 5.18 – 5.12 (m, 1H), 5.08 – 5.03 (m, 1H), 3.69 (q, *J* = 7.0 Hz, 1H), 3.21 (dd, *J* = 14.1, 7.0 Hz, 1H), 3.03 (dd, *J* = 14.2, 7.7 Hz, 1H), 1.48 (s, 9H), 1.40 (d, *J* = 7.0 Hz, 3H). **^{13}C NMR (125 MHz, $CDCl_3$)** δ 168.79, 164.98, 150.94, 140.68, 132.81, 131.64, 128.31, 127.88, 125.04, 124.26, 123.82, 120.22, 119.27, 110.69, 90.57, 83.30, 82.68, 57.27, 39.89, 32.28, 28.16, 17.10. **ESI-MS:** calculated $[C_{26}H_{27}NO_3 + Na]^+$: 424.1883, found: 424.1888. $[\alpha]^{20}_D$ = -75.4 (c = 1.00, CH_2Cl_2). The product was analyzed by HPLC to determine the enantiomeric excess: >99% ee (CHIRALPAK IE, hexane/*i*-PrOH = 99/1, detector: 254 nm, T = 25 °C, flow rate: 0.5 mL/min), t_1(minor) = 12.6 min, t_2(major) = 13.6 min.

Methyl (2*S*,3*R*)-2-(benzo[d]oxazol-2-yl)-2,3-dimethyl-5-phenylpent-4-ynoate (3c)

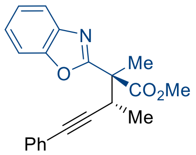

Colorless oil (48.2 mg, 96% yield, 19:1 dr). R_f = 0.30 (Hexane/EtOAc = 19/1). It was prepared according to the general procedure described above. **^{1}H NMR (500 MHz, $CDCl_3$)** δ 7.78 – 7.73 (m, 1H), 7.57 – 7.51 (m, 1H), 7.36 – 7.32 (m, 2H), 7.23 – 7.15 (m, 5H), 3.98 (q, *J* = 7.0 Hz, 1H), 3.75 (s, 3H), 1.92 (s, 3H), 1.38 (d, *J* = 7.0 Hz, 3H). **^{13}C NMR (125 MHz, $CDCl_3$)** δ 171.26, 165.80, 151.07, 141.00, 131.66, 128.17, 127.93, 125.22, 124.46, 123.29, 120.35, 110.85, 89.70, 83.35, 53.10, 52.91, 33.55, 17.42, 16.77. **ESI-MS:** calculated $[C_{21}H_{19}NO_3 + H]^+$: 334.1438, found: 334.1444. $[\alpha]^{20}_D$ = 118.2 (c = 0.94, CH_2Cl_2). The product was analyzed by HPLC to determine the enantiomeric excess: >99% ee (CHIRALPAK IC, hexane/*i*-PrOH = 99/1, detector: 254 nm, T = 25 °C, flow rate: 0.5 mL/min), t_1(major) = 19.2 min, t_2(minor) = 29.3 min.

Ethyl (2*S*,3*R*)-2-(benzo[d]oxazol-2-yl)-2,3-dimethyl-5-phenylpent-4-ynoate (3d)

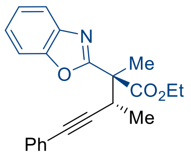

Colorless oil (49.0 mg, 94% yield, 18:1 dr). R_f = 0.40 (Hexane/EtOAc = 19/1). It was prepared according to the general procedure described above. **^{1}H NMR (500 MHz, $CDCl_3$)** δ 7.79 – 7.73 (m, 1H), 7.57 – 7.50 (m, 1H), 7.36 – 7.32 (m, 2H), 7.24 – 7.15 (m, 5H), 4.22 (q, *J* = 7.1 Hz, 2H), 3.98 (q, *J* = 7.0 Hz, 1H), 1.91 (s, 3H), 1.39 (d, *J* = 7.0 Hz, 3H), 1.23 (t, *J* = 7.1 Hz, 3H). **^{13}C NMR (125 MHz, $CDCl_3$)** δ 170.70, 165.94, 151.06, 141.01, 131.64, 128.17, 127.90, 125.15, 124.40, 123.34, 120.32, 110.81, 89.85, 83.28, 62.08, 52.91, 33.49, 17.46, 16.73, 14.18. **ESI-MS:** calculated $[C_{22}H_{21}NO_3 + Na]^+$: 370.1414, found: 370.1423. $[\alpha]^{20}_D$ = 112.7 (c = 0.98, CH_2Cl_2). The product was analyzed by HPLC to determine the enantiomeric excess: >99% ee (CHIRALPAK IC, hexane/*i*-PrOH = 99/1, detector: 254 nm, T = 25 °C, flow rate: 0.5 mL/min), t_1(major) = 18.9 min, t_2(minor) = 30.2 min.

Benzyl (2*S*,3*R*)-2-(benzo[d]oxazol-2-yl)-2,3-dimethyl-5-phenylpent-4-ynoate (3e)

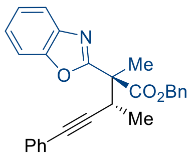

Colorless oil (53.0 mg, 86% yield, >20:1 dr). R_f = 0.40 (Hexane/EtOAc = 19/1). It was prepared according to the general procedure described above. **^{1}H NMR (500 MHz, $CDCl_3$)** δ 7.78 – 7.73 (m, 1H), 7.54 – 7.48 (m, 1H), 7.36 – 7.31 (m, 2H), 7.28 – 7.25 (m, 5H), 7.22 – 7.15 (m, 5H), 5.20 (s, 2H), 3.98 (q, J = 7.0 Hz, 1H), 1.93 (s, 3H), 1.33 (d, J = 7.0 Hz, 3H). **^{13}C NMR (125 MHz, $CDCl_3$)** δ 170.58, 165.73, 151.06, 140.97, 135.43, 131.67, 128.59, 128.37, 128.17, 128.02, 127.93, 125.22, 124.45, 123.29, 120.35, 110.80, 89.75, 83.39, 67.55, 53.10, 33.52, 17.57, 16.70. **ESI-MS:** calculated $[C_{27}H_{23}NO_3 + Na]^+$: 432.1570, found: 432.1579. $[\alpha]^{20}_D$ = 77.8 (c = 1.00, CH_2Cl_2). The product was analyzed by HPLC to determine the enantiomeric excess: >99% ee (CHIRALPAK IC, hexane/*i*-PrOH = 99/1, detector: 254 nm, T = 25 °C, flow rate: 0.5 mL/min), t_1(major) = 20.8 min, t_2(minor) = 31.7 min.

Ethyl (2*S*,3*R*)-2-(benzo[d]oxazol-2-yl)-2-ethyl-3-methyl-5-phenylpent-4-ynoate (3f)

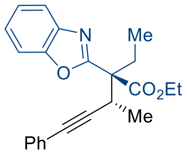

Colorless oil (48.0 mg, 89% yield, 14:1 dr). R_f = 0.40 (Hexane/EtOAc = 19/1). It was prepared according to the general procedure described above. **^{1}H NMR (500 MHz, $CDCl_3$)** δ 7.79 – 7.75 (m, 1H), 7.56 – 7.51 (m, 1H), 7.40 – 7.36 (m, 2H), 7.36 – 7.33 (m, 2H), 7.30 – 7.26 (m, 3H), 4.33 – 4.27 (m, 2H), 3.76 (q, J = 7.0 Hz, 1H), 2.56 – 2.48 (m, 1H), 2.41 – 2.31 (m, 1H), 1.38 (d, J = 7.1 Hz, 3H), 1.28 (t, J = 7.1 Hz, 3H), 0.97 (t, J = 7.5 Hz, 3H). **^{13}C NMR (125 MHz, $CDCl_3$)** δ 170.36, 164.96, 150.96, 140.70, 131.70, 128.28, 127.91, 125.10, 124.32, 123.75, 120.25, 110.77, 90.29, 83.23, 61.66, 57.50, 32.32, 28.76, 17.20, 14.38, 9.42. **ESI-MS:** calculated $[C_{23}H_{23}NO_3 + Na]^+$: 384.1570, found: 384.1573. $[\alpha]^{20}_D$ = -94.0 (c = 0.86, CH_2Cl_2). The product was analyzed by HPLC to determine the enantiomeric excess: >99% ee (CHIRALPAK IC, hexane/*i*-PrOH = 99/1, detector: 254 nm, T = 25 °C, flow rate: 0.5 mL/min), t_1(minor) = 24.4 min, t_2(major) = 28.3 min.

Ethyl (2*S*,3*R*)-2-(benzo[d]oxazol-2-yl)-3-methyl-5-phenyl-2-propylpent-4-ynoate (3g)

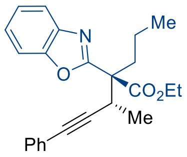

Colorless oil (50.8 mg, 90% yield, >20:1 dr). R_f = 0.45 (Hexane/EtOAc = 19/1). It was prepared according to the general procedure described above. **^{1}H NMR (500 MHz, $CDCl_3$)** δ 7.80 – 7.74 (m, 1H), 7.56 – 7.51 (m, 1H), 7.41 – 7.37 (m, 2H), 7.36 – 7.32 (m, 2H), 7.31 – 7.26 (m, 3H), 4.34 – 4.25 (m, 2H), 3.77 (q, J = 7.0 Hz, 1H), 2.47 – 2.40 (m, 1H), 2.33 – 2.25 (m, 1H), 1.51 – 1.44 (m, 1H), 1.38 (d, J = 7.0 Hz, 3H), 1.27 (t, J = 7.1 Hz, 3H), 1.24 – 1.15 (m, 1H), 0.95 (t, J = 7.3 Hz, 3H). **^{13}C NMR (125 MHz, $CDCl_3$)** δ 170.41, 165.15, 150.92, 140.71, 131.69, 128.28, 127.90, 125.08, 124.31, 123.76, 120.24, 110.77, 90.32, 83.22, 61.64, 57.05, 37.74, 32.53, 18.14, 17.26, 14.54, 14.36. **ESI-MS:** calculated $[C_{24}H_{25}NO_3 + Na]^+$: 398.1727, found: 398.1732. $[\alpha]^{20}_D$ = -95.9 (c = 1.00, CH_2Cl_2). The product was analyzed by HPLC to determine the enantiomeric excess: >99% ee (CHIRALPAK IC, hexane/*i*-PrOH = 99/1, detector: 280 nm, T = 25 °C, flow rate: 0.5 mL/min), t_1(minor) = 15.6 min, t_2(major) = 26.5 min.

Ethyl (*S*)-2-(benzo[d]oxazol-2-yl)-2-((*R*)-4-phenylbut-3-yn-2-yl)hexanoate (3h)

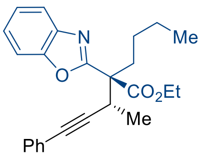

Colorless oil (53.6 mg, 92% yield, >20:1 dr). R_f = 0.45 (Hexane/EtOAc = 19/1). It was prepared according to the general procedure described above. **^{1}H NMR (500 MHz, CDCl$_3$)** δ 7.80 – 7.74 (m, 1H), 7.56 – 7.50 (m, 1H), 7.41 – 7.36 (m, 2H), 7.36 – 7.32 (m, 2H), 7.31 – 7.26 (m, 3H), 4.34 – 4.26 (m, 2H), 3.82 – 3.74 (m, 1H), 2.50 – 2.42 (m, 1H), 2.35 – 2.27 (m, 1H), 1.45 – 1.33 (m, 6H), 1.27 (t, *J* = 7.1 Hz, 3H), 1.18 – 1.09 (m, 1H), 0.89 (t, *J* = 7.3 Hz, 3H). **^{13}C NMR (125 MHz, CDCl$_3$)** δ 170.43, 165.17, 150.93, 140.72, 131.69, 128.28, 127.89, 125.08, 124.30, 123.78, 120.26, 110.77, 90.34, 83.23, 61.64, 56.97, 35.33, 32.41, 26.78, 23.10, 17.30, 14.37, 14.00. **ESI-MS:** calculated [$C_{25}H_{27}NO_3$ + Na]$^+$: 412.1883, found: 412.1894. $[\alpha]^{20}_D$ = -91.9 (c = 1.02, CH_2Cl_2). The product was analyzed by HPLC to determine the enantiomeric excess: >99% ee (CHIRALPAK IE, hexane/*i*-PrOH = 99/1, detector: 280 nm, T = 25 °C, flow rate: 0.5 mL/min), t_1(minor) = 16.8 min, t_2(major) = 17.8 min.

Ethyl (*S*)-2-(benzo[d]oxazol-2-yl)-2-((*R*)-4-phenylbut-3-yn-2-yl)octanoate (3i)

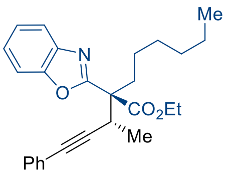

Colorless oil (61.0 mg, 97% yield, >20:1 dr). R_f = 0.50 (Hexane/EtOAc = 19/1). It was prepared according to the general procedure described above. **^{1}H NMR (500 MHz, CDCl$_3$)** δ 7.80 – 7.75 (m, 1H), 7.56 – 7.51 (m, 1H), 7.40 – 7.37 (m, 2H), 7.36 – 7.32 (m, 2H), 7.30 – 7.27 (m, 3H), 4.33 – 4.26 (m, 2H), 3.77 (q, *J* = 7.0 Hz, 1H), 2.49 – 2.41 (m, 1H), 2.34 – 2.26 (m, 1H), 1.47 – 1.41 (m, 1H), 1.38 (d, *J* = 7.0 Hz, 3H), 1.35 – 1.30 (m, 2H), 1.29 – 1.23 (m, 7H), 1.19 – 1.10 (m, 1H), 0.86 – 0.82 (m, 3H). **^{13}C NMR (125 MHz, CDCl$_3$)** δ 170.44, 165.17, 150.93, 140.73, 131.69, 128.28, 127.89, 125.08, 124.30, 123.78, 120.26, 110.76, 90.35, 83.22, 61.64, 57.01, 35.56, 32.42, 31.60, 29.64, 24.55, 22.68, 17.29, 14.37, 14.14. **ESI-MS:** calculated [$C_{27}H_{31}NO_3$ + Na]$^+$: 440.2196, found: 440.2202. $[\alpha]^{20}_D$ = -80.7 (c = 0.97, CH_2Cl_2). The product was analyzed by HPLC to determine the enantiomeric excess: >99% ee (CHIRALPAK AD-H, hexane/*i*-PrOH = 99/1, detector: 280 nm, T = 25 °C, flow rate: 1.0 mL/min), t_1(minor) = 8.3 min, t_2(major) = 14.4 min.

Ethyl (2*S*,3*R*)-2-(benzo[d]oxazol-2-yl)-2-(3-((tert-butyldimethylsilyl)oxy)propyl)-3 -methyl-5-phenylpent-4-ynoate (3j)

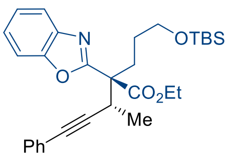

Colorless oil (68.8 mg, 91% yield, >20:1 dr). R_f = 0.45 (Hexane/EtOAc = 19/1). It was prepared according to the general procedure described above. **^{1}H NMR (500 MHz, CDCl$_3$)** δ 7.78 – 7.73 (m, 1H), 7.54 – 7.50 (m, 1H), 7.40 – 7.36 (m, 2H), 7.35 – 7.32 (m, 2H), 7.30 – 7.26 (m, 3H), 4.33 – 4.25 (m, 2H), 3.78 (q, *J* = 7.0 Hz, 1H), 3.66 – 3.58 (m, 2H), 2.51 – 2.36 (m, 2H), 1.73 – 1.67 (m, 1H), 1.48 – 1.42 (m, 1H), 1.40 (d, *J* = 7.0 Hz, 3H), 1.27 (t, *J* = 7.1 Hz, 3H), 0.85 (s, 9H), 0.02 – -0.01 (m, 6H). **^{13}C NMR (125 MHz, CDCl$_3$)** δ 170.28, 164.97, 150.94, 140.73, 131.71, 128.26, 127.91, 125.10, 124.32, 123.72, 120.27, 110.78, 90.16, 83.32, 63.13, 61.71, 56.75, 32.64, 32.08, 28.17, 26.03, 18.41, 17.32, 14.36, -5.21. **ESI-MS:** calculated [$C_{30}H_{39}NO_4Si$ + Na]$^+$: 528.2541, found: 528.2551. $[\alpha]^{20}_D$ = -63.4 (c = 0.94, CH_2Cl_2). The product was analyzed by HPLC to determine the enantiomeric excess: >99% ee (CHIRALPAK IE, hexane/*i*-PrOH = 99/1, detector: 280 nm, T = 25 °C, flow rate: 0.5 mL/min), t_1(major) = 13.5 min, t_2(minor) = 15.3 min.

Ethyl (2S,3R)-2-(benzo[d]oxazol-2-yl)-2-(3-methoxypropyl)-3-methyl-5-phenylpent-4-ynoate (3k)

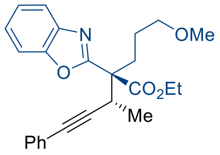

Colorless oil (54.7 mg, 90% yield, >20:1 dr). R_f = 0.40 (Hexane/EtOAc = 9/1). Prepared according to the above general procedure, but the reaction was performed at 20 °C. **^{1}H NMR (500 MHz, CDCl$_3$)** δ 7.79 – 7.74 (m, 1H), 7.55 – 7.51 (m, 1H), 7.40 – 7.36 (m, 2H), 7.36 – 7.32 (m, 2H), 7.30 – 7.27 (m, 3H), 4.33 – 4.26 (m, 2H), 3.78 (q, J = 7.0 Hz, 1H), 3.43 – 3.37 (m, 2H), 3.29 (s, 3H), 2.54 – 2.47 (m, 1H), 2.43 – 2.34 (m, 1H), 1.79 – 1.73 (m, 1H), 1.53 – 1.46 (m, 1H), 1.39 (d, J = 7.0 Hz, 3H), 1.27 (t, J = 7.1 Hz, 3H). **^{13}C NMR (125 MHz, CDCl$_3$)** δ 170.24, 164.84, 150.94, 140.68, 131.69, 128.28, 127.93, 125.13, 124.34, 123.68, 120.27, 110.79, 90.12, 83.37, 72.62, 61.76, 58.55, 56.74, 32.70, 32.26, 24.96, 17.27, 14.35. **ESI-MS:** calculated [$C_{25}H_{27}NO_4$ + Na]$^+$: 428.1832, found: 428.1837. $[\alpha]^{20}_D$ = -83.7 (c = 1.08, CH_2Cl_2). The product was analyzed by HPLC to determine the enantiomeric excess: >99% ee (CHIRALPAK IC, hexane/i-PrOH = 98/2, detector: 280 nm, T = 25 °C, flow rate: 1.0 mL/min), t_1(major) = 16.2 min, t_2(minor) = 17.4 min.

Ethyl (2R,3R)-2-(benzo[d]oxazol-2-yl)-2-fluoro-3-methyl-5-phenylpent-4-ynoate (3l)

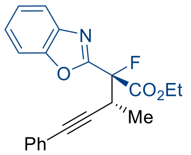

Colorless oil (50.4 mg, 96% yield, >20:1 dr). R_f = 0.30 (Hexane/EtOAc = 19/1). It was prepared according to the general procedure described above. **^{1}H NMR (500 MHz, CDCl$_3$)** δ 7.85 – 7.79 (m, 1H), 7.62 – 7.57 (m, 1H), 7.43 – 7.36 (m, 2H), 7.27 – 7.18 (m, 5H), 4.43 – 4.32 (m, 2H), 4.16 – 4.03 (m, 1H), 1.51 (d, J = 7.1 Hz, 3H), 1.33 (t, J = 7.1 Hz, 3H). **^{13}C NMR (125 MHz, CDCl$_3$)** δ 165.51 (d, J = 25.9 Hz), 159.28 (d, J = 25.9 Hz), 150.93, 140.61, 131.80, 128.24, 128.19, 126.27, 125.07, 122.85, 121.12, 111.27, 93.79 (d, J = 199.2 Hz), 86.46 (d, J = 2.4 Hz), 84.15, 63.35, 34.16 (d, J = 21.9 Hz), 15.57 (d, J = 3.6 Hz), 14.19. **^{19}F NMR (470 MHz, CDCl$_3$)** δ -168.9 (s). **ESI-MS:** calculated [$C_{21}H_{18}FNO_3$ + Na]$^+$: 374.1163, found: 374.1171. $[\alpha]^{20}_D$ = 66.9 (c = 0.98, CH_2Cl_2). The product was analyzed by HPLC to determine the enantiomeric excess: >99% ee (CHIRALPAK IF, hexane/i-PrOH = 99/1, detector: 254 nm, T = 25 °C, flow rate: 0.5 mL/min), t_1(minor) = 24.8 min, t_2(major) = 28.9 min.

Tert-butyl (2S,3R)-2-(benzo[d]oxazol-2-yl)-3-methyl-2-phenethyl-5-phenylpent-4-ynoate (3m)

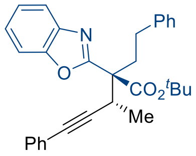

Colorless oil (66.5 mg, 95% yield, 12:1 dr). R_f = 0.55 (Hexane/EtOAc = 19/1). It was prepared according to the general procedure described above. **^{1}H NMR (500 MHz, CDCl$_3$)** δ 7.79 – 7.75 (m, 1H), 7.57 – 7.53 (m, 1H), 7.42 – 7.38 (m, 2H), 7.36 – 7.32 (m, 2H), 7.30 – 7.27 (m, 3H), 7.26 – 7.23 (m, 2H), 7.22 – 7.19 (m, 2H), 7.18 – 7.14 (m, 1H), 3.80 (q, J = 7.0 Hz, 1H), 2.90 – 2.83 (m, 1H), 2.78 – 2.70 (m, 1H), 2.58 – 2.45 (m, 2H), 1.51 (s, 9H), 1.42 (d, J = 7.0 Hz, 3H). **^{13}C NMR (125 MHz, CDCl$_3$)** δ 169.15, 165.09, 150.98, 141.79, 140.76, 131.67, 128.59, 128.52, 128.33, 127.92, 126.10, 125.07, 124.28, 123.79, 120.28, 110.75, 90.50, 83.25, 82.68, 57.46, 37.97, 32.79, 31.41, 28.19, 17.32. **ESI-MS:** calculated [$C_{31}H_{31}NO_3$ + Na]$^+$: 488.2196, found: 488.2202. $[\alpha]^{20}_D$ = -72.3 (c = 0.98, CH_2Cl_2). The product was analyzed by HPLC to determine the enantiomeric excess: >99% ee (CHIRALPAK AD-H, hexane/i-PrOH = 99/1, detector: 254 nm, T = 25 °C, flow rate: 1.0 mL/min), t_1(major) = 6.7 min, t_2(minor) = 10.4 min.

Ethyl (2*R*,3*R*)-2-(benzo[d]thiazol-2-yl)-2,3-dimethyl-5-phenylpent-4-ynoate (3n)

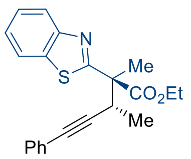

Colorless oil (45.7 mg, 84% yield, 13:1 dr). R_f = 0.45 (Hexane/EtOAc = 19/1). It was prepared according to the general procedure described above. **^{1}H NMR (400 MHz, $CDCl_3$)** δ 8.05 (d, *J* = 8.1 Hz, 1H), 7.87 (d, *J* = 7.9 Hz, 1H), 7.48 – 7.42 (m, 1H), 7.38 – 7.33 (m, 1H), 7.29 – 7.18 (m, 5H), 4.24 (q, *J* = 7.1 Hz, 2H), 3.91 (q, *J* = 6.9 Hz, 1H), 1.94 (s, 3H), 1.35 (d, *J* = 6.9 Hz, 3H), 1.25 (t, *J* = 7.1 Hz, 3H). **^{13}C NMR (100 MHz, $CDCl_3$)** δ 172.22, 172.09, 152.45, 135.61, 131.62, 128.22, 127.96, 125.92, 125.16, 123.38, 121.51, 90.32, 84.08, 61.98, 56.46, 35.74, 19.05, 17.06, 14.17. **ESI-MS:** calculated $[C_{22}H_{21}NO_2S + H]^+$: 364.1366, found: 364.1371. $[\alpha]^{20}_D$ = 84.7 (c = 0.95, CH_2Cl_2). The product was analyzed by HPLC to determine the enantiomeric excess: >99% ee (CHIRALPAK IC, hexane/*i*-PrOH = 99/1, detector: 254 nm, T = 25 °C, flow rate: 0.5 mL/min), t_1(major) = 18.3 min, t_2(minor) = 28.5 min.

Methyl (2*R*,3*R*)-2-fluoro-3-methyl-5-phenyl-2-(pyridin-2-yl)pent-4-ynoate (3o)

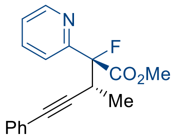

Colorless oil (31.5 mg, 71% yield, 5:1 dr). R_f = 0.45 (Hexane/EtOAc = 4/1). Prepared according to the above general procedure, but the reaction was performed at 20 °C. **^{1}H NMR (400 MHz, $CDCl_3$)** δ 8.67 – 8.60 (m, 1H), 7.80 – 7.70 (m, 2H), 7.31 – 7.25 (m, 2H), 7.21 – 7.11 (m, 4H), 4.13 (dq, *J* = 29.8, 7.0 Hz, 1H), 3.82 (s, 3H), 1.48 (d, *J* = 7.1 Hz, 3H). **^{13}C NMR (100 MHz, $CDCl_3$)** δ 168.46 (d, *J* = 25.6 Hz), 156.38 (d, *J* = 27.4 Hz), 149.23 (d, *J* = 2.7 Hz), 136.80 (d, *J* = 1.7 Hz), 131.58, 128.14, 127.94, 123.63, 123.21, 120.45 (d, *J* = 9.5 Hz), 98.71 (d, *J* = 197.3 Hz), 88.08, 83.46, 53.29, 34.45 (d, *J* = 21.2 Hz), 15.52 (d, *J* = 4.5 Hz). **^{19}F NMR (375 MHz, $CDCl_3$)** δ -177.4 (s). **ESI-MS:** calculated $[C_{18}H_{16}FNO_2 + H]^+$: 298.1238, found: 298.1248. $[\alpha]^{20}_D$ = 50.3 (c = 1.03, CH_2Cl_2). The product was analyzed by HPLC to determine the enantiomeric excess: >99% ee (CHIRALPAK OJ-H, hexane/*i*-PrOH = 95/5, detector: 254 nm, T = 25 °C, flow rate: 1.0 mL/min), t_1(minor) = 14.6 min, t_2(major) = 15.5 min.

Ethyl (*S*)-2-(benzo[d]oxazol-2-yl)-2-((*R*)-4-(4-fluorophenyl)but-3-yn-2-yl)pent-4-enoate (3p)

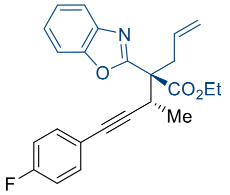

Colorless oil (53.5 mg, 91% yield, >20:1 dr). R_f = 0.45 (Hexane/EtOAc = 19/1). It was prepared according to the general procedure described above. **^{1}H NMR (500 MHz, $CDCl_3$)** δ 7.80 – 7.72 (m, 1H), 7.56 – 7.50 (m, 1H), 7.38 – 7.32 (m, 4H), 7.01 – 6.93 (m, 2H), 5.87 – 5.75 (m, 1H), 5.18 – 5.11 (m, 1H), 5.09 – 5.02 (m, 1H), 4.34 – 4.23 (m, 2H), 3.73 (q, *J* = 7.0 Hz, 1H), 3.23 (dd, *J* = 14.2, 7.1 Hz, 1H), 3.08 (dd, *J* = 14.2, 7.5 Hz, 1H), 1.40 (d, *J* = 7.0 Hz, 3H), 1.26 (t, *J* = 7.1 Hz, 3H). **^{13}C NMR (125 MHz, $CDCl_3$)** δ 169.83, 164.51, 162.36 (d, *J* = 247.4 Hz), 150.93, 140.64, 133.49 (d, *J* = 8.2 Hz), 132.47, 125.21, 124.41, 120.31, 119.70 (d, *J* = 3.4 Hz), 119.51, 115.53 (d, *J* = 21.9 Hz), 110.73, 89.79, 82.49, 61.83, 56.83, 39.73, 32.19, 17.10, 14.35. **^{19}F NMR (470 MHz, $CDCl_3$)** δ -111.7 (s). **ESI-MS:** calculated $[C_{24}H_{22}FNO_3 + Na]^+$: 414.1476, found: 414.1484. $[\alpha]^{20}_D$ = -71.3 (c = 1.02, CH_2Cl_2). The product was analyzed by HPLC to determine the enantiomeric excess: >99% ee (CHIRALPAK IC, hexane/*i*-PrOH = 99/1, detector: 254 nm, T = 25 °C, flow rate: 0.5 mL/min), t_1(minor) = 15.3 min, t_2(major) = 27.4 min.

Ethyl (*S*)-2-(benzo[d]oxazol-2-yl)-2-((*R*)-4-(4-chlorophenyl)but-3-yn-2-yl)pent-4-enoate (3q)

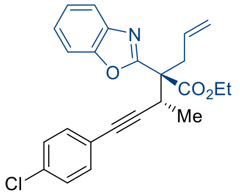

Colorless oil (55.6 mg, 91% yield, 18:1 dr). R_f = 0.45 (Hexane/EtOAc = 19/1). It was prepared according to the general procedure described above. **^{1}H NMR (500 MHz, $CDCl_3$)** δ 7.80 – 7.73 (m, 1H), 7.55 – 7.49 (m, 1H), 7.37 – 7.32 (m, 2H), 7.32 – 7.27 (m, 2H), 7.27 – 7.24 (m, 2H), 5.87 – 5.75 (m, 1H), 5.18 – 5.11 (m, 1H), 5.09 – 5.03 (m, 1H), 4.32 – 4.24 (m, 2H), 3.73 (q, *J* = 7.0 Hz, 1H), 3.22 (dd, *J* = 14.2, 7.1 Hz, 1H), 3.08 (dd, *J* = 14.2, 7.5 Hz, 1H), 1.40 (d, *J* = 7.1 Hz, 3H), 1.26 (t, *J* = 7.1 Hz, 3H). **^{13}C NMR (125 MHz, $CDCl_3$)** δ 169.80, 164.44, 150.93, 140.62, 133.93, 132.90, 132.41, 128.61, 125.23, 124.42, 122.13, 120.30, 119.55, 110.73, 91.24, 82.47, 61.85, 56.79, 39.69, 32.21, 17.01, 14.35. **ESI-MS:** calculated $[C_{24}H_{22}ClNO_3 + Na]^+$: 430.1180, found: 430.1187. $[\alpha]^{20}_D$ = -83.0 (c = 1.04, CH_2Cl_2). The product was analyzed by HPLC to determine the enantiomeric excess: >99% ee (CHIRALPAK IG, hexane/*i*-PrOH = 99/1, detector: 254 nm, T = 25 °C, flow rate: 0.5 mL/min), t_1(major) = 24.6 min, t_2(minor) = 29.3 min.

Ethyl (*S*)-2-(benzo[d]oxazol-2-yl)-2-((*R*)-4-(4-bromophenyl)but-3-yn-2-yl)pent-4-enoate (3r)

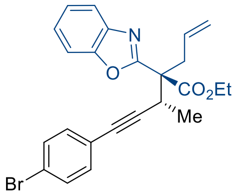

Colorless oil (59.1 mg, 87% yield, >20:1 dr). R_f = 0.45 (Hexane/EtOAc = 19/1). It was prepared according to the general procedure described above. **^{1}H NMR (500 MHz, $CDCl_3$)** δ 7.78 – 7.74 (m, 1H), 7.54 – 7.49 (m, 1H), 7.43 – 7.39 (m, 2H), 7.36 – 7.33 (m, 2H), 7.25 – 7.20 (m, 2H), 5.85 – 5.75 (m, 1H), 5.17 – 5.11 (m, 1H), 5.08 – 5.04 (m, 1H), 4.33 – 4.24 (m, 2H), 3.73 (q, *J* = 7.0 Hz, 1H), 3.22 (dd, *J* = 14.2, 7.1 Hz, 1H), 3.07 (dd, *J* = 14.2, 7.5 Hz, 1H), 1.40 (d, *J* = 7.1 Hz, 3H), 1.26 (t, *J* = 7.1 Hz, 3H). **^{13}C NMR (125 MHz, $CDCl_3$)** δ 169.79, 164.43, 150.93, 140.62, 133.14, 132.40, 131.54, 125.24, 124.42, 122.60, 122.12, 120.31, 119.56, 110.73, 91.46, 82.53, 61.86, 56.78, 39.67, 32.22, 16.98, 14.36. **ESI-MS:** calculated $[C_{24}H_{22}BrNO_3 + Na]^+$: 474.0675, found: 474.0683. $[\alpha]^{20}_D$ = -73.8 (c = 0.97, CH_2Cl_2). The product was analyzed by HPLC to determine the enantiomeric excess: >99% ee (CHIRALPAK IG, hexane/*i*-PrOH = 99/1, detector: 254 nm, T = 25 °C, flow rate: 0.5 mL/min), t_1(major) = 26.0 min, t_2(minor) = 31.3 min.

Ethyl (*S*)-2-(benzo[d]oxazol-2-yl)-2-((*R*)-4-(p-tolyl)but-3-yn-2-yl)pent-4-enoate (3s)

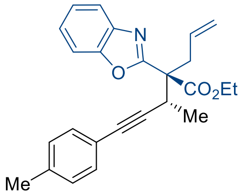

Colorless oil (51.0 mg, 88% yield, 18:1 dr). R_f = 0.45 (Hexane/EtOAc = 19/1). It was prepared according to the general procedure described above. **^{1}H NMR (500 MHz, $CDCl_3$)** δ 7.83 – 7.76 (m, 1H), 7.60 – 7.53 (m, 1H), 7.39 – 7.35 (m, 2H), 7.33 – 7.29 (m, 2H), 7.14 – 7.10 (m, 2H), 5.89 – 5.80 (m, 1H), 5.20 – 5.13 (m, 1H), 5.09 – 5.05 (m, 1H), 4.36 – 4.27 (m, 2H), 3.77 (q, *J* = 7.0 Hz, 1H), 3.27 (dd, *J* = 14.2, 7.1 Hz, 1H), 3.11 (dd, *J* = 14.2, 7.5 Hz, 1H), 2.36 (s, 3H), 1.43 (d, *J* = 7.0 Hz, 3H), 1.29 (t, *J* = 7.1 Hz, 3H). **^{13}C NMR (125 MHz, $CDCl_3$)** δ 169.85, 164.63, 150.93, 140.65, 137.96, 132.59, 131.55, 129.04, 125.14, 124.34, 120.59, 120.27, 119.41, 110.75, 89.28, 83.61, 61.78, 56.94, 39.83, 32.32, 21.56, 17.23, 14.37. **ESI-MS:** calculated $[C_{25}H_{25}NO_3 + Na]^+$: 410.1727, found: 410.1725. $[\alpha]^{20}_D$ = -77.0 (c = 1.00, CH_2Cl_2). The product was analyzed by HPLC to determine the enantiomeric excess: >99% ee

(CHIRALPAK IE, hexane/*i*-PrOH = 99/1, detector: 254 nm, T = 25 °C, flow rate: 0.5 mL/min), t_1(minor) = 21.6 min, t_2(major) = 23.8 min.

Ethyl (*S*)-2-(benzo[d]oxazol-2-yl)-2-((*R*)-4-(4-(2-ethoxy-2-oxoethyl)phenyl)but-3-yn-2-yl) pent-4-enoate (3t)

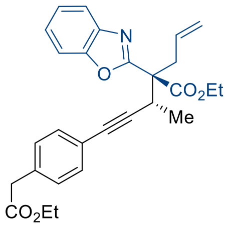

Colorless oil (62.2 mg, 90% yield, >20:1 dr). R_f = 0.40 (Hexane/EtOAc = 9/1). It was prepared according to the general procedure described above. **^{1}H NMR (400 MHz, CDCl$_3$)** δ 7.78 – 7.72 (m, 1H), 7.56 – 7.49 (m, 1H), 7.36 – 7.31 (m, 4H), 7.23 – 7.18 (m, 2H), 5.88 – 5.72 (m, 1H), 5.17 – 5.09 (m, 1H), 5.08 – 5.01 (m, 1H), 4.33 – 4.24 (m, 2H), 4.14 (q, *J* = 7.1 Hz, 2H), 3.74 (q, *J* = 7.0 Hz, 1H), 3.59 (s, 2H), 3.23 (dd, *J* = 14.2, 7.1 Hz, 1H), 3.08 (dd, *J* = 14.2, 7.5 Hz, 1H), 1.40 (d, *J* = 7.0 Hz, 3H), 1.25 (q, *J* = 7.3 Hz, 6H). **^{13}C NMR (100 MHz, CDCl$_3$)** δ 171.32, 169.80, 164.57, 150.94, 140.67, 134.00, 132.56, 131.83, 129.24, 125.15, 124.35, 122.48, 120.28, 119.41, 110.73, 90.25, 83.29, 61.78, 61.04, 56.89, 41.39, 39.75, 32.29, 17.14, 14.34, 14.26. **ESI-MS:** calculated $[C_{28}H_{29}NO_5 + Na]^+$: 482.1938, found: 482.1947. $[\alpha]^{20}_D$ = -69.6 (c = 1.04, CH_2Cl_2). The product was analyzed by HPLC to determine the enantiomeric excess: >99% ee (CHIRALPAK IC, hexane/*i*-PrOH = 98/2, detector: 254 nm, T = 25 °C, flow rate: 1.0 mL/min), t_1(minor) = 22.5 min, t_2(major) = 39.0 min.

Ethyl (*S*)-2-(benzo[d]oxazol-2-yl)-2-((*R*)-4-(4-methoxyphenyl)but-3-yn-2-yl)pent-4 -enoate (3u)

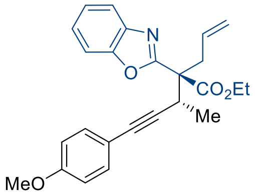

Colorless oil (53.5 mg, 88% yield, >20:1 dr). R_f = 0.25 (Hexane/EtOAc = 19/1). It was prepared according to the general procedure described above. **^{1}H NMR (500 MHz, CDCl$_3$)** δ 7.80 – 7.74 (m, 1H), 7.56 – 7.51 (m, 1H), 7.36 – 7.30 (m, 4H), 6.85 – 6.78 (m, 2H), 5.87 – 5.76 (m, 1H), 5.18 – 5.09 (m, 1H), 5.07 – 5.02 (m, 1H), 4.35 – 4.24 (m, 2H), 3.80 (s, 3H), 3.73 (q, *J* = 7.0 Hz, 1H), 3.23 (dd, *J* = 14.2, 7.1 Hz, 1H), 3.08 (dd, *J* = 14.2, 7.5 Hz, 1H), 1.40 (d, *J* = 7.0 Hz, 3H), 1.27 (t, *J* = 7.1 Hz, 3H). **^{13}C NMR (125 MHz, CDCl$_3$)** δ 169.86, 164.65, 159.36, 150.92, 140.65, 133.03, 132.62, 125.13, 124.34, 120.27, 119.38, 115.82, 113.90, 110.73, 88.48, 83.33, 61.77, 56.96, 55.38, 39.83, 32.34, 17.28, 14.37. **ESI-MS:** calculated $[C_{25}H_{25}NO_4 + Na]^+$: 426.1676, found: 426.1689. $[\alpha]^{20}_D$ = -78.5 (c = 1.02, CH_2Cl_2). The product was analyzed by HPLC to determine the enantiomeric excess: >99% ee (CHIRALPAK IC, hexane/*i*-PrOH = 99/1, detector: 254 nm, T = 25 °C, flow rate: 1.0 mL/min), t_1(minor) = 13.8 min, t_2(major) = 24.8 min.

Ethyl (*S*)-2-(benzo[d]oxazol-2-yl)-2-((*R*)-4-(m-tolyl)but-3-yn-2-yl)pent-4-enoate (3v)

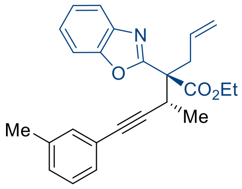

Colorless oil (51.2 mg, 88% yield, >20:1 dr). R_f = 0.40 (Hexane/EtOAc = 19/1). It was prepared according to the general procedure described above. **^{1}H NMR (500 MHz, CDCl$_3$)** δ 7.80 – 7.72 (m, 1H), 7.56 – 7.51 (m, 1H), 7.37 – 7.32 (m, 2H), 7.23 – 7.14 (m, 3H), 7.12 – 7.07 (m, 1H), 5.87 – 5.75 (m, 1H), 5.17 – 5.11 (m, 1H), 5.08 – 5.01 (m, 1H), 4.35 – 4.26 (m, 2H), 3.75 (q, *J* = 7.0 Hz, 1H), 3.25 (dd, *J* = 14.2, 7.1 Hz, 1H), 3.09 (dd, *J* = 14.2, 7.5 Hz, 1H), 2.31 (s, 3H), 1.42 (d, *J* = 7.0 Hz, 3H), 1.27 (t, *J* = 7.1 Hz, 3H). **^{13}C NMR (125 MHz, CDCl$_3$)** δ 169.84, 164.62, 150.94, 140.66, 137.92, 132.56, 132.30, 128.84, 128.73, 128.19,

125.15, 124.36, 123.45, 120.28, 119.45, 110.75, 89.66, 83.70, 61.80, 56.89, 39.79, 32.27, 21.32, 17.21, 14.35. **ESI-MS:** calculated $[C_{25}H_{25}NO_3 + Na]^+$: 410.1727, found: 410.1738. $[\alpha]^{20}_D$ = -72.9 (c = 1.00, CH_2Cl_2). The product was analyzed by HPLC to determine the enantiomeric excess: >99% ee (CHIRALPAK IG, hexane/*i*-PrOH = 99/1, detector: 254 nm, T = 25 °C, flow rate: 0.5 mL/min), t_1(major) = 24.0 min, t_2(minor) = 26.0 min.

Ethyl (*S*)-2-(benzo[d]oxazol-2-yl)-2-((*R*)-4-(2-chlorophenyl)but-3-yn-2-yl)pent-4-enoate (3w)

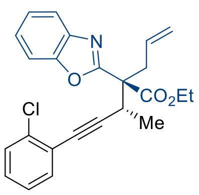

Colorless oil (52.9 mg, 86% yield, 12:1 dr). R_f = 0.35 (Hexane/EtOAc = 19/1). It was prepared according to the general procedure described above. **^{1}H NMR (500 MHz, $CDCl_3$)** δ 7.79 – 7.73 (m, 1H), 7.56 – 7.51 (m, 1H), 7.45 – 7.40 (m, 1H), 7.38 – 7.32 (m, 3H), 7.23 – 7.15 (m, 2H), 5.89 – 5.77 (m, 1H), 5.20 – 5.14 (m, 1H), 5.08 – 5.03 (m, 1H), 4.33 – 4.26 (m, 2H), 3.83 (q, *J* = 7.0 Hz, 1H), 3.30 (dd, *J* = 14.2, 7.1 Hz, 1H), 3.11 (dd, *J* = 14.2, 7.5 Hz, 1H), 1.45 (d, *J* = 7.0 Hz, 3H), 1.26 (t, *J* = 7.1 Hz, 3H). **^{13}C NMR (125 MHz, $CDCl_3$)** δ 169.77, 164.49, 150.97, 140.64, 135.98, 133.54, 132.50, 129.26, 128.97, 126.39, 125.16, 124.36, 123.49, 120.27, 119.57, 110.83, 95.59, 80.41, 61.88, 56.73, 39.71, 32.40, 17.13, 14.33. **ESI-MS:** calculated $[C_{24}H_{22}ClNO_3 + Na]^+$: 430.1180, found: 430.1193. $[\alpha]^{20}_D$ = -63.0 (c = 1.00, CH_2Cl_2). The product was analyzed by HPLC to determine the enantiomeric excess: >99% ee (CHIRALPAK IE, hexane/*i*-PrOH = 99/1, detector: 254 nm, T = 25 °C, flow rate: 0.5 mL/min), t_1(minor) = 23.5 min, t_2(major) = 24.7 min.

Ethyl (*S*)-2-(benzo[d]oxazol-2-yl)-2-((*R*)-4-(o-tolyl)but-3-yn-2-yl)pent-4-enoate (3x)

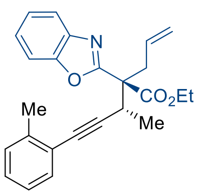

Colorless oil (52.8 mg, 91% yield, 12:1 dr). R_f = 0.40 (Hexane/EtOAc = 19/1). It was prepared according to the general procedure described above. **^{1}H NMR (500 MHz, $CDCl_3$)** δ 7.78 – 7.74 (m, 1H), 7.54 – 7.50 (m, 1H), 7.38 – 7.33 (m, 3H), 7.22 – 7.15 (m, 2H), 7.14 – 7.09 (m, 1H), 5.91 – 5.80 (m, 1H), 5.19 – 5.12 (m, 1H), 5.09 – 5.04 (m, 1H), 4.28 (q, *J* = 7.1 Hz, 2H), 3.82 (q, *J* = 7.0 Hz, 1H), 3.27 (dd, *J* = 14.2, 7.0 Hz, 1H), 3.09 (dd, *J* = 14.2, 7.6 Hz, 1H), 2.41 (s, 3H), 1.43 (d, *J* = 7.0 Hz, 3H), 1.26 (t, *J* = 7.1 Hz, 3H). **^{13}C NMR (125 MHz, $CDCl_3$)** δ 169.86, 164.60, 150.95, 140.65, 140.22, 132.61, 132.09, 129.40, 127.95, 125.51, 125.16, 124.37, 123.42, 120.28, 119.44, 110.75, 93.99, 82.42, 61.81, 56.87, 39.76, 32.51, 20.82, 17.37, 14.34. **ESI-MS:** calculated $[C_{25}H_{25}NO_3 + Na]^+$: 410.1727, found: 410.1730. $[\alpha]^{20}_D$ = -63.3 (c = 1.00, CH_2Cl_2). The product was analyzed by HPLC to determine the enantiomeric excess: >99% ee (CHIRALPAK IC, hexane/*i*-PrOH = 99/1, detector: 254 nm, T = 25 °C, flow rate: 0.5 mL/min), t_1(minor) = 17.1 min, t_2(major) = 24.1 min.

Ethyl (*S*)-2-(benzo[d]oxazol-2-yl)-2-((*R*)-4-(thiophen-2-yl)but-3-yn-2-yl)pent-4-enoate (3y)

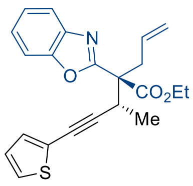

Colorless oil (51.2 mg, 90% yield, >20:1 dr). R_f = 0.35 (Hexane/EtOAc = 19/1). It was prepared according to the general procedure described above. **^{1}H NMR (500 MHz, $CDCl_3$)** δ 7.79 – 7.73 (m, 1H), 7.58 – 7.52 (m, 1H), 7.37 – 7.32 (m, 2H), 7.22 – 7.16 (m, 1H), 7.15 – 7.09 (m, 1H), 6.97 – 6.91 (m, 1H), 5.85 – 5.73 (m, 1H), 5.19 – 5.12 (m, 1H), 5.09 – 5.04 (m, 1H), 4.34 – 4.25 (m, 2H), 3.76 (q, *J* = 7.0 Hz, 1H), 3.21 (dd, *J* = 14.2, 7.1 Hz, 1H), 3.07 (dd, *J* = 14.2, 7.5

Hz, 1H), 1.39 (d, J = 7.0 Hz, 3H), 1.28 (t, J = 7.1 Hz, 3H). **^{13}C NMR (125 MHz, CDCl$_3$)** δ 169.76, 164.40, 150.96, 140.60, 132.38, 131.42, 126.91, 126.52, 125.20, 124.38, 123.71, 120.28, 119.58, 110.83, 94.14, 76.74, 61.88, 56.84, 39.72, 32.47, 16.91, 14.36. **ESI-MS:** calculated [$C_{22}H_{21}NO_3S$ + Na]$^+$: 402.1134, found: 402.1139. $[\alpha]^{20}_D$ = -74.4 (c = 1.00, CH_2Cl_2). The product was analyzed by HPLC to determine the enantiomeric excess: >99% ee (CHIRALPAK IC, hexane/*i*-PrOH = 99/1, detector: 254 nm, T = 25 °C, flow rate: 0.5 mL/min), t_1(minor) = 22.4 min, t_2(major) = 38.7 min.

Ethyl (2*S*,3*R*)-2-allyl-2-(benzo[d]oxazol-2-yl)-3-methylhex-4-ynoate (3z)

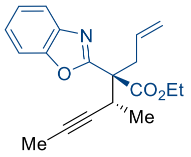

Colorless oil (38.6 mg, 83% yield, 15:1 dr). R_f = 0.40 (Hexane/EtOAc = 19/1). Prepared according to the above general procedure, but the reaction was performed at 20 °C. **^{1}H NMR (500 MHz, CDCl$_3$)** δ 7.81 – 7.67 (m, 1H), 7.57 – 7.47 (m, 1H), 7.37 – 7.28 (m, 2H), 5.84 – 5.64 (m, 1H), 5.12 – 4.96 (m, 2H), 4.33 – 4.18 (m, 2H), 3.54 – 3.42 (m, 1H), 3.14 (dd, J = 13.8, 6.7 Hz, 1H), 3.00 (dd, J = 13.9, 7.4 Hz, 1H), 1.80 (s, 3H), 1.32 – 1.22 (m, 6H). **^{13}C NMR (125 MHz, CDCl$_3$)** δ 169.97, 164.77, 150.86, 140.66, 132.77, 125.06, 124.30, 120.25, 119.17, 110.72, 79.17, 78.96, 61.65, 56.83, 39.87, 31.92, 17.58, 14.32, 3.76. **ESI-MS:** calculated [$C_{19}H_{21}NO_3$ + Na]$^+$: 334.1414, found: 334.1416. $[\alpha]^{20}_D$ = -43.0 (c = 0.93, CH_2Cl_2). The product was analyzed by HPLC to determine the enantiomeric excess: >99% ee (CHIRALPAK IC, hexane/*i*-PrOH = 99/1, detector: 254 nm, T = 25 °C, flow rate: 0.5 mL/min), t_1(minor) = 17.8 min, t_2(major) = 25.1 min.

Ethyl (2*S*,3*R*)-2-allyl-2-(benzo[d]oxazol-2-yl)-3-methylhept-4-ynoate (3aa)

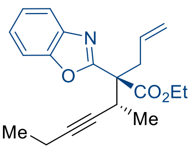

Colorless oil (45.0 mg, 92% yield, >20:1 dr). R_f = 0.40 (Hexane/EtOAc = 19/1). Prepared according to the above general procedure, but the reaction was performed at 20 °C. **^{1}H NMR (500 MHz, CDCl$_3$)** δ 7.78 – 7.71 (m, 1H), 7.54 – 7.49 (m, 1H), 7.36 – 7.30 (m, 2H), 5.83 – 5.72 (m, 1H), 5.12 – 4.98 (m, 2H), 4.27 (q, J = 7.1 Hz, 2H), 3.52 – 3.45 (m, 1H), 3.15 (dd, J = 14.1, 7.1 Hz, 1H), 3.00 (dd, J = 14.1, 7.5 Hz, 1H), 2.16 (qd, J = 7.5, 2.2 Hz, 2H), 1.30 – 1.24 (m, 6H), 1.09 (t, J = 7.5 Hz, 3H). **^{13}C NMR (125 MHz, CDCl$_3$)** δ 169.95, 164.81, 150.86, 140.66, 132.81, 125.03, 124.27, 120.24, 119.14, 110.69, 84.97, 79.55, 61.62, 56.91, 39.78, 31.88, 17.58, 14.34, 14.25, 12.59. **ESI-MS:** calculated [$C_{20}H_{23}NO_3$ + Na]$^+$: 348.1570, found: 348.1579. $[\alpha]^{20}_D$ = -44.6 (c = 1.03, CH_2Cl_2). The product was analyzed by HPLC to determine the enantiomeric excess: >99% ee (CHIRALPAK IC, hexane/*i*-PrOH = 99/1, detector: 254 nm, T = 25 °C, flow rate: 1.0 mL/min), t_1(minor) = 8.5 min, t_2(major) = 11.2 min.

Ethyl (2*S*,3*R*)-2-allyl-2-(benzo[d]oxazol-2-yl)-3-methylnon-4-ynoate (3ab)

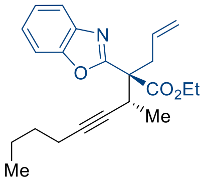

Colorless oil (48.0 mg, 91% yield, >20:1 dr). R_f = 0.40 (Hexane/EtOAc = 19/1). Prepared according to the above general procedure, but the reaction was performed at 20 °C. **^{1}H NMR (400 MHz, CDCl$_3$)** δ 7.77 – 7.69 (m, 1H), 7.54 – 7.48 (m, 1H), 7.36 – 7.29 (m, 2H), 5.87 – 5.72 (m, 1H), 5.12 – 4.95 (m, 2H), 4.26 (q, J = 7.1 Hz, 2H), 3.56 – 3.45 (m, 1H), 3.16 (dd, J = 14.1, 7.0 Hz, 1H), 3.00 (dd, J = 14.1, 7.5 Hz, 1H), 2.16 (td, J = 6.8, 2.2 Hz, 2H), 1.46 – 1.34 (m, 4H), 1.30 – 1.24 (m, 6H), 0.87 (t, J = 7.2 Hz, 3H). **^{13}C NMR (100 MHz, CDCl$_3$)** δ 169.97,

164.88, 150.91, 140.73, 132.93, 125.03, 124.27, 120.26, 119.05, 110.68, 83.59, 80.22, 61.61, 56.94, 39.71, 31.98, 31.09, 21.92, 18.55, 17.60, 14.31, 13.71. **ESI-MS:** calculated [$C_{22}H_{27}NO_3$ + Na]$^+$: 376.1883, found: 376.1898. $[\alpha]^{20}_D$ = -47.0 (c = 0.92, CH_2Cl_2). The product was analyzed by HPLC to determine the enantiomeric excess: >99% ee (CHIRALPAK IC, hexane/*i*-PrOH = 99/1, detector: 254 nm, T = 25 °C, flow rate: 0.5 mL/min), t_1(minor) = 16.7 min, t_2(major) = 22.4 min.

Ethyl (2*S*,3*R*)-2-allyl-2-(benzo[d]oxazol-2-yl)-3-methylundec-4-ynoate (3ac)

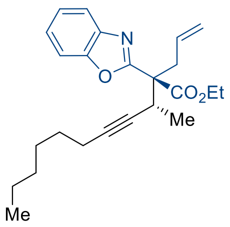

Colorless oil (53.0 mg, 93% yield, >20:1 dr). R_f = 0.45 (Hexane/EtOAc = 19/1). Prepared according to the above general procedure, but the reaction was performed at 20 °C. **^{1}H NMR (400 MHz, CDCl$_3$)** δ 7.77 – 7.69 (m, 1H), 7.54 – 7.46 (m, 1H), 7.35 – 7.29 (m, 2H), 5.86 – 5.70 (m, 1H), 5.11 – 4.95 (m, 2H), 4.26 (q, *J* = 7.1 Hz, 2H), 3.55 – 3.45 (m, 1H), 3.16 (dd, *J* = 14.1, 7.1 Hz, 1H), 3.00 (dd, *J* = 14.1, 7.5 Hz, 1H), 2.15 (td, *J* = 6.9, 2.2 Hz, 2H), 1.47 – 1.23 (m, 14H), 0.87 (t, *J* = 6.9 Hz, 3H). **^{13}C NMR (100 MHz, CDCl$_3$)** δ 169.95, 164.89, 150.91, 140.74, 132.92, 125.02, 124.27, 120.26, 119.05, 110.68, 83.70, 80.20, 61.60, 56.92, 39.73, 31.97, 31.49, 28.99, 28.56, 22.68, 18.89, 17.63, 14.32, 14.17. **ESI-MS:** calculated [$C_{24}H_{31}NO_3$ + Na]$^+$: 404.2196, found: 404.2207. $[\alpha]^{20}_D$ = -45.8 (c = 1.00, CH_2Cl_2). The product was analyzed by HPLC to determine the enantiomeric excess: >99% ee (CHIRALPAK IC, hexane/*i*-PrOH = 99/1, detector: 254 nm, T = 25 °C, flow rate: 0.5 mL/min), t_1(minor) = 15.2 min, t_2(major) = 21.3 min.

Ethyl (2*S*,3*R*)-2-allyl-2-(benzo[d]oxazol-2-yl)-8-chloro-3-methyloct-4-ynoate (3ad)

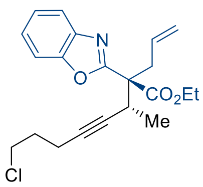

Colorless oil (51.6 mg, 92% yield, >20:1 dr). R_f = 0.30 (Hexane/EtOAc = 19/1). Prepared according to the above general procedure, but the reaction was performed at 20 °C. **^{1}H NMR (400 MHz, CDCl$_3$)** δ 7.77 – 7.71 (m, 1H), 7.56 – 7.50 (m, 1H), 7.37 – 7.30 (m, 2H), 5.88 – 5.71 (m, 1H), 5.15 – 4.99 (m, 2H), 4.26 (q, *J* = 7.1 Hz, 2H), 3.71 – 3.58 (m, 2H), 3.52 – 3.40 (m, 1H), 3.15 (dd, *J* = 14.2, 6.9 Hz, 1H), 2.98 (dd, *J* = 14.2, 7.7 Hz, 1H), 2.36 (td, *J* = 6.6, 2.3 Hz, 2H), 1.95 – 1.85 (m, 2H), 1.29 – 1.23 (m, 6H). **^{13}C NMR (100 MHz, CDCl$_3$)** δ 169.97, 164.64, 150.92, 140.66, 132.71, 125.15, 124.35, 120.27, 119.23, 110.72, 81.65, 81.43, 61.71, 56.87, 43.72, 39.58, 31.87, 31.71, 17.31, 16.33, 14.33. **ESI-MS:** calculated [$C_{21}H_{24}ClNO_3$ + Na]$^+$: 396.1337, found: 396.1346. $[\alpha]^{20}_D$ = -41.2 (c = 1.00, CH_2Cl_2). The product was analyzed by HPLC to determine the enantiomeric excess: >99% ee (CHIRALPAK IC, hexane/*i*-PrOH = 99/1, detector: 254 nm, T = 25 °C, flow rate: 0.5 mL/min), t_1(minor) = 16.9 min, t_2(major) = 24.2 min.

Ethyl (2*S*,3*R*)-2-allyl-2-(benzo[d]oxazol-2-yl)-9-((tert-butyldimethylsilyl)oxy)-3-methylnon-4-ynoate (3ae)

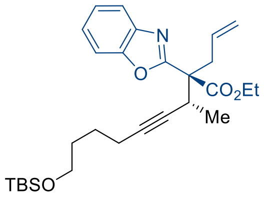

Colorless oil (63.8 mg, 88% yield, >20:1 dr). R_f = 0.45 (Hexane/EtOAc = 19/1). Prepared according to the above general procedure, but the reaction was performed at 20 °C. **^{1}H NMR (400 MHz, CDCl$_3$)** δ 7.76 – 7.70 (m, 1H), 7.54 – 7.49 (m, 1H), 7.35 – 7.29 (m, 2H), 5.86 – 5.72 (m, 1H), 5.12 – 4.98 (m, 2H), 4.26 (q, *J* = 7.1 Hz, 2H), 3.59 (t, *J* = 6.2

Hz, 2H), 3.53 – 3.46 (m, 1H), 3.15 (dd, J = 14.1, 7.0 Hz, 1H), 2.99 (dd, J = 14.1, 7.5 Hz, 1H), 2.18 (td, J = 6.8, 2.2 Hz, 2H), 1.63 – 1.48 (m, 4H), 1.31 – 1.23 (m, 6H), 0.88 (s, 9H), 0.04 (s, 6H). **^{13}C NMR (100 MHz, CDCl$_3$)** δ 169.95, 164.86, 150.92, 140.73, 132.88, 125.04, 124.28, 120.25, 119.10, 110.72, 83.43, 80.43, 62.82, 61.61, 56.92, 39.73, 32.01, 31.96, 26.10, 25.47, 18.70, 18.47, 17.60, 14.34, -5.16. **ESI-MS:** calculated [$C_{28}H_{41}NO_4Si$ + Na]$^+$: 506.2697, found: 506.2706. $[\alpha]^{20}_D$ = -36.7 (c = 1.03, CH_2Cl_2). The product was analyzed by HPLC to determine the enantiomeric excess: >99% ee (CHIRALPAK IG, hexane/*i*-PrOH = 99/1, detector: 254 nm, T = 25 °C, flow rate: 0.5 mL/min), t_1(minor) = 11.4 min, t_2(major) = 14.3 min.

Ethyl (2*S*,3*R*)-2-allyl-2-(benzo[d]oxazol-2-yl)-3-methyl-6-phenoxyhex-4-ynoate (3af)

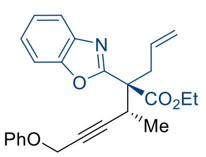

Colorless oil (57.9 mg, 96% yield, >20:1 dr). R_f = 0.30 (Hexane/EtOAc = 19/1). Prepared according to the above general procedure, but the reaction was performed at 20 °C. **^{1}H NMR (400 MHz, CDCl$_3$)** δ 7.76 – 7.70 (m, 1H), 7.46 – 7.40 (m, 1H), 7.36 – 7.29 (m, 2H), 7.28 – 7.23 (m, 2H), 7.01 – 6.89 (m, 3H), 5.79 – 5.64 (m, 1H), 5.07 – 4.96 (m, 2H), 4.70 (d, J = 1.9 Hz, 2H), 4.20 (q, J = 7.1 Hz, 2H), 3.61 – 3.51 (m, 1H), 3.11 (dd, J = 14.2, 7.0 Hz, 1H), 2.97 (dd, J = 14.2, 7.6 Hz, 1H), 1.29 (d, J = 7.1 Hz, 3H), 1.21 (t, J = 7.1 Hz, 3H). **^{13}C NMR (100 MHz, CDCl$_3$)** δ 169.72, 164.37, 157.94, 150.92, 140.62, 132.36, 129.46, 125.18, 124.38, 121.33, 120.28, 119.47, 115.15, 110.77, 88.08, 78.33, 61.81, 56.53, 56.36, 39.54, 31.71, 16.92, 14.25. **ESI-MS:** calculated [$C_{25}H_{25}NO_4$ + H]$^+$: 404.1856, found: 404.1866. $[\alpha]^{20}_D$ = -46.3 (c = 1.10, CH_2Cl_2). The product was analyzed by HPLC to determine the enantiomeric excess: >99% ee (CHIRALPAK IF, hexane/*i*-PrOH = 99/1, detector: 254 nm, T = 25 °C, flow rate: 0.5 mL/min), t_1(minor) = 19.0 min, t_2(major) = 21.8 min.

Ethyl (*S*)-2-(benzo[d]oxazol-2-yl)-2-((*R*)-4-cyclohexylbut-3-yn-2-yl)pent-4-enoate (3ag)

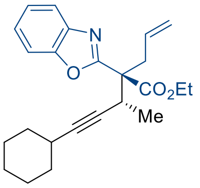

Colorless oil (48.9 mg, 86% yield, 18:1 dr). R_f = 0.45 (Hexane/EtOAc = 19/1). Prepared according to the above general procedure, but the reaction was performed at 20 °C. **^{1}H NMR (500 MHz, CDCl$_3$)** δ 7.79 – 7.70 (m, 1H), 7.56 – 7.47 (m, 1H), 7.38 – 7.28 (m, 2H), 5.88 – 5.70 (m, 1H), 5.14 – 4.93 (m, 2H), 4.35 – 4.15 (m, 2H), 3.60 – 3.42 (m, 1H), 3.16 (dd, J = 13.9, 6.9 Hz, 1H), 2.99 (dd, J = 13.8, 7.5 Hz, 1H), 2.48 – 2.27 (m, 1H), 1.76 – 1.63 (m, 4H), 1.48 – 1.24 (m, 12H). **^{13}C NMR (125 MHz, CDCl$_3$)** δ 169.98, 164.88, 150.90, 140.68, 132.91, 125.02, 124.26, 120.22, 119.11, 110.68, 87.68, 80.34, 61.62, 57.01, 39.71, 32.83, 31.87, 28.98, 26.12, 24.66, 17.58, 14.34. **ESI-MS:** calculated [$C_{24}H_{29}NO_3$ + Na]$^+$: 402.2040, found: 402.2048. $[\alpha]^{20}_D$ = -46.5 (c = 0.94, CH_2Cl_2). The product was analyzed by HPLC to determine the enantiomeric excess: >99% ee (CHIRALPAK IC, hexane/*i*-PrOH = 99/1, detector: 254 nm, T = 25 °C, flow rate: 0.5 mL/min), t_1(minor) = 16.0 min, t_2(major) = 21.6 min.

Ethyl (*S*)-2-(benzo[d]oxazol-2-yl)-2-((*R*)-4-(cyclohex-1-en-1-yl)but-3-yn-2-yl)pent-4-enoate (3ah)

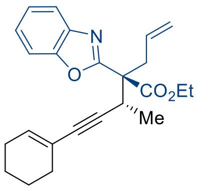

Colorless oil (51.3 mg, 91% yield, >20:1 dr). R_f = 0.40 (Hexane/EtOAc = 19/1). Prepared according to the above general procedure, but the reaction was performed at 20 °C. **^{1}H NMR (400 MHz, $CDCl_3$)** δ 7.76 – 7.70 (m, 1H), 7.54 – 7.48 (m, 1H), 7.36 – 7.29 (m, 2H), 6.05 – 5.94 (m, 1H), 5.84 – 5.70 (m, 1H), 5.14 – 4.97 (m, 2H), 4.27 (q, *J* = 7.1 Hz, 2H), 3.62 (q, *J* = 7.0 Hz, 1H), 3.16 (dd, *J* = 14.1, 7.2 Hz, 1H), 3.02 (dd, *J* = 14.1, 7.5 Hz, 1H), 2.12 – 2.00 (m, 4H), 1.64 – 1.52 (m, 4H), 1.33 (d, *J* = 7.0 Hz, 3H), 1.27 (t, *J* = 7.1 Hz, 3H). **^{13}C NMR (100 MHz, $CDCl_3$)** δ 169.85, 164.75, 150.92, 140.72, 133.91, 132.75, 125.07, 124.30, 120.85, 120.27, 119.23, 110.71, 87.06, 85.36, 61.68, 56.97, 39.84, 32.31, 29.46, 25.67, 22.46, 21.68, 17.43, 14.34. **ESI-MS:** calculated $[C_{24}H_{27}NO_3 + Na]^+$: 400.1883, found: 400.1882. $[\alpha]^{20}_D$ = -54.2 (c = 1.00, CH_2Cl_2). The product was analyzed by HPLC to determine the enantiomeric excess: >99% ee (CHIRALPAK IF, hexane/*i*-PrOH = 99/1, detector: 254 nm, T = 25 °C, flow rate: 0.5 mL/min), t_1(minor) = 13.4 min, t_2(major) = 15.5 min.

Ethyl (*S*)-2-(benzo[d]oxazol-2-yl)-2-((*R*)-1,4-diphenylbut-3-yn-2-yl)pent-4-enoate (3ai)

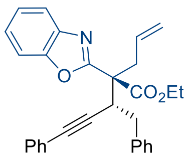

Colorless oil (60.0 mg, 89% yield, >20:1 dr). R_f = 0.35 (Hexane/EtOAc = 19/1). It was prepared according to the general procedure described above. **^{1}H NMR (400 MHz, $CDCl_3$)** δ 7.83 – 7.77 (m, 1H), 7.58 – 7.52 (m, 1H), 7.44 – 7.38 (m, 2H), 7.37 – 7.21 (m, 10H), 5.89 – 5.72 (m, 1H), 5.19 – 5.00 (m, 2H), 4.42 – 4.25 (m, 2H), 3.83 (dd, *J* = 11.4, 2.8 Hz, 1H), 3.41 – 3.28 (m, 2H), 3.20 (dd, *J* = 14.2, 7.3 Hz, 1H), 2.70 – 2.57 (m, 1H), 1.30 (t, *J* = 7.1 Hz, 3H). **^{13}C NMR (100 MHz, $CDCl_3$)** δ 169.76, 164.63, 150.99, 140.78, 139.89, 132.50, 131.59, 129.61, 128.29, 128.26, 127.97, 126.58, 125.24, 124.45, 123.63, 120.44, 119.65, 110.77, 88.31, 85.65, 61.96, 56.66, 40.90, 40.07, 37.75, 14.39. **ESI-MS:** calculated $[C_{30}H_{27}NO_3 + Na]^+$: 472.1883, found: 472.1886. $[\alpha]^{20}_D$ = -136.8 (c = 1.00, CH_2Cl_2). The product was analyzed by HPLC to determine the enantiomeric excess: >99% ee (CHIRALPAK IE, hexane/*i*-PrOH = 99/1, detector: 254 nm, T = 25 °C, flow rate: 0.5 mL/min), t_1(major) = 18.1 min, t_2(minor) = 20.5 min.

Ethyl (*S*)-2-(benzo[d]oxazol-2-yl)-2-((*R*)-1,5-diphenylpent-1-yn-3-yl)pent-4-enoate (3aj)

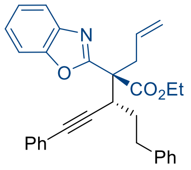

Colorless oil (53.0 mg, 76% yield, 7:1 dr). R_f = 0.35 (Hexane/EtOAc = 19/1). Prepared according to the above general procedure, but the reaction was performed at 20 °C. **^{1}H NMR (500 MHz, $CDCl_3$)** δ 7.81 – 7.75 (m, 1H), 7.58 – 7.53 (m, 1H), 7.50 – 7.44 (m, 2H), 7.38 – 7.23 (m, 10H), 5.89 – 5.79 (m, 1H), 5.17 – 5.03 (m, 2H), 4.37 – 4.26 (m, 2H), 3.64 (dd, *J* = 11.7, 2.8 Hz, 1H), 3.27 (dd, *J* = 14.2, 7.1 Hz, 1H), 3.15 – 3.03 (m, 2H), 2.88 – 2.81 (m, 1H), 2.25 – 2.16 (m, 1H), 1.84 – 1.73 (m, 1H), 1.28 (t, *J* = 7.1 Hz, 3H). **^{13}C NMR (125 MHz, $CDCl_3$)** δ 169.82, 164.59, 150.92, 141.55, 140.67, 132.57, 131.75, 128.70, 128.46, 128.35, 128.05, 126.06, 125.15, 124.36, 123.67, 120.34, 119.44, 110.76, 88.73, 84.99, 61.84, 56.77, 39.84, 37.97, 34.45, 32.53, 14.35. **ESI-MS:** calculated $[C_{31}H_{29}NO_3 + Na]^+$: 486.2040, found: 486.2053. $[\alpha]^{20}_D$ = -87.2 (c = 1.00, CH_2Cl_2). The product was analyzed by HPLC to determine the enantiomeric excess: >99% ee (CHIRALPAK IG, hexane/*i*-PrOH = 99/1, detector: 254 nm, T = 25 °C, flow rate: 0.5 mL/min), t_1(major) = 21.6 min, t_2(minor) = 25.4 min.

Ethyl (2*S*,3*R*)-2-allyl-2-(benzo[d]oxazol-2-yl)-3-benzylhex-4-ynoate (3ak)

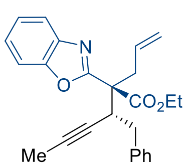

Colorless oil (45.9 mg, 79% yield, 17:1 dr). R_f = 0.35 (Hexane/EtOAc = 19/1). It was prepared according to the general procedure described above. **^{1}H NMR (500 MHz, $CDCl_3$)** δ 7.85 – 7.79 (m, 1H), 7.61 – 7.55 (m, 1H), 7.42 – 7.36 (m, 4H), 7.35 – 7.31 (m, 2H), 7.27 – 7.23 (m, 1H), 5.87 – 5.74 (m, 1H), 5.14 – 5.00 (m, 2H), 4.42 – 4.31 (m, 2H), 3.63 – 3.53 (m, 1H), 3.32 – 3.22 (m, 2H), 3.15 (dd, *J* = 14.1, 7.4 Hz, 1H), 2.61 – 2.52 (m, 1H), 1.79 (d, *J* = 2.4 Hz, 3H), 1.33 (t, *J* = 7.1 Hz, 3H). **^{13}C NMR (125 MHz, $CDCl_3$)** δ 169.87, 164.80, 150.87, 140.73, 140.27, 132.65, 129.46, 128.20, 126.39, 125.12, 124.37, 120.38, 119.37, 110.72, 81.06, 77.14, 61.79, 56.68, 40.46, 40.14, 38.05, 14.36, 3.74. **ESI-MS:** calculated $[C_{25}H_{25}NO_3 + Na]^+$: 410.1727, found: 410.1741. $[\alpha]^{20}_D$ = -40.6 (c = 0.90, CH_2Cl_2). The product was analyzed by HPLC to determine the enantiomeric excess: >99% ee (CHIRALPAK IE, hexane/*i*-PrOH = 99/1, detector: 254 nm, T = 25 °C, flow rate: 0.5 mL/min), t_1(major) = 17.0 min, t_2(minor) = 19.0 min.

Ethyl (2*S*,3*R*)-2-allyl-2-(benzo[d]oxazol-2-yl)-3-ethylnon-4-ynoate (3al)

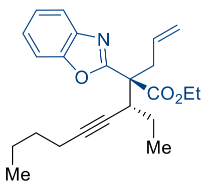

Colorless oil (48.4 mg, 88% yield, 12:1 dr). R_f = 0.50 (Hexane/EtOAc = 19/1). Prepared according to the above general procedure, but the reaction was performed at 20 °C. **^{1}H NMR (400 MHz, $CDCl_3$)** δ 7.78 – 7.70 (m, 1H), 7.53 – 7.48 (m, 1H), 7.34 – 7.29 (m, 2H), 5.88 – 5.75 (m, 1H), 5.11 – 4.96 (m, 2H), 4.25 (q, *J* = 7.1 Hz, 2H), 3.26 – 3.15 (m, 2H), 3.01 (dd, *J* = 14.1, 7.6 Hz, 1H), 2.18 (td, *J* = 6.8, 2.2 Hz, 2H), 1.84 – 1.74 (m, 1H), 1.48 – 1.35 (m, 4H), 1.29 – 1.23 (m, 4H), 1.06 (t, *J* = 7.3 Hz, 3H), 0.88 (t, *J* = 7.1 Hz, 3H). **^{13}C NMR (100 MHz, $CDCl_3$)** δ 170.07, 165.10, 150.92, 140.77, 133.13, 124.98, 124.24, 120.27, 118.95, 110.68, 84.61, 78.76, 61.59, 56.97, 40.02, 39.85, 31.15, 24.45, 21.94, 18.58, 14.30, 13.71, 12.98. **ESI-MS:** calculated $[C_{23}H_{29}NO_3 + Na]^+$: 390.2040, found: 390.2049. $[\alpha]^{20}_D$ = -47.9 (c = 0.92, CH_2Cl_2). The product was analyzed by HPLC to determine the enantiomeric excess: >99% ee (CHIRALPAK IC, hexane/*i*-PrOH = 99/1, detector: 254 nm, T = 25 °C, flow rate: 0.5 mL/min), t_1(minor) = 15.0 min, t_2(major) = 16.1 min.

2.4 Limitations of the Ni/Cu dual-catalyzed propargylation

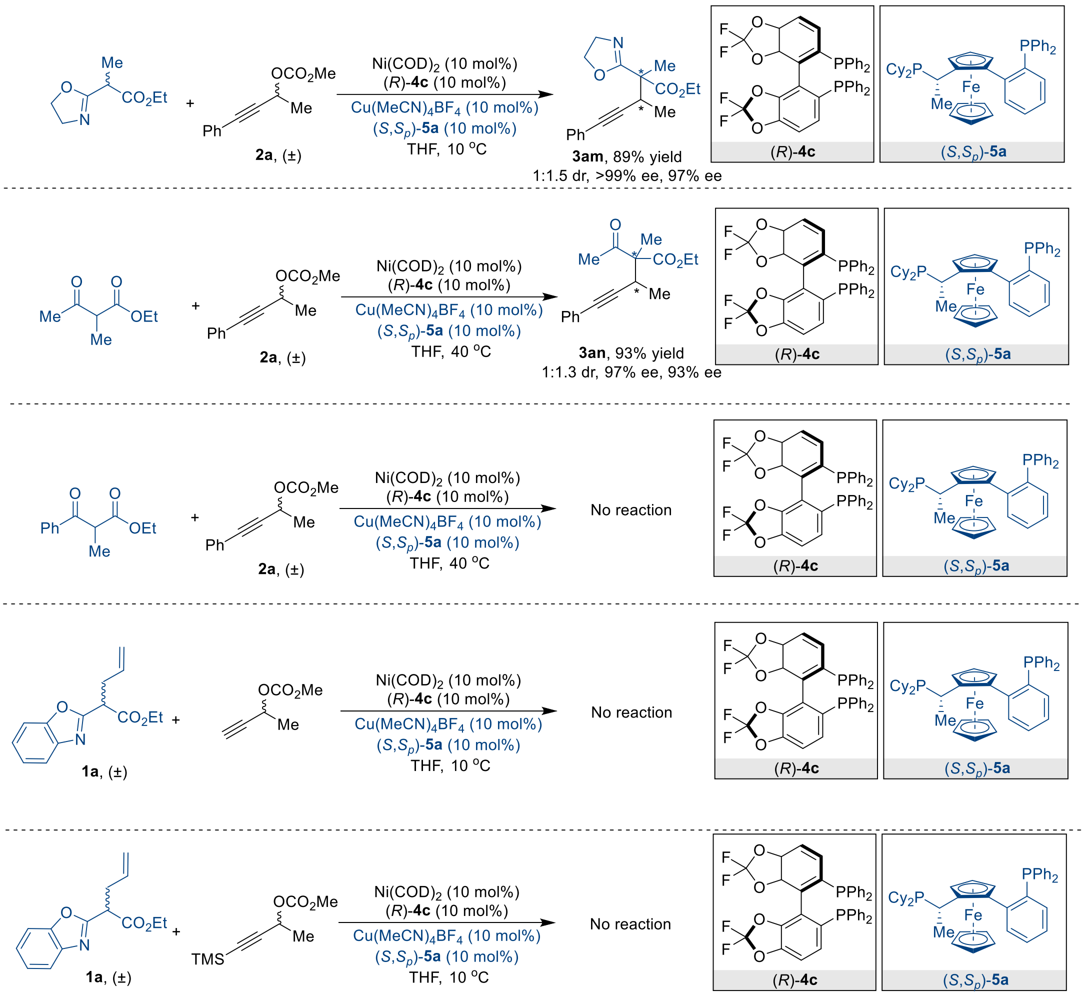

Ethyl 2-(4,5-dihydrooxazol-2-yl)-2,3-dimethyl-5-phenylpent-4-ynoate (3am)

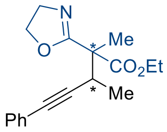

Colorless oil (40.0 mg, 89% yield, 1:1.5 dr). R_f = 0.35 (Hexane/EtOAc = 3/1). It was prepared according to the general procedure described above. **^{1}H NMR (400 MHz, $CDCl_3$)** δ 7.40 – 7.33 (m, 2H), 7.29 – 7.23 (m, 3H), 4.35 – 4.14 (m, 4H), 3.89 (t, J = 9.5 Hz, 2H), 3.70 – 3.58 (m, 1H), 1.62 (s, 3H), 1.34 – 1.24 (m, 6H). **^{13}C NMR (100 MHz, $CDCl_3$)** δ 171.06, 167.73, 131.67, 128.23, 127.83, 123.68, 90.47, 82.70, 68.10, 61.63, 54.46, 51.84, 32.49, 17.20, 16.79, 14.20. **ESI-MS:** calculated $[C_{18}H_{21}NO_3 + Na]^+$: 322.1414, found: 322.1419. $[\alpha]^{20}_{D}$ = 23.8 (c = 0.95, CH_2Cl_2). The product was analyzed by HPLC to determine the enantiomeric excess: >99% ee, 97% ee (CHIRALPAK IE, hexane/*i*-PrOH = 99/1, detector: 254 nm, T = 25 °C, flow rate: 1.0 mL/min), t_1(major) = 18.2 min, t_2(minor) = 19.8 min, t_3(minor) = 28.2 min, t_4(major) = 41.7 min.

Ethyl 2-acetyl-2,3-dimethyl-5-phenylpent-4-ynoate (3an)

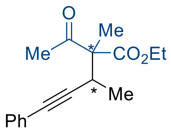

Colorless oil (38.0mg, 93% yield, 1:1.3 dr). R_f = 0.50 (Hexane/EtOAc = 19/1). Prepared according to the above general procedure, but the reaction was performed at 40 °C. **^{1}H NMR (400 MHz, $CDCl_3$)** δ 7.39 – 7.32 (m, 2H), 7.30 – 7.24 (m, 3H), 4.31 – 4.14 (m, 2H), 3.69 – 3.58 (m, 1H), 2.30 (s, 3H), 1.49 (s, 3H), 1.30 – 1.20 (m, 6H). **^{13}C NMR (100 MHz, $CDCl_3$)** δ 204.03, 171.09, 131.68, 128.31,

128.08, 123.28, 90.07, 83.37, 63.49, 61.74, 31.29, 26.70, 16.91, 15.41, 14.18. **ESI-MS:** calculated $[C_{17}H_{20}O_3 + Na]^+$: 295.1305, found: 295.1310. $[\alpha]^{20}_D$ = 16.4 (c = 0.96, CH_2Cl_2). The major conformational product was analyzed by HPLC to determine the enantiomeric excess: 97% ee (CHIRALPAK IG, hexane/*i*-PrOH = 98/2, detector: 254 nm, T = 25 °C, flow rate: 1.0 mL/min), t_1(minor) = 7.6 min, t_2(major) = 9.4 min. The minor conformational product was analyzed by HPLC to determine the enantiomeric excess: 93% ee (CHIRALPAK IG, hexane/*i*-PrOH = 99/1, detector: 254 nm, T = 25 °C, flow rate: 0.5 mL/min), t_1(major) = 18.9 min, t_2(minor) = 20.0 min.

2.5 Stereodivergent synthesis

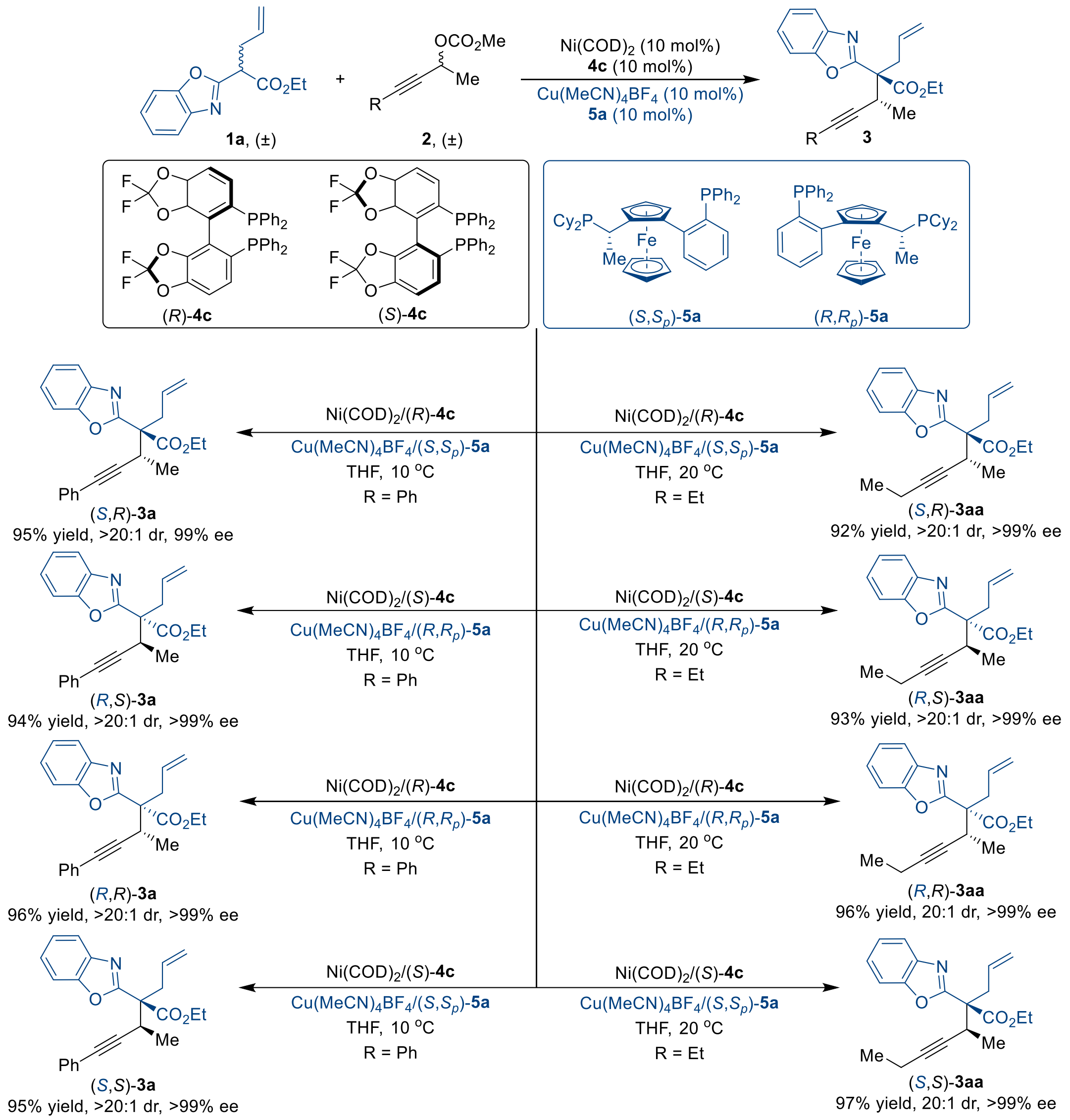

Ethyl (*S*)-2-(benzo[d]oxazol-2-yl)-2-((*R*)-4-phenylbut-3-yn-2-yl)pent-4-enoate ((*S,R*)-3a)

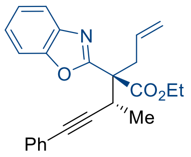

Colorless oil (53.2 mg, 95% yield, >20:1 dr). R_f = 0.40 (Hexane/EtOAc = 19/1). Benzoxazolyl acetate **1a** (0.15 mmol, 1.0 equiv.), propargylic carbonate **2** (0.3 mmol, 2.0 equiv.), Ni(COD)$_2$ (4.1 mg, 0.015 mmol, 10 mol%), (*R*)-**4c** (10.2 mg, 0.015 mmol, 10 mol%), Cu(MeCN)$_4$BF$_4$ (4.7 mg, 0.015 mmol, 10 mol%), (*S,S$_p$*)-**5a** (10.1 mg, 0.015 mmol, 10 mol%), and THF were used. The resulting solution was stirred for approximately 72 hours at 10 °C until substrate **1a** was completely consumed (monitored by TLC). The reaction mixture was subsequently concentrated under vacuum and purified by flash column chromatography on silica gel to afford the desired product (*S,R*)-**3a**. **^{1}H NMR (500 MHz, CDCl$_3$)** δ 7.78 – 7.74 (m, 1H), 7.56 – 7.51 (m, 1H), 7.41 – 7.37 (m, 2H), 7.36 – 7.32 (m, 2H), 7.31 – 7.27 (m, 3H), 5.86 – 5.76 (m, 1H), 5.17 – 5.03 (m, 2H), 4.34 – 4.26 (m, 2H), 3.75 (q, *J* = 7.0 Hz, 1H), 3.28 – 3.19 (m, 1H), 3.12 – 3.05 (m, 1H), 1.41 (d, *J* = 7.0 Hz, 3H), 1.27 (t, *J* = 7.1 Hz, 3H). **^{13}C NMR (125 MHz, CDCl$_3$)** δ 169.84, 164.58, 150.95, 140.65, 132.54, 131.69, 128.30, 127.96, 125.18, 124.38, 123.67, 120.30, 119.48, 110.76, 90.11, 83.55, 61.82, 56.90, 39.80, 32.26, 17.17, 14.37. **ESI-MS:**

calculated [$C_{24}H_{23}NO_3$ + Na]$^+$: 396.1570, found: 396.1578. $[\alpha]^{20}_D$ = -83.3 (c = 0.66, CH_2Cl_2). The product was analyzed by HPLC to determine the enantiomeric excess: >99% ee (CHIRALPAK IC, hexane/*i*-PrOH = 99/1, detector: 254 nm, T = 25 °C, flow rate: 0.5 mL/min), t_1(minor) = 15.8 min, t_2(major) = 26.6 min.

Ethyl (*R*)-2-(benzo[d]oxazol-2-yl)-2-((*S*)-4-phenylbut-3-yn-2-yl)pent-4-enoate ((*R*,*S*)-3a)

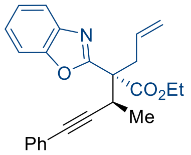

Colorless oil (52.7 mg, 94% yield, >20:1 dr). R_f = 0.40 (Hexane/EtOAc = 19/1). Benzoxazolyl acetate **1a** (0.15 mmol, 1.0 equiv.), propargylic carbonate **2** (0.3 mmol, 2.0 equiv.), Ni(COD)$_2$ (4.1 mg, 0.015 mmol, 10 mol%), (*S*)-**4c** (10.2 mg, 0.015 mmol, 10 mol%), Cu(MeCN)$_4$BF$_4$ (4.7 mg, 0.015 mmol, 10 mol%), (*R,R$_p$*)-**5a** (10.1 mg, 0.015 mmol, 10 mol%), and THF were used. The resulting solution was stirred for approximately 72 hours at 10 °C until substrate **1a** was completely consumed (monitored by TLC). The reaction mixture was subsequently concentrated under vacuum and purified by flash column chromatography on silica gel to afford the desired product (*R,S*)-**3a**. **^{1}H NMR (400 MHz, CDCl3)** δ 7.81 – 7.72 (m, 1H), 7.57 – 7.49 (m, 1H), 7.42 – 7.31 (m, 4H), 7.31 – 7.25 (m, 3H), 5.90 – 5.73 (m, 1H), 5.19 – 5.00 (m, 2H), 4.34 – 4.22 (m, 2H), 3.75 (q, *J* = 7.0 Hz, 1H), 3.29 – 3.20 (m, 1H), 3.15 – 3.03 (m, 1H), 1.41 (d, *J* = 7.0 Hz, 3H), 1.26 (t, *J* = 7.1 Hz, 3H). **^{13}C NMR (100 MHz, CDCl$_3$)** δ 169.84, 164.61, 150.97, 140.70, 132.59, 131.70, 128.30, 127.96, 125.17, 124.38, 123.71, 120.31, 119.44, 110.75, 90.15, 83.59, 61.81, 56.93, 39.80, 32.31, 17.19, 14.36. **ESI-MS:** calculated [$C_{24}H_{23}NO_3$ + Na]$^+$: 396.1570, found: 396.1575. $[\alpha]^{20}_D$ = 72.2 (c = 0.96, CH_2Cl_2). The product was analyzed by HPLC to determine the enantiomeric excess: >99% ee (CHIRALPAK IC, hexane/*i*-PrOH = 99/1, detector: 254 nm, T = 25 °C, flow rate: 0.5 mL/min), t_1(major) = 15.8 min, t_2(minor) = 26.6 min.

Ethyl (*R*)-2-(benzo[d]oxazol-2-yl)-2-((*R*)-4-phenylbut-3-yn-2-yl)pent-4-enoate ((*R*,*R*)-3a)

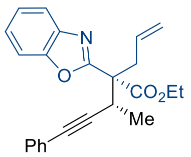

Colorless oil (53.9 mg, 96% yield, >20:1 dr). R_f = 0.40 (Hexane/EtOAc = 19/1). Benzoxazolyl acetate **1a** (0.15 mmol, 1.0 equiv.), propargylic carbonate **2** (0.3 mmol, 2.0 equiv.), Ni(COD)$_2$ (4.1 mg, 0.015 mmol, 10 mol%), (*R*)-**4c** (10.2 mg, 0.015 mmol, 10 mol%), Cu(MeCN)$_4$BF$_4$ (4.7 mg, 0.015 mmol, 10 mol%), (*R,R$_p$*)-**5a** (10.1 mg, 0.015 mmol, 10 mol%), and THF were used. The resulting solution was stirred for approximately 72 hours at 10 °C until substrate **1a** was completely consumed (monitored by TLC). The reaction mixture was subsequently concentrated under vacuum and purified by flash column chromatography on silica gel to afford the desired product (*R,R*)-**3a**. **^{1}H NMR (500 MHz, CDCl$_3$)** δ 7.81 – 7.76 (m, 1H), 7.57 – 7.52 (m, 1H), 7.37 – 7.31 (m, 2H), 7.29 – 7.25 (m, 2H), 7.25 – 7.19 (m, 3H), 6.07 – 5.89 (m, 1H), 5.28 – 5.21 (m, 1H), 5.17 – 5.12 (m, 1H), 4.27 – 4.19 (m, 2H), 3.68 (q, *J* = 7.0 Hz, 1H), 3.35 – 3.19 (m, 2H), 1.49 (d, *J* = 7.0 Hz, 3H), 1.23 (t, *J* = 7.1 Hz, 3H). **^{13}C NMR (125 MHz, CDCl$_3$)** δ 170.08, 164.18, 150.84, 140.83, 132.75, 131.64, 128.20, 127.93, 125.12, 124.36, 123.36, 120.36, 119.43, 110.69, 89.41, 84.02, 61.86, 56.89, 40.20, 33.06, 17.82, 14.28. **ESI-MS:** calculated [$C_{24}H_{23}NO_3$ + Na]$^+$: 396.1570, found: 396.1579. $[\alpha]^{20}_D$ = -151.0 (c = 0.95, CH_2Cl_2). The product was analyzed by HPLC to determine the enantiomeric excess: >99% ee (CHIRALPAK IC, hexane/*i*-PrOH = 99/1, detector: 254 nm, T = 25 °C, flow rate: 0.5 mL/min), t_1(minor) = 12.3 min, t_2(major) = 13.4 min.

Ethyl (*S*)-2-(benzo[d]oxazol-2-yl)-2-((*S*)-4-phenylbut-3-yn-2-yl)pent-4-enoate ((*S,S*)-3a)

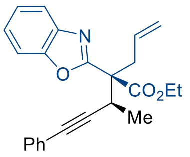

Colorless oil (53.2 mg, 95% yield, >20:1 dr). R_f = 0.40 (Hexane/EtOAc = 19/1). Benzoxazolyl acetate **1a** (0.15 mmol, 1.0 equiv.), propargylic carbonate **2** (0.3 mmol, 2.0 equiv.), $Ni(COD)_2$ (4.1 mg, 0.015 mmol, 10 mol%), (*S*)-**4c** (10.2 mg, 0.015 mmol, 10 mol%), $Cu(MeCN)_4BF_4$ (4.7 mg, 0.015 mmol, 10 mol%), (*S,S$_p$*)-**5a** (10.1 mg, 0.015 mmol, 10 mol%), and THF were used. The resulting solution was stirred for approximately 72 hours at 10 °C until substrate **1a** was completely consumed (monitored by TLC). The reaction mixture was subsequently concentrated under vacuum and purified by flash column chromatography on silica gel to afford the desired product (*S,S*)-**3a**. **^{1}H NMR (500 MHz, $CDCl_3$)** δ 7.82 – 7.76 (m, 1H), 7.57 – 7.51 (m, 1H), 7.37 – 7.32 (m, 2H), 7.29 – 7.25 (m, 2H), 7.25 – 7.19 (m, 3H), 6.04 – 5.91 (m, 1H), 5.29 – 5.20 (m, 1H), 5.17 – 5.11 (m, 1H), 4.24 (q, *J* = 7.1 Hz, 2H), 3.68 (q, *J* = 7.0 Hz, 1H), 3.35 – 3.20 (m, 2H), 1.49 (d, *J* = 7.0 Hz, 3H), 1.23 (t, *J* = 7.1 Hz, 3H). **^{13}C NMR (125 MHz, $CDCl_3$)** δ 170.09, 164.19, 150.85, 140.84, 132.76, 131.66, 128.21, 127.94, 125.13, 124.37, 123.37, 120.38, 119.43, 110.70, 89.42, 84.02, 61.87, 56.90, 40.21, 33.06, 17.83, 14.29. **ESI-MS:** calculated $[C_{24}H_{23}NO_3 + Na]^+$: 396.1570, found: 396.1565. $[\alpha]^{20}_D$ = 145.9 (c = 0.97, CH_2Cl_2). The product was analyzed by HPLC to determine the enantiomeric excess: >99% ee (CHIRALPAK IC, hexane/*i*-PrOH = 99/1, detector: 254 nm, T = 25 °C, flow rate: 0.5 mL/min), t_1(major) = 12.4 min, t_2(minor) = 13.7 min.

Ethyl (2*S*,3*R*)-2-allyl-2-(benzo[d]oxazol-2-yl)-3-methylhept-4-ynoate ((*S,R*)-3aa)

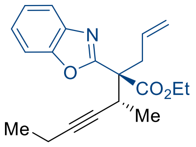

Colorless oil (45.0 mg, 92% yield, >20:1 dr). R_f = 0.40 (Hexane/EtOAc = 19/1). Benzoxazolyl acetate **1a** (0.15 mmol, 1.0 equiv.), propargylic carbonate **2** (0.3 mmol, 2.0 equiv.), $Ni(COD)_2$ (4.1 mg, 0.015 mmol, 10 mol%), (*R*)-**4c** (10.2 mg, 0.015 mmol, 10 mol%), $Cu(MeCN)_4BF_4$ (4.7 mg, 0.015 mmol, 10 mol%), (*S,S$_p$*)-**5a** (10.1 mg, 0.015 mmol, 10 mol%), and THF were used. The resulting solution was stirred for approximately 72 hours at 20 °C until substrate **1a** was completely consumed (monitored by TLC). The reaction mixture was subsequently concentrated under vacuum and purified by flash column chromatography on silica gel to afford the desired product (*S,R*)-**3aa**. **^{1}H NMR (500 MHz, $CDCl_3$)** δ 7.78 – 7.71 (m, 1H), 7.54 – 7.49 (m, 1H), 7.36 – 7.30 (m, 2H), 5.83 – 5.72 (m, 1H), 5.12 – 4.98 (m, 2H), 4.27 (q, *J* = 7.1 Hz, 2H), 3.52 – 3.45 (m, 1H), 3.15 (dd, *J* = 14.1, 7.1 Hz, 1H), 3.00 (dd, *J* = 14.1, 7.5 Hz, 1H), 2.16 (qd, *J* = 7.5, 2.2 Hz, 2H), 1.30 – 1.24 (m, 6H), 1.09 (t, *J* = 7.5 Hz, 3H). **^{13}C NMR (125 MHz, $CDCl_3$)** δ 169.95, 164.81, 150.86, 140.66, 132.81, 125.03, 124.27, 120.24, 119.14, 110.69, 84.97, 79.55, 61.62, 56.91, 39.78, 31.88, 17.58, 14.34, 14.25, 12.59. **ESI-MS:** calculated $[C_{20}H_{23}NO_3 + Na]^+$: 348.1570, found: 348.1579. $[\alpha]^{20}_D$ = -44.6 (c = 1.03, CH_2Cl_2). The product was analyzed by HPLC to determine the enantiomeric excess: >99% ee (CHIRALPAK IC, hexane/*i*-PrOH = 99/1, detector: 254 nm, T = 25 °C, flow rate: 1.0 mL/min), t_1(minor) = 8.5 min, t_2(major) = 11.2 min.

Ethyl (2*R*,3*S*)-2-allyl-2-(benzo[d]oxazol-2-yl)-3-methylhept-4-ynoate ((*R*,*S*)-3aa)

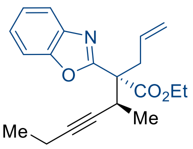

Colorless oil (45.5 mg, 93% yield, >20:1 dr). R_f = 0.40 (Hexane/EtOAc = 19/1). Benzoxazolyl acetate **1a** (0.15 mmol, 1.0 equiv.), propargylic carbonate **2** (0.3 mmol, 2.0 equiv.), $Ni(COD)_2$ (4.1 mg, 0.015 mmol, 10 mol%), (*S*)-**4c** (10.2 mg, 0.015 mmol, 10 mol%), $Cu(MeCN)_4BF_4$ (4.7 mg, 0.015 mmol, 10 mol%), (*R,R$_p$*)-**5a** (10.1 mg, 0.015 mmol, 10 mol%), and THF were used. The resulting solution was stirred for approximately 72 hours at 20 °C until substrate **1a** was completely consumed (monitored by TLC). The reaction mixture was subsequently concentrated under vacuum and purified by flash column chromatography on silica gel to afford the desired product (*R,S*)-**3aa**. **^{1}H NMR (400 MHz, $CDCl_3$)** δ 7.77 – 7.69 (m, 1H), 7.55 – 7.48 (m, 1H), 7.35 – 7.28 (m, 2H), 5.86 – 5.70 (m, 1H), 5.11 – 4.94 (m, 2H), 4.27 (q, *J* = 7.1 Hz, 2H), 3.54 – 3.43 (m, 1H), 3.15 (dd, *J* = 14.1, 7.1 Hz, 1H), 3.00 (dd, *J* = 14.1, 7.5 Hz, 1H), 2.16 (qd, *J* = 7.5, 2.2 Hz, 2H), 1.31 – 1.24 (m, 6H), 1.09 (t, *J* = 7.5 Hz, 3H). **^{13}C NMR (100 MHz, $CDCl_3$)** δ 169.96, 164.86, 150.90, 140.72, 132.88, 125.03, 124.28, 120.26, 119.10, 110.69, 84.99, 79.60, 61.61, 56.94, 39.78, 31.92, 17.59, 14.33, 14.24, 12.58. **ESI-MS:** calculated $[C_{20}H_{23}NO_3 + Na]^+$: 348.1570, found: 348.1571. $[\alpha]^{20}_D$ = 45.0 (c = 1.00, CH_2Cl_2). The product was analyzed by HPLC to determine the enantiomeric excess: >99% ee (CHIRALPAK IC, hexane/*i*-PrOH = 99/1, detector: 254 nm, T = 25 °C, flow rate: 1.0 mL/min), t_1(major) = 8.5 min, t_2(minor) = 11.1 min.

Ethyl (2*R*,3*R*)-2-allyl-2-(benzo[d]oxazol-2-yl)-3-methylhept-4-ynoate ((*R*,*R*)-3aa)

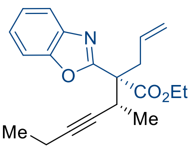

Colorless oil (47.0 mg, 96% yield, 20:1 dr). R_f = 0.40 (Hexane/EtOAc = 19/1). Benzoxazolyl acetate **1a** (0.15 mmol, 1.0 equiv.), propargylic carbonate **2** (0.3 mmol, 2.0 equiv.), $Ni(COD)_2$ (4.1 mg, 0.015 mmol, 10 mol%), (*R*)-**4c** (10.2 mg, 0.015 mmol, 10 mol%), $Cu(MeCN)_4BF_4$ (4.7 mg, 0.015 mmol, 10 mol%), (*R,R$_p$*)-**5a** (10.1 mg, 0.015 mmol, 10 mol%), and THF were used. The resulting solution was stirred for approximately 72 hours at 20 °C until substrate **1a** was completely consumed (monitored by TLC). The reaction mixture was subsequently concentrated under vacuum and purified by flash column chromatography on silica gel to afford the desired product (*R,R*)-**3aa**. **^{1}H NMR (500 MHz, $CDCl_3$)** δ 7.80 – 7.74 (m, 1H), 7.55 – 7.48 (m, 1H), 7.36 – 7.31 (m, 2H), 6.00 – 5.89 (m, 1H), 5.23 – 5.15 (m, 1H), 5.13 – 5.08 (m, 1H), 4.20 (q, *J* = 7.0 Hz, 2H), 3.44 – 3.37 (m, 1H), 3.24 (dd, *J* = 14.0, 6.3 Hz, 1H), 3.13 (dd, *J* = 14.0, 8.3 Hz, 1H), 2.05 (qd, *J* = 7.4, 1.8 Hz, 2H), 1.37 (d, *J* = 7.0 Hz, 3H), 1.21 (t, *J* = 7.1 Hz, 3H), 0.98 (t, *J* = 7.5 Hz, 3H). **^{13}C NMR (125 MHz, $CDCl_3$)** δ 170.25, 164.40, 150.79, 140.81, 132.98, 124.97, 124.24, 120.29, 119.11, 110.64, 85.48, 78.88, 61.67, 56.97, 40.23, 32.65, 18.20, 14.25, 14.13, 12.43. **ESI-MS:** calculated $[C_{20}H_{23}NO_3 + Na]^+$: 348.1570, found: 348.1583. $[\alpha]^{20}_D$ = -99.6 (c = 0.98, CH_2Cl_2). The product was analyzed by HPLC to determine the enantiomeric excess: >99% ee (CHIRALPAK IC, hexane/*i*-PrOH = 99/1, detector: 254 nm, T = 25 °C, flow rate: 1.0 mL/min), t_1(minor) = 6.1 min, t_2(major) = 7.4 min.

Ethyl (2*S*,3*S*)-2-allyl-2-(benzo[d]oxazol-2-yl)-3-methylhept-4-ynoate ((*S*,*S*)-3aa)

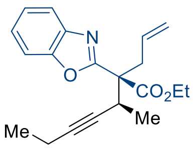

Colorless oil (47.2 mg, 97% yield, 20:1 dr). R_f = 0.40 (Hexane/EtOAc = 19/1). Benzoxazolyl acetate **1a** (0.15 mmol, 1.0 equiv.), propargylic carbonate **2** (0.3 mmol, 2.0 equiv.), $Ni(COD)_2$ (4.1 mg, 0.015 mmol, 10 mol%), (*S*)-**4c** (10.2 mg, 0.015 mmol, 10 mol%), $Cu(MeCN)_4BF_4$ (4.7 mg, 0.015 mmol, 10 mol%), (*S,S$_p$*)-**5a** (10.1 mg, 0.015 mmol, 10 mol%), and THF were used. The resulting solution was stirred for approximately 72 hours at 20 °C until substrate **1a** was completely consumed (monitored by TLC). The reaction mixture was subsequently concentrated under vacuum and purified by flash column chromatography on silica gel to afford the desired product (*S,S*)-**3aa**. **^{1}H NMR (500 MHz, $CDCl_3$)** δ 7.80 – 7.74 (m, 1H), 7.56 – 7.49 (m, 1H), 7.36 – 7.31 (m, 2H), 6.01 – 5.86 (m, 1H), 5.24 – 5.15 (m, 1H), 5.13 – 5.07 (m, 1H), 4.24 – 4.16 (m, 2H), 3.43 – 3.36 (m, 1H), 3.24 (dd, *J* = 14.0, 6.3 Hz, 1H), 3.13 (dd, *J* = 14.0, 8.3 Hz, 1H), 2.05 (qd, *J* = 7.4, 2.0 Hz, 2H), 1.37 (d, *J* = 7.0 Hz, 3H), 1.21 (t, *J* = 7.1 Hz, 3H), 0.98 (t, *J* = 7.5 Hz, 3H). **^{13}C NMR (125 MHz, $CDCl_3$)** δ 170.28, 164.45, 150.84, 140.87, 133.04, 124.99, 124.26, 120.33, 119.12, 110.67, 85.51, 78.93, 61.70, 57.01, 40.25, 32.68, 18.23, 14.29, 14.16, 12.47. **ESI-MS:** calculated $[C_{20}H_{23}NO_3 + Na]^+$: 348.1570, found: 348.1568. $[\alpha]^{20}_D$ = 100.7 (c = 0.96, CH_2Cl_2). The product was analyzed by HPLC to determine the enantiomeric excess: >99% ee (CHIRALPAK IC, hexane/*i*-PrOH = 99/1, detector: 254 nm, T = 25 °C, flow rate: 1.0 mL/min), t_1(major) = 6.1 min, t_2(minor) = 7.4 min.

2.6 Optimization of enantioselective propargylation of primary propargylic carbonate

Table S3. Optimization of enantioselective propargylation of primary propargylic carbonate

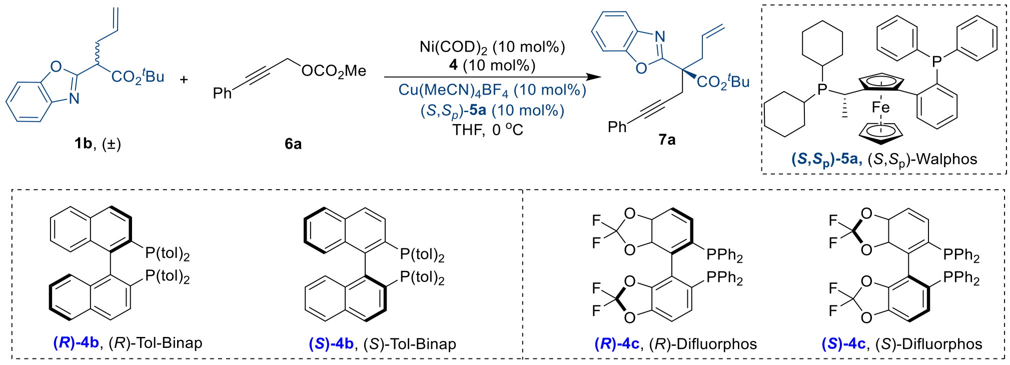

Entry	[Ni]	[Cu]	4	5	Yield (%)[b]	ee (%)[c]
1	Ni(COD)$_2$	Cu(MeCN)$_4$BF$_4$	(*R*)-**4c**	(*S*,*S$_p$*)-**5a**	91	68
2	Ni(COD)$_2$	Cu(MeCN)$_4$BF$_4$	(*S*)-**4c**	(*S*,*S$_p$*)-**5a**	93	92
3	Ni(COD)$_2$	Cu(MeCN)$_4$BF$_4$	(*S*)-**4b**	(*S*,*S$_p$*)-**5a**	96	97
4	Ni(COD)$_2$	Cu(MeCN)$_4$BF$_4$	(*R*)-**4b**	(*S*,*S$_p$*)-**5a**	90	21
5		Cu(MeCN)$_4$BF$_4$	(*S*)-**4b**	(*S*,*S$_p$*)-**5a**	nr	—
6	Ni(COD)$_2$		(*S*)-**4b**	(*S*,*S$_p$*)-**5a**	93	85
7	Ni(COD)$_2$	Cu(MeCN)$_4$BF$_4$		(*S*,*S$_p$*)-**5a**	nr	—
8	Ni(COD)$_2$	Cu(MeCN)$_4$BF$_4$	(*S*)-**4b**		41	55
9	-	Cu(MeCN)$_4$BF$_4$		(*S*,*S$_p$*)-**5a**	nr	—
10	Ni(COD)$_2$	-	(*S*)-**4b**		93	86

[a]Reactions in this table were conducted with **1b** (0.15 mmol), **6a** (0.3 mmol), Ni(COD)$_2$ (10 mol%), **4** (10 mol%), Cu(MeCN)$_4$BF$_4$ (10 mol%), and (*S*,*S$_p$*)-**5a** (10 mol%) in tetrahydrofuran (THF) at 0 °C for 72 h. [b]Isolated yields after chromatography. [c]ee values were determined by high-performance liquid chromatography analysis. nr = no reaction.

2.7 General procedure for the synthesis of product 7

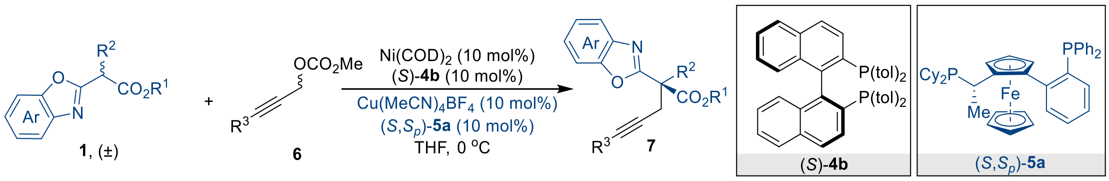

In a nitrogen-filled glove box, an oven-dried 10 mL screw-cap reaction tube equipped with a stir bar was changed with Ni(COD)$_2$ (4.1 mg, 0.015 mmol, 10 mol%) and (*S*)-**4b** (10.2 mg, 0.015 mmol, 10 mol%) in THF (1 mL) at rt for approximately 20 min; Meanwhile, Cu(MeCN)$_4$BF$_4$ (4.7 mg, 0.015 mmol, 10 mol%) and (*S,S$_p$*)-**5a** (10.1 mg, 0.015 mmol, 10 mol%) were stirred in THF (1 mL) in a Schlenk flask under a nitrogen atmosphere for 30 min. Benzoxazole ester **1** (0.15 mmol, 1.0 equiv.) was added to the Schlenk flask containing copper complex and stirred for approximately 5 min. Propargylic carbonate **6** (0.3 mmol, 2.0 equiv.) was then transformed into the Screw-cap reaction tube containing the nickel complex and stirred for an additional 5 min. The nickel complex solution was then combined with the copper complex solution and the resulting solution was stirred for approximately 72 hours at 0 °C until substrate **1** was completely consumed (monitored by TLC). The reaction mixture was

subsequently concentrated under vacuum and purified by flash column chromatography on silica gel to afford the desired product **7**.

Tert-butyl (*S*)-2-(benzo[d]oxazol-2-yl)-2-(3-phenylprop-2-yn-1-yl)pent-4-enoate (7a)

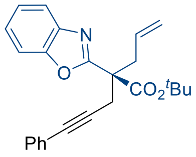

Colorless oil (56.0 mg, 96% yield). R_f = 0.45 (Hexane/EtOAc = 19/1). It was prepared according to the general procedure described above. **^{1}H NMR (500 MHz, CDCl$_3$)** δ 7.80 – 7.71 (m, 1H), 7.56 – 7.49 (m, 1H), 7.37 – 7.27 (m, 4H), 7.27 – 7.21 (m, 3H), 5.75 – 5.65 (m, 1H), 5.28 – 5.20 (m, 1H), 5.17 – 5.10 (m, 1H), 3.37 – 3.27 (m, 2H), 3.21 – 3.12 (m, 2H), 1.42 (s, 9H). **^{13}C NMR (125 MHz, CDCl$_3$)** δ 169.03, 165.26, 151.02, 140.94, 131.92, 131.72, 128.27, 128.02, 125.18, 124.45, 123.37, 120.32, 120.14, 110.73, 84.59, 83.79, 82.82, 52.87, 37.84, 27.96, 24.87. **ESI-MS:** calculated [$C_{25}H_{25}NO_3$ + Na]$^+$: 410.1727, found: 410.1742. $[\alpha]^{20}_D$ = 38.1 (c = 1.10, CH_2Cl_2). The product was analyzed by HPLC to determine the enantiomeric excess: 97% ee (CHIRALPAK AD-H, hexane/*i*-PrOH = 99/1, detector: 254 nm, T = 25 °C, flow rate: 1.0 mL/min), t_1(major) = 7.7 min, t_2(minor) = 10.0 min.

Tert-butyl (*S*)-2-(benzo[d]oxazol-2-yl)-2-methyl-5-phenylpent-4-ynoate (7b)

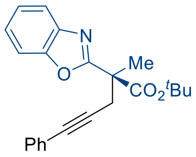

White solid (51.1 mg, 94% yield). R_f = 0.40 (Hexane/EtOAc = 19/1). It was prepared according to the general procedure described above. **^{1}H NMR (500 MHz, CDCl$_3$)** δ 7.83 – 7.68 (m, 1H), 7.58 – 7.48 (m, 1H), 7.37 – 7.21 (m, 7H), 3.39 – 3.23 (m, 2H), 1.89 (s, 3H), 1.42 (s, 9H). **^{13}C NMR (125 MHz, CDCl$_3$)** δ 170.17, 166.34, 151.08, 141.06, 131.72, 128.27, 128.02, 125.14, 124.43, 123.35, 120.28, 110.73, 84.89, 83.64, 82.65, 49.54, 28.19, 27.92, 21.38. **ESI-MS:** calculated [$C_{23}H_{23}NO_3$ + Na]$^+$: 384.1570, found: 384.1578. $[\alpha]^{20}_D$ = 59.4 (c = 0.92, CH_2Cl_2). The product was analyzed by HPLC to determine the enantiomeric excess: 94% ee (CHIRALPAK AD-H, hexane/*i*-PrOH = 99/1, detector: 254 nm, T = 25 °C, flow rate: 1.0 mL/min), t_1(major) = 7.6 min, t_2(minor) = 9.3 min.

Tert-butyl (*S*)-2-(benzo[d]oxazol-2-yl)-2-ethyl-5-phenylpent-4-ynoate (7c)

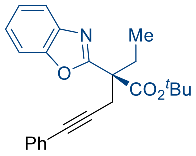

White solid (53.7 mg, 95% yield). R_f = 0.40 (Hexane/EtOAc = 19/1). It was prepared according to the general procedure described above. **^{1}H NMR (500 MHz, CDCl$_3$)** δ 7.79 – 7.72 (m, 1H), 7.56 – 7.50 (m, 1H), 7.36 – 7.31 (m, 2H), 7.32 – 7.27 (m, 2H), 7.26 – 7.20 (m, 3H), 3.39 – 3.29 (m, 2H), 2.46 (q, *J* = 7.5 Hz, 2H), 1.42 (s, 9H), 0.95 (t, *J* = 7.5 Hz, 3H). **^{13}C NMR (125 MHz, CDCl$_3$)** δ 169.63, 165.81, 151.00, 140.99, 131.73, 128.25, 127.97, 125.09, 124.38, 123.41, 120.27, 110.71, 84.70, 83.45, 82.55, 53.60, 27.96, 26.57, 24.52, 8.62. **ESI-MS:** calculated [$C_{24}H_{25}NO_3$ + Na]$^+$: 398.1721, found: 398.1730. $[\alpha]^{20}_D$ = 69.1 (c = 1.04, CH_2Cl_2). The product was analyzed by HPLC to determine the enantiomeric excess: 97% ee (CHIRALPAK AD-H, hexane/*i*-PrOH = 99/1, detector: 254 nm, T = 25 °C, flow rate: 1.0 mL/min), t_1(major) = 7.0 min, t_2(minor) = 10.3 min.

Tert-butyl (*S*)-2-(benzo[d]oxazol-2-yl)-5-phenyl-2-propylpent-4-ynoate (7d)

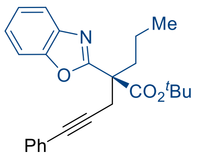

Colorless oil (46.0 mg, 79% yield). R_f = 0.45 (Hexane/EtOAc = 19/1). It was prepared according to the general procedure described above. **^{1}H NMR (500 MHz, $CDCl_3$)** δ 7.80 – 7.69 (m, 1H), 7.56 – 7.48 (m, 1H), 7.36 – 7.20 (m, 7H), 3.41 – 3.29 (m, 2H), 2.46 – 2.32 (m, 2H), 1.44 – 1.24 (m, 11H), 0.99 (t, *J* = 7.0 Hz, 3H). **^{13}C NMR (125 MHz, $CDCl_3$)** δ 169.68, 165.96, 150.98, 140.99, 131.73, 128.26, 127.97, 125.09, 124.39, 123.44, 120.27, 110.72, 84.76, 83.49, 82.55, 53.19, 35.60, 27.95, 25.04, 17.55, 14.54. **ESI-MS:** calculated $[C_{25}H_{27}NO_3 + Na]^+$: 412.1883, found: 412.1888. $[\alpha]^{20}_{D}$ = 59.4 (c = 1.00, CH_2Cl_2). The product was analyzed by HPLC to determine the enantiomeric excess: 95% ee (CHIRALPAK AD-H, hexane/*i*-PrOH = 99/1, detector: 254 nm, T = 25 °C, flow rate: 1.0 mL/min), t_1(major) = 6.1 min, t_2(minor) = 18.1 min.

Tert-butyl (*S*)-2-(benzo[d]oxazol-2-yl)-2-(3-phenylprop-2-yn-1-yl)hexanoate (7e)

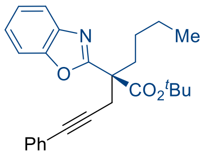

Colorless oil (54.5 mg, 90% yield). R_f = 0.45 (Hexane/EtOAc = 19/1). It was prepared according to the general procedure described above. **^{1}H NMR (400 MHz, $CDCl_3$)** δ 7.79 – 7.72 (m, 1H), 7.56 – 7.49 (m, 1H), 7.35 – 7.21 (m, 7H), 3.39 – 3.28 (m, 2H), 2.47 – 2.35 (m, 2H), 1.41 (s, 9H), 1.38 – 1.19 (m, 4H), 0.92 (t, *J* = 7.2 Hz, 3H). **^{13}C NMR (100 MHz, $CDCl_3$)** δ 169.70, 166.00, 151.01, 141.05, 131.75, 128.26, 127.96, 125.08, 124.38, 123.48, 120.30, 110.72, 84.79, 83.51, 82.53, 53.16, 33.05, 27.97, 26.24, 24.98, 23.03, 14.05. **ESI-MS:** calculated $[C_{26}H_{29}NO_3 + Na]^+$: 426.2040, found: 426.2039. $[\alpha]^{20}_{D}$ = 49.0 (c = 1.04, CH_2Cl_2). The product was analyzed by HPLC to determine the enantiomeric excess: 96% ee (CHIRALPAK AD-H, hexane/*i*-PrOH = 99/1, detector: 254 nm, T = 25 °C, flow rate: 1.0 mL/min), t_1(major) = 6.4 min, t_2(minor) = 11.5 min.

Tert-butyl (*S*)-2-(benzo[d]oxazol-2-yl)-2-(3-((tert-butyldimethylsilyl)oxy)propyl)-5-phenylpent-4-ynoate (7f)

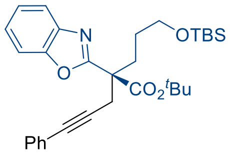

Colorless oil (72.6 mg, 93% yield). R_f = 0.40 (Hexane/EtOAc = 19/1). It was prepared according to the general procedure described above. **^{1}H NMR (400 MHz, $CDCl_3$)** δ 7.77 – 7.72 (m, 1H), 7.55 – 7.49 (m, 1H), 7.35 – 7.22 (m, 7H), 3.66 (t, *J* = 6.4 Hz, 2H), 3.41 – 3.29 (m, 2H), 2.48 – 2.39 (m, 2H), 1.64 – 1.56 (m, 1H), 1.50 – 1.44 (m, 1H), 1.42 (s, 9H), 0.86 (s, 9H), 0.04 – 0.00 (m, 6H). **^{13}C NMR (100 MHz, $CDCl_3$)** δ 169.58, 165.79, 151.03, 141.04, 131.77, 128.23, 127.96, 125.09, 124.39, 123.46, 120.30, 110.73, 84.66, 83.60, 82.60, 63.16, 52.99, 30.04, 27.97, 27.70, 26.06, 25.19, 18.43, -5.19. **ESI-MS:** calculated $[C_{31}H_{41}NO_4Si + Na]^+$: 542.2697, found: 542.2703. $[\alpha]^{20}_{D}$ = 41.2 (c = 1.03, CH_2Cl_2). The product was analyzed by HPLC to determine the enantiomeric excess: 97% ee (CHIRALPAK IA, hexane/*i*-PrOH = 99/1, detector: 254 nm, T = 25 °C, flow rate: 1.0 mL/min), t_1(major) = 4.4 min, t_2(minor) = 5.8 min.

Tert-butyl (*S*)-2-(benzo[d]oxazol-2-yl)-2-(3-methoxypropyl)-5-phenylpent-4-ynoate (7g)

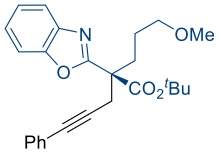

Yellow oil (53.7 mg, 85% yield). R_f = 0.40 (Hexane/EtOAc = 9/1). It was prepared according to the general procedure described above. **^{1}H NMR (400 MHz, $CDCl_3$)** δ 7.78 – 7.70 (m, 1H), 7.55 – 7.49 (m, 1H), 7.35 – 7.22 (m, 7H), 3.45 – 3.39 (m, 2H), 3.38 – 3.31 (m, 2H), 3.30 (s, 3H), 2.51 –

2.39 (m, 2H), 1.68 – 1.61 (m, 1H), 1.59 – 1.50 (m, 1H), 1.42 (s, 9H). **^{13}C NMR (100 MHz, CDCl$_3$)** δ 169.50, 165.69, 151.02, 141.01, 131.76, 128.26, 128.00, 125.13, 124.42, 123.43, 120.31, 110.74, 84.59, 83.66, 82.72, 72.62, 58.59, 52.95, 30.26, 27.97, 25.22, 24.46. **ESI-MS:** calculated [$C_{26}H_{29}NO_4$ + Na]$^+$: 442.1989, found: 442.1989. $[\alpha]^{20}_D$ = 58.2 (c = 0.98, CH_2Cl_2). The product was analyzed by HPLC to determine the enantiomeric excess: 95% ee (CHIRALPAK AD-H, hexane/*i*-PrOH = 95/5, detector: 260 nm, T = 25 °C, flow rate: 1.0 mL/min), t_1(major) = 5.1 min, t_2(minor) = 11.5 min.

Tert-butyl (*S*)-2-(benzo[d]oxazol-2-yl)-2-phenethyl-5-phenylpent-4-ynoate (7h)

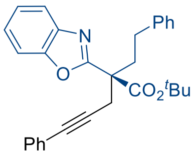

Colorless oil (65.7 mg, 97% yield). R_f = 0.40 (Hexane/EtOAc = 19/1). It was prepared according to the general procedure described above. **^{1}H NMR (400 MHz, CDCl$_3$)** δ 7.79 – 7.71 (m, 1H), 7.56 – 7.50 (m, 1H), 7.37 – 7.29 (m, 4H), 7.27 – 7.14 (m, 8H), 3.50 – 3.38 (m, 2H), 2.77 – 2.55 (m, 4H), 1.44 (s, 9H). **^{13}C NMR (100 MHz, CDCl$_3$)** δ 169.44, 165.54, 151.02, 141.36, 141.03, 131.77, 128.57, 128.52, 128.30, 128.05, 126.14, 125.18, 124.45, 123.38, 120.35, 110.74, 84.52, 83.81, 82.82, 53.10, 35.54, 30.76, 28.01, 25.23. **ESI-MS:** calculated [$C_{30}H_{29}NO_3$ + Na]$^+$: 474.2040, found: 474.2047. $[\alpha]^{20}_D$ = 59.1 (c = 0.94, CH_2Cl_2). The product was analyzed by HPLC to determine the enantiomeric excess: 94% ee (CHIRALPAK AD-H, hexane/*i*-PrOH = 99/1, detector: 254 nm, T = 25 °C, flow rate: 1.0 mL/min), t_1(major) = 10.2 min, t_2(minor) = 15.7 min.

Tert-butyl (*S*)-2-(benzo[d]oxazol-2-yl)-2-isobutyl-5-phenylpent-4-ynoate (7i)

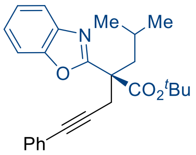

Colorless oil (54.9 mg, 91% yield). R_f = 0.45 (Hexane/EtOAc = 19/1). It was prepared according to the general procedure described above. **^{1}H NMR (400 MHz, CDCl$_3$)** δ 7.78 – 7.71 (m, 1H), 7.55 – 7.50 (m, 1H), 7.36 – 7.28 (m, 4H), 7.27 – 7.22 (m, 3H), 3.47 – 3.33 (m, 2H), 2.46 – 2.33 (m, 2H), 1.84 – 1.74 (m, 1H), 1.40 (s, 9H), 0.97 (d, *J* = 6.7 Hz, 3H), 0.79 (d, *J* = 6.6 Hz, 3H). **^{13}C NMR (100 MHz, CDCl$_3$)** δ 169.89, 166.23, 150.89, 141.04, 131.73, 128.27, 127.97, 125.13, 124.42, 123.51, 120.30, 110.69, 84.96, 83.73, 82.60, 52.79, 41.52, 27.92, 25.23, 24.45, 24.29, 23.76. **ESI-MS:** calculated [$C_{26}H_{29}NO_3$ + Na]$^+$: 426.2040, found: 426.2049. $[\alpha]^{20}_D$ = 49.0 (c = 1.08, CH_2Cl_2). The product was analyzed by HPLC to determine the enantiomeric excess: 92% ee (CHIRALPAK AD-H, hexane/*i*-PrOH = 99/1, detector: 254 nm, T = 25 °C, flow rate: 1.0 mL/min), t_1(major) = 6.4 min, t_2(minor) = 7.4 min.

Tert-butyl (*S*)-2-(benzo[d]oxazol-2-yl)-2-(cyclopropylmethyl)-5-phenylpent-4-ynoate (7j)

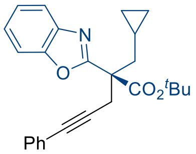

Colorless oil (54.6 mg, 91% yield). R_f = 0.40 (Hexane/EtOAc = 19/1). It was prepared according to the general procedure described above. **^{1}H NMR (400 MHz, CDCl$_3$)** δ 7.79 – 7.72 (m, 1H), 7.55 – 7.50 (m, 1H), 7.36 – 7.32 (m, 2H), 7.31 – 7.27 (m, 2H), 7.26 – 7.21 (m, 3H), 3.54 – 3.45 (m, 2H), 2.40 – 2.31 (m, 2H), 1.42 (s, 9H), 0.70 – 0.58 (m, 1H), 0.51 – 0.43 (m, 1H), 0.40 – 0.32 (m, 1H), 0.26 – 0.18 (m, 1H), 0.10 – 0.02 (m, 1H). **^{13}C NMR (100 MHz, CDCl$_3$)** δ 169.60, 165.96, 150.95, 141.04, 131.72, 128.27, 127.98, 125.11, 124.41, 123.46, 120.27, 110.69, 85.15, 83.75, 82.58, 53.80, 37.94, 27.96, 25.05, 6.14, 4.48, 4.05. **ESI-MS:** calculated [$C_{26}H_{27}NO_3$ + Na]$^+$: 424.1883, found: 424.1890. $[\alpha]^{20}_D$ = 41.1 (c = 1.08, CH_2Cl_2). The product was analyzed by HPLC to

determine the enantiomeric excess: 96% ee (CHIRALPAK AD-H, hexane/*i*-PrOH = 99/1, detector: 254 nm, T = 25 °C, flow rate: 1.0 mL/min), t_1(major) = 9.1 min, t_2(minor) = 11.1 min.

Methyl (*R*)-2-(benzo[d]oxazol-2-yl)-2-methyl-5-phenylpent-4-ynoate (7k)

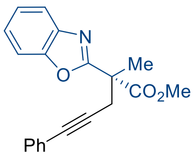

White solid (46.1 mg, 96% yield). R_f = 0.25 (Hexane/EtOAc = 19/1). Prepared according to the above general procedure, but (*S,S*)-BPE [(*S,S*)-**5d**] (10 mol%) was used instead of (*S,S$_p$*)-**5a**. **^{1}H NMR (500 MHz, $CDCl_3$)** δ 7.80 – 7.72 (m, 1H), 7.56 – 7.50 (m, 1H), 7.36 – 7.31 (m, 2H), 7.30 – 7.26 (m, 2H), 7.25 – 7.20 (m, 3H), 3.75 (s, 3H), 3.37 (s, 2H), 1.96 (s, 3H). **^{13}C NMR (125 MHz, $CDCl_3$)** δ 171.78, 165.61, 151.06, 140.94, 131.69, 128.22, 128.06, 125.29, 124.52, 123.13, 120.31, 110.81, 84.40, 83.88, 53.21, 48.86, 28.26, 21.36. **ESI-MS:** calculated $[C_{20}H_{17}NO_3 + Na]^+$: 342.1101, found: 342.1104. $[\alpha]^{20}_D$ = -271.0 (c = 0.93, CH_2Cl_2). The product was analyzed by HPLC to determine the enantiomeric excess: 90% ee (CHIRALPAK AD-H, hexane/*i*-PrOH = 98/2, detector: 254 nm, T = 25 °C, flow rate: 1.0 mL/min), t_1(minor) = 9.4 min, t_2(major) = 12.4 min.

Ethyl (*R*)-2-(benzo[d]oxazol-2-yl)-2-methyl-5-phenylpent-4-ynoate (7l)

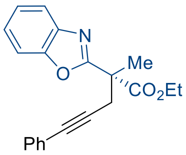

White solid (47.5 mg, 95% yield). R_f = 0.30 (Hexane/EtOAc = 19/1). Prepared according to the above general procedure, but (*S,S*)-BPE [(*S,S*)-**5d**] (10 mol%) was used instead of (*S,S$_p$*)-**5a**. **^{1}H NMR (500 MHz, $CDCl_3$)** δ 7.83 – 7.69 (m, 1H), 7.58 – 7.49 (m, 1H), 7.37 – 7.31 (m, 2H), 7.31 – 7.26 (m, 2H), 7.26 – 7.20 (m, 3H), 4.29 – 4.16 (m, 2H), 3.43 – 3.29 (m, 2H), 1.94 (s, 3H), 1.22 (t, *J* = 7.1 Hz, 3H). **^{13}C NMR (125 MHz, $CDCl_3$)** δ 171.27, 165.85, 151.12, 141.02, 131.74, 128.27, 128.08, 125.28, 124.52, 123.25, 120.35, 110.82, 84.56, 83.83, 62.23, 48.92, 28.27, 21.38, 14.18. **ESI-MS:** calculated $[C_{21}H_{19}NO_3 + H]^+$: 334.1438, found: 334.1439. $[\alpha]^{20}_D$ = -80.1 (c = 1.00, CH_2Cl_2). The product was analyzed by HPLC to determine the enantiomeric excess: 90% ee (CHIRALPAK AD-H, hexane/*i*-PrOH = 98/2, detector: 254 nm, T = 25 °C, flow rate: 1.0 mL/min), t_1(minor) = 8.6 min, t_2(major) = 12.1 min.

Isopropyl (*R*)-2-(benzo[d]oxazol-2-yl)-2-methyl-5-phenylpent-4-ynoate (7m)

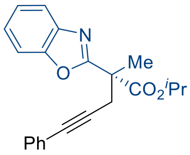

White solid (51.0 mg, 98% yield). R_f = 0.30 (Hexane/EtOAc = 19/1). The reaction was performed by using Ni(COD)$_2$ (10 mol%), (*S*)-Difluorphos [(*S*)-**4c**](10 mol%), $Cu(MeCN)_4BF_4$ (10 mol%), (*S,S*)-BPE [(*S,S*)-**5d**] (10 mol%) in 1,4-Dioxane at 10 °C for 72 h. **^{1}H NMR (500 MHz, $CDCl_3$)** δ 7.79 – 7.72 (m, 1H), 7.57 – 7.49 (m, 1H), 7.36 – 7.31 (m, 2H), 7.31 – 7.26 (m, 2H), 7.25 – 7.19 (m, 3H), 5.14 – 5.05 (m, 1H), 3.42 – 3.28 (m, 2H), 1.93 (s, 3H), 1.23 – 1.16 (m, 6H). **^{13}C NMR (125 MHz, $CDCl_3$)** δ 170.67, 165.96, 151.05, 140.97, 131.69, 128.23, 128.02, 125.20, 124.45, 123.23, 120.27, 110.72, 84.62, 83.72, 69.83, 48.92, 28.16, 21.60, 21.30. **ESI-MS:** calculated $[C_{22}H_{21}NO_3 + Na]^+$: 370.1414, found: 370.1418. $[\alpha]^{20}_D$ = -68.3 (c = 1.04, CH_2Cl_2). The product was analyzed by HPLC to determine the enantiomeric excess: 90% ee (CHIRALPAK AD-H, hexane/*i*-PrOH = 99/1, detector: 254 nm, T = 25 °C, flow rate: 1.0 mL/min), t_1(minor) = 9.5 min, t_2(major) = 13.1 min.

Tert-butyl (*S*)-2-(benzo[d]oxazol-2-yl)-2-(3-(4-fluorophenyl)prop-2-yn-1-yl)pent-4-enoate (7n)

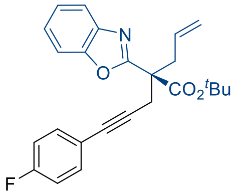

White solid (56.4 mg, 93% yield). R_f = 0.40 (Hexane/EtOAc = 19/1). It was prepared according to the general procedure described above. **^{1}H NMR (400 MHz, $CDCl_3$)** δ 7.79 – 7.72 (m, 1H), 7.56 – 7.50 (m, 1H), 7.38 – 7.32 (m, 2H), 7.29 – 7.24 (m, 2H), 6.99 – 6.87 (m, 2H), 5.76 – 5.64 (m, 1H), 5.29 – 5.20 (m, 1H), 5.18 – 5.10 (m, 1H), 3.37 – 3.24 (m, 2H), 3.21 – 3.08 (m, 2H), 1.42 (s, 9H). **^{13}C NMR (100 MHz, $CDCl_3$)** δ 169.02, 165.24, 162.40 (d, *J* = 247.4 Hz), 151.05, 140.97, 133.56 (d, *J* = 8.3 Hz) 131.91, 125.22, 124.49, 120.34, 120.15, 119.45 (d, *J* = 3.5 Hz), 115.53 (d, *J* = 22.0 Hz), 110.74, 84.32, 82.87, 82.75, 52.87, 37.89, 27.98, 24.87. **^{19}F NMR (375 MHz, $CDCl_3$)** δ -111.6 (s). **ESI-MS:** calculated $[C_{25}H_{24}FNO_3 + Na]^+$: 428.1632, found: 428.1644. $[\alpha]^{20}_D$ = 42.8 (c = 1.10, CH_2Cl_2). The product was analyzed by HPLC to determine the enantiomeric excess: 99% ee (CHIRALPAK AD-H, hexane/*i*-PrOH = 99/1, detector: 254 nm, T = 25 °C, flow rate: 1.0 mL/min), t_1(major) = 8.6 min, t_2(minor) = 10.0 min.

Tert-butyl (*S*)-2-(benzo[d]oxazol-2-yl)-2-(3-(4-chlorophenyl)prop-2-yn-1-yl)pent-4 -enoate (7o)

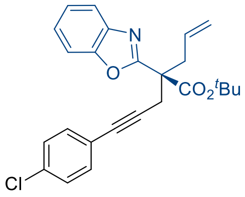

White solid (58.8 mg, 93% yield). R_f = 0.40 (Hexane/EtOAc = 19/1). Prepared according to the above general procedure, but the reaction was performed at 20 °C. **^{1}H NMR (500 MHz, $CDCl_3$)** δ 7.79 – 7.72 (m, 1H), 7.57 – 7.49 (m, 1H), 7.37 – 7.31 (m, 2H), 7.23 – 7.17 (m, 4H), 5.74 – 5.64 (m, 1H), 5.27 – 5.19 (m, 1H), 5.17 – 5.10 (m, 1H), 3.37 – 3.25 (m, 2H), 3.21 – 3.10 (m, 2H), 1.41 (s, 9H). **^{13}C NMR (125 MHz, $CDCl_3$)** δ 168.97, 165.16, 151.02, 140.91, 134.02, 132.95, 131.83, 128.61, 125.24, 124.50, 121.83, 120.33, 120.21, 110.74, 85.75, 82.91, 82.72, 52.81, 37.89, 27.96, 24.89. **ESI-MS:** calculated $[C_{25}H_{24}ClNO_3 + Na]^+$: 444.1337, found: 444.1349. $[\alpha]^{20}_D$ = 49.5 (c = 0.95, CH_2Cl_2). The product was analyzed by HPLC to determine the enantiomeric excess: 96% ee (CHIRALPAK AD-H, hexane/*i*-PrOH = 99/1, detector: 254 nm, T = 25 °C, flow rate: 1.0 mL/min), t_1(major) = 8.8 min, t_2(minor) = 9.7 min.

Tert-butyl (*S*)-2-(benzo[d]oxazol-2-yl)-2-(3-(4-bromophenyl)prop-2-yn-1-yl)pent-4 -enoate (7p)

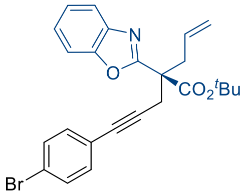

White solid (65.5 mg, 94% yield). R_f = 0.40 (Hexane/EtOAc = 19/1). It was prepared according to the general procedure described above. **^{1}H NMR (500 MHz, $CDCl_3$)** δ 7.79 – 7.72 (m, 1H), 7.57 – 7.51 (m, 1H), 7.41 – 7.32 (m, 4H), 7.18 – 7.12 (m, 2H), 5.74 – 5.64 (m, 1H), 5.27 – 5.20 (m, 1H), 5.16 – 5.11 (m, 1H), 3.34 – 3.24 (m, 2H), 3.19 – 3.09 (m, 2H), 1.41 (s, 9H). **^{13}C NMR (125 MHz, $CDCl_3$)** δ 168.97, 165.15, 151.03, 140.92, 133.19, 131.83, 131.55, 125.25, 124.51, 122.31, 122.22, 120.34, 120.22, 110.74, 85.98, 82.92, 82.79, 52.81, 37.90, 27.97, 24.93. **ESI-MS:** calculated $[C_{25}H_{24}BrNO_3 + Na]^+$: 488.0832, found: 488.0834. $[\alpha]^{20}_D$ = 50.1 (c = 1.08, CH_2Cl_2). The product was analyzed by HPLC to determine the enantiomeric excess: 98% ee (CHIRALPAK AD-H, hexane/*i*-PrOH = 99/1, detector: 254 nm, T = 25 °C, flow rate: 1.0 mL/min), t_1(major) = 9.8 min, t_2(minor) = 11.0 min.

Tert-butyl (*S*)-2-(benzo[d]oxazol-2-yl)-2-(3-(p-tolyl)prop-2-yn-1-yl)pent-4-enoate (7q)

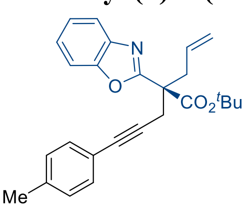

White solid (57.9 mg, 96% yield). $R_f = 0.40$ (Hexane/EtOAc = 19/1). It was prepared according to the general procedure described above. **^{1}H NMR (400 MHz, $CDCl_3$)** δ 7.79 – 7.72 (m, 1H), 7.56 – 7.49 (m, 1H), 7.37 – 7.31 (m, 2H), 7.23 – 7.15 (m, 2H), 7.09 – 7.01 (m, 2H), 5.77 – 5.64 (m, 1H), 5.28 – 5.19 (m, 1H), 5.17 – 5.09 (m, 1H), 3.37 – 3.23 (m, 2H), 3.22 – 3.10 (m, 2H), 2.31 (s, 3H), 1.42 (s, 9H). **^{13}C NMR (100 MHz, $CDCl_3$)** δ 169.09, 165.34, 151.05, 141.00, 138.05, 132.01, 131.62, 129.03, 125.15, 124.43, 120.34, 120.07, 110.73, 83.87, 83.81, 82.78, 52.94, 37.86, 27.99, 24.93, 21.54. **ESI-MS:** calculated $[C_{26}H_{27}NO_3 + Na]^+$: 424.1883, found: 424.1892. $[\alpha]^{20}_D = 47.6$ (c = 1.10, CH_2Cl_2). The product was analyzed by HPLC to determine the enantiomeric excess: 98% ee (CHIRALPAK AD-H, hexane/*i*-PrOH = 99/1, detector: 254 nm, T = 25 °C, flow rate: 1.0 mL/min), t_1(major) = 8.7 min, t_2(minor) = 9.9 min.

Tert-butyl (*S*)-2-(benzo[d]oxazol-2-yl)-2-(3-(4-methoxyphenyl)prop-2-yn-1-yl)pent-4 -enoate (7r)

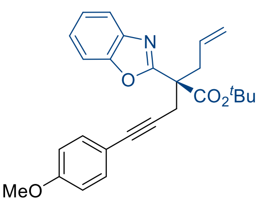

Colorless oil (59.9 mg, 96% yield). $R_f = 0.30$ (Hexane/EtOAc = 19/1). It was prepared according to the general procedure described above. **^{1}H NMR (400 MHz, $CDCl_3$)** δ 7.78 – 7.72 (m, 1H), 7.56 – 7.50 (m, 1H), 7.37 – 7.31 (m, 2H), 7.26 – 7.20 (m, 2H), 6.82 – 6.73 (m, 2H), 5.77 – 5.64 (m, 1H), 5.27 – 5.19 (m, 1H), 5.15 – 5.09 (m, 1H), 3.77 (s, 3H), 3.37 – 3.24 (m, 2H), 3.21 – 3.11 (m, 2H), 1.42 (s, 9H). **^{13}C NMR (100 MHz, $CDCl_3$)** δ 169.12, 165.38, 159.42, 151.05, 141.01, 133.11, 132.04, 125.15, 124.43, 120.33, 120.05, 115.59, 113.91, 110.73, 83.59, 83.00, 82.77, 55.38, 52.97, 37.86, 27.99, 24.93. **ESI-MS:** calculated $[C_{26}H_{27}NO_4 + Na]^+$: 440.1832, found: 440.1839. $[\alpha]^{20}_D = 51.8$ (c = 0.97, CH_2Cl_2). The product was analyzed by HPLC to determine the enantiomeric excess: 99% ee (CHIRALPAK AD-H, hexane/*i*-PrOH = 97/3, detector: 254 nm, T = 25 °C, flow rate: 1.0 mL/min), t_1(major) = 7.2 min, t_2(minor) = 7.9 min.

Tert-butyl (*S*)-2-(benzo[d]oxazol-2-yl)-2-(3-(3-chlorophenyl)prop-2-yn-1-yl)pent-4 -enoate (7s)

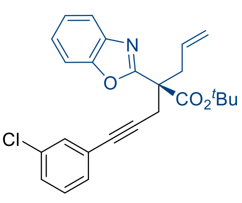

Colorless oil (59.4 mg, 94% yield). $R_f = 0.40$ (Hexane/EtOAc = 19/1). It was prepared according to the general procedure described above. **^{1}H NMR (400 MHz, $CDCl_3$)** δ 7.79 – 7.72 (m, 1H), 7.57 – 7.50 (m, 1H), 7.37 – 7.32 (m, 2H), 7.29 – 7.26 (m, 1H), 7.25 – 7.20 (m, 1H), 7.20 – 7.13 (m, 2H), 5.77 – 5.64 (m, 1H), 5.29 – 5.20 (m, 1H), 5.18 – 5.11 (m, 1H), 3.37 – 3.25 (m, 2H), 3.21 – 3.10 (m, 2H), 1.42 (s, 9H). **^{13}C NMR (100 MHz, $CDCl_3$)** δ 168.94, 165.14, 151.05, 140.95, 134.11, 131.83, 131.63, 129.88, 129.52, 128.36, 125.26, 125.07, 124.52, 120.37, 120.24, 110.74, 86.15, 82.94, 82.50, 52.82, 37.91, 27.99, 24.90. **ESI-MS:** calculated $[C_{25}H_{24}ClNO_3 + Na]^+$: 444.1337, found: 444.1350. $[\alpha]^{20}_D = 46.7$ (c = 0.97, CH_2Cl_2). The product was analyzed by HPLC to determine the enantiomeric excess: 97% ee (CHIRALPAK AD-H, hexane/*i*-PrOH = 99/1, detector: 254 nm, T = 25 °C, flow rate: 1.0 mL/min), t_1(major) = 7.0 min, t_2(minor) = 10.0 min.

Tert-butyl (*S*)-2-(benzo[d]oxazol-2-yl)-2-(3-(2-chlorophenyl)prop-2-yn-1-yl)pent-4-enoate (7t)

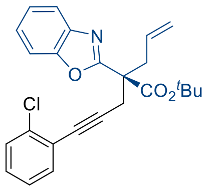

Colorless oil (58.2 mg, 92% yield). R_f = 0.35 (Hexane/EtOAc = 19/1). It was prepared according to the general procedure described above. **^{1}H NMR (400 MHz, $CDCl_3$)** δ 7.78 – 7.70 (m, 1H), 7.56 – 7.50 (m, 1H), 7.38 – 7.27 (m, 4H), 7.20 – 7.10 (m, 2H), 5.81 – 5.66 (m, 1H), 5.34 – 5.24 (m, 1H), 5.19 – 5.10 (m, 1H), 3.44 – 3.33 (m, 2H), 3.27 – 3.17 (m, 2H), 1.41 (s, 9H). **^{13}C NMR (100 MHz, $CDCl_3$)** δ 168.96, 165.18, 151.08, 141.05, 136.04, 133.52, 131.92, 129.23, 129.03, 126.38, 125.17, 124.44, 123.27, 120.35, 120.27, 110.74, 90.28, 82.91, 80.54, 52.85, 37.77, 27.99, 25.04. **ESI-MS:** calculated $[C_{25}H_{24}ClNO_3 + Na]^+$: 444.1337, found: 444.1346. $[\alpha]^{20}_D$ = 44.3 (c = 0.95, CH_2Cl_2). The product was analyzed by HPLC to determine the enantiomeric excess: 92% ee (CHIRALPAK AD-H, hexane/*i*-PrOH = 99/1, detector: 254 nm, T = 25 °C, flow rate: 1.0 mL/min), t_1(major) = 8.5 min, t_2(minor) = 13.2 min.

Tert-butyl (*S*)-2-(benzo[d]oxazol-2-yl)-2-(3-(m-tolyl)prop-2-yn-1-yl)pent-4-enoate (7u)

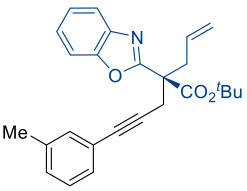

Colorless oil (54.7 mg, 91% yield). R_f = 0.40 (Hexane/EtOAc = 19/1). It was prepared according to the general procedure described above. **^{1}H NMR (500 MHz, $CDCl_3$)** δ 7.78 – 7.72 (m, 1H), 7.57 – 7.50 (m, 1H), 7.38 – 7.31 (m, 2H), 7.15 – 7.09 (m, 3H), 7.09 – 7.04 (m, 1H), 5.76 – 5.66 (m, 1H), 5.28 – 5.20 (m, 1H), 5.16 – 5.10 (m, 1H), 3.36 – 3.26 (m, 2H), 3.21 – 3.12 (m, 2H), 2.27 (s, 3H), 1.42 (s, 9H). **^{13}C NMR (125 MHz, $CDCl_3$)** δ 169.07, 165.31, 151.04, 140.98, 137.93, 132.35, 131.95, 128.91, 128.77, 128.17, 125.17, 124.44, 123.18, 120.33, 120.14, 110.75, 84.18, 83.95, 82.82, 52.90, 37.83, 27.98, 24.88, 21.29. **ESI-MS:** calculated $[C_{26}H_{27}NO_3 + Na]^+$: 424.1883, found: 424.1888. $[\alpha]^{20}_D$ = 47.4 (c = 1.08, CH_2Cl_2). The product was analyzed by HPLC to determine the enantiomeric excess: 97% ee (CHIRALPAK AD-H, hexane/*i*-PrOH = 99/1, detector: 254 nm, T = 25 °C, flow rate: 1.0 mL/min), t_1(major) = 6.7 min, t_2(minor) = 9.4 min.

Tert-butyl (*S*)-2-(benzo[d]oxazol-2-yl)-2-(3-(o-tolyl)prop-2-yn-1-yl)pent-4-enoate (7v)

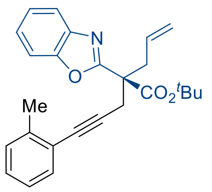

Colorless oil (54.3 mg, 90% yield). R_f = 0.40 (Hexane/EtOAc = 19/1). Prepared according to the above general procedure, but the reaction was performed at 20 °C. **^{1}H NMR (400 MHz, $CDCl_3$)** δ 7.78 – 7.70 (m, 1H), 7.56 – 7.50 (m, 1H), 7.36 – 7.31 (m, 2H), 7.28 (d, *J* = 7.5 Hz, 1H), 7.17 – 7.01 (m, 3H), 5.80 – 5.67 (m, 1H), 5.31 – 5.21 (m, 1H), 5.18 – 5.11 (m, 1H), 3.44 – 3.30 (m, 2H), 3.26 – 3.16 (m, 2H), 2.24 (s, 3H), 1.41 (s, 9H). **^{13}C NMR (100 MHz, $CDCl_3$)** δ 169.05, 165.32, 151.02, 140.97, 140.22, 132.10, 131.92, 129.37, 127.99, 125.48, 125.17, 124.44, 123.15, 120.31, 120.14, 110.71, 88.38, 82.82, 82.58, 52.88, 37.81, 27.97, 25.05, 20.72. **ESI-MS:** calculated $[C_{26}H_{27}NO_3 + Na]^+$: 424.1883, found: 424.1880. $[\alpha]^{20}_D$ = 47.8 (c = 1.08, CH_2Cl_2). The product was analyzed by HPLC to determine the enantiomeric excess: 92% ee (CHIRALPAK AD-H, hexane/*i*-PrOH = 99/1, detector: 254 nm, T = 25 °C, flow rate: 1.0 mL/min), t_1(major) = 6.5 min, t_2(minor) = 8.5 min.

Tert-butyl (*S*)-2-(benzo[d]oxazol-2-yl)-2-(3-(thiophen-2-yl)prop-2-yn-1-yl)pent-4-enoate (7w)

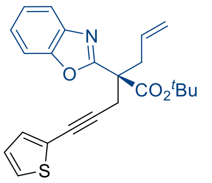

Colorless oil (55.6 mg, 94% yield). R_f = 0.40 (Hexane/EtOAc = 19/1). It was prepared according to the general procedure described above. **^{1}H NMR (500 MHz, $CDCl_3$)** δ 7.78 – 7.71 (m, 1H), 7.57 – 7.50 (m, 1H), 7.38 – 7.31 (m, 2H), 7.19 – 7.12 (m, 1H), 7.09 – 7.03 (m, 1H), 6.93 – 6.87 (m, 1H), 5.74 – 5.63 (m, 1H), 5.29 – 5.20 (m, 1H), 5.16 – 5.10 (m, 1H), 3.41 – 3.27 (m, 2H), 3.19 – 3.07 (m, 2H), 1.43 (s, 9H). **^{13}C NMR (125 MHz, $CDCl_3$)** δ 168.95, 165.11, 151.04, 140.93, 131.85, 131.67, 126.87, 126.58, 125.21, 124.47, 123.42, 120.36, 120.24, 110.76, 88.75, 82.95, 52.82, 37.94, 27.98, 25.19. **ESI-MS:** calculated $[C_{23}H_{23}NO_3S + Na]^+$: 416.1291, found: 416.1289. $[\alpha]^{20}_D$ = 41.3 (c = 1.08, CH_2Cl_2). The product was analyzed by HPLC to determine the enantiomeric excess: 98% ee (CHIRALPAK AD-H, hexane/*i*-PrOH = 99/1, detector: 254 nm, T = 25 °C, flow rate: 1.0 mL/min), t_1(major) = 8.7 min, t_2(minor) = 12.4 min.

Tert-butyl (S)-2-allyl-2-(benzo[d]oxazol-2-yl)hex-4-ynoate (7x)

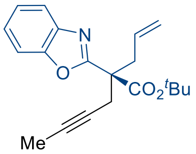

Colorless oil (44.0 mg, 90% yield). R_f = 0.40 (Hexane/EtOAc = 19/1). Prepared according to the above general procedure, but the reaction was performed at 20 °C. **^{1}H NMR (500 MHz, $CDCl_3$)** δ 7.77 – 7.69 (m, 1H), 7.54 – 7.48 (m, 1H), 7.38 – 7.30 (m, 2H), 5.71 – 5.61 (m, 1H), 5.22 – 5.15 (m, 1H), 5.12 – 5.06 (m, 1H), 3.13 – 2.98 (m, 4H), 1.71 (t, *J* = 2.5 Hz, 3H), 1.39 (s, 9H). **^{13}C NMR (125 MHz, $CDCl_3$)** δ 169.24, 165.50, 151.00, 140.97, 132.06, 125.08, 124.36, 120.30, 119.85, 110.70, 82.60, 79.11, 73.59, 52.84, 37.65, 27.94, 24.23, 3.62. **ESI-MS:** calculated $[C_{20}H_{23}NO_3 + Na]^+$: 348.1570, found: 348.1572. $[\alpha]^{20}_D$ = 12.5 (c = 0.88, CH_2Cl_2). The product was analyzed by HPLC to determine the enantiomeric excess: 89% ee (CHIRALPAK AD-H, hexane/*i*-PrOH = 99/1, detector: 270 nm, T = 25 °C, flow rate: 1.0 mL/min), t_1(major) = 5.4 min, t_2(minor) = 7.5 min.

Tert-butyl (*S*)-2-allyl-2-(benzo[d]oxazol-2-yl)-7-phenylhept-4-ynoate (7y)

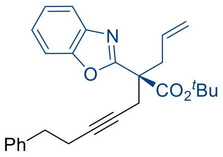

White solid (56.1 mg, 90% yield). R_f = 0.40 (Hexane/EtOAc = 19/1). Prepared according to the above general procedure, but the reaction was performed at 20 °C. **^{1}H NMR (500 MHz, $CDCl_3$)** δ 7.79 – 7.72 (m, 1H), 7.56 – 7.49 (m, 1H), 7.38 – 7.31 (m, 2H), 7.25 – 7.19 (m, 2H), 7.19 – 7.14 (m, 1H), 7.15 – 7.08 (m, 2H), 5.73 – 5.59 (m, 1H), 5.21 – 5.05 (m, 2H), 3.13 – 3.00 (m, 4H), 2.78 – 2.64 (m, 2H), 2.47 – 2.26 (m, 2H), 1.39 (s, 9H). **^{13}C NMR (125 MHz, $CDCl_3$)** δ 169.16, 165.48, 150.99, 140.97, 140.81, 132.01, 128.46, 128.40, 126.26, 125.09, 124.39, 120.29, 119.87, 110.69, 83.04, 82.59, 75.30, 52.80, 37.56, 35.31, 27.94, 24.21, 20.94. **ESI-MS:** calculated $[C_{27}H_{29}NO_3 + Na]^+$: 438.2040, found: 438.2038. $[\alpha]^{20}_D$ = 17.7 (c = 1.10, CH_2Cl_2). The product was analyzed by HPLC to determine the enantiomeric excess: 95% ee (CHIRALPAK AD-H, hexane/*i*-PrOH = 99/1, detector: 254 nm, T = 25 °C, flow rate: 1.0 mL/min), t_1(major) = 7.3 min, t_2(minor) = 9.9 min.

Tert-butyl (*S*)-2-(benzo[d]oxazol-2-yl)-2-(3-cyclohexylprop-2-yn-1-yl)pent-4-enoate (7z)

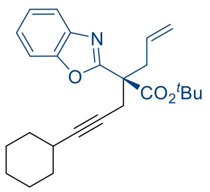

Colorless oil (53.2 mg, 90% yield). R_f = 0.45 (Hexane/EtOAc = 19/1). Prepared according to the above general procedure, but the reaction was performed at 20 °C. **^{1}H NMR (500 MHz, $CDCl_3$)** δ 7.76 – 7.69 (m, 1H), 7.55 – 7.48 (m, 1H), 7.35 – 7.29 (m, 2H), 5.72 – 5.61 (m, 1H), 5.23 – 5.16 (m, 1H), 5.12 – 5.06 (m, 1H), 3.12 – 3.02 (m, 4H), 2.33 – 2.22 (m, 1H), 1.70 – 1.48 (m, 5H), 1.39 (s, 9H), 1.35 – 1.17 (m, 5H). **^{13}C NMR (125 MHz, $CDCl_3$)** δ 169.20, 165.58, 150.98, 141.00, 132.14, 125.03, 124.33, 120.24, 119.81, 110.66, 88.16, 82.51, 74.52, 52.96, 37.63, 32.80, 28.95, 27.97, 26.01, 24.66, 24.26. **ESI-MS:** calculated $[C_{25}H_{31}NO_3 + Na]^+$: 416.2196, found: 416.2202. $[\alpha]^{20}_D$ = 16.0 (c = 1.04, CH_2Cl_2). The product was analyzed by HPLC to determine the enantiomeric excess: 98% ee (CHIRALPAK AD-H, hexane/*i*-PrOH = 99/1, detector: 254 nm, T = 25 °C, flow rate: 1.0 mL/min), t_1(major) = 5.1 min, t_2(minor) = 6.7 min.

Tert-butyl (*S*)-2-allyl-2-(benzo[d]oxazol-2-yl)dec-4-ynoate (7aa)

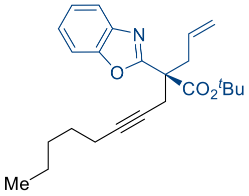

Colorless oil (52.9 mg, 92% yield). R_f = 0.45 (Hexane/EtOAc = 19/1). Prepared according to the above general procedure, but the reaction was performed at 20 °C. **^{1}H NMR (500 MHz, $CDCl_3$)** δ 7.76 – 7.68 (m, 1H), 7.53 – 7.46 (m, 1H), 7.35 – 7.27 (m, 2H), 5.73 – 5.60 (m, 1H), 5.23 – 5.15 (m, 1H), 5.14 – 5.06 (m, 1H), 3.17 – 2.96 (m, 4H), 2.09 – 2.02 (m, 2H), 1.41 – 1.31 (m, 11H), 1.26 – 1.18 (m, 4H), 0.82 (t, *J* = 7.0 Hz, 3H). **^{13}C NMR (125 MHz, $CDCl_3$)** δ 169.21, 165.54, 150.98, 140.98, 132.10, 125.04, 124.34, 120.27, 119.82, 110.67, 83.93, 82.54, 74.45, 52.88, 37.62, 30.97, 28.59, 27.95, 24.25, 22.24, 18.72, 14.05. **ESI-MS:** calculated $[C_{24}H_{31}NO_3 + Na]^+$: 404.2196, found: 404.2190. $[\alpha]^{20}_D$ = 18.8 (c = 1.02, CH_2Cl_2). The product was analyzed by HPLC to determine the enantiomeric excess: 91% ee (CHIRALPAK AD-H, hexane/*i*-PrOH = 99/1, detector: 254 nm, T = 25 °C, flow rate: 1.0 mL/min), t_1(major) = 4.1 min, t_2(minor) = 5.7 min.

Methyl (*S*)-2-(benzo[d]oxazol-2-yl)-2-methylhex-4-ynoate (7ab)

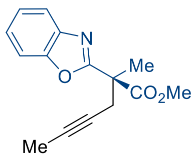

Colorless oil (27.3 mg, 71% yield). R_f = 0.25 (Hexane/EtOAc = 19/1). Prepared according to the above general procedure, but (*R*)-Difluorphos [(*R*)-**4c**] (10 mol%) was used instead of (*S*)-**4b**, and the reaction was performed at 20 °C. **^{1}H NMR (500 MHz, $CDCl_3$)** δ 7.77 – 7.71 (m, 1H), 7.54 – 7.48 (m, 1H), 7.38 – 7.29 (m, 2H), 3.72 (s, 3H), 3.08 (2.4 Hz, 2H), 1.86 (s, 3H), 1.70 (t, *J* = 2.4 Hz, 3H). **^{13}C NMR (125 MHz, $CDCl_3$)** δ 172.04, 165.89, 151.07, 141.00, 125.23, 124.48, 120.33, 110.84, 79.34, 73.53, 53.17, 48.80, 27.62, 21.21, 3.64. **ESI-MS:** calculated $[C_{15}H_{15}NO_3 + Na]^+$: 280.0944, found: 280.0949. $[\alpha]^{20}_D$ = 54.0 (c = 1.00, CH_2Cl_2). The product was analyzed by HPLC to determine the enantiomeric excess: 88% ee (CHIRALPAK AD-H, hexane/*i*-PrOH = 99/1, detector: 254 nm, T = 25 °C, flow rate: 1.0 mL/min), t_1(major) = 10.0 min, t_2(minor) = 13.0 min.

Ethyl (*R*)-2-(benzo[d]oxazol-2-yl)-2-(2-(benzyloxy)ethyl)hex-4-ynoate (7ac)

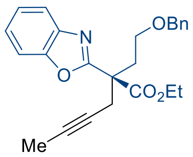

Colorless oil (52.7 mg, 90% yield). R_f = 0.40 (Hexane/EtOAc = 9/1). Prepared according to the above general procedure, but (*R*)-Difluorphos [(*R*)-**4c**] (10 mol%) was used instead of (*S*)-**4b**, and the reaction was performed at 20 ºC. **^{1}H NMR (500 MHz, $CDCl_3$)** δ 7.78 – 7.70 (m, 1H), 7.52 – 7.45 (m, 1H), 7.36 – 7.29 (m, 2H), 7.26 – 7.18 (m, 3H), 7.15 (d, *J* = 6.7 Hz, 2H), 4.37 (s, 2H), 4.18 – 4.05 (m, 2H), 3.63 (t, *J* = 6.2 Hz, 2H), 3.23 – 3.13 (m, 2H), 2.84 – 2.65 (m, 2H), 1.71 – 1.65 (m, 3H), 1.14 (t, *J* = 7.1 Hz, 3H). **^{13}C NMR (125 MHz, $CDCl_3$)** δ 170.66, 165.50, 150.99, 140.98, 138.15, 128.29, 127.57, 127.50, 125.12, 124.37, 120.26, 110.80, 79.33, 73.41, 73.09, 66.14, 62.01, 50.86, 33.18, 24.75, 14.04, 3.65. **ESI-MS:** calculated $[C_{24}H_{25}NO_4 + Na]^+$: 414.1676, found: 414.1686. $[\alpha]^{20}_D$ = 20.9 (c = 1.00, CH_2Cl_2). The product was analyzed by HPLC to determine the enantiomeric excess: 90% ee (CHIRALPAK AD-H, hexane/*i*-PrOH = 99/1, detector: 254 nm, T = 25 °C, flow rate: 1.0 mL/min), t_1(major) = 25.0 min, t_2(minor) = 28.2 min.

3 Synthetic utilities and stereodivergent transformations

3.1 Synthetic transformations of propargylated product 3a

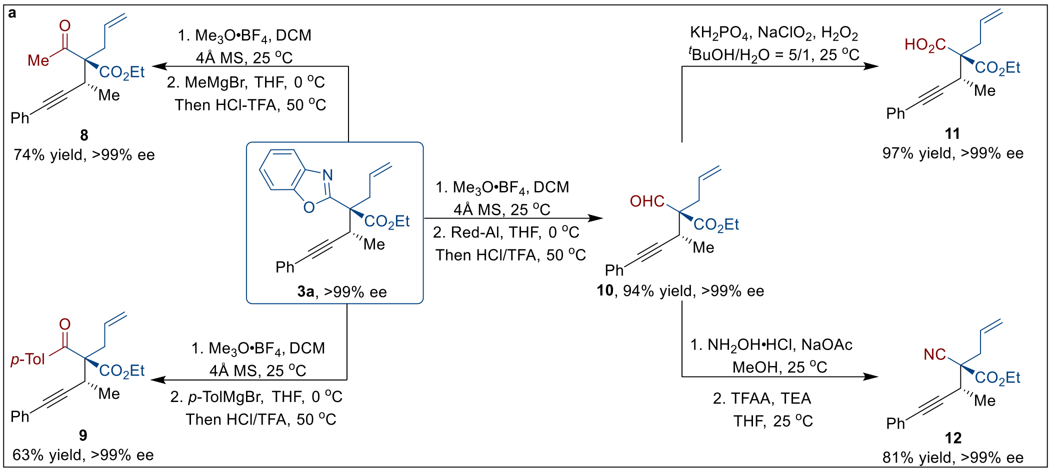

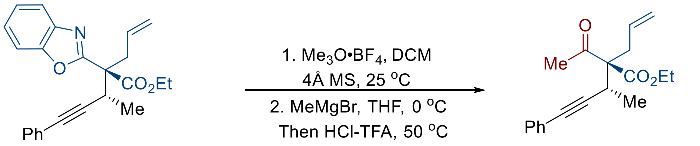

3a, >99% ee **8**, 74% yield, >99% ee

To a solution of **3a** (105.0 mg, 0.28 mmol) in dry DCM (3 mL) was added 4Å molecular sieves (200 mg), and the mixture was stirred vigorously for 10 minutes at 25 °C under an argon atmosphere. Trimethyloxonium tetrafluoroborate (414 mg, 2.8 mmol, 10.0 equiv.) was then added, and the mixture was stirred for 48 hours. After complete consumption of the starting material **3a**, the reaction mixture was concentrated by filtering off the molecular sieves to give the crude *N*-methylbenzoxazolium salt. The residue was dissolved in dry THF (2 mL) and cooled to 0 °C. A solution of MeMgBr (0.19 mL, 0.56 mmol, 2.0 equiv., 3.0 M in Et_2O) was added dropwise via syringe. The reaction mixture was stirred for 2 hours at 0 °C. Subsequently, 1 N aqueous hydrochloric acid solution (4 mL) and trifluoroacetic acid (214 µL, 2.8 mmol, 10.0 equiv.) were added sequentially. After stirring for 2 hours at 50 °C, the biphasic reaction mixture was cooled to 25 °C and extracted with ethyl acetate. The combined organic layers were dried over $MgSO_4$, filtered, and concentrated under vacuum. The residue was purified by silica gel chromatography to afford desired product **8** (61.7 mg, 74% yield).

Ethyl (*S*)-2-acetyl-2-((*R*)-4-phenylbut-3-yn-2-yl)pent-4-enoate (8)

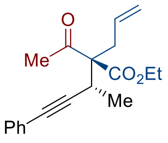

Colorless oil (61.7 mg, 74% yield, >20:1 dr). R_f = 0.30 (Hexane/EtOAc = 19/1). **^{1}H NMR (500 MHz, $CDCl_3$)** δ 7.41 – 7.35 (m, 2H), 7.32 – 7.26 (m, 3H), 5.78 – 5.66 (m, 1H), 5.19 – 5.06 (m, 2H), 4.32 – 4.20 (m, 2H), 3.43 (q, J = 7.0 Hz, 1H), 2.92 (dd, J = 14.4, 7.2 Hz, 1H), 2.68 (dd, J = 14.4, 7.6 Hz, 1H), 2.30 (s, 3H), 1.30 (t, J = 7.1 Hz, 3H), 1.26 (d, J = 7.0 Hz, 3H). **^{13}C NMR (125 MHz, $CDCl_3$)** δ 203.77, 170.61, 132.72, 131.60, 128.35, 128.05, 123.46, 119.16, 90.62, 83.58, 66.37, 61.46, 37.56, 29.99, 29.05, 17.04, 14.33. **ESI-MS:** calculated $[C_{19}H_{22}O_3 + Na]^+$: 321.1461, found: 321.1461. $[\alpha]^{20}_D$ = -46.7 (c = 1.00, CH_2Cl_2). The product was analyzed by HPLC to determine the enantiomeric excess: >99% ee (CHIRALPAK IC, hexane/*i*-PrOH = 99/1, detector: 254 nm, flow rate: 0.5 mL/min), t_1(minor) = 14.2 min, t_2(major) = 15.4 min.

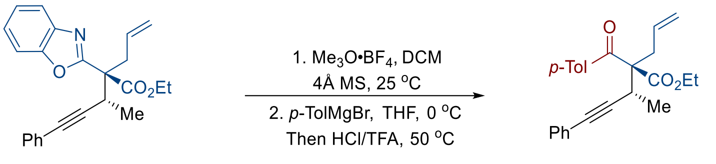

3a, >99% ee **9**, 63% yield, >99% ee

3a (63.3 mg, 0.17 mmol) and 4Å molecular sieves (150 mg) were added to dry DCM (2 mL), and the mixture was stirred vigorously for 10 minutes at 25 °C under an argon atmosphere. Trimethyloxonium tetrafluoroborate (251 mg, 1.7 mmol, 10.0 equiv.) was then added, and the mixture was stirred for 48 hours. After complete consumption of the starting material **3a**, the reaction mixture was concentrated by filtering off the molecular sieves to give the crude *N*-methylbenzoxazolium salt. The residue was dissolved in dry THF (1.5 mL) and cooled to 0 °C. A solution of *p*-TolMgBr (0.34 mL, 0.34 mmol, 2.0 equiv., 1.0 M in THF) was added dropwise via syringe. The reaction mixture was stirred for 1 hour at 0 °C. Subsequently, 1 N aqueous hydrochloric acid solution (2 mL) and trifluoroacetic acid (130 μL, 1.7 mmol, 10.0 equiv.) were added sequentially. After stirring for 2 hours at 50 °C, the biphasic reaction mixture was cooled to 25 °C and extracted with ethyl acetate. The combined organic layers were dried over $MgSO_4$, filtered, and concentrated under vacuum. The residue was purified by silica gel chromatography to afford desired product **9** (40.3 mg, 63% yield).

Ethyl (*S*)-2-(4-methylbenzoyl)-2-((*R*)-4-phenylbut-3-yn-2-yl)pent-4-enoate (9)

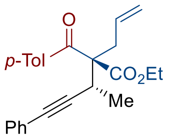

Colorless oil (40.3 mg, 63% yield, >20:1 dr). R_f = 0.35 (Hexane/EtOAc = 19/1). **^{1}H NMR (400 MHz, $CDCl_3$)** δ 7.74 (d, *J* = 8.2 Hz, 2H), 7.46 – 7.33 (m, 2H), 7.32 – 7.24 (m, 3H), 7.20 (d, *J* = 8.1 Hz, 2H), 5.78 – 5.54 (m, 1H), 5.15 – 4.89 (m, 2H), 4.30 – 4.09 (m, 2H), 3.67 (q, *J* = 6.8 Hz, 1H), 3.33 (dd, *J* = 14.6, 8.1 Hz, 1H), 2.95 (dd, *J* = 14.5, 6.8 Hz, 1H), 2.39 (s, 3H), 1.38 (d, *J* = 6.9 Hz, 3H), 1.13 (t, *J* = 7.1 Hz, 3H). **^{13}C NMR (100 MHz, $CDCl_3$)** δ 194.91, 171.39, 143.54, 133.84, 132.20, 131.70, 129.14, 128.88, 128.28, 127.89, 123.73, 119.55, 90.54, 83.89, 63.43, 61.41, 38.81, 30.90, 21.69, 17.86, 14.08. **ESI-MS:** calculated $[C_{25}H_{26}O_3 + Na]^+$: 397.1774, found: 397.1790. $[\alpha]^{20}_D$ = -83.3 (c = 0.93, CH_2Cl_2). The product was analyzed by HPLC to determine the enantiomeric excess: >99% ee (CHIRALPAK IG, hexane/*i*-PrOH = 99/1, detector: 254 nm, flow rate: 0.5 mL/min), t_1(major) = 25.3 min, t_2(minor) = 33.4 min.

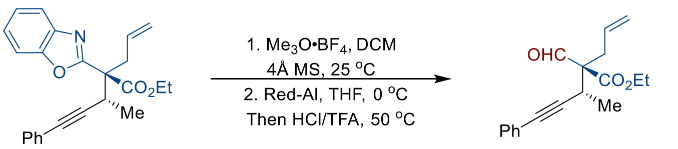

3a, >99% ee **10**, 94% yield, >99% ee

To a solution of **3a** (100.0 mg, 0.268 mmol) in dry DCM (3 mL) was added 4Å molecular sieves (200 mg), and the mixture was stirred vigorously for 10 minutes at 25 °C under an argon atmosphere. Trimethyloxonium tetrafluoroborate (396 mg, 2.68 mmol, 10.0 equiv.) was then added, and the mixture was stirred for 48 hours. After complete consumption of the starting material **3a**, the reaction mixture was concentrated by filtering off the molecular sieves to give the crude *N*-methylbenzoxazolium salt. The crude product was then dissolved in dry THF (2 mL) and cooled to 0 °C. Sodium bis(2-methoxyethoxy)aluminum hydride in toluene (Red-Al, 70% wt, 153 μL, 0.536 mmol, 2.0 equiv.) was added dropwise to the solution via a syringe. The

reaction mixture was stirred for 2 hours at 0 °C. Subsequently, 1 N aqueous hydrochloric acid solution (4 mL) and trifluoroacetic acid (205 μL, 2.68 mmol, 10.0 equiv.) were added sequentially. After stirring for 2 hours at 50 °C, the biphasic reaction mixture was cooled to 25 °C and extracted with ethyl acetate. The combined organic layers were dried over $MgSO_4$, filtered, and concentrated under vacuum. The residue was purified by silica gel chromatography to afford desired product **10** (71.8 mg, 94% yield).

Ethyl (*S*)-2-formyl-2-((*R*)-4-phenylbut-3-yn-2-yl)pent-4-enoate (10)

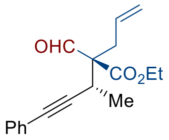

Colorless oil (71.8 mg, 94% yield, >20:1 dr). R_f = 0.40 (Hexane/EtOAc = 19/1). **^{1}H NMR (500 MHz, $CDCl_3$)** δ 9.99 (s, 1H), 7.40 – 7.34 (m, 2H), 7.31 – 7.26 (m, 3H), 5.81 – 5.66 (m, 1H), 5.17 – 5.05 (m, 2H), 4.33 – 4.22 (m, 2H), 3.37 (q, *J* = 7.1 Hz, 1H), 2.81 – 2.72 (m, 1H), 2.53 (dd, *J* = 14.0, 8.1 Hz, 1H), 1.30 (t, *J* = 7.1 Hz, 3H), 1.22 (d, *J* = 7.2 Hz, 3H). **^{13}C NMR (125 MHz, $CDCl_3$)** δ 199.77, 170.55, 132.32, 131.68, 128.39, 128.26, 123.12, 119.41, 89.20, 84.21, 63.03, 61.62, 35.32, 30.45, 15.98, 14.40. **ESI-MS:** calculated $[C_{18}H_{20}O_3 + Na]^+$: 307.1305, found: 307.1306. $[\alpha]^{20}_D$ = 6.5 (c = 0.88, CH_2Cl_2). The product was analyzed by HPLC to determine the enantiomeric excess: >99% ee (CHIRALPAK AS-H, hexane/*i*-PrOH = 99/1, detector: 254 nm, flow rate: 0.5 mL/min), t_1(minor) = 10.9 min, t_2(major) = 13.4 min.

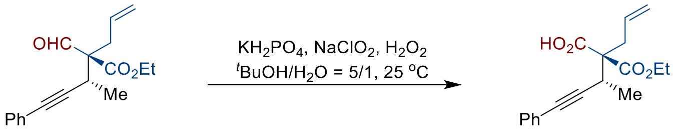

10, >99% ee **11**, 97% yield, >99% ee

Compound **10** (34.1 mg, 0.12 mmol) was dissolved in 3 mL of a mixture of *tert*-butanol: H_2O (5:1), then KH_2PO_4 (32.7 mg, 0.24 mmol, 2.0 equiv.), H_2O_2 (0.1 mL of a 30% solution) and $NaClO_2$ (43.4 mg, 0.48 mmol, 4.0 equiv.) were added. The resulting solution was stirred at 25 °C for 12 hours and then quenched with a hydrochloric acid solution (1.0 M). The resulting solution was extracted with ethyl acetate. The combined organic layers were dried over Na_2SO_4, filtered, and concentrated in vacuo. The residue was purified by flash column chromatography on silica gel to give desired product **11** (35.0 mg, 97% yield).

(*S*)-2-(ethoxycarbonyl)-2-((*R*)-4-phenylbut-3-yn-2-yl)pent-4-enoic acid (11)

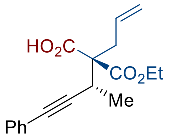

Colorless oil (35.0 mg, 97% yield, >20:1 dr). R_f = 0.40 (DCM/MeOH = 9/1). **^{1}H NMR (500 MHz, $CDCl_3$)** δ 7.43 – 7.35 (m, 2H), 7.32 – 7.26 (m, 3H), 5.91 – 5.65 (m, 1H), 5.25 – 5.03 (m, 2H), 4.38 – 4.22 (m, 2H), 3.39 (q, *J* = 7.0 Hz, 1H), 3.05 – 2.79 (m, 2H), 1.38 (d, *J* = 7.0 Hz, 3H), 1.32 (t, *J* = 7.1 Hz, 3H). **^{13}C NMR (125 MHz, $CDCl_3$)** δ 173.24, 172.50, 132.30, 131.67, 128.37, 128.18, 123.27, 119.78, 89.04, 84.05, 62.52, 61.21, 39.32, 32.82, 17.48, 14.21. **ESI-MS:** calculated $[C_{18}H_{20}O_4 + Na]^+$: 323.1254, found: 323.1256. $[\alpha]^{20}_D$ = -42.1 (c = 1.05, CH_2Cl_2). The product was analyzed by HPLC to determine the enantiomeric excess: >99% ee (CHIRALPAK AD-H, hexane/*i*-PrOH/AcOH = 98/2/0.1, detector: 254 nm, flow rate: 0.5 mL/min), t_1(major) = 29.8 min, t_2(minor) = 34.7 min.

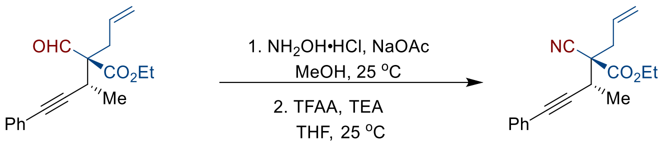

10, >99% ee **12**, 81% yield, >99% ee

NH_2OH•HCl (28.5 mg, 0.41 mmol, 1.5 equiv.) and sodium acetate (33.6 mg, 0.41 mmol, 1.5 equiv.) were added to a solution of **10** (77.0 mg, 0.27 mmol) in MeOH (2 mL), and the resulting mixture was stirred at room temperature for 12 hours. Saturated $NaHCO_3$ aq. was added, and the mixture was extracted with CH_2Cl_2 (three times). The organic layers were combined, dried over Na_2SO_4, and concentrated in vacuo. The crude product was then dissolved in anhydrous tetrahydrofuran (2 mL), and then triethylamine (273.2 mg, 2.7 mmol, 10.0 equiv.) and trifluoroacetic anhydride (283.5 mg, 1.35 mmol, 5.0 equiv.) were added to the stirred solution. The resulting mixture was stirred at room temperature for 12 hours. After completion of the reaction, the mixture was poured into water, neutralized with a 10% solution of $NaHCO_3$, and extracted with ethyl acetate. The combined organic layers were dried over Na_2SO_4, filtered, and concentrated in vacuo. The residue was purified by silica gel chromatography to afford desired product **12** (61.6 mg, 81% yield).

Ethyl (*S*)-2-cyano-2-((*R*)-4-phenylbut-3-yn-2-yl)pent-4-enoate (12)

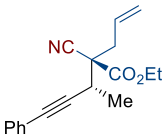

Colorless oil (61.6 mg, 81% yield, >20:1 dr). R_f = 0.30 (Hexane/EtOAc = 19/1). **^{1}H NMR (500 MHz, $CDCl_3$)** δ 7.44 – 7.36 (m, 2H), 7.33 – 7.26 (m, 3H), 5.90 – 5.74 (m, 1H), 5.36 – 5.18 (m, 2H), 4.35 – 4.18 (m, 2H), 3.24 (q, J = 7.0 Hz, 1H), 2.76 – 2.62 (m, 2H), 1.48 (d, J = 7.0 Hz, 3H), 1.30 (t, J = 7.1 Hz, 3H). **^{13}C NMR (125 MHz, $CDCl_3$)** δ 167.31, 131.83, 130.56, 128.48, 128.36, 122.69, 121.29, 117.61, 87.29, 84.82, 62.94, 54.82, 39.14, 33.70, 16.69, 14.28. **ESI-MS:** calculated $[C_{18}H_{19}NO_2 + Na]^+$: 304.1308, found: 304.1312. $[\alpha]^{20}_D$ = -9.2 (c = 0.75, CH_2Cl_2). The product was analyzed by HPLC to determine the enantiomeric excess: >99% ee (CHIRALCEL OJ-H, hexane/*i*-PrOH = 99/1, detector: 254 nm, flow rate: 0.5 mL/min), t_1(major) = 27.8 min, t_2(minor) = 31.3 min.

3.2 Concise asymmetric synthesis of cyclic unsaturated ketone 15

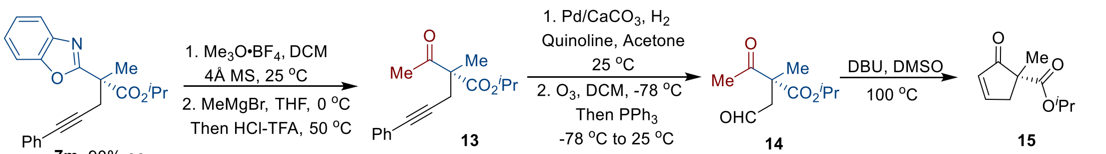

7m, 90% ee **13** 91% yield, 90% ee **14** 64% yield **15** 70% yield, 90% ee

To a solution of **7m** (104.2 mg, 0.3 mmol) in dry DCM (3 mL) was added 4Å molecular sieves (200 mg), and the mixture was stirred vigorously for 10 minutes at 25 °C under an argon atmosphere. Trimethyloxonium tetrafluoroborate (443.7 mg, 3.0 mmol, 10.0 equiv.) was then added, and the mixture was stirred for 48 hours. After complete consumption of the starting material **7m**, the reaction mixture was concentrated by filtering off the molecular sieves to give the crude *N*-methylbenzoxazolium salt. The residue was dissolved in dry THF (2 mL) and cooled to 0 °C. A solution of MeMgBr (0.2 mL, 0.6 mmol, 2.0 equiv., 3.0 M in Et_2O) was added dropwise via syringe. The reaction mixture was stirred for 2 hours at 0 °C. Subsequently, 1 N aqueous hydrochloric acid solution (4 mL) and trifluoroacetic acid (230 μL, 3.0 mmol, 10.0 equiv.) were added sequentially. After stirring for 2 hours at 50 °C, the biphasic reaction

mixture was cooled to 25 °C and extracted with ethyl acetate. The combined organic layers were dried over $MgSO_4$, filtered, and concentrated under vacuum. The residue was purified by silica gel chromatography to afford desired product **13** (74.5 mg, 91% yield).

Isopropyl (*R*)-2-acetyl-2-methyl-5-phenylpent-4-ynoate (13)

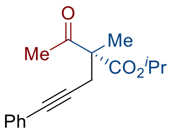

Colorless oil (74.5 mg, 91% yield). R_f = 0.35 (Hexane/EtOAc = 19/1). **^{1}H NMR (500 MHz, $CDCl_3$)** δ 7.39 – 7.32 (m, 2H), 7.29 – 7.23 (m, 3H), 5.15 – 5.05 (m, 1H), 2.97 (d, *J* = 17.1 Hz, 1H), 2.89 (d, *J* = 17.1 Hz, 1H), 2.22 (s, 3H), 1.53 (s, 3H), 1.28 – 1.24 (m, 6H). **^{13}C NMR (125 MHz, $CDCl_3$)** δ 204.20, 171.14, 131.69, 128.31, 128.05, 123.31, 85.05, 83.50, 69.42, 59.48, 26.19, 25.94, 21.65, 21.63, 19.35. **ESI-MS:** calculated $[C_{17}H_{20}O_3 + Na]^+$: 295.1305, found: 295.1311. $[\alpha]^{20}_D$ = 56.6 (c = 0.65, CH_2Cl_2). The product was analyzed by HPLC to determine the enantiomeric excess: 90% ee (CHIRALPAK IG, hexane/*i*-PrOH = 99/1, detector: 254 nm, flow rate: 1.0 mL/min), t_1(major) = 11.5 min, t_2(minor) = 13.9 min.

The reaction flask with hydrogen balloon was charged with **13** (150.0 mg, 0.55 mmol), Lindlar catalyst (Pd/$CaCO_3$, 58.9 mg), quinoline (71.0 mg, 0.55 mmol, 1.0 equiv.), and acetone (3 mL) was used. After stirring the mixture at 25 °C for 4 hours (monitored by TLC), the catalyst was removed through a short celite, and the resulting solvent was concentrated under reduced pressure to obtain the crude olefin product, which did not require purification and could proceed directly to the next reaction. The crude olefin product was dissolved in DCM (2 mL) and cooled to -78 °C. Ozone was bubbled into the mixture while the temperature was kept at -78 °C. When the color turned to blue, the ozone bubbling was stopped. Oxygen gas was passed through the solution for 3 minutes. PPh_3 (288.5 mg, 1.1 mmol, 2.0 equiv.) was then added and the reaction mixture was allowed to warm to 25 °C. After stirring at 25 °C for 2 hours, the mixture was concentrated in vacuo. The residue was purified by chromatography on silica gel to afford product **14** (70.6 mg, 64% yield).

Isopropyl (*R*)-2-acetyl-2-methyl-4-oxobutanoate (14)

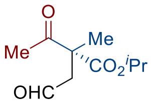

Colorless oil (70.6 mg, 64% yield). R_f = 0.30 (Hexane/EtOAc = 19/1). **^{1}H NMR (500 MHz, $CDCl_3$)** δ 9.70 (s, 1H), 5.10 – 5.02 (m, 1H), 2.97 (d, *J* = 17.8 Hz, 1H), 2.87 (d, *J* = 17.8 Hz, 1H), 2.24 (s, 3H), 1.49 (s, 3H), 1.25 – 1.21 (m, 6H). **^{13}C NMR (125 MHz, $CDCl_3$)** δ 204.82, 199.24, 171.21, 69.76, 57.20, 48.67, 26.14, 21.60, 21.57, 20.57. **ESI-MS:** calculated $[C_{10}H_{16}O_4 + Na]^+$: 223.0941, found: 223.0936. $[\alpha]^{20}_D$ = 43.1 (c = 1.00, CH_2Cl_2).

To a stirred solution of **14** (30 mg, 0.15 mmol) in DMSO (3 mL) was dropwise added 1,8-diazabicyclo[5.4.0]undec-7-ene (22.8 mg, 0.15 mmol, 1.0 equiv.) at room temperature. After the reaction mixture was stirred for 30 min at 100 °C, the reaction was quenched by adding 1 M HCl aqueous solution and extraction with Et_2O. The combined organic layers were washed with brine, and dried over $MgSO_4$. After the solvent was removed in vacuo, the resulting residue was purified by column chromatography to afford product **15** (19.2 mg, 70% yield).

Isopropyl (*R*)-1-methyl-2-oxocyclopent-3-ene-1-carboxylate (15)

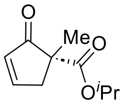

Colorless oil (19.2 mg, 70% yield). R_f = 0.35 (Hexane/EtOAc = 9/1). **^{1}H NMR (500 MHz, $CDCl_3$)** δ 7.78 – 7.65 (m, 1H), 6.22 – 6.10 (m, 1H), 5.02 – 4.93 (m, 1H), 3.27 – 3.12 (m, 1H), 2.59 – 2.46 (m, 1H), 1.37 (s, 3H), 1.22 – 1.16 (m, 6H).

^{13}C NMR (125 MHz, CDCl$_3$)** δ 207.03, 171.18, 163.19, 131.82, 69.01, 53.51, 42.86, 21.67, 21.60, 20.61. **ESI-MS:** calculated [$C_{10}H_{14}O_3$ + Na]$^+$: 205.0835, found: 205.0837. $[\alpha]^{20}_D$ = 24.3 (c = 0.75, CH_2Cl_2). The product was analyzed by HPLC to determine the enantiomeric excess: 90% ee (CHIRALPAK AD-H, hexane/*i*-PrOH = 99/1, detector: 230 nm, flow rate: 1.0 mL/min), t_1(minor) = 8.7 min, t_2(major) = 9.5 min.

3.3 Concise asymmetric formal synthesis of (-)-Yezo'otogirin G

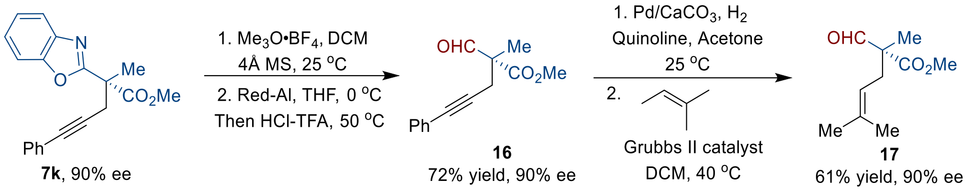

To a solution of **7k** (95.8 mg, 0.3 mmol) in dry DCM (3 mL) was added 4Å molecular sieves (200 mg), and the mixture was stirred vigorously for 10 minutes at 25 °C under an argon atmosphere. Trimethyloxonium tetrafluoroborate (443.7 mg, 3.0 mmol, 10.0 equiv.) was then added, and the mixture was stirred for 48 hours. After complete consumption of the starting material **7k**, the reaction mixture was concentrated by filtering off the molecular sieves to give the crude *N*-methylbenzoxazolium salt. The crude product was then dissolved in dry THF (2 mL) and cooled to 0 °C. Sodium bis(2-methoxyethoxy)aluminum hydride in toluene (Red-Al, 70% wt, 171 μL, 0.6 mmol, 2.0 equiv.) was added dropwise to the solution via a syringe. The reaction mixture was stirred for 2 hours at 0 °C. Subsequently, 1 N aqueous hydrochloric acid solution (4 mL) and trifluoroacetic acid (230 μL, 3.0 mmol, 10.0 equiv.) were added sequentially. After stirring for 2 hours at 50 °C, the biphasic reaction mixture was cooled to 25 °C and extracted with ethyl acetate. The combined organic layers were dried over $MgSO_4$, filtered, and concentrated under vacuum. The residue was purified by silica gel chromatography to afford desired product **16** (50.0 mg, 72% yield).

Methyl (*R*)-2-formyl-2-methyl-5-phenylpent-4-ynoate (16)

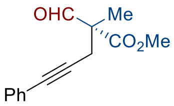

Colorless oil (50.0 mg, 72% yield). R_f = 0.40 (Hexane/EtOAc = 9/1). **^{1}H NMR (500 MHz, CDCl$_3$)** δ 9.68 (s, 1H), 7.31 – 7.27 (m, 2H), 7.23 – 7.19 (m, 3H), 3.72 (s, 3H), 2.91 (d, *J* = 17.0 Hz, 1H), 2.84 (d, *J* = 17.0 Hz, 1H), 1.44 (s, 3H). **^{13}C NMR (125 MHz, CDCl$_3$)** δ 197.88, 171.42, 131.75, 128.36, 128.27, 123.03, 84.00, 83.88, 57.37, 53.00, 24.82, 17.37. **ESI-MS:** calculated [$C_{14}H_{14}O_3$ + Na]$^+$: 253.0835, found: 253.0843. $[\alpha]^{20}_D$ = 7.4 (c = 0.50, CH_2Cl_2). The product was analyzed by HPLC to determine the enantiomeric excess: 90% ee (CHIRALPAK AS-H, hexane/*i*-PrOH = 99/1, detector: 254 nm, flow rate: 1.0 mL/min), t_1(major) = 11.5 min, t_2(minor) = 12.4 min.

The reaction flask with hydrogen balloon was charged with **16** (50.0 mg, 0.217 mmol), Lindlar catalyst ($Pd/CaCO_3$, 10 mg), quinoline (56.0 mg, 0.434 mmol, 2.0 equiv.), and acetone (2 mL). After stirring the mixture at 25 °C for 3 hours (monitored by TLC), the catalyst was removed through a short celite. The resulting solvent was concentrated under reduced pressure to obtain the crude olefin product, which did not require purification and could proceed directly to the next step. The crude olefin product was taken to a Schlenk tube and mixed with Grubbs second-generation catalyst (28.0 mg, 0.033 mmol, 15 mmol%), dichloromethane (1.5 mL), and 2-methylbut-2-ene (230 μL, 2.17 mmol, 10.0 equiv.) under a nitrogen atmosphere. The reaction

mixture was stirred at 40 °C for 24 hours. The reaction mixture was then cooled to room temperature, diluted with water, and extracted with dichloromethane three times. The combined organic layers were dried over anhydrous Na_2SO_4 and concentrated under reduced pressure. Finally, the resulting residue was purified by column chromatography on silica gel to afford product **17** (24.5 mg, 61% yield).

Methyl (*R*)-2-formyl-2,5-dimethylhex-4-enoate (17)

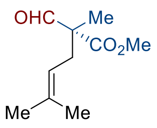

Colorless oil (24.5 mg, 61% yield). R_f = 0.30 (Hexane/EtOAc = 19/1). **^{1}H NMR (500 MHz, CDCl$_3$)** δ 9.69 (s, 1H), 5.07 – 4.93 (m, 1H), 3.74 (s, 3H), 2.55 (dd, *J* = 14.4, 7.7 Hz, 1H), 2.47 (dd, *J* = 14.4, 7.5 Hz, 1H), 1.69 (s, 3H), 1.60 (s, 3H), 1.27 (s, 3H). **^{13}C NMR (125 MHz, CDCl$_3$)** δ 199.85, 172.71, 136.40, 117.15, 58.15, 52.54, 33.16, 26.07, 18.00, 16.63. **ESI-MS:** calculated $[C_{10}H_{16}O_3 + Na]^+$: 207.0992, found: 207.0992. $[\alpha]^{20}_D$ = 4.8 (c = 0.73, CH_2Cl_2). The product was analyzed by HPLC to determine the enantiomeric excess: 90% ee (CHIRALPAK IE, hexane/*i*-PrOH = 99/1, detector: 220 nm, flow rate: 0.5 mL/min), t_1(minor) = 29.9 min, t_2(major) = 36.9 min.

3.4 Stereodivergent total synthesis of all four stereoisomers of Methohexital

3.4.1 Total synthesis of (*S,S*)-Methohexital

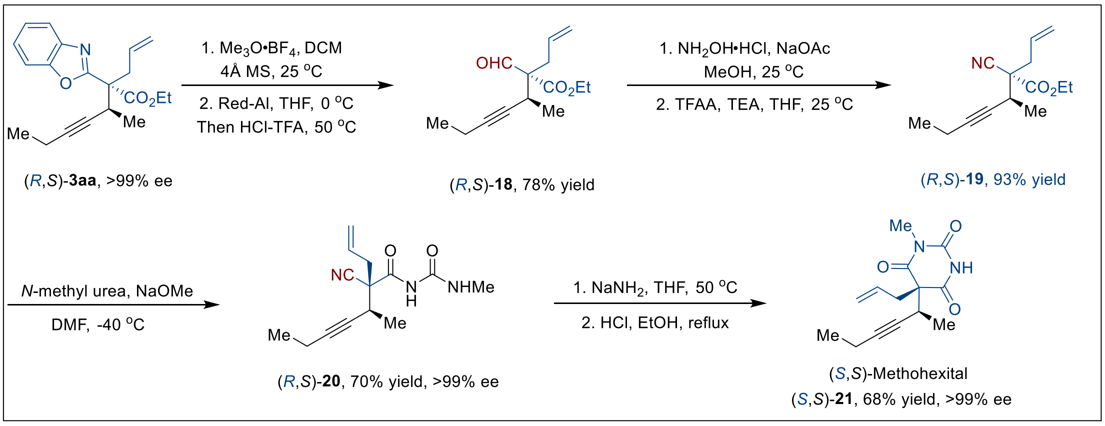

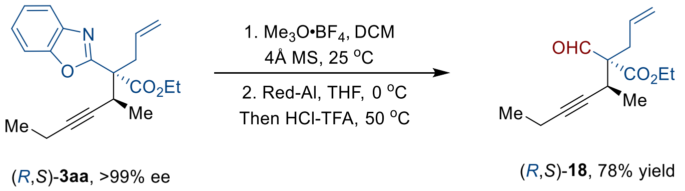

To a solution of (*R,S*)-**3aa** (390.5 mg, 1.2 mmol) in dry DCM (12 mL) was added 4Å molecular sieves (800 mg), and the mixture was stirred vigorously for 10 minutes at 25 °C under an argon atmosphere. Trimethyloxonium tetrafluoroborate (1775 mg, 12 mmol, 10.0 equiv.) was then added, and the mixture was stirred for 48 hours. After complete consumption of the starting material (*R,S*)-**3aa**, the reaction mixture was concentrated by filtering off the molecular sieves to give the crude *N*-methylbenzoxazolium salt. The crude product was then dissolved in dry THF (8 mL) and cooled to 0 °C. Sodium bis(2-methoxyethoxy)aluminum hydride in toluene (Red-Al, 70% wt, 686 μL, 2.4 mmol, 2.0 equiv.) was added to the solution dropwise via syringe. The reaction mixture was stirred for 2 hours at 0 °C. Subsequently, 1 N aqueous hydrochloric acid solution (16 mL) and trifluoroacetic acid (919 μL, 12 mmol, 10.0

equiv.) were added sequentially. After stirring for 2 hours at 50 °C, the biphasic reaction mixture was cooled to 25 °C and extracted with ethyl acetate. The combined organic layers were dried over $MgSO_4$, filtered, and concentrated under vacuum. The residue was purified by silica gel chromatography to afford the desired product (*R,S*)-**18** (222.3 mg, 78% yield).

Ethyl (2*R*,3*S*)-2-allyl-2-formyl-3-methylhept-4-ynoate ((*R*,*S*)-18)

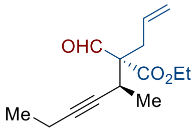

Colorless oil (78% yield, >20:1 dr). R_f = 0.50 (Hexane/EtOAc = 19/1). **^{1}H NMR (500 MHz, $CDCl_3$)** δ 9.91 (s, 1H), 5.78 – 5.62 (m, 1H), 5.15 – 4.98 (m, 2H), 4.24 (q, *J* = 7.1 Hz, 2H), 3.15 – 3.03 (m, 1H), 2.68 (dd, *J* = 14.0, 6.4 Hz, 1H), 2.39 (dd, *J* = 13.9, 8.4 Hz, 1H), 2.15 (qd, *J* = 7.5, 2.3 Hz, 2H), 1.29 (t, *J* = 7.1 Hz, 3H), 1.13 – 1.05 (m, 6H). **^{13}C NMR (125 MHz, $CDCl_3$)** δ 200.29, 170.77, 132.67, 119.03, 85.86, 78.99, 62.97, 61.42, 34.94, 30.13, 16.11, 14.36, 14.19, 12.48. **ESI-MS:** calculated $[C_{14}H_{20}O_3 + Na]^+$: 259.1305, found: 259.1312. $[\alpha]^{20}_D$ = -16.4 (c = 0.50, CH_2Cl_2).

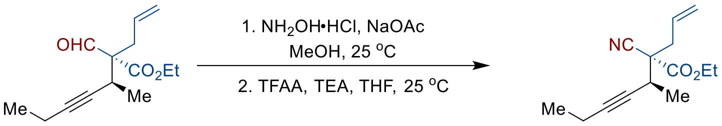

(*R*,*S*)-**18** (*R*,*S*)-**19**, 93% yield

To a solution of (*R,S*)-**18** (185.0 mg, 0.78 mmol) in MeOH (5 mL), NH_2OH•HCl (81.3 mg, 1.17 mmol, 1.5 equiv.) and sodium acetate (95.9 mg, 1.17 mmol, 1.5 equiv.) were added, and the resulting mixture was stirred at room temperature for 12 hours. Saturated $NaHCO_3$ aq. was then added, and the mixture was extracted with CH_2Cl_2 (three times). The organic layers were combined, dried over Na_2SO_4, and concentrated in vacuo. The crude product was then dissolved in anhydrous tetrahydrofuran (5 mL), and then triethylamine (789.3 mg, 7.8 mmol, 10.0 equiv.) and trifluoroacetic anhydride (819.1 mg, 3.9 mmol, 5.0 equiv.) were added to the stirred solution. The resulting mixture was stirred at room temperature for 12 hours. After completion of the reaction, the mixture was poured into water, neutralized with a 10% solution of $NaHCO_3$, and extracted with ethyl acetate. The combined organic layers were dried over Na_2SO_4, filtered, and concentrated in vacuo. The residue was purified by silica gel chromatography to afford the desired product (*R,S*)-**19** (170.0 mg, 93% yield).

Ethyl (2*R*,3*S*)-2-allyl-2-cyano-3-methylhept-4-ynoate ((*R*,*S*)-19)

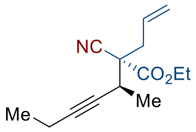

Colorless oil (93% yield, >20:1 dr). R_f = 0.45 (Hexane/EtOAc = 19/1). **^{1}H NMR (500 MHz, $CDCl_3$)** δ 5.85 – 5.74 (m, 1H), 5.29 – 5.19 (m, 2H), 4.33 – 4.21 (m, 2H), 3.03 – 2.93 (m, 1H), 2.67 – 2.54 (m, 2H), 2.16 (qd, *J* = 7.5, 2.2 Hz, 2H), 1.36 – 1.30 (m, 6H), 1.10 (t, *J* = 7.5 Hz, 3H). **^{13}C NMR (125 MHz, $CDCl_3$)** δ 167.53, 130.75, 121.03, 117.77, 86.55, 62.73, 55.12, 39.06, 33.19, 16.93, 14.28, 14.03, 12.45. **ESI-MS:** calculated $[C_{14}H_{19}NO_2 + Na]^+$: 256.1308, found: 256.1314. $[\alpha]^{20}_D$ = 6.8 (c = 0.50, CH_2Cl_2).

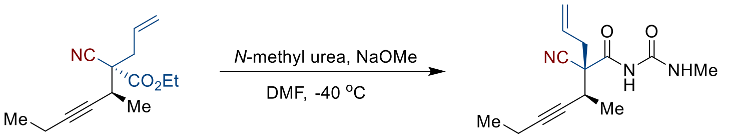

(*R*,*S*)-**19** (*R*,*S*)-**20**, 70% yield, >99% ee

To a solution of (*R,S*)-**19** (150.0 mg, 0.64 mmol) in DMF (5 mL), methylurea (94.8 mg, 1.28 mmol, 2.0 equiv.) and NaOMe (69.1 mg, 1.28 mmol, 2.0 equiv.) were added, and the resulting mixture was stirred at -40 °C for 12 hours. After completion, the reaction was quenched by the addition of water and extraction with ethyl acetate. The combined organic layers were dried over anhydrous Na_2SO_4 and concentrated under vacuum. The residue was purified by silica gel chromatography to afford the desired product (*R,S*)-**20** (117.7 mg, 70% yield).

(2*R*,3*S*)-2-allyl-2-cyano-3-methyl-*N*-(methylcarbamoyl)hept-4-ynamide ((*R*,*S*)-20)

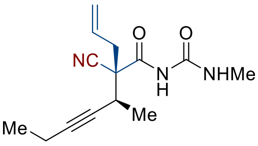

White solid (70% yield, >20:1 dr). R_f = 0.20 (Hexane/EtOAc = 4/1). **^{1}H NMR (500 MHz, $CDCl_3$)** δ 8.56 (s, 1H), 8.04 (s, 1H), 5.82 – 5.71 (m, 1H), 5.33 – 5.24 (m, 2H), 3.05 – 2.98 (m, 1H), 2.88 (d, J = 4.8 Hz, 3H), 2.69 (dd, J = 13.7, 7.4 Hz, 1H), 2.62 (dd, J = 13.7, 7.1 Hz, 1H), 2.20 (qd, J = 7.5, 2.2 Hz, 2H), 1.37 (d, J = 7.0 Hz, 3H), 1.12 (t, J = 7.5 Hz, 3H). **^{13}C NMR (125 MHz, $CDCl_3$)** δ 168.18, 152.64, 129.85, 121.99, 117.54, 88.57, 76.96, 57.06, 38.98, 32.91, 26.60, 17.17, 13.82, 12.42. **ESI-MS:** calculated $[C_{14}H_{19}N_3O_2 + Na]^+$: 284.1369, found: 284.1378. $[\alpha]^{20}_D$ = 36.2 (c = 1.05, CH_2Cl_2). The product was analyzed by HPLC to determine the enantiomeric excess: >99% ee (CHIRALPAK IA, hexane/*i*-PrOH = 98/2, detector: 211 nm, flow rate: 1.0 mL/min), t_1(minor) = 17.8 min, t_2(major) = 20.8 min.

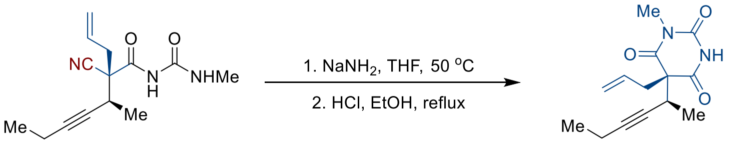

(*R*,*S*)-**20**, >99% ee

(*S*,*S*)-Methohexital
(*S*,*S*)-**21**, 68% yield, >99% ee

To a solution of (*R,S*)-**20** (30.0 mg, 0.115 mmol) in THF (1 mL) was added $NaNH_2$ (13.7 mg, 0.35 mmol, 3.0 equiv.), and the resulting mixture was stirred at 50 °C for 12 hours. The reaction was then cooled to room temperature, diluted with water, and extracted with ethyl acetate three times. The combined organic layers were concentrated under reduced pressure. The residue was dissolved in EtOH (1.5 mL), followed by the addition of a 6.0 M HCl solution (2.0 mL). The reaction mixture was stirred under reflux conditions for 3 hours. The reaction mixture was then cooled to room temperature, diluted with water, and extracted with ethyl acetate. The combined organic layers were dried over anhydrous Na_2SO_4 and concentrated under vacuum. The resulting residue was purified by silica gel chromatography to afford the desired product (*S,S*)-**21** (20.4 mg, 68% yield).

(*S*)-5-allyl-5-((*S*)-hex-3-yn-2-yl)-1-methylpyrimidine-2,4,6(1H,3H,5H)-trione ((*S*,*S*)-21)

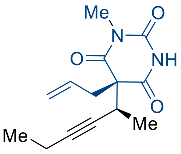

Colorless oil (68% yield, >20:1 dr). R_f = 0.30 (Hexane/EtOAc = 4/1). **^{1}H NMR (500 MHz, $CDCl_3$)** δ 5.60 – 5.48 (m, 1H), 5.16 – 4.98 (m, 2H), 3.27 (s, 3H), 3.12 – 3.02 (m, 1H), 2.85 (dd, J = 12.8, 7.5 Hz, 1H), 2.64 (dd, J = 12.9, 7.2 Hz, 1H), 2.08 (qd, J = 7.5, 2.2 Hz, 2H), 1.27 (d, J = 7.1 Hz, 3H), 1.02 (t, J = 7.5 Hz, 3H). **^{13}C NMR (125 MHz, $CDCl_3$)** δ 170.93, 170.00,

150.44, 131.25, 120.96, 86.51, 77.63, 59.75, 38.17, 35.74, 27.74, 16.13, 13.93, 12.31. **ESI-MS:** calculated $[C_{14}H_{18}N_2O_3 + H]^+$: 263.1390, found: 263.1398. $[\alpha]^{20}_D$ = 60.0 (c = 0.95, CH_2Cl_2). The product was analyzed by HPLC to determine the enantiomeric excess: >99% ee (CHIRALPAK IA, hexane/*i*-PrOH = 97/3, detector: 211 nm, flow rate: 1.0 mL/min), t_1(major) = 16.5 min, t_2(minor) = 18.2 min.

3.4.2 Total synthesis of (*R*,*R*)-Methohexital

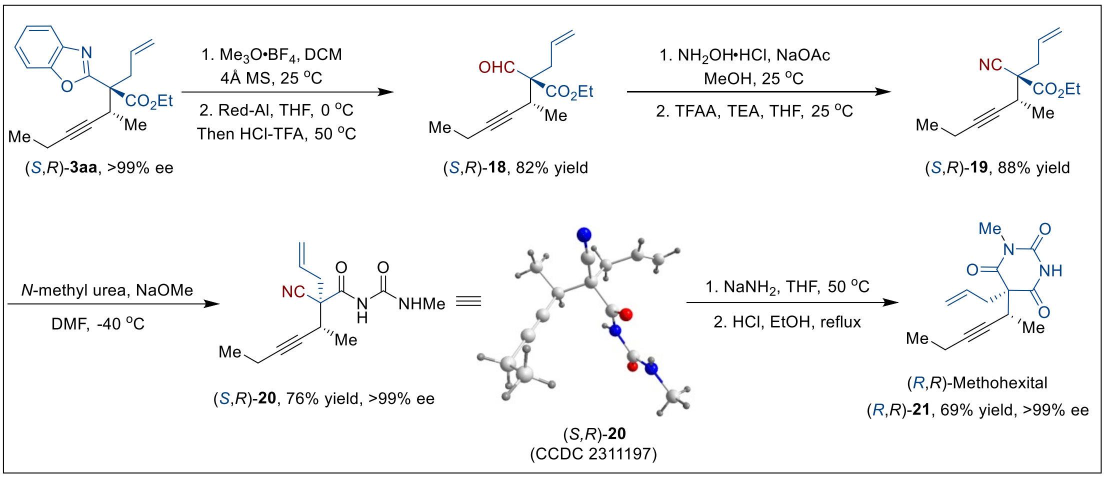

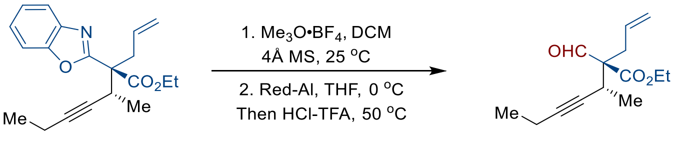

(*S*,*R*)-**3aa**, >99% ee (*S*,*R*)-**18**, 82% yield

To a solution of (*S,R*)-**3aa** (325.0 mg, 1.0 mmol) in dry DCM (10 mL) was added 4Å molecular sieves (700 mg), and the mixture was stirred vigorously for 10 minutes at 25 °C under an argon atmosphere. Trimethyloxonium tetrafluoroborate (1479 mg, 10 mmol, 10.0 equiv.) was then added, and the mixture was stirred for 48 hours. After complete consumption of the starting material (*S,R*)-**3aa**, the reaction mixture was concentrated by filtering off the molecular sieves to give the crude *N*-methylbenzoxazolium salt. The crude product was then dissolved in dry THF (6 mL) and cooled to 0 °C. Sodium bis(2-methoxyethoxy)aluminum hydride in toluene (Red-Al, 70% wt, 571 µL, 2.0 mmol, 2.0 equiv.) was added to the solution dropwise via syringe. The reaction mixture was stirred for 2 hours at 0 °C. Subsequently, 1 N aqueous hydrochloric acid solution (12 mL) and trifluoroacetic acid (766 µL, 10 mmol, 10.0 equiv.) were added sequentially. After stirring for 2 hours at 50 °C, the biphasic reaction mixture was cooled to 25 °C and extracted with ethyl acetate. The combined organic layers were dried over $MgSO_4$, filtered, and concentrated under vacuum. The residue was purified by silica gel chromatography to afford the desired product (*S,R*)-**18** (194.0 mg, 82% yield).

Ethyl (2*S*,3*R*)-2-allyl-2-formyl-3-methylhept-4-ynoate ((*S*,*R*)-18)

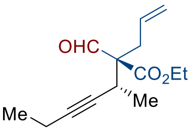

Colorless oil (82% yield, >20:1 dr). R_f = 0.50 (Hexane/EtOAc = 19/1). **^{1}H NMR (500 MHz, CDCl$_3$)** δ 9.91 (s, 1H), 5.75 – 5.64 (m, 1H), 5.13 – 5.01 (m, 2H), 4.24 (q, *J* = 7.1 Hz, 2H), 3.13 – 3.05 (m, 1H), 2.68 (dd, *J* = 14.0, 6.4 Hz, 1H), 2.39 (dd, *J* = 14.0, 8.2 Hz, 1H), 2.15 (qd, *J* = 7.5, 2.2 Hz, 2H), 1.29 (t, *J* = 7.1 Hz, 3H), 1.12 – 1.05 (m, 6H). **^{13}C NMR (125 MHz, CDCl$_3$)** δ 200.23, 170.76,

132.69, 119.00, 85.87, 79.02, 62.98, 61.40, 34.95, 30.14, 16.13, 14.36, 14.18, 12.48. **ESI-MS:** calculated $[C_{14}H_{20}O_3 + Na]^+$: 259.1305, found: 259.1305. $[\alpha]^{20}_D$ = 14.8 (c = 0.55, CH_2Cl_2).

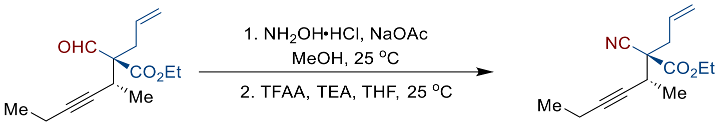

(*S*,*R*)-**18** (*S*,*R*)-**19**, 88% yield

To a solution of (*S,R*)-**18** (180.0 mg, 0.76 mmol) in MeOH (5 mL), NH_2OH•HCl (79.2 mg, 1.14 mmol, 1.5 equiv.) and sodium acetate (93.5 mg, 1.14 mmol, 1.5 equiv.) were added, and the resulting mixture was stirred at room temperature for 12 hours. Saturated $NaHCO_3$ aq. was added, and the mixture was extracted with CH_2Cl_2 (three times). The organic layers were combined, dried over Na_2SO_4, and concentrated in vacuo. The crude product was then dissolved in anhydrous tetrahydrofuran (5 mL), and then triethylamine (769.0 mg, 7.6 mmol, 10.0 equiv.) and trifluoroacetic anhydride (798.1 mg, 3.8 mmol, 5.0 equiv.) were added to the stirred solution. The resulting mixture was stirred at room temperature for 12 hours. After completion of the reaction, the mixture was poured into water, neutralized with a 10% solution of $NaHCO_3$, and extracted with ethyl acetate. The combined organic layers were dried over Na_2SO_4, filtered, and concentrated in vacuo. The residue was purified by silica gel chromatography to afford the desired product (*S,R*)-**19** (156.5 mg, 88% yield).

Ethyl (2*S*,3*R*)-2-allyl-2-cyano-3-methylhept-4-ynoate ((*S,R*)-19)

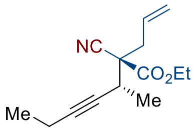

Colorless oil (88% yield, >20:1 dr). R_f = 0.45 (Hexane/EtOAc = 19/1). **^{1}H NMR (500 MHz, $CDCl_3$)** δ 5.85 – 5.73 (m, 1H), 5.28 – 5.18 (m, 2H), 4.32 – 4.20 (m, 2H), 3.02 – 2.93 (m, 1H), 2.65 – 2.55 (m, 2H), 2.15 (qd, *J* = 7.5, 2.2 Hz, 2H), 1.37 – 1.29 (m, 6H), 1.10 (t, *J* = 7.5 Hz, 3H). **^{13}C NMR (125 MHz, $CDCl_3$)** δ 167.52, 130.74, 121.03, 117.76, 86.54, 62.72, 55.11, 39.05, 33.18, 16.92, 14.28, 14.03, 12.44. **ESI-MS:** calculated $[C_{14}H_{19}NO_2 + Na]^+$: 256.1308, found: 256.1310. $[\alpha]^{20}_D$ = -7.8 (c = 1.00, CH_2Cl_2).

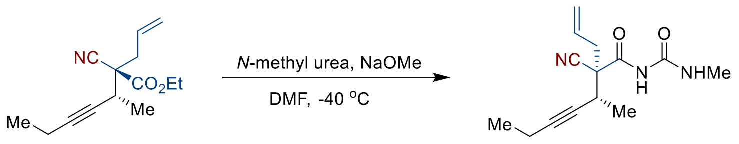

(*S*,*R*)-**19** (*S*,*R*)-**20**, 76% yield, >99% ee

To a solution of (*S,R*)-**19** (75.0 mg, 0.32 mmol) in DMF (3 mL), methylurea (47.4 mg, 0.64 mmol, 2.0 equiv.) and NaOMe (34.6 mg, 0.64 mmol, 2.0 equiv.) were added, and the resulting mixture was stirred at -40 °C for 12 hours. After completion, the reaction was quenched by the addition of water and extraction with ethyl acetate. The combined organic layers were dried over anhydrous Na_2SO_4 and concentrated under vacuum. The residue was purified by silica gel chromatography to afford the desired product (*S,R*)-**20** (63.7 mg, 76% yield).

(2*S*,3*R*)-2-allyl-2-cyano-3-methyl-*N*-(methylcarbamoyl)hept-4-ynamide ((*S*,*R*)-20)

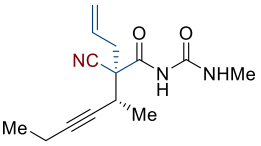

White solid (76% yield, >20:1 dr). R_f = 0.20 (Hexane/EtOAc = 4/1). **^{1}H NMR (500 MHz, $CDCl_3$)** δ 8.60 (s, 1H), 8.04 (s, 1H), 5.83 – 5.70 (m, 1H), 5.32 – 5.22 (m, 2H), 3.07 – 2.98 (m, 1H), 2.88 (d, *J* = 4.8 Hz, 3H), 2.69 (dd, *J* = 13.7, 7.4 Hz, 1H), 2.62 (dd, *J* = 13.7, 7.1 Hz, 1H), 2.19 (qd, *J* = 7.4, 2.0 Hz, 2H), 1.37 (d, *J* = 7.0 Hz, 3H), 1.11 (t, *J* = 7.5 Hz, 3H). **^{13}C NMR (125 MHz, $CDCl_3$)** δ 168.17, 152.65, 129.85, 121.95, 117.52, 88.52, 76.96, 57.06, 38.95, 32.89, 26.59, 17.16, 13.81, 12.41. **ESI-MS:** calculated $[C_{14}H_{19}N_3O_2 + Na]^+$: 284.1369, found: 284.1377. $[\alpha]^{20}_D$ = -35.1 (c = 0.50, CH_2Cl_2). The product was analyzed by HPLC to determine the enantiomeric excess: >99% ee (CHIRALPAK IA, hexane/*i*-PrOH = 98/2, detector: 211 nm, flow rate: 1.0 mL/min), t_1(major) = 18.0 min, t_2(minor) = 20.9 min.

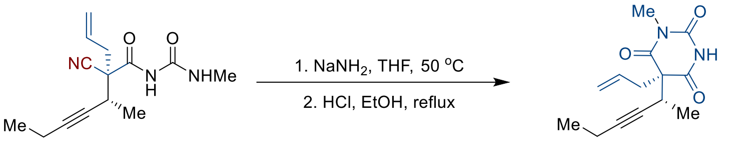

(*S*,*R*)-**20**, >99% ee

(*R*,*R*)-Methohexital
(*R*,*R*)-**21**, 69% yield, >99% ee

To a solution of (*S,R*)-**20** (27.0 mg, 0.10 mmol) in THF (1 mL) was added $NaNH_2$ (11.7 mg, 0.3 mmol, 3.0 equiv.), and the resulting mixture was stirred at 50 °C for 12 hours. The reaction was then cooled to room temperature, diluted with water, and extracted with ethyl acetate three times. The combined organic layers were concentrated under reduced pressure. The residue was dissolved in EtOH (1.5 mL), followed by the addition of a 6.0 M HCl solution (2.0 mL). The reaction mixture was stirred under reflux conditions for 3 hours. The reaction mixture was then cooled to room temperature, diluted with water, and extracted with ethyl acetate. The combined organic layers were dried over anhydrous Na_2SO_4 and concentrated under vacuum. The resulting residue was purified by silica gel chromatography to afford the desired product (*R,R*)-**21** (18.6 mg, 69% yield).

(*R*)-5-allyl-5-((*R*)-hex-3-yn-2-yl)-1-methylpyrimidine-2,4,6(1H,3H,5H)-trione ((*R,R*)-21)

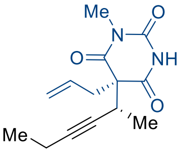

Colorless oil (69% yield, >20:1 dr). R_f = 0.30 (Hexane/EtOAc = 4/1). **^{1}H NMR (500 MHz, $CDCl_3$)** δ 5.60 – 5.48 (m, 1H), 5.19 – 5.03 (m, 2H), 3.28 (s, 3H), 3.13 – 3.03 (m, 1H), 2.85 (dd, *J* = 12.9, 7.5 Hz, 1H), 2.64 (dd, *J* = 12.9, 7.2 Hz, 1H), 2.09 (qd, *J* = 7.5, 2.3 Hz, 2H), 1.28 (d, *J* = 7.1 Hz, 3H), 1.02 (t, *J* = 7.5 Hz, 3H). **^{13}C NMR (125 MHz, $CDCl_3$)** δ 170.86, 170.00, 150.38, 131.25, 120.98, 86.53, 77.62, 59.76, 38.18, 35.75, 27.75, 16.13, 13.94, 12.32. **ESI-MS:** calculated $[C_{14}H_{18}N_2O_3 + H]^+$: 263.1390, found: 263.1399. $[\alpha]^{20}_D$ = -69.6 (c = 0.85, CH_2Cl_2). The product was analyzed by HPLC to determine the enantiomeric excess: >99% ee (CHIRALPAK IA, hexane/*i*-PrOH = 97/3, detector: 211 nm, flow rate: 1.0 mL/min), t_1(minor) = 16.6 min, t_2(major) = 18.2 min.

3.4.3 Total synthesis of (*S,R*)-Methohexital

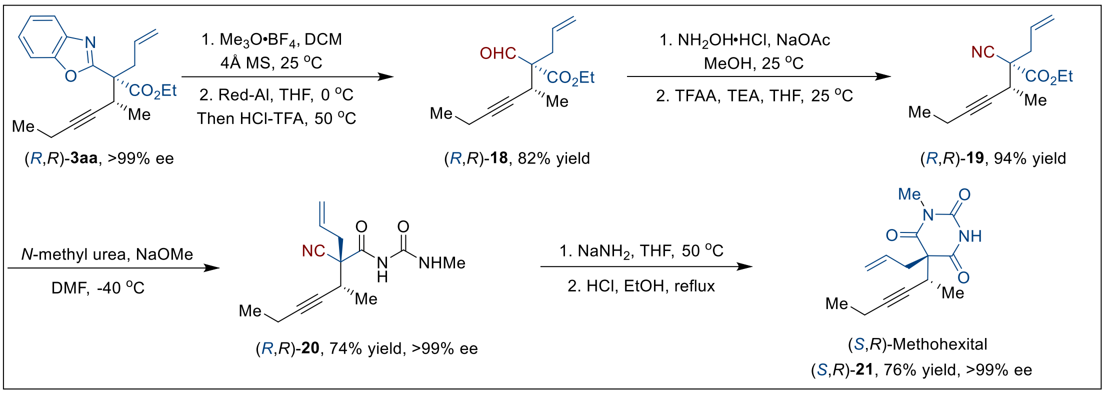

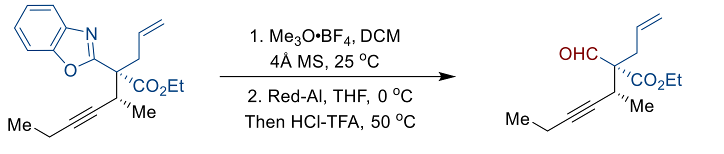

(*R*,*R*)-**3aa**, >99% ee (*R*,*R*)-**18**, 82% yield

To a solution of (*R,R*)-**3aa** (390.5 mg, 1.2 mmol) in dry DCM (12 mL) was added 4Å molecular sieves (800 mg), and the mixture was stirred vigorously for 10 minutes at 25 °C under an argon atmosphere. Trimethyloxonium tetrafluoroborate (1775 mg, 12 mmol, 10.0 equiv.) was then added, and the mixture was stirred for 48 hours. After complete consumption of the starting material (*R,R*)-**3aa**, the reaction mixture was concentrated by filtering off the molecular sieves to give the crude *N*-methylbenzoxazolium salt. The crude product was then dissolved in dry THF (8 mL) and cooled to 0 °C. Sodium bis(2-methoxyethoxy)aluminum hydride in toluene (Red-Al, 70% wt, 686 µL, 2.4 mmol, 2.0 equiv.) was added to the solution dropwise via syringe. The reaction mixture was stirred for 2 hours at 0 °C. Subsequently, 1 N aqueous hydrochloric acid solution (16 mL) and trifluoroacetic acid (919 µL, 12 mmol, 10.0 equiv.) were added sequentially. After stirring for 2 hours at 50 °C, the biphasic reaction mixture was cooled to 25 °C and extracted with ethyl acetate. The combined organic layers were dried over $MgSO_4$, filtered, and concentrated under vacuum. The residue was purified by silica gel chromatography to afford the desired product (*R,R*)-**18** (233.3 mg, 82% yield).

Ethyl (2*R*,3*R*)-2-allyl-2-formyl-3-methylhept-4-ynoate ((*R*,*R*)-18)

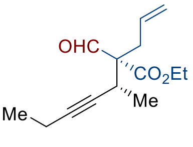

Colorless oil (82% yield, 20:1 dr). R_f = 0.50 (Hexane/EtOAc = 19/1). **^{1}H NMR (500 MHz, CDCl$_3$)** δ 9.87 (s, 1H), 5.76 – 5.66 (m, 1H), 5.13 – 5.02 (m, 2H), 4.22 (q, *J* = 7.1 Hz, 2H), 3.07 – 2.95 (m, 1H), 2.76 (dd, *J* = 13.9, 6.6 Hz, 1H), 2.53 (dd, *J* = 13.9, 8.1 Hz, 1H), 2.13 (qd, *J* = 7.5, 2.3 Hz, 2H), 1.32 – 1.25 (m, 6H), 1.08 (t, *J* = 7.5 Hz, 3H). **^{13}C NMR (125 MHz, CDCl$_3$)** δ 199.05, 170.57, 132.72, 119.06, 86.14, 78.20, 62.96, 61.48, 36.45, 31.06, 17.53, 14.35, 14.19, 12.47. **ESI-MS:** calculated $[C_{14}H_{20}O_3 + Na]^+$: 259.1305, found: 259.1312. $[\alpha]^{20}_D$ = -21.1 (c = 0.50, CH_2Cl_2).

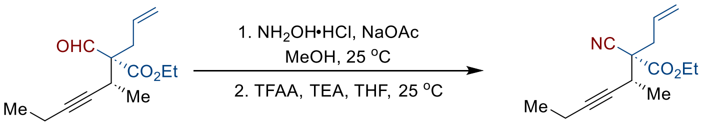

(*R*,*R*)-**18** (*R*,*R*)-**19**, 94% yield

To a solution of (*R,R*)-**18** (110.0 mg, 0.465 mmol) in MeOH (3 mL), NH_2OH•HCl (48.6 mg, 0.70 mmol, 1.5 equiv.) and sodium acetate (57.4 mg, 0.70 mmol, 1.5 equiv.) were added, and the resulting mixture was stirred at room temperature for 12 hours. Saturated $NaHCO_3$ aq. was added, and the mixture was extracted with CH_2Cl_2 (three times). The organic layers were combined, dried over Na_2SO_4, and concentrated in vacuo. The crude product was then dissolved in anhydrous tetrahydrofuran (3 mL), and then triethylamine (475.6 mg, 4.7 mmol, 10.0 equiv.) and trifluoroacetic anhydride (483.1 mg, 2.3 mmol, 5.0 equiv.) were added to the stirred solution. The resulting mixture was stirred at room temperature for 12 hours. After completion of the reaction, the mixture was poured into water, neutralized with a 10% solution of $NaHCO_3$, and extracted with ethyl acetate. The combined organic layers were dried over Na_2SO_4, filtered, and concentrated in vacuo. The residue was purified by silica gel chromatography to afford the desired product (*R,R*)-**19** (102.0 mg, 94% yield).

Ethyl (2*R*,3*R*)-2-allyl-2-cyano-3-methylhept-4-ynoate ((*R*,*R*)-19)

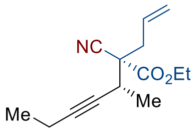

Colorless oil (94% yield, 20:1 dr). R_f = 0.45 (Hexane/EtOAc = 19/1). **^{1}H NMR (500 MHz, $CDCl_3$)** δ 5.89 – 5.71 (m, 1H), 5.29 – 5.12 (m, 2H), 4.25 (q, *J* = 7.1 Hz, 2H), 3.03 – 2.88 (m, 2H), 2.62 (dd, *J* = 13.8, 8.2 Hz, 1H), 2.20 (qd, *J* = 7.5, 2.2 Hz, 2H), 1.33 – 1.25 (m, 6H), 1.14 (t, *J* = 7.5 Hz, 3H). **^{13}C NMR (125 MHz, $CDCl_3$)** δ 167.83, 130.98, 120.66, 117.38, 87.18, 77.04, 62.92, 55.35, 41.19, 33.60, 18.27, 14.27, 14.09, 12.50. **ESI-MS:** calculated $[C_{14}H_{19}NO_2 + Na]^+$: 256.1308, found: 256.1309. $[\alpha]^{20}_D$ = -72.8 (c = 0.50, CH_2Cl_2).

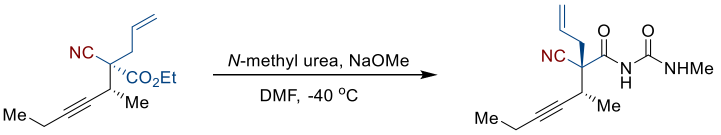

(*R*,*R*)-**19** (*R*,*R*)-**20**, 74% yield, >99% ee

To a solution of (*R,R*)-**19** (84.0 mg, 0.36 mmol) in DMF (3 mL), methylurea (53.3 mg, 0.72 mmol, 2.0 equiv.) and NaOMe (38.9 mg, 0.72 mmol, 2.0 equiv.) were added, and the resulting mixture was stirred at -40 °C for 12 hours. After completion, the reaction was quenched by the addition of water and extraction with ethyl acetate. The combined organic layers were dried over anhydrous Na_2SO_4 and concentrated under vacuum. The residue was purified by silica gel chromatography to afford the desired product (*R,R*)-**20** (69.7 mg, 74% yield).

(2*R*,3*R*)-2-allyl-2-cyano-3-methyl-*N*-(methylcarbamoyl)hept-4-ynamide ((*R*,*R*)-20)

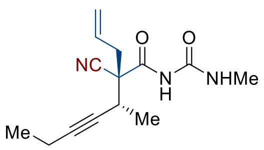

White solid (74% yield, 20:1 dr). R_f = 0.20 (Hexane/EtOAc = 4/1). **^{1}H NMR (500 MHz, $CDCl_3$)** δ 8.80 (s, 1H), 8.11 – 7.98 (m, 1H), 5.83 – 5.72 (m, 1H), 5.28 – 5.21 (m, 2H), 3.10 – 3.00 (m, 1H), 2.95 – 2.85 (m, 4H), 2.71 (dd, *J* = 14.0, 8.1 Hz, 1H), 2.20 (qd, *J* = 7.5, 2.2 Hz, 2H), 1.27 (d, *J* = 6.9 Hz, 3H), 1.13 (t, *J* = 7.5 Hz, 3H). **^{13}C NMR (125 MHz, $CDCl_3$)** δ 168.16, 152.74, 130.24, 121.62, 117.44, 87.70, 76.87, 57.03, 40.32, 33.21, 26.61, 18.06, 13.99, 12.46. **ESI-MS:** calculated $[C_{14}H_{19}N_3O_2 + Na]^+$: 284.1369, found: 284.1373. $[\alpha]^{20}_D$ = -57.6 (c = 0.50, CH_2Cl_2). The product was analyzed by HPLC to determine the enantiomeric excess: >99% ee (CHIRALPAK IA, hexane/*i*-PrOH = 98/2, detector: 211 nm, flow rate: 1.0 mL/min), t_1(minor) = 14.9 min, t_2(major) = 16.1 min.

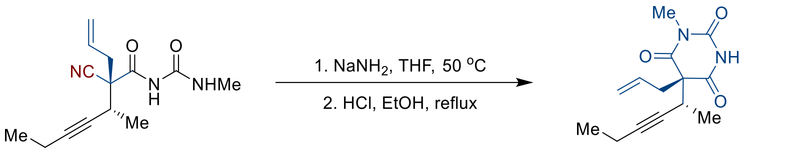

(*R*,*R*)-**20**, >99% ee

(*S*,*R*)-Methohexital
(*S*,*R*)-**21**, 76% yield, >99% ee

To a solution of (*R*,*R*)-**20** (30.0 mg, 0.115 mmol) in THF (1 mL) was added $NaNH_2$ (13.7 mg, 0.35 mmol, 3.0 equiv.), and the resulting mixture was stirred at 50 °C for 12 hours. The reaction was then cooled to room temperature, diluted with water, and extracted with ethyl acetate three times. The combined organic layers were concentrated under reduced pressure. The residue was dissolved in EtOH (1.5 mL), followed by the addition of a 6.0 M HCl solution (2.0 mL). The reaction mixture was stirred under reflux conditions for 3 hours. The reaction mixture was then cooled to room temperature, diluted with water, and extracted with ethyl acetate. The combined organic layers were dried over anhydrous Na_2SO_4 and concentrated under vacuum. The resulting residue was purified by silica gel chromatography to afford the desired product (*S*,*R*)-**21** (22.9 mg, 76% yield).

(*S*)-5-allyl-5-((*R*)-hex-3-yn-2-yl)-1-methylpyrimidine-2,4,6(1H,3H,5H)-trione ((*S*,*R*)-21)

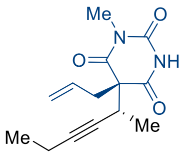

White solid (76% yield, 20:1 dr). R_f = 0.30 (Hexane/EtOAc = 4/1). **^{1}H NMR (500 MHz, CDCl$_3$)** δ 5.59 – 5.47 (m, 1H), 5.18 – 5.04 (m, 2H), 3.28 (s, 3H), 3.12 – 3.02 (m, 1H), 2.85 (dd, *J* = 12.9, 7.4 Hz, 1H), 2.65 (dd, *J* = 12.9, 7.3 Hz, 1H), 2.08 (qd, *J* = 7.5, 2.3 Hz, 2H), 1.28 (d, *J* = 7.1 Hz, 3H), 1.02 (t, *J* = 7.5 Hz, 3H). **^{13}C NMR (125 MHz, CDCl$_3$)** δ 171.79, 169.04, 150.43, 131.26, 120.97, 86.53, 77.60, 59.88, 38.14, 36.12, 27.85, 16.14, 13.98, 12.30. **ESI-MS:** calculated $[C_{14}H_{18}N_2O_3 + H]^+$: 263.1390, found: 263.1391. $[\alpha]^{20}_D$ = -31.5 (c = 1.10, CH_2Cl_2). The product was analyzed by HPLC to determine the enantiomeric excess: >99% ee (CHIRALPAK IA, hexane/*i*-PrOH = 97/3, detector: 211 nm, flow rate: 1.0 mL/min), t_1(minor) = 14.6 min, t_2(major) = 15.5 min.

3.4.4 Total synthesis of (*R*,*S*)-Methohexital

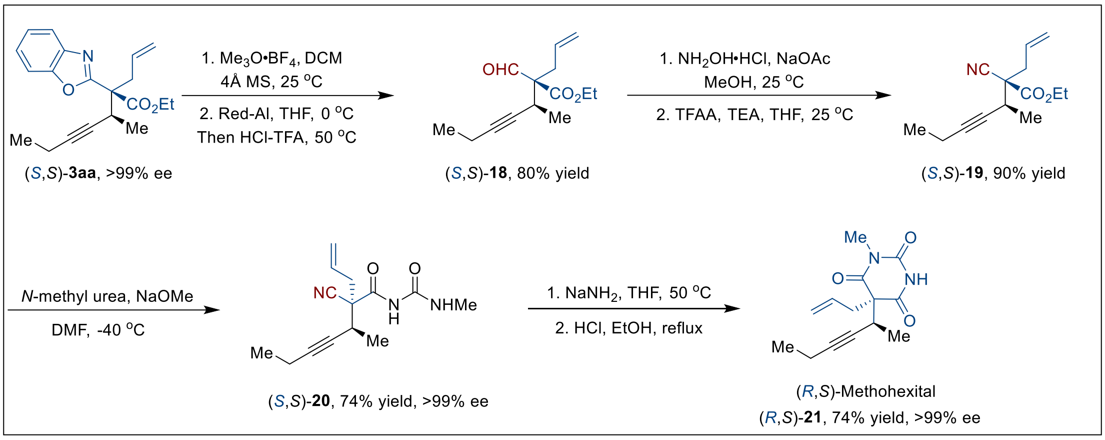

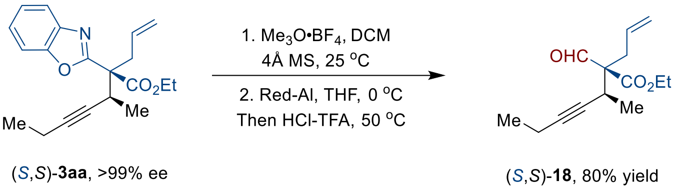

To a solution of (*S*,*S*)-**3aa** (390.5 mg, 1.2 mmol) in dry DCM (12 mL) was added 4Å molecular sieves (800 mg), and the mixture was stirred vigorously for 10 minutes at 25 °C under an argon atmosphere. Trimethyloxonium tetrafluoroborate (1775 mg, 12 mmol, 10.0 equiv.) was then added, and the mixture was stirred for 48 hours. After complete consumption of the starting material (*S*,*S*)-**3aa**, the reaction mixture was concentrated by filtering off the molecular sieves to give the crude *N*-methylbenzoxazolium salt. The crude product was then dissolved in dry THF (8 mL) and cooled to 0 °C. Sodium bis(2-methoxyethoxy)aluminum hydride in toluene (Red-Al, 70% wt, 686 μL, 2.4 mmol, 2.0 equiv.) was added to the solution dropwise via syringe. The reaction mixture was stirred for 2 hours at 0 °C. Subsequently, 1 N aqueous hydrochloric acid solution (16 mL) and trifluoroacetic acid (919 μL, 12 mmol, 10.0 equiv.) were added sequentially. After stirring for 2 hours at 50 °C, the biphasic reaction mixture was cooled to 25 °C and extracted with ethyl acetate. The combined organic layers were dried over $MgSO_4$, filtered, and concentrated under vacuum. The residue was purified by silica gel chromatography to afford the desired product (*S*,*S*)-**18** (227.3 mg, 80% yield).

Ethyl (2*S*,3*S*)-2-allyl-2-formyl-3-methylhept-4-ynoate ((*S*,*S*)-18)

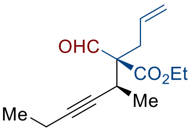

Colorless oil (80% yield, 20:1 dr). R_f = 0.50 (Hexane/EtOAc = 19/1). **^{1}H NMR (500 MHz, $CDCl_3$)** δ 9.87 (s, 1H), 5.82 – 5.62 (m, 1H), 5.19 – 4.99 (m, 2H), 4.22 (q, *J* = 7.1 Hz, 2H), 3.08 – 2.95 (m, 1H), 2.76 (dd, *J* = 13.9, 6.6 Hz, 1H), 2.53 (dd, *J* = 13.9, 8.1 Hz, 1H), 2.13 (qd, *J* = 7.5, 2.3 Hz, 2H), 1.34 – 1.25 (m, 6H), 1.08 (t, *J* = 7.5 Hz, 3H). **^{13}C NMR (125 MHz, $CDCl_3$)** δ 199.05, 170.57, 132.72, 119.06, 86.14, 78.20, 62.97, 61.48, 36.45, 31.06, 17.53, 14.35, 14.19, 12.47. **ESI-MS:** calculated $[C_{14}H_{20}O_3 + Na]^+$: 259.1305, found: 259.1306. $[\alpha]^{20}_D$ = 23.2 (c = 0.50, CH_2Cl_2).

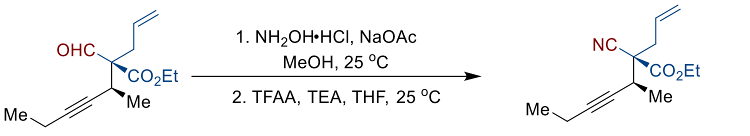

(S,S)-**18** (S,S)-**19**, 90% yield, >99% e.e.

To a solution of (S,S)-**18** (105.0 mg, 0.444 mmol) in MeOH (3 mL), $NH_2OH{\bullet}HCl$ (46.6 mg, 0.67 mmol, 1.5 equiv.) and sodium acetate (54.9 mg, 0.67 mmol, 1.5 equiv.) were added, and the resulting mixture was stirred at room temperature for 12 hours. Saturated $NaHCO_3$ aq. was added, and the mixture was extracted with CH_2Cl_2 (three times). The organic layers were combined, dried over Na_2SO_4, and concentrated in vacuo. The crude product was then dissolved in anhydrous tetrahydrofuran (3 mL), and then triethylamine (445.2 mg, 4.4 mmol, 10.0 equiv.) and trifluoroacetic anhydride (462.1 mg, 2.2 mmol, 5.0 equiv.) were added to the stirred solution. The resulting mixture was stirred at room temperature for 12 hours. After completion of the reaction, the mixture was poured into water, neutralized with a 10% solution of $NaHCO_3$, and extracted with ethyl acetate. The combined organic layers were dried over Na_2SO_4, filtered, and concentrated in vacuo. The residue was purified by silica gel chromatography to afford the desired product (S,S)-**19** (93.0 mg, 90% yield).

Ethyl (2*S*,3*S*)-2-allyl-2-cyano-3-methylhept-4-ynoate ((*S,S*)-19)

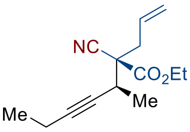

Colorless oil (90% yield, 20:1 dr). R_f = 0.45 (Hexane/EtOAc = 19/1). **^{1}H NMR (500 MHz, $CDCl_3$)** δ 5.87 – 5.76 (m, 1H), 5.26 – 5.17 (m, 2H), 4.25 (q, J = 7.1 Hz, 2H), 3.00 – 2.90 (m, 2H), 2.63 (dd, J = 13.8, 8.3 Hz, 1H), 2.21 (qd, J = 7.5, 2.2 Hz, 2H), 1.33 – 1.27 (m, 6H), 1.14 (t, J = 7.5 Hz, 3H). **^{13}C NMR (125 MHz, $CDCl_3$)** δ 167.84, 130.99, 120.67, 117.39, 87.19, 77.05, 62.93, 55.36, 41.20, 33.61, 18.28, 14.28, 14.10, 12.51. **ESI-MS:** calculated $[C_{14}H_{19}NO_2 + Na]^+$: 256.1308, found: 256.1313. $[\alpha]^{20}_D$ = 76.0 (c = 0.50, CH_2Cl_2).

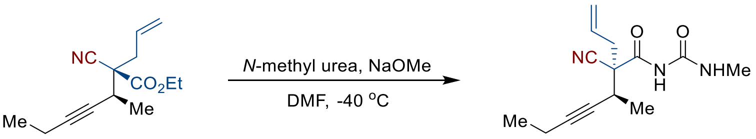

(S,S)-**19** (S,S)-**20**, 74% yield, >99% ee

To a solution of (S,S)-**19** (75.0 mg, 0.32 mmol) in DMF (3 mL), methylurea (47.4 mg, 0.64 mmol, 2.0 equiv.) and NaOMe (34.6 mg, 0.64 mmol, 2.0 equiv.) were added, and the resulting mixture was stirred at -40 °C for 12 hours. After completion, the reaction was quenched by the addition of water and extraction with ethyl acetate. The combined organic layers were dried over anhydrous Na_2SO_4 and concentrated under vacuum. The residue was purified by silica gel chromatography to afford the desired product (S,S)-**20** (62.3 mg, 74% yield).

(2*S*,3*S*)-2-allyl-2-cyano-3-methyl-*N*-(methylcarbamoyl)hept-4-ynamide ((*S,S*)-20)

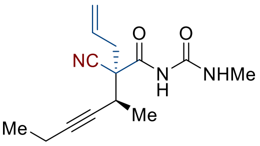

White solid (74% yield, 20:1 dr). R_f = 0.20 (Hexane/EtOAc = 4/1). **^{1}H NMR (500 MHz, $CDCl_3$)** δ 8.71 (s, 1H), 8.13 – 7.97 (m, 1H), 5.84 – 5.70 (m, 1H), 5.34 – 5.20 (m, 2H), 3.08 – 3.00 (m, 1H), 2.95 – 2.86 (m, 4H), 2.70 (dd, J = 13.9, 8.0 Hz, 1H), 2.21 (qd, J = 7.5, 2.1 Hz, 2H), 1.27 (d, J = 6.9 Hz, 3H), 1.14 (t, J = 7.5 Hz, 3H). **^{13}C NMR (125 MHz, $CDCl_3$)** δ 168.13, 152.65, 130.23, 121.67, 117.48, 87.74, 76.88, 56.98, 40.37, 33.26, 26.62, 18.06, 14.00, 12.48.

ESI-MS: calculated [$C_{14}H_{19}N_3O_2$ + Na]$^+$: 284.1369, found: 284.1374. $[\alpha]^{20}_D$ = 59.5 (c = 0.50, CH_2Cl_2). The product was analyzed by HPLC to determine the enantiomeric excess: >99% ee (CHIRALPAK IA, hexane/*i*-PrOH = 98/2, detector: 211 nm, flow rate: 1.0 mL/min), t_1(major) = 14.8 min, t_2(minor) = 16.2 min.

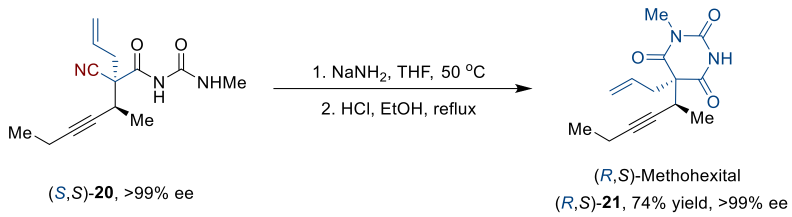

To a solution of (*S,S*)-**20** (30.0 mg, 0.115 mmol) in THF (1 mL) was added $NaNH_2$ (13.7 mg, 0.35 mmol, 3.0 equiv.), and the resulting mixture was stirred at 50 °C for 12 hours. The reaction was then cooled to room temperature, diluted with water, and extracted with ethyl acetate three times. The combined organic layers were concentrated under reduced pressure. The residue was dissolved in EtOH (1.5 mL), followed by the addition of a 6.0 M HCl solution (2.0 mL). The reaction mixture was stirred under reflux conditions for 3 hours. The reaction mixture was then cooled to room temperature, diluted with water, and extracted with ethyl acetate. The combined organic layers were dried over anhydrous Na_2SO_4 and concentrated under vacuum. The resulting residue was purified by silica gel chromatography to afford the desired product (*R,S*)-**21** (22.3 mg, 74% yield).

(*R*)-5-allyl-5-((*S*)-hex-3-yn-2-yl)-1-methylpyrimidine-2,4,6(1H,3H,5H)-trione ((*R,S*)-21)

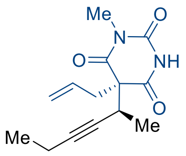

White solid (74% yield, 20:1 dr). R_f = 0.30 (Hexane/EtOAc = 4/1). **^{1}H NMR (500 MHz, CDCl$_3$)** δ 5.59 – 5.47 (m, 1H), 5.18 – 5.03 (m, 2H), 3.28 (s, 3H), 3.11 – 3.03 (m, 1H), 2.84 (dd, *J* = 12.9, 7.4 Hz, 1H), 2.64 (dd, *J* = 12.9, 7.3 Hz, 1H), 2.13 – 2.03 (m, 2H), 1.28 (d, *J* = 7.1 Hz, 3H), 1.07 – 0.98 (m, 3H). **^{13}C NMR (125 MHz, CDCl$_3$)** δ 171.79, 169.12, 150.51, 131.26, 120.95, 86.53, 77.60, 59.87, 38.13, 36.12, 27.84, 16.13, 13.97, 12.30. **ESI-MS:** calculated [$C_{14}H_{18}N_2O_3$ + H]$^+$: 263.1390, found: 263.1389. $[\alpha]^{20}_D$ = 31.5 (c = 1.20, CH_2Cl_2). The product was analyzed by HPLC to determine the enantiomeric excess: >99% ee (CHIRALPAK IA, hexane/*i*-PrOH = 97/3, detector: 211 nm, flow rate: 1.0 mL/min), t_1(major) = 14.5 min, t_2(minor) = 15.6 min.

4 Mechanistic studies

4.1 Kinetic studies

To better understand the underlying mechanism of this process, we conducted a kinetic study using gas chromatography-mass spectrometry (GC-MS). By studying the effect of copper catalyst addition on the reaction rate, we further demonstrated that Ni/Cu bimetallic synergistic catalysis is significantly better than single metal nickel catalysis at improving the reaction rate and final yield, thereby improving stereoselectivity.

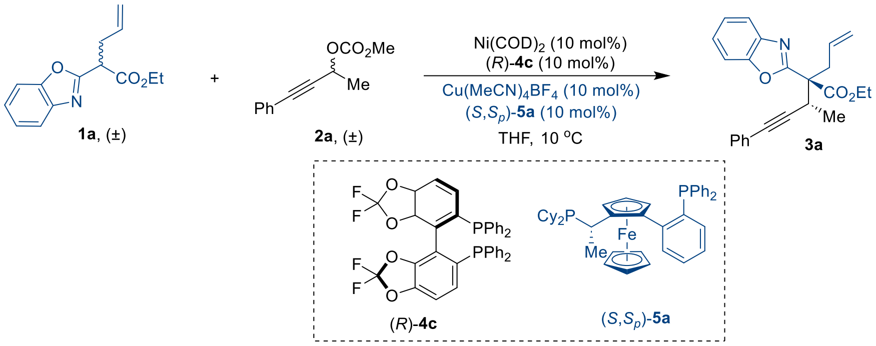

Time (h)	Standard condition (Yield of **3a**)	Time (h)	Without copper catalyst (Yield of **3a**)
0.5	11.6%	2	0.9%
1	22.7%	4	2.8%
1.5	32.5%	6	5.4%
2	45.2%	8	7.4%
2.5	52.5%	10	9.0%
3	65.5%		

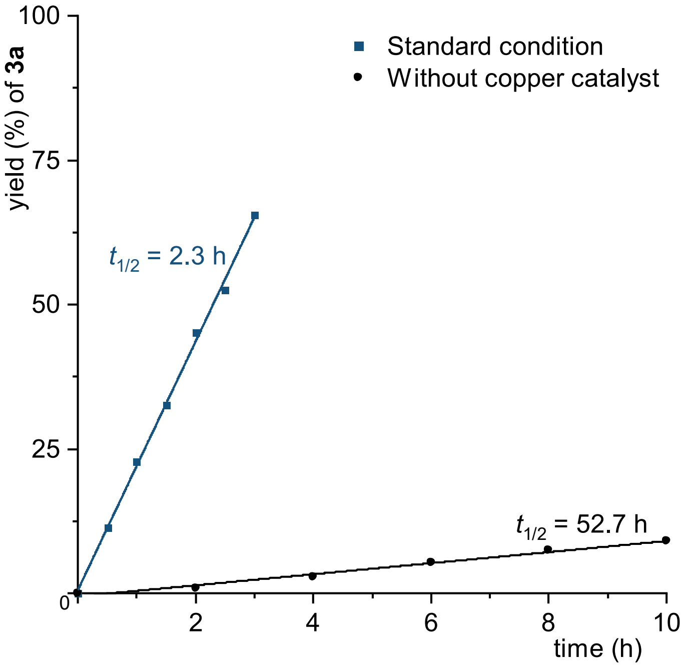

Figure S1. Kinetic studies

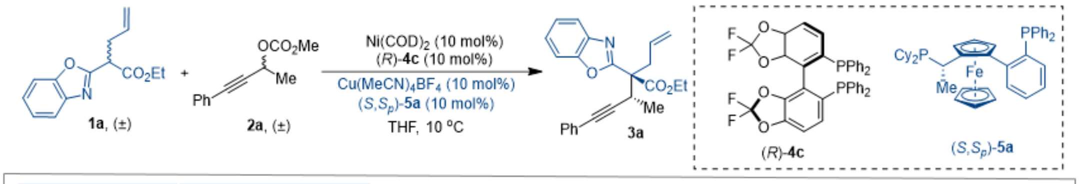

time (h)	yield of 3a
0.5	11.6%
1	22.7%
1.5	32.5%
2	45.2%
2.5	52.5%
3	65.5%
6	72%
12	76%
18	80%
24	83%
36	87%
48	92%
60	95%
72	95%

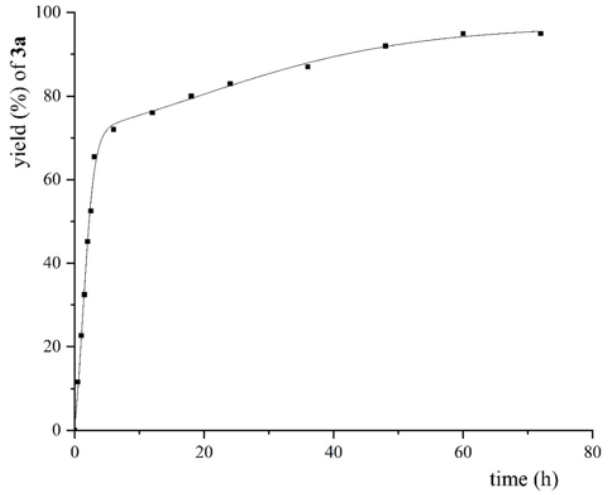

Figure S2. Comprehensive kinetic profile spanning from 0 to 72 h

4.2 Kinetic resolution studies

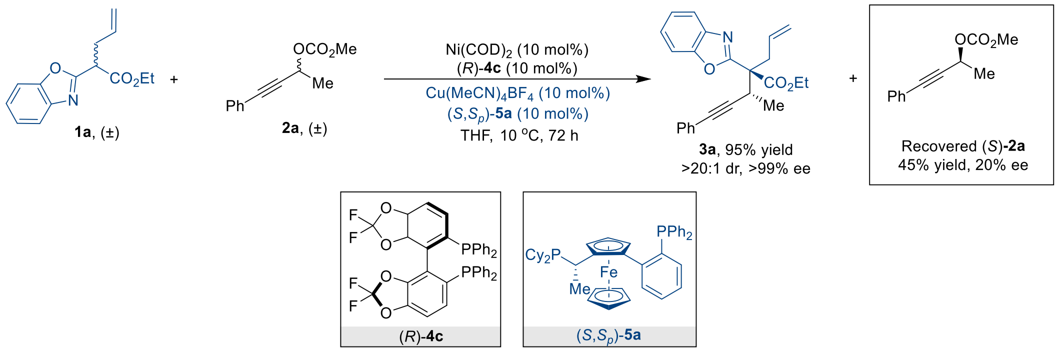

Procedure: In a nitrogen-filled glove box, an oven-dried 10 mL screw-cap reaction tube equipped with a stir bar was charged with Ni(COD)$_2$ (4.1 mg, 0.015 mmol, 10 mol%) and (*R*)-**4c** (10.2 mg, 0.015 mmol, 10 mol%) in THF (1 mL) at rt for about 20 min; Meanwhile, Cu(MeCN)$_4$BF$_4$ (4.7 mg, 0.015 mmol, 10 mol%) and (*S,S$_p$*)-**5a** (10.1 mg, 0.015 mmol, 10 mol%) were stirred in THF (1 mL) in a Schlenk flask under a nitrogen atmosphere at rt for 30 min. Benzoxazole ester **1a** (0.15 mmol, 1.0 equiv.) was added to the Schlenk flask containing copper complex and stirred for approximately 5 min. Propargylic carbonate **2a** (0.3 mmol, 2.0 equiv.) was then transformed into a Screw-cap reaction tube containing a nickel complex and stirred for an additional 5 min. The nickel complex solution was then combined with the copper complex solution, and the resulting solution was stirred for approximately 72 hours at 10 °C until substrate **1a** was completely consumed (monitored by TLC).

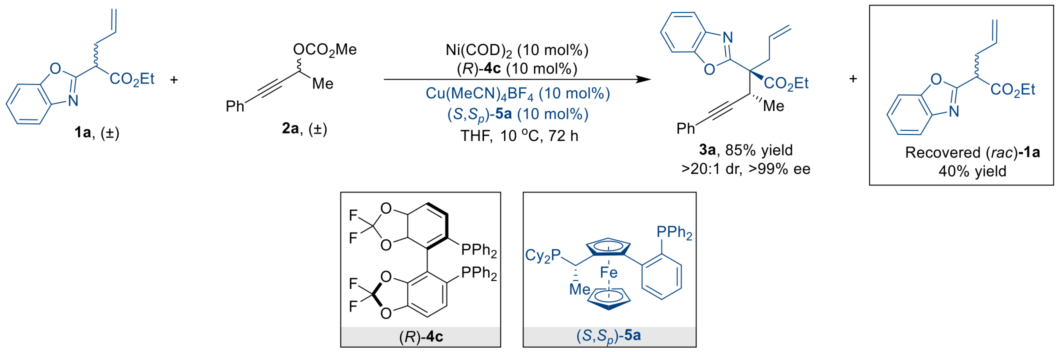

Procedure: In a nitrogen-filled glove box, an oven-dried 10 mL screw-cap reaction tube equipped with a stir bar was charged with $Ni(COD)_2$ (4.1 mg, 0.015 mmol, 10 mol%) and (*R*)-**4c** (10.2 mg, 0.015 mmol, 10 mol%) in THF (1 mL) at rt for about 20 min; Meanwhile, $Cu(MeCN)_4BF_4$ (4.7 mg, 0.015 mmol, 10 mol%) and (*S,S$_p$*)-**5a** (10.1 mg, 0.015 mmol, 10 mol%) were stirred in THF (1 mL) in a Schlenk flask under a nitrogen atmosphere at rt for 30 min. Benzoxazole ester **1a** (0.3 mmol, 1.0 equiv.) was added to the Schlenk flask containing copper complex and stirred for approximately 5 min. Propargylic carbonate **2a** (0.15 mmol, 2.0 equiv.) was then transformed into a Screw-cap reaction tube containing a nickel complex and stirred for an additional 5 min. The nickel complex solution was then combined with the copper complex solution, and the resulting solution was stirred for approximately 72 hours at 10 °C until substrate **2a** was completely consumed (monitored by TLC).

4.3 Investigation of the relationship between the enantiopurity of 4c and the diastereoselectivity of 3a

We investigated the correlation between the enantiomeric purity of nickel complex ligand **4c** and the diastereoselectivity of the resulting product **3a**, and revealed a positive correlation between the enantiomeric purity of the ligand **4c** and the Log(dr of **3a**). The specific enantiomeric excess (ee) value of ligand **4c** was prepared by combining a certain amount of optically pure **4c** with optically pure *ent*-**4c**. Nine reactions were conducted in parallel, each containing **4c** with optical purities of >-99% (*S*-**4c**), -75%, -50%, -25%, racemic (50% *R*-**4c** and 50% *S*-**4c**), 25%, 50%, 75%, and >99% (*R*-**4c**).

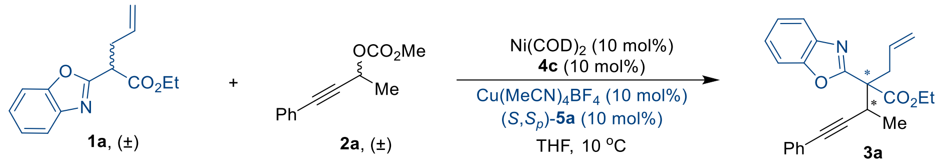

Enantiopurity of **4c** (% ee)	dr (*anti*:*syn*) of **3a**	log(dr of **3a)**
-100 (*S*)	1:40	-1.579783
-75	1:5.6	-0.748188
-50	1:3.1	-0.491362
-25	1:1.7	-0.230449
0	1:1	0
25	1.5:1	0.176091
50	2.8:1	0.447158
75	5.2:1	0.716003
100 (*R*)	25:1	1.39794

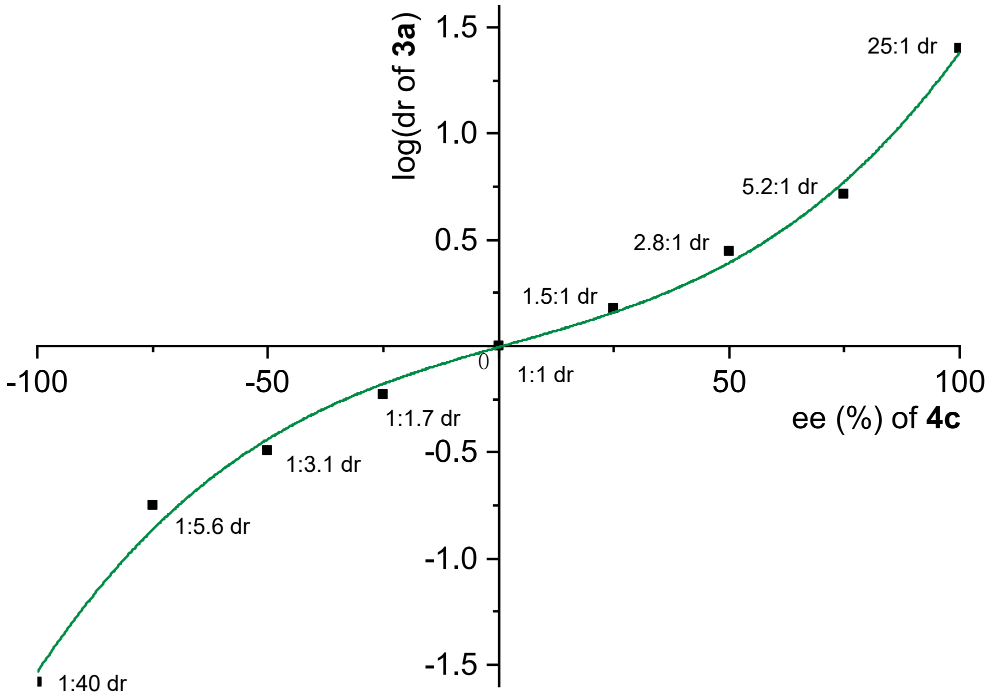

Figure S3. The relationship between the enantiopurity of 4c and the diastereoselectivity of 3a

4.4 Investigation of the relationship between the enantiopurity of 5a and the diastereoselectivity of 3a

We investigated the correlation between the enantiomeric purity of copper complex ligand **5a** and the proportion of diastereoselectivity in the resulting product **3a**, and revealed a positive correlation between the enantiomeric purity of the ligand **5a** and the Log(dr of **3a**). The specific enantiomeric excess (ee) value of ligand **5a** was prepared by combining a certain amount of optically pure **5a** with optically pure *ent*-**5a**. Nine reactions were conducted in parallel, each containing **5a** with optical purities of >-99% (R,R_p-**5a**), -75%, -50%, -25%, racemic (50% R,R_p-**5a** and 50% S,S_p-**5a**), 25%, 50%, 75%, and >99% (S,S_p-**5a**).

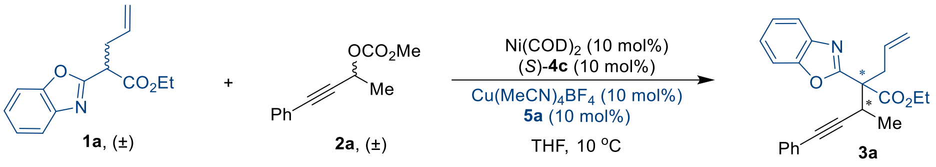

Enantiopurity of **5a** (% ee)	dr (*anti*:*syn*) of **3a**	log(dr of **3a**)
-100 (R,R_p)	1:36	-1.556303
-75	1:6.9	-0.838849
-50	1:4	-0.60206
-25	1:2.1	-0.32222
0	1:1.4	-0.146128
25	1.34:1	0.127105
50	2:1	0.30103
75	4.2:1	0.623249
100 (S,S_p)	25:1	1.39794

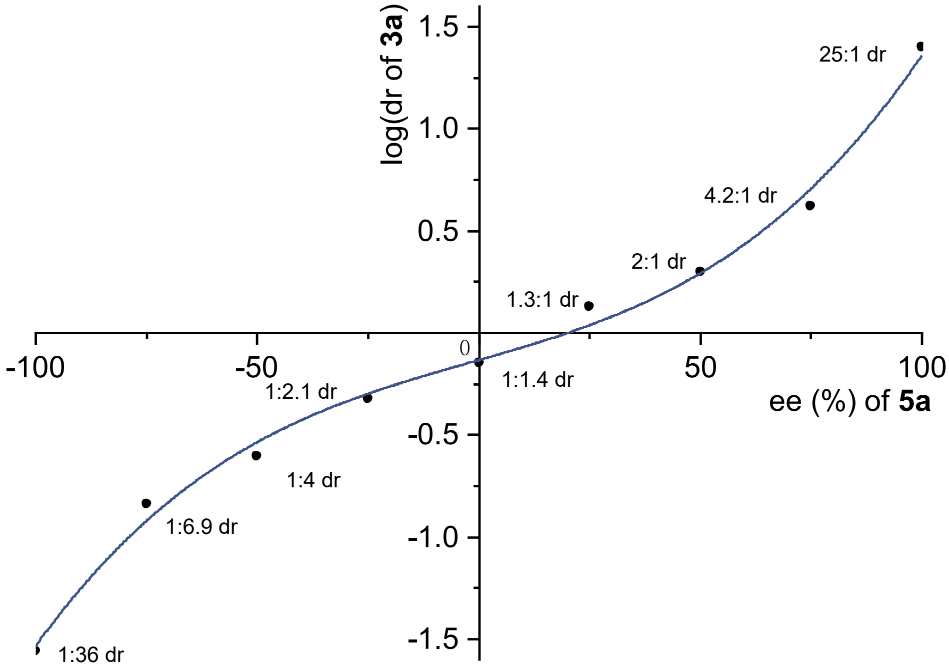

Figure S4. The relationship between the enantiopurity of 5a and the diastereoselectivity of 3a

4.5 Nonlinear effect investigation

We conducted a nonlinear effect study to investigate the relationship between the enantiopurity of ligand **5a** of chiral copper complexes and the enantiomeric purity of the generated product **7a**. The specific enantiomeric excess (ee) value of ligand **5a** was prepared by combining a certain amount of optically pure **5a** with optically pure *ent*-**5a**. Nine reactions were conducted in parallel, each containing **5a** with optical purities of >-99% (R,R_p-**5a**), -75%, -50%, -25%, racemic (50% R,R_p-**5a** and 50% S,S_p-**5a**), 25%, 50%, 75%, and >99% (S,S_p-**5a**).

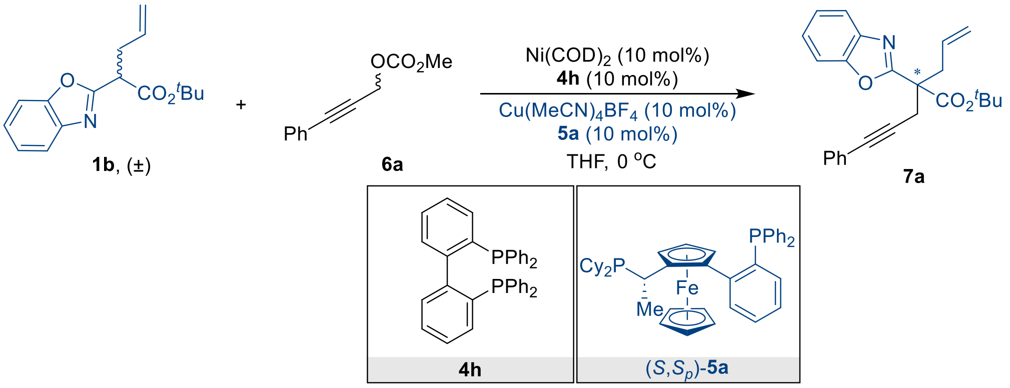

Entry	Enantiopurity of **5a** (% ee)	Enantiopurity of **7a** (% ee)
1	-100 (R,R_p)	-81
2	-75	-59
3	-50	-39
4	-25	-18
5	0	0
6	25	21
7	50	42
8	75	59
9	100 (S,S_p)	81

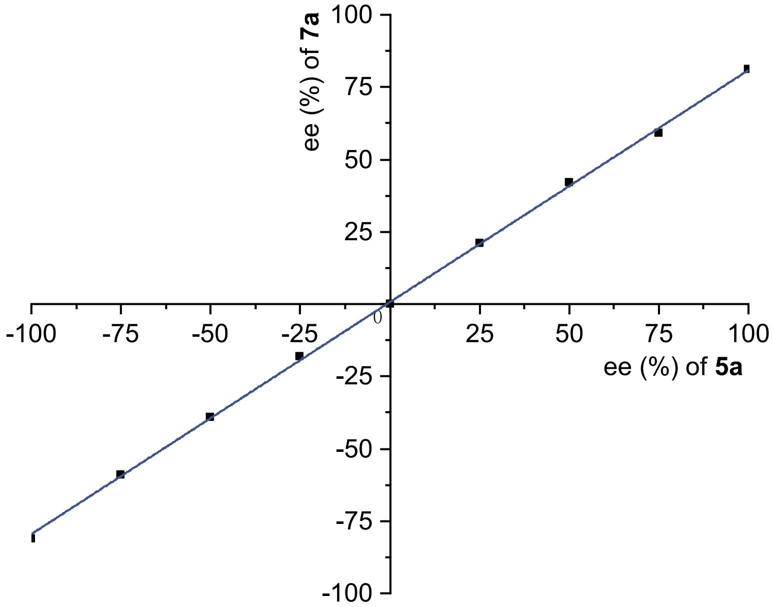

Figure S5. Nonlinear effect investigation

4.6 ^{31}P NMR studies

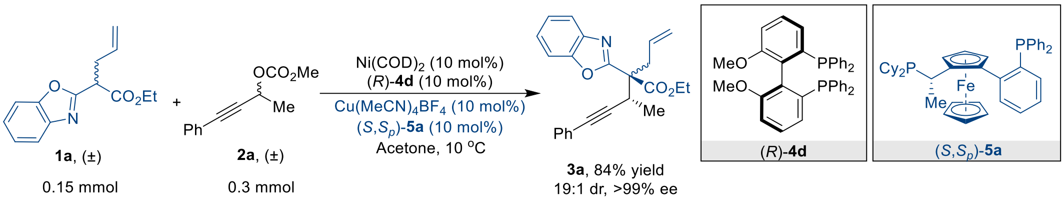

Under template reaction conditions, using (*R*)-**4d** as the ligand for the nickel complex and acetone as the reaction solvent, the template reaction can still yield the target compound **3a** with excellent results. The ^{31}P NMR spectra at 20 °C were recorded with a Bruker AV 400 MHz spectrometer. The ^{31}P NMR spectra in Figure S6 were done in a J. Young tube.

To further investigate the effect of the coordination interactions between the nickel complex and copper complex, in situ ^{31}P NMR studies were conducted to identify reactive intermediates potentially participating in the catalytic cycle (Figure S6). Upon the combination of $Ni(COD)_2$ and diphosphine ligand (*R*)-**4d**, a distinct singlet peak at δ = 31.6 ppm was identified in the ^{31}P NMR spectra (Figure S6, **II** vs **I**). The addition of propargylic carbonate **2a** led to the disappearance of the Ni[(*R*)-**4d**] complex and concomitantly the formation of a new allenylnickel species {Ni^+(allenyl)[(*R*)-**4d**]}($^-OCO_2Me$) complex, characterized by two doublets at δ = 37.1 ppm (d, *J* = 24.4 Hz) and 34.6 ppm (d, *J* = 24.4 Hz) (Figure S6, **III**). Furthermore, the co-mixing of the Cu[(*S,Sp*)-**5a**] complex and the allenylnickel species {Ni^+(allenyl)[(*R*)-**4d**]}($^-OCO_2Me$) complex has no effect on the peak positions of the individual complexes, nor does it give rise to the appearance of any new peaks (Figure S6, **V** vs **III** and **IV**). The results suggest that ligand exchange between the nickel complex and the copper complex may not have occurred.

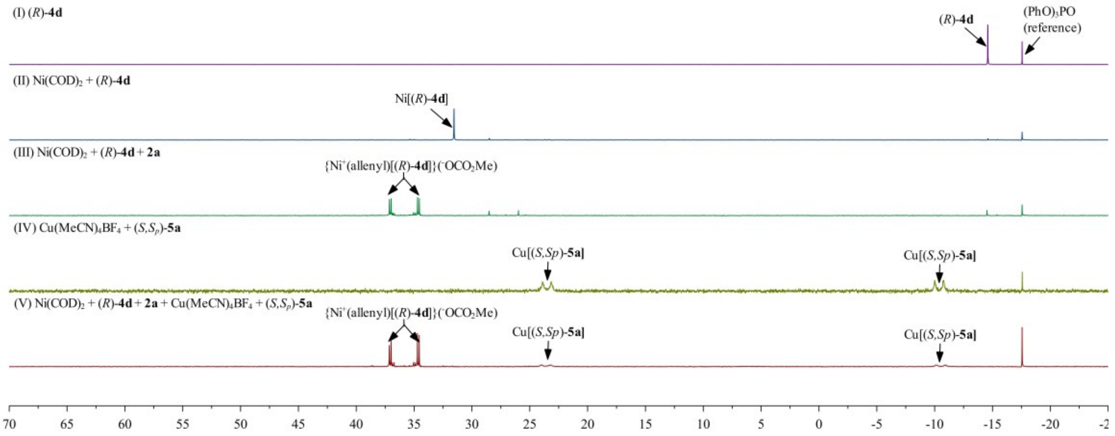

Figure S6. ^{31}P NMR spectra. (**I**) (*R*)-**4d** (11.6 mg, 0.02 mmol), triphenyl phosphate (2 mg, reference), and acetone-d6 (0.6 mL) were added to a J. Young tube, and the sample was analyzed by ^{31}P NMR; (**II**) $Ni(COD)_2$ (5.5 mg, 0.02 mmol, 1.0 equiv), (*R*)-**4d** (11.6 mg, 0.02 mmol, 1.0 equiv), and acetone-d6 (0.6 mL) were added to a J. Young tube in the glove box. The solution was shaken for 30 minutes at room temperature, then triphenyl phosphate (2 mg) was added and the sample was analyzed by ^{31}P NMR; (**III**) $Ni(COD)_2$ (5.5 mg, 0.02 mmol, 1.0 equiv), (*R*)-**4d** (11.6 mg, 0.02 mmol, 1.0 equiv), and acetone-d6 (0.6 mL) were added to a J. Young tube in the glove box. After the solution was

shaken for 30 minutes, (*rac*)-**2a** (4.1 mg, 0.02mmol, 1.0 equiv) was added, and the solution was shaken for 30 minutes. Then triphenyl phosphate (2 mg) was added and the sample was analyzed by ^{31}P NMR; (**IV**) $Cu(MeCN)_4BF_4$ (6.3 mg, 0.02 mmol, 1.0 equiv), (*S,Sp*)-**5a** (13.4 mg, 0.02 mmol, 1.0 equiv), and acetone-d6 (0.6 mL) were added to a J. Young tube in the glove box. The solution was shaken for 30 minutes at room temperature, then triphenyl phosphate (2 mg) was added and the sample was analyzed by ^{31}P NMR; (**V**) $Ni(COD)_2$ (5.5 mg, 0.02 mmol, 1.0 equiv), (*R*)-**4d** (11.6 mg, 0.02 mmol, 1.0 equiv), and acetone-d6 (0.6 mL) were added to a J. Young tube in the glove box. After the solution was shaken for 30 minutes, (*rac*)-**1a** (4.1 mg, 0.02 mmol, 1.0 equiv) was added and shaken for 30 minutes. $Cu(MeCN)_4BF_4$ (6.3 mg, 0.02 mmol, 1.0 equiv), (*S,Sp*)-**5a** (13.4 mg, 0.02 mmol, 1.0 equiv), and acetone-d6 (0.6 mL) were added to another J. Young tube in the glove box. The solution was shaken for 30 minutes at room temperature. The copper complex was then added into the reaction system containing the allenylnickel species {Ni^+(allenyl)[(*R*)-**4d**]}($^-OCO_2Me$) and shaken for 30 minutes. Subsequently, then triphenyl phosphate (2 mg) was added and the sample was analyzed by ^{31}P NMR.

5. X-ray crystallography data

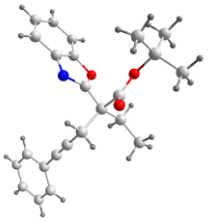

(CCDC **2311196**)

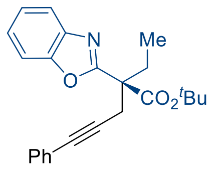

7c

Chemical formula	$C_{24}H_{25}NO_3$
Formula weight	375.45
Space group	P 1 21 1
Z	2
α, Å	8.9430(3)
b, Å	10.4307(4)
c, Å	11.7728(5)
α,°	90
β,°	101.053(2)
γ,°	90
V, Å^3	1077.82(7)
Flack parameter	0.12(15)

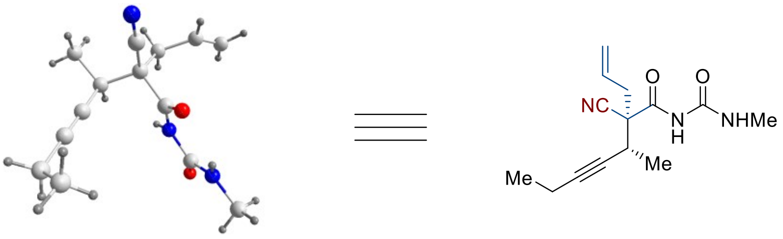

(CCDC **2311197**)　　　　(*S*,*R*)-**20**

Chemical formula	$C_{14}H_{19}N_3O_2$
Formula weight	261.32
Space group	P 1 21 1
Z	4
α, Å	7.8515(2)
b, Å	19.8312(4)
c, Å	10.1008(2)
α,°	90
β,°	110.2460(10)
γ,°	90
V, Å^3	1475.57(6)
Flack parameter	0.03(11)

6. NMR spectra

^{1}H NMR of (*S*,*R*)-**3a (500 MHz, CDCl3)**

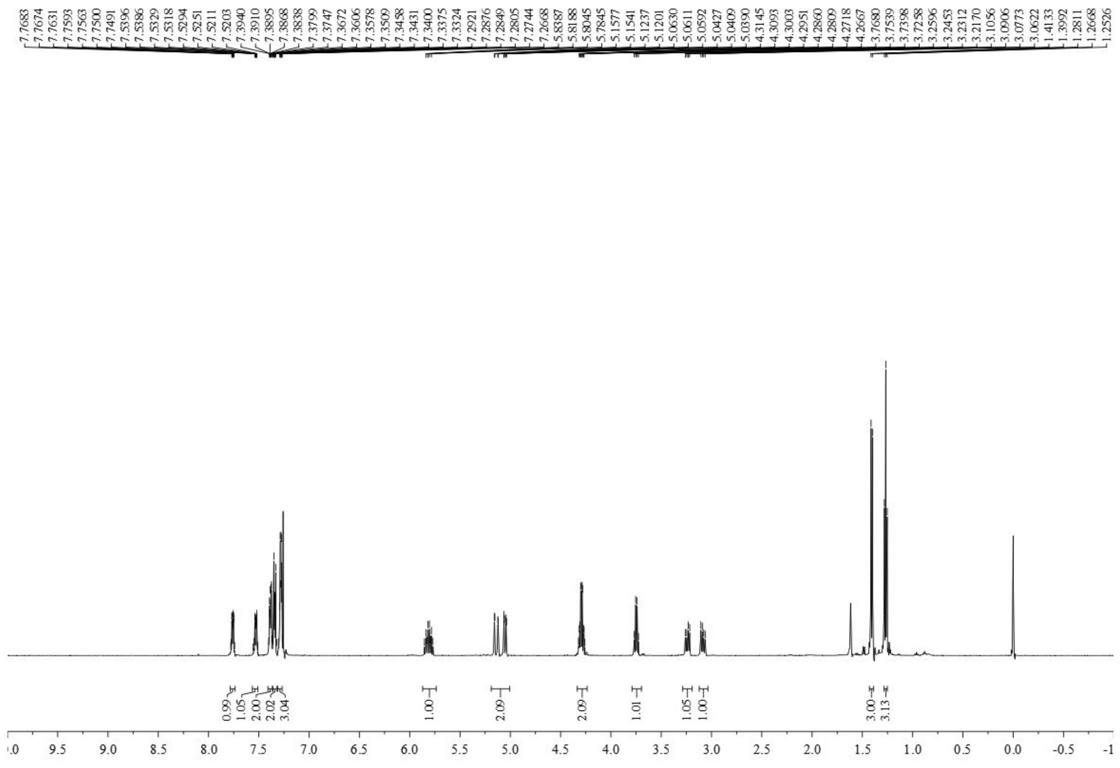

^{13}C NMR of (*S*,*R*)-**3a (125 MHz, CDCl3)**

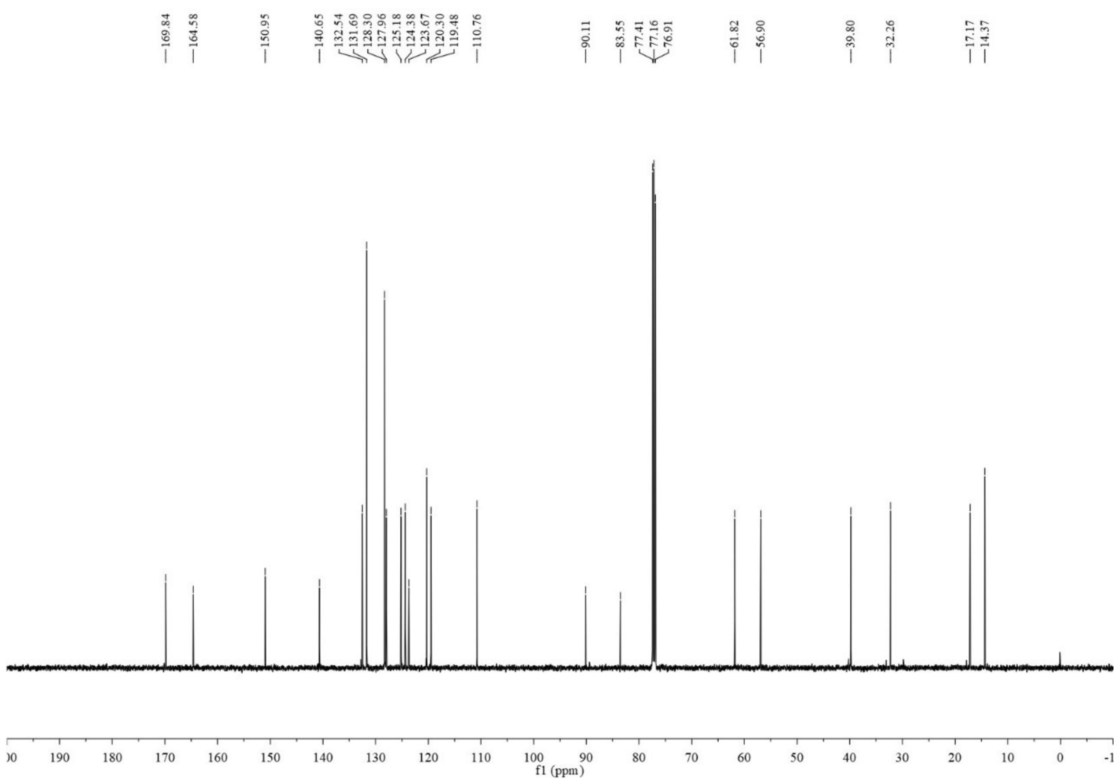

^{1}H NMR of (*R*,*S*)**-3a (400 MHz, CDCl$_3$)**

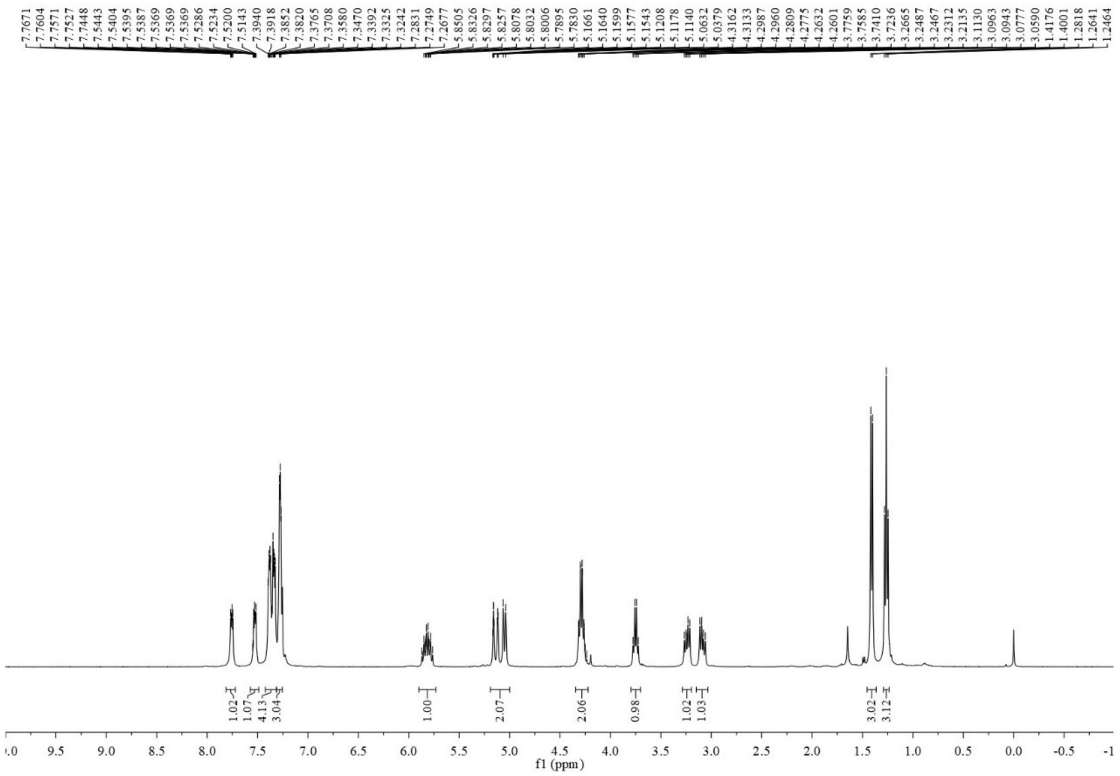

^{13}C NMR of (*R*,*S*)**-3a (100 MHz, CDCl$_3$)**

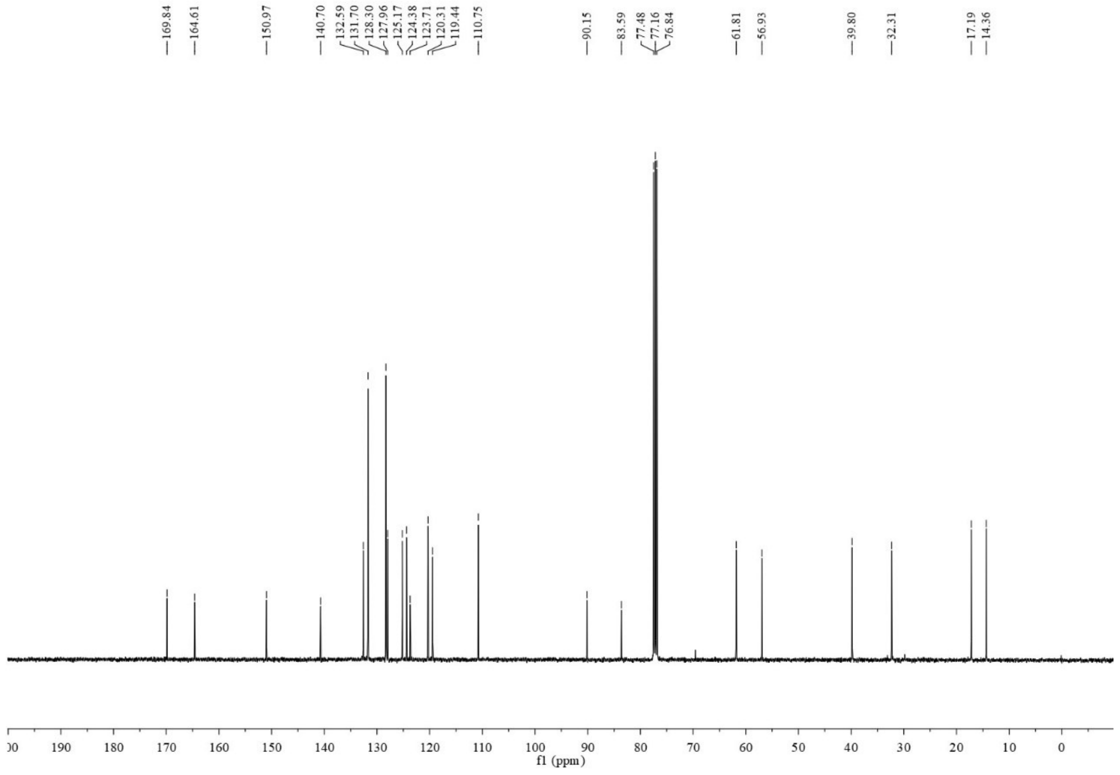

^{1}H NMR of (*R*,*R*)-**3a (500 MHz, CDCl$_3$)**

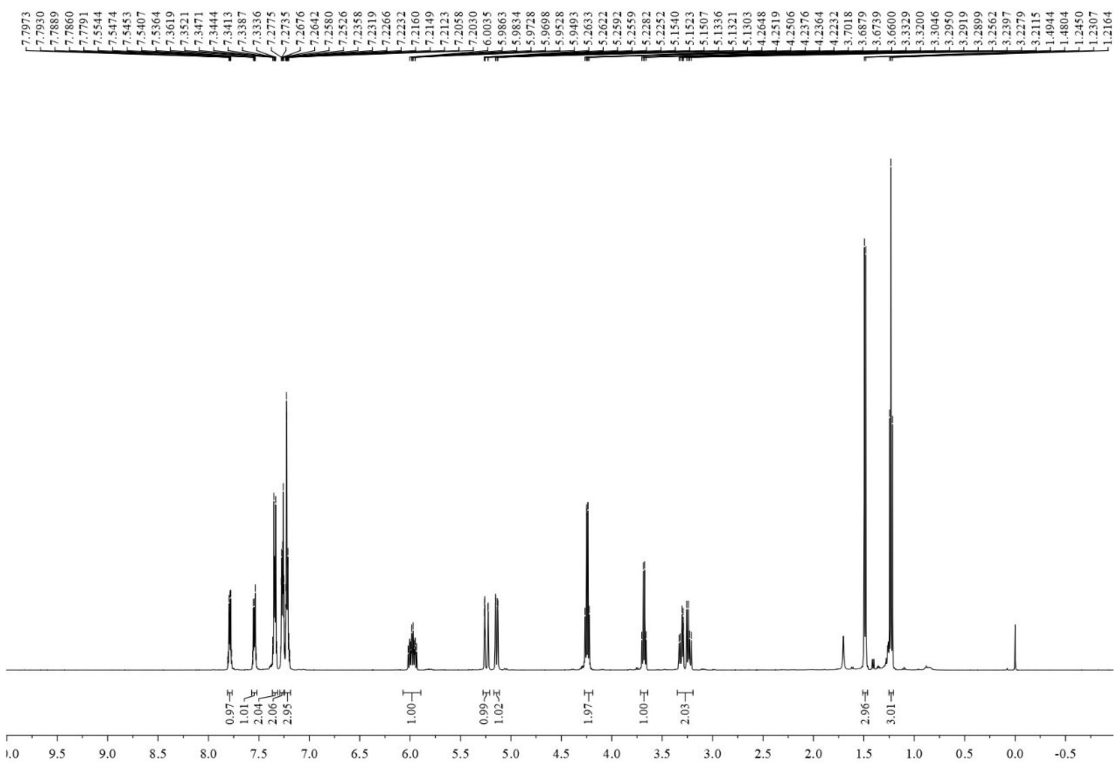

^{13}C NMR of (*R*,*R*)-**3a (125 MHz, CDCl$_3$)**

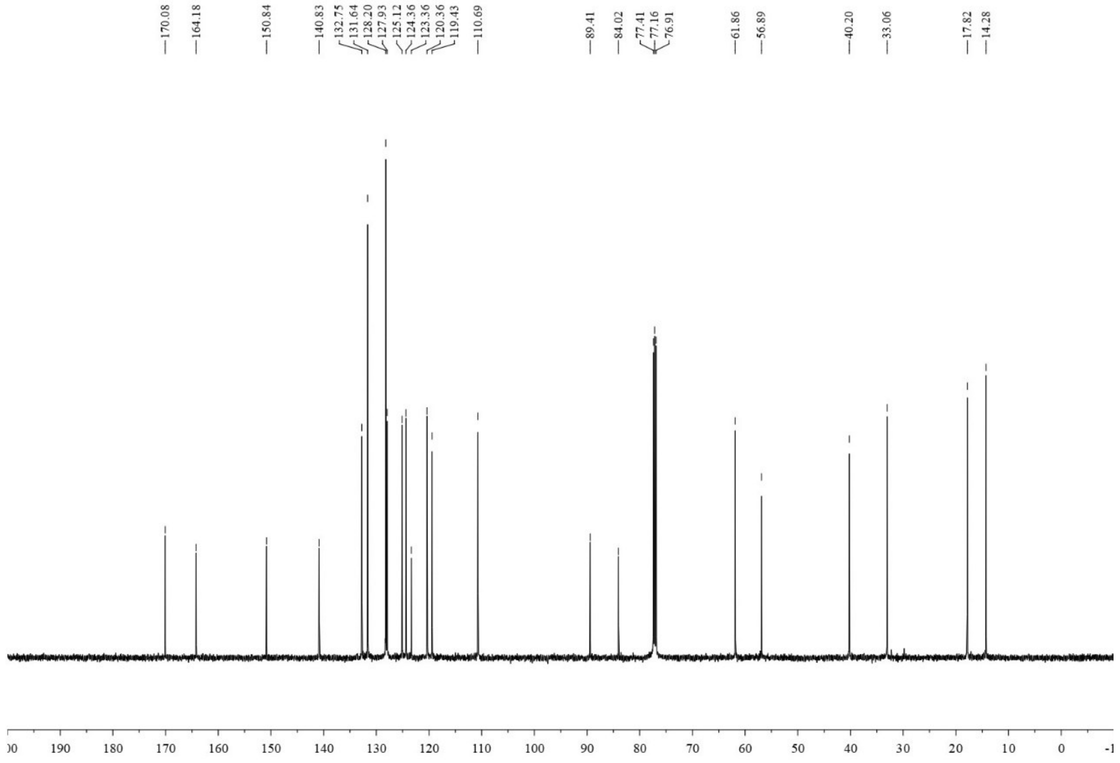

^{1}H NMR of (*S*,*S*)-3a (500 MHz, $CDCl_3$)

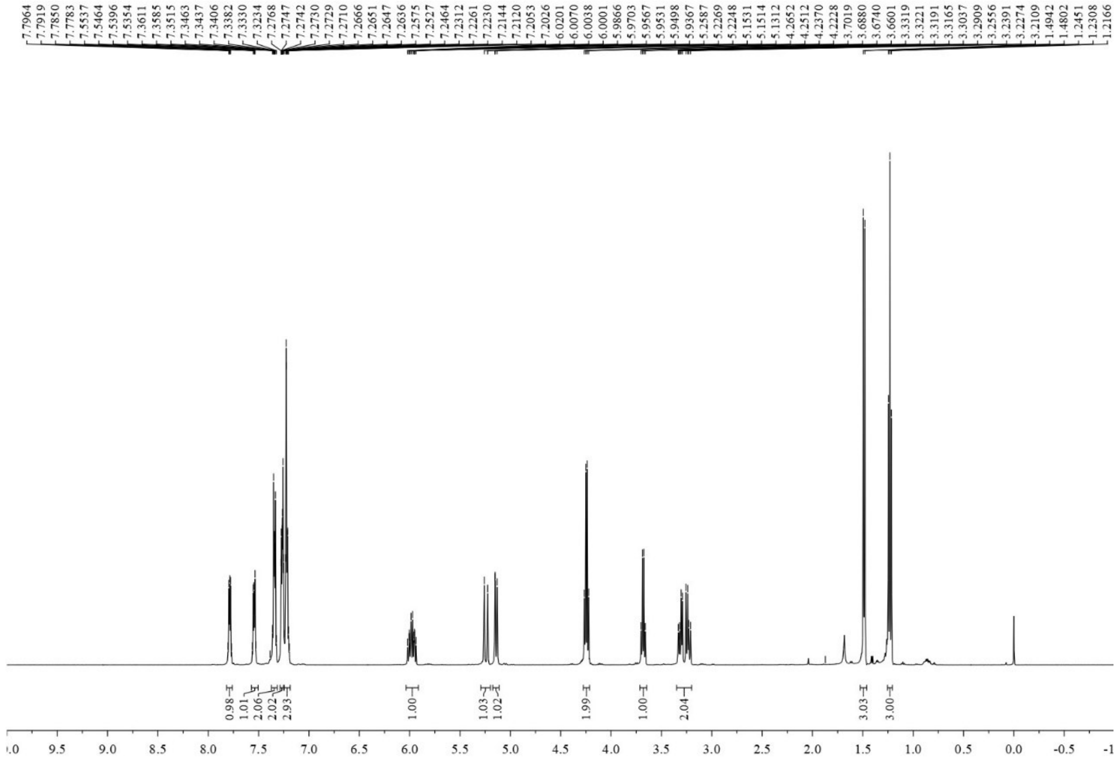

^{13}C NMR of (*S*,*S*)-3a (125 MHz, $CDCl_3$)

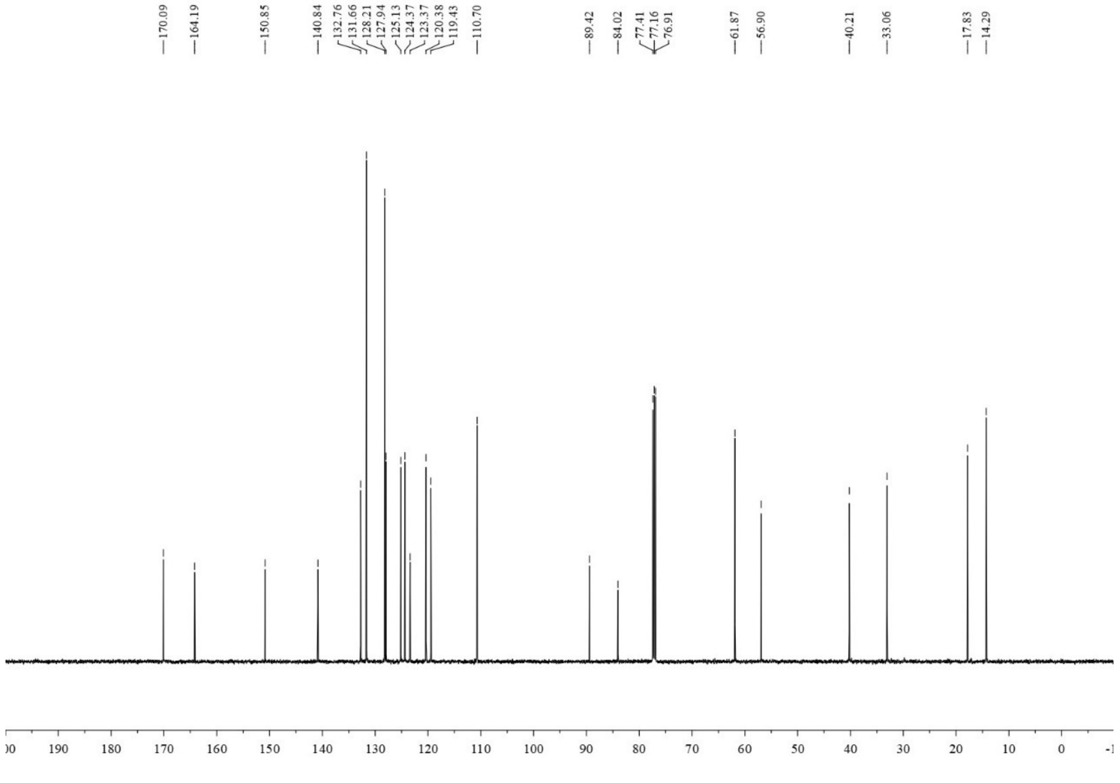

^{1}H NMR of **3b (500 MHz, CDCl$_3$)**

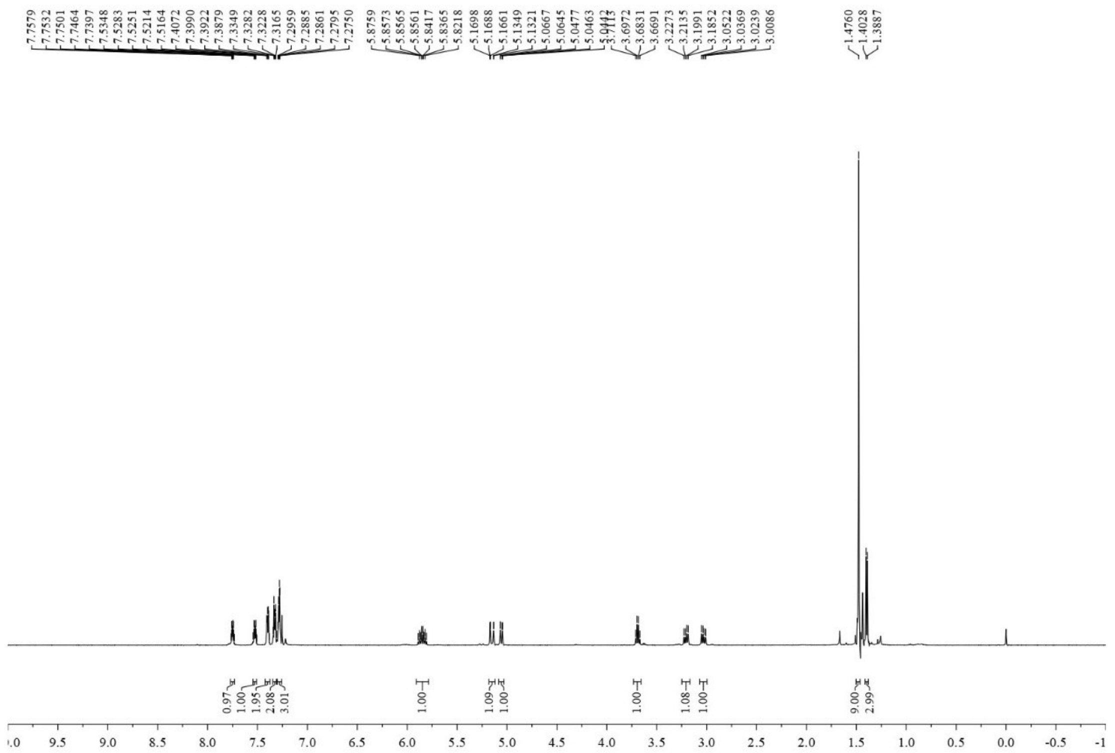

^{13}C NMR of **3b (125 MHz, CDCl$_3$)**

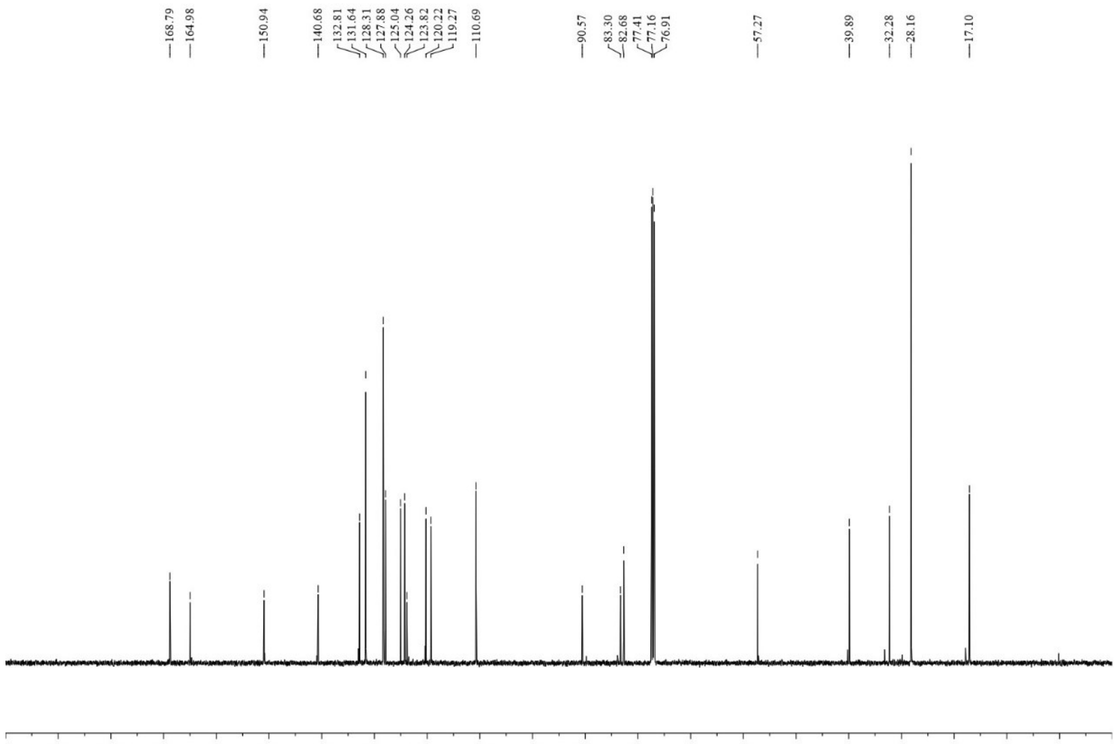

^{1}H NMR of **3c (500 MHz, CDCl₃)**

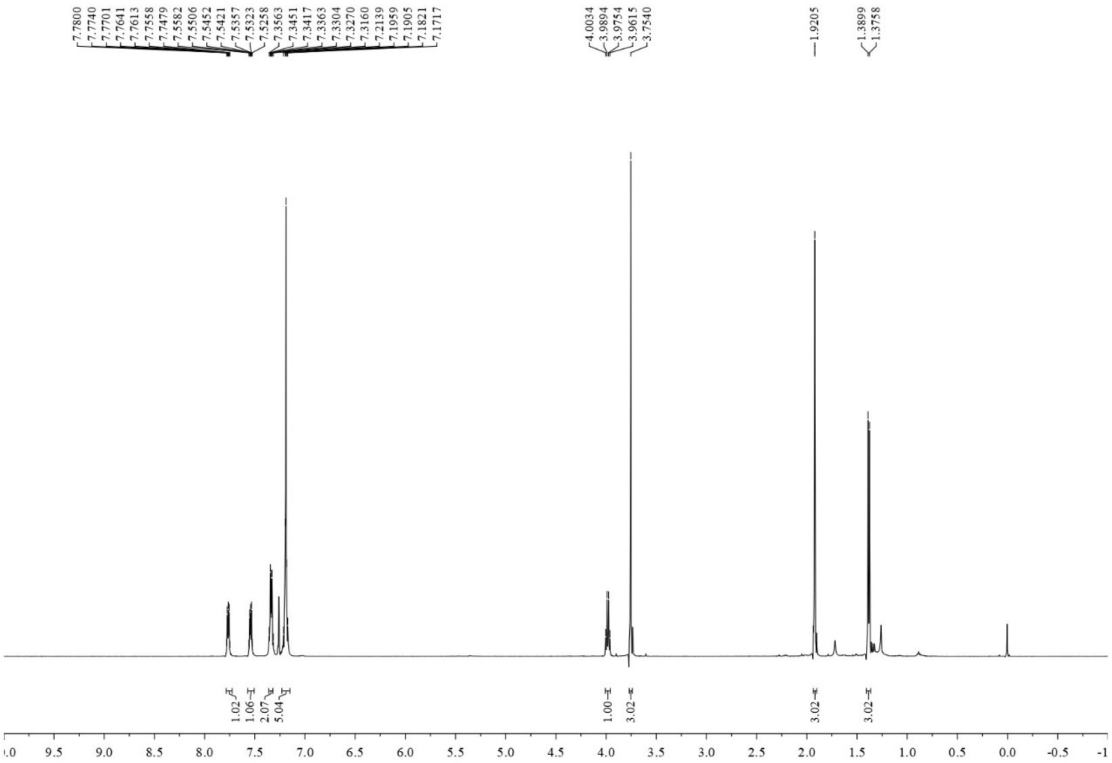

^{13}C NMR of **3c (125 MHz, CDCl₃)**

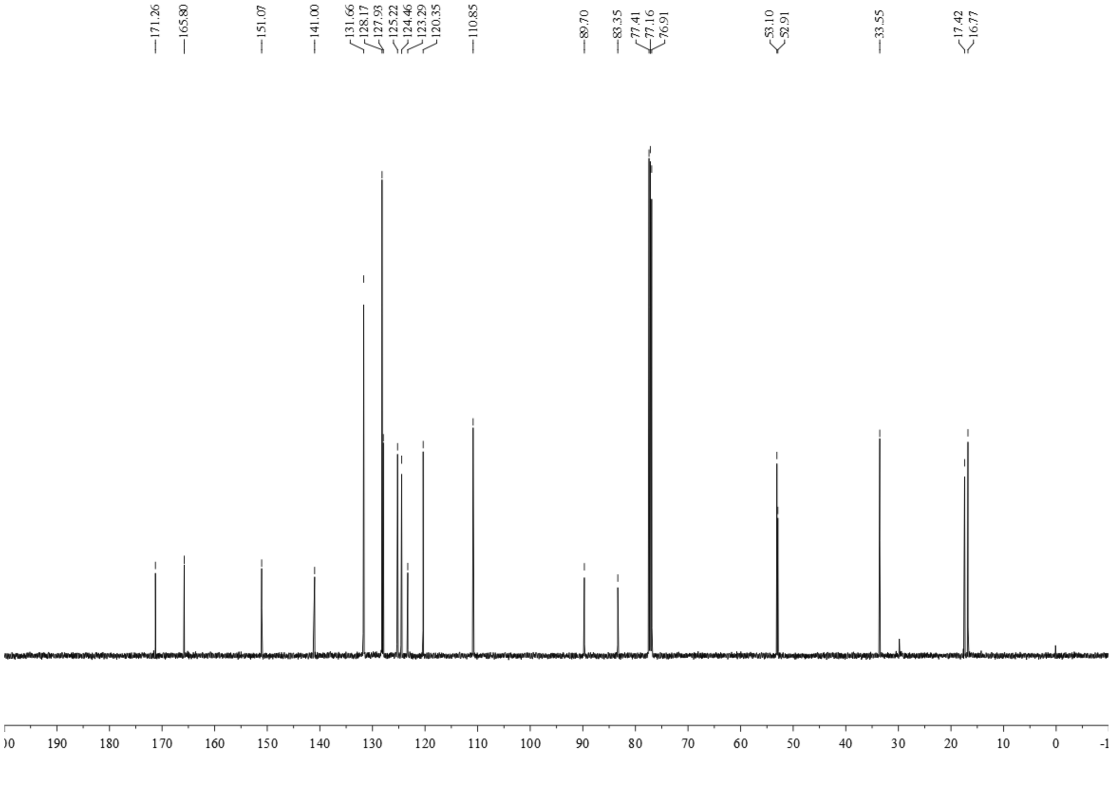

^{1}H NMR of **3d (500 MHz, CDCl$_3$)**

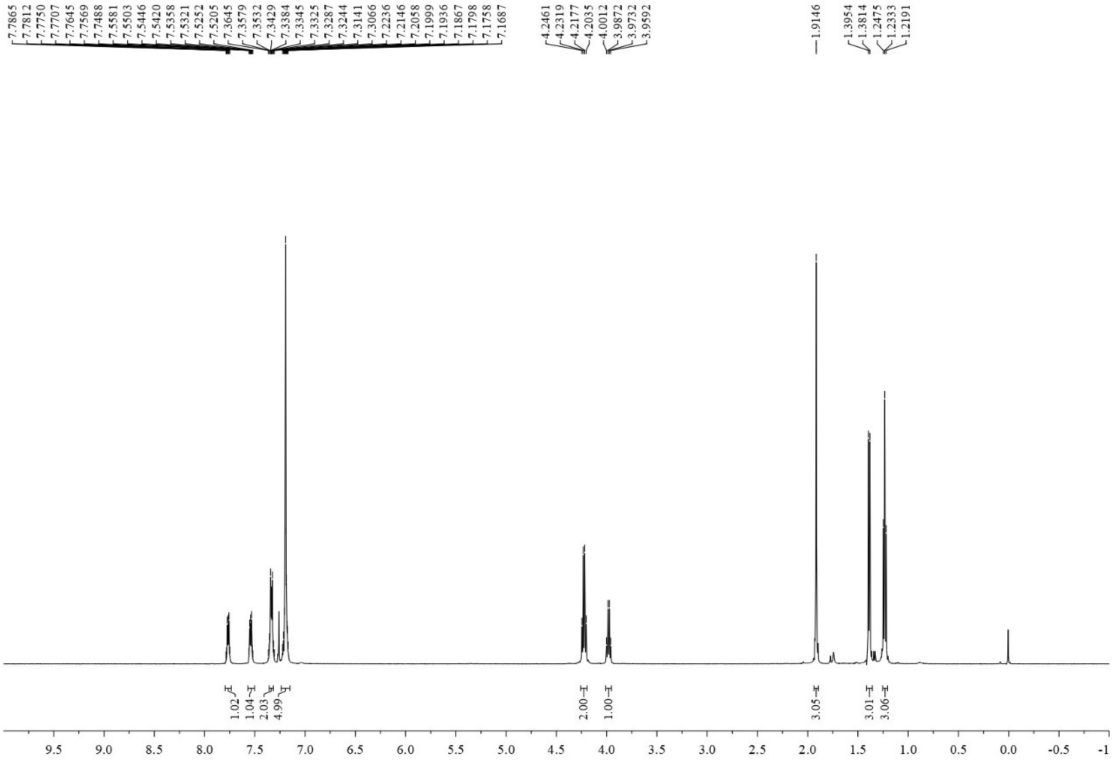

^{13}C NMR of **3d (125 MHz, CDCl$_3$)**

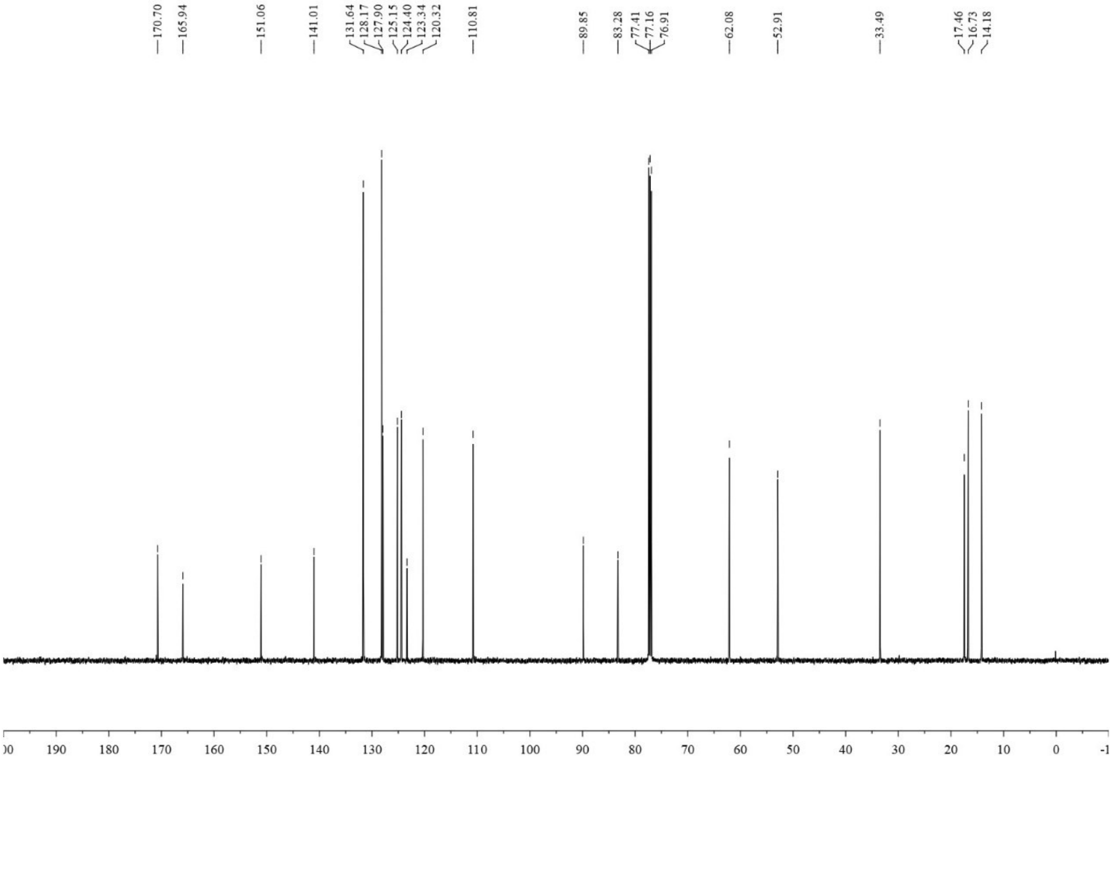

^{1}H NMR of 3e (500 MHz, $CDCl_3$)

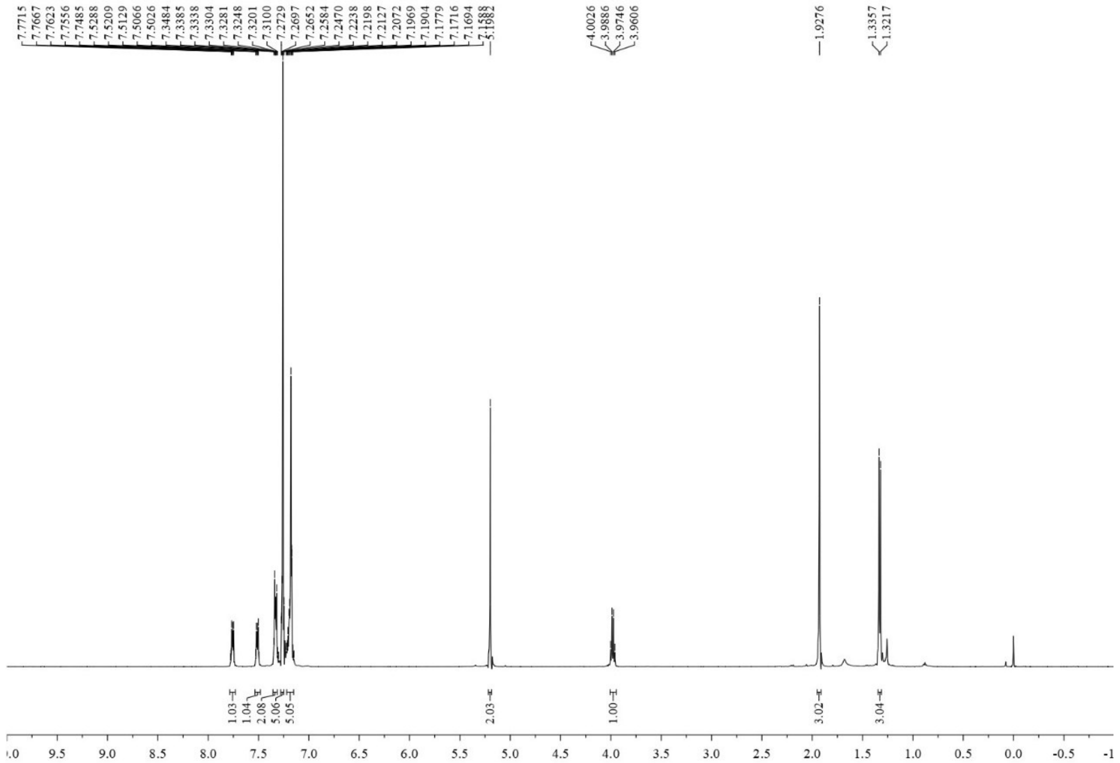

^{13}C NMR of 3e (125 MHz, $CDCl_3$)

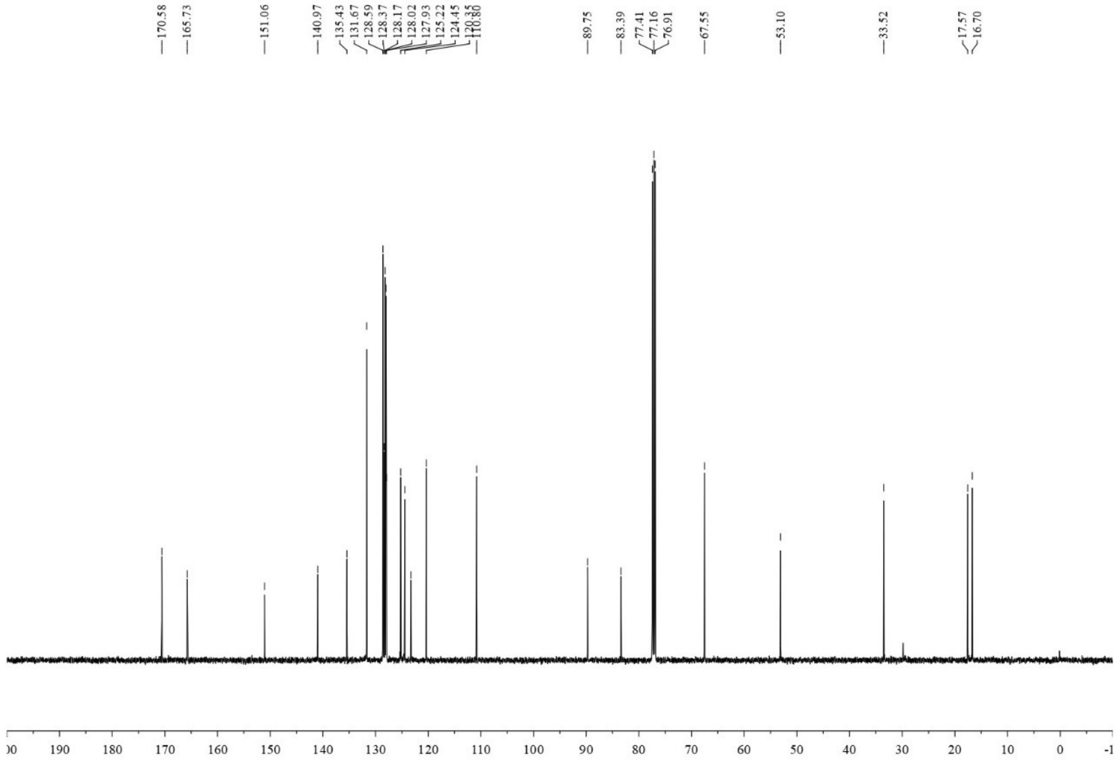

^{1}H NMR of **3f (500 MHz, CDCl$_3$)**

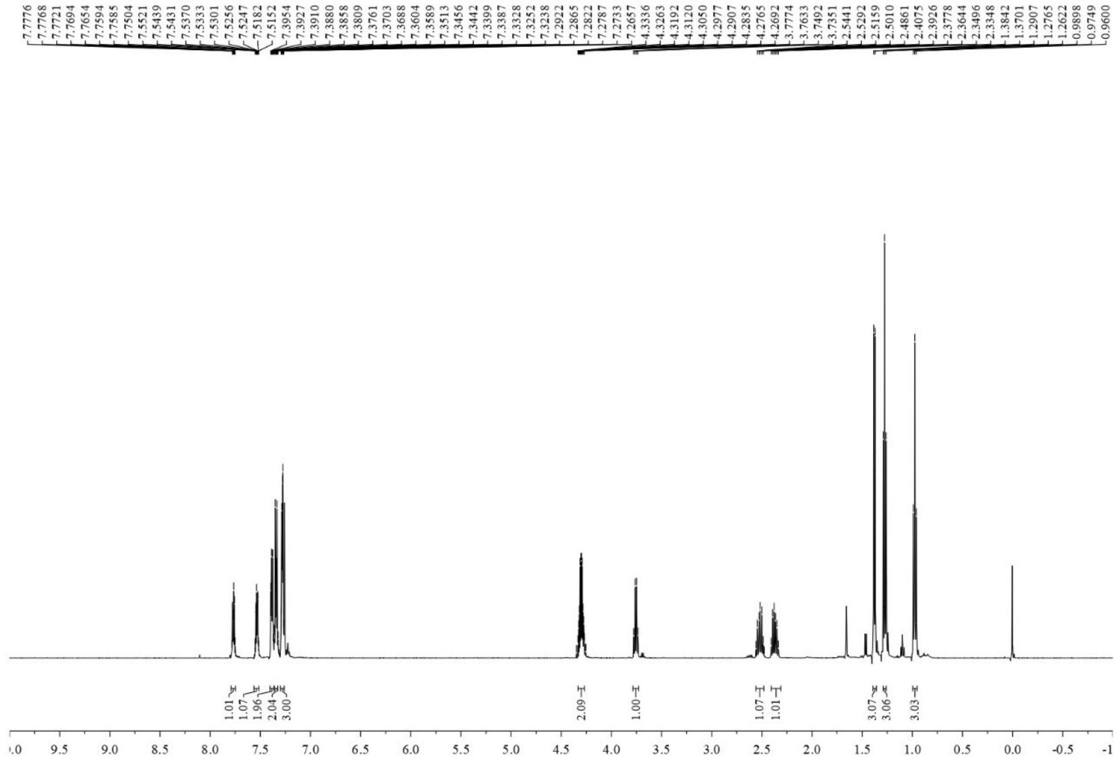

^{13}C NMR of **3f (125 MHz, CDCl$_3$)**

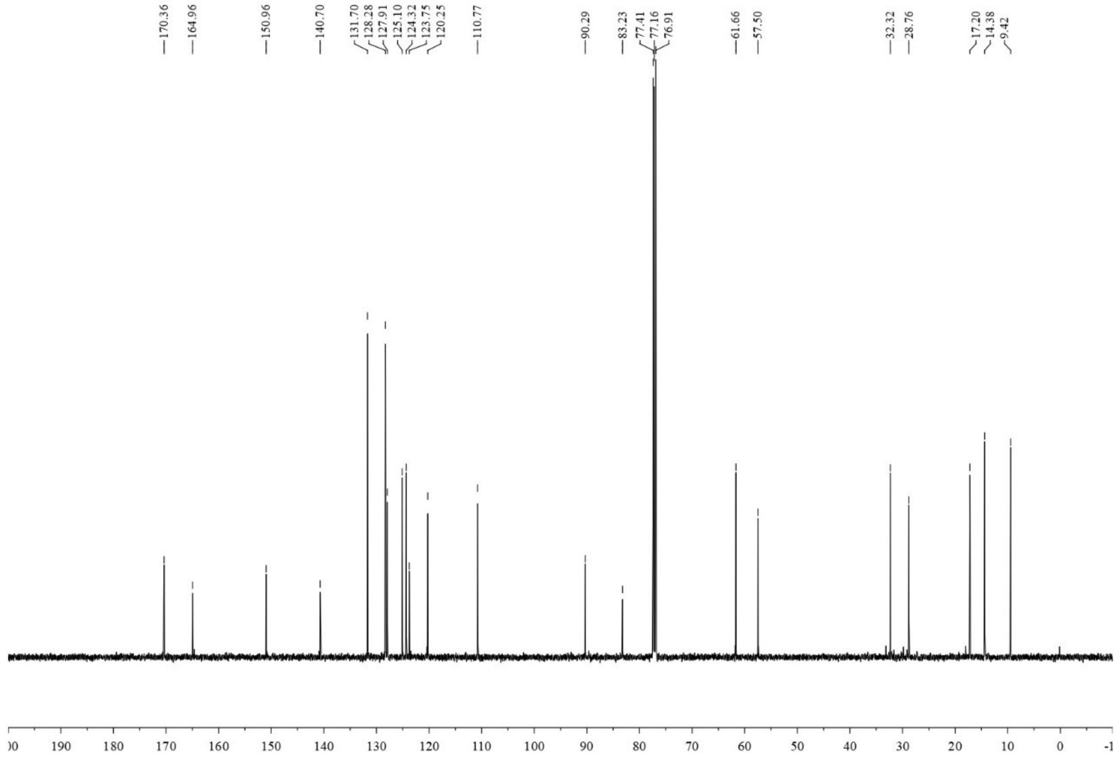

^{1}H NMR of **3g (500 MHz, CDCl$_3$)**

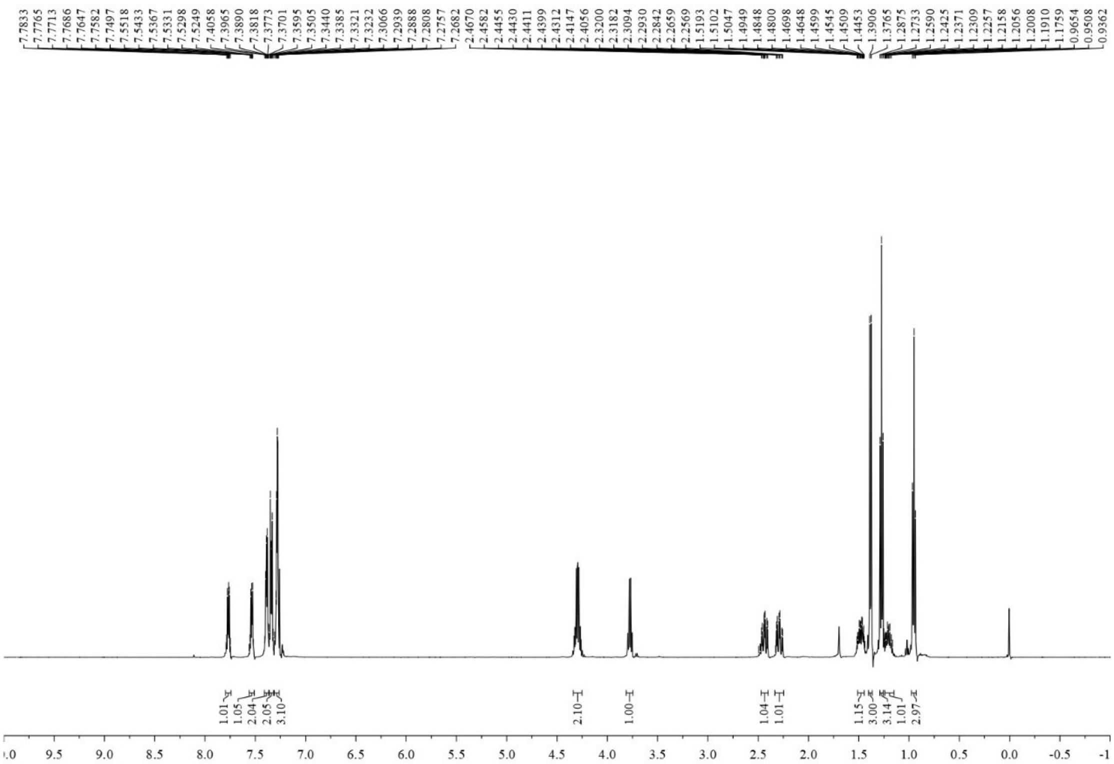

^{13}C NMR of **3g (125 MHz, CDCl$_3$)**

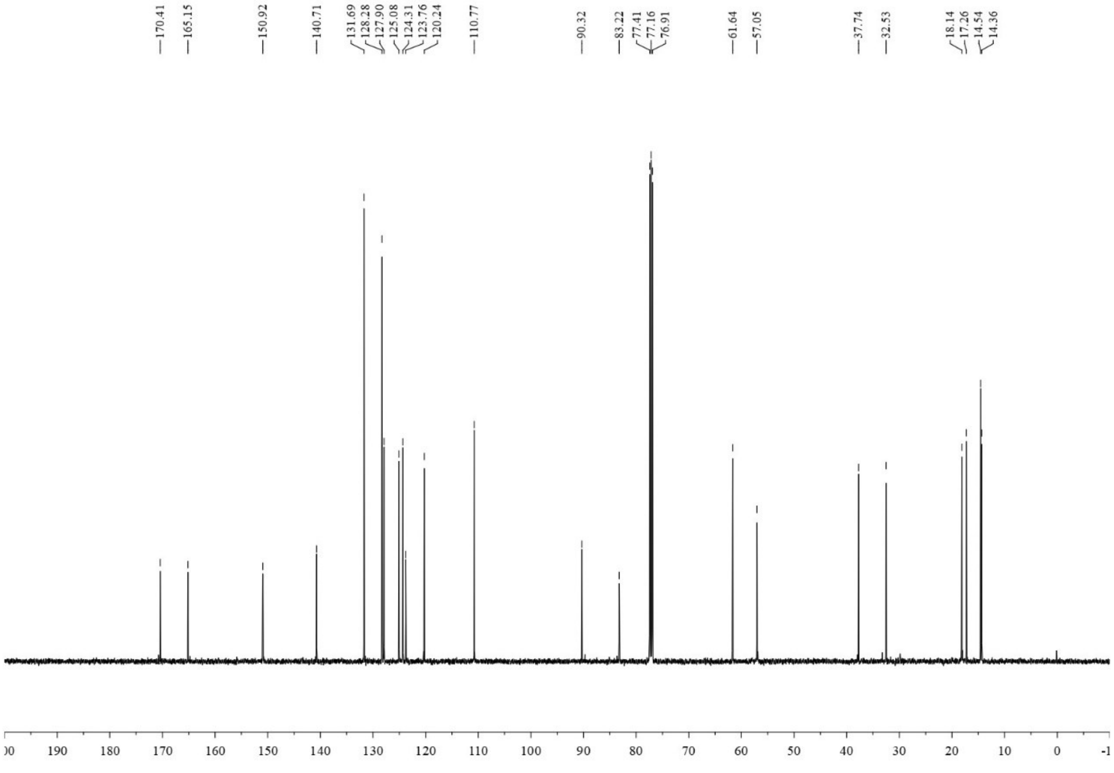

^{1}H NMR of **3h (500 MHz, CDCl$_3$)**

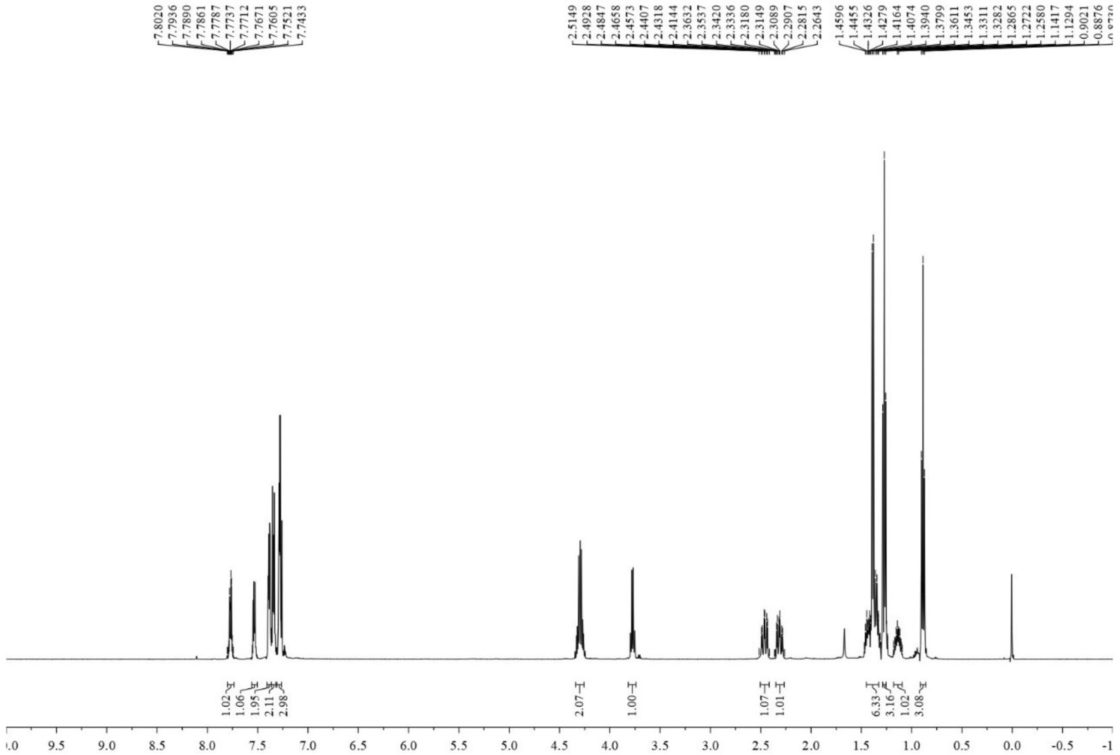

^{13}C NMR of **3h (125 MHz, CDCl$_3$)**

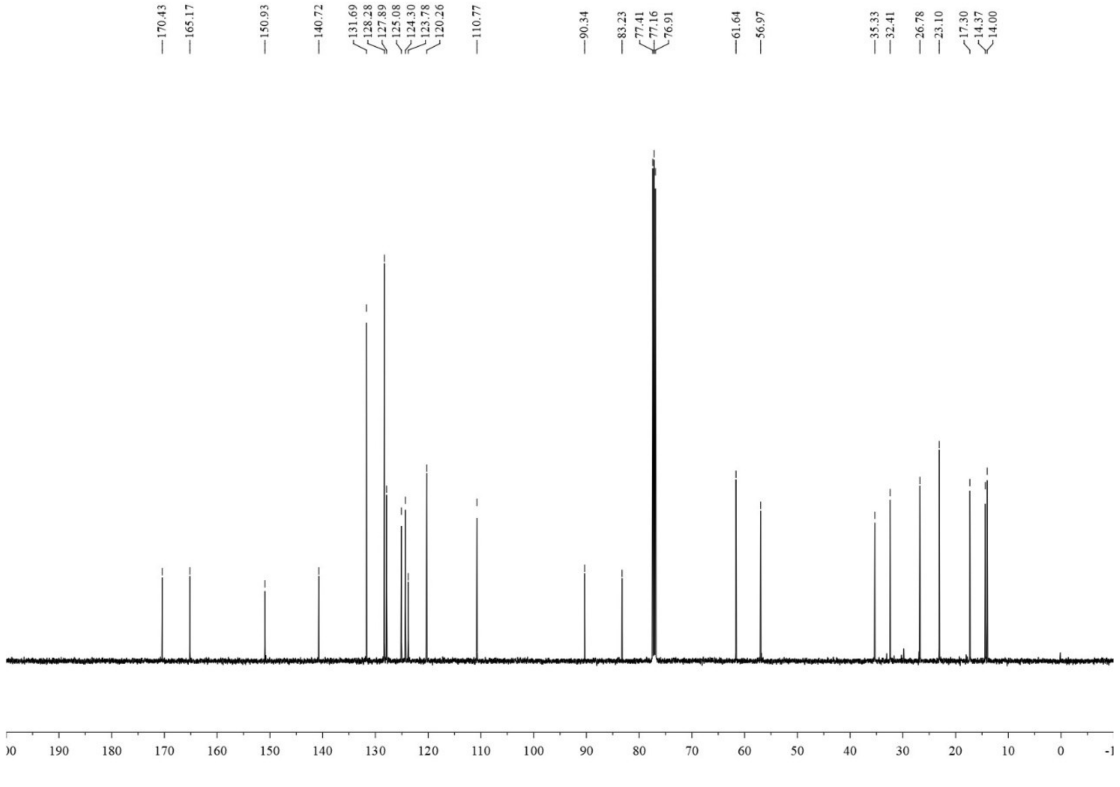

^{1}H NMR of **3i (500 MHz, CDCl$_3$)**

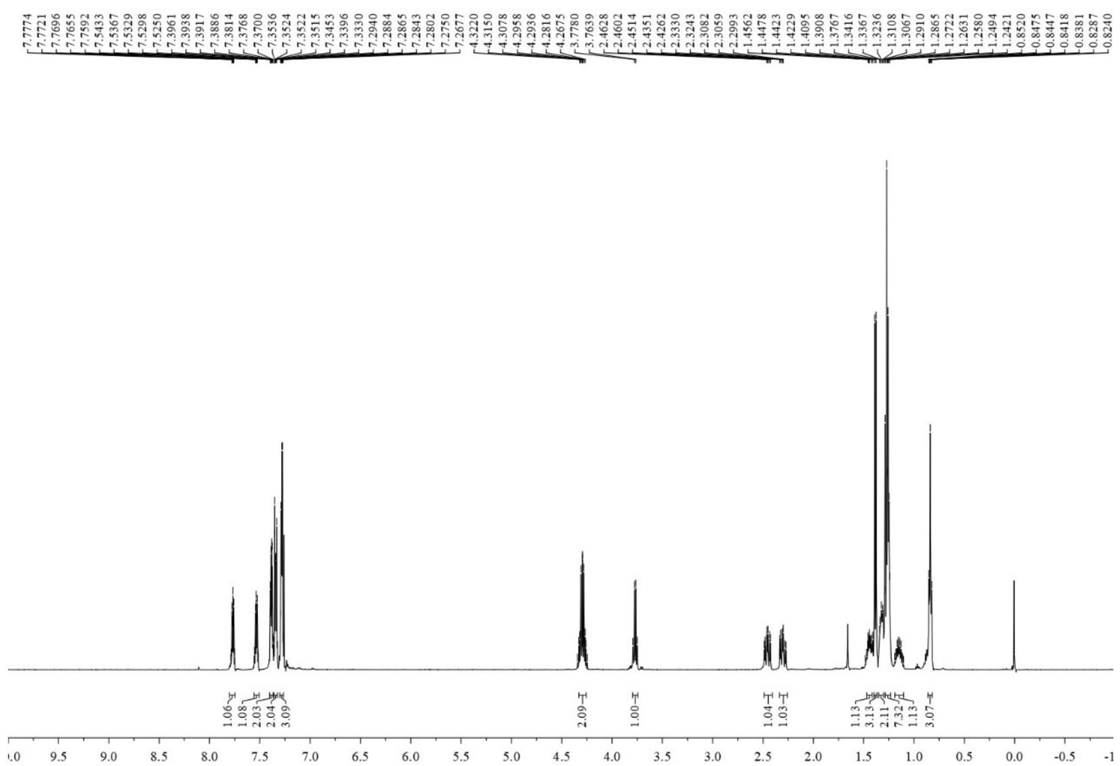

^{13}C NMR of **3i (125 MHz, CDCl$_3$)**

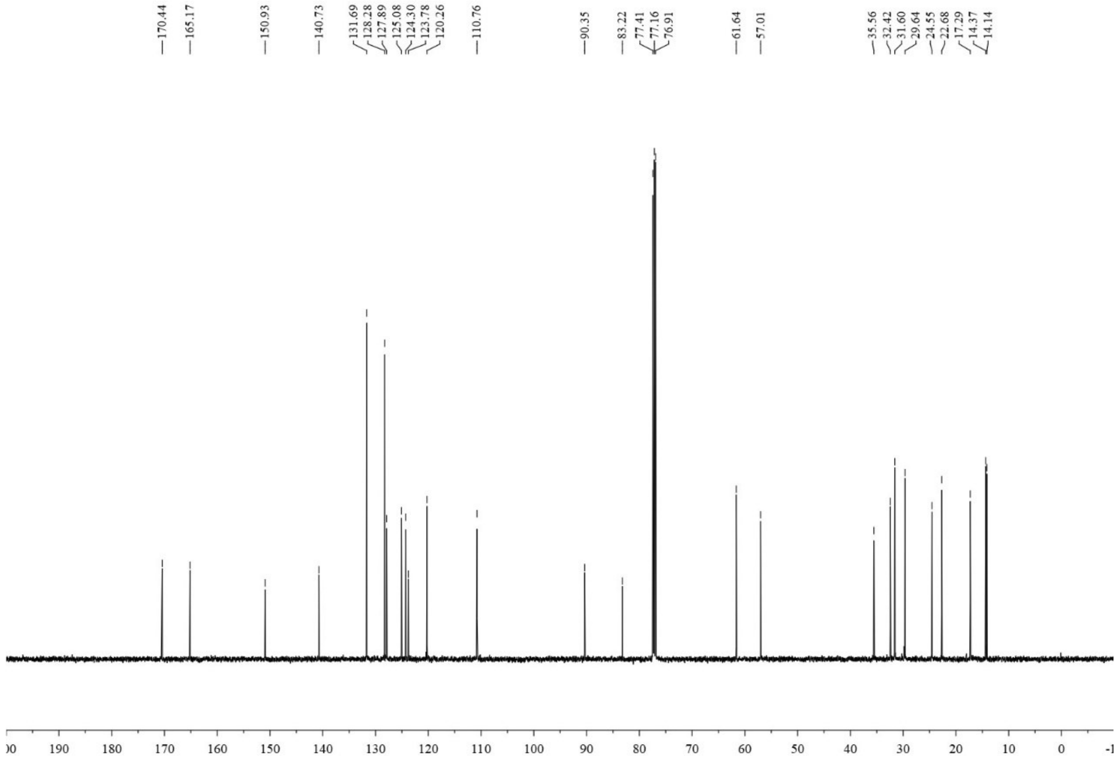

^{1}H NMR of **3j (500 MHz, CDCl$_3$)**

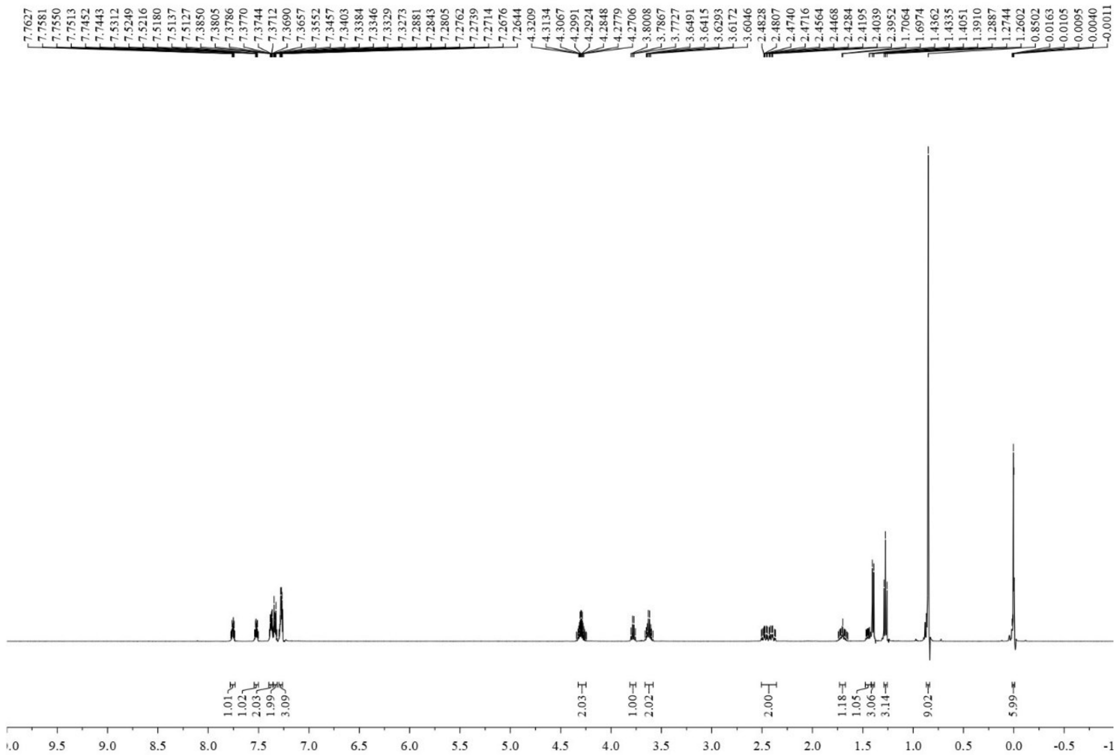

^{13}C NMR of **3j (125 MHz, CDCl$_3$)**

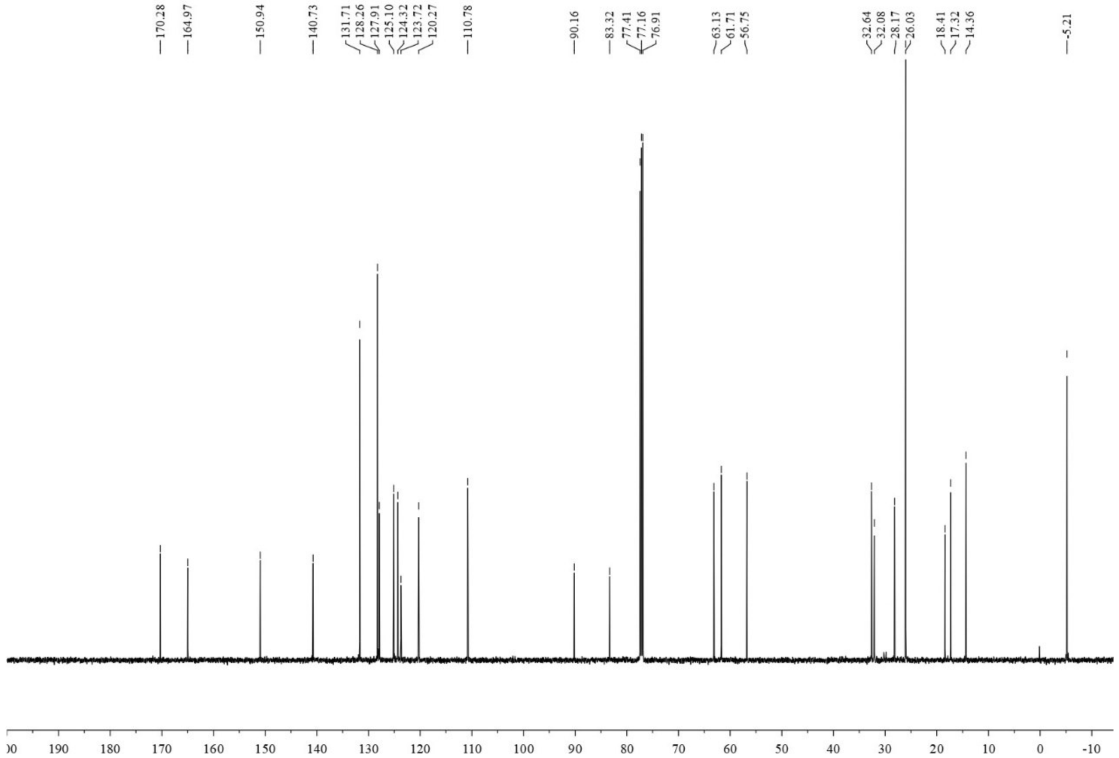

^{1}H NMR of **3k (500 MHz, $CDCl_3$)**

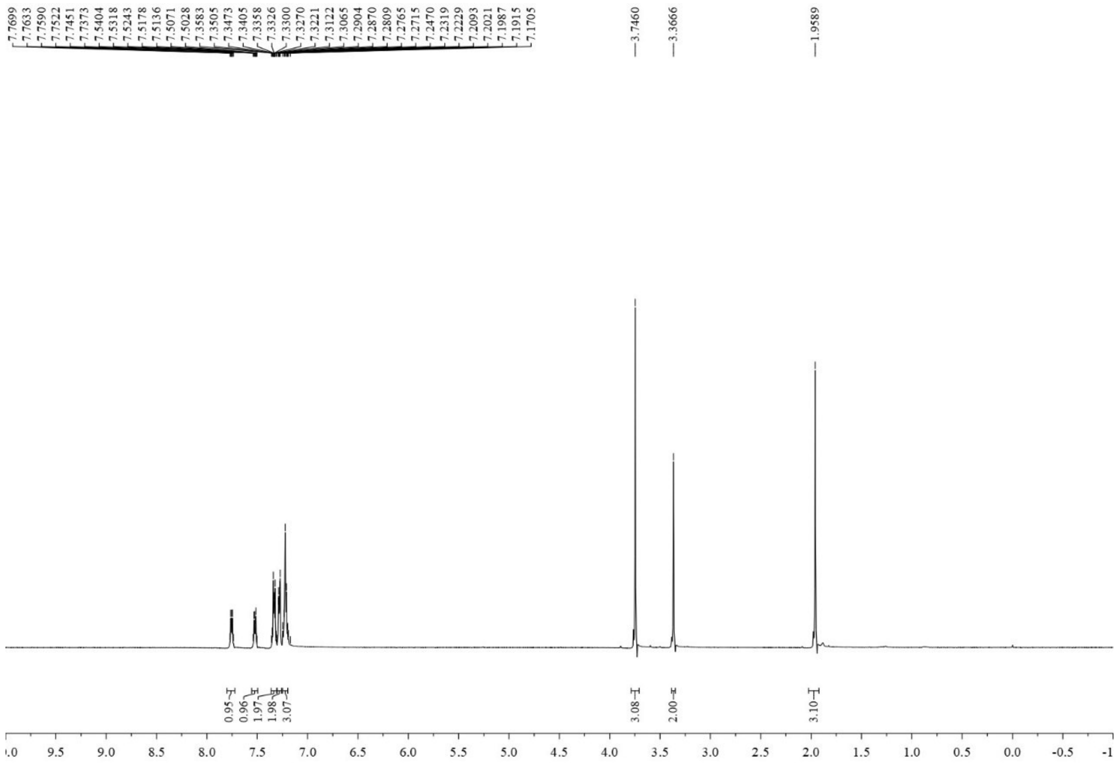

^{13}C NMR of **3k (125 MHz, $CDCl_3$)**

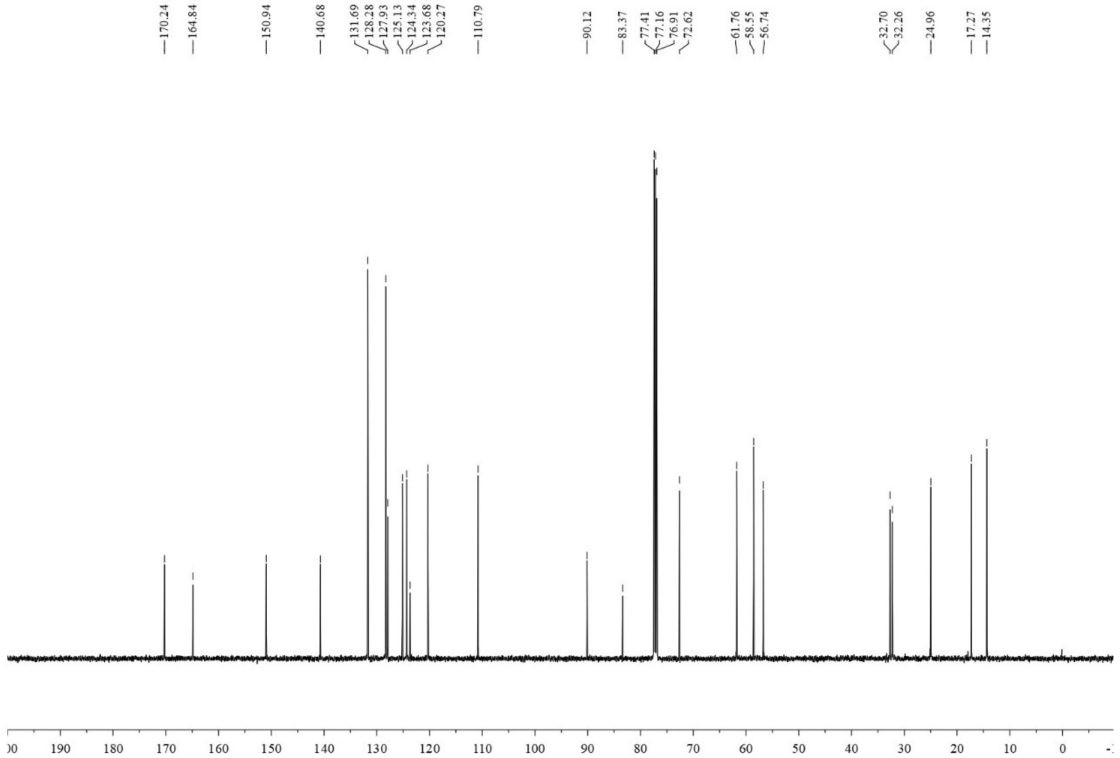

^{1}H NMR of **3l (500 MHz, CDCl$_3$)**

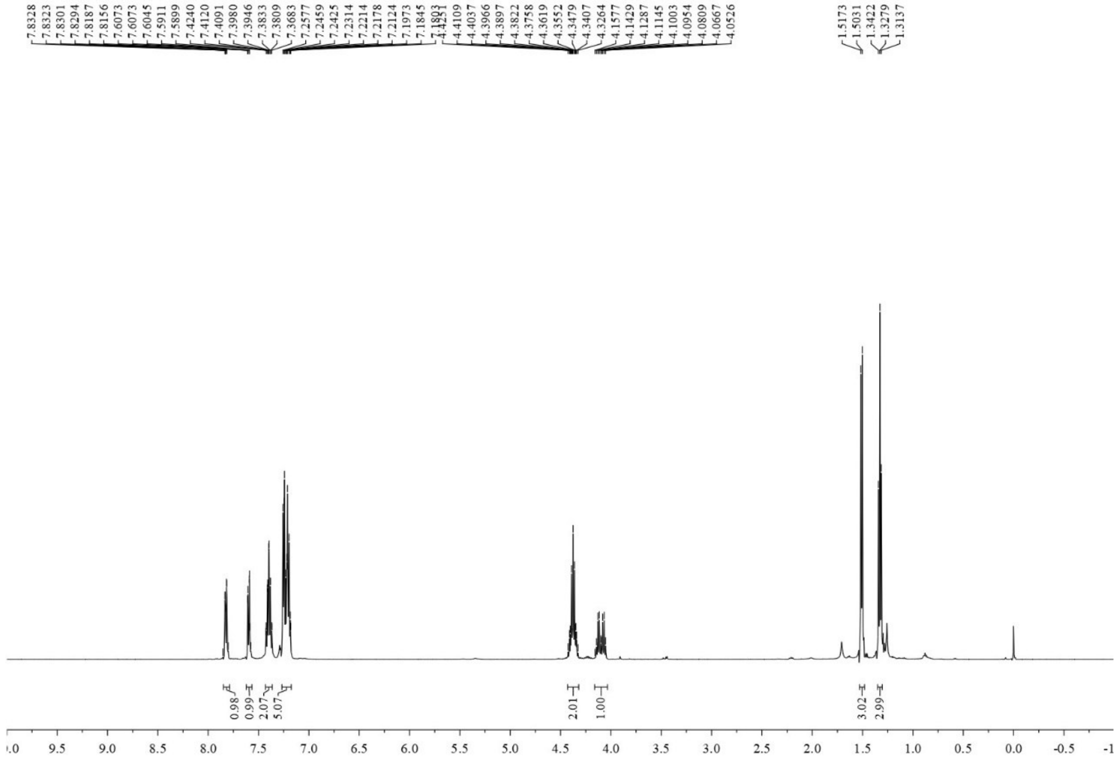

^{13}C NMR of **3l (125 MHz, CDCl$_3$)**

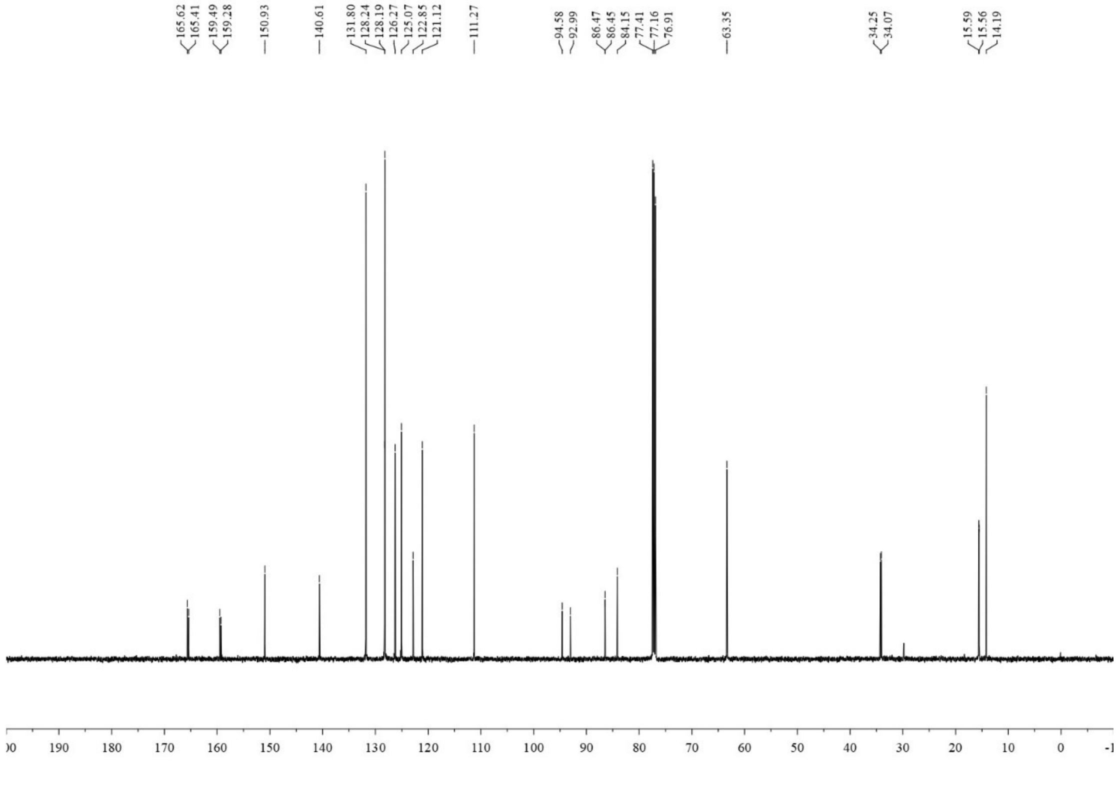

^{19}F NMR of **3l (470 MHz, $CDCl_3$)**

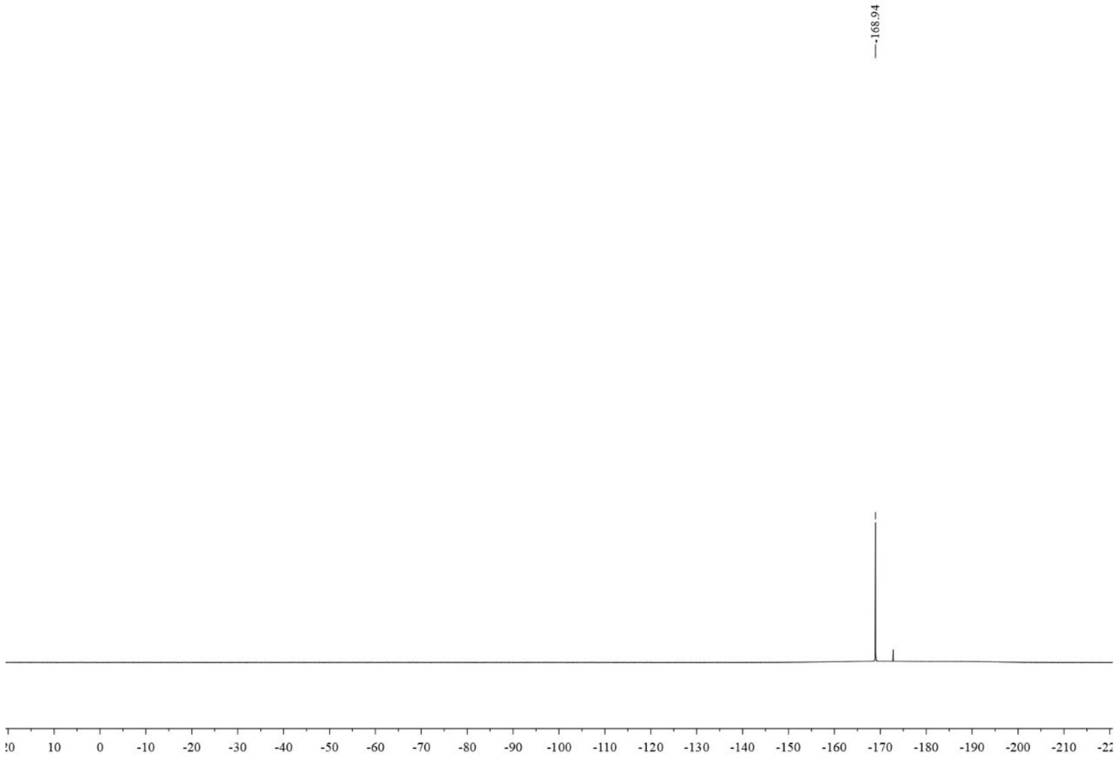

^{1}H NMR of 3m (500 MHz, $CDCl_3$)

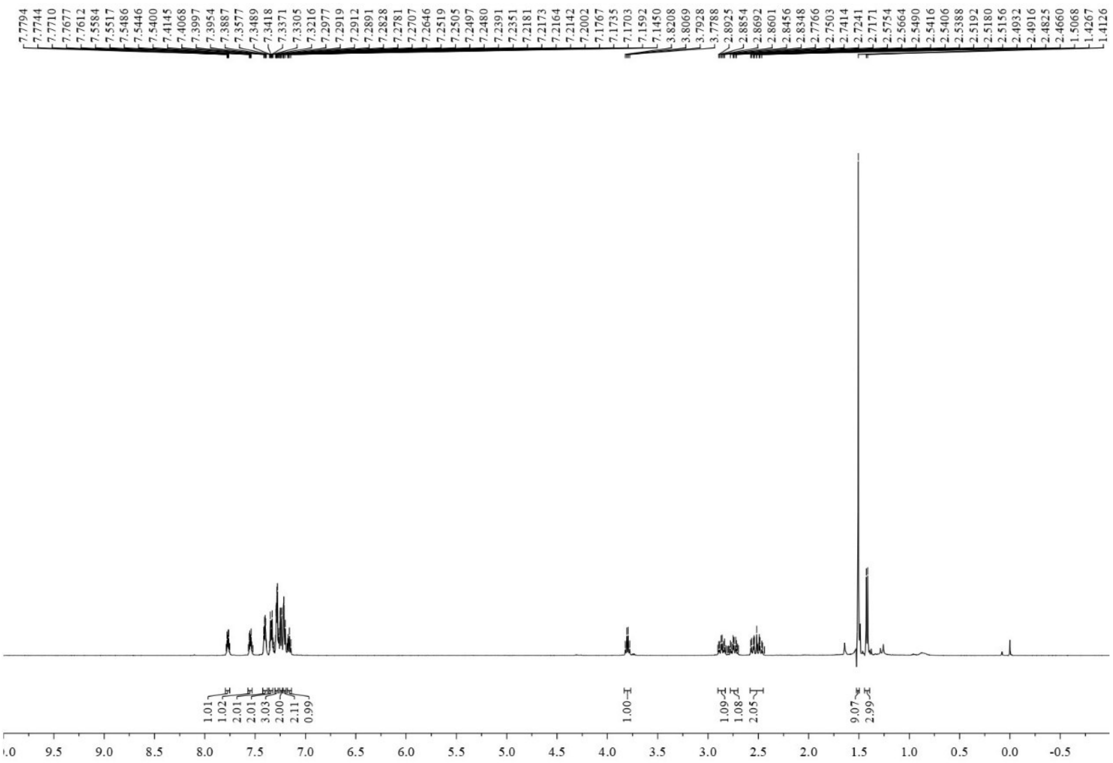

^{13}C NMR of 3m (125 MHz, $CDCl_3$)

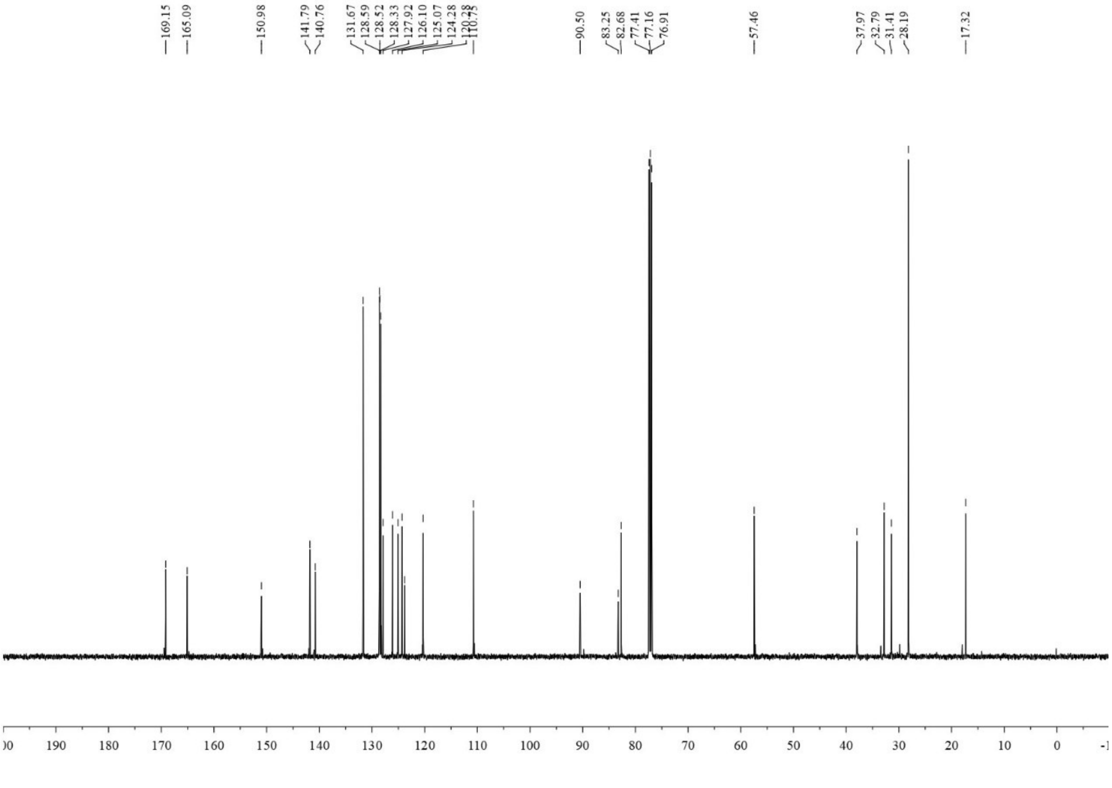

^{1}H NMR of **3n (400 MHz, CDCl$_3$)**

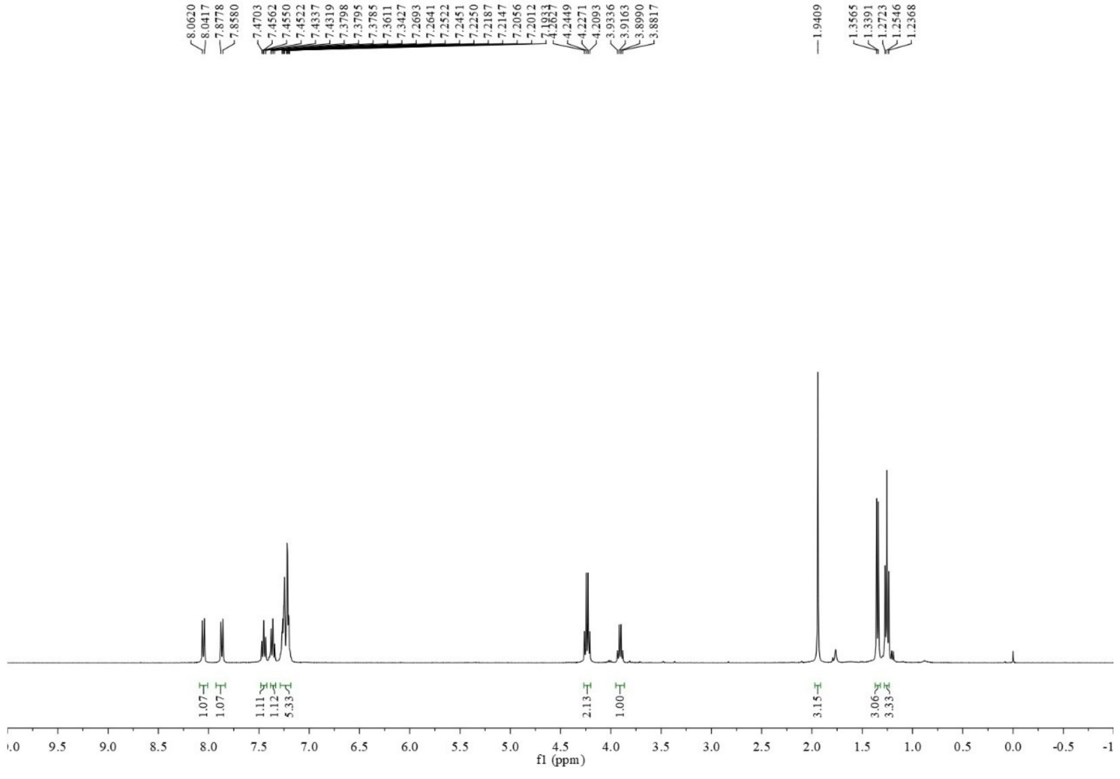

^{13}C NMR of **3n (100 MHz, CDCl$_3$)**

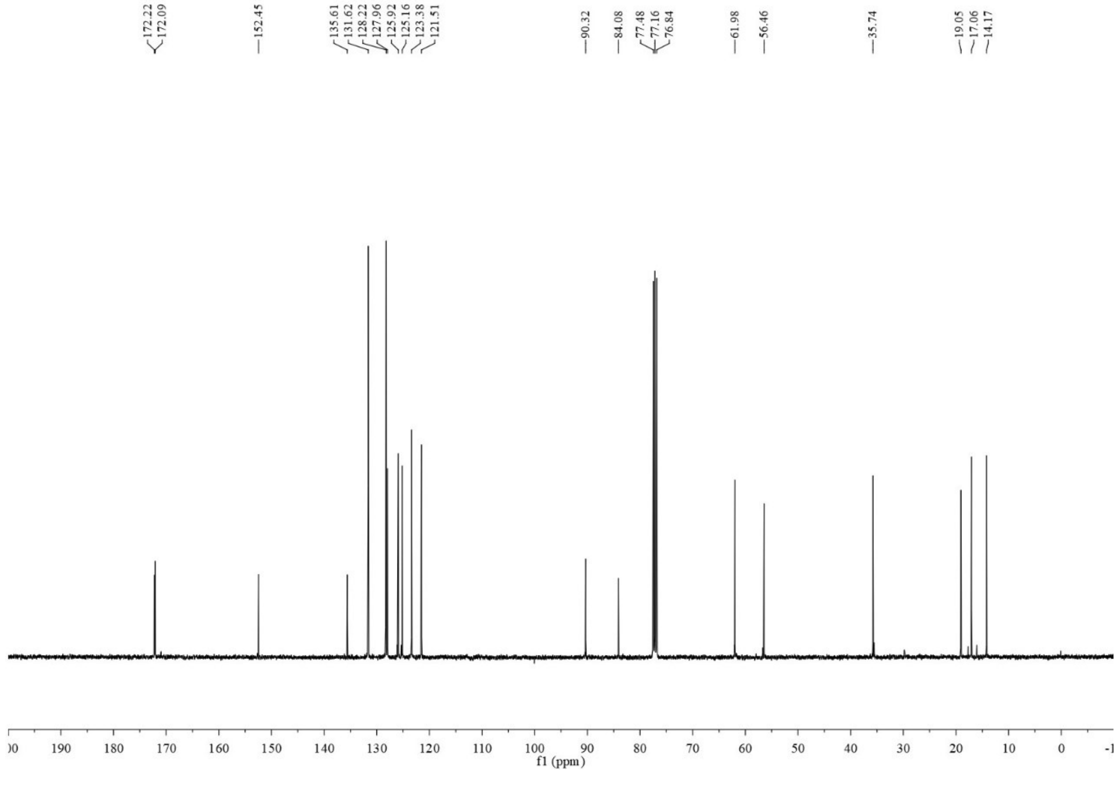

^{1}H NMR of **3o (400 MHz, CDCl$_3$)**

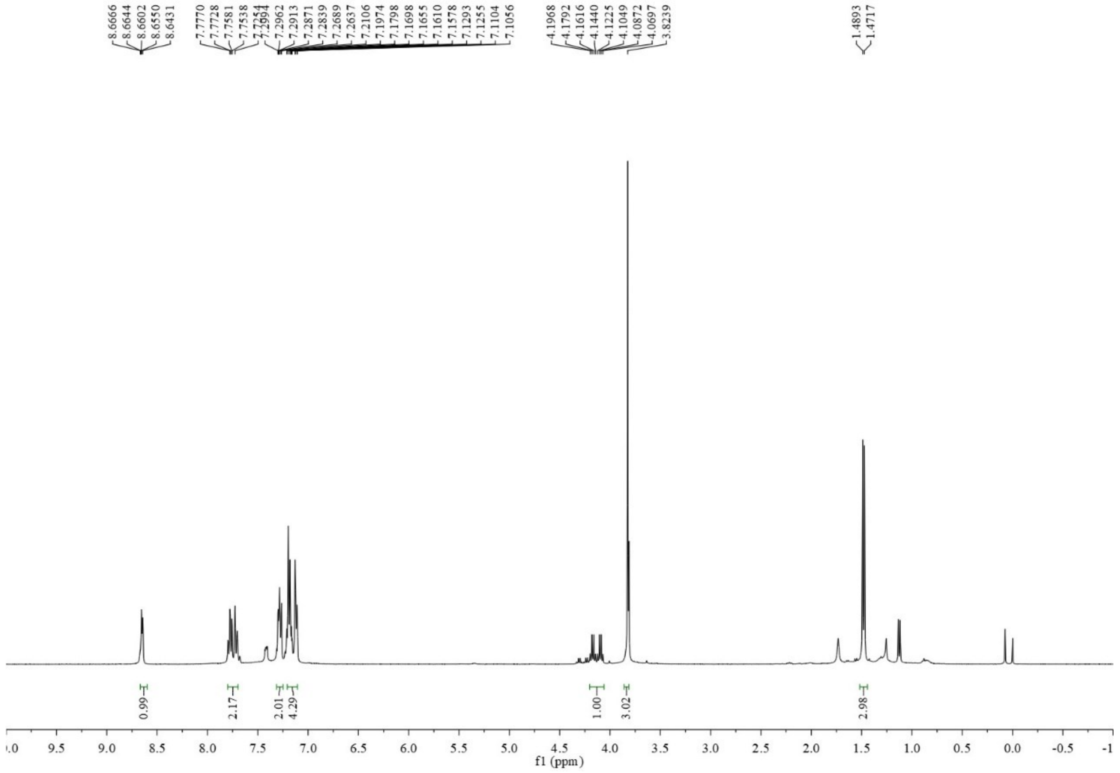

^{13}C NMR of **3o (100 MHz, CDCl$_3$)**

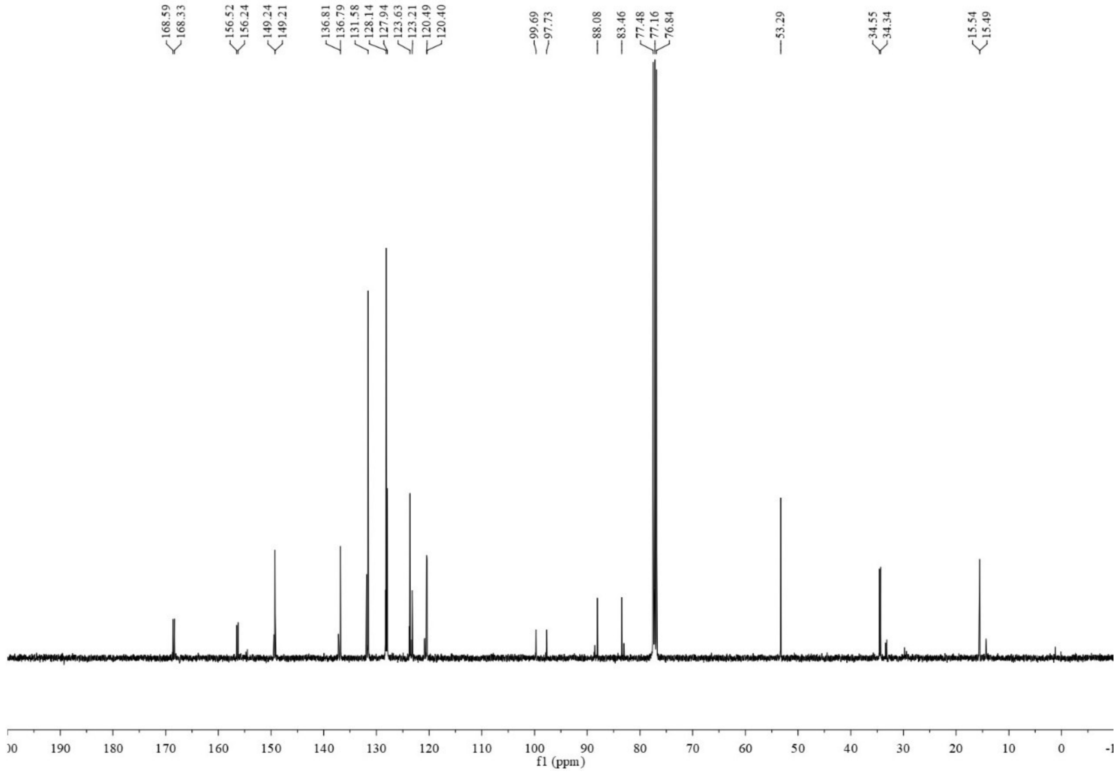

^{19}F NMR of **3o (375 MHz, CDCl$_3$)**

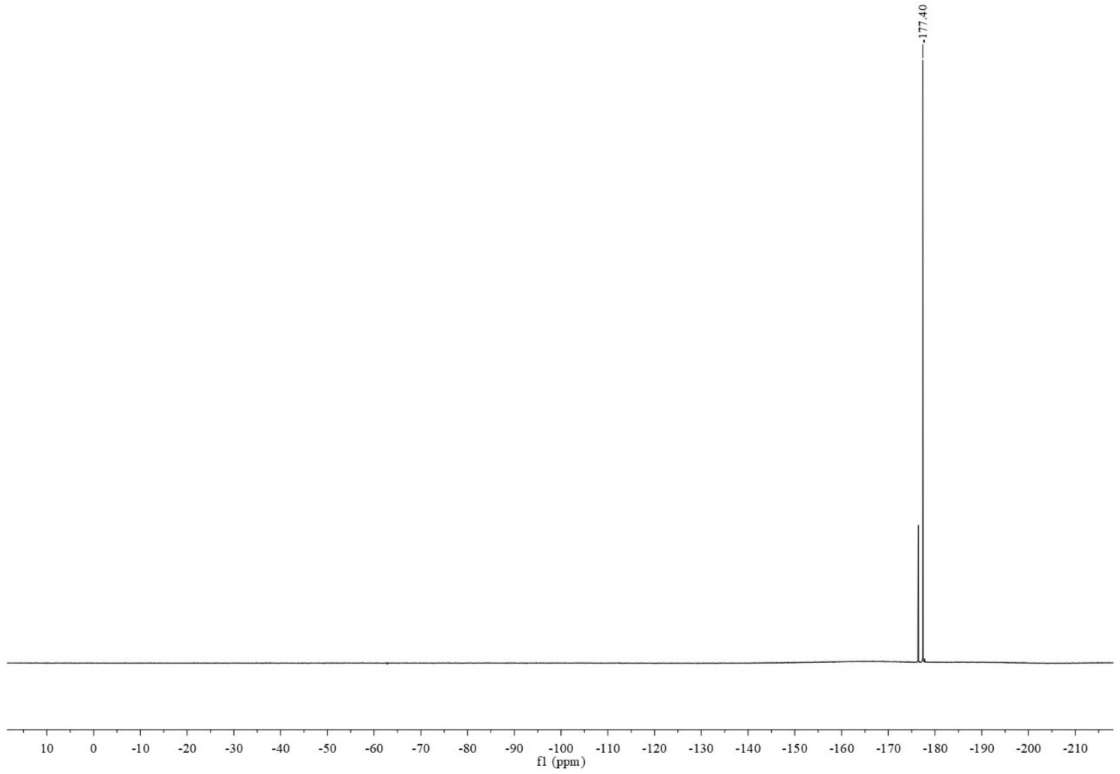

^{1}H NMR of **3p** (500 MHz, $CDCl_3$)

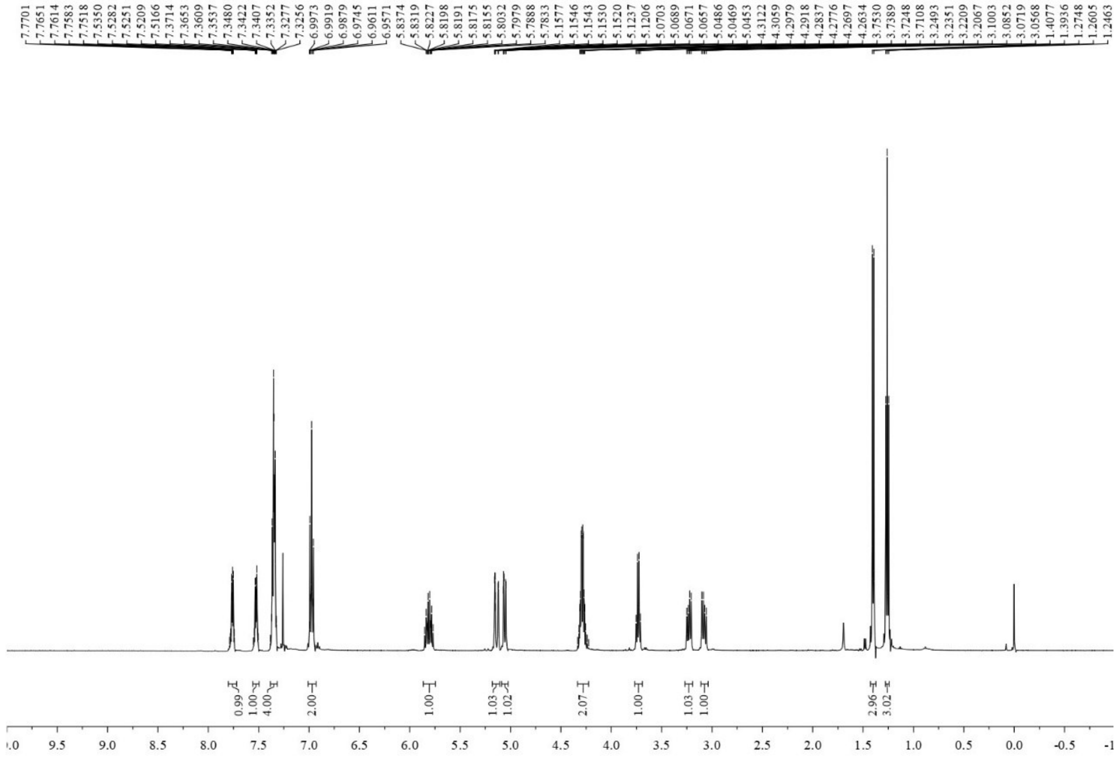

^{13}C NMR of **3p** (125 MHz, $CDCl_3$)

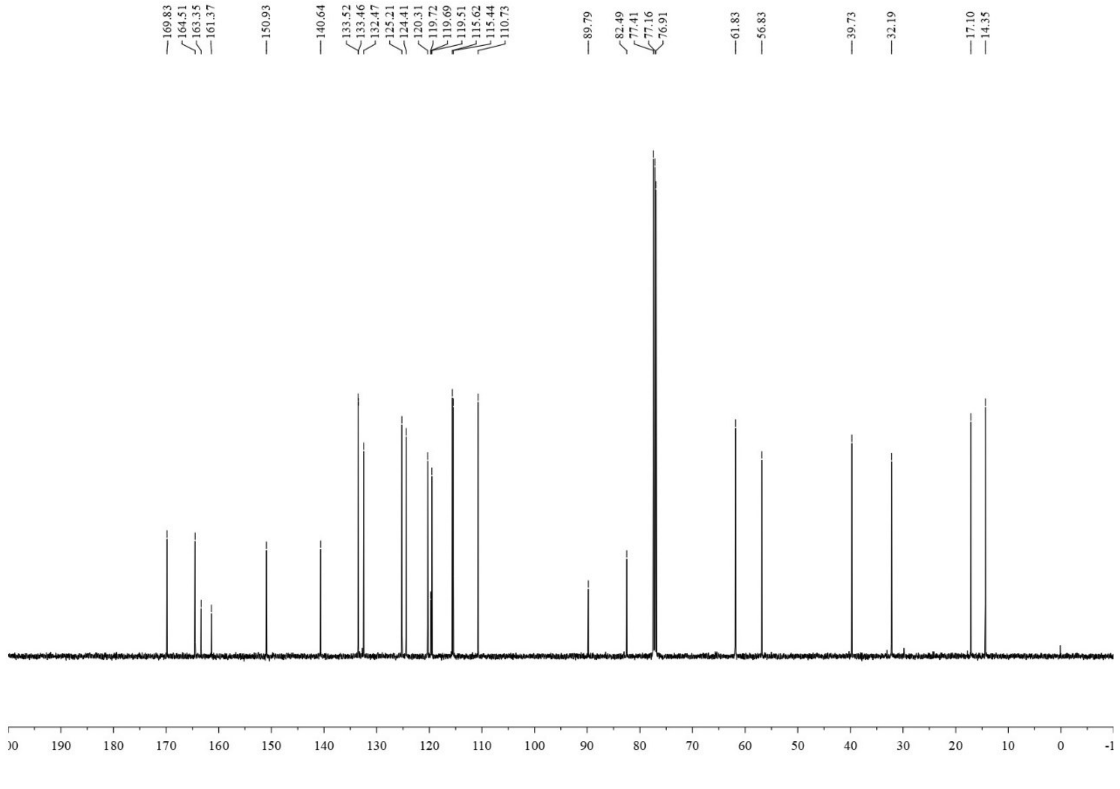

^{19}F NMR of **3p (470 MHz, CDCl$_3$)**

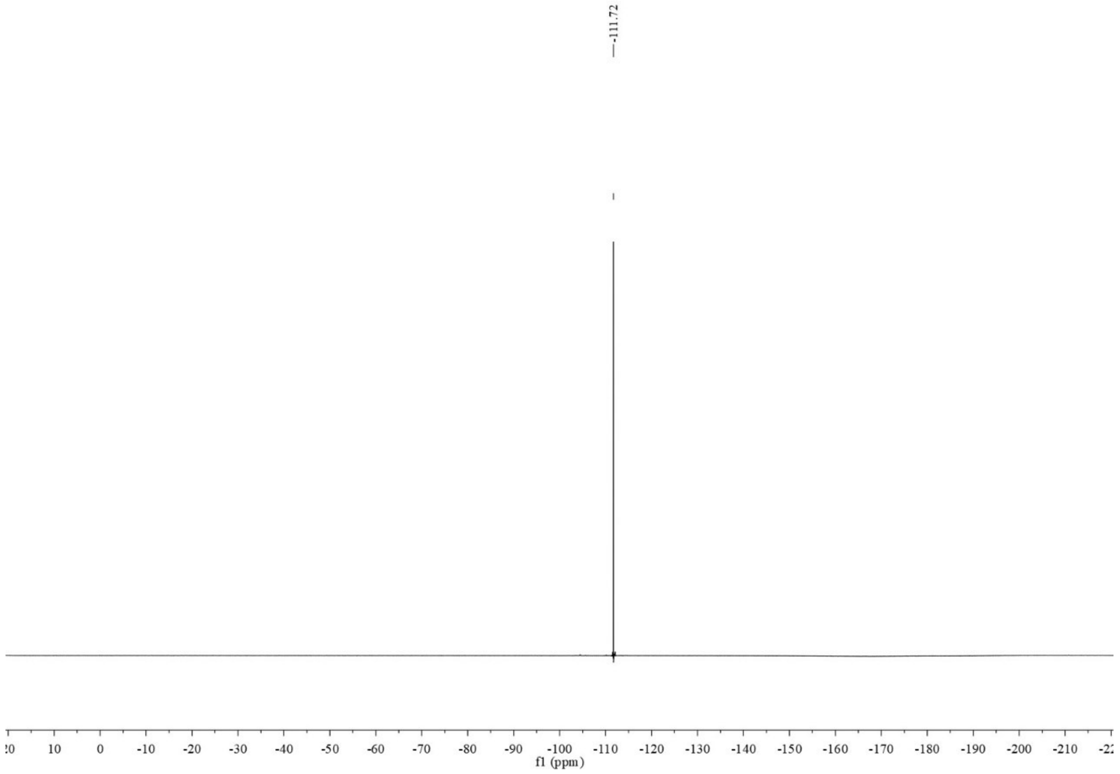

^{1}H NMR of **3q (500 MHz, CDCl$_3$)**

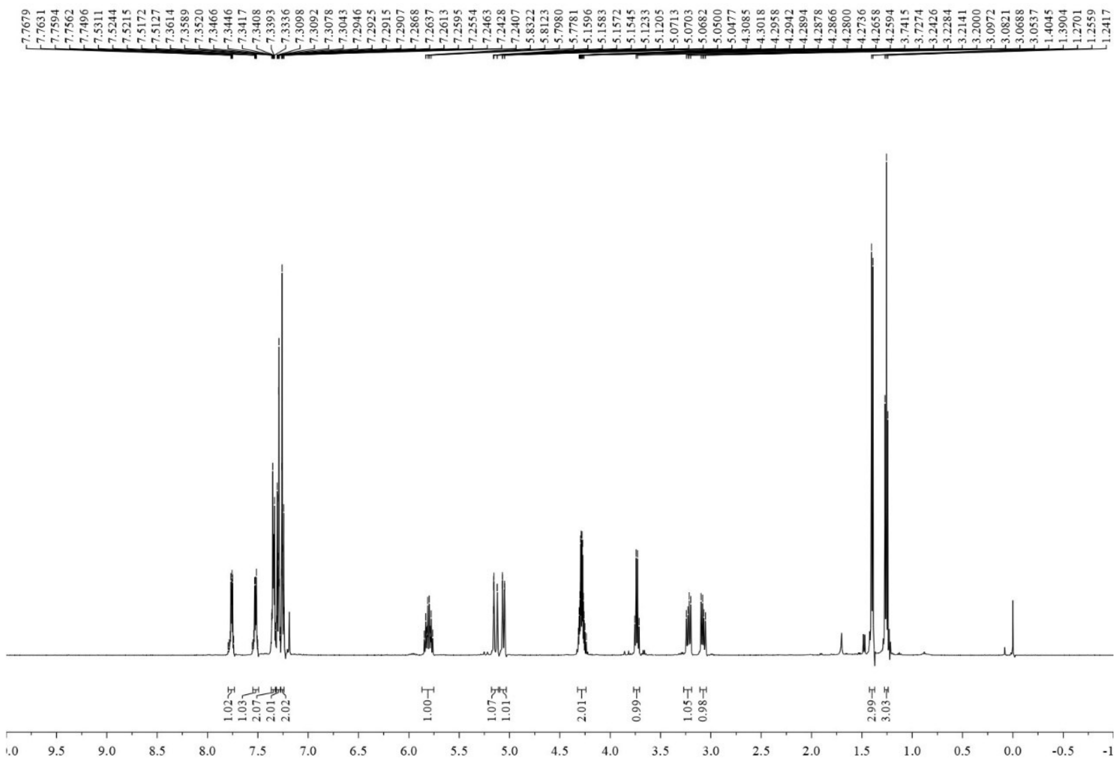

^{13}C NMR of **3q (125 MHz, CDCl$_3$)**

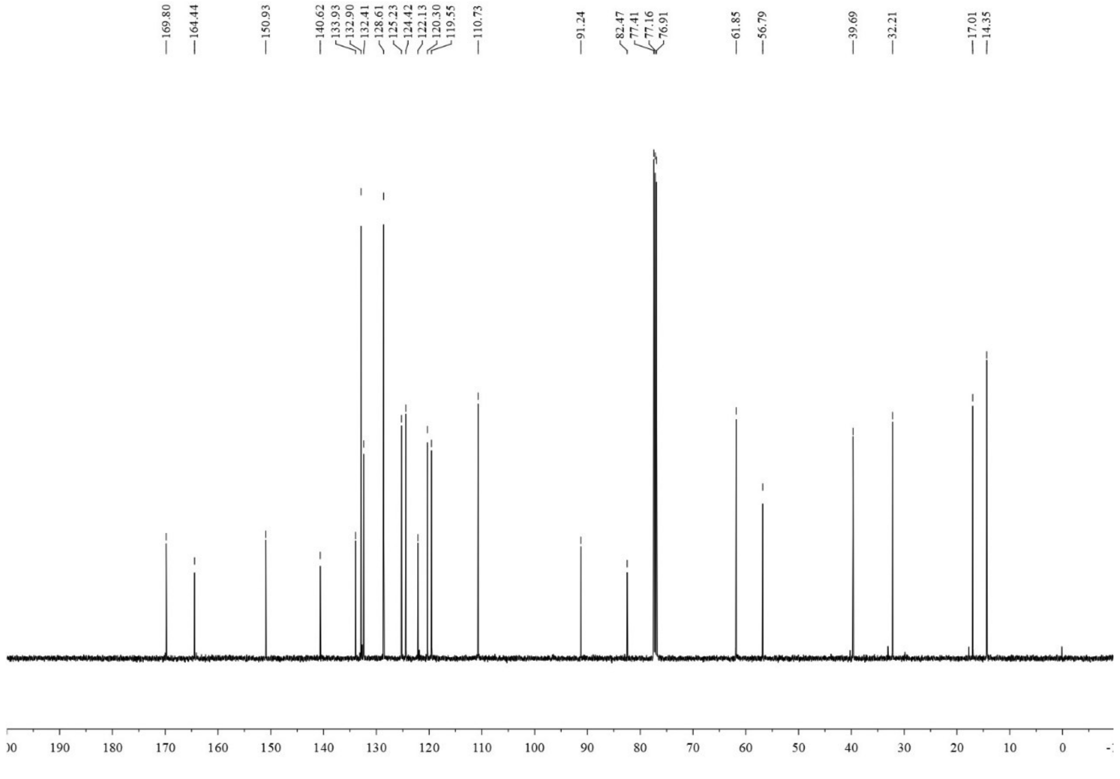

^{1}H NMR of **3r (500 MHz, CDCl$_3$)**

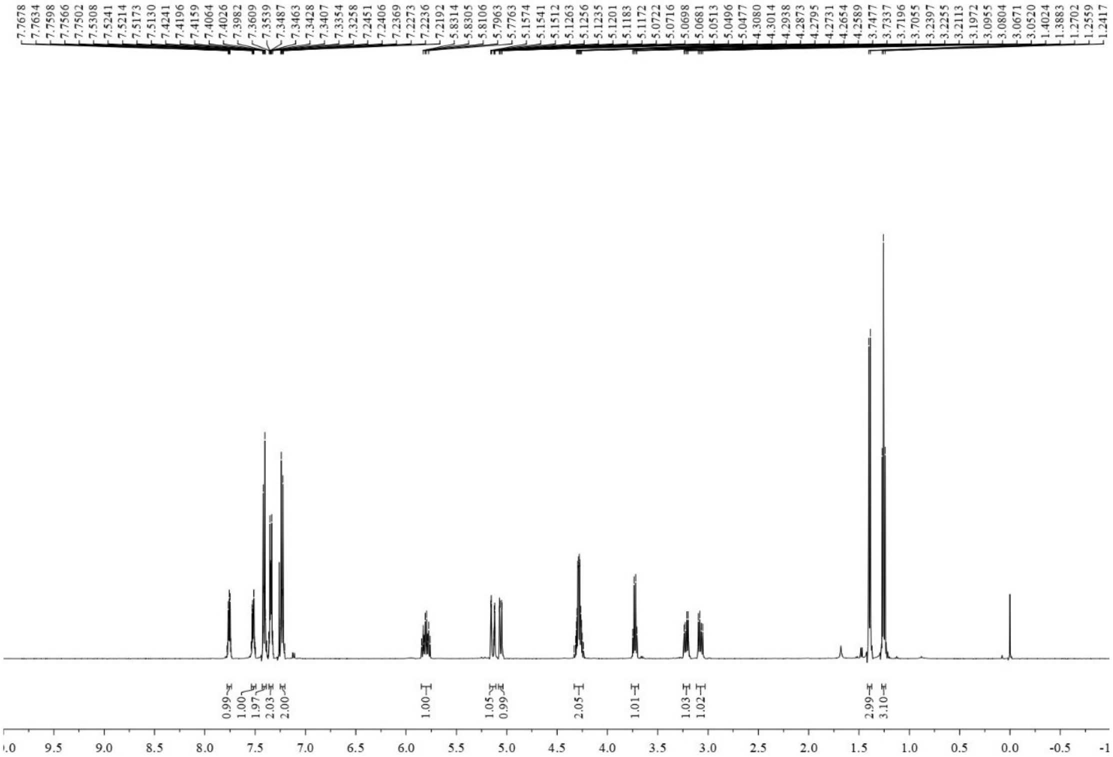

^{13}C NMR of **3r (125 MHz, CDCl$_3$)**

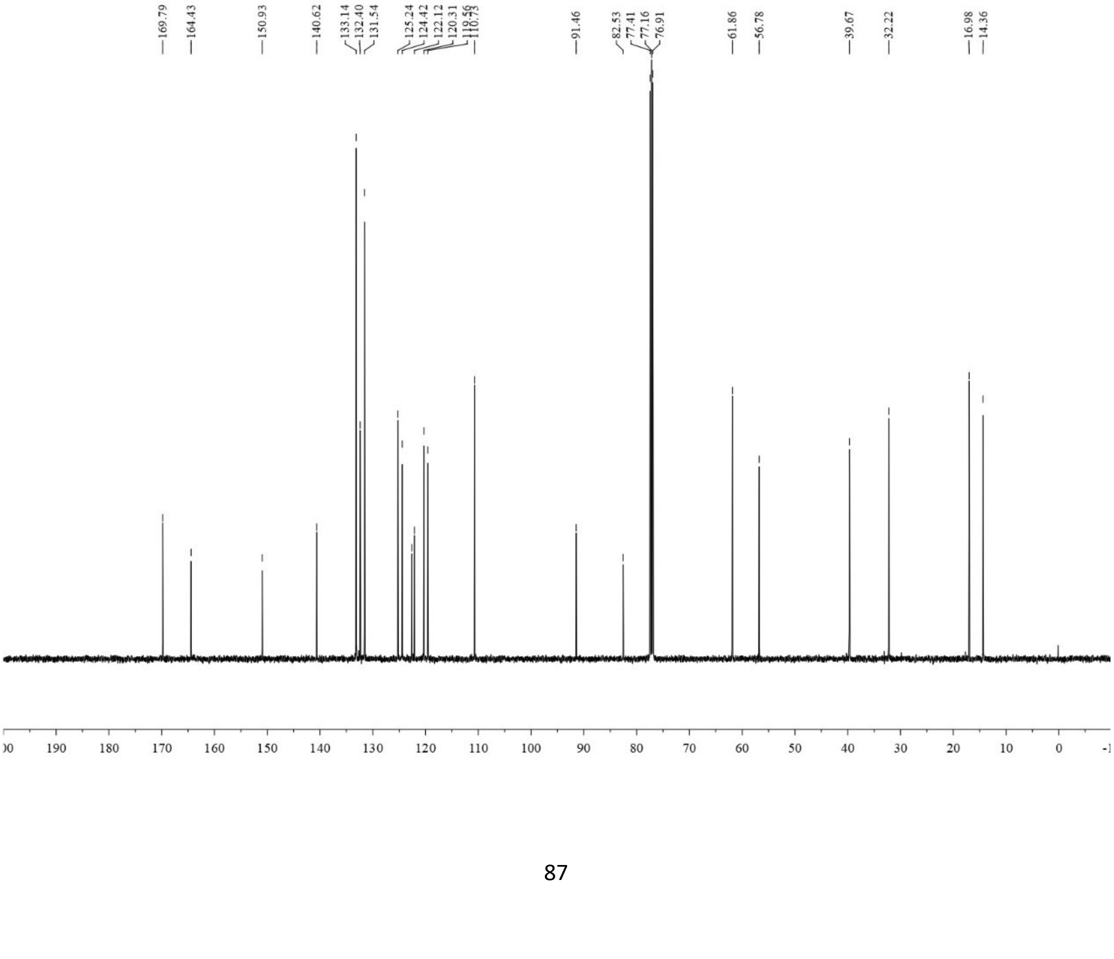

^{1}H NMR of **3s (500 MHz, CDCl$_3$)**

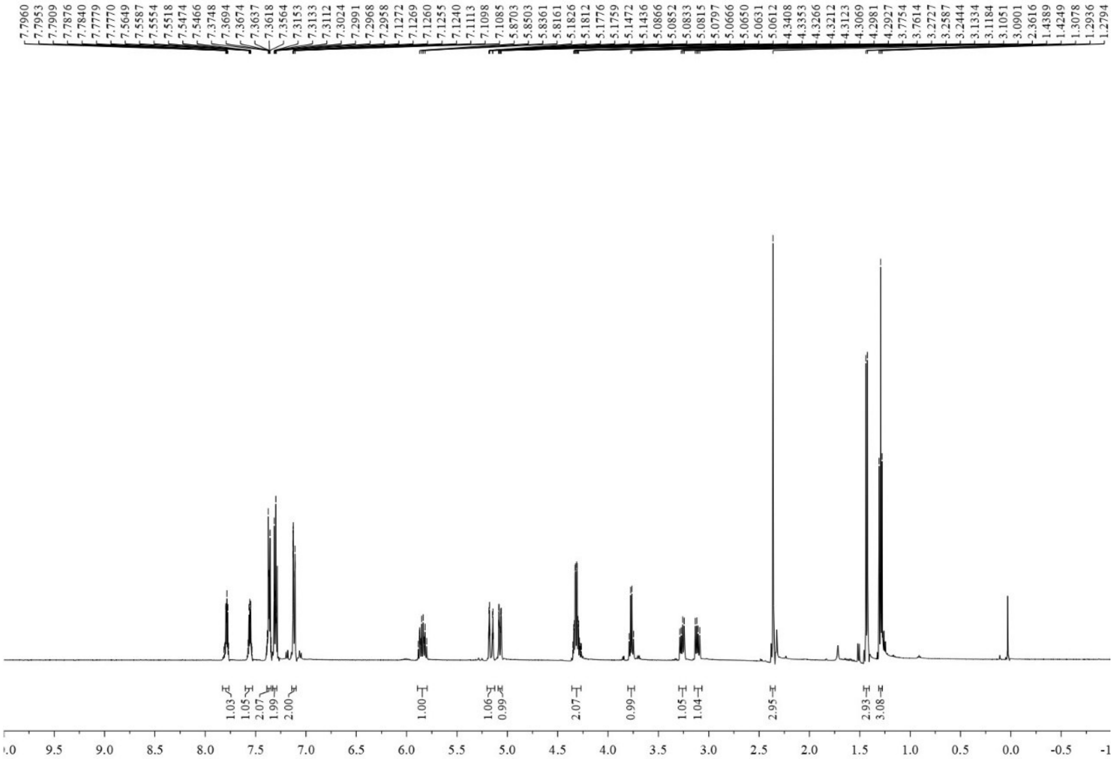

^{13}C NMR of **3s (125 MHz, CDCl$_3$)**

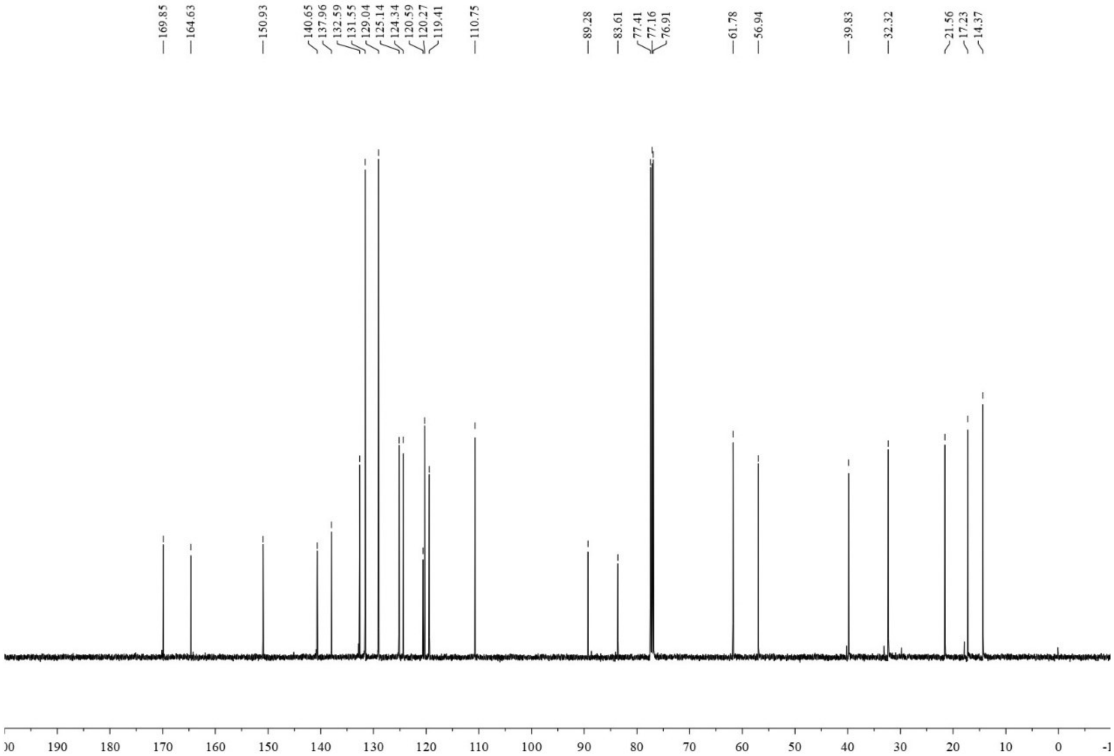

^{1}H NMR of **3t (400 MHz, $CDCl_3$)**

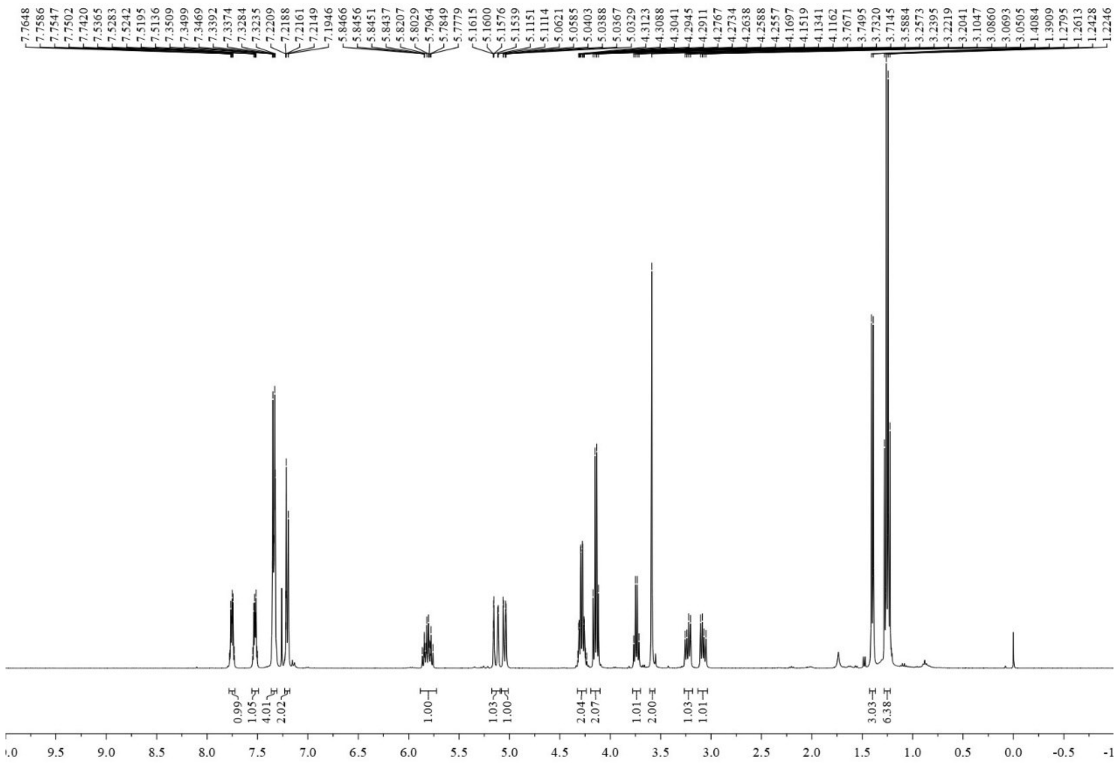

^{13}C NMR of **3t (100 MHz, $CDCl_3$)**

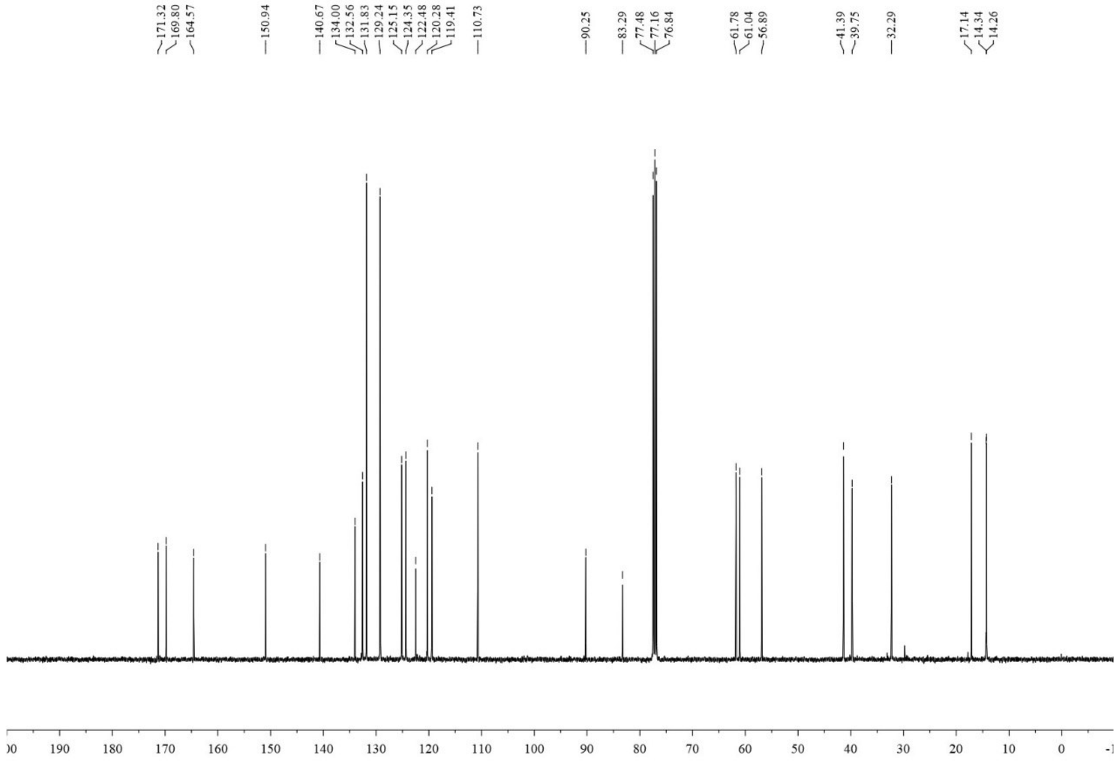

^{1}H NMR of 3u (500 MHz, $CDCl_3$)

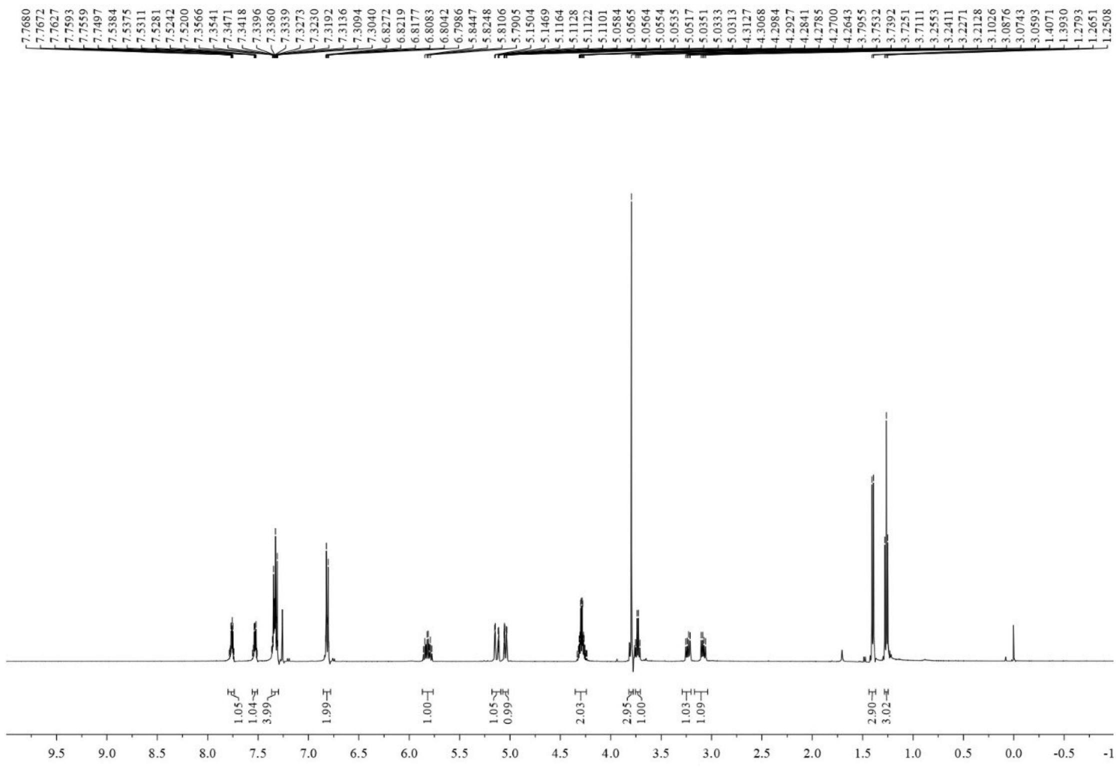

^{13}C NMR of 3u (125 MHz, $CDCl_3$)

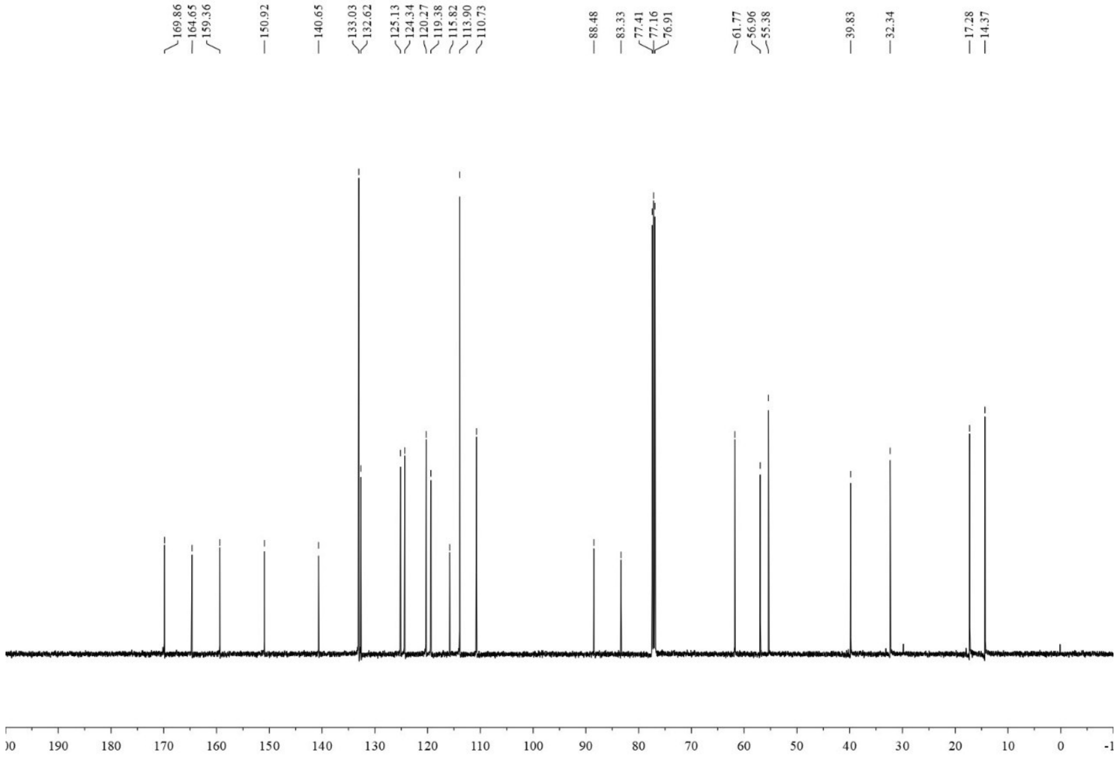

^{1}H NMR of **3v (500 MHz, $CDCl_3$)**

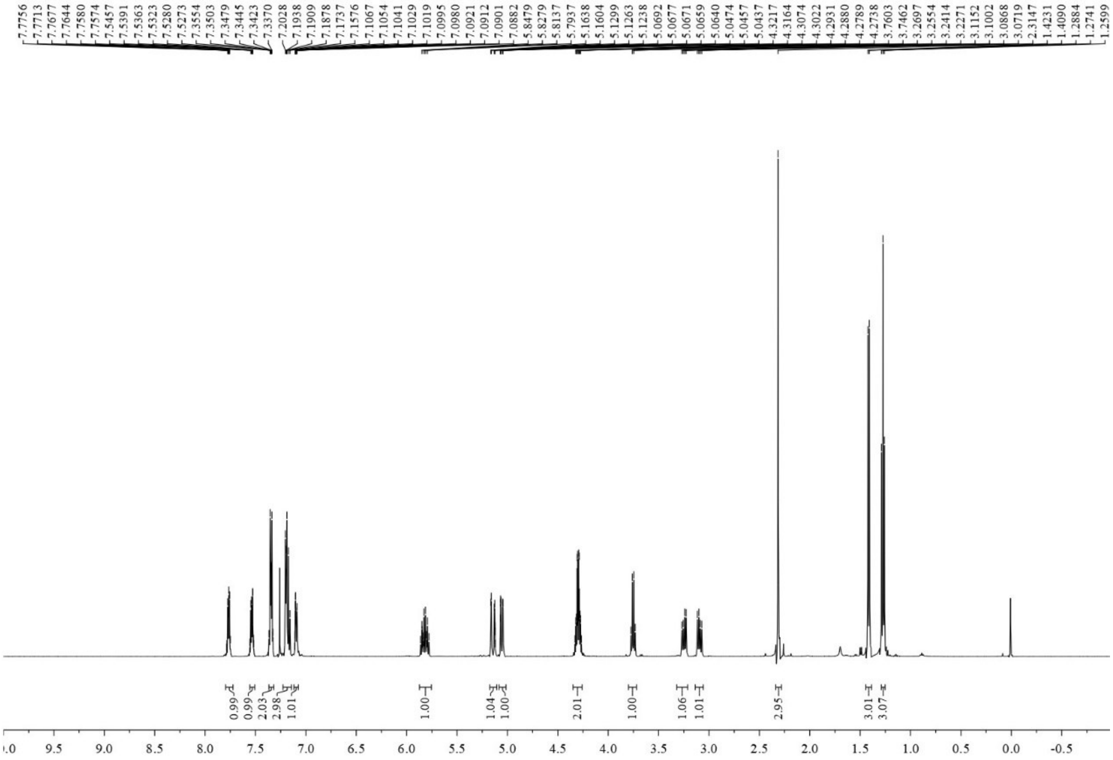

^{13}C NMR of **3v (125 MHz, $CDCl_3$)**

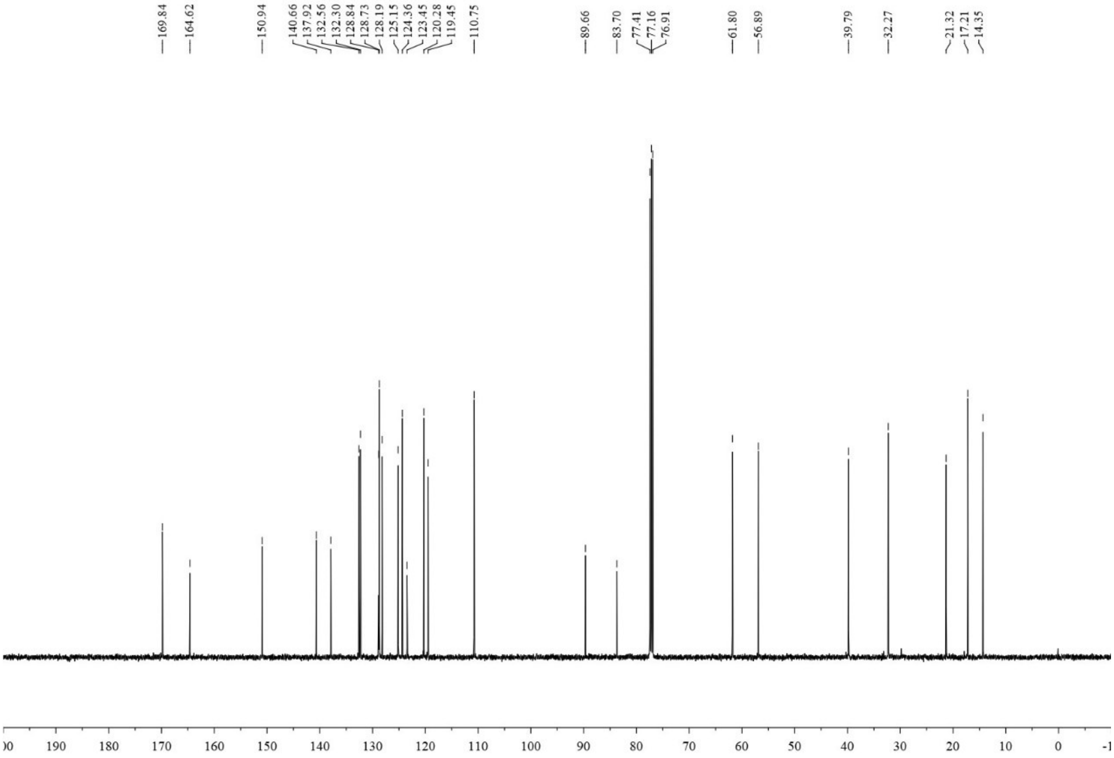

^{1}H NMR of **3w (500 MHz, CDCl$_3$)**

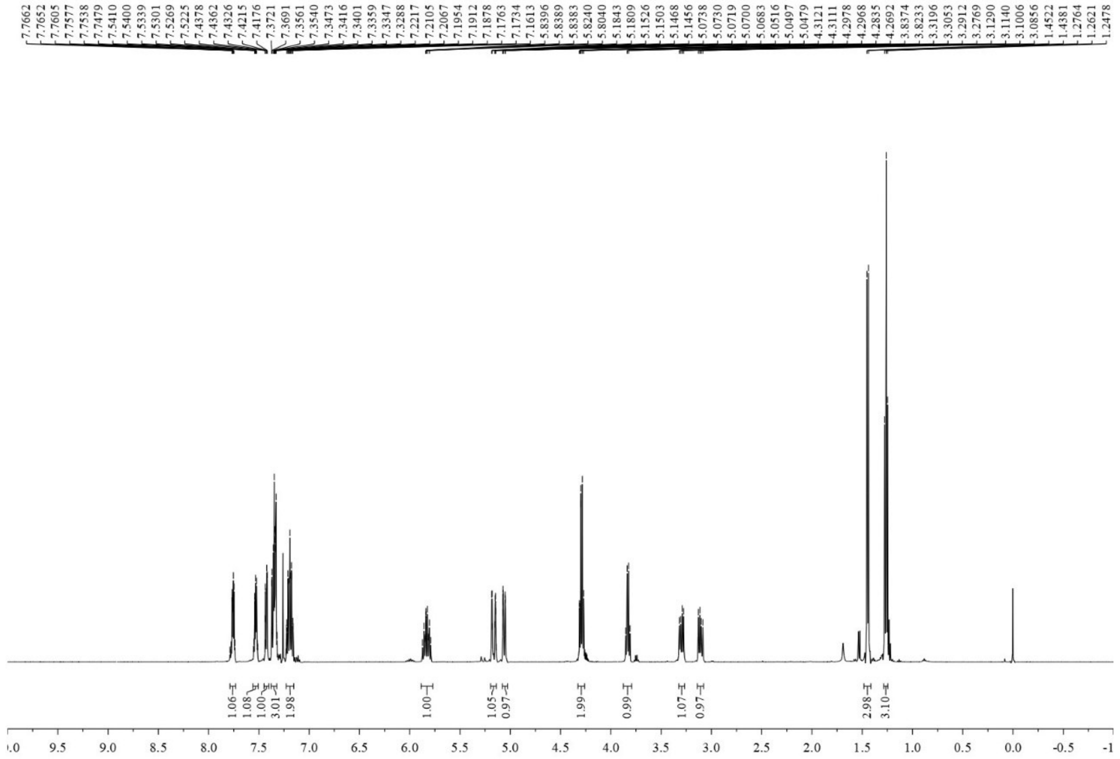

^{13}C NMR of **3w (125 MHz, CDCl$_3$)**

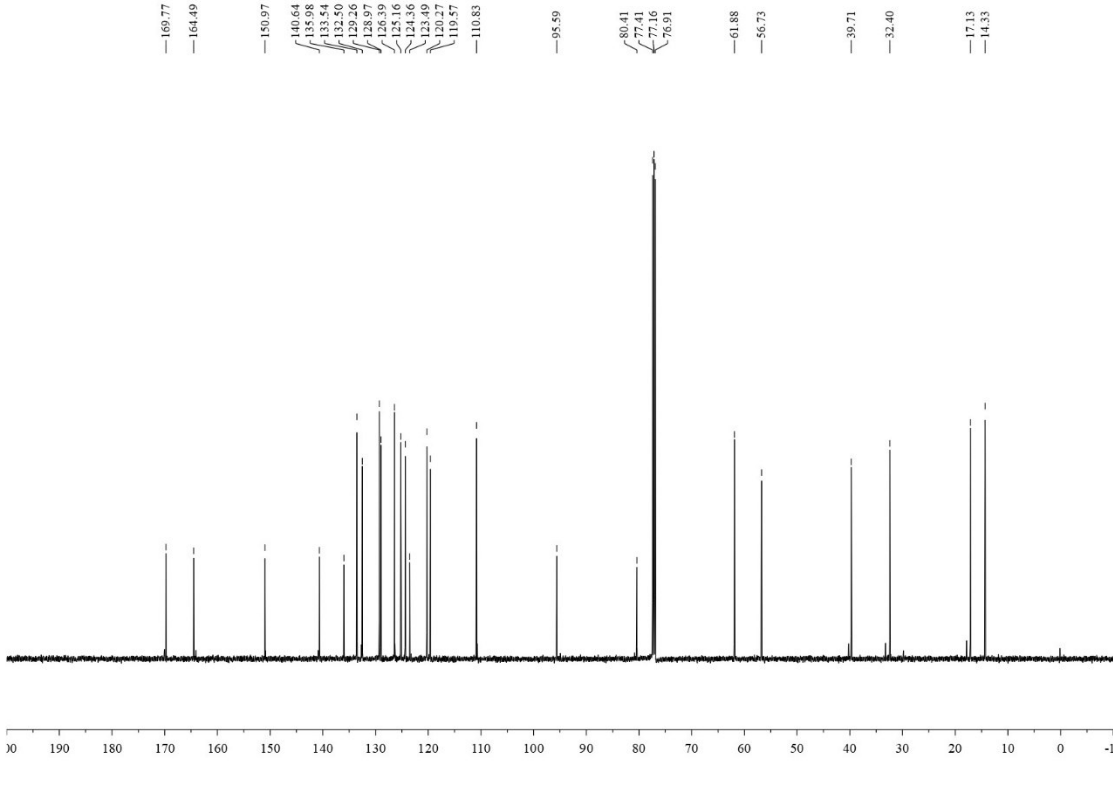

^{1}H NMR of 3x (500 MHz, $CDCl_3$)

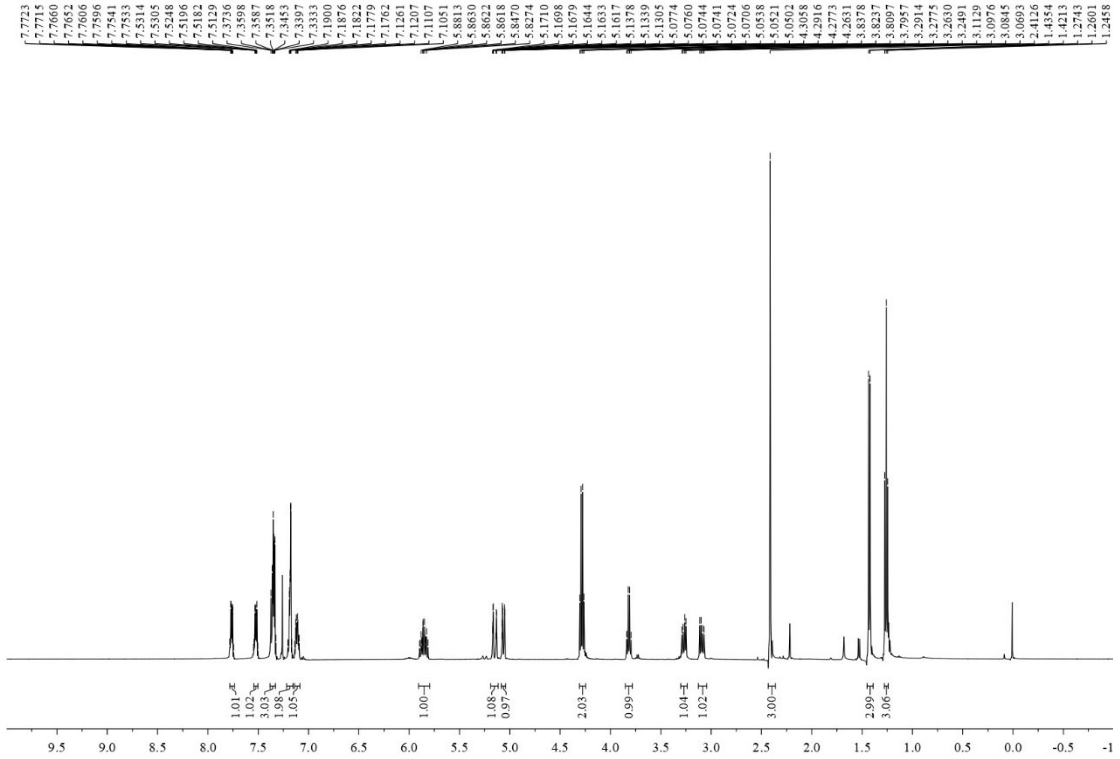

^{13}C NMR of 3x (125 MHz, $CDCl_3$)

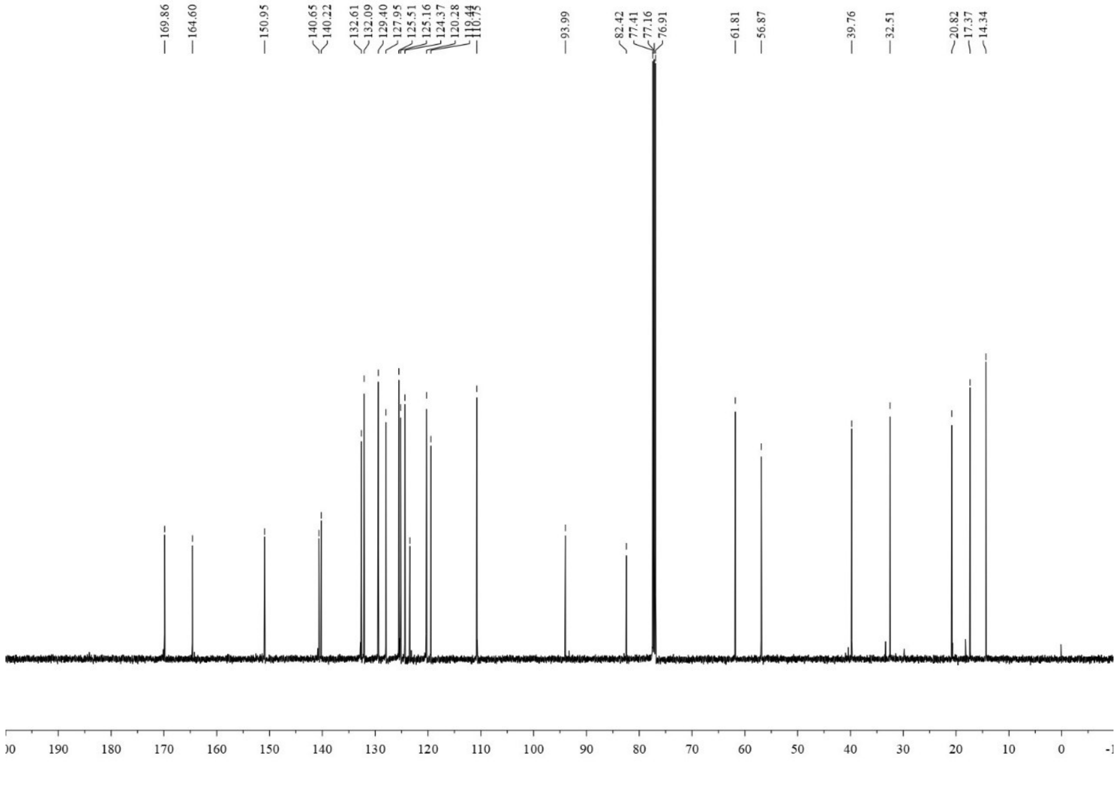

^{1}H NMR of **3y (500 MHz, CDCl$_3$)**

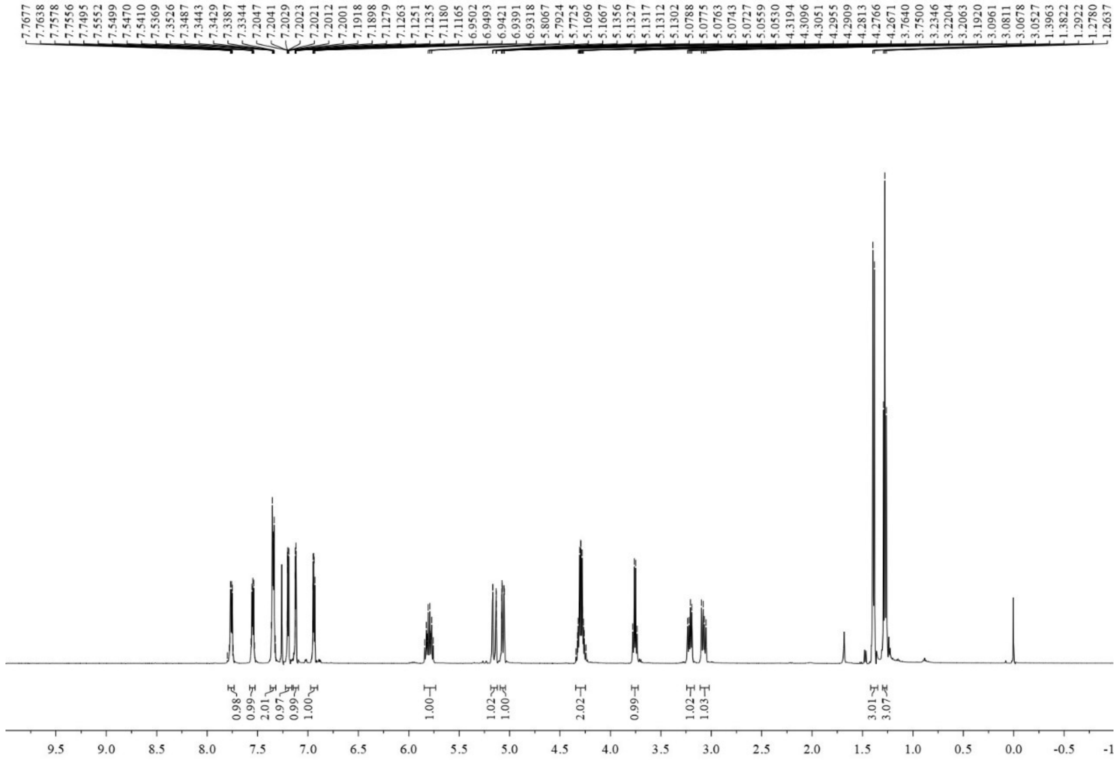

^{13}C NMR of **3y (125 MHz, CDCl$_3$)**

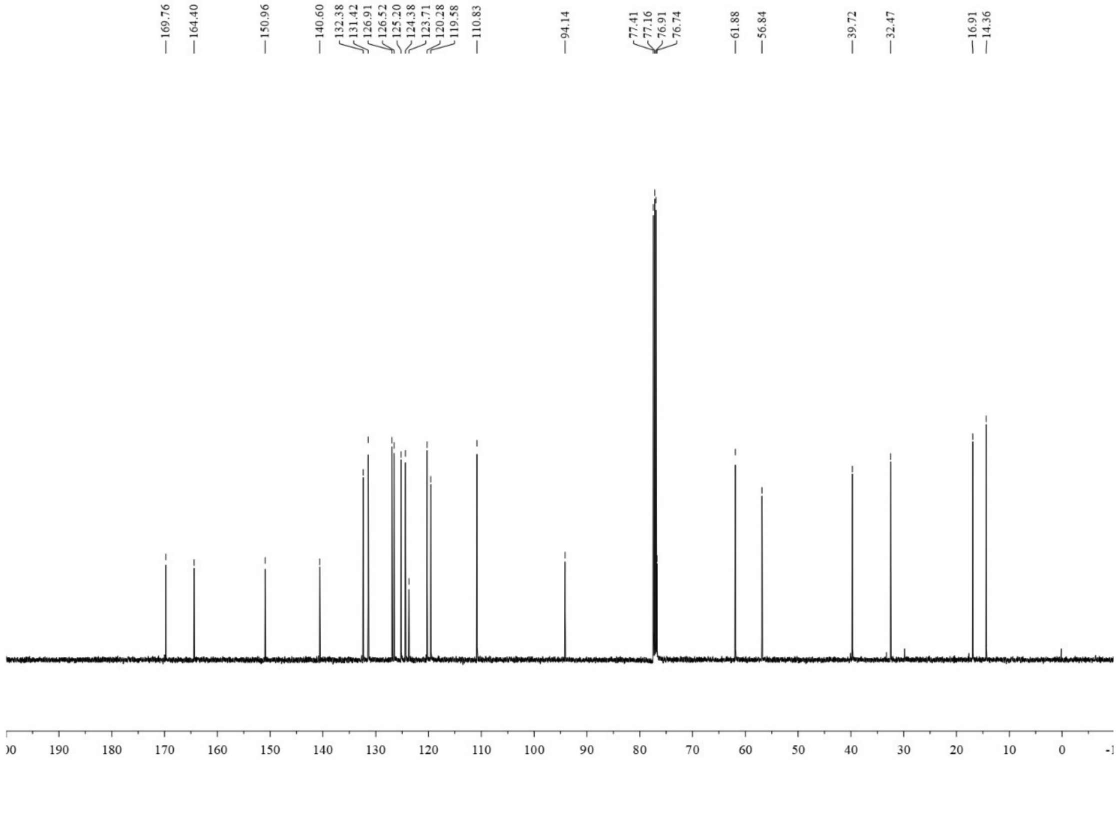

^{1}H NMR of **3z (500 MHz, CDCl$_3$)**

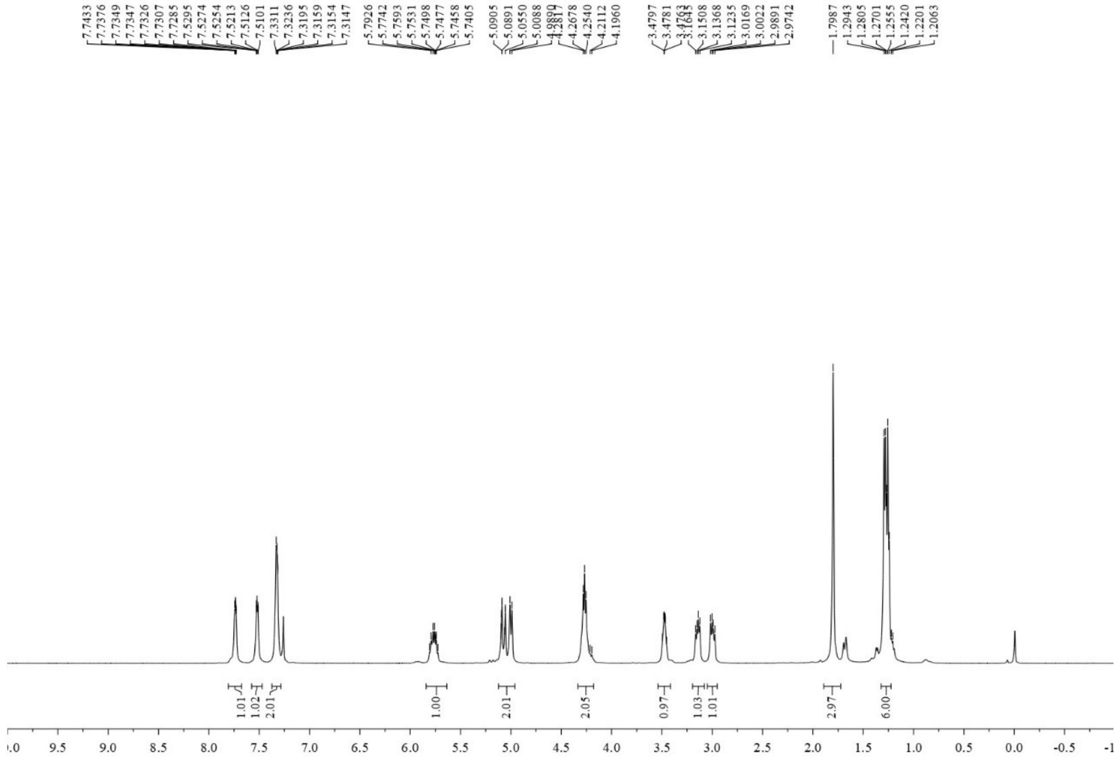

^{13}C NMR of **3z (125 MHz, CDCl$_3$)**

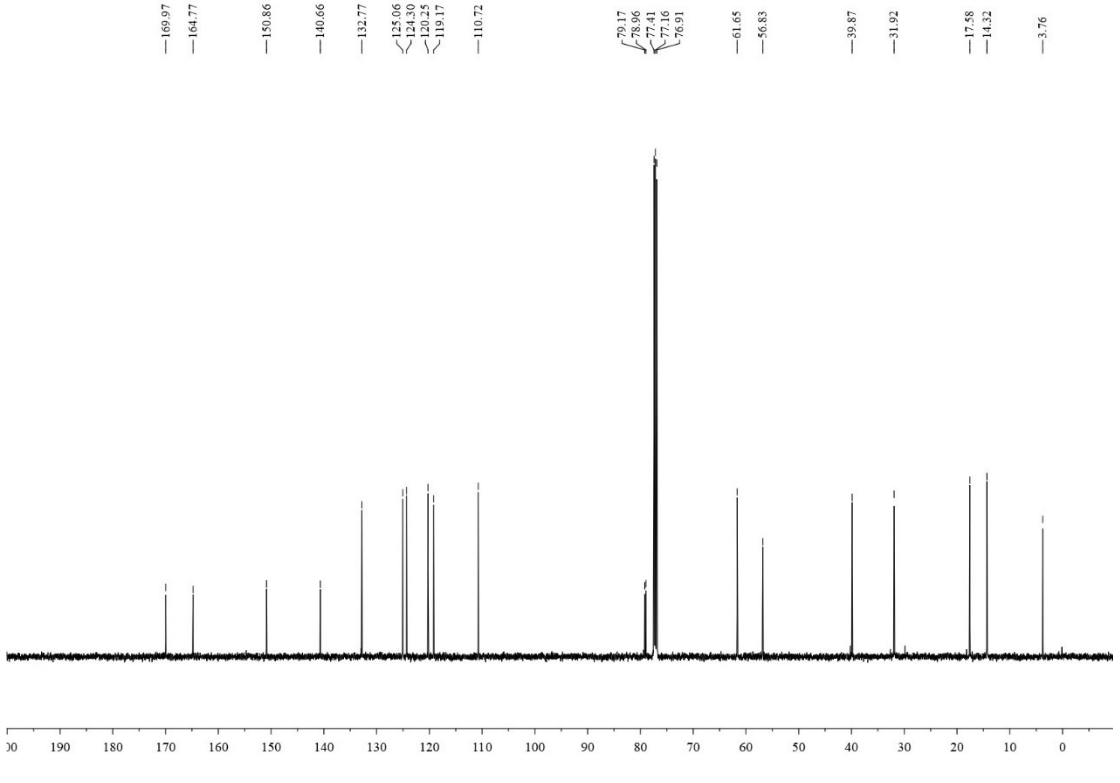

^{1}H NMR of (*S,R*)-**3aa (500 MHz, CDCl$_3$)**

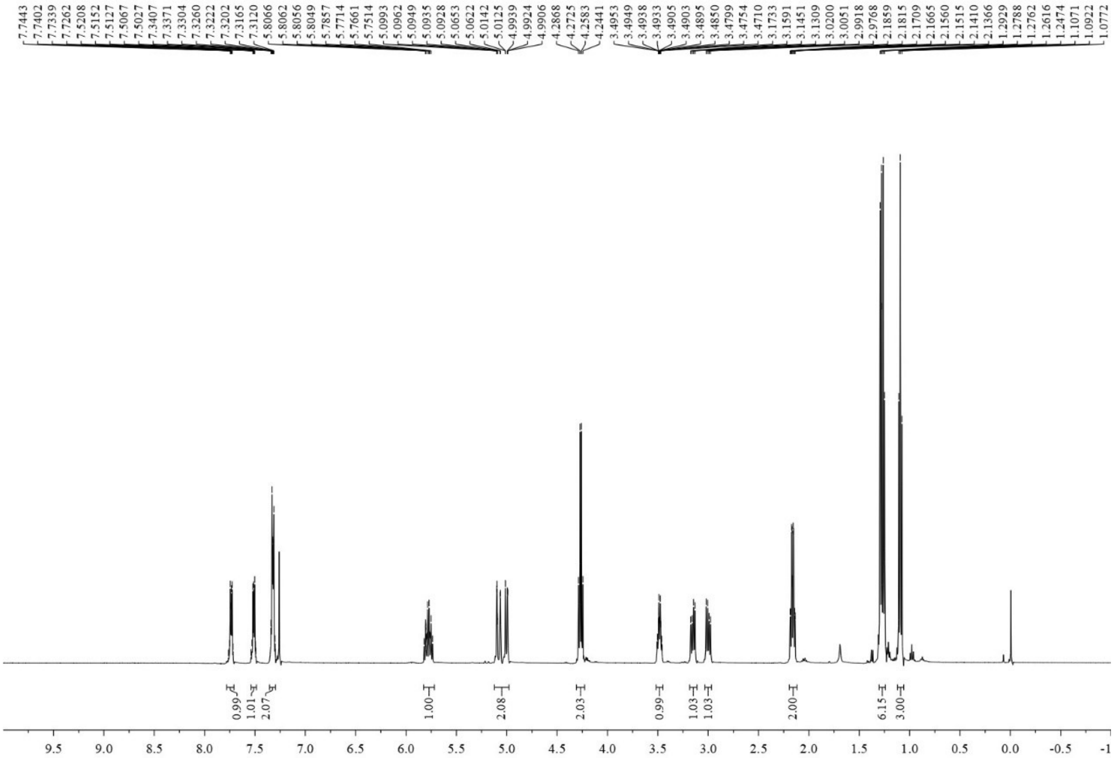

^{13}C NMR of (*S,R*)-**3aa (125 MHz, CDCl$_3$)**

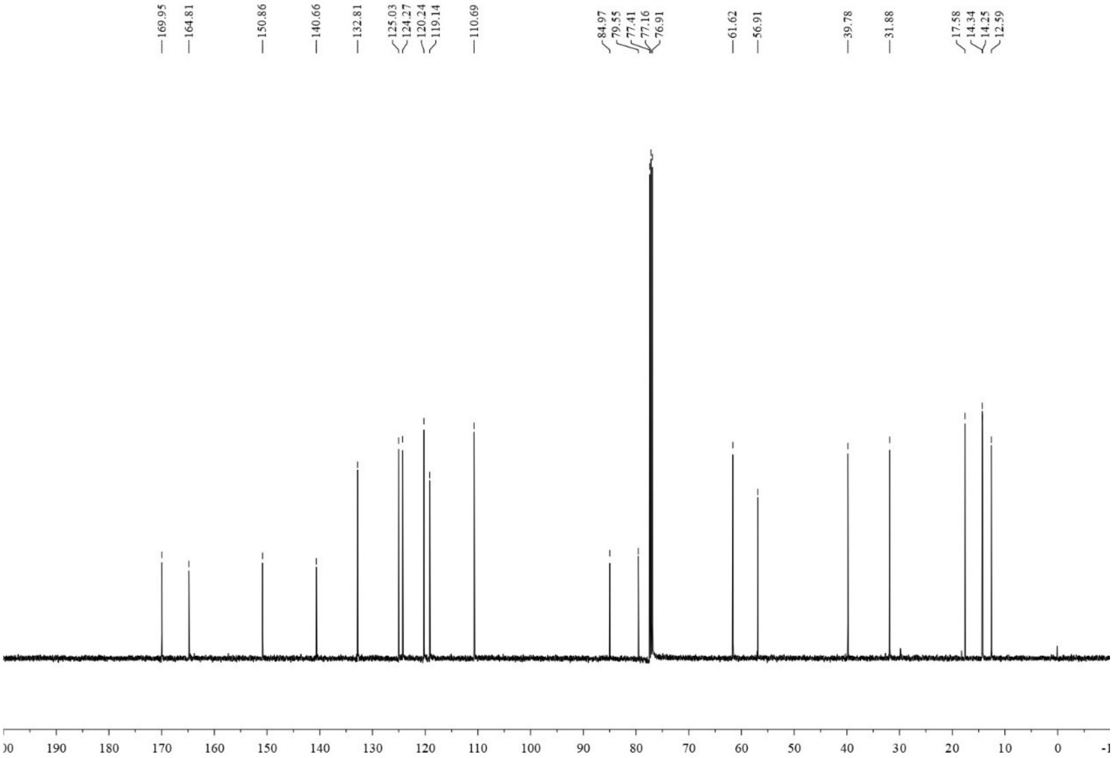

^{1}H NMR of (*R,S*)**-3aa (400 MHz, CDCl$_3$)**

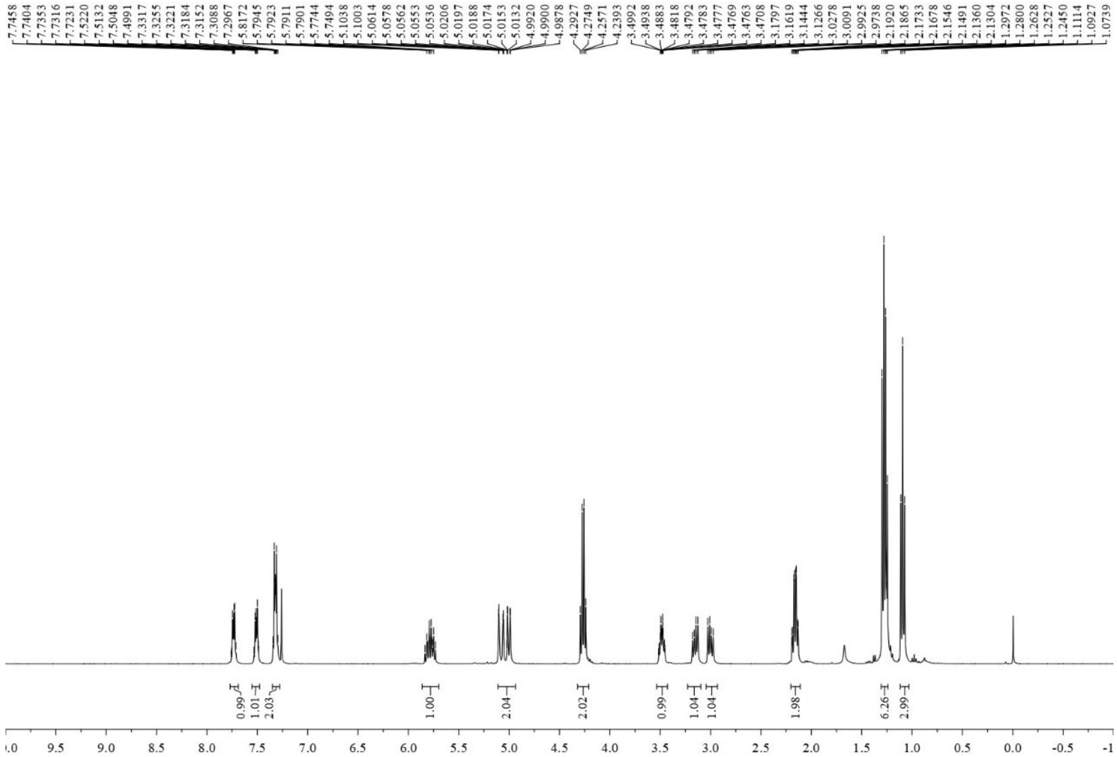

^{13}C NMR of (*R,S*)**-3aa (100 MHz, CDCl$_3$)**

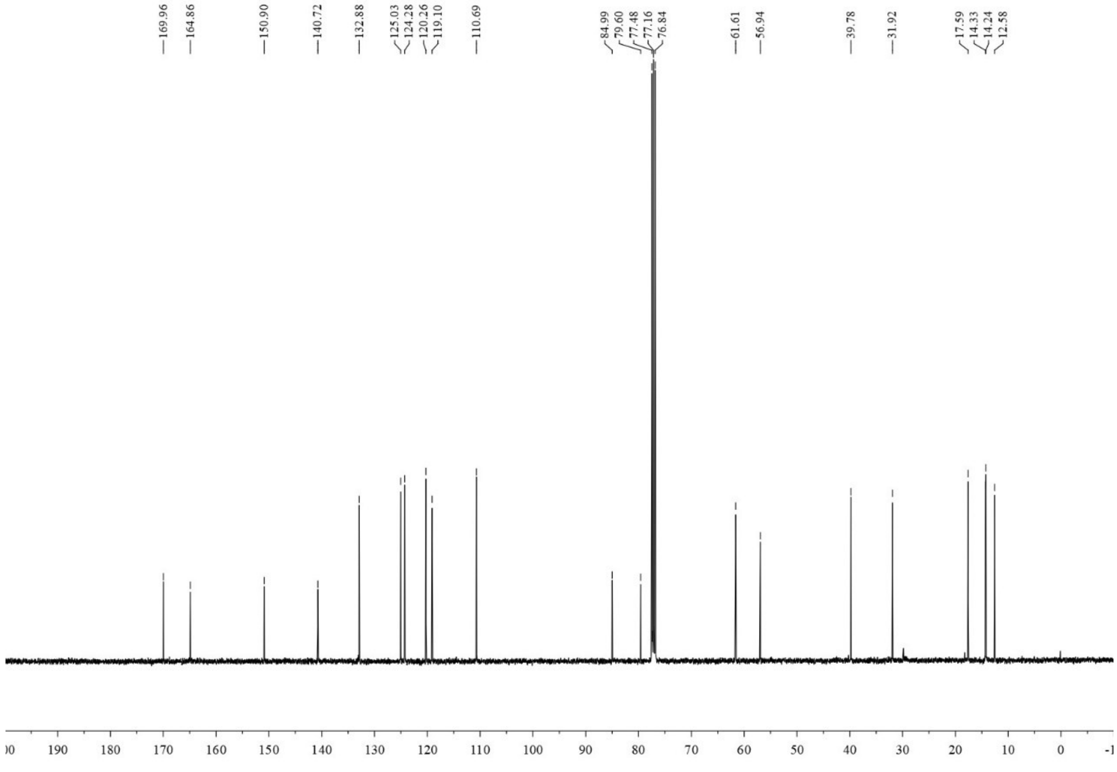

^{1}H NMR of (*R,R*)-**3aa (500 MHz, CDCl$_3$)**

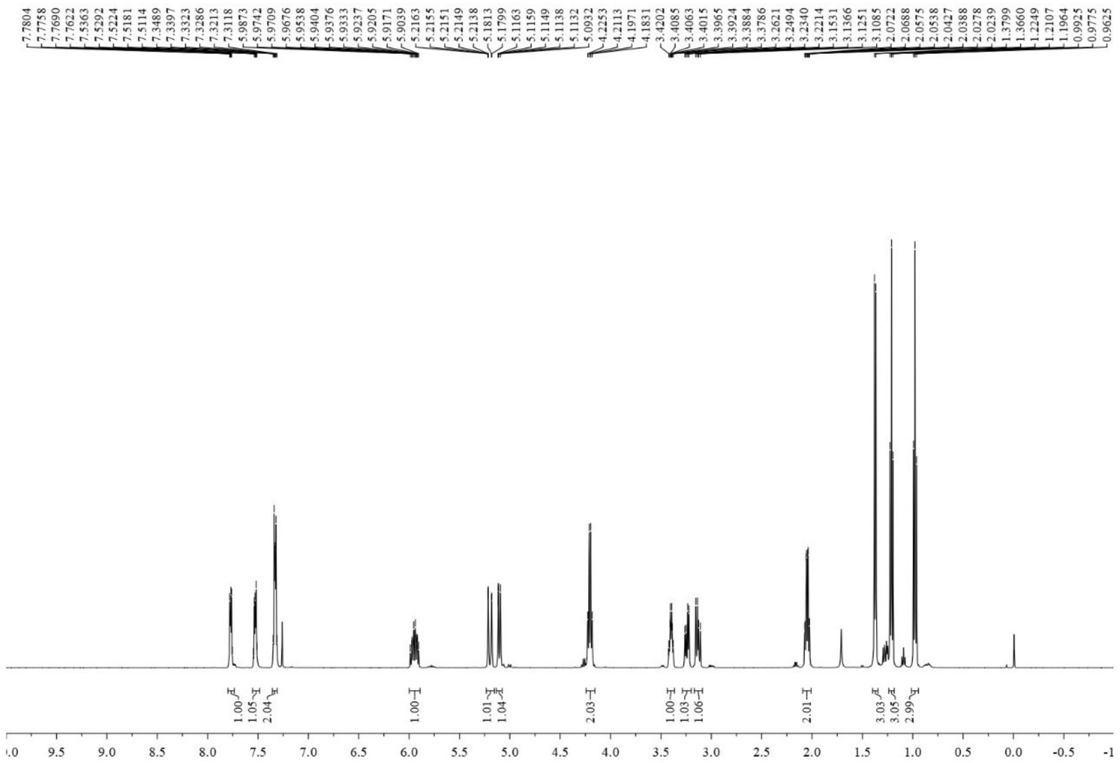

^{13}C NMR of (*R,R*)-**3aa (125 MHz, CDCl$_3$)**

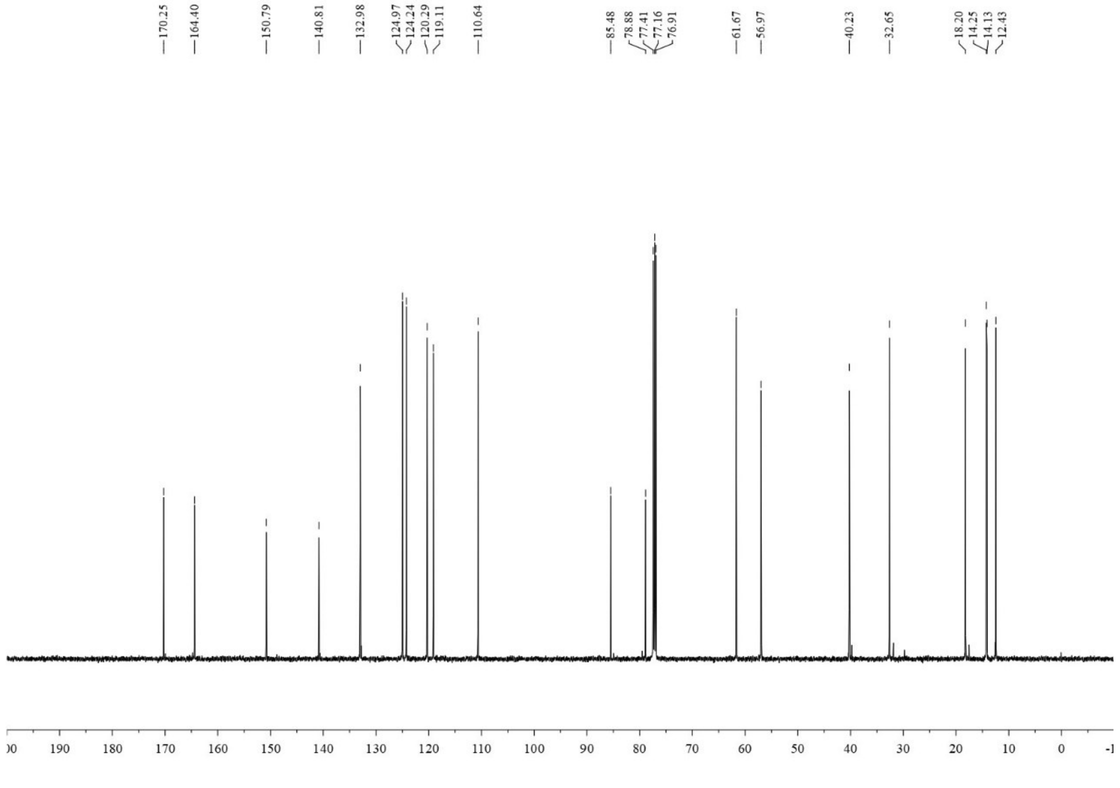

^{1}H NMR of (*S,S*)-3aa (500 MHz, CDCl$_3$)

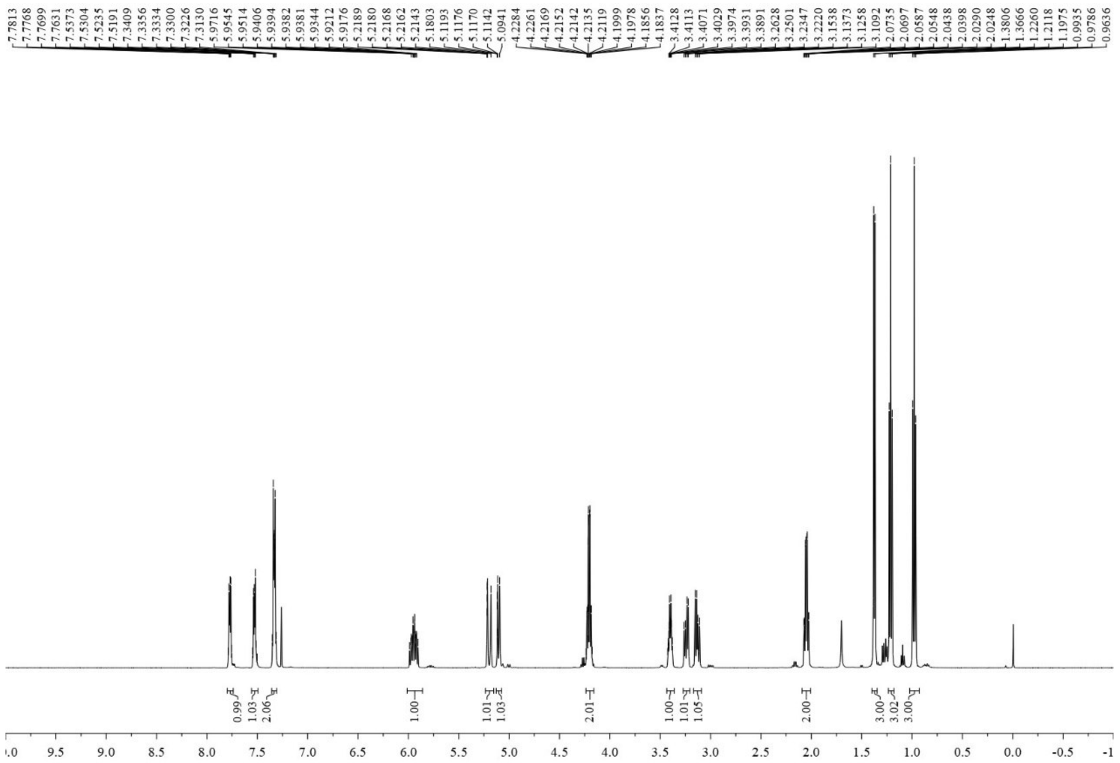

^{13}C NMR of (*S,S*)-3aa (125 MHz, CDCl$_3$)

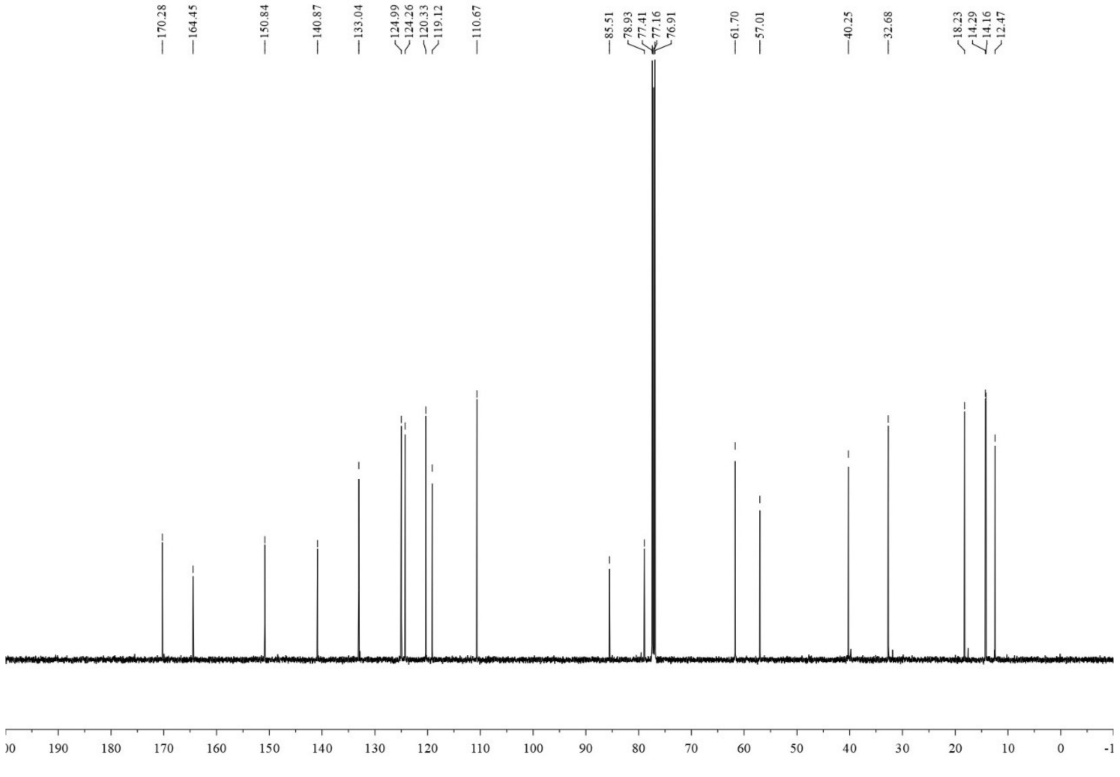

^{1}H NMR of 3ab (400 MHz, CDCl$_3$)

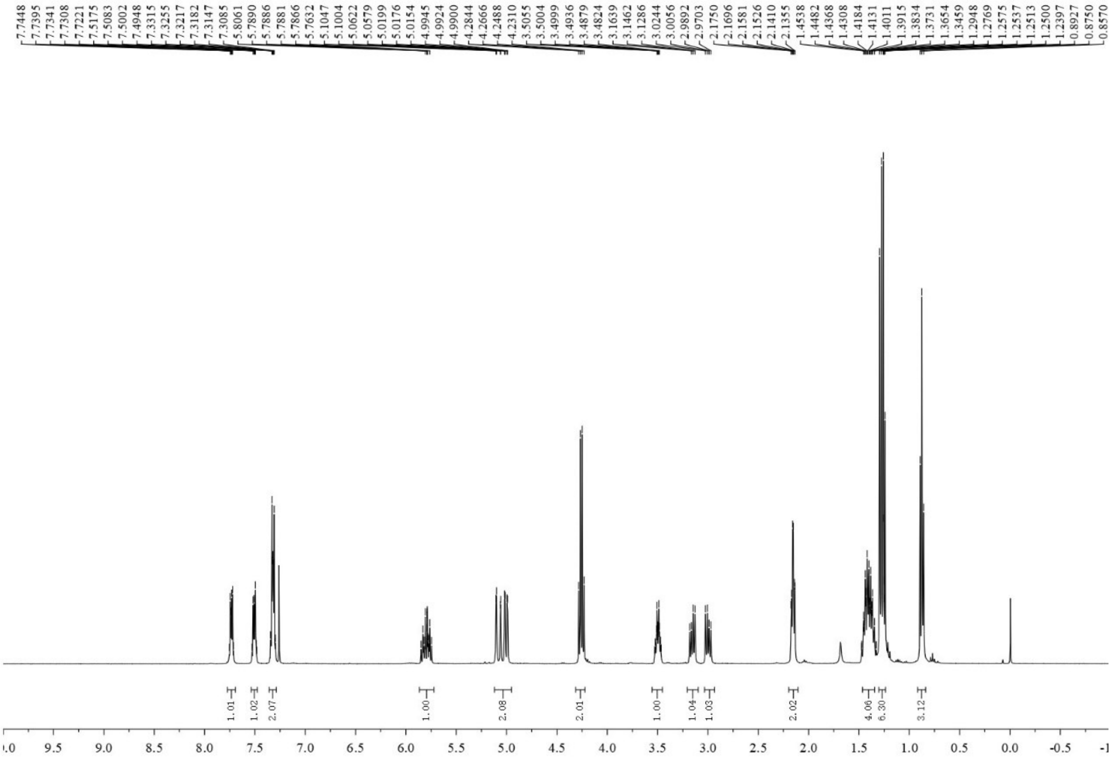

^{13}C NMR of 3ab (100 MHz, CDCl$_3$)

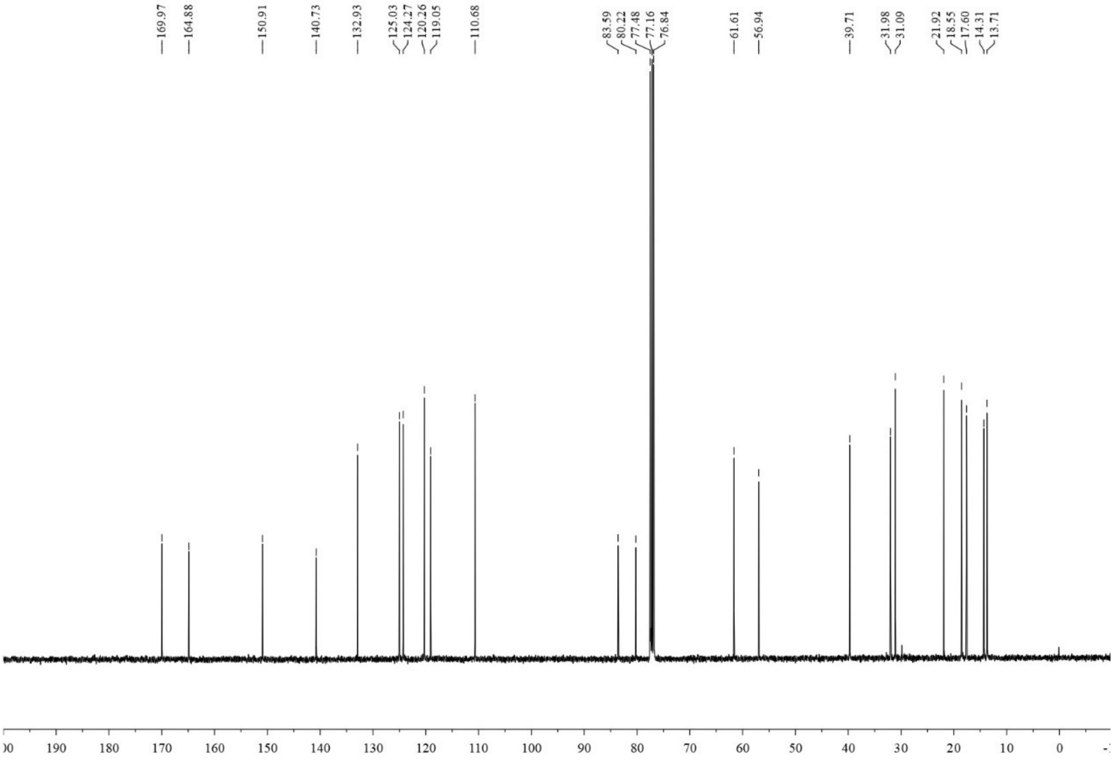

^{1}H NMR of **3ac (400 MHz, CDCl$_3$)**

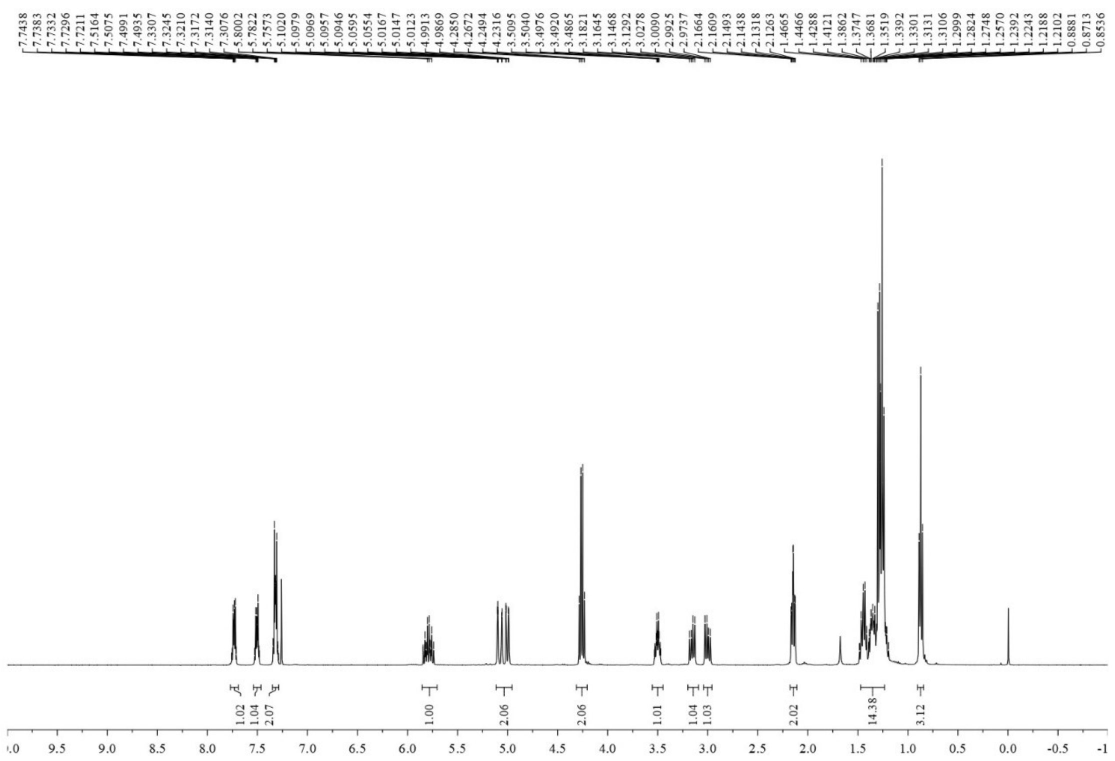

^{13}C NMR of **3ac (100 MHz, CDCl$_3$)**

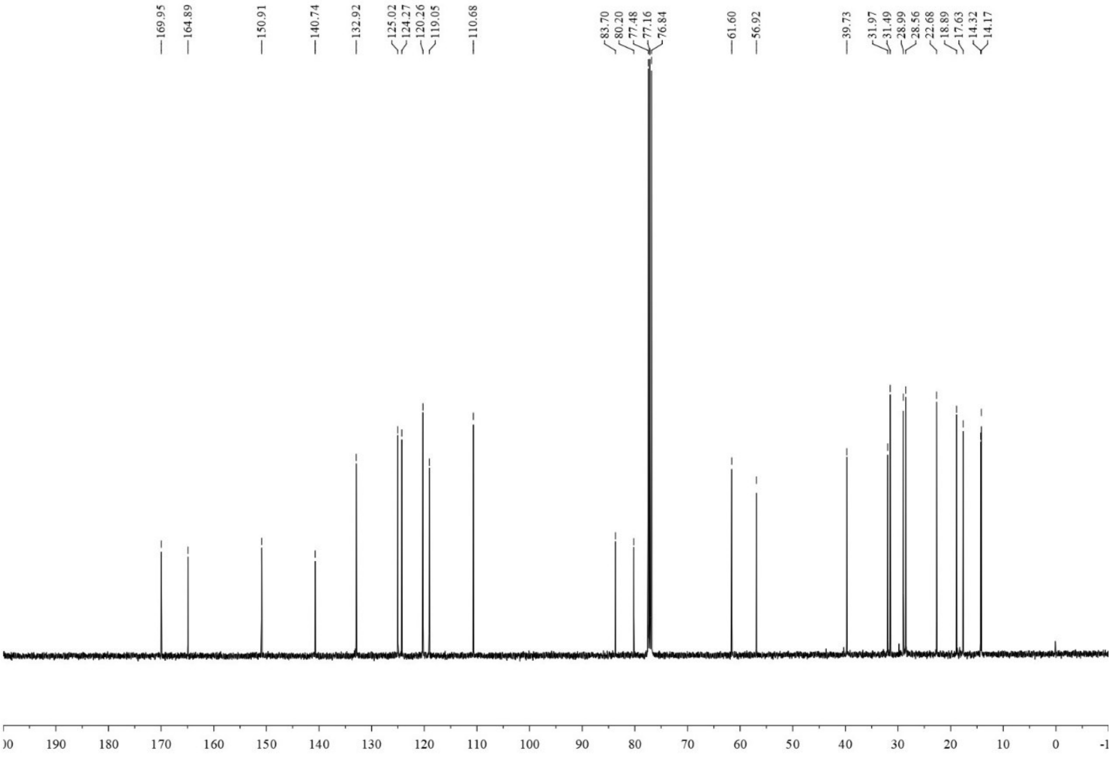

^{1}H **NMR** of **3ad (400 MHz, $CDCl_3$)**

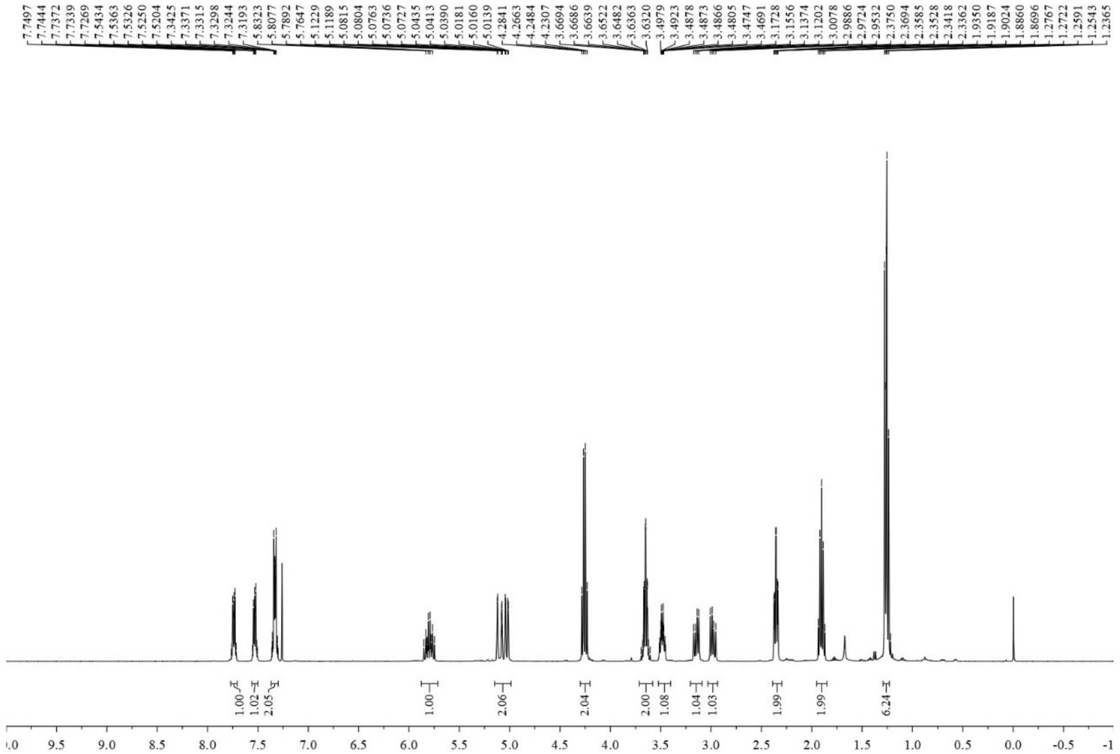

^{13}C **NMR** of **3ad (100 MHz, $CDCl_3$)**

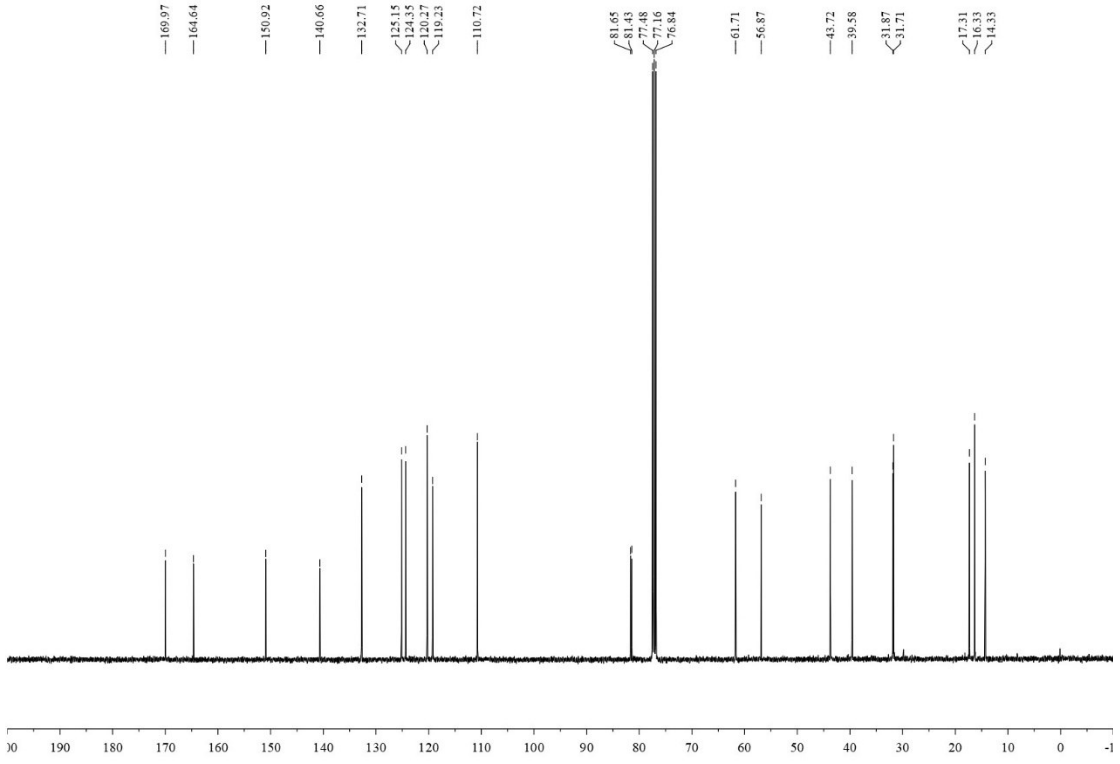

^{1}H NMR of **3ae (400 MHz, CDCl$_3$)**

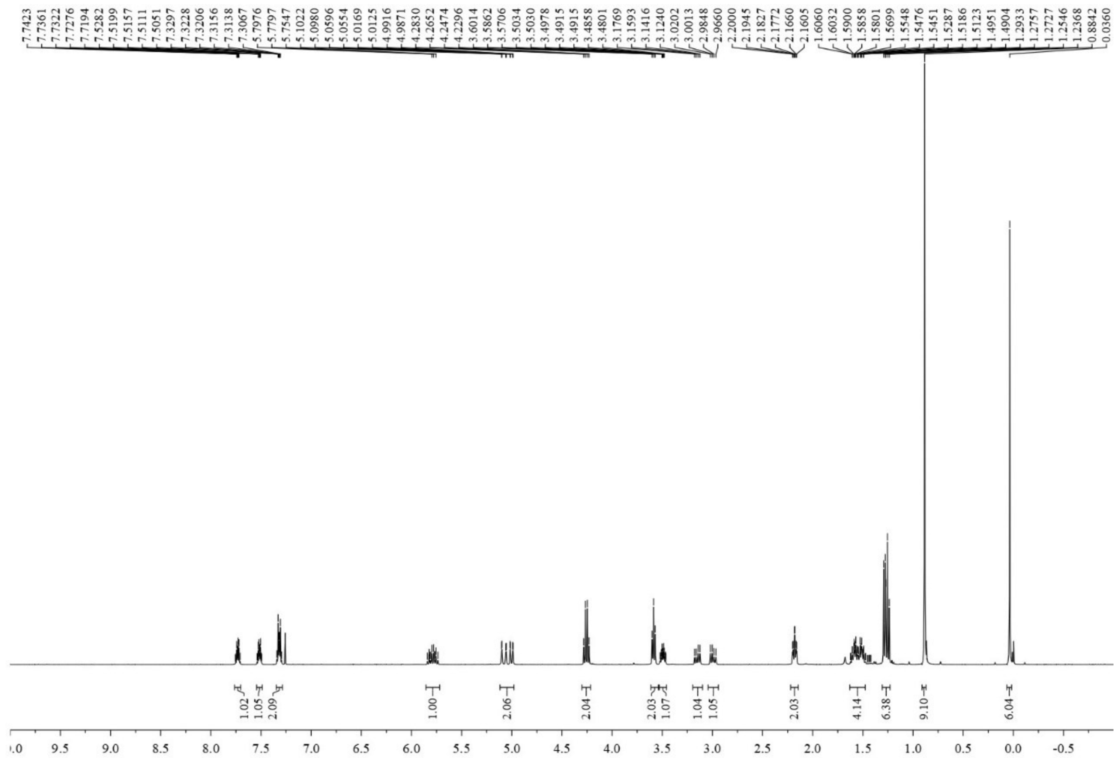

^{13}C NMR of **3ae (100 MHz, CDCl$_3$)**

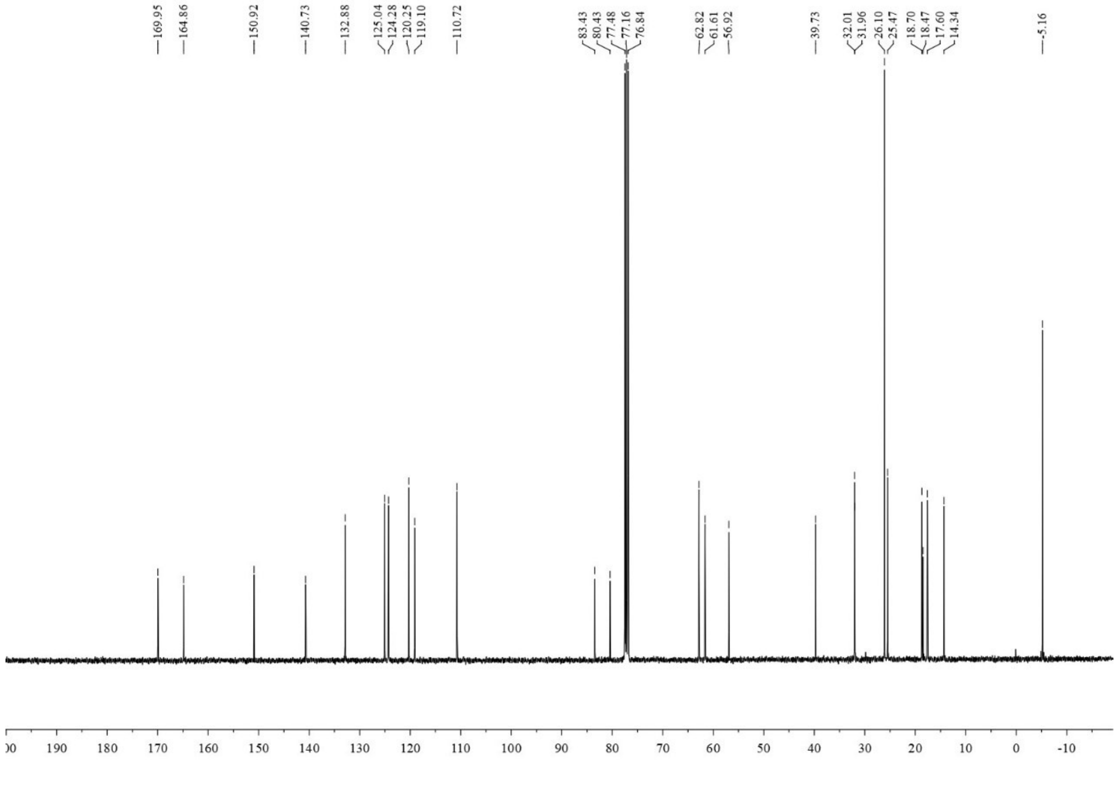

^{1}H NMR of **3af (400 MHz, $CDCl_3$)**

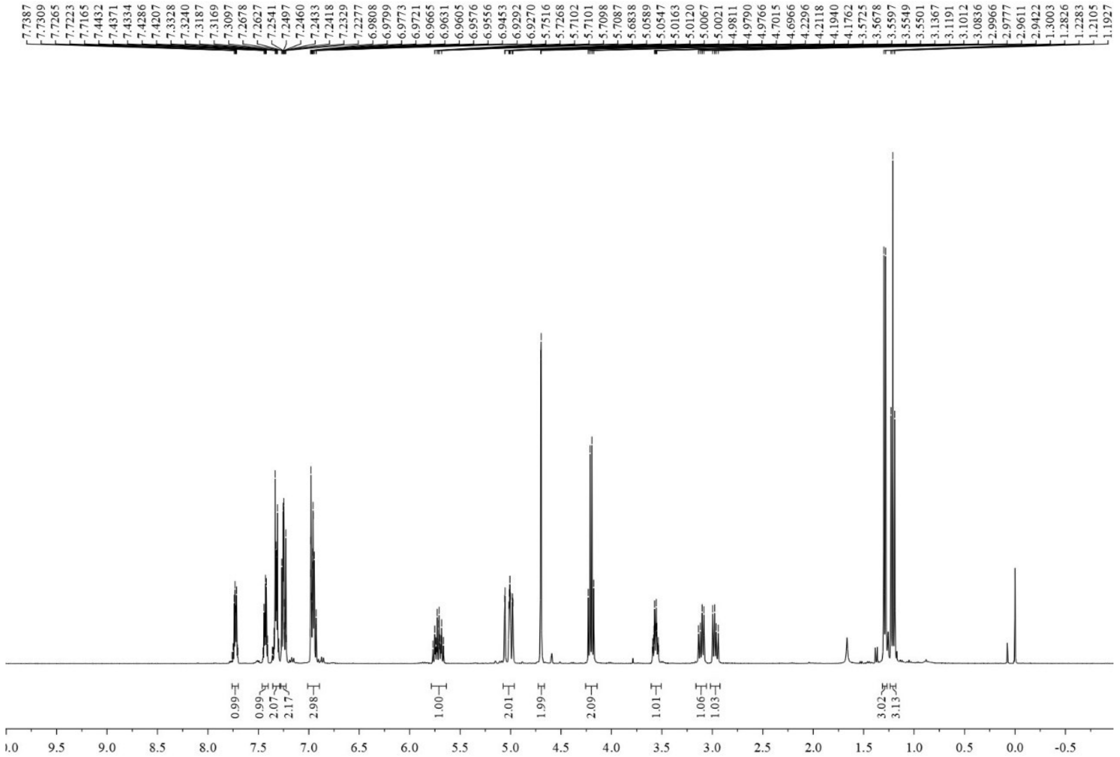

^{13}C NMR of **3af (100 MHz, $CDCl_3$)**

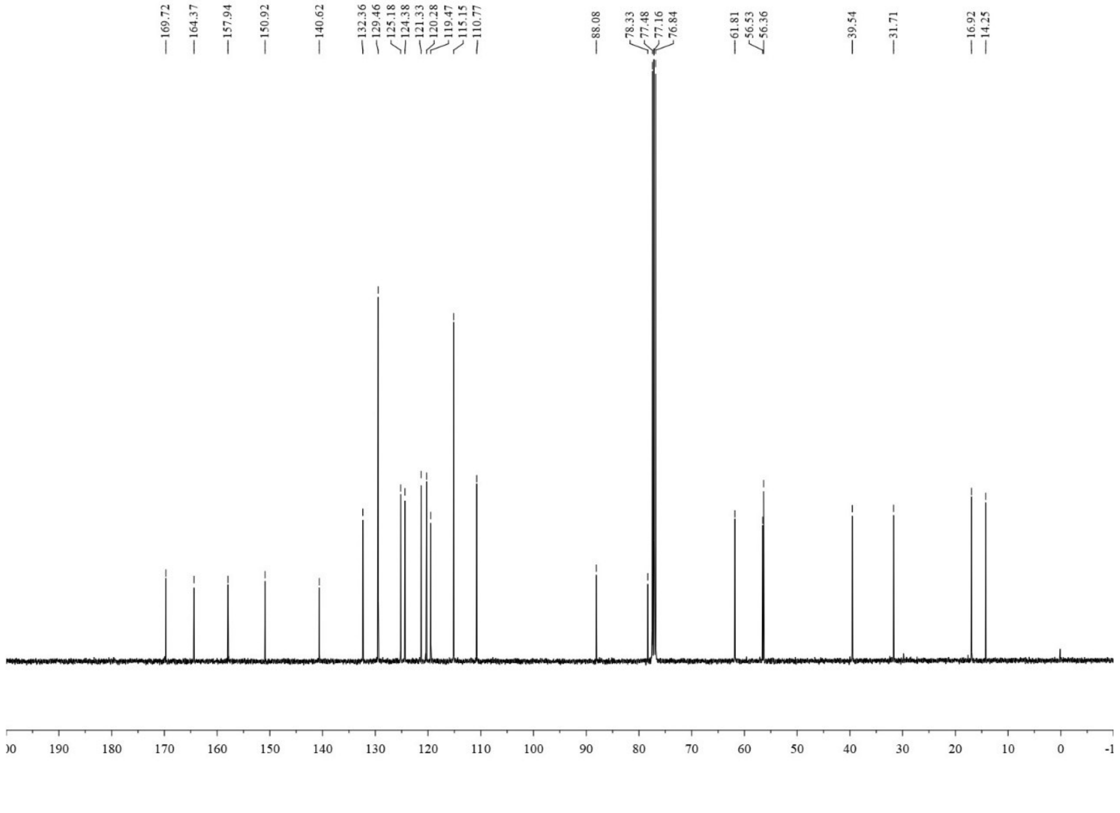

^{1}H **NMR** of **3ag (500 MHz, CDCl$_3$)**

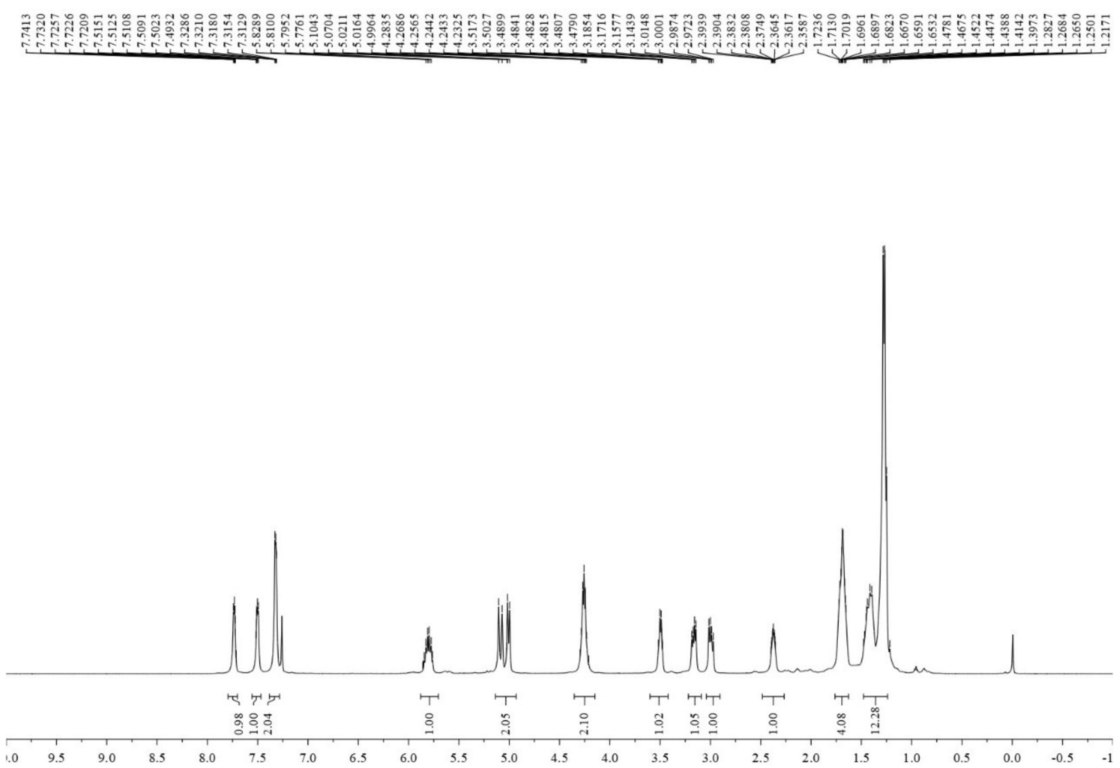

^{13}C **NMR** of **3ag (125 MHz, CDCl$_3$)**

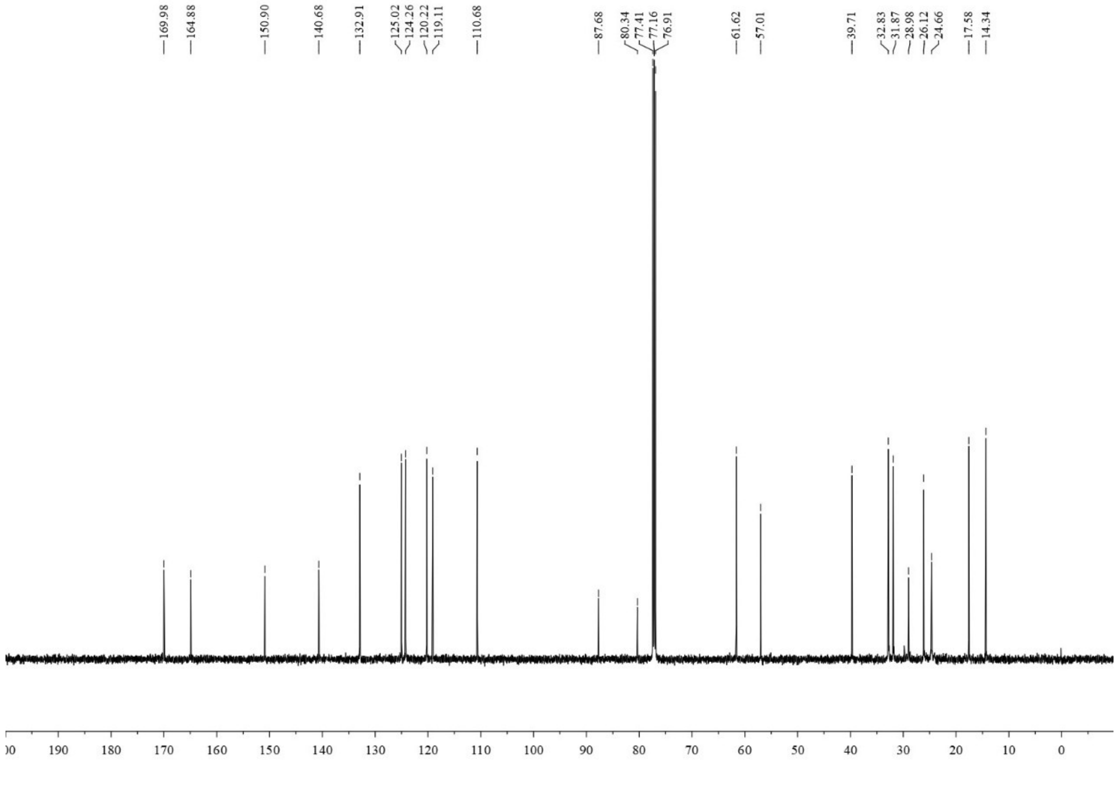

^{1}H NMR of **3ah (400 MHz, CDCl$_3$)**

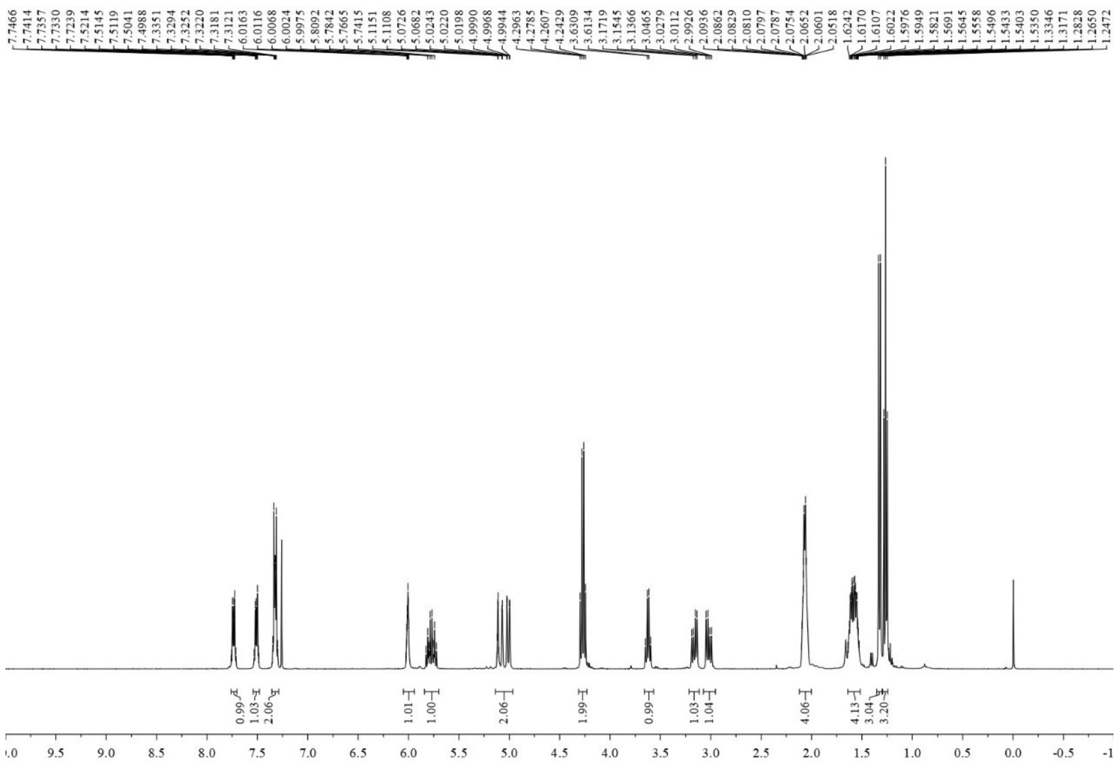

^{13}C NMR of **3ah (100 MHz, CDCl$_3$)**

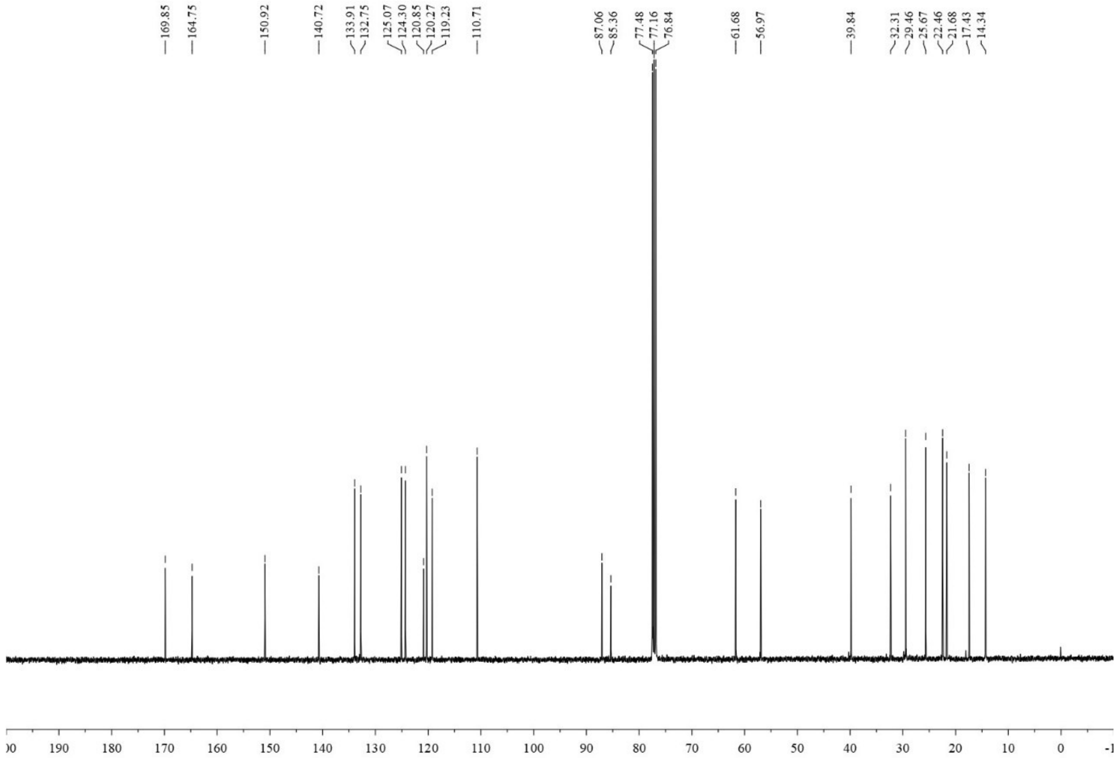

^{1}H NMR of **3ai (400 MHz, CDCl$_3$)**

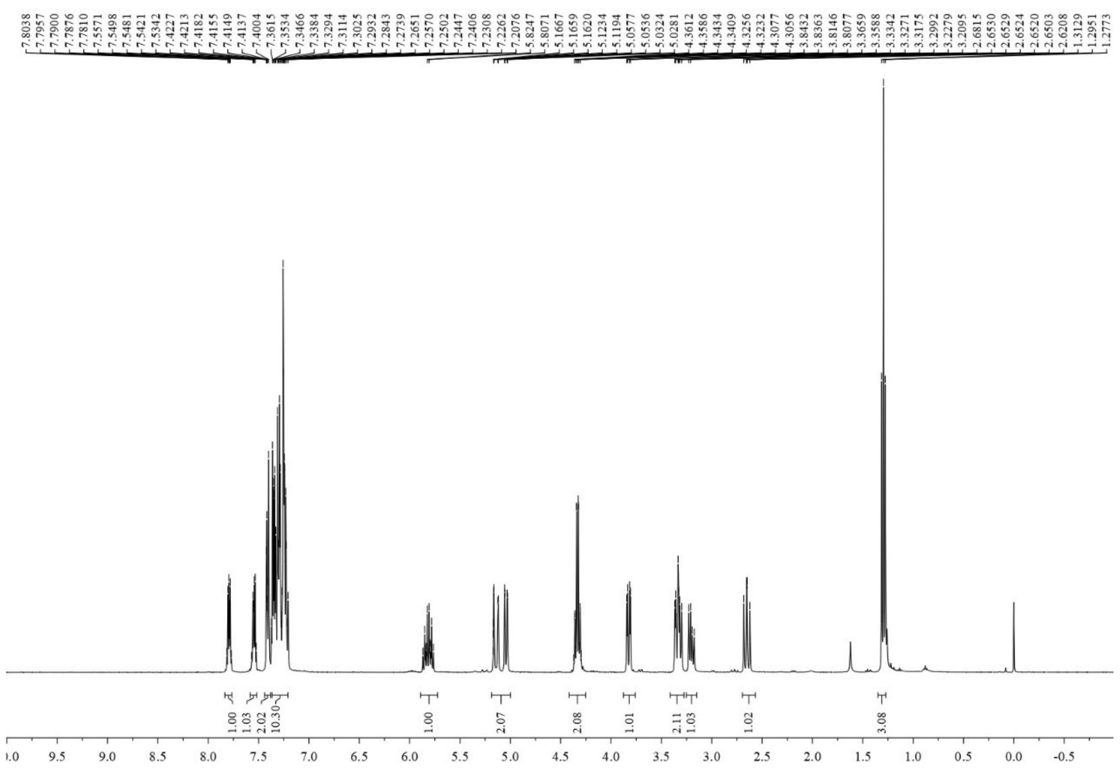

^{13}C NMR of **3ai (100 MHz, CDCl$_3$)**

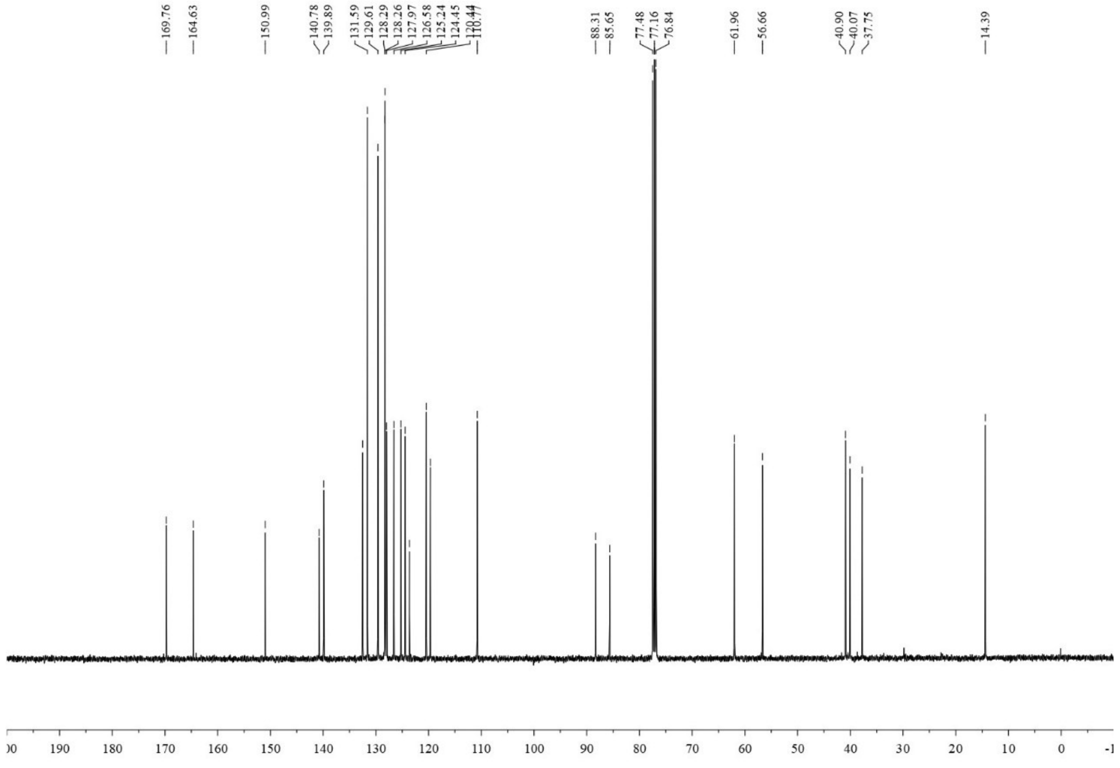

^{1}H NMR of **3aj (500 MHz, CDCl$_3$)**

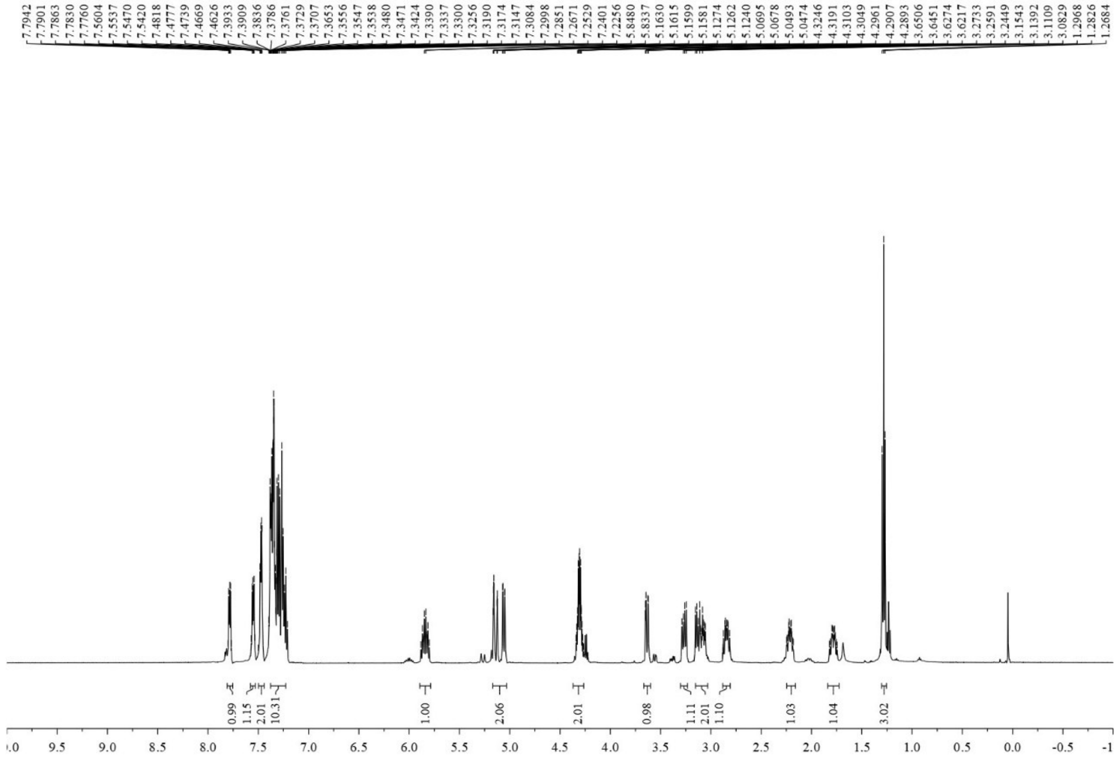

^{13}C NMR of **3aj (125 MHz, CDCl$_3$)**

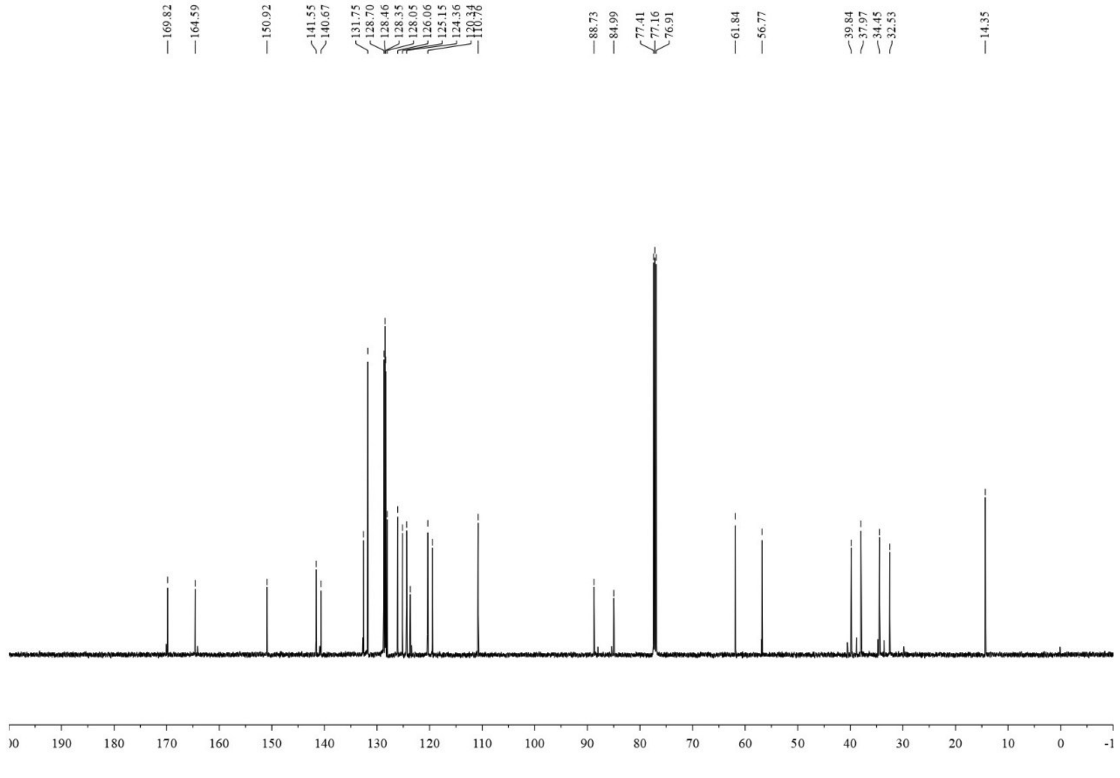

^{1}H NMR of **3ak** (500 MHz, $CDCl_3$)

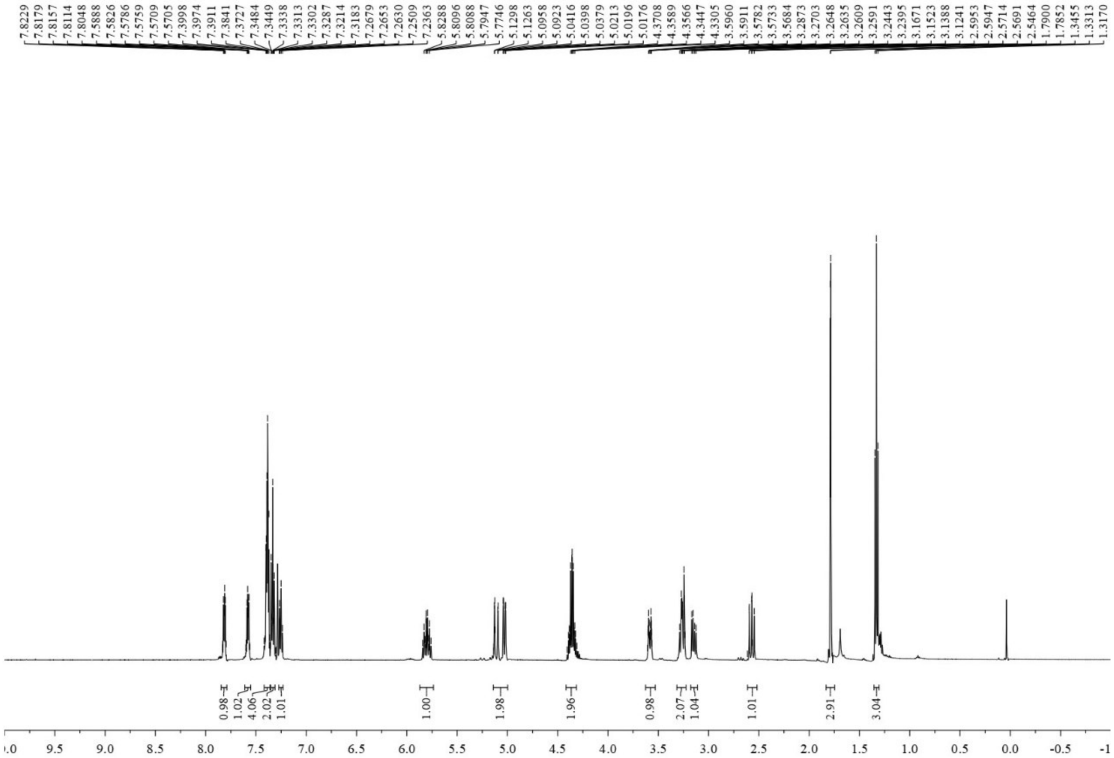

^{13}C NMR of **3ak** (125 MHz, $CDCl_3$)

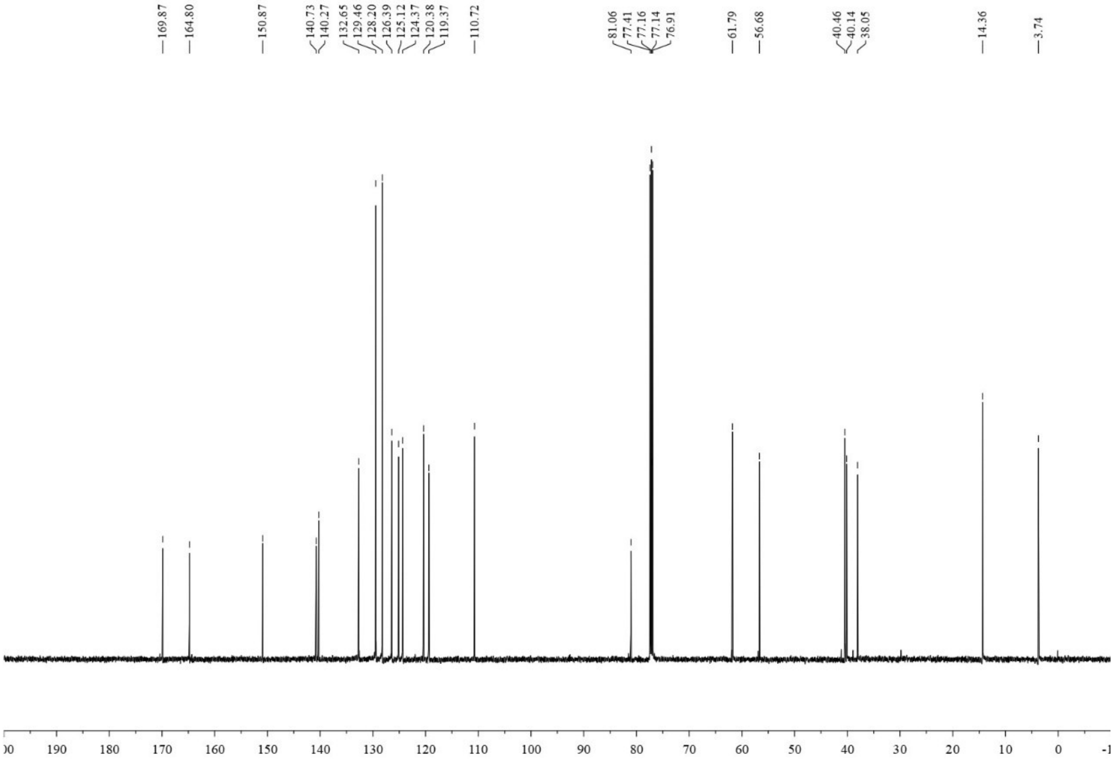

^{1}H NMR of **3al (400 MHz, CDCl$_3$)**

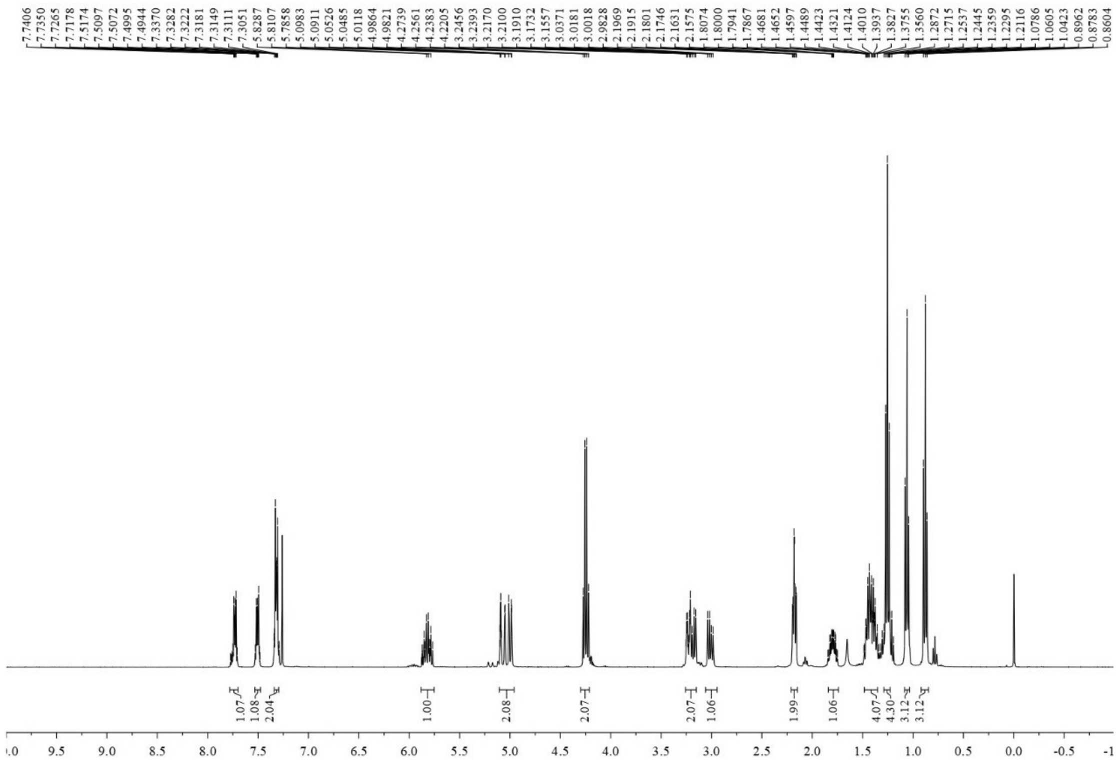

^{13}C NMR of **3al (100 MHz, CDCl$_3$)**

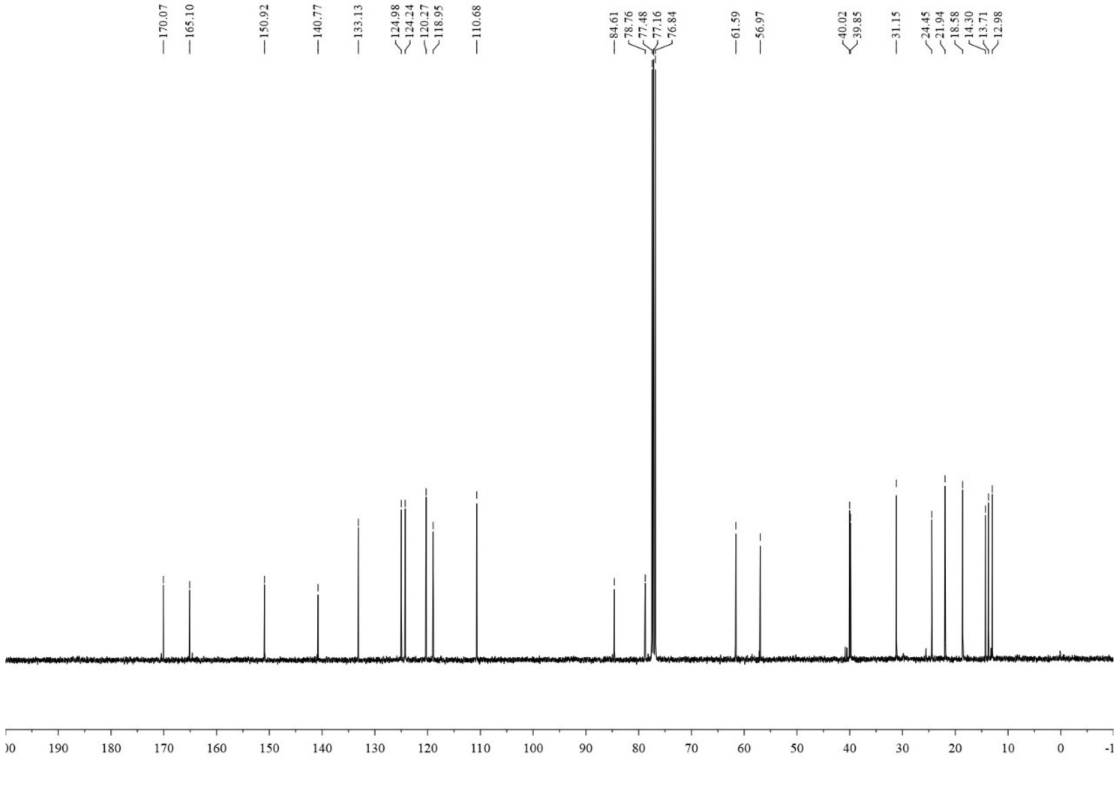

^{1}H NMR of **3am (400 MHz, CDCl$_3$)**

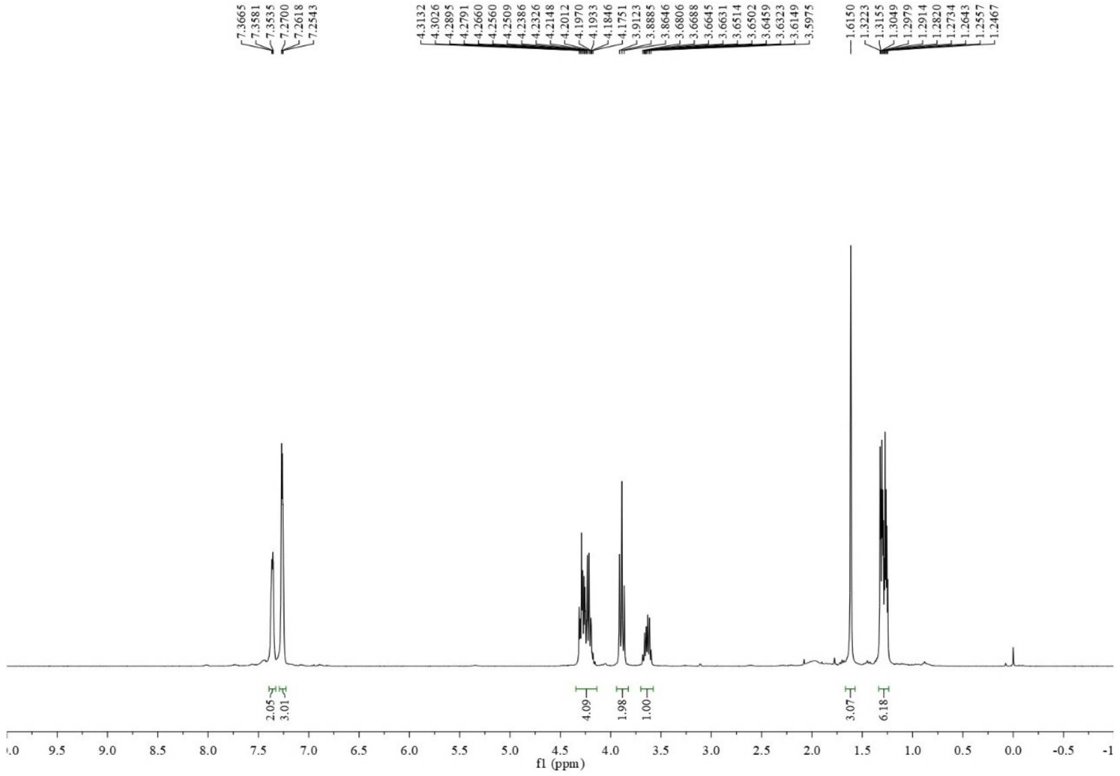

^{13}C NMR of **3am (100 MHz, CDCl$_3$)**

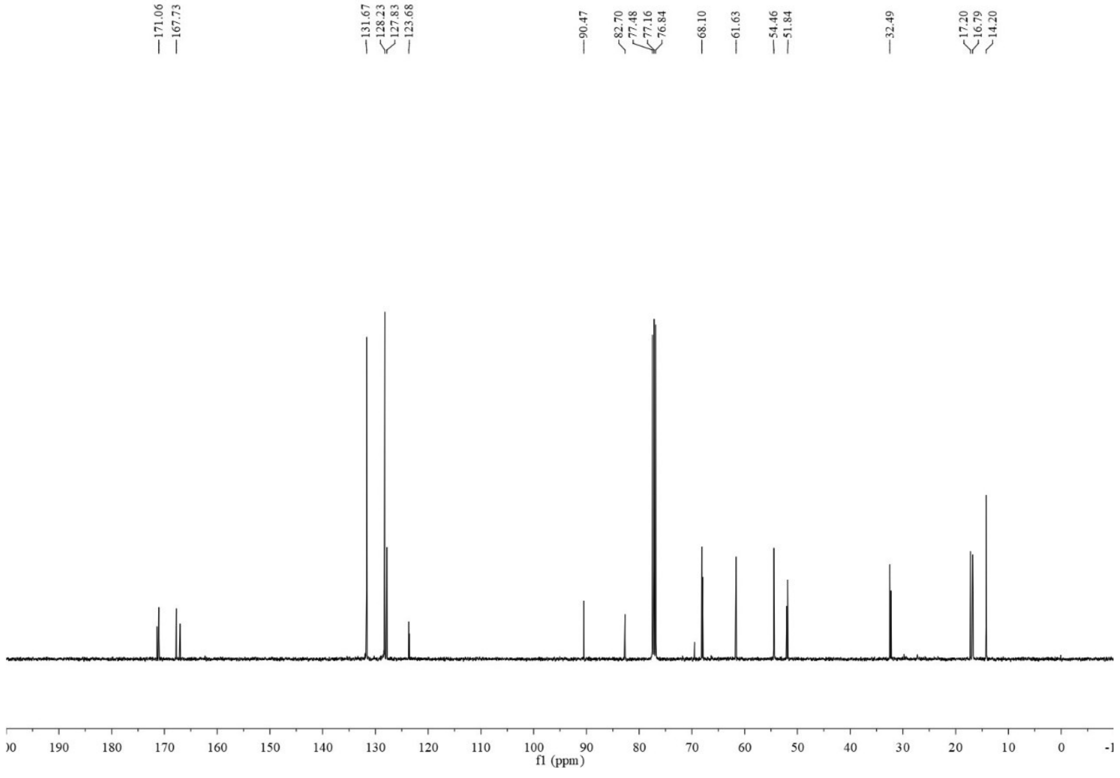

^{1}H NMR of **3an (400 MHz, CDCl$_3$)**

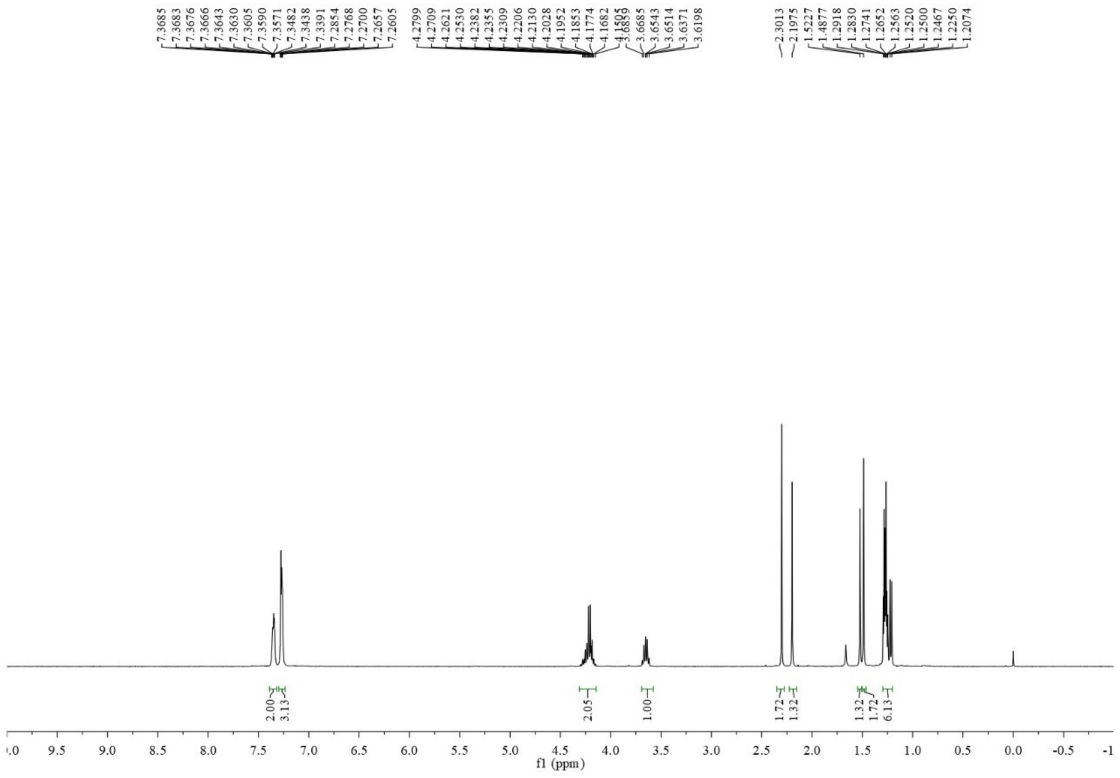

^{13}C NMR of **3an (100 MHz, CDCl$_3$)**

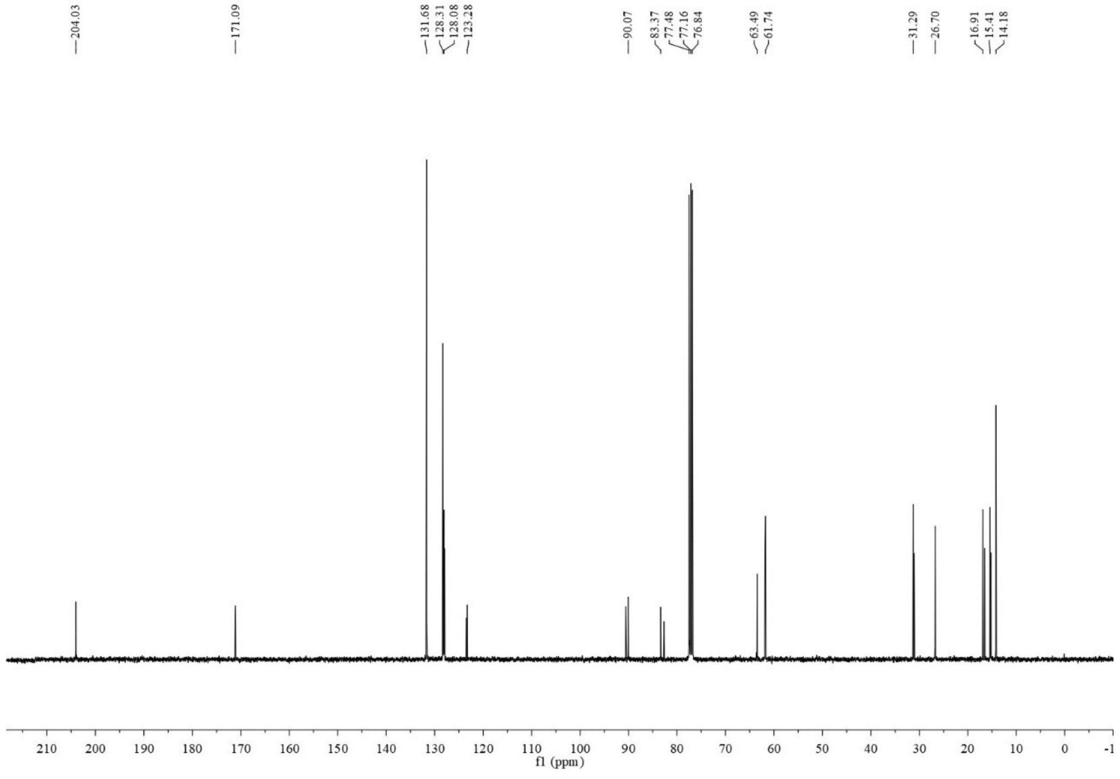

^{1}H NMR of **7a (500 MHz, CDCl$_3$)**

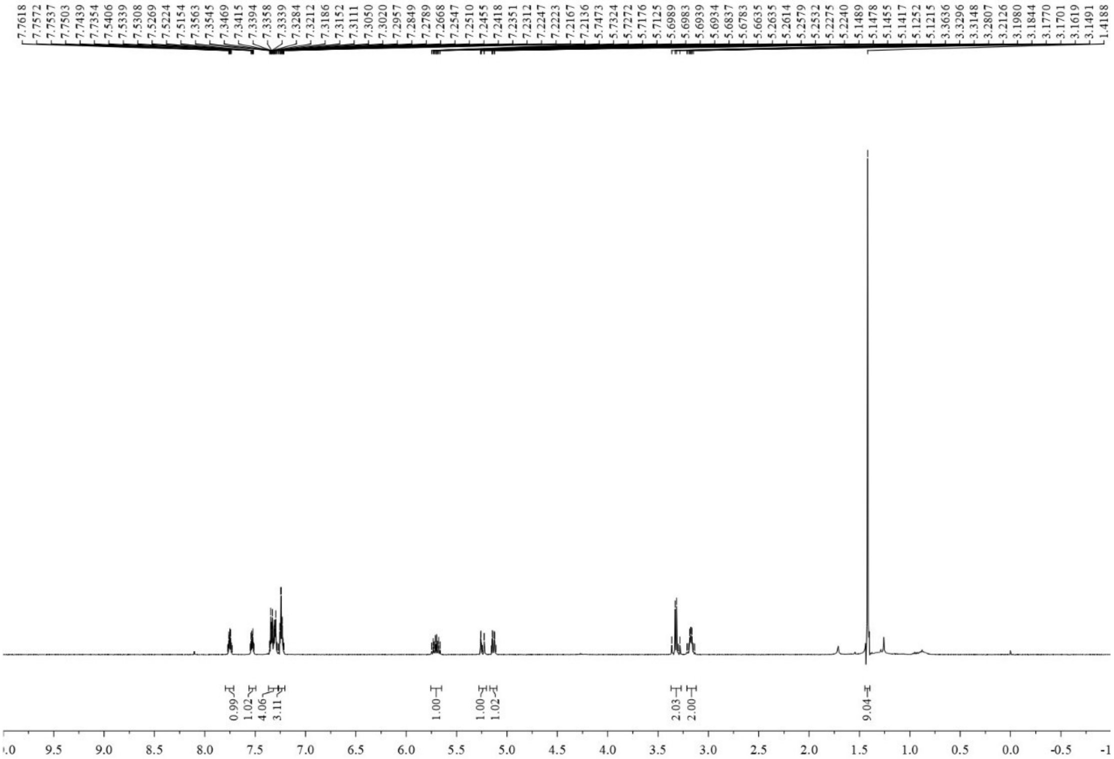

^{13}C NMR of **7a (125 MHz, CDCl$_3$)**

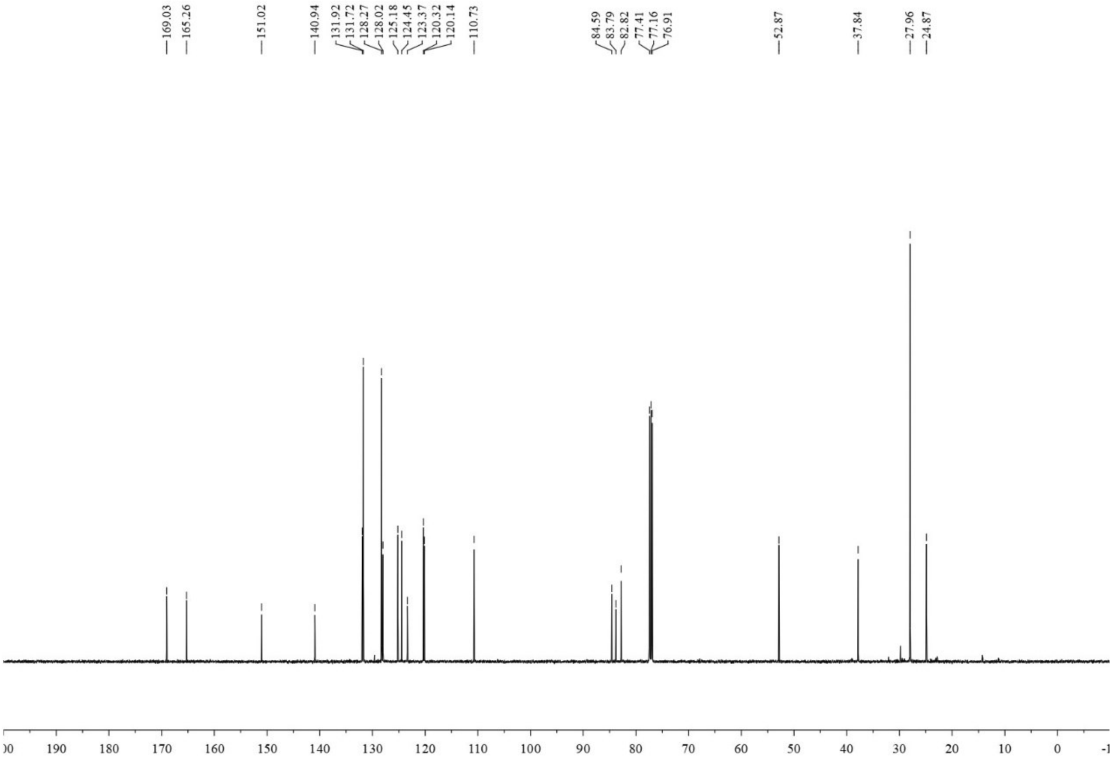

^{1}H NMR of **7b (500 MHz, $CDCl_3$)**

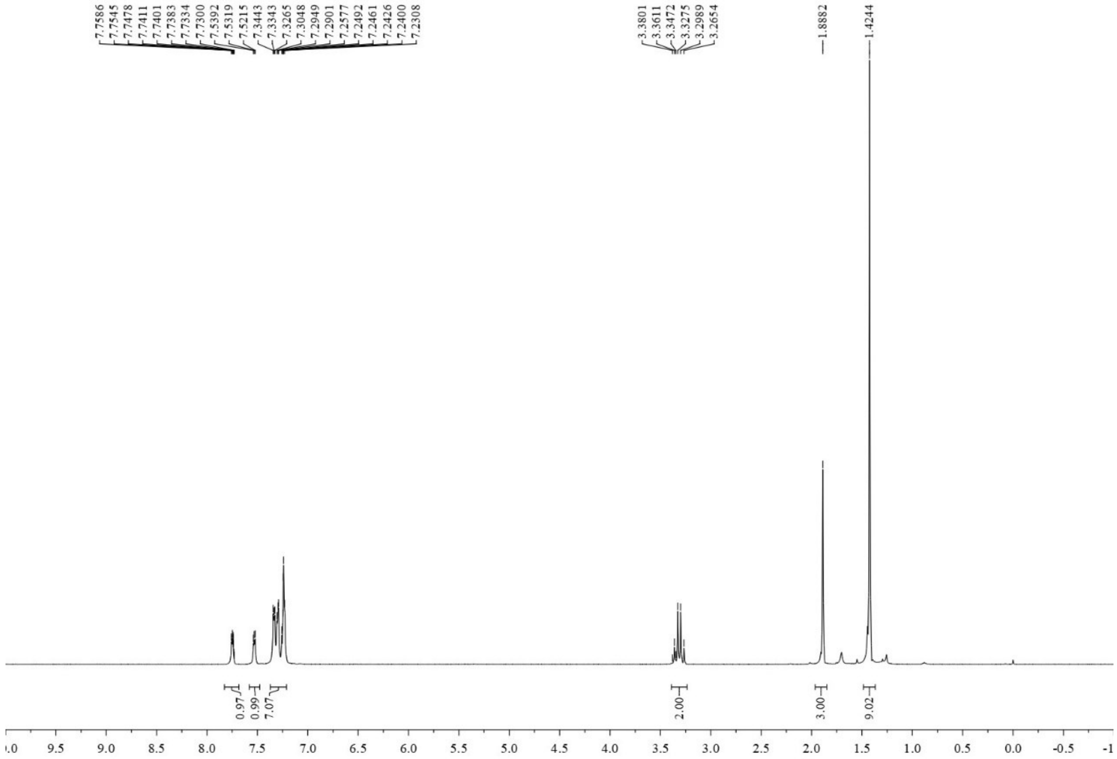

^{13}C NMR of **7b (125 MHz, $CDCl_3$)**

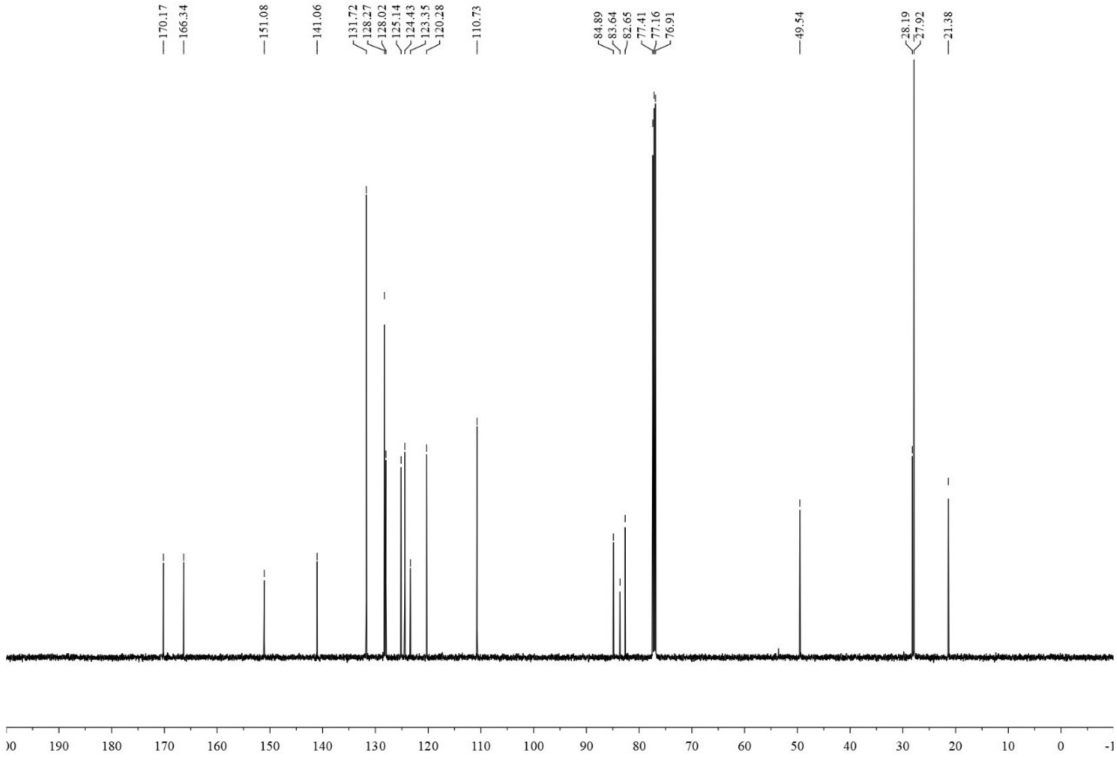

^{1}H NMR of **7c (500 MHz, $CDCl_3$)**

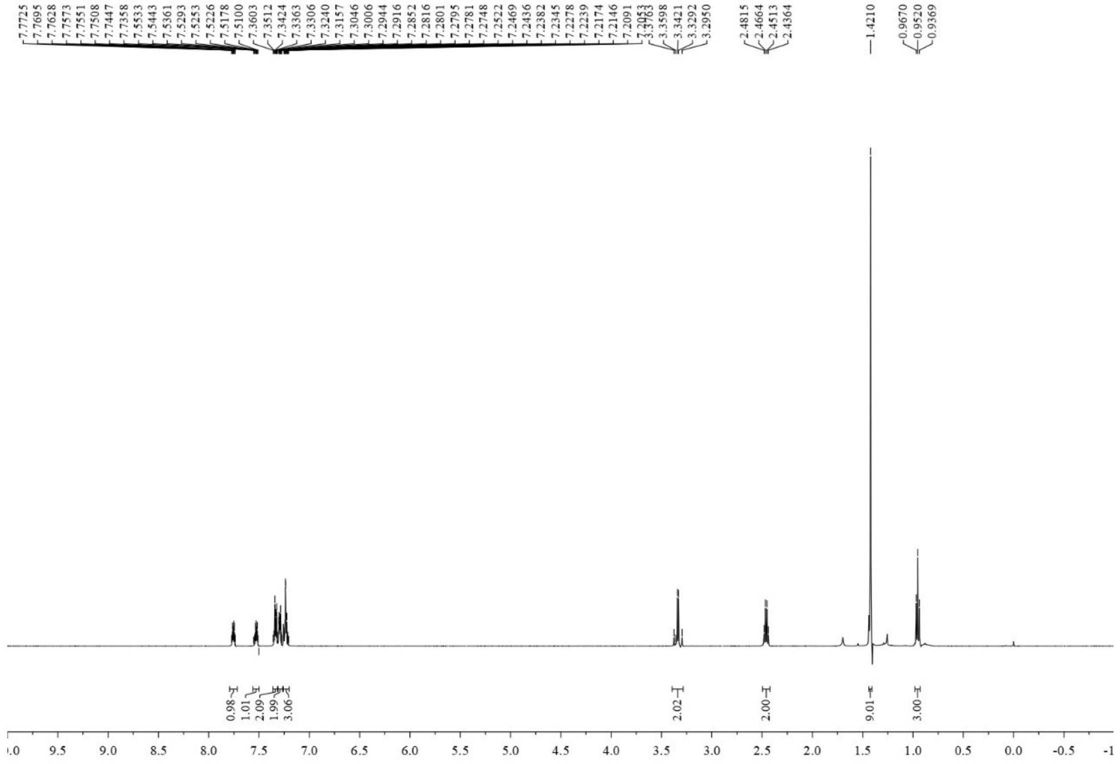

^{13}C NMR of **7c (125 MHz, $CDCl_3$)**

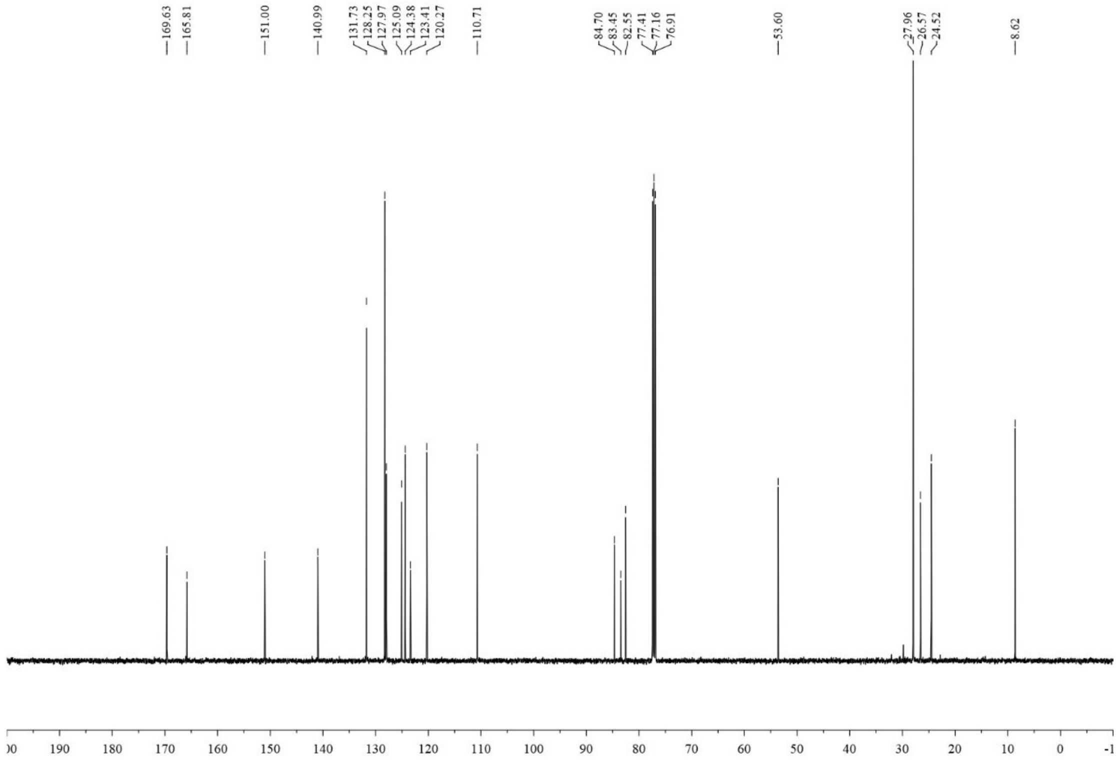

^{1}H NMR of **7d (500 MHz, CDCl$_3$)**

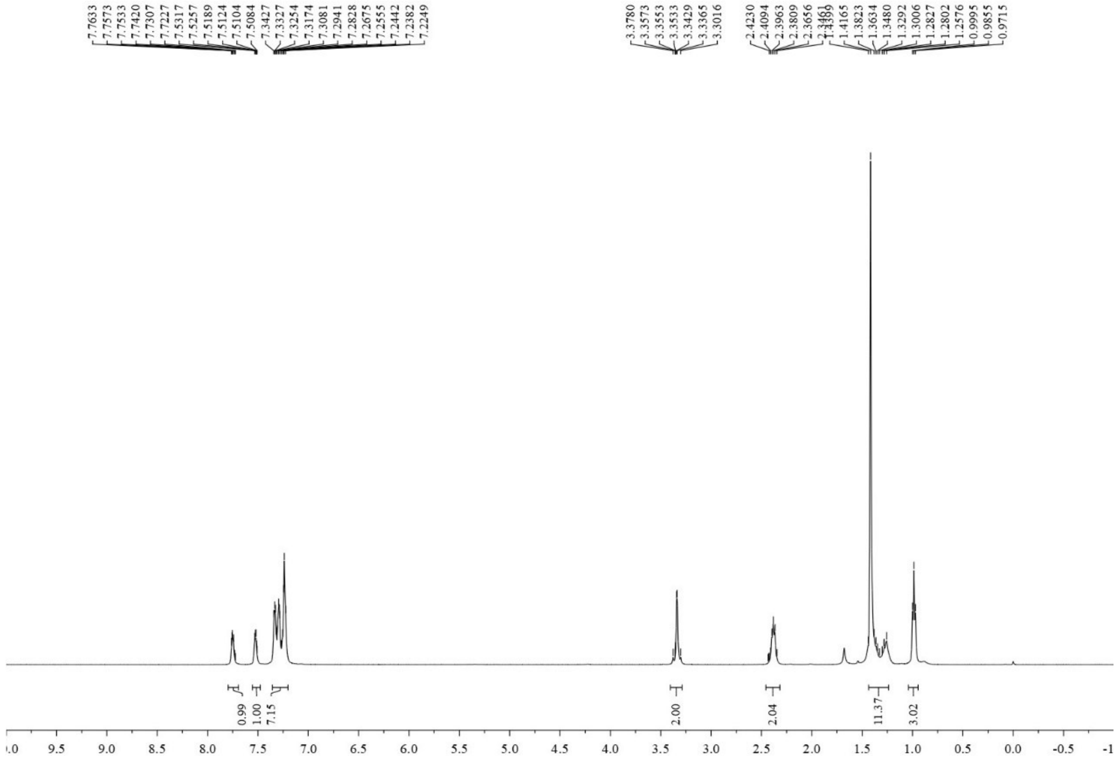

^{13}C NMR of **7d (125 MHz, CDCl$_3$)**

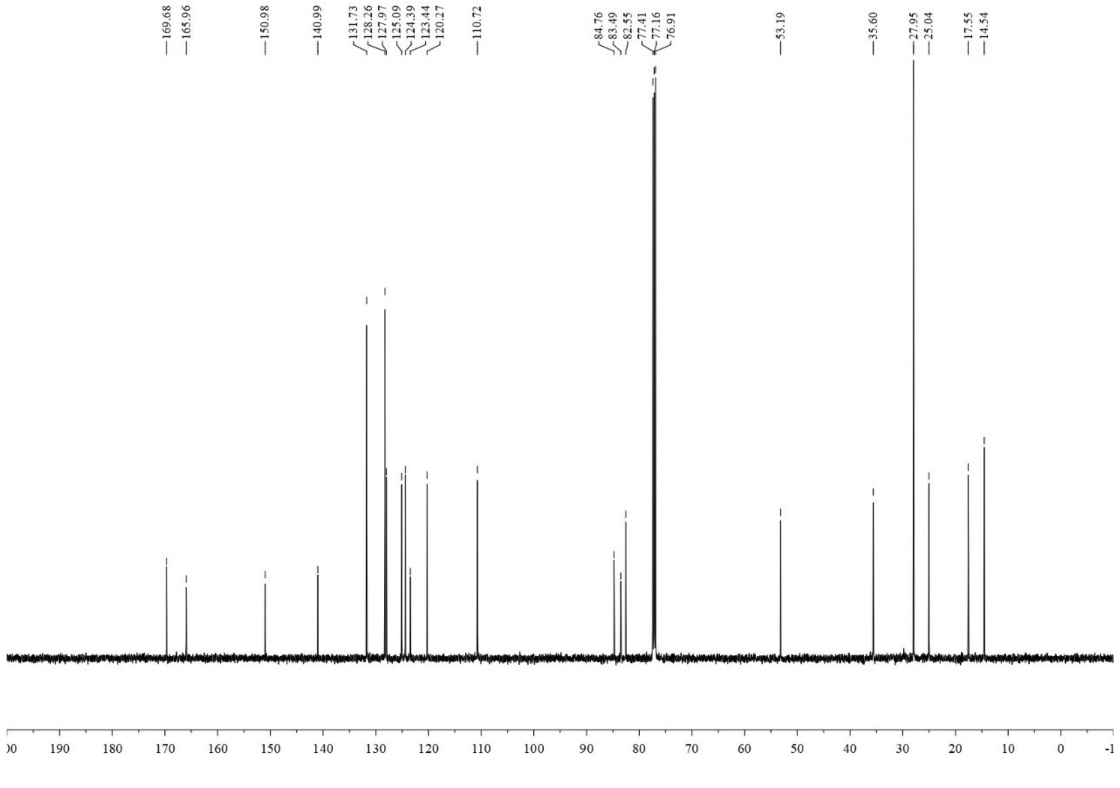

^{1}H NMR of **7e (400 MHz, $CDCl_3$)**

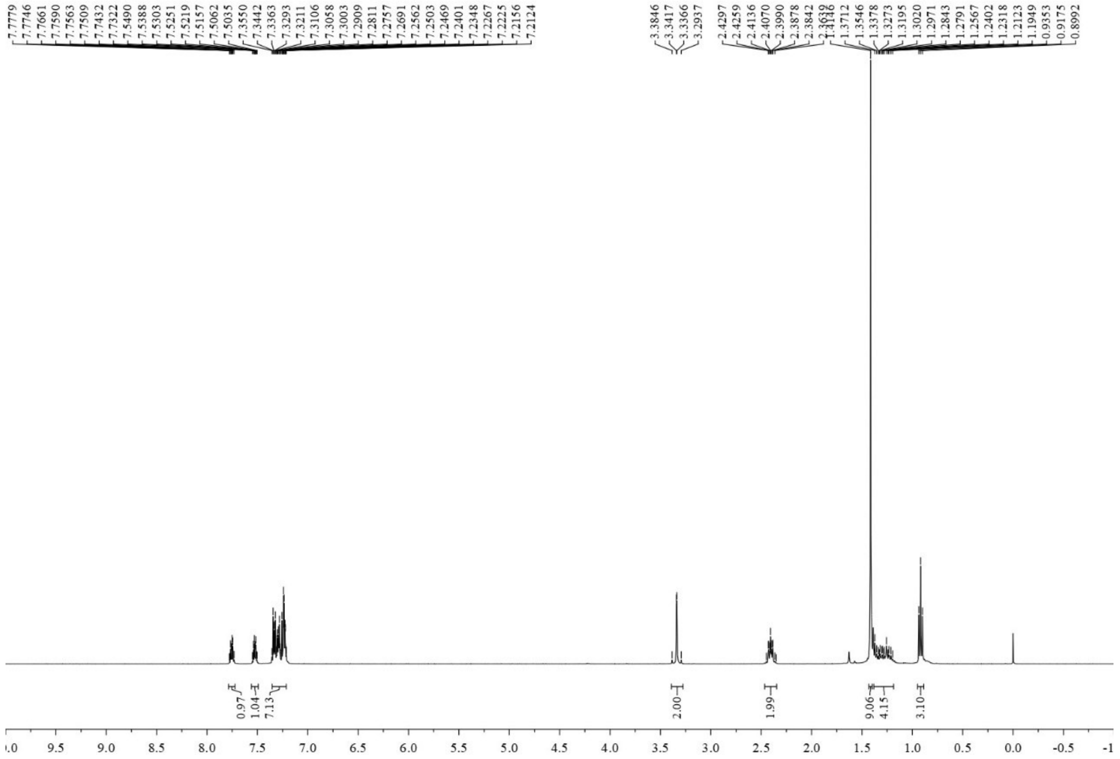

^{13}C NMR of **7e (100 MHz, $CDCl_3$)**

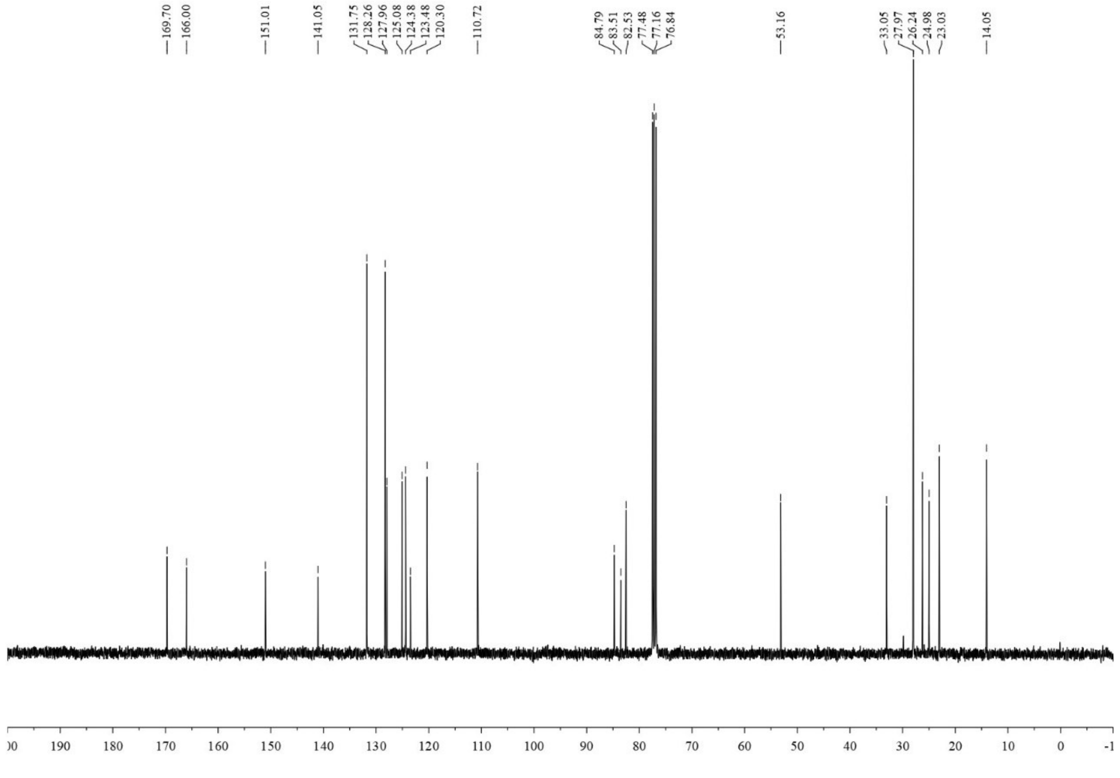

^{1}H NMR of **7f (400 MHz, CDCl$_3$)**

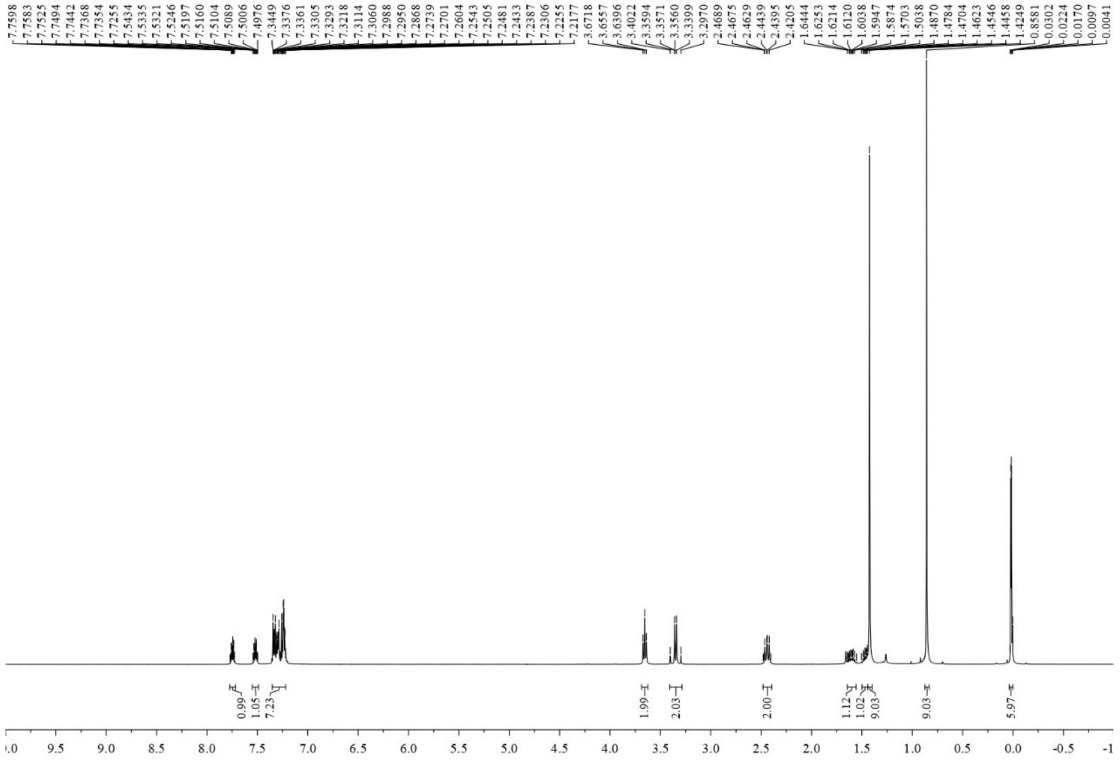

^{13}C NMR of **7f (100 MHz, CDCl$_3$)**

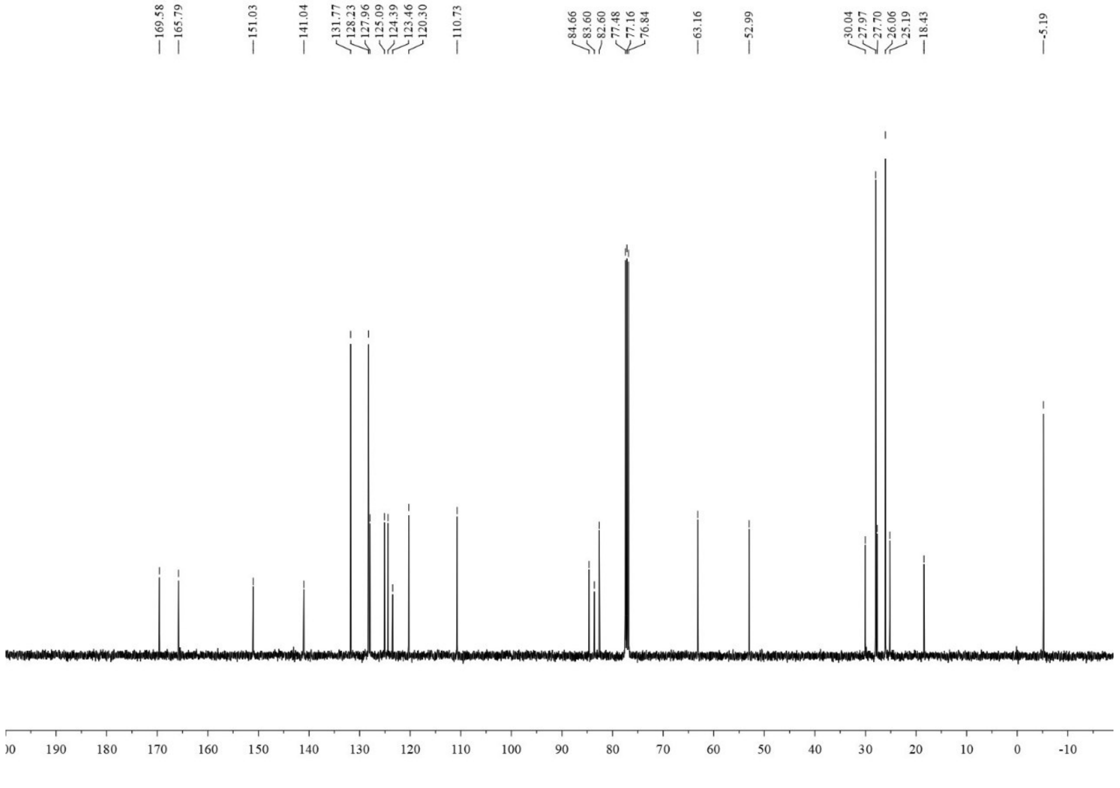

^{1}H NMR of **7g (400 MHz, CDCl$_3$)**

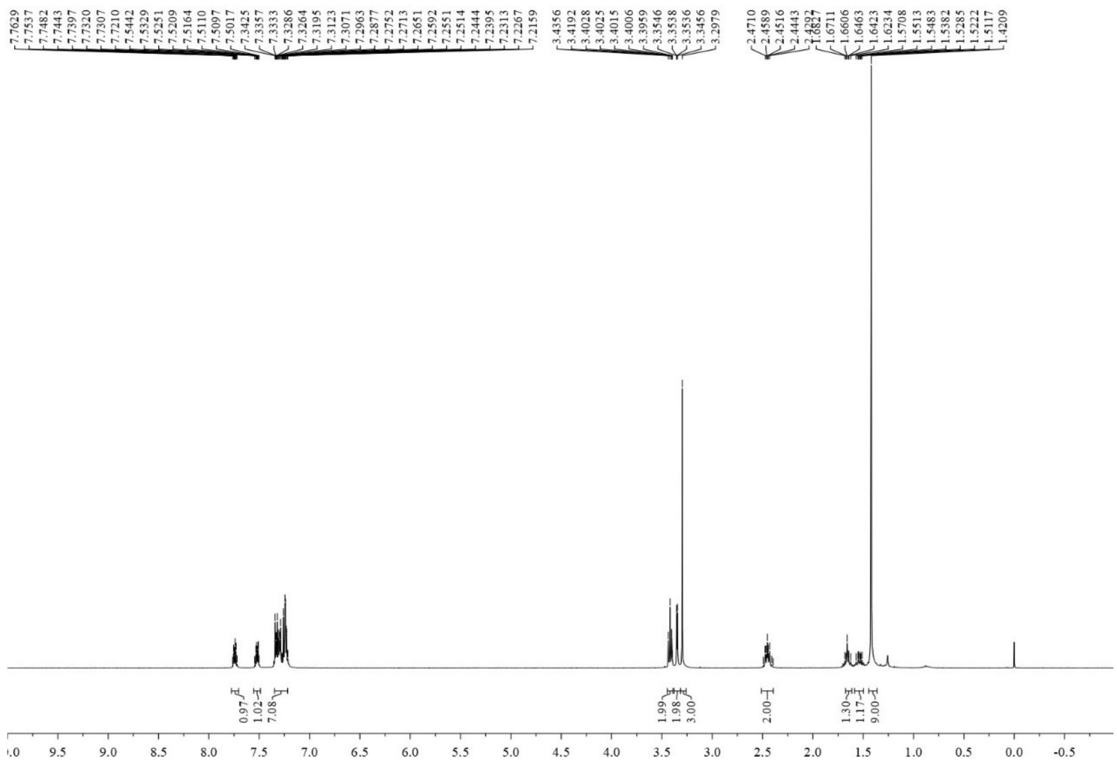

^{13}C NMR of **7g (100 MHz, CDCl$_3$)**

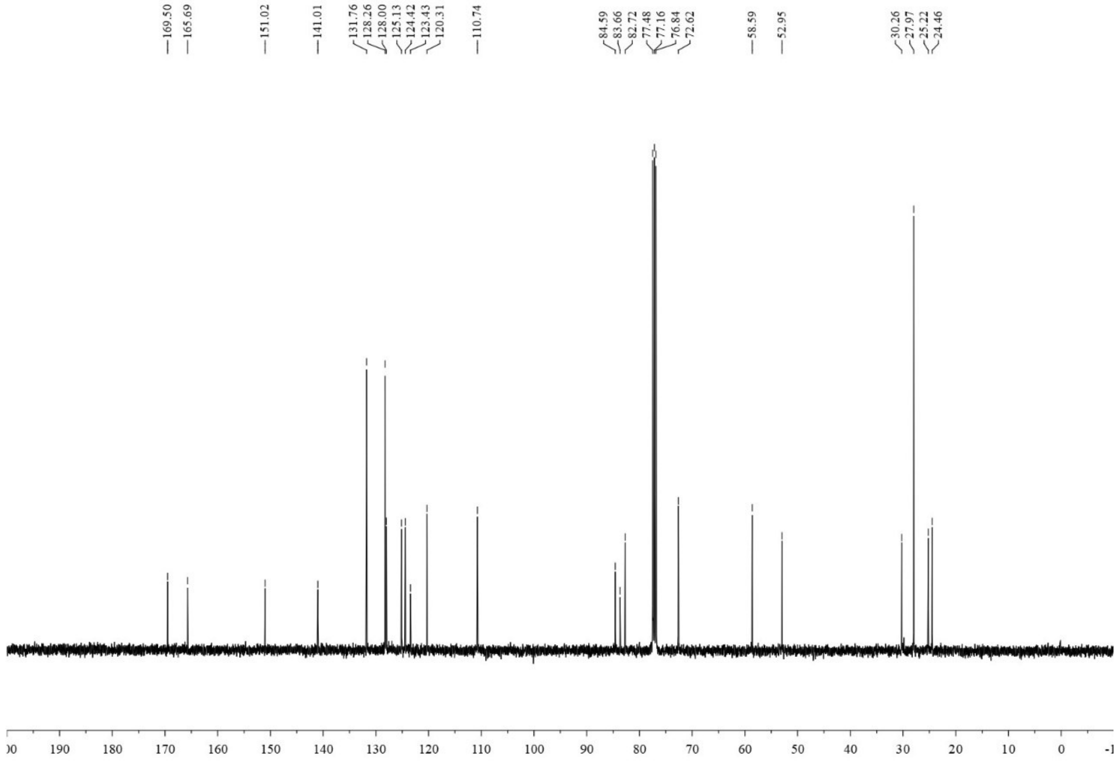

^{1}H NMR of **7h (400 MHz, CDCl$_3$)**

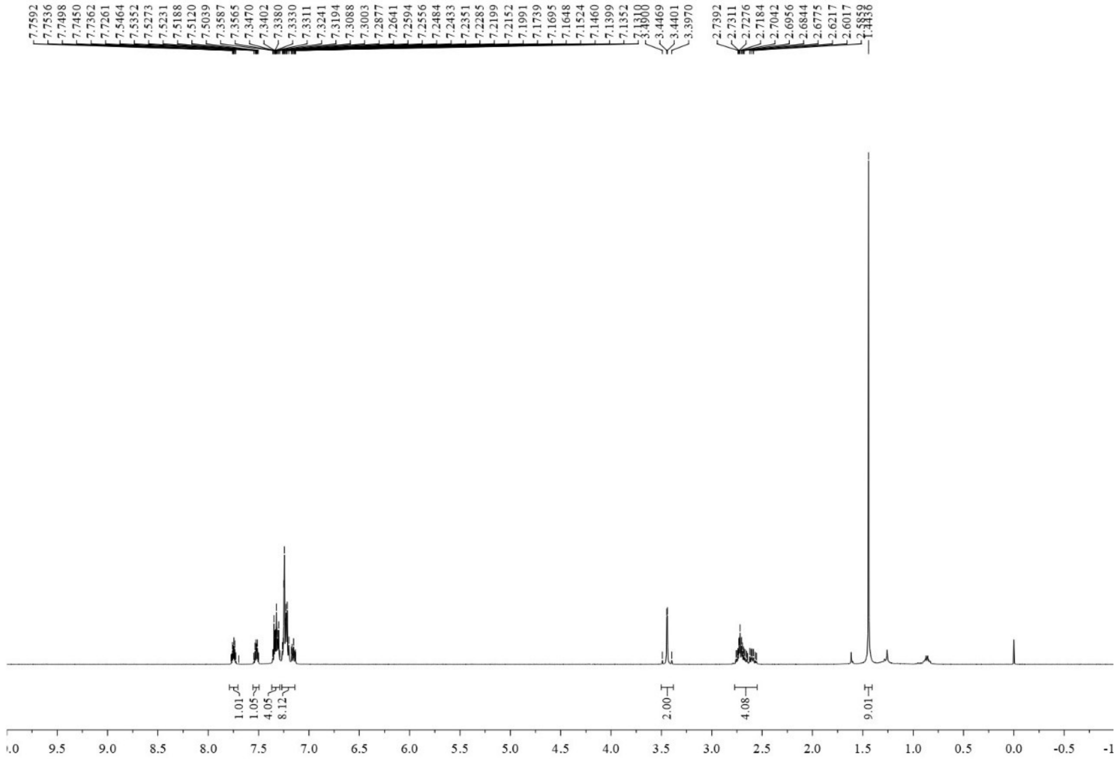

^{13}C NMR of **7h (100 MHz, CDCl$_3$)**

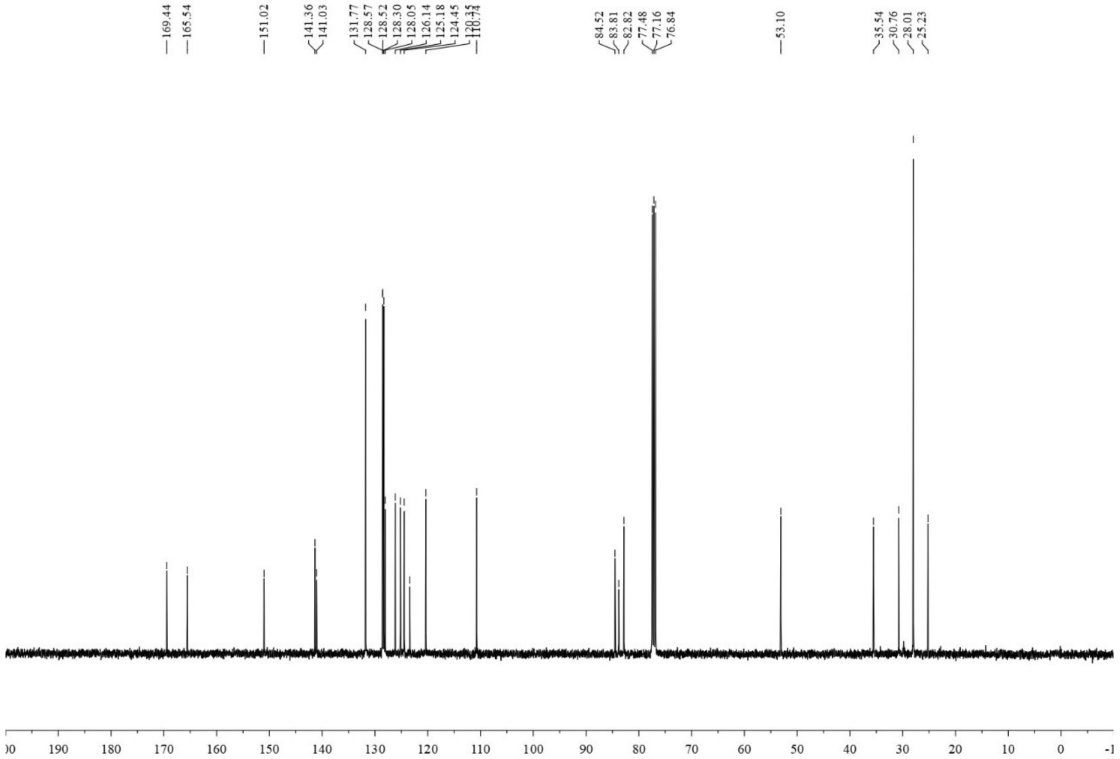

^{1}H NMR of **7i (400 MHz, CDCl$_3$)**

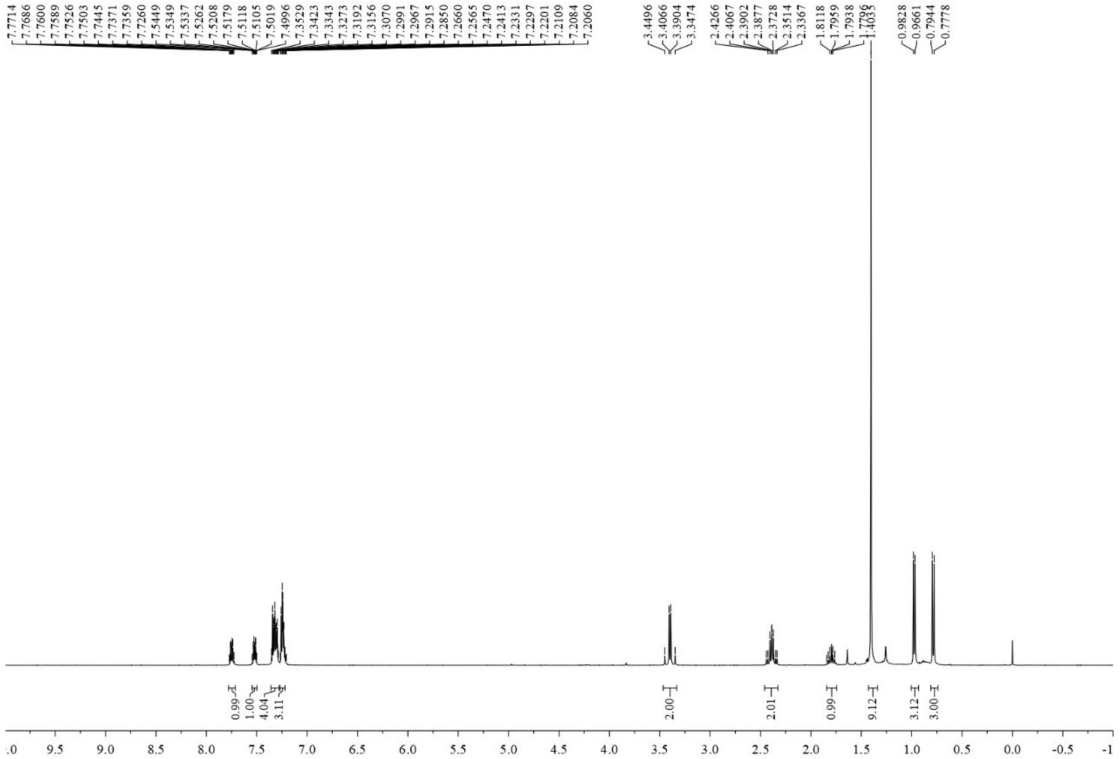

^{13}C NMR of **7i (100 MHz, CDCl$_3$)**

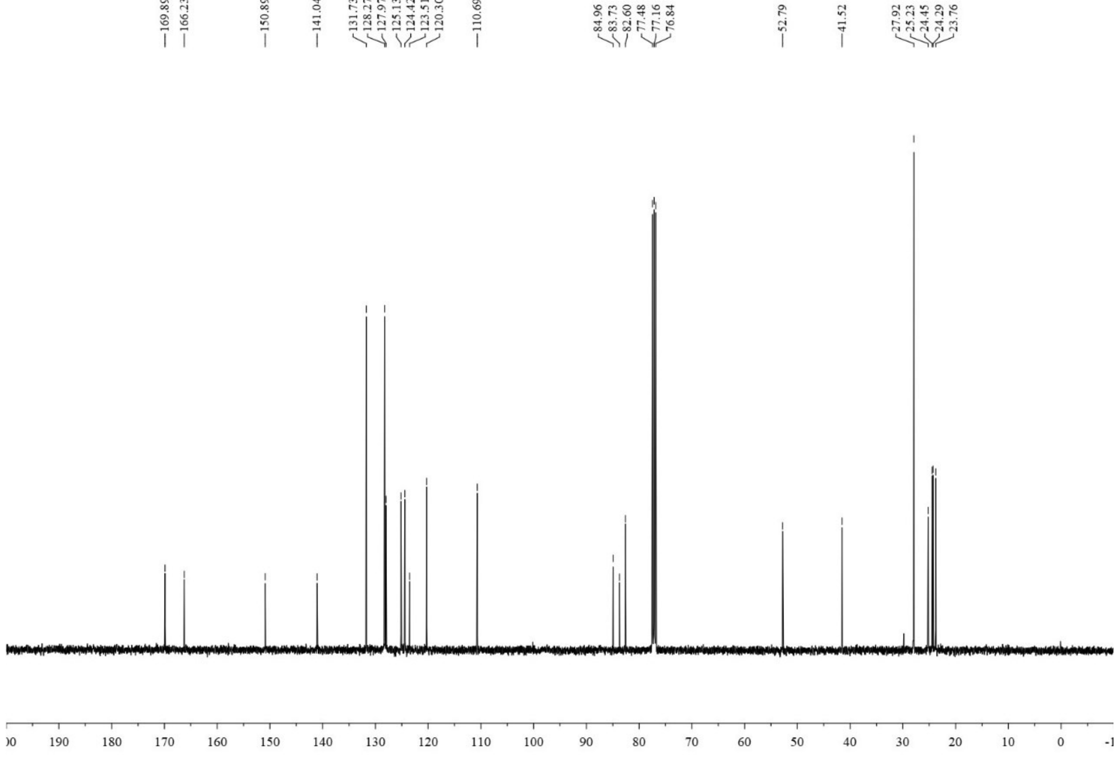

^{1}H NMR of **7j (400 MHz, $CDCl_3$)**

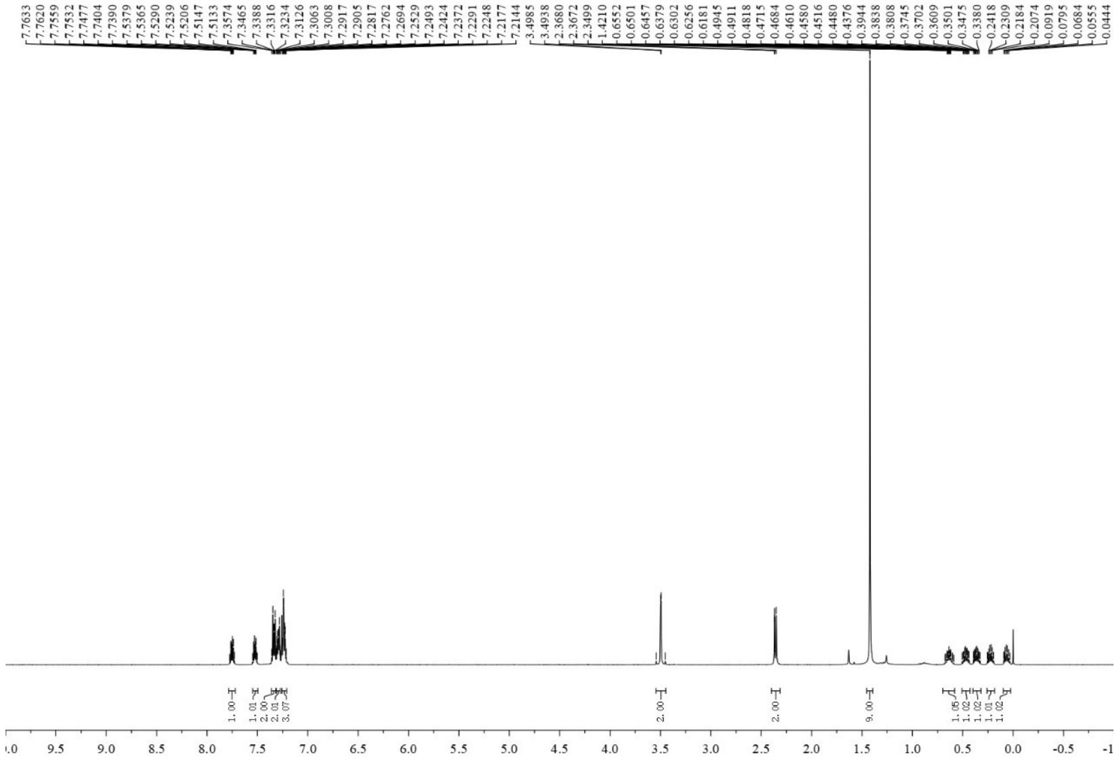

^{13}C NMR of **7j (100 MHz, $CDCl_3$)**

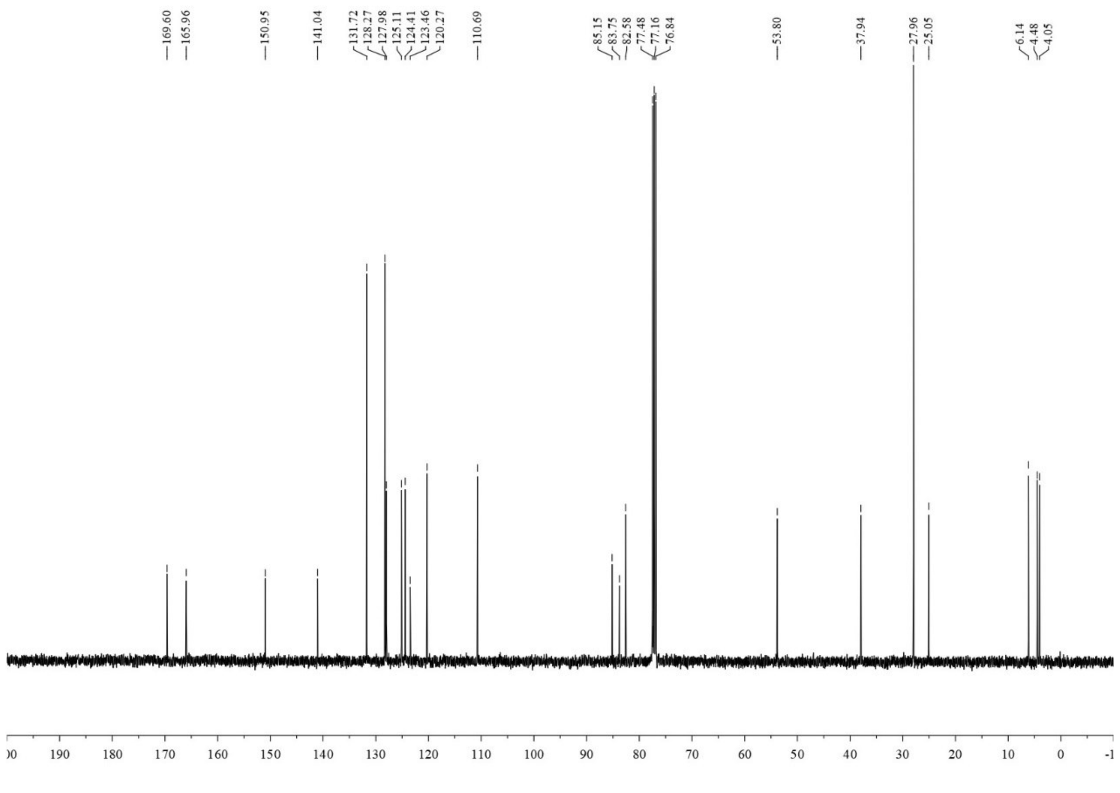

^{1}H NMR of **7k (500 MHz, CDCl$_3$)**

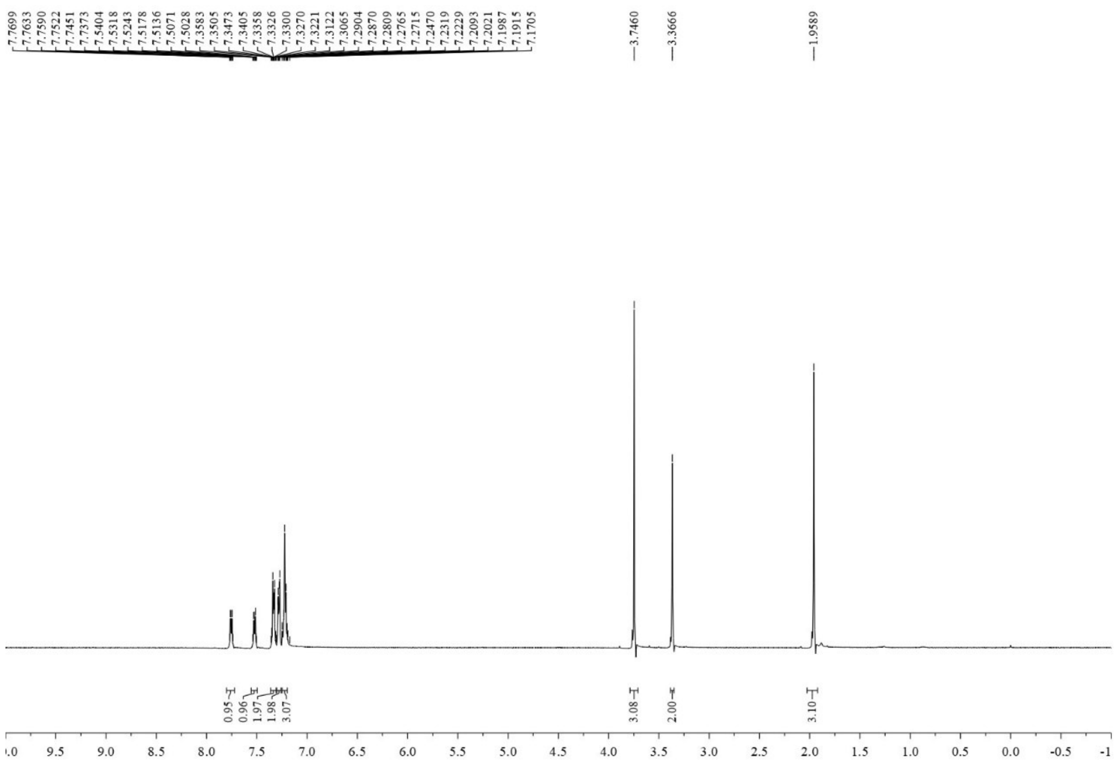

^{13}C NMR of **7k (125 MHz, CDCl$_3$)**

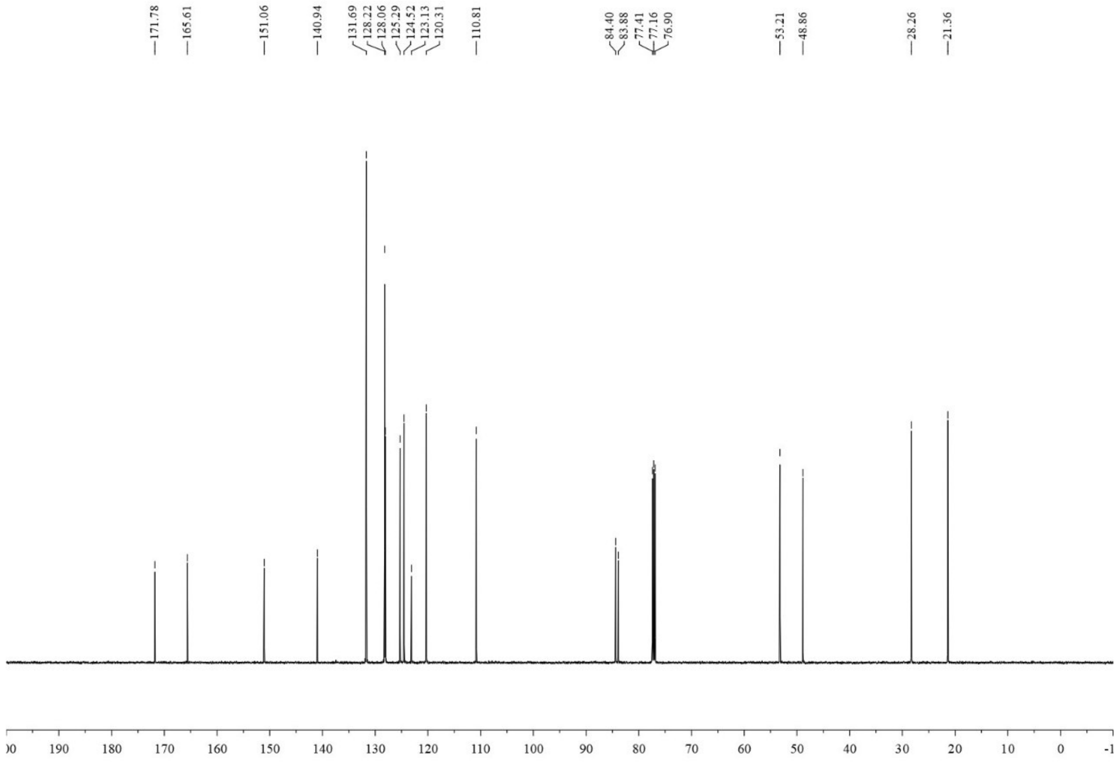

^{1}H NMR of **7l (500 MHz, CDCl$_3$)**

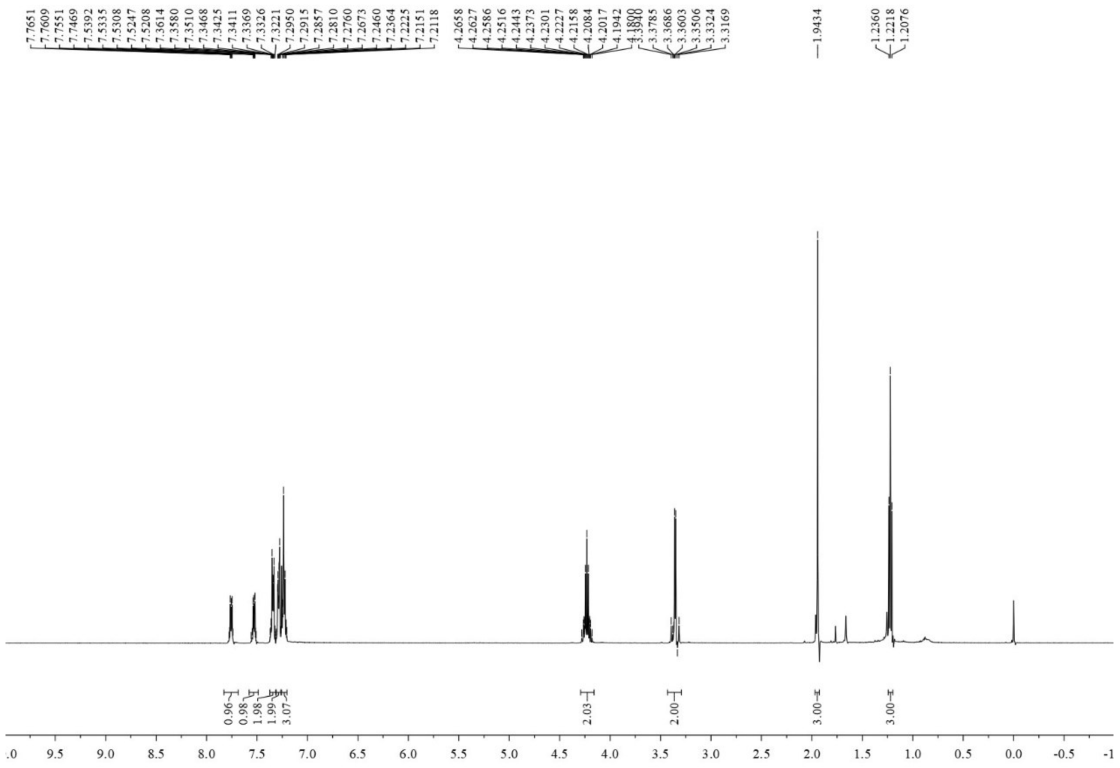

^{13}C NMR of **7l (125 MHz, CDCl$_3$)**

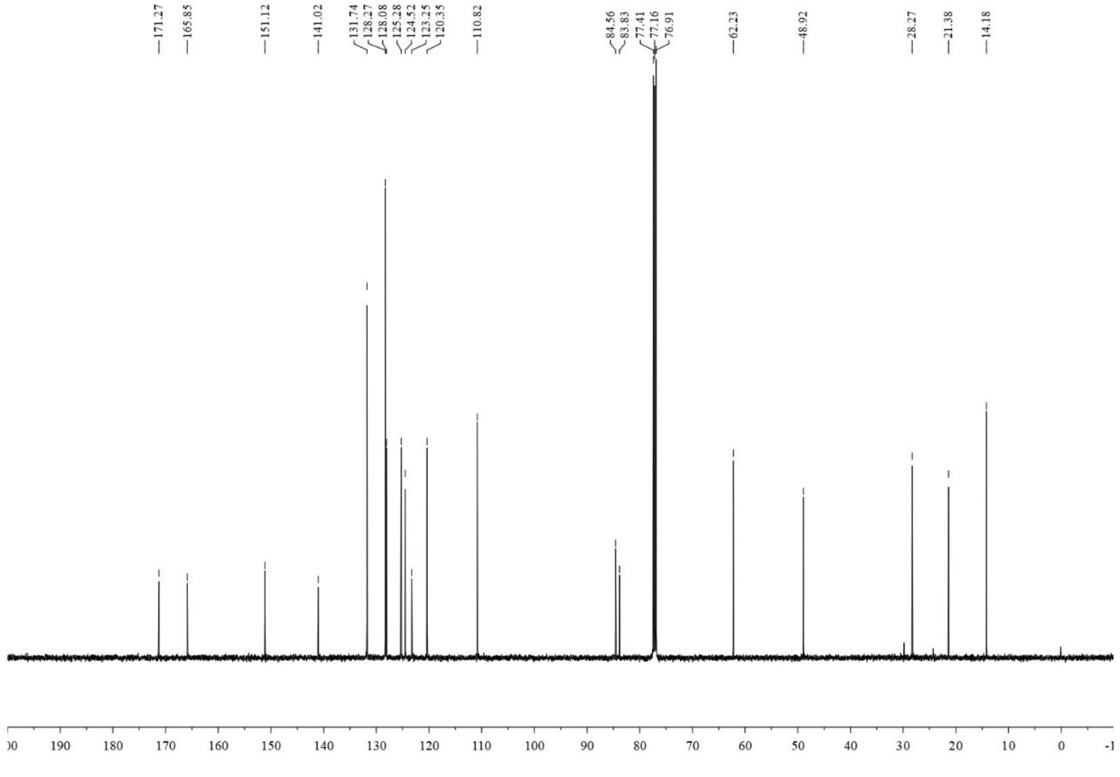

^{1}H NMR of **7m (500 MHz, $CDCl_3$)**

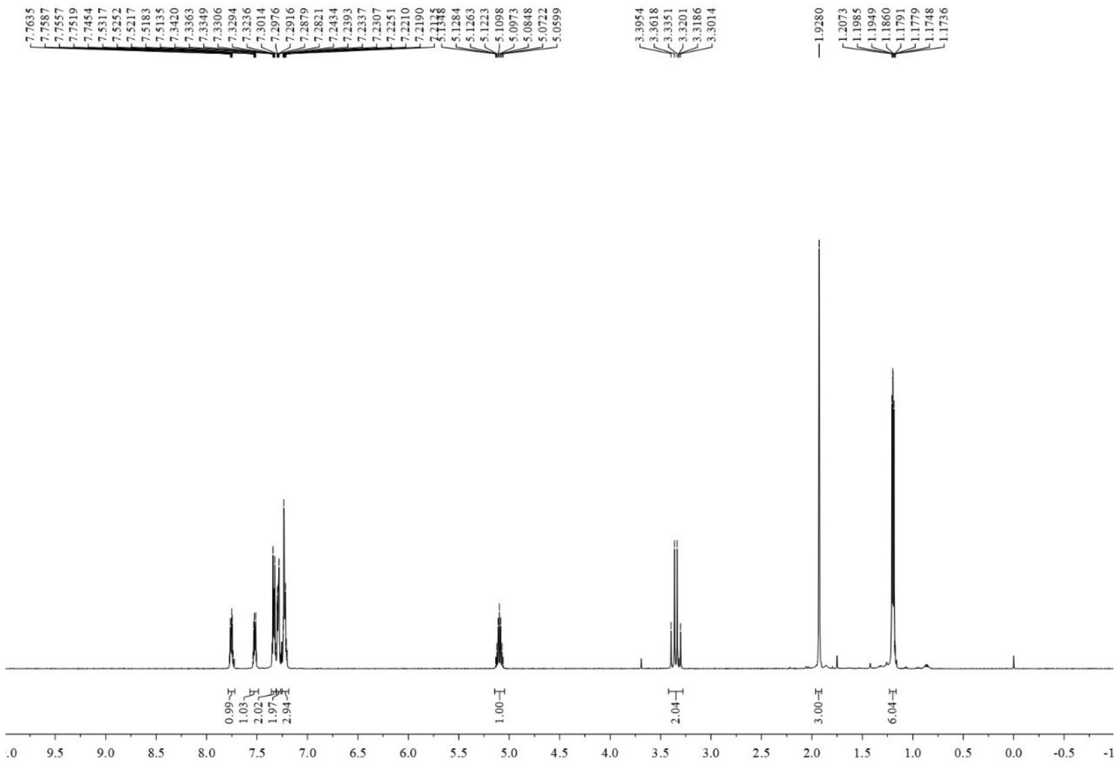

^{13}C NMR of **7m (125 MHz, $CDCl_3$)**

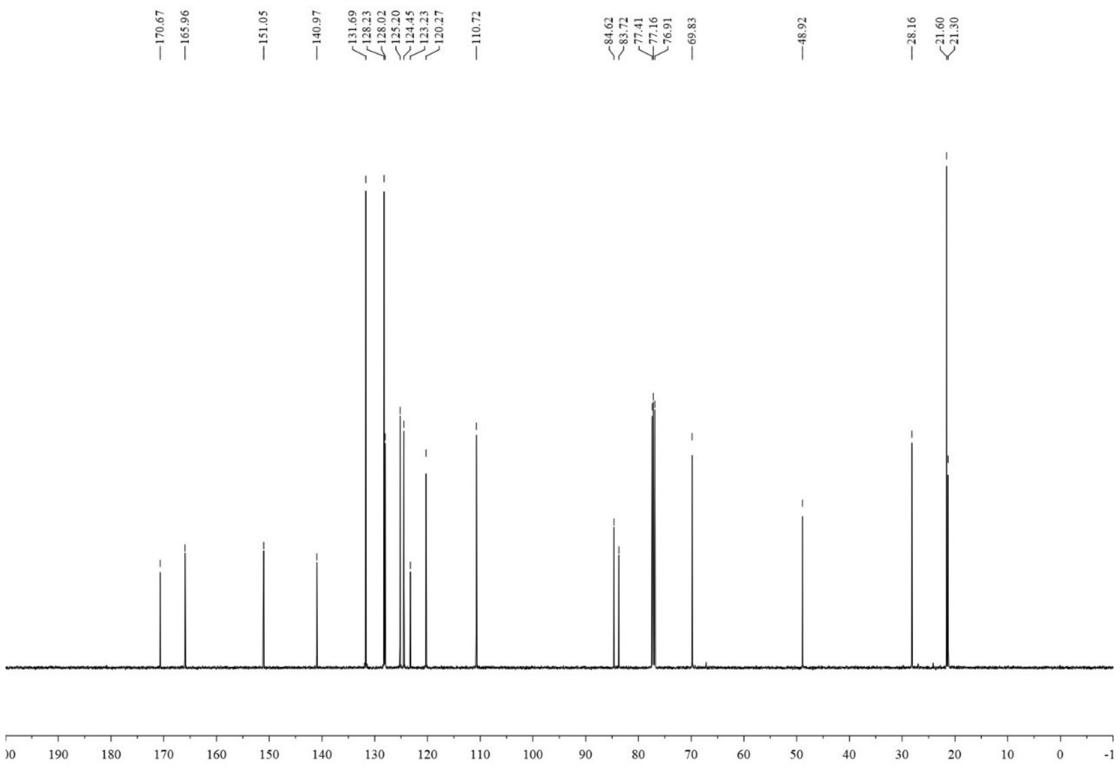

^{1}H NMR of **7n (400 MHz, CDCl$_3$)**

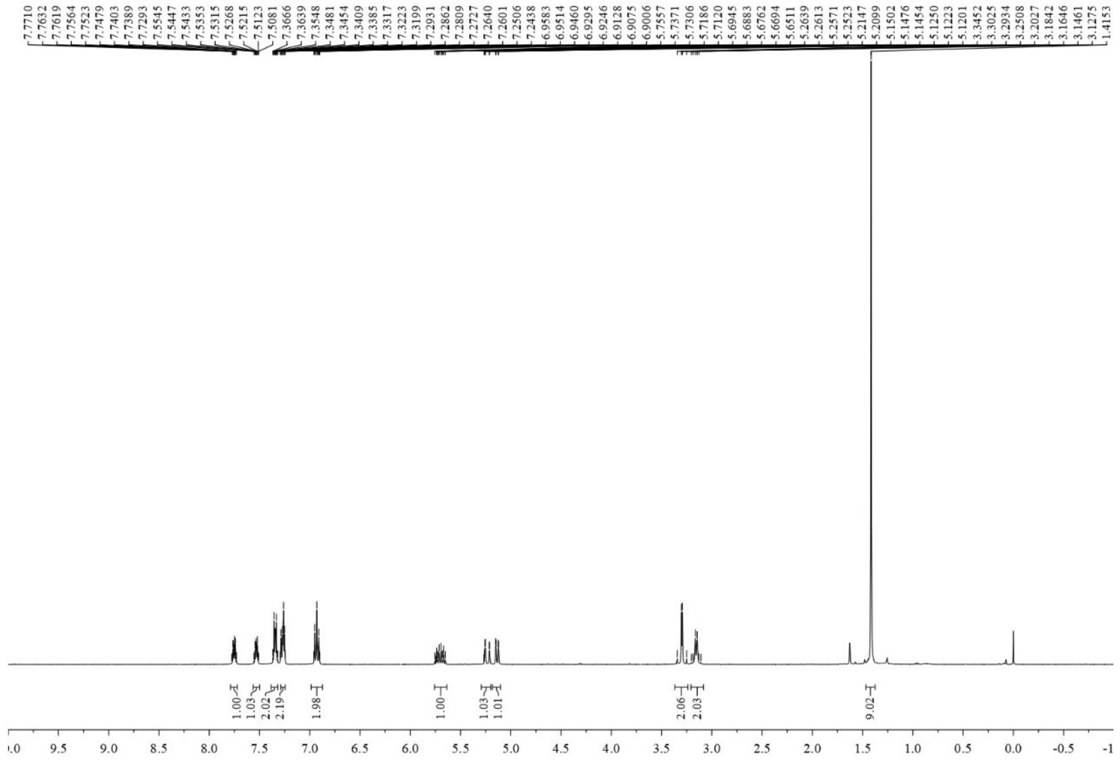

^{13}C NMR of **7n (100 MHz, CDCl$_3$)**

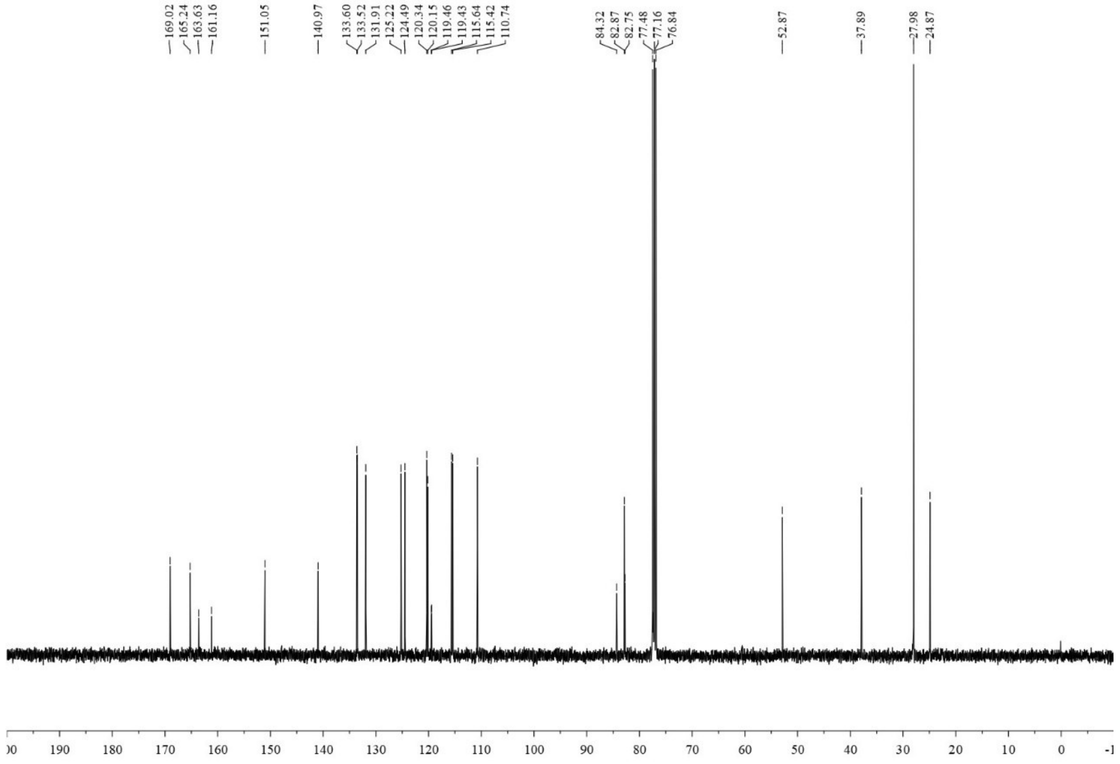

^{19}F NMR of **7n (375 MHz, CDCl$_3$)**

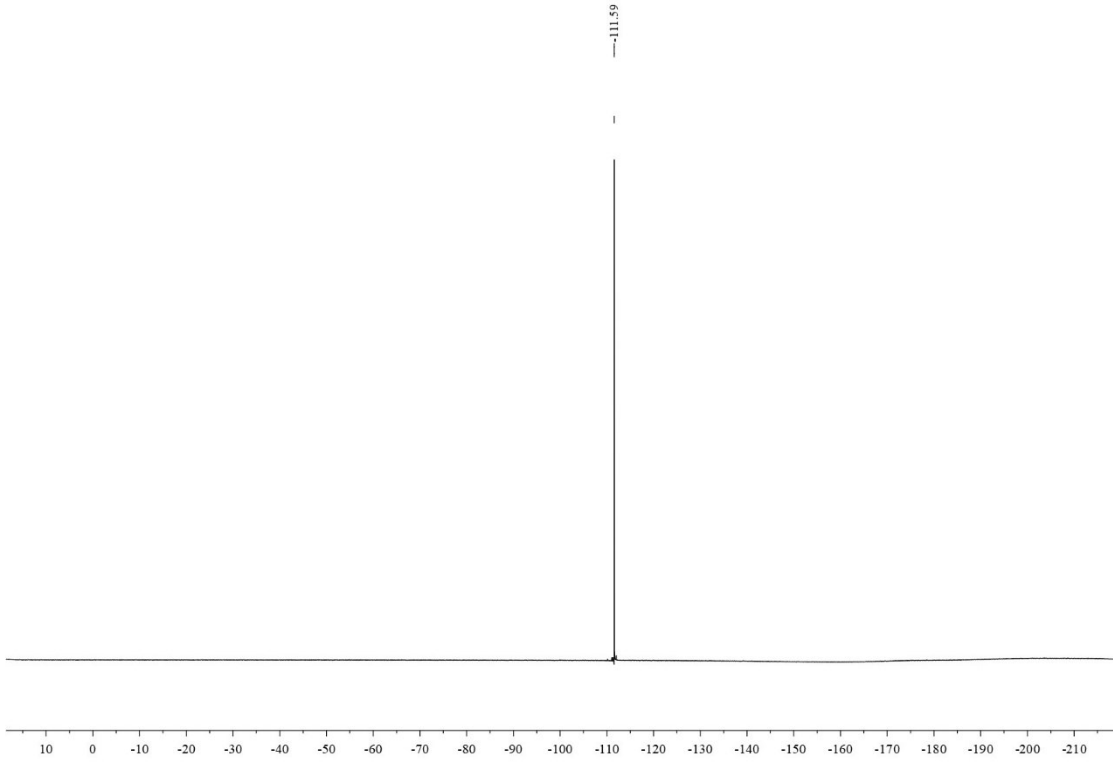

^{1}H NMR of **7o (500 MHz, CDCl$_3$)**

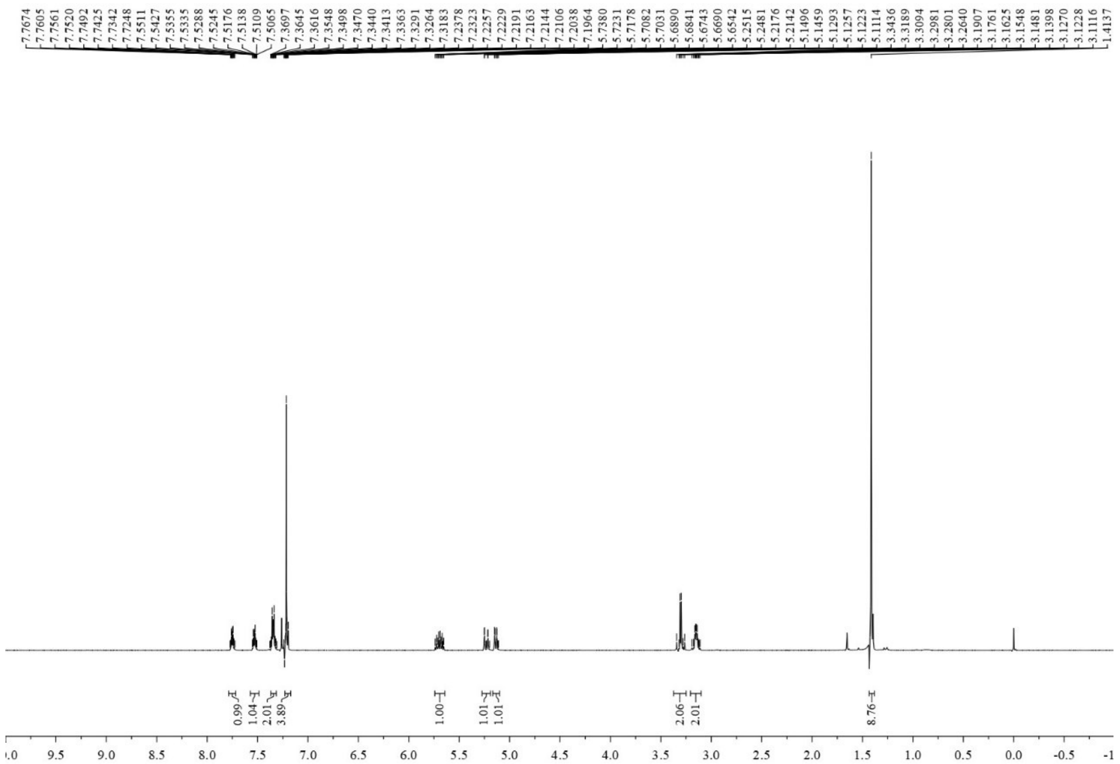

^{13}C NMR of **7o (125 MHz, CDCl$_3$)**

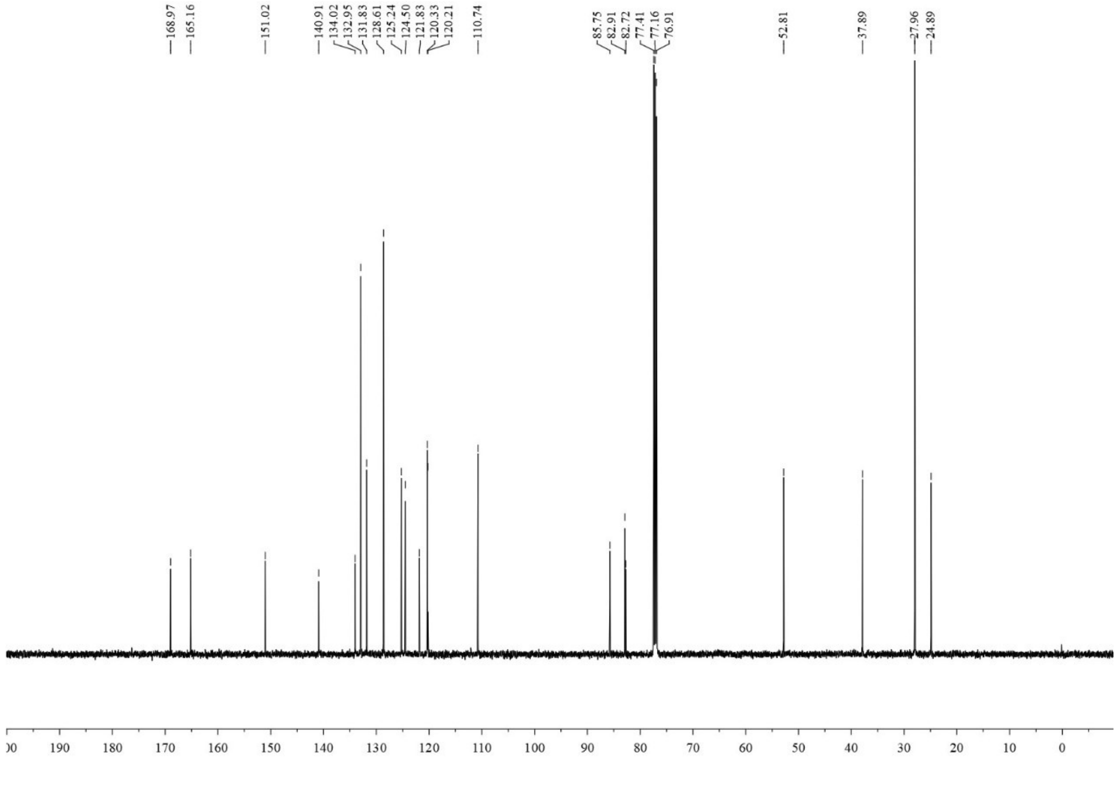

^{1}H NMR of **7p (500 MHz, CDCl$_3$)**

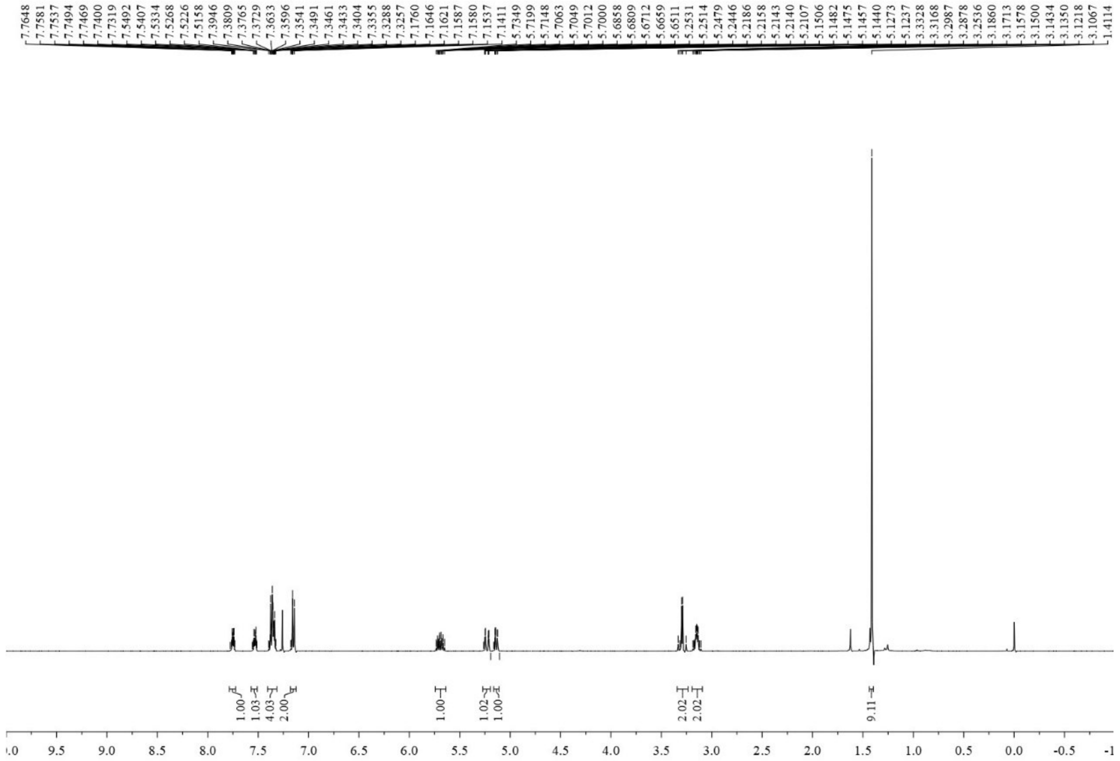

^{13}C NMR of **7p (125 MHz, CDCl$_3$)**

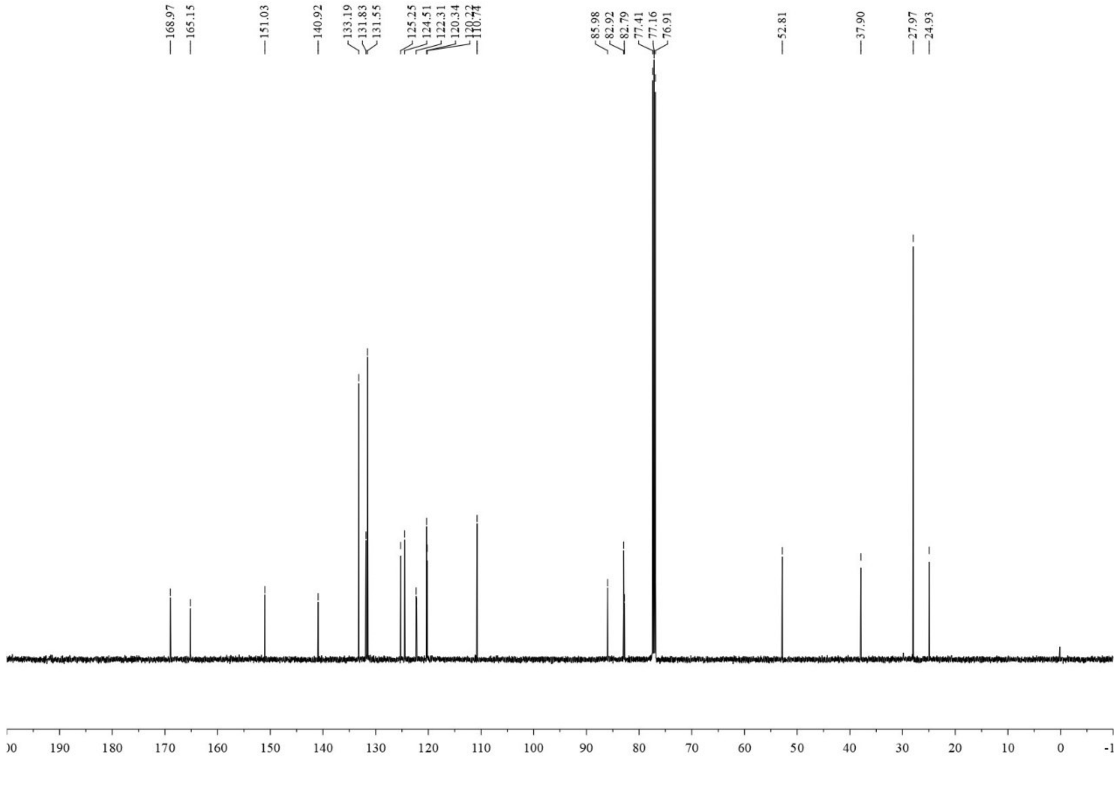

^{1}H NMR of **7q (400 MHz, CDCl$_3$)**

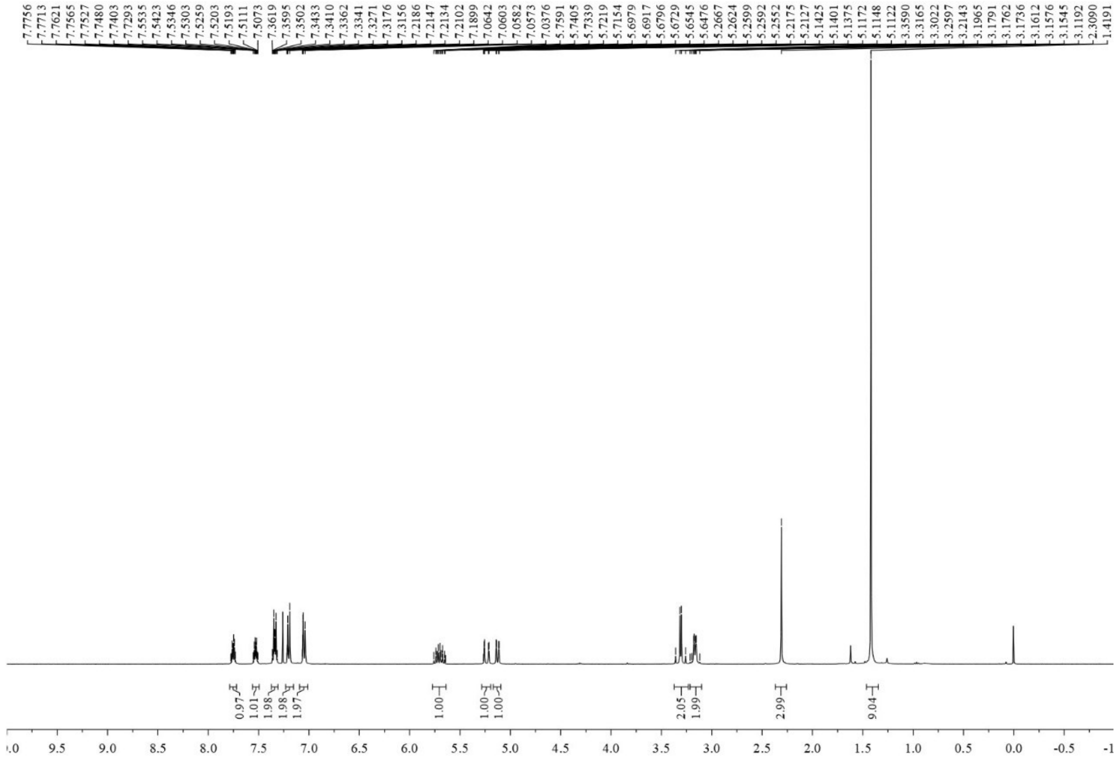

^{13}C NMR of **7q (100 MHz, CDCl$_3$)**

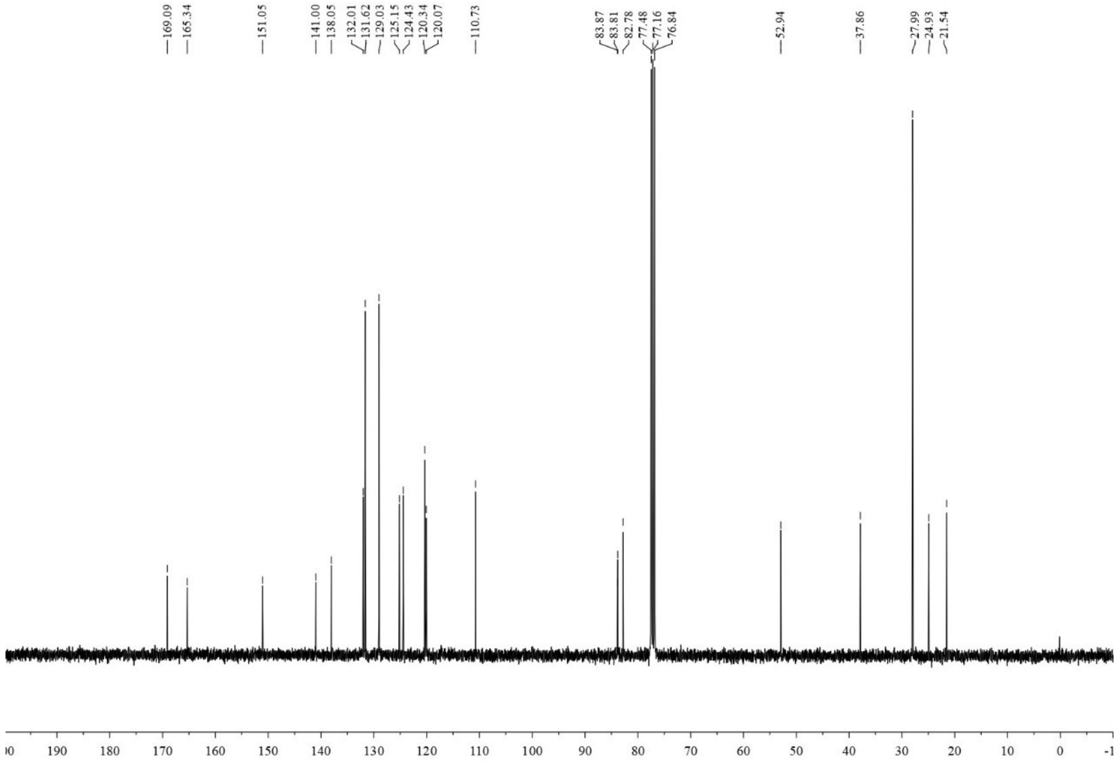

^{1}H NMR of **7r (400 MHz, CDCl$_3$)**

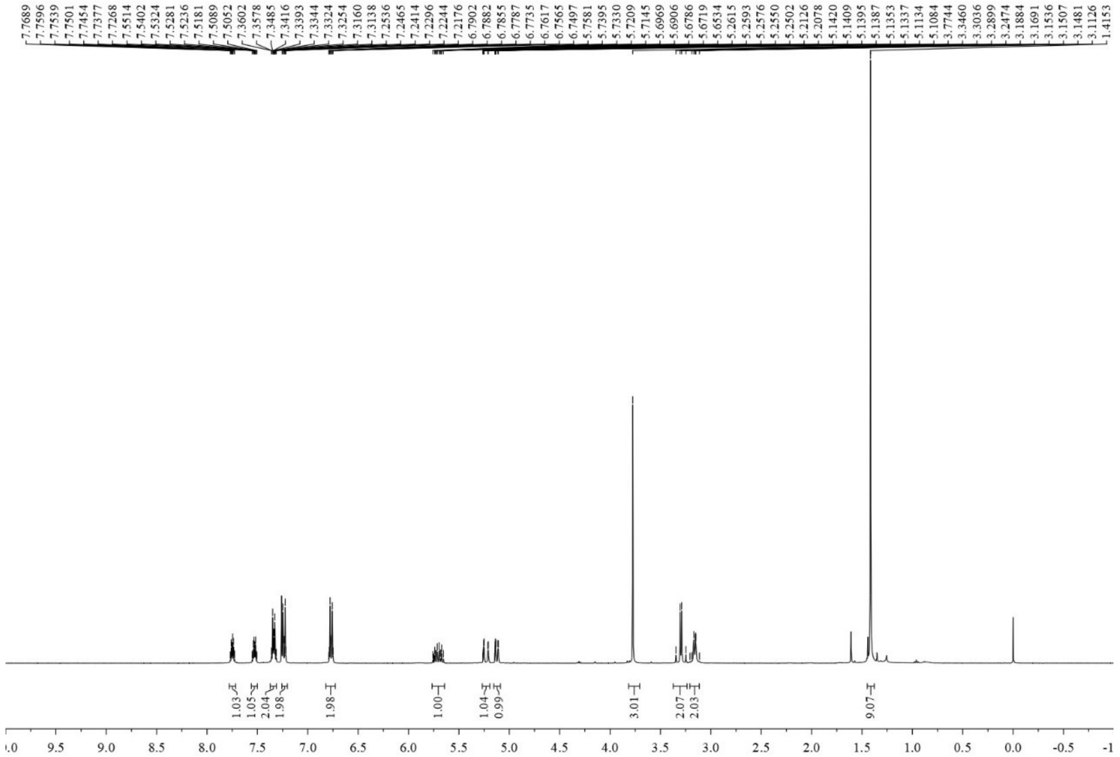

^{13}C NMR of **7r (100 MHz, CDCl$_3$)**

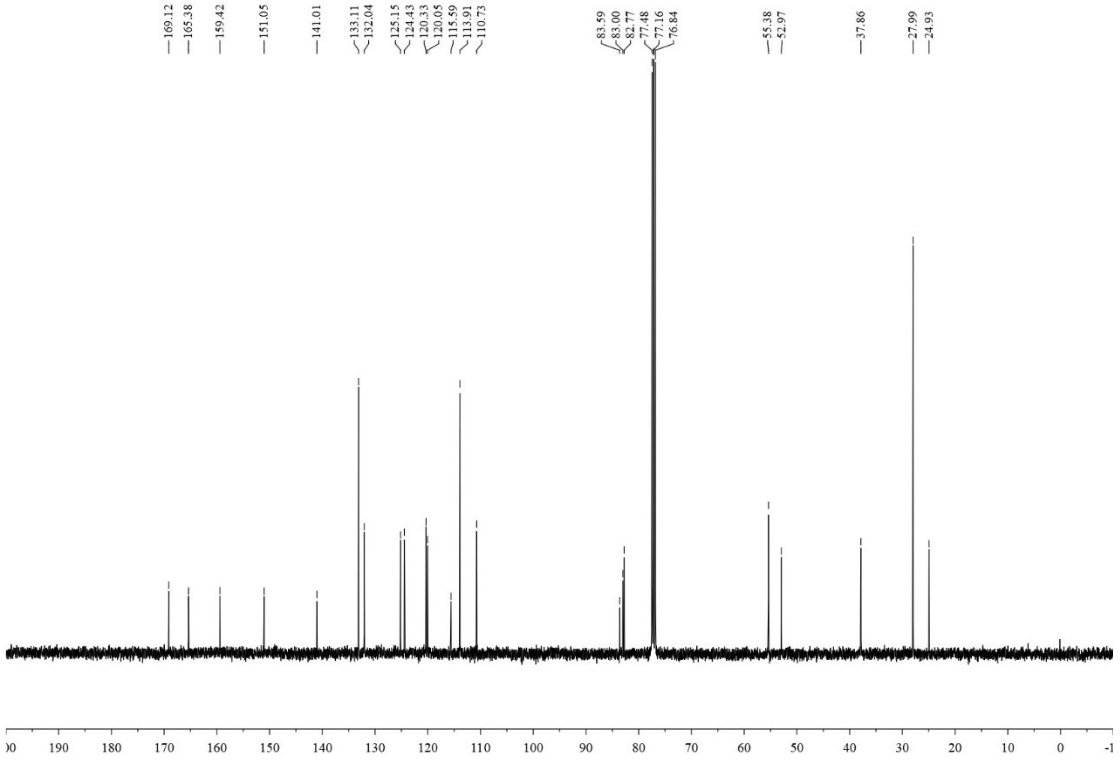

^{1}H NMR of **7s** (400 MHz, $CDCl_3$)

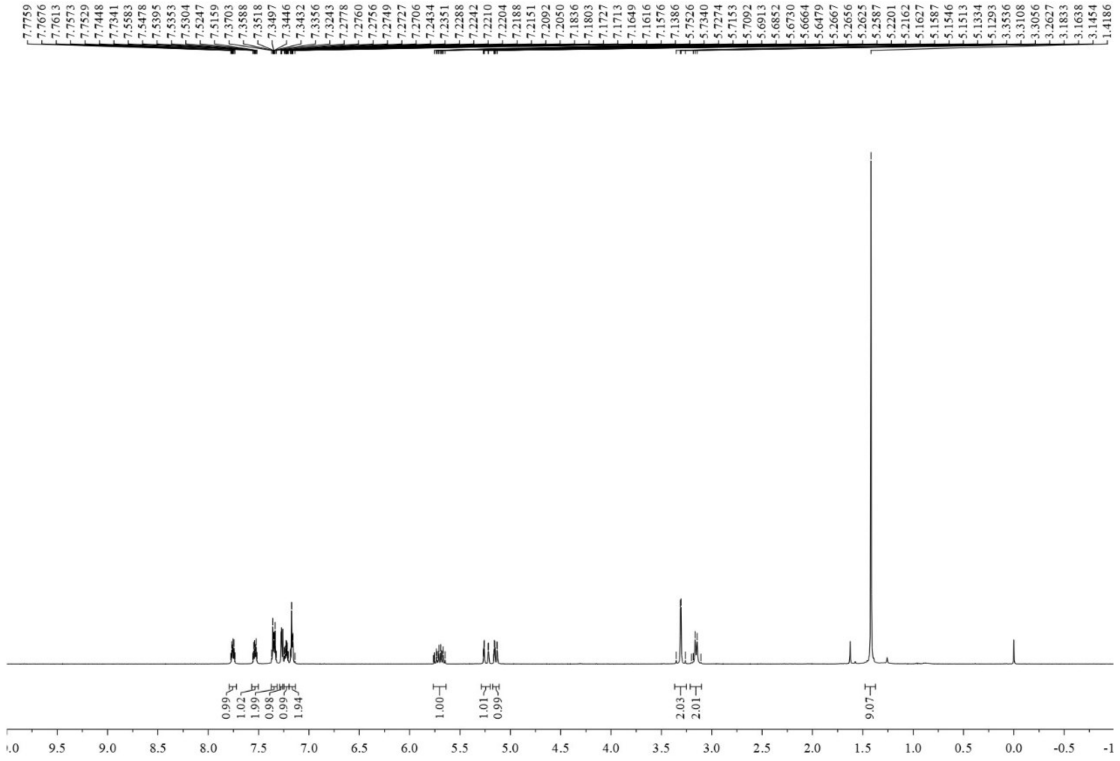

^{13}C NMR of **7s** (100 MHz, $CDCl_3$)

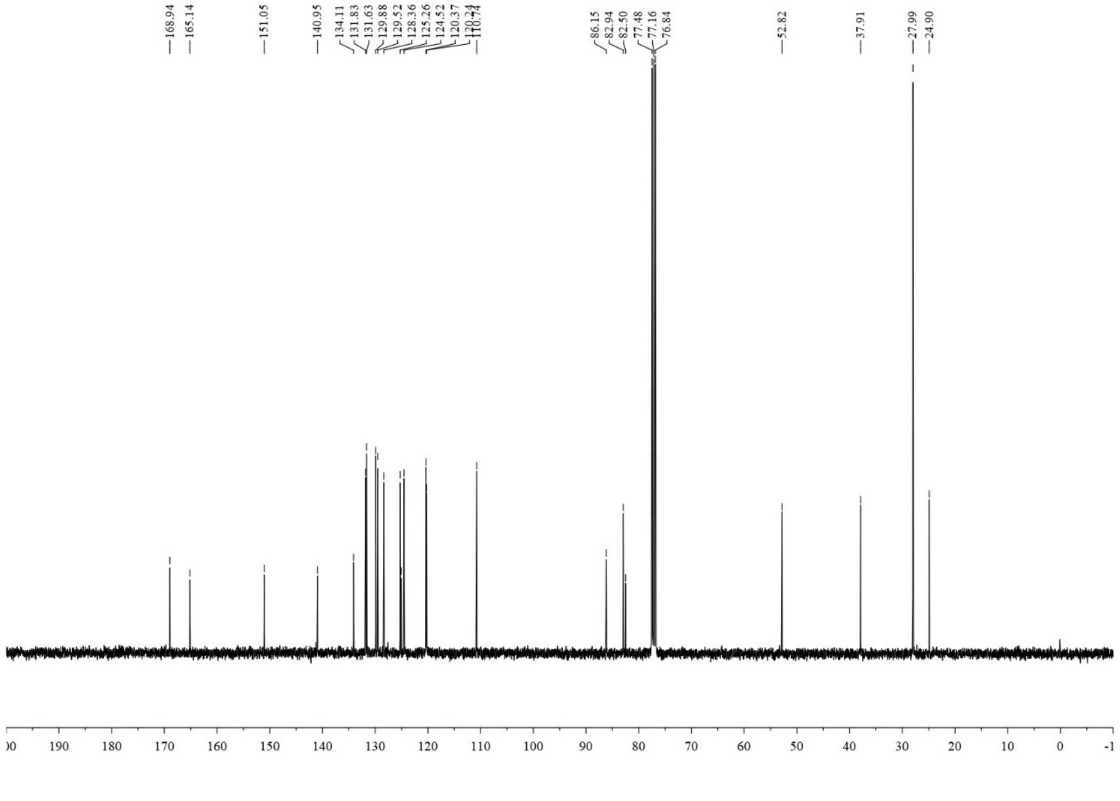

^{1}H NMR of **7t (400 MHz, $CDCl_3$)**

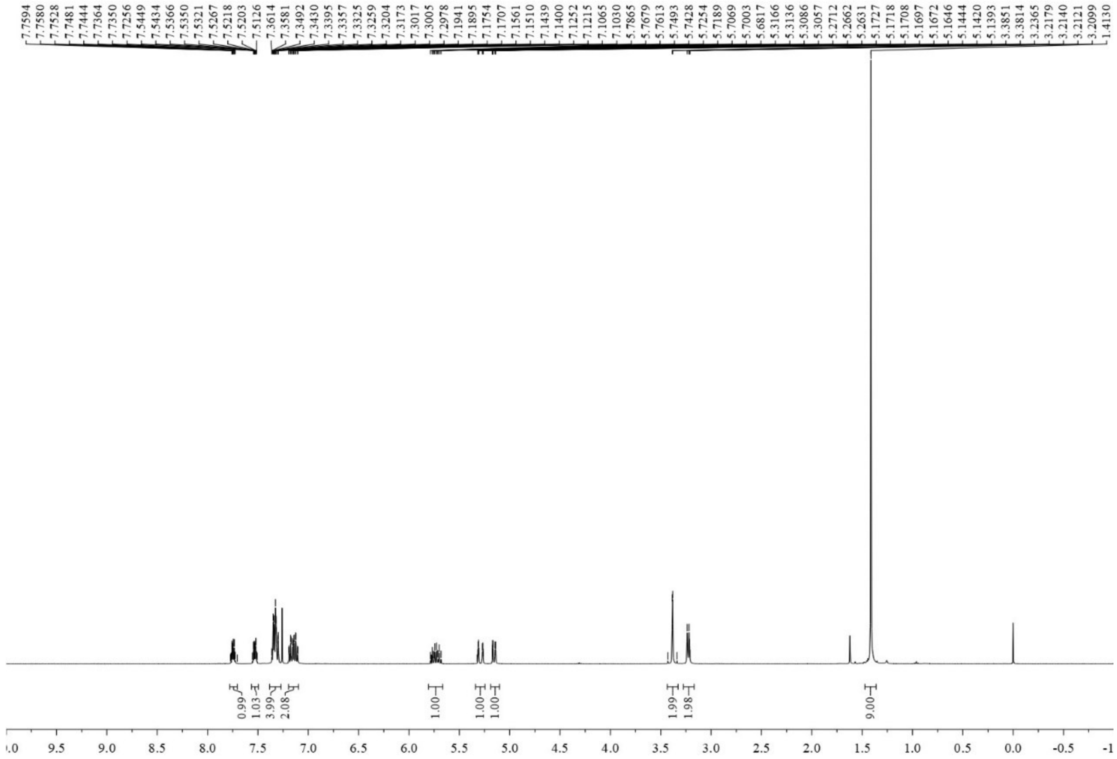

^{13}C NMR of **7t (100 MHz, $CDCl_3$)**

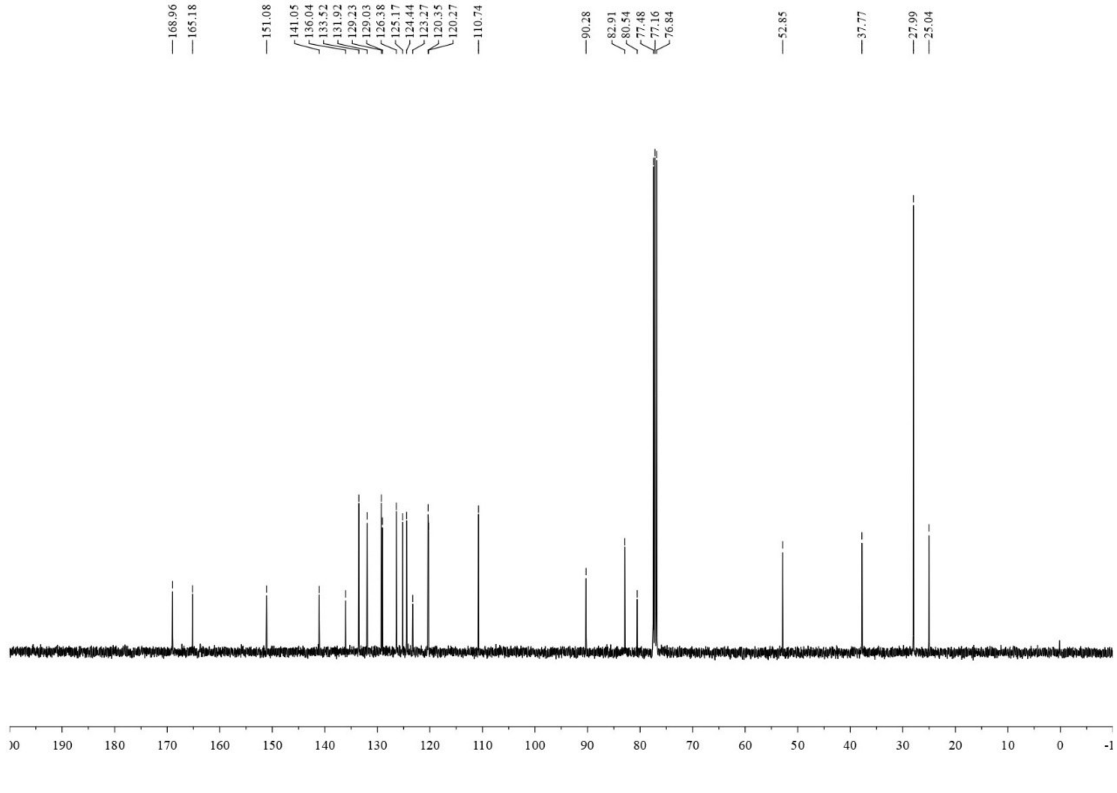

^{1}H NMR of **7u (500 MHz, CDCl$_3$)**

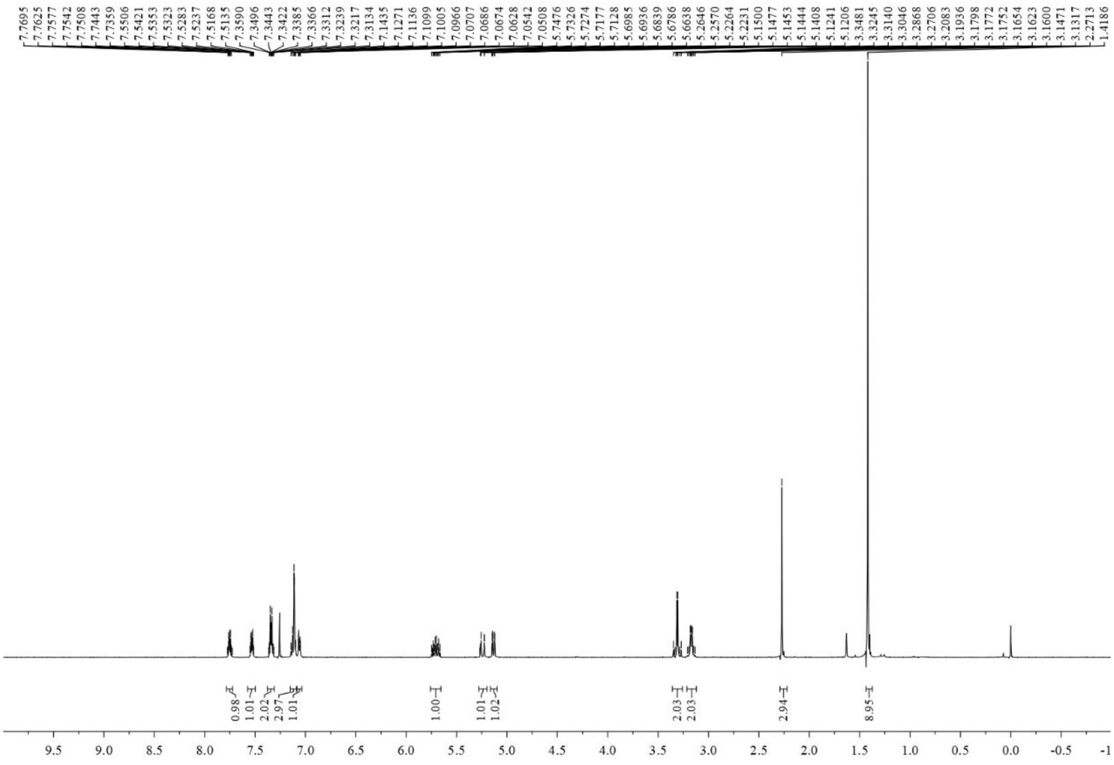

^{13}C NMR of **7u (125 MHz, CDCl$_3$)**

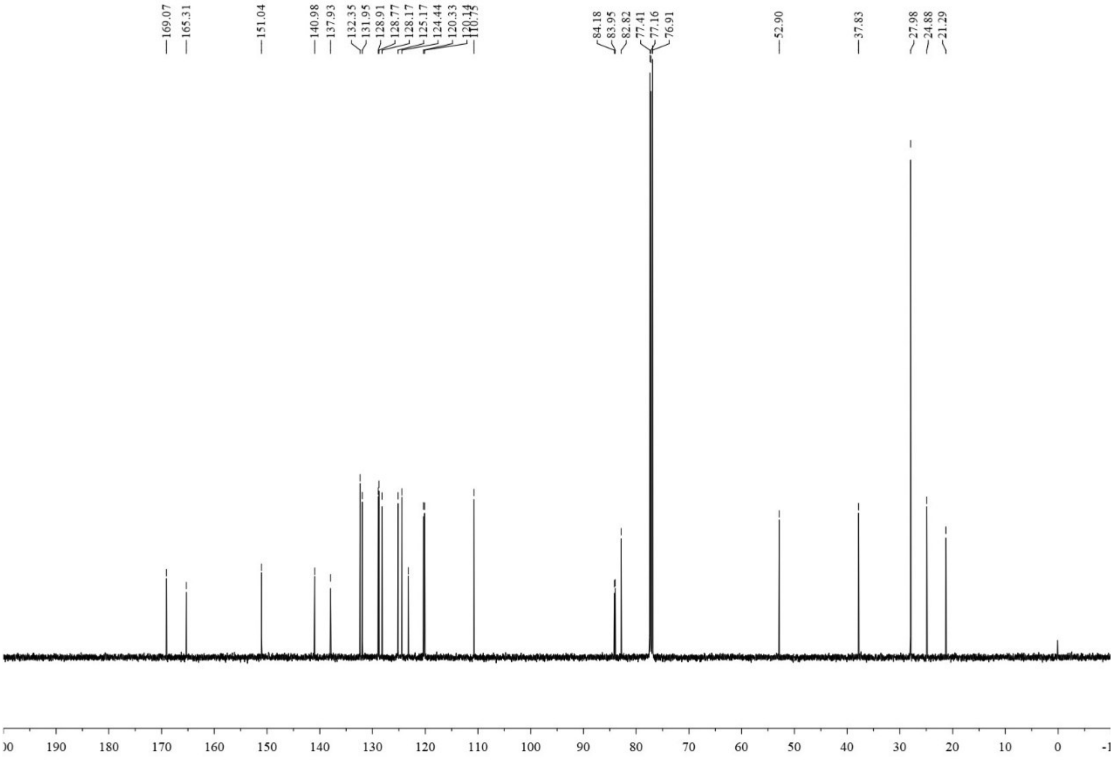

^{1}H NMR of **7v (400 MHz, CDCl$_3$)**

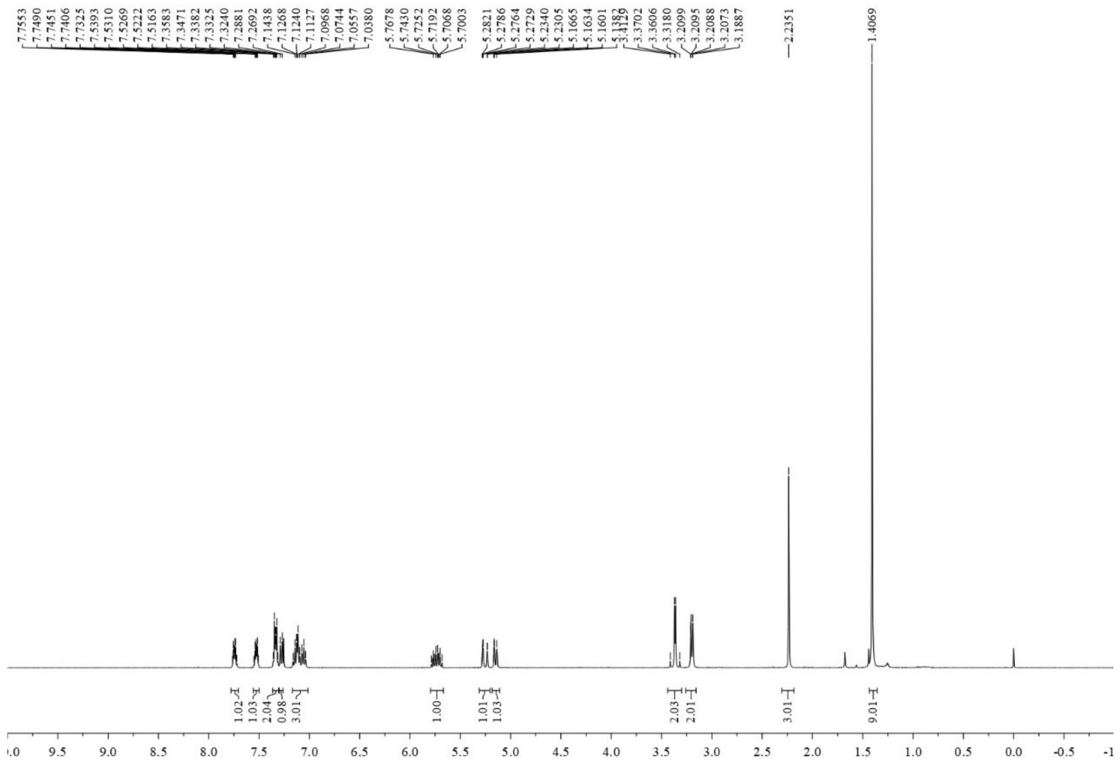

^{13}C NMR of **7v (100 MHz, CDCl$_3$)**

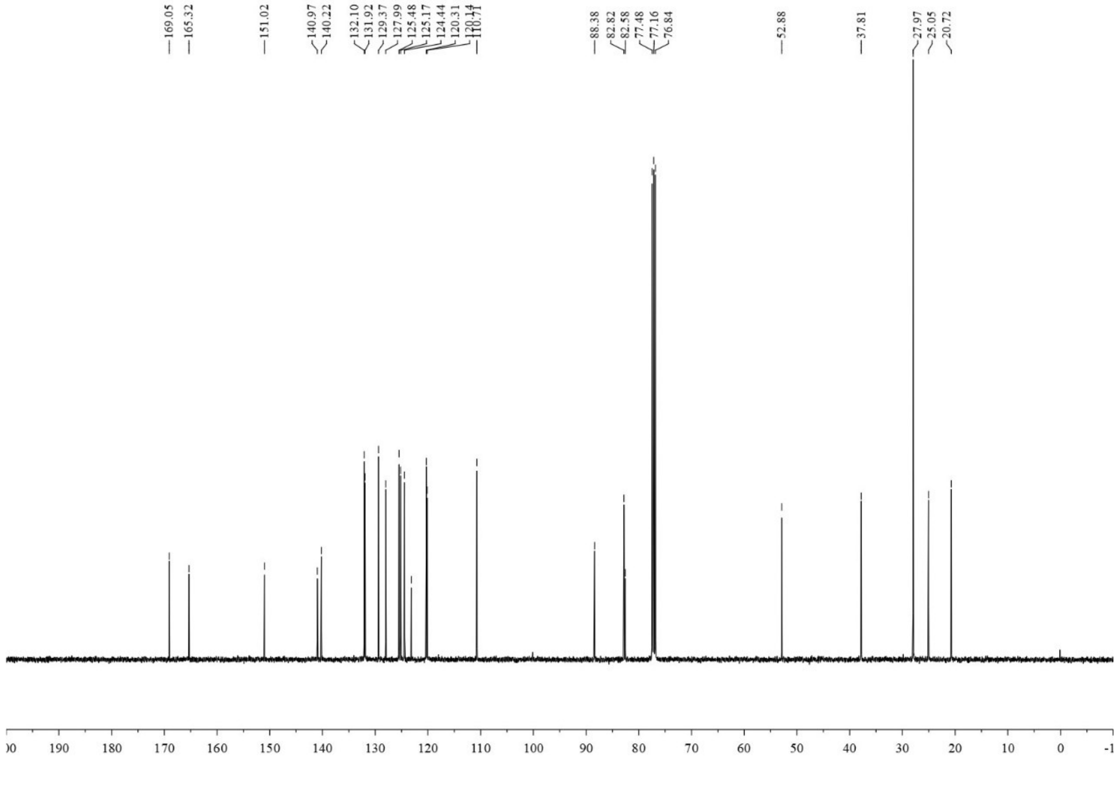

^{1}H NMR of **7w (500 MHz, $CDCl_3$)**

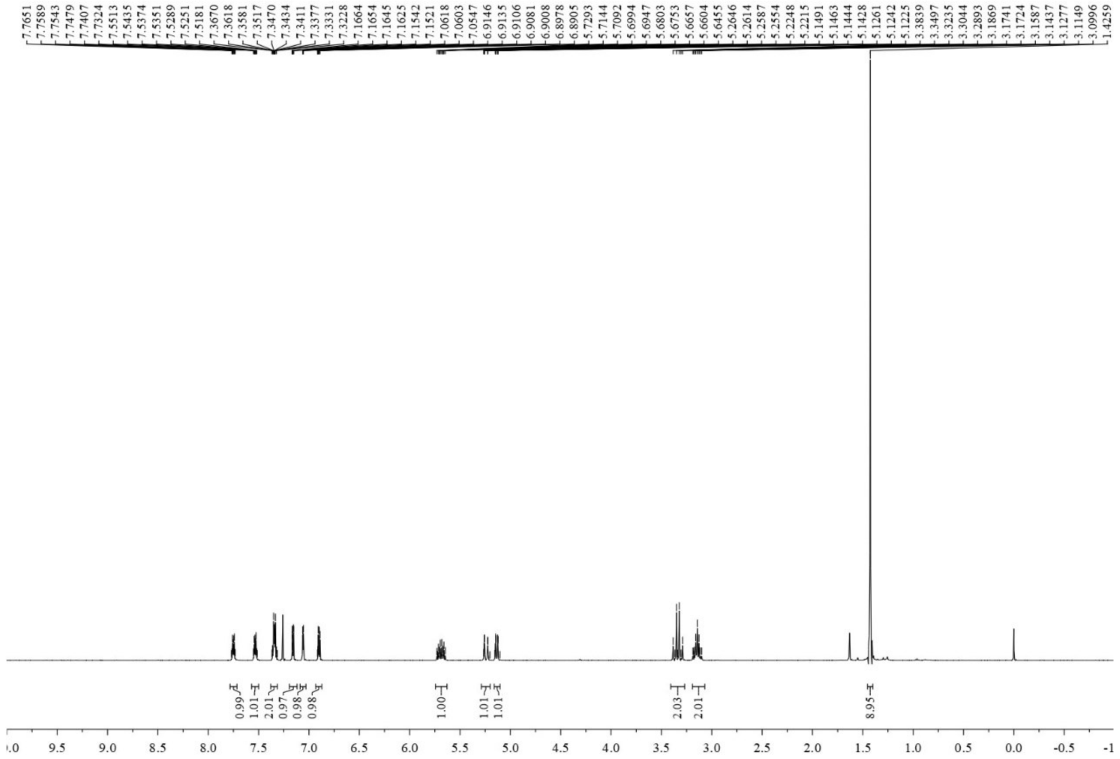

^{13}C NMR of **7w (125 MHz, $CDCl_3$)**

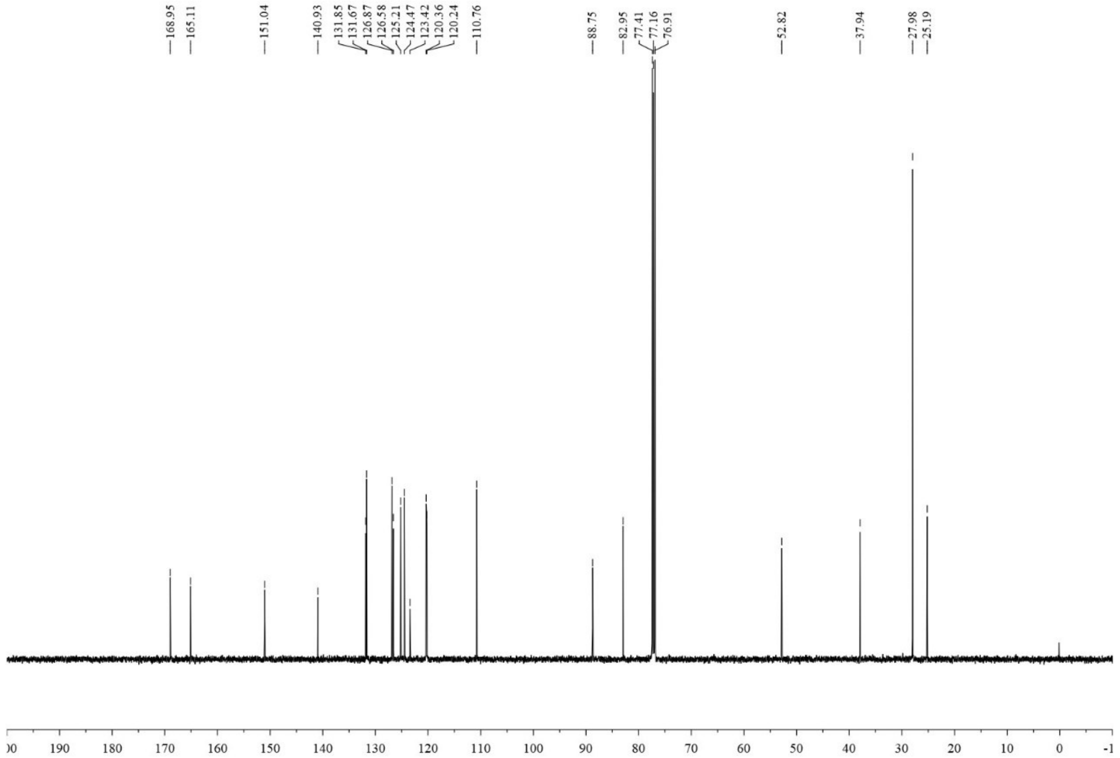

^{1}H NMR of **7x (500 MHz, $CDCl_3$)**

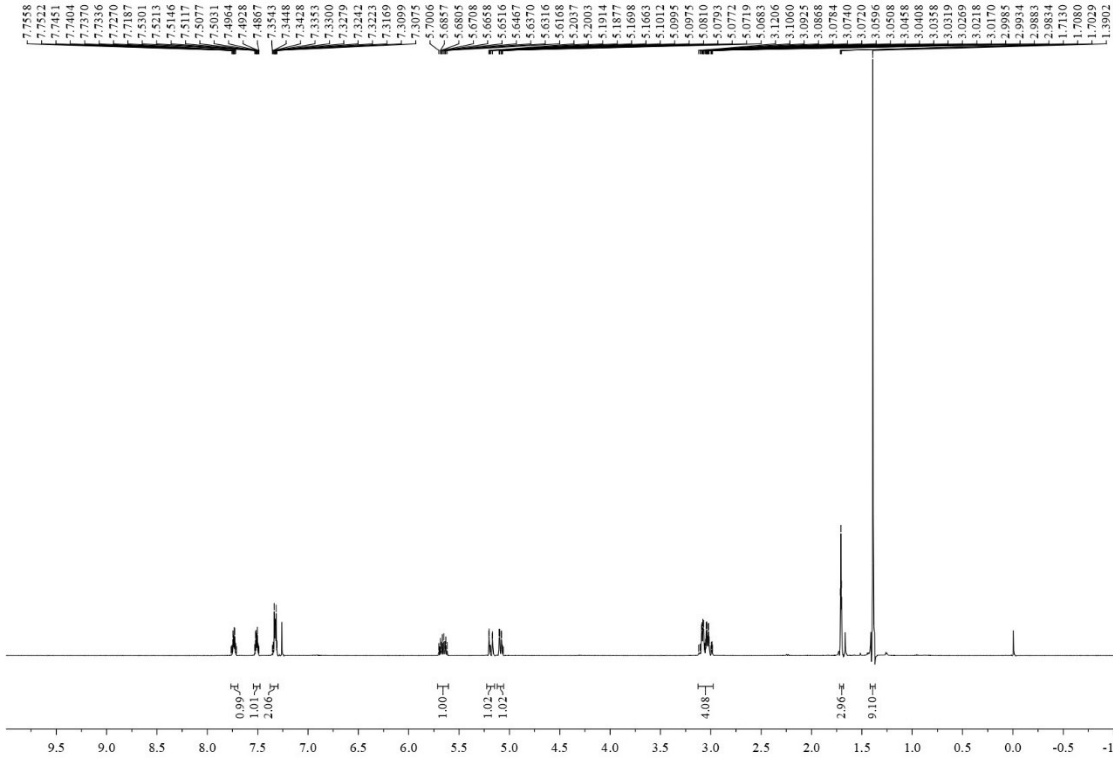

^{13}C NMR of **7x (125 MHz, $CDCl_3$)**

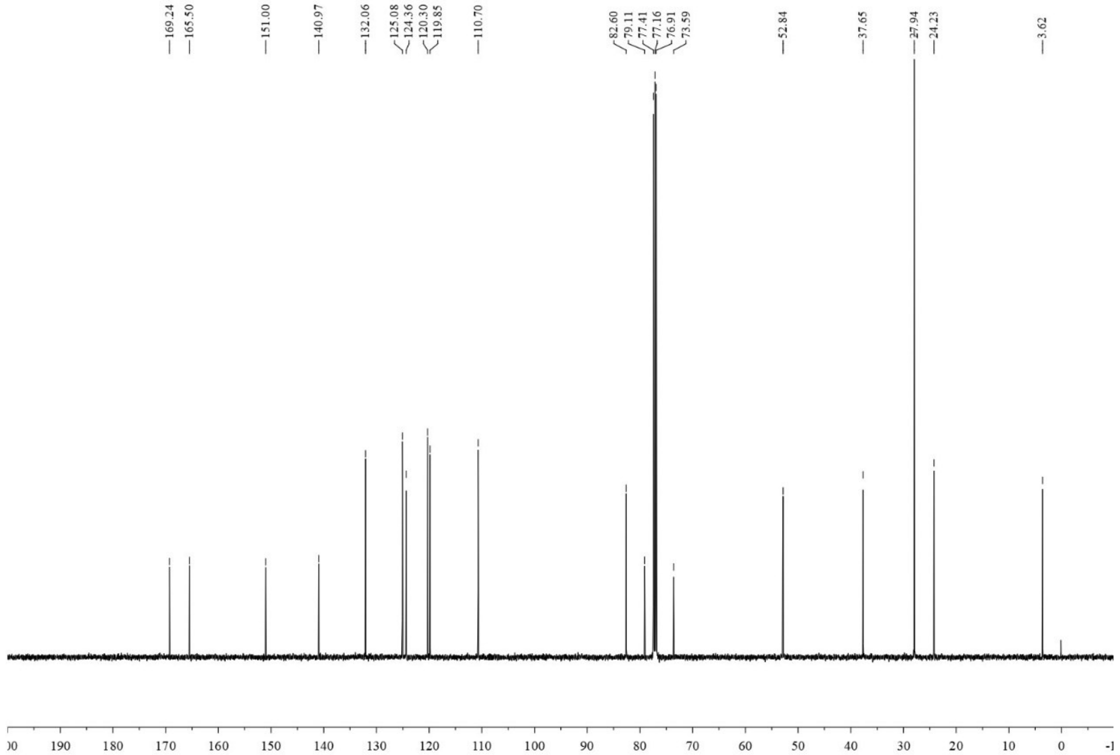

^{1}H NMR of **7y (500 MHz, CDCl$_3$)**

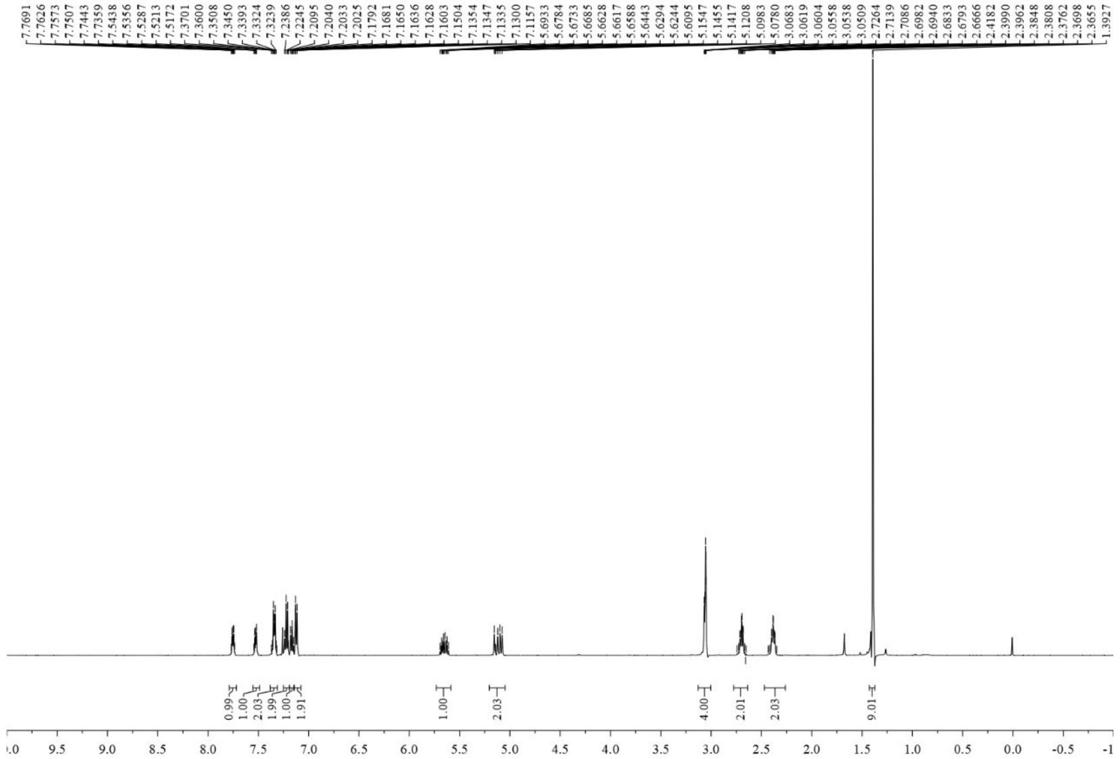

^{13}C NMR of **7y (125 MHz, CDCl$_3$)**

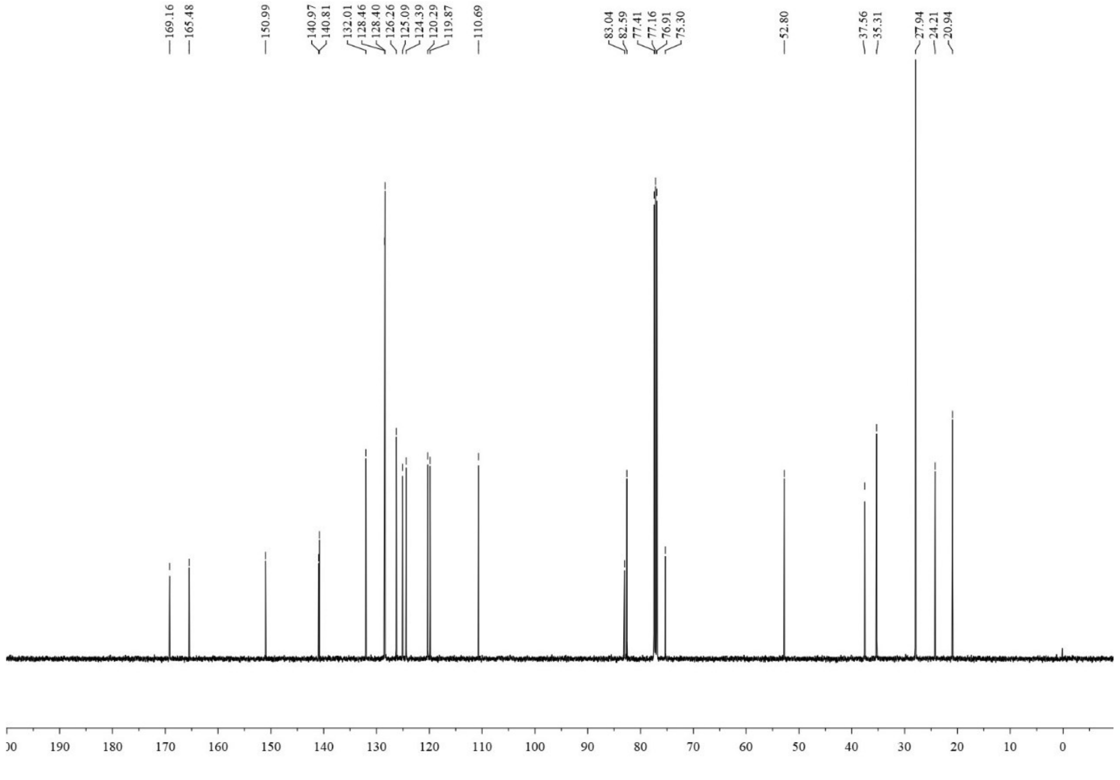

^{1}H NMR of **7z (500 MHz, CDCl$_3$)**

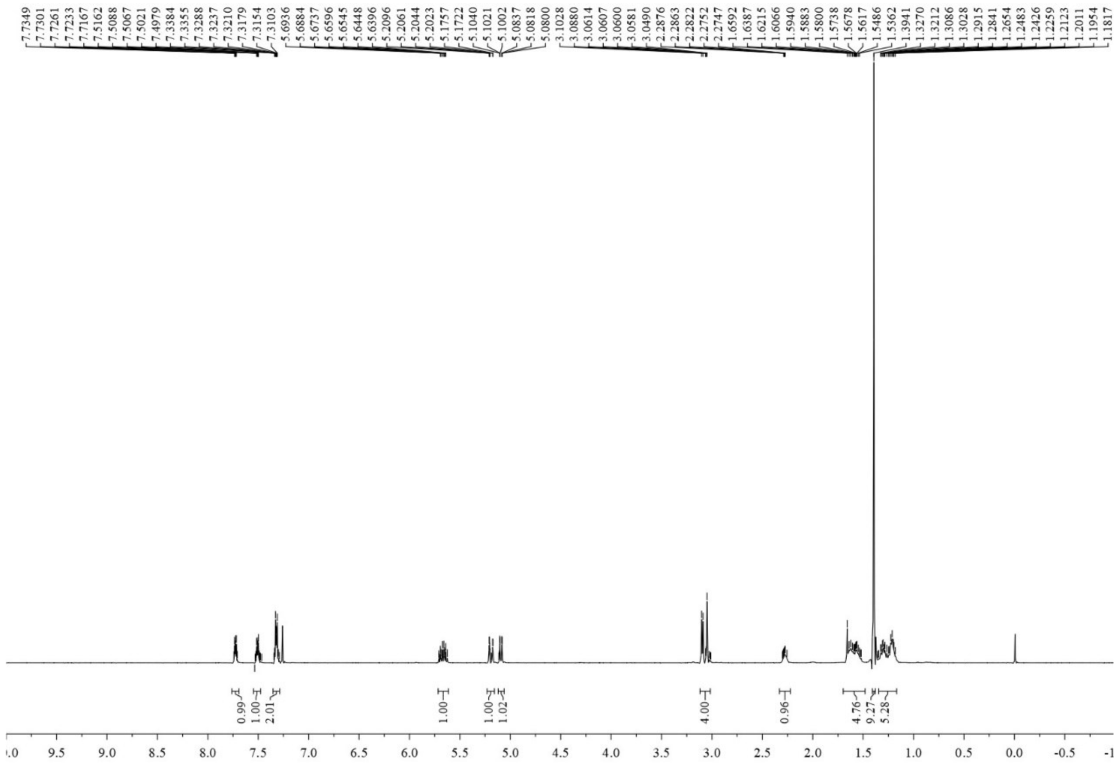

^{13}C NMR of **7z (125 MHz, CDCl$_3$)**

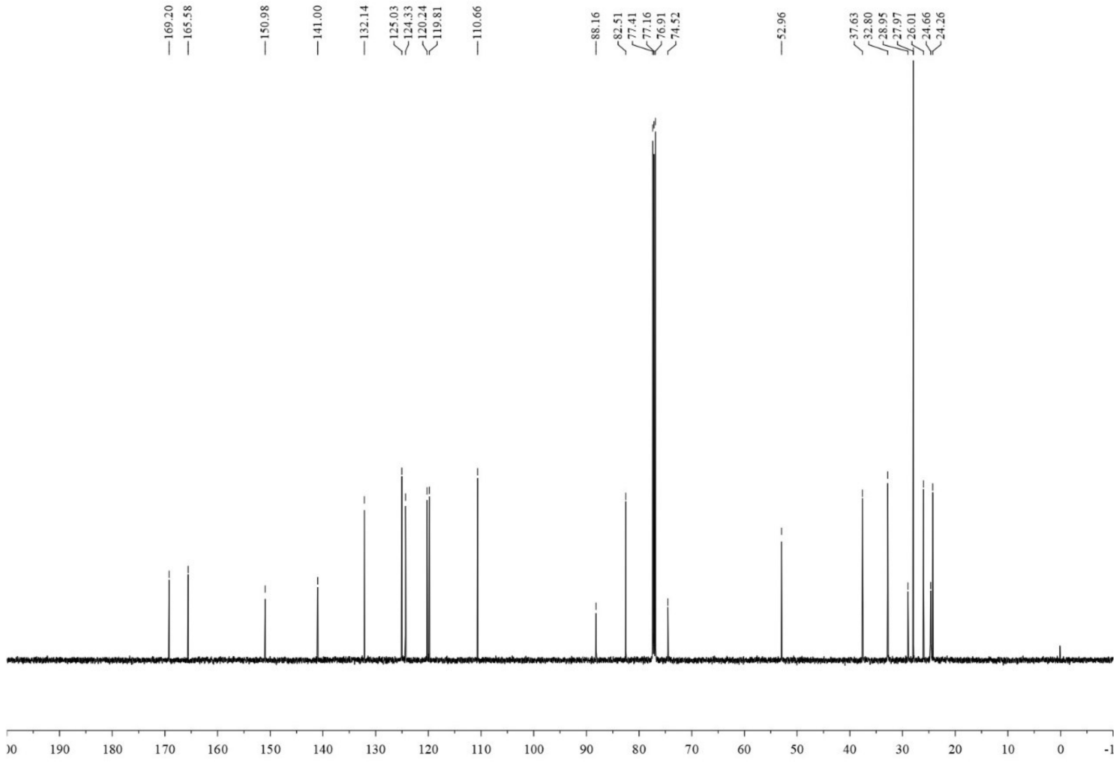

^{1}H NMR of **7aa (500 MHz, $CDCl_3$)**

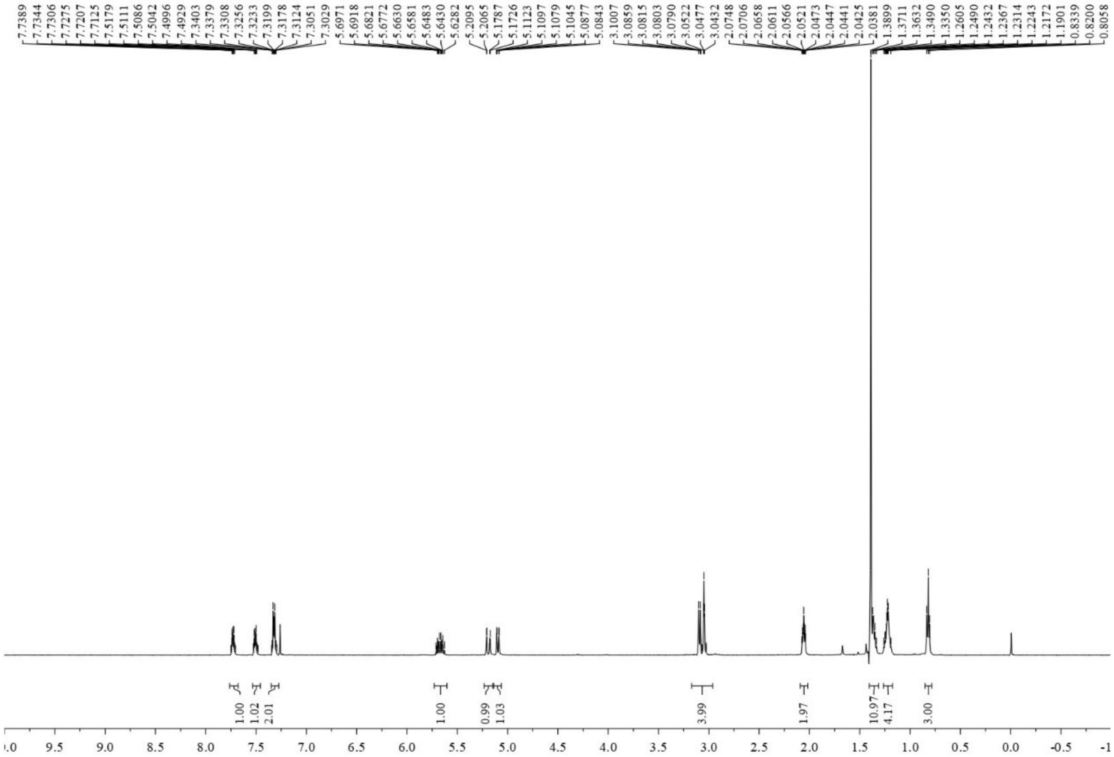

^{13}C NMR of **7aa (125 MHz, $CDCl_3$)**

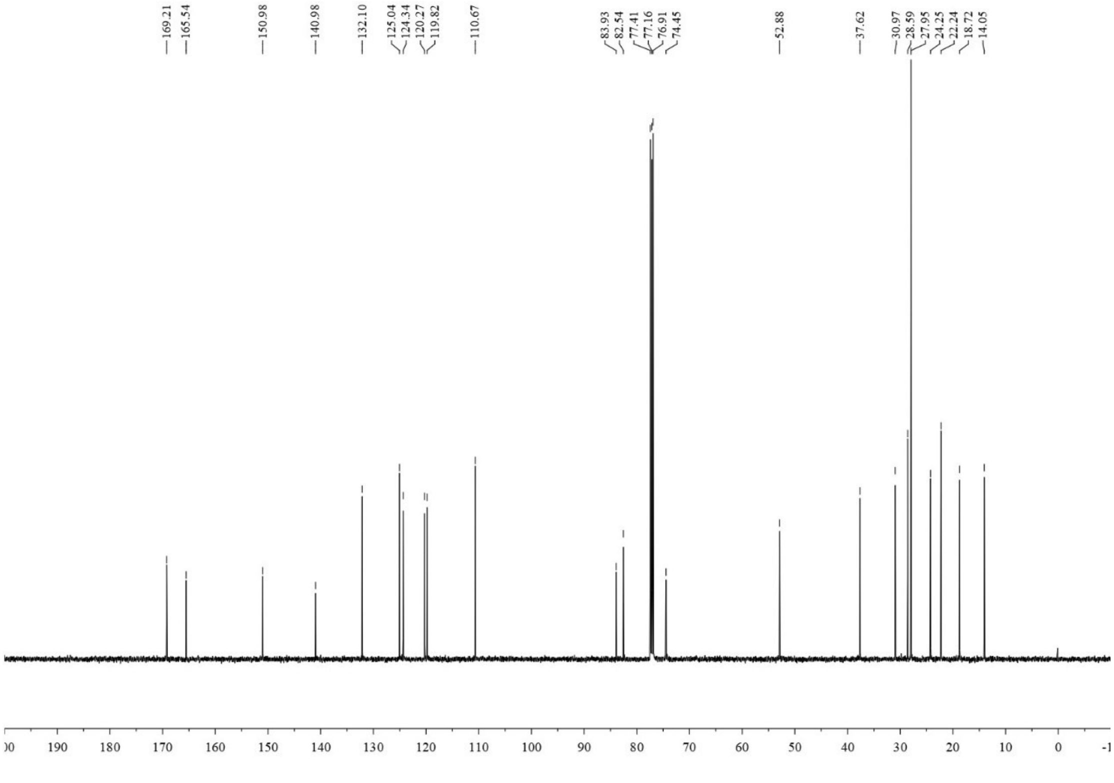

^{1}H NMR of **7ab (500 MHz, CDCl$_3$)**

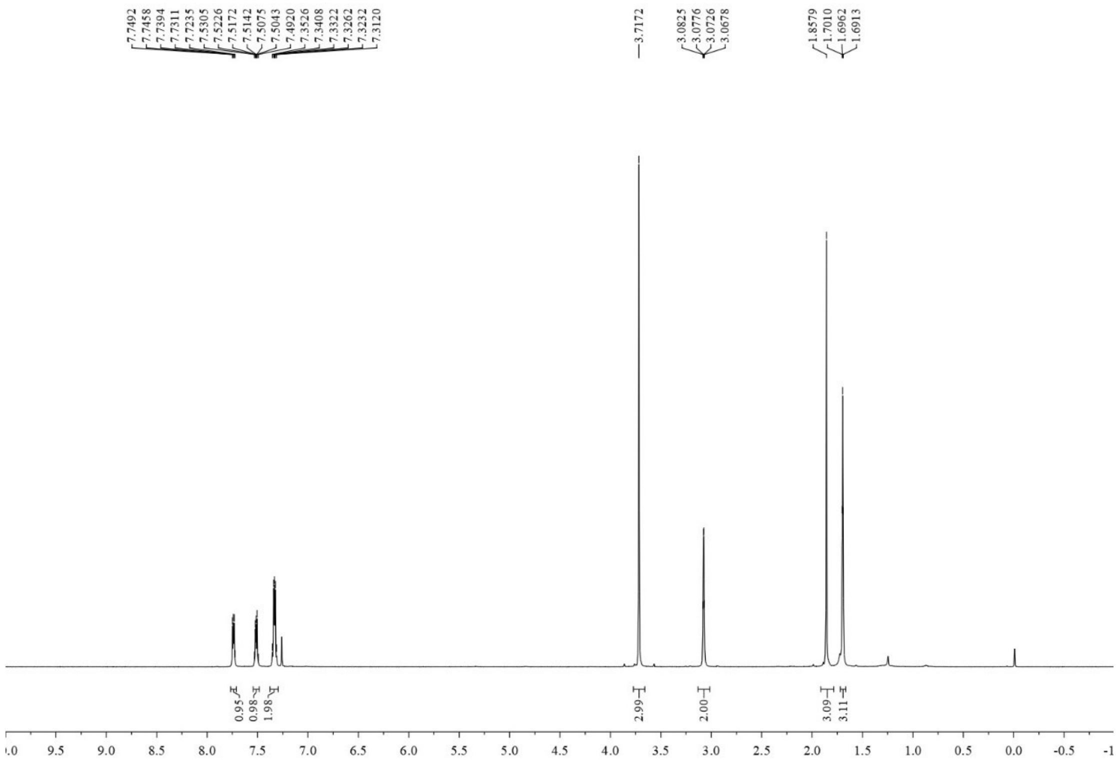

^{13}C NMR of **7ab (125 MHz, CDCl$_3$)**

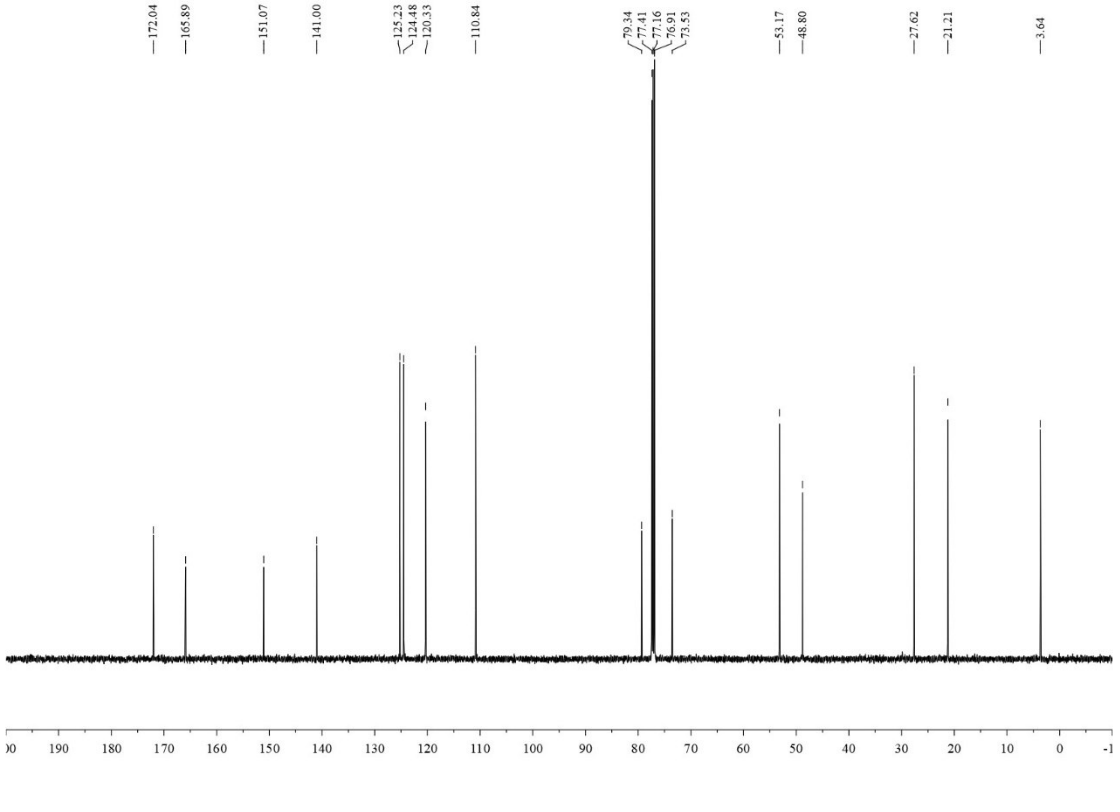

^{1}H NMR of **7ac (500 MHz, CDCl$_3$)**

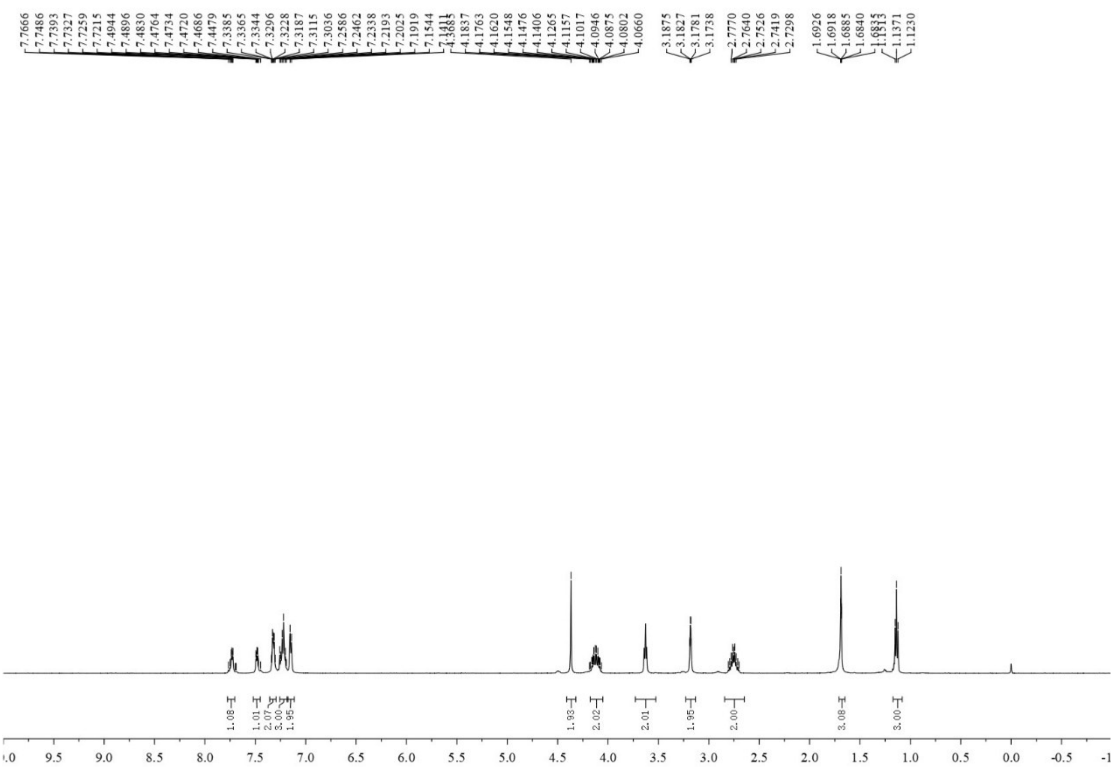

^{13}C NMR of **7ac (125 MHz, CDCl$_3$)**

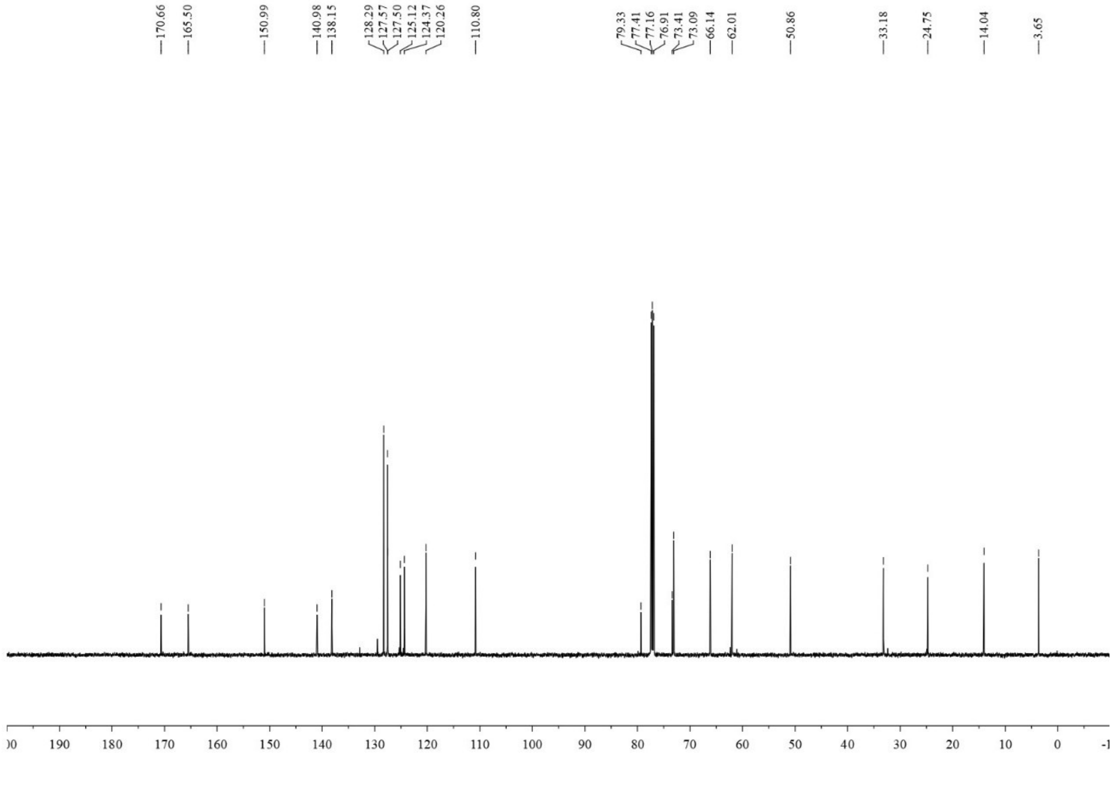

^{1}H NMR of **8 (500 MHz, CDCl$_3$)**

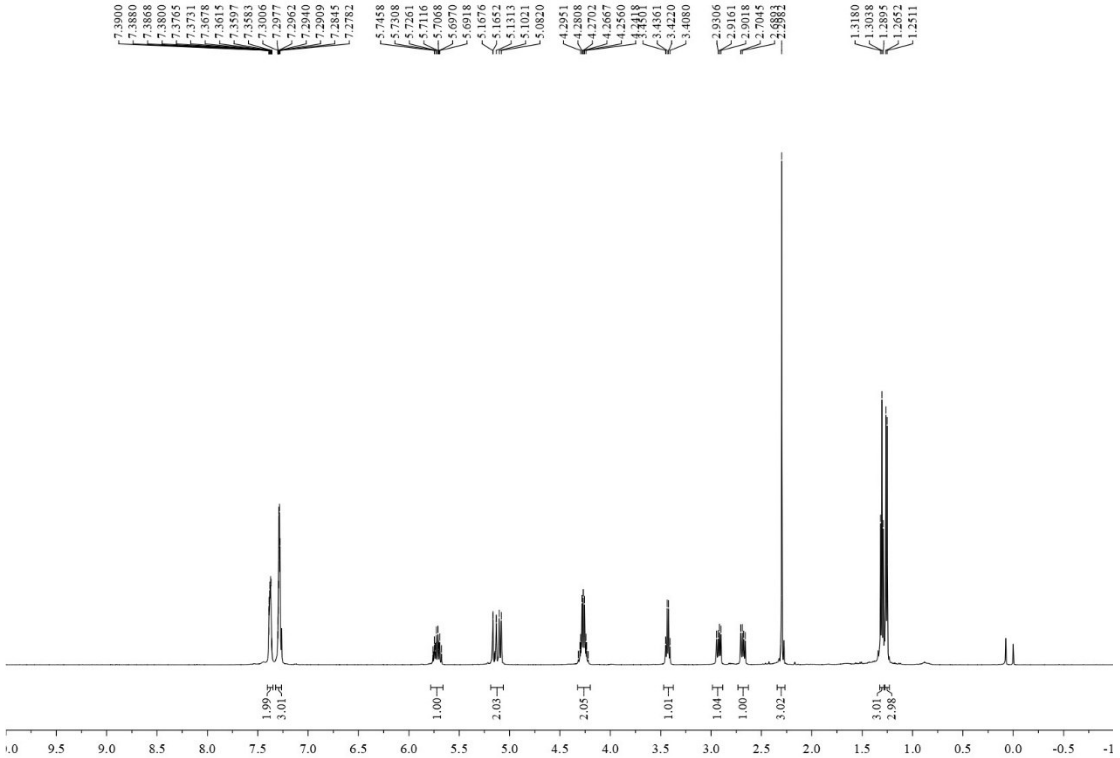

^{13}C NMR of **8 (125 MHz, CDCl$_3$)**

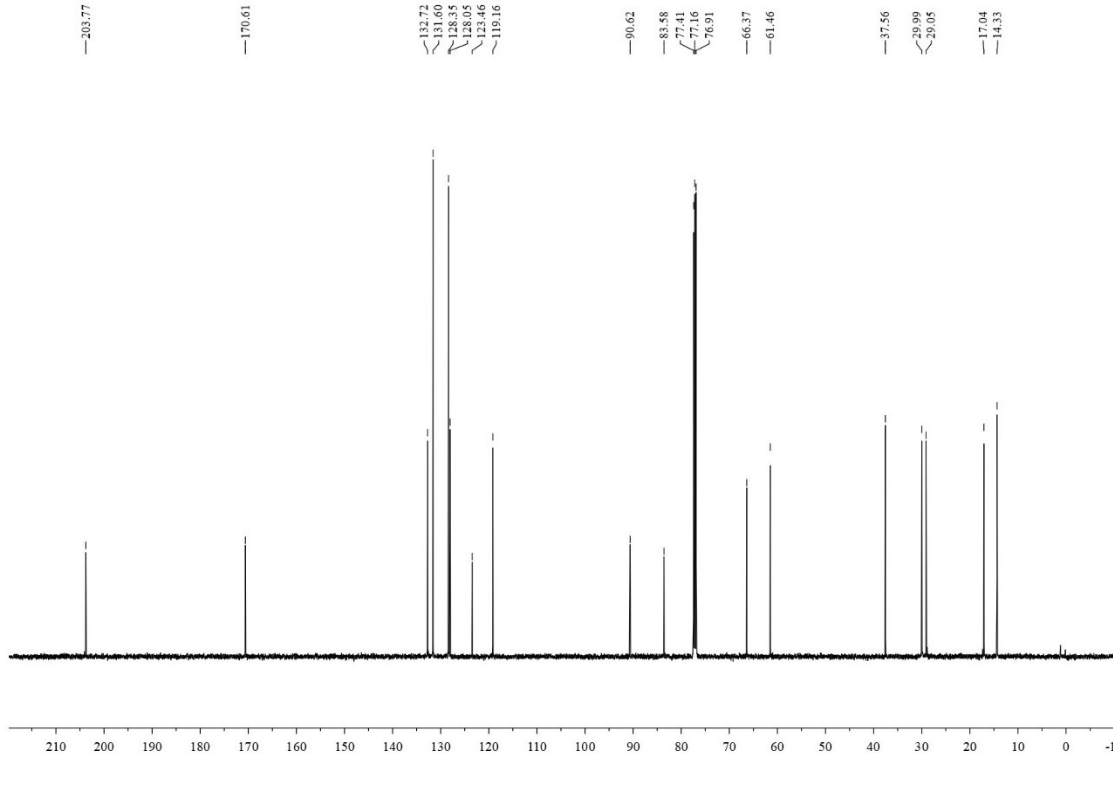

^{1}H NMR of **9 (400 MHz, CDCl$_3$)**

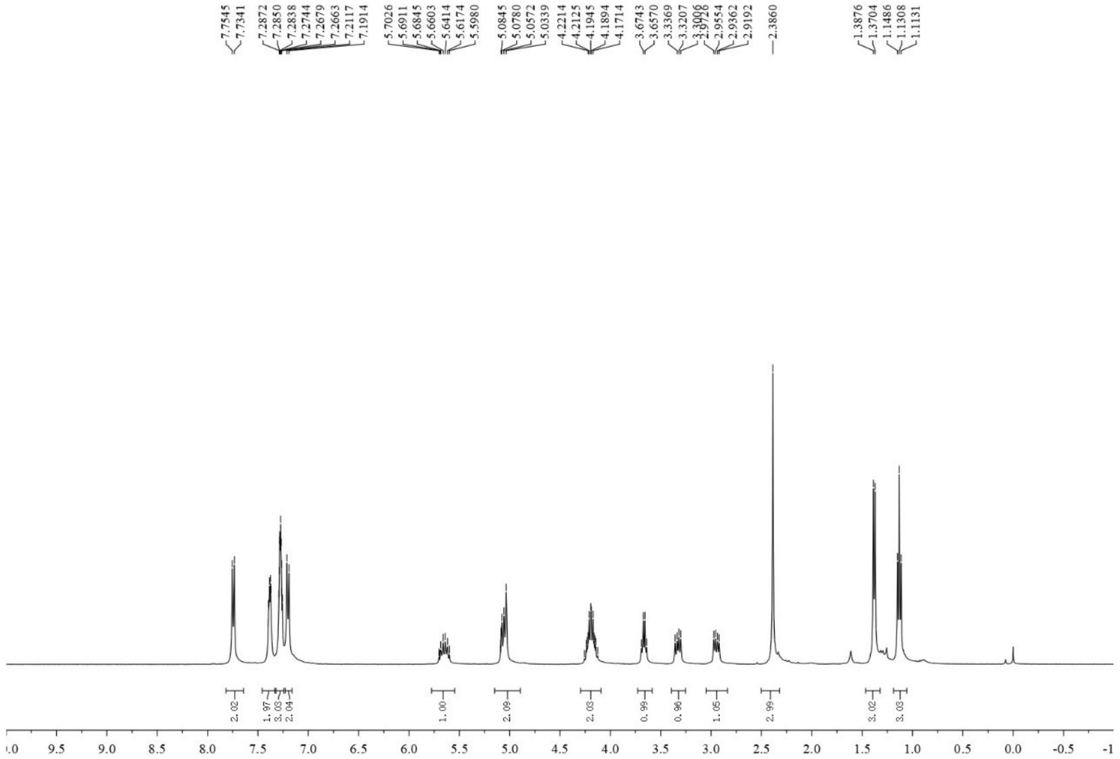

^{13}C NMR of **9 (100 MHz, CDCl$_3$)**

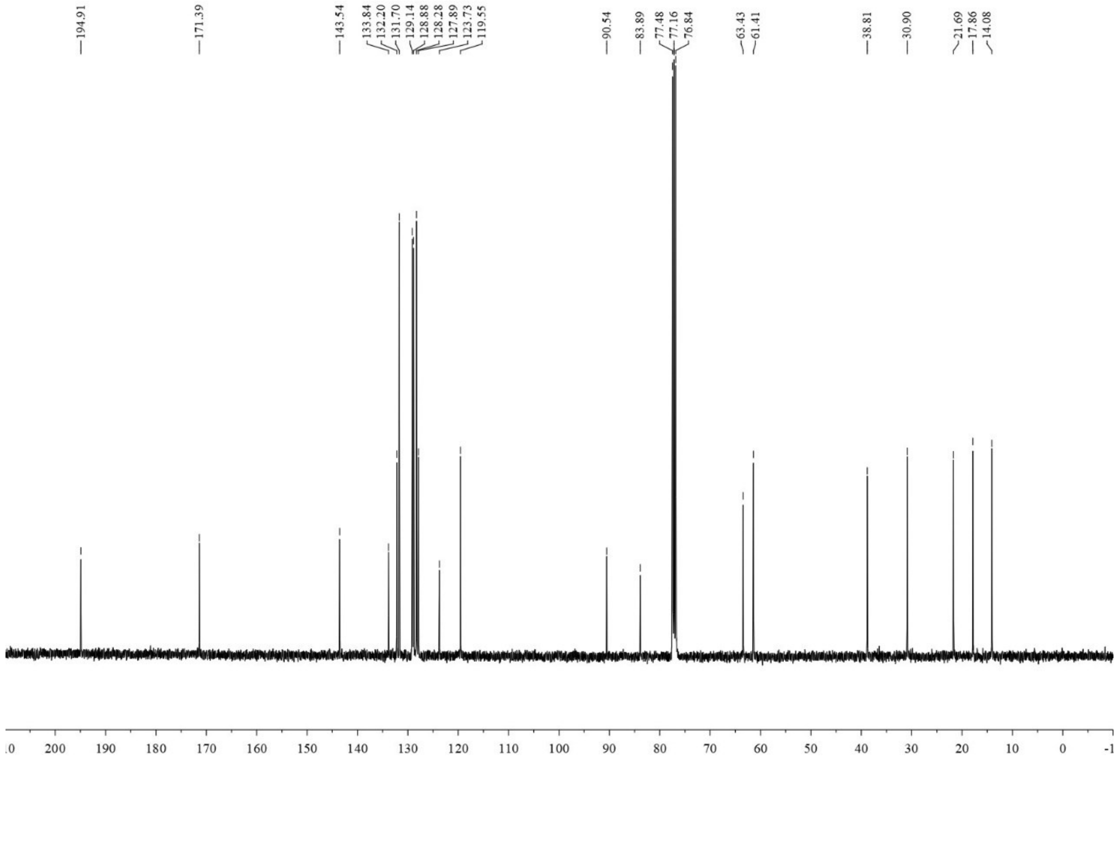

¹H NMR of **10 (500 MHz, CDCl$_3$)**

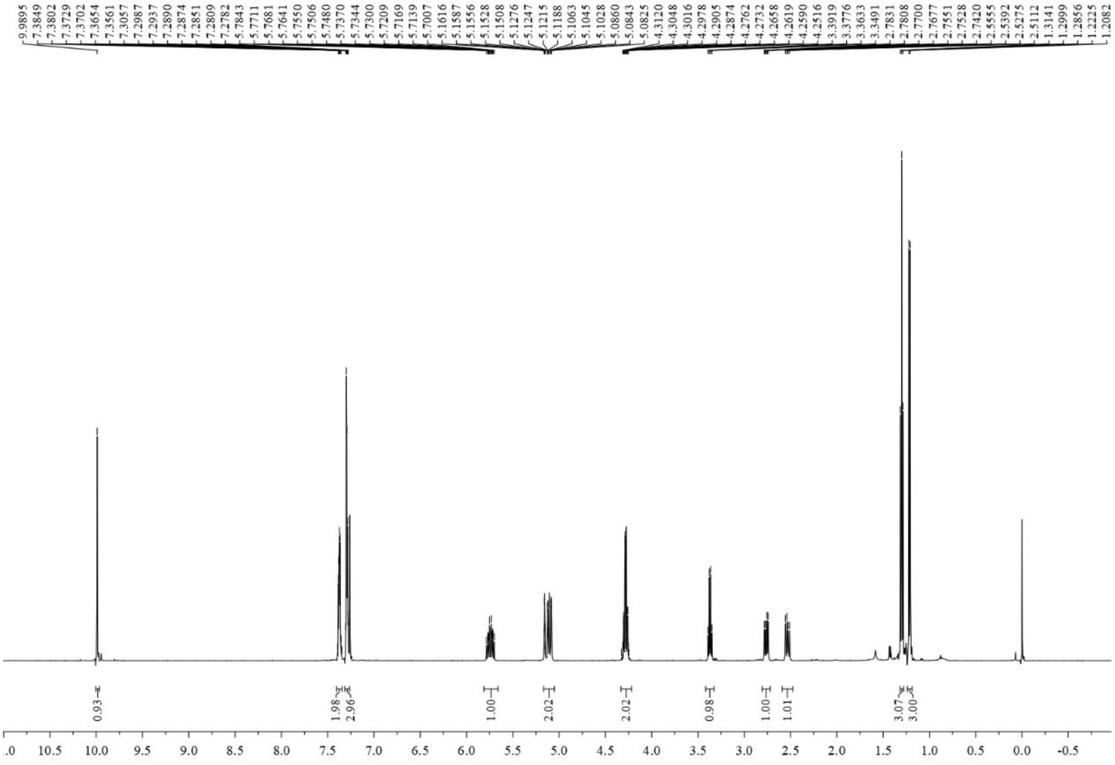

¹³C NMR of **10 (125 MHz, CDCl$_3$)**

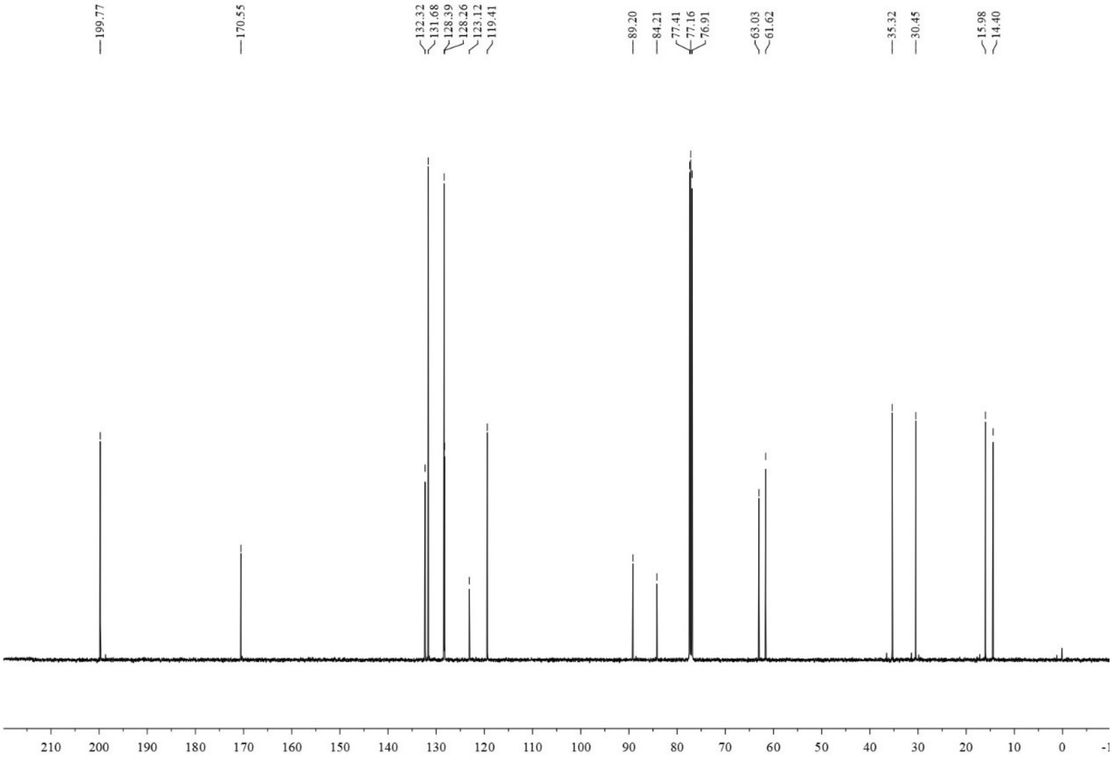

^{1}H NMR of **11 (500 MHz, CDCl$_3$)**

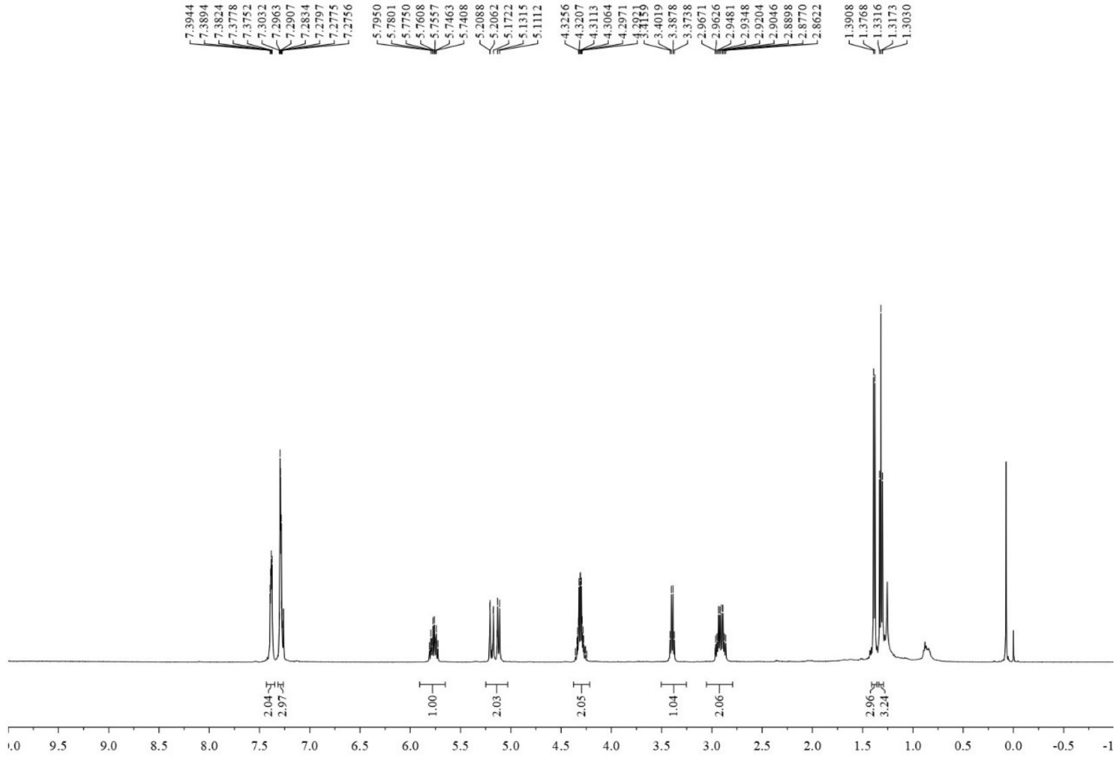

^{13}C NMR of **11 (125 MHz, CDCl$_3$)**

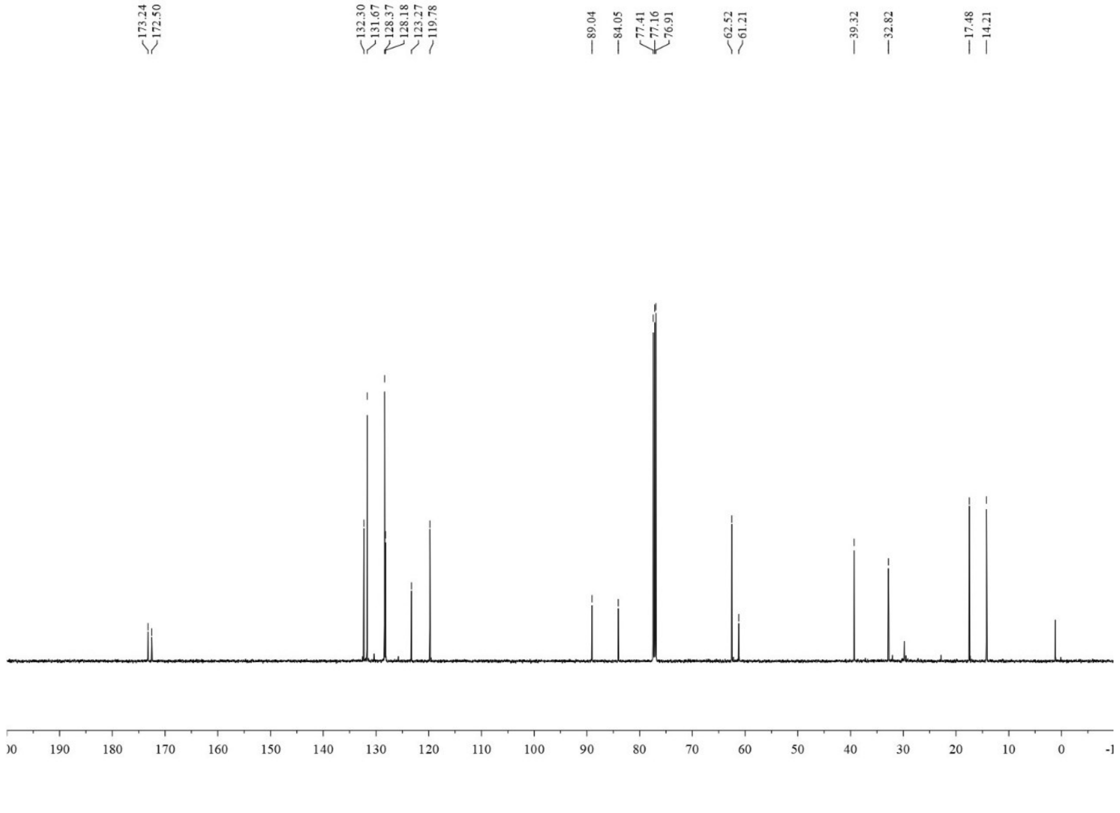

^{1}H NMR of **12 (500 MHz, $CDCl_3$)**

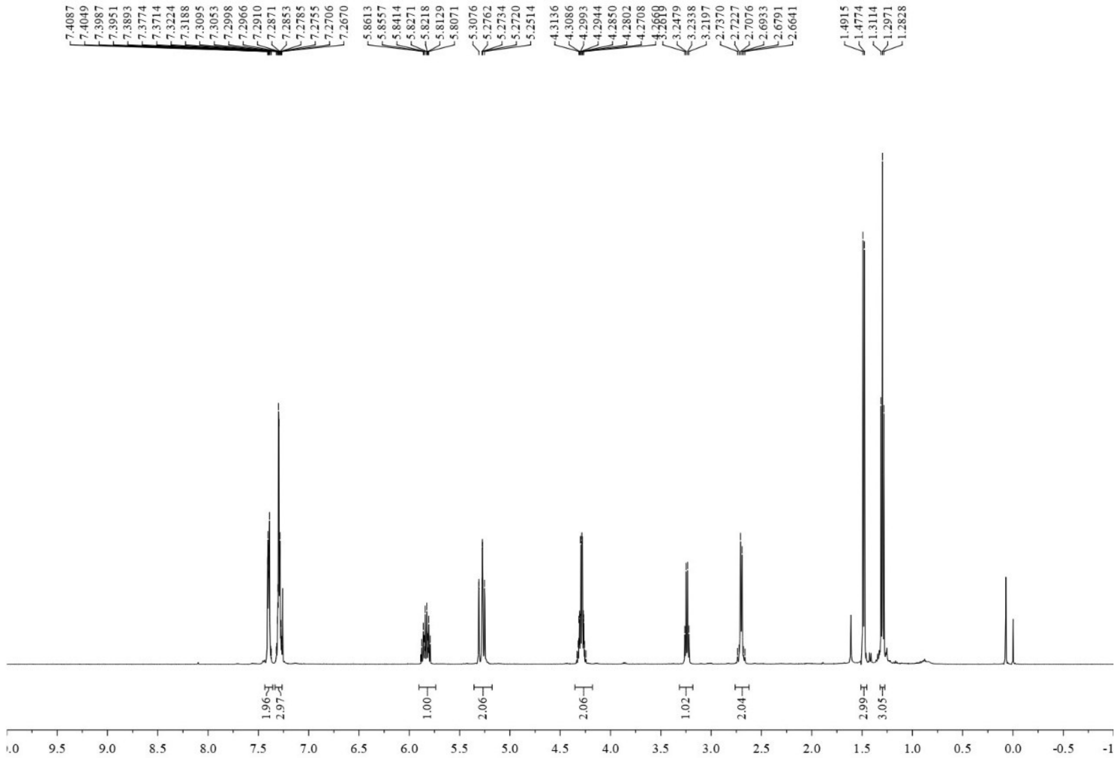

^{13}C NMR of **12 (125 MHz, $CDCl_3$)**

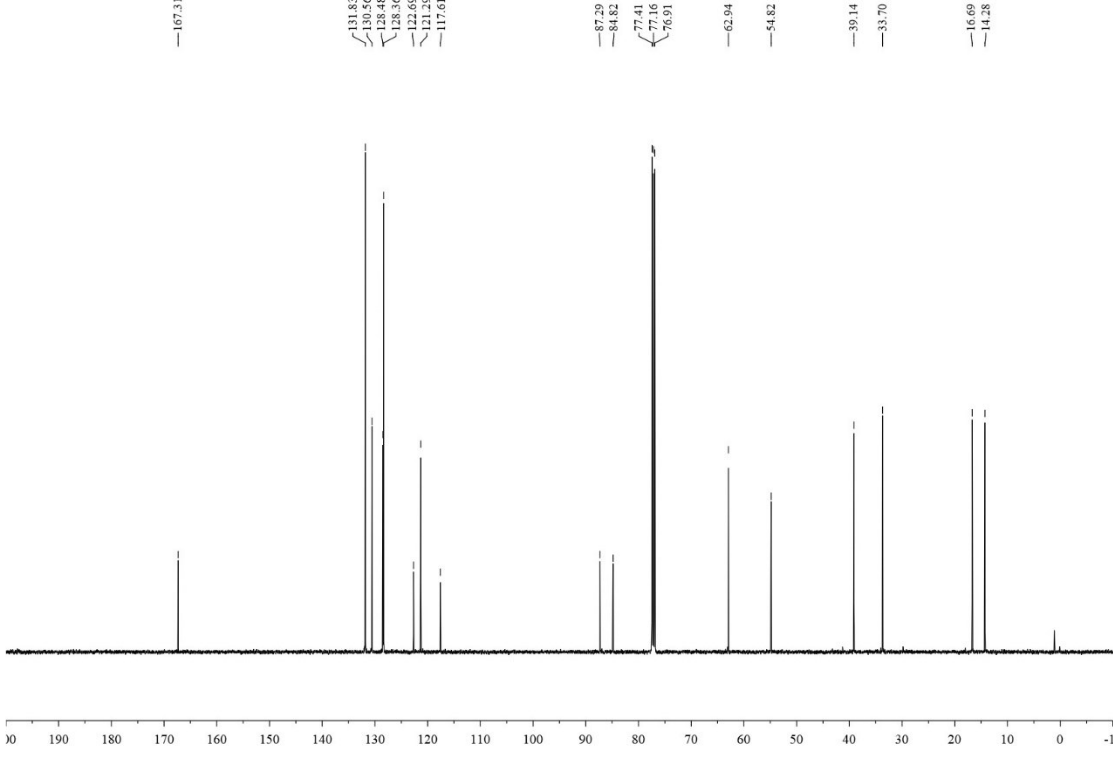

^{1}H NMR of **13 (500 MHz, CDCl$_3$)**

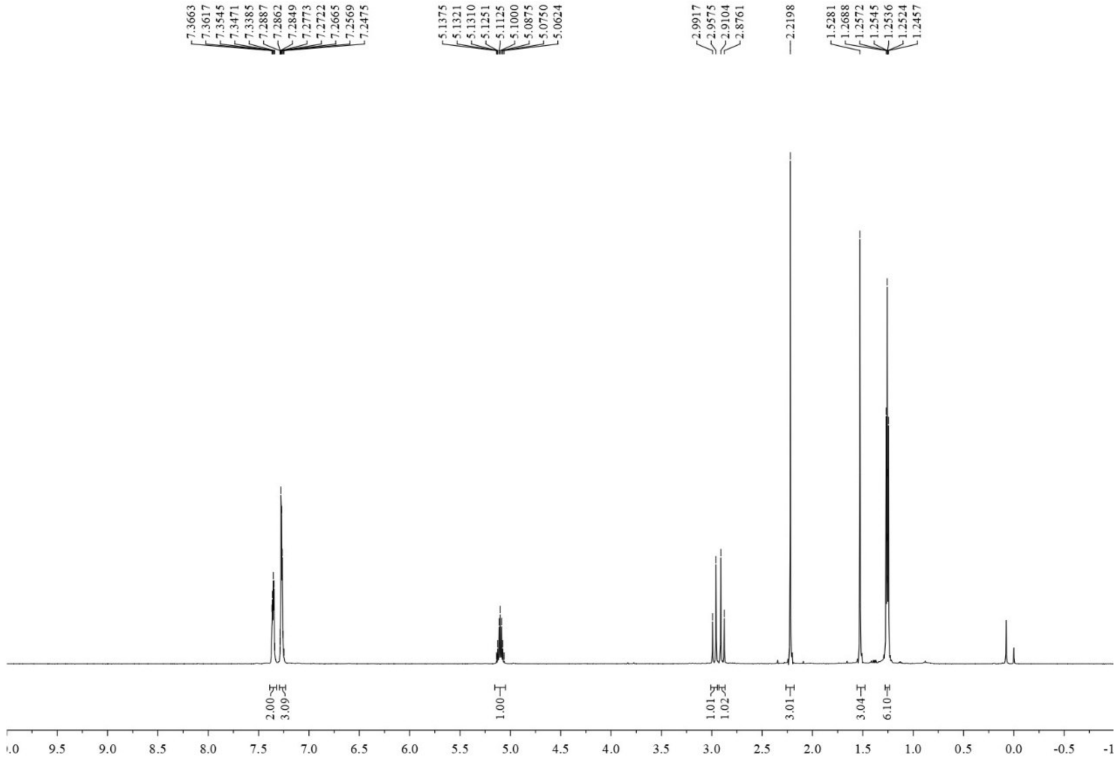

^{13}C NMR of **13 (125 MHz, CDCl$_3$)**

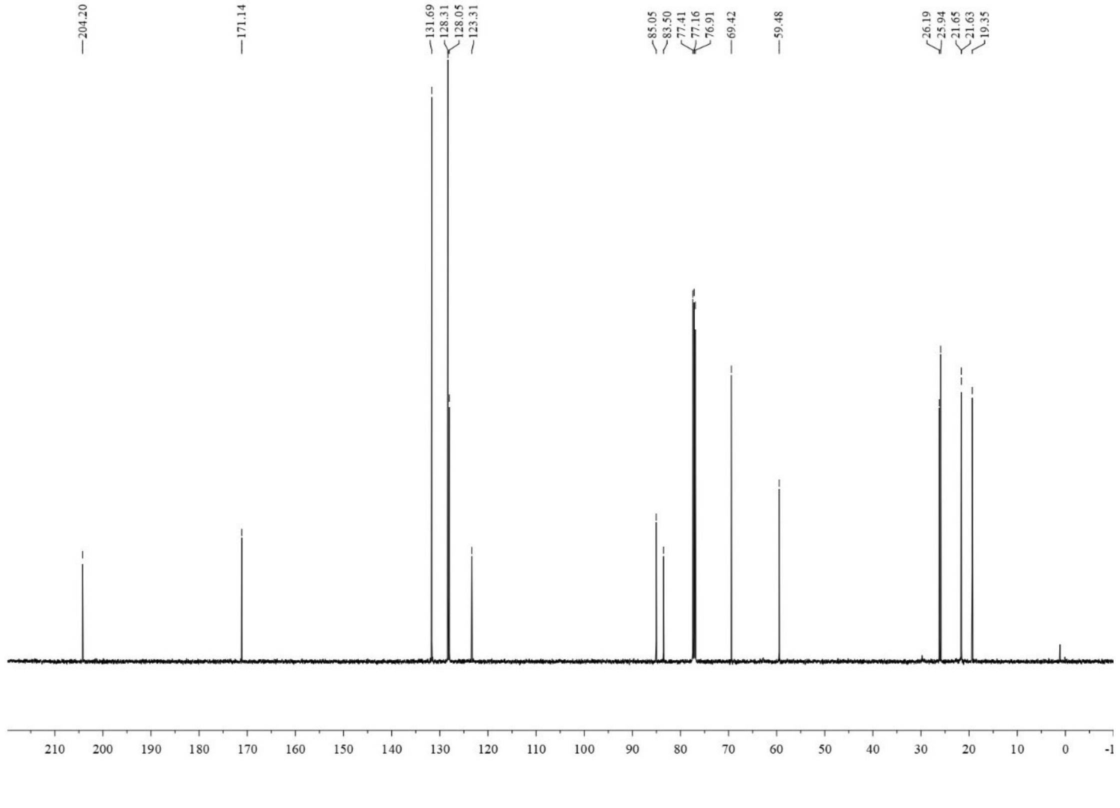

^{1}H NMR of **14 (500 MHz, CDCl$_3$)**

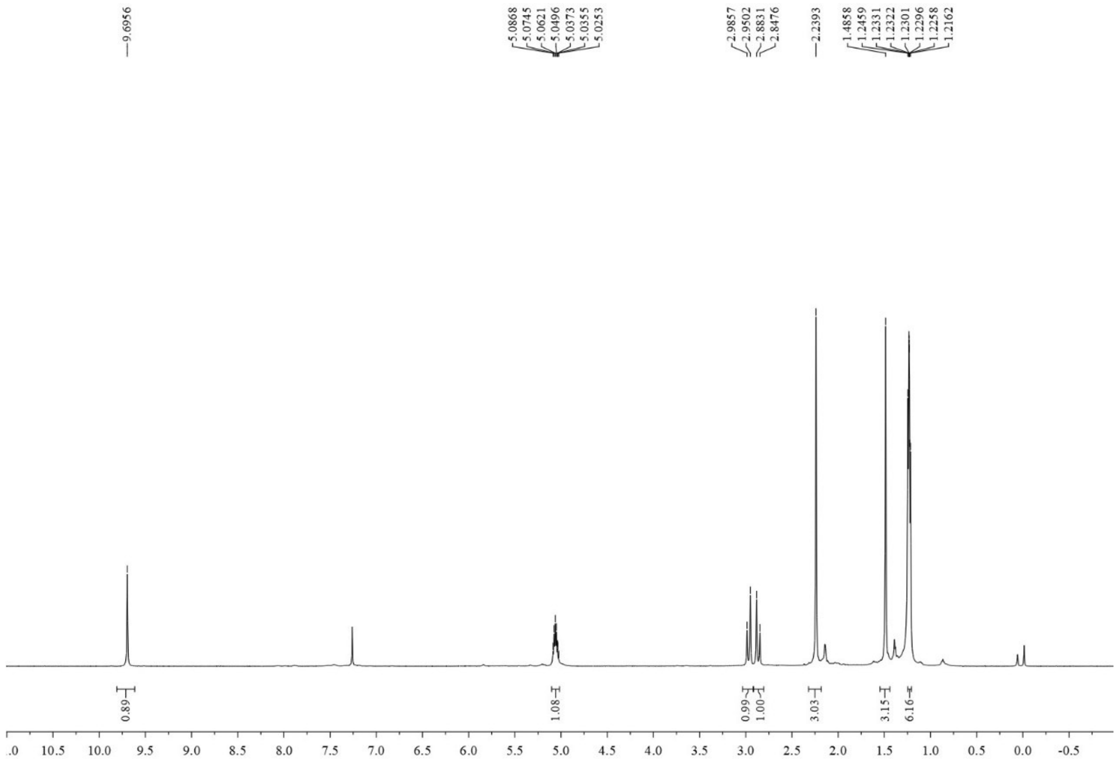

^{13}C NMR of **14 (125 MHz, CDCl$_3$)**

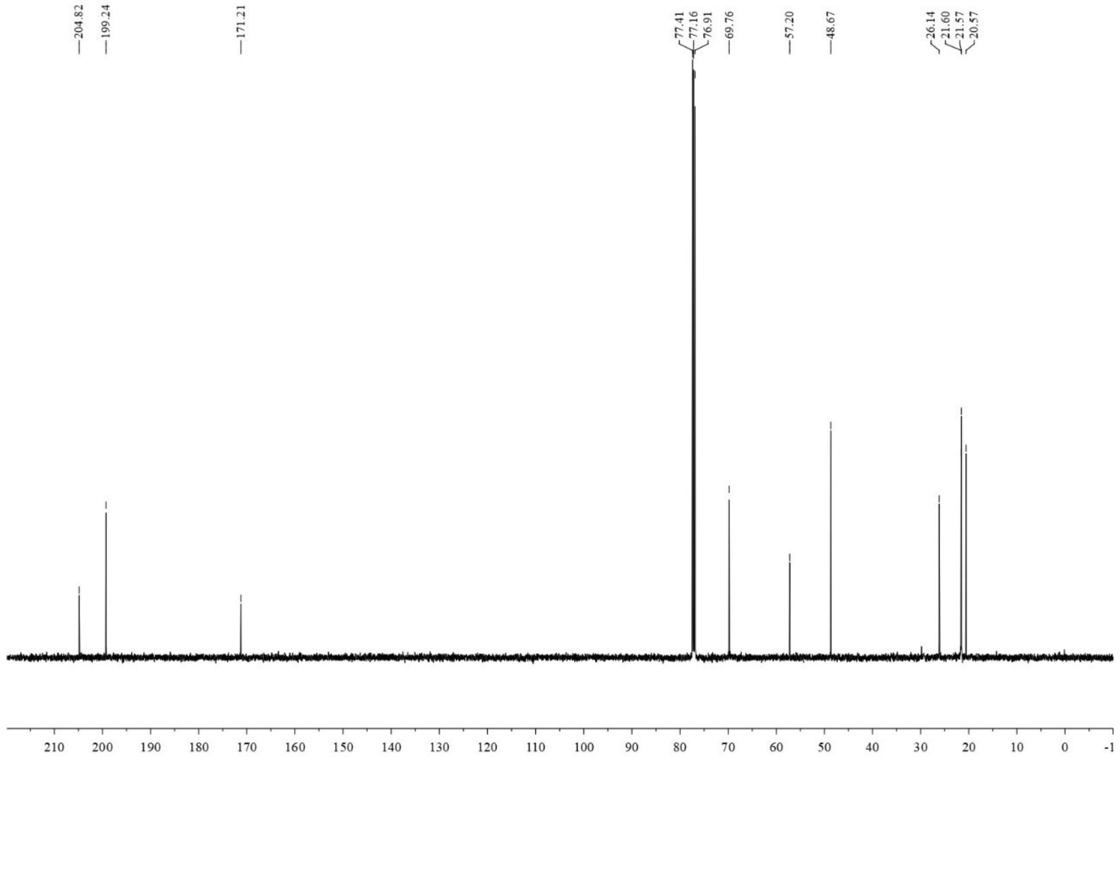

^{1}H NMR of **15 (500 MHz, CDCl$_3$)**

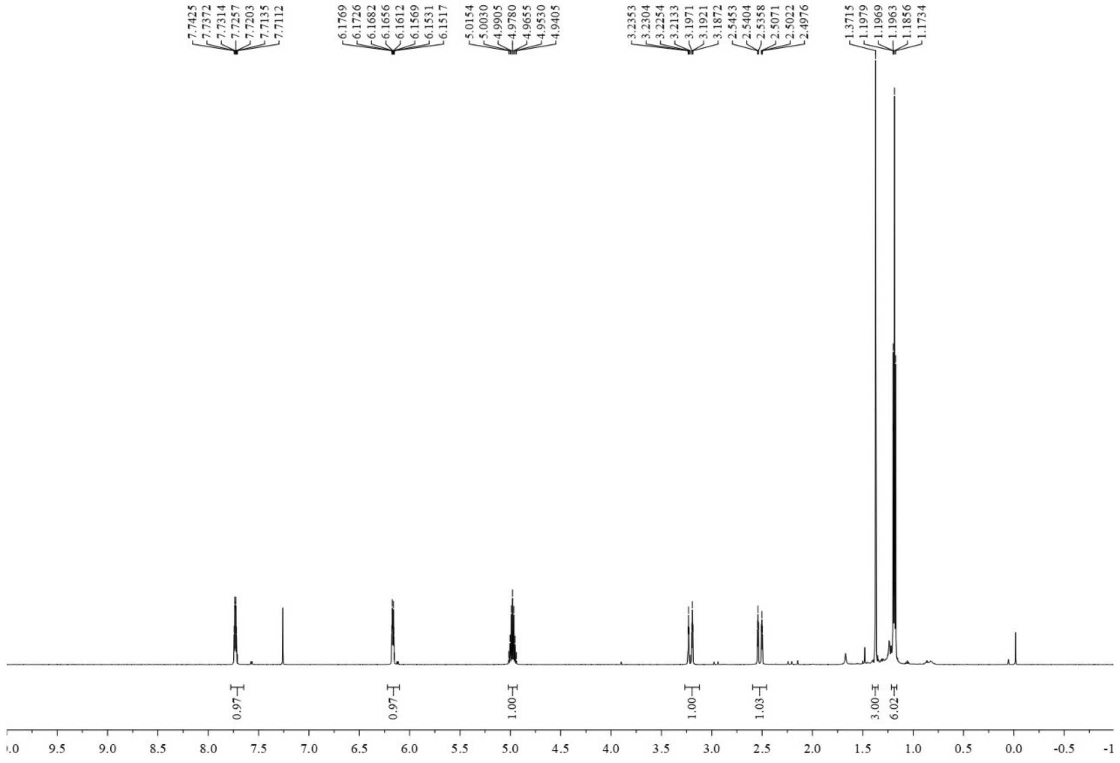

^{13}C NMR of **15 (125 MHz, CDCl$_3$)**

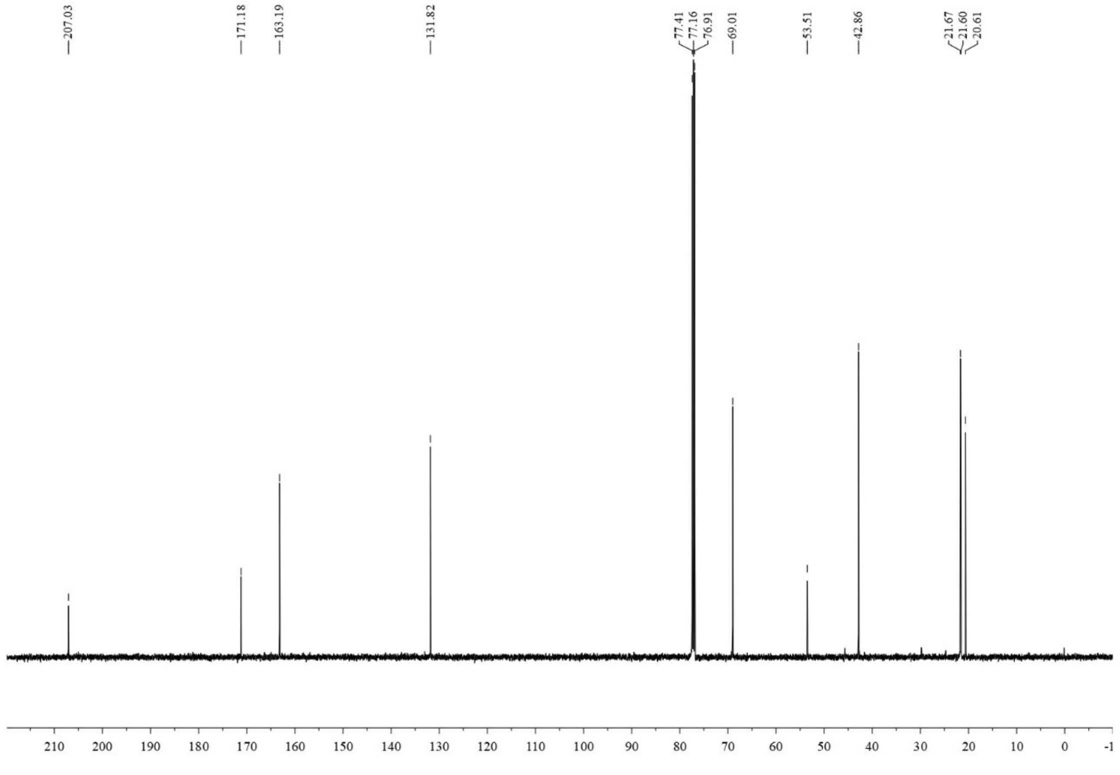

^{1}H **NMR** of **16 (500 MHz, CDCl$_3$)**

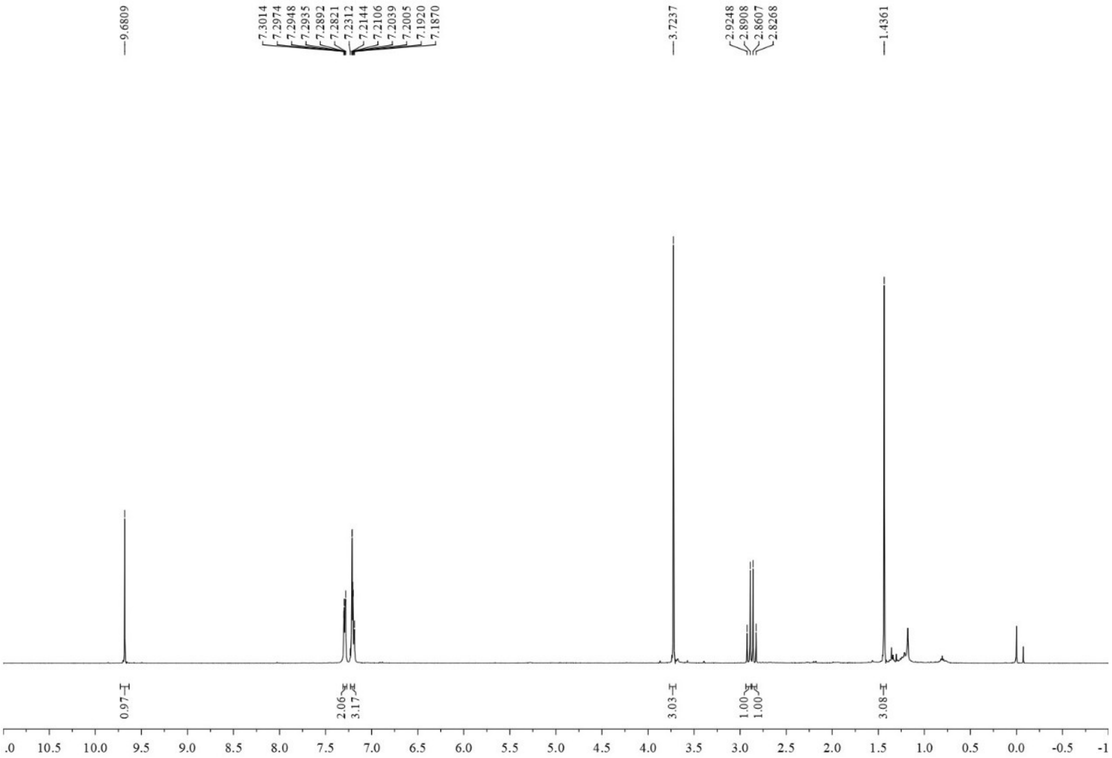

^{13}C **NMR** of **16 (125 MHz, CDCl$_3$)**

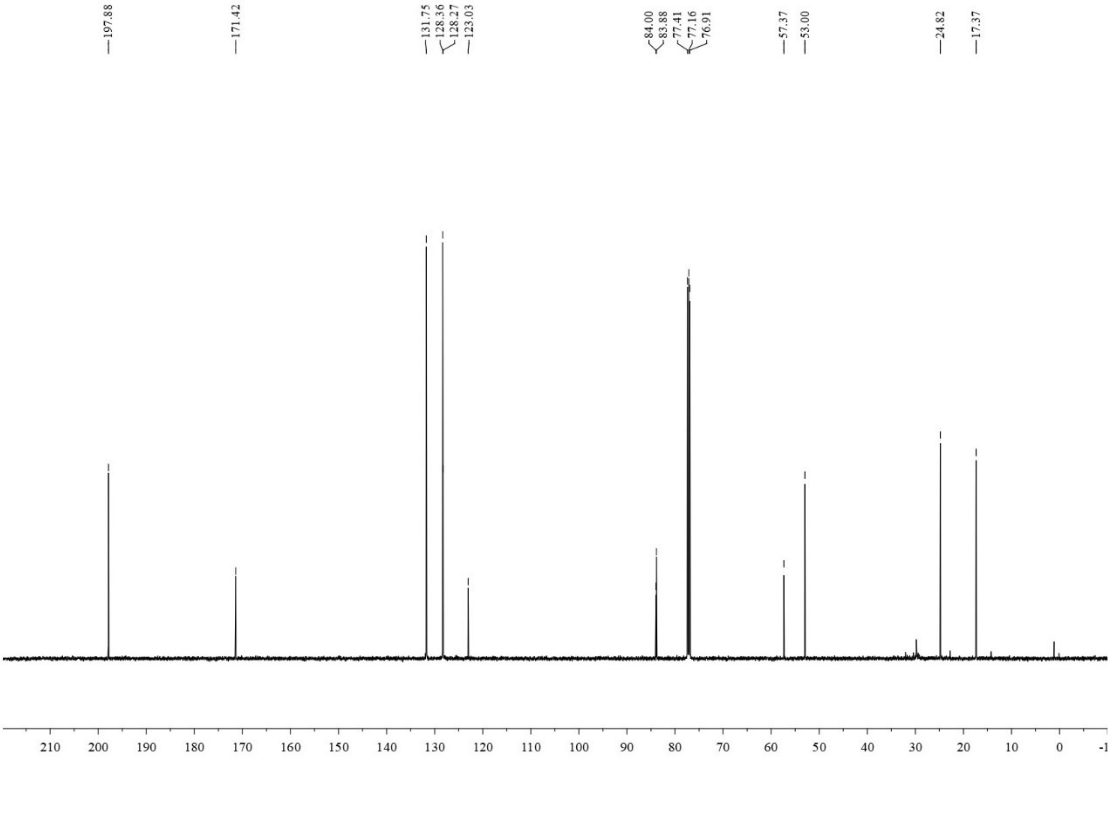

^{1}H NMR of **17 (500 MHz, $CDCl_3$)**

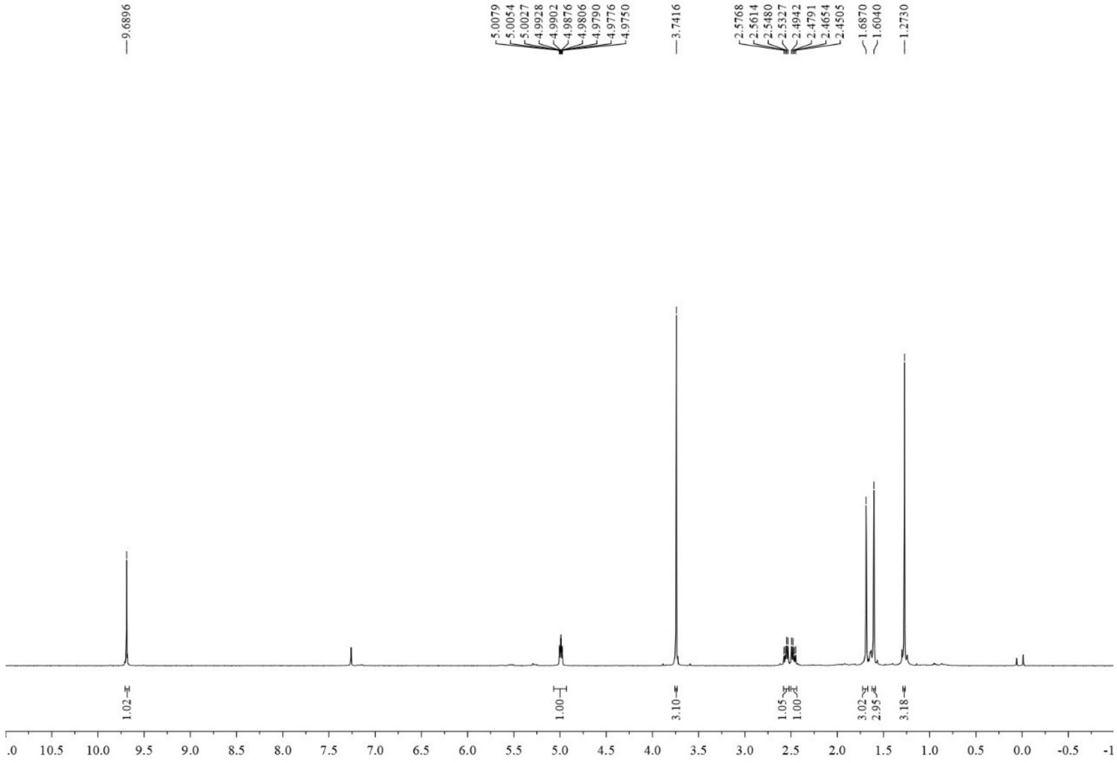

^{13}C NMR of **17 (125 MHz, $CDCl_3$)**

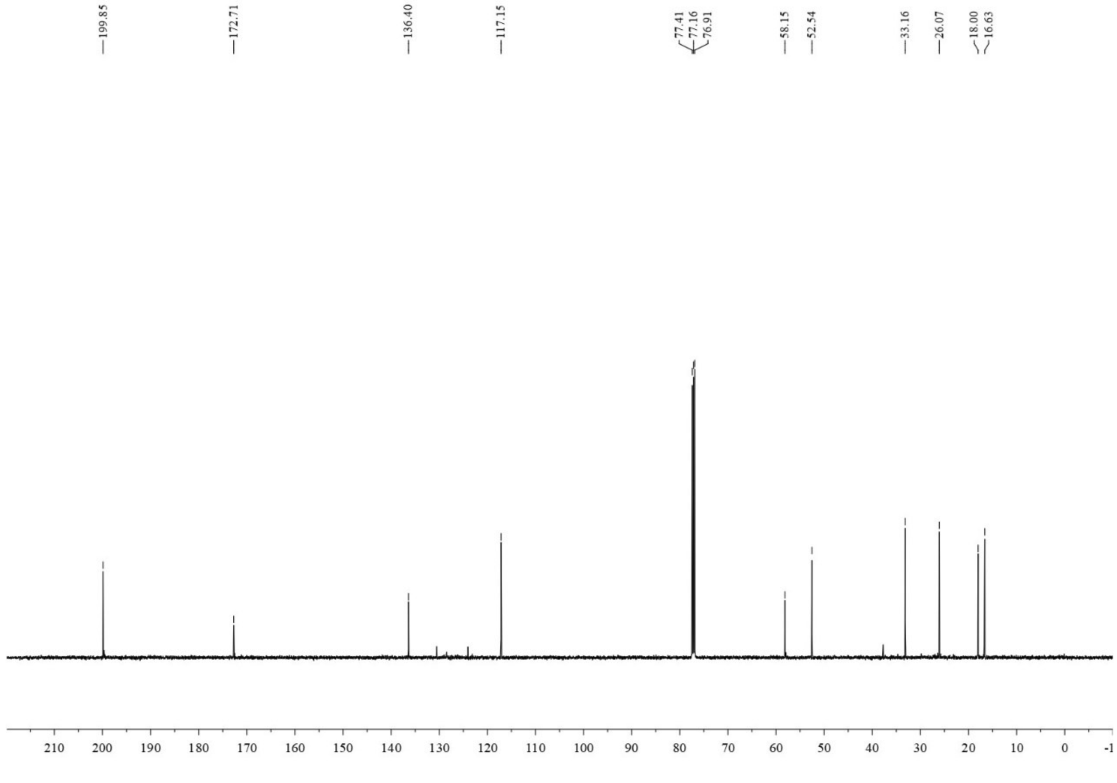

^{1}H NMR of (*R,S*)**-18 (500 MHz, CDCl$_3$)**

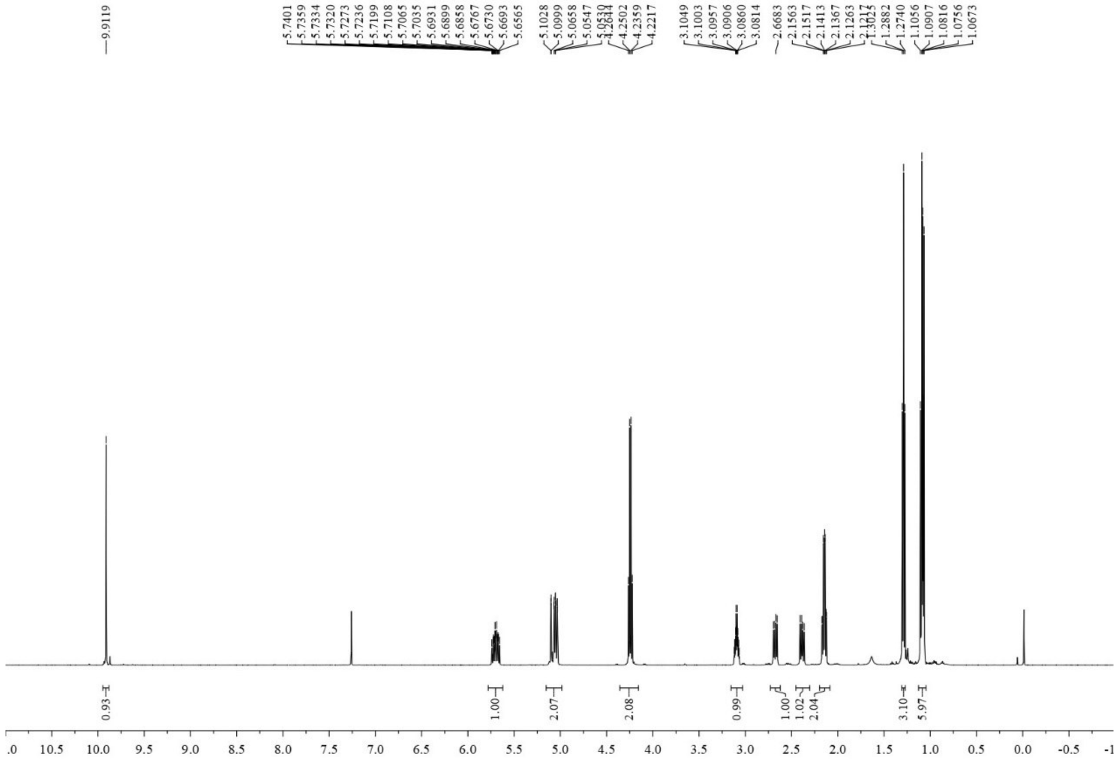

^{13}C NMR of (*R,S*)**-18 (125 MHz, CDCl$_3$)**

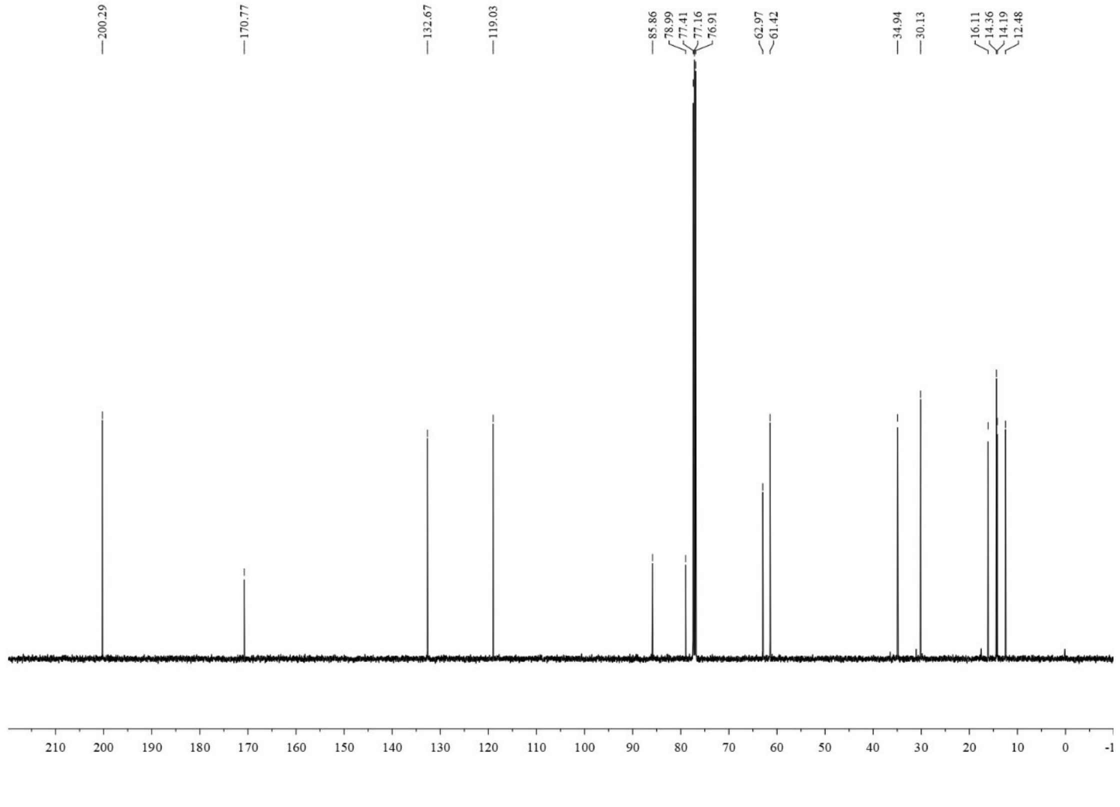

^{1}H NMR of (*S,R*)**-18 (500 MHz, $CDCl_3$)**

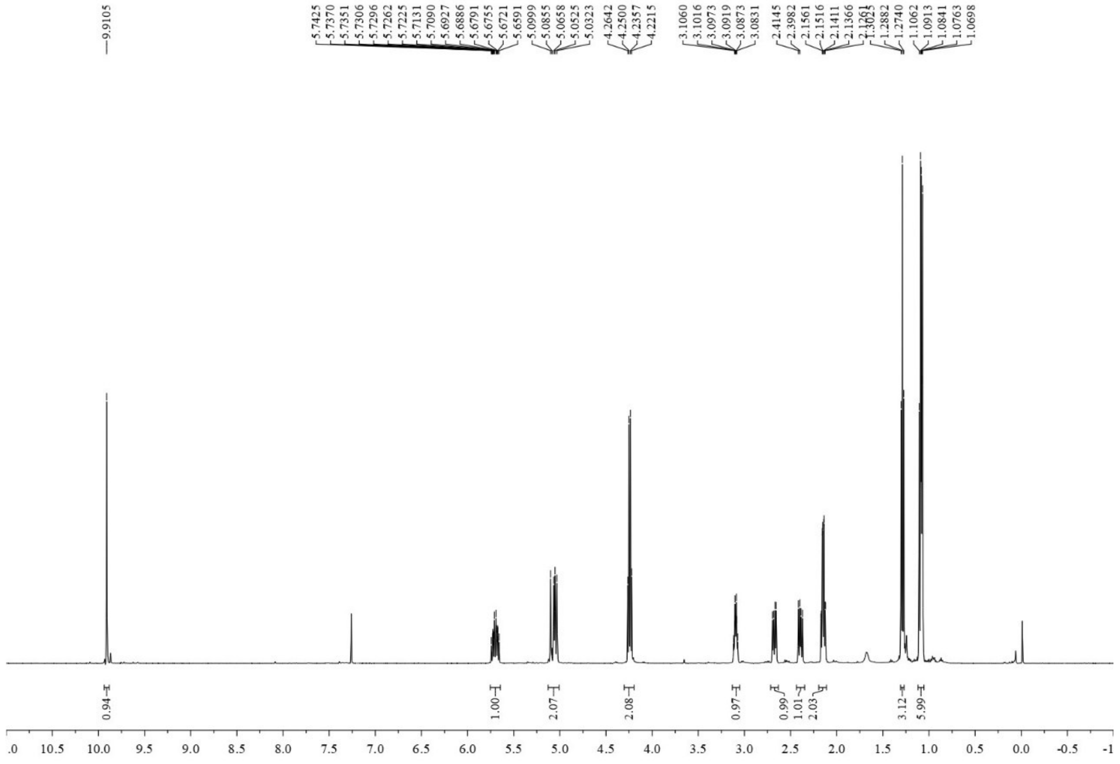

^{13}C NMR of (*S,R*)**-18 (125 MHz, $CDCl_3$)**

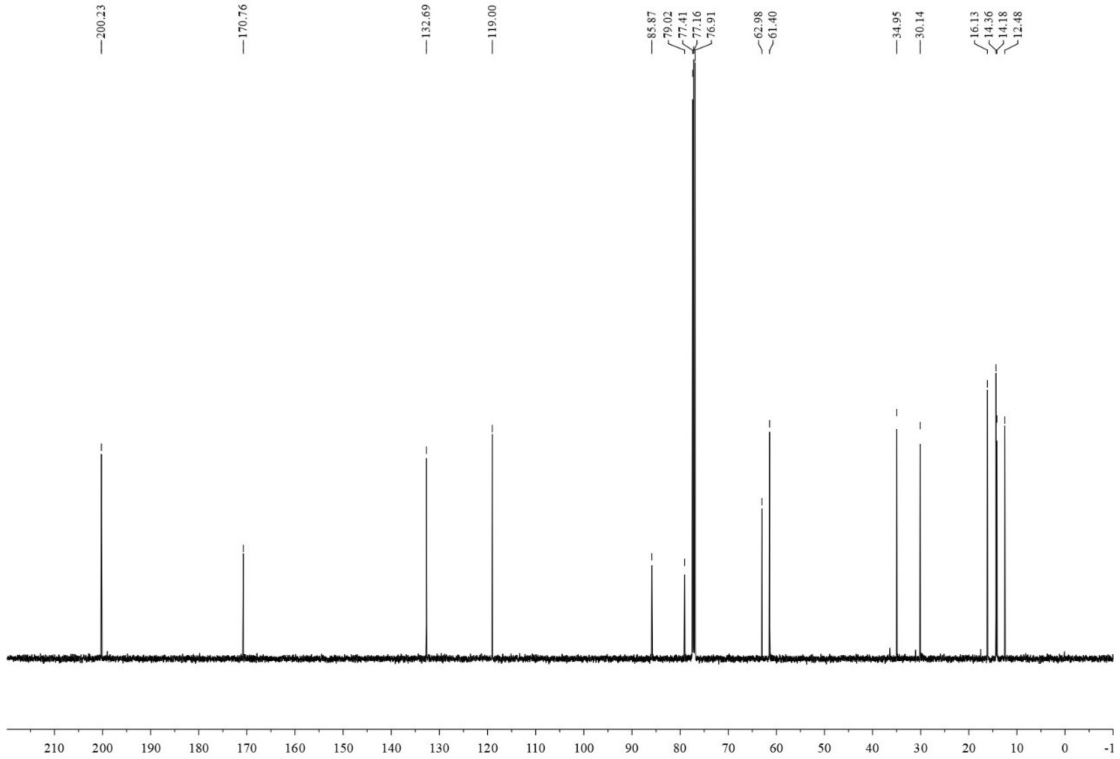

¹H NMR of (*R,R*)**-18 (500 MHz, CDCl$_3$)**

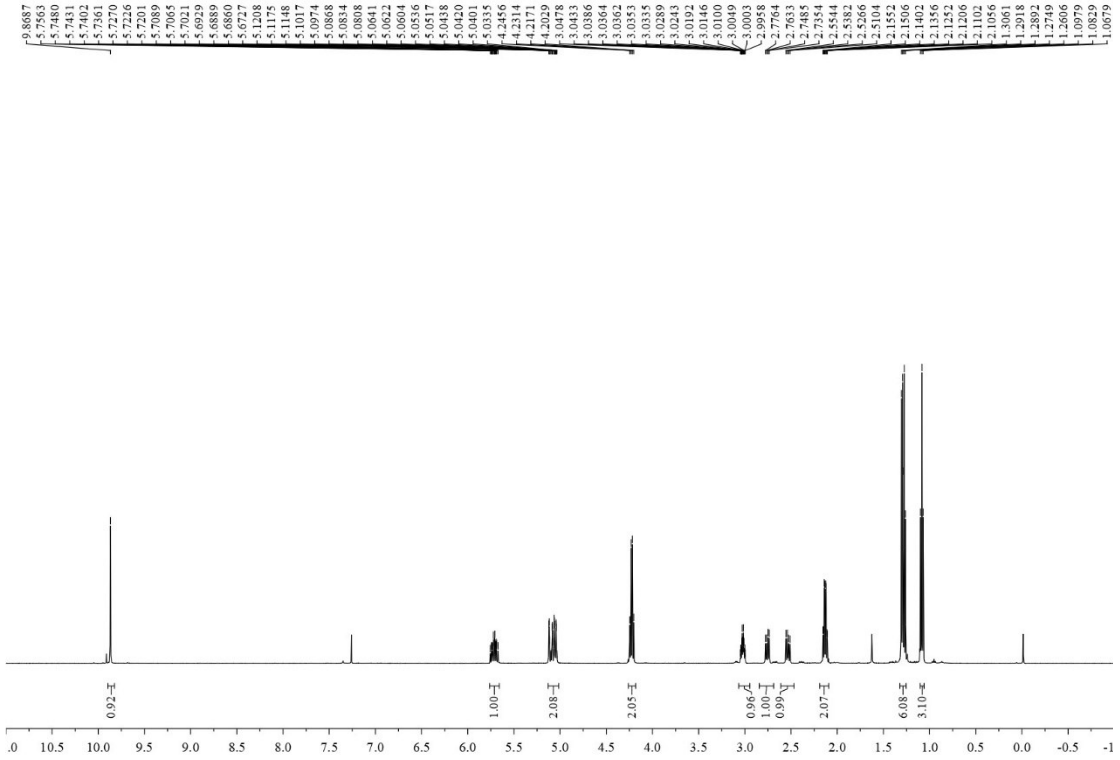

¹³C NMR of (*R,R*)**-18 (125 MHz, CDCl$_3$)**

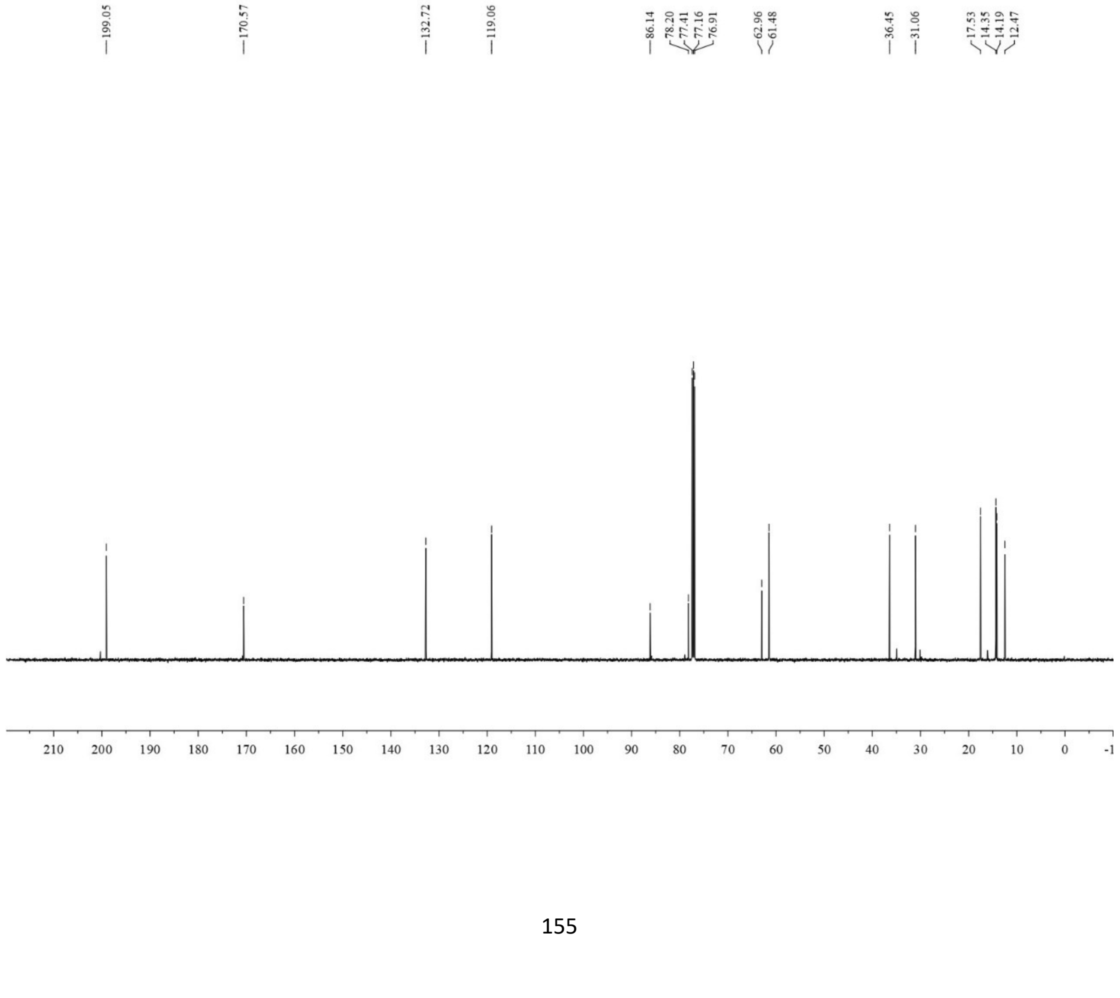

^{1}H NMR of (*S,S*)-**18 (500 MHz, CDCl$_3$)**

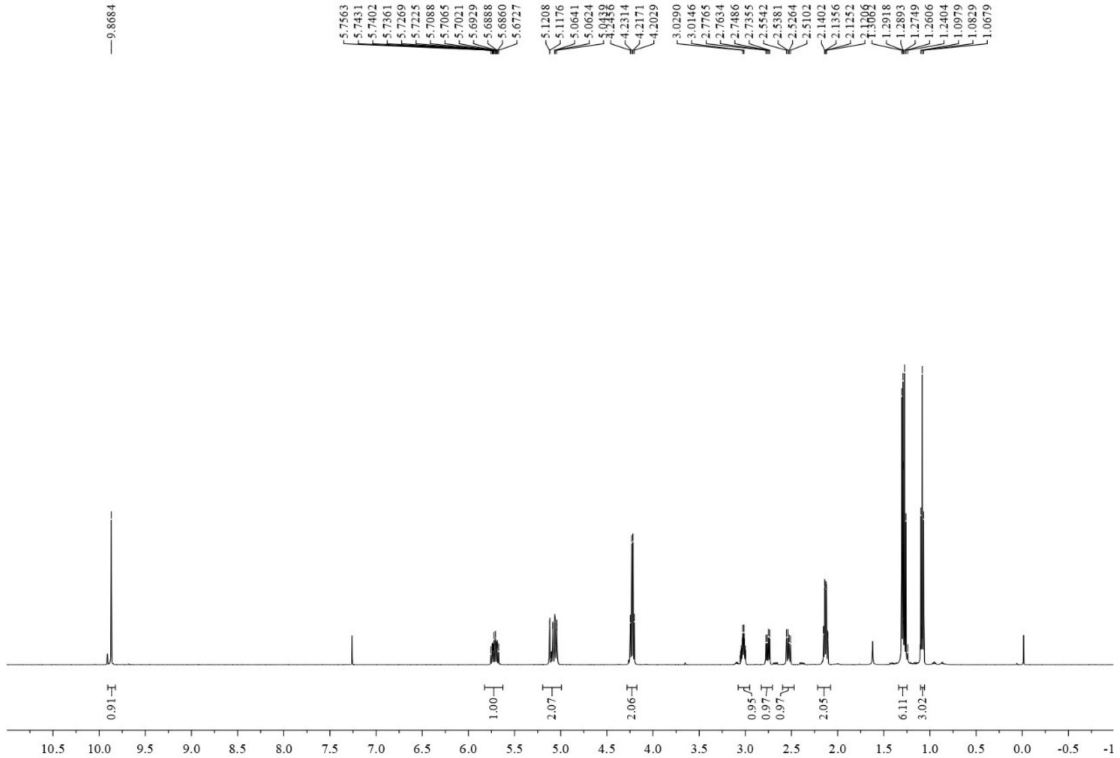

^{13}C NMR of (*S,S*)-**18 (125 MHz, CDCl$_3$)**

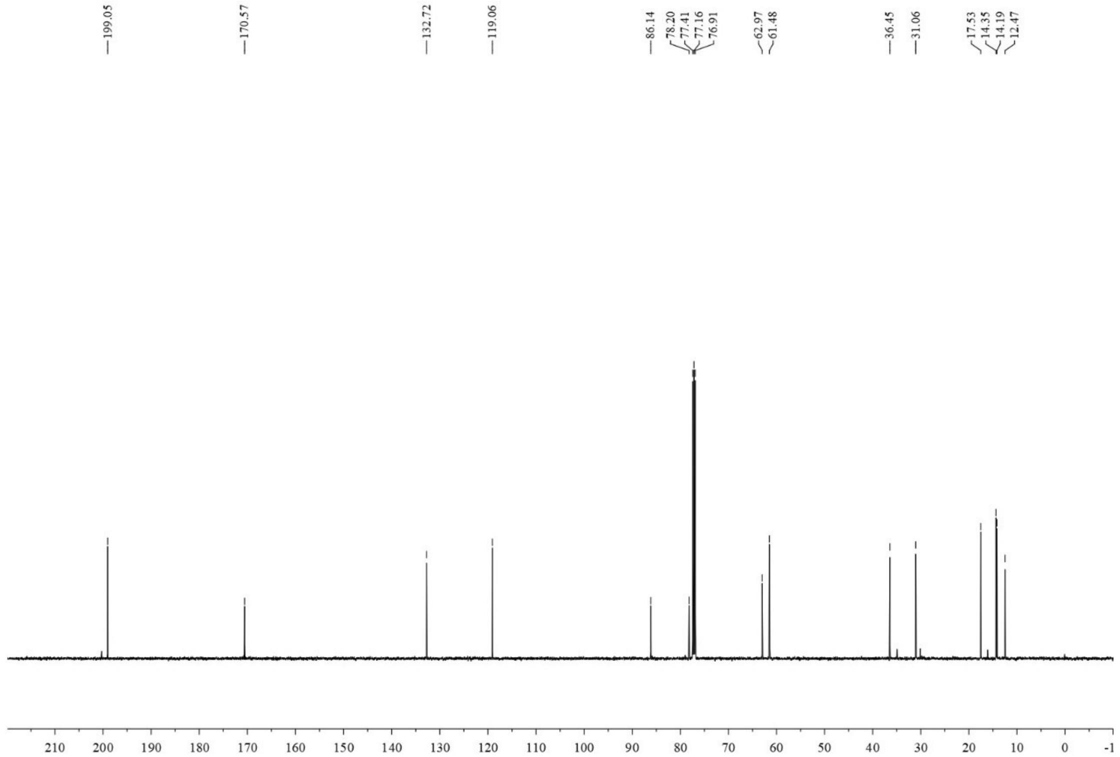

^{1}H NMR of (*R,S*)**-19 (500 MHz, CDCl$_3$)**

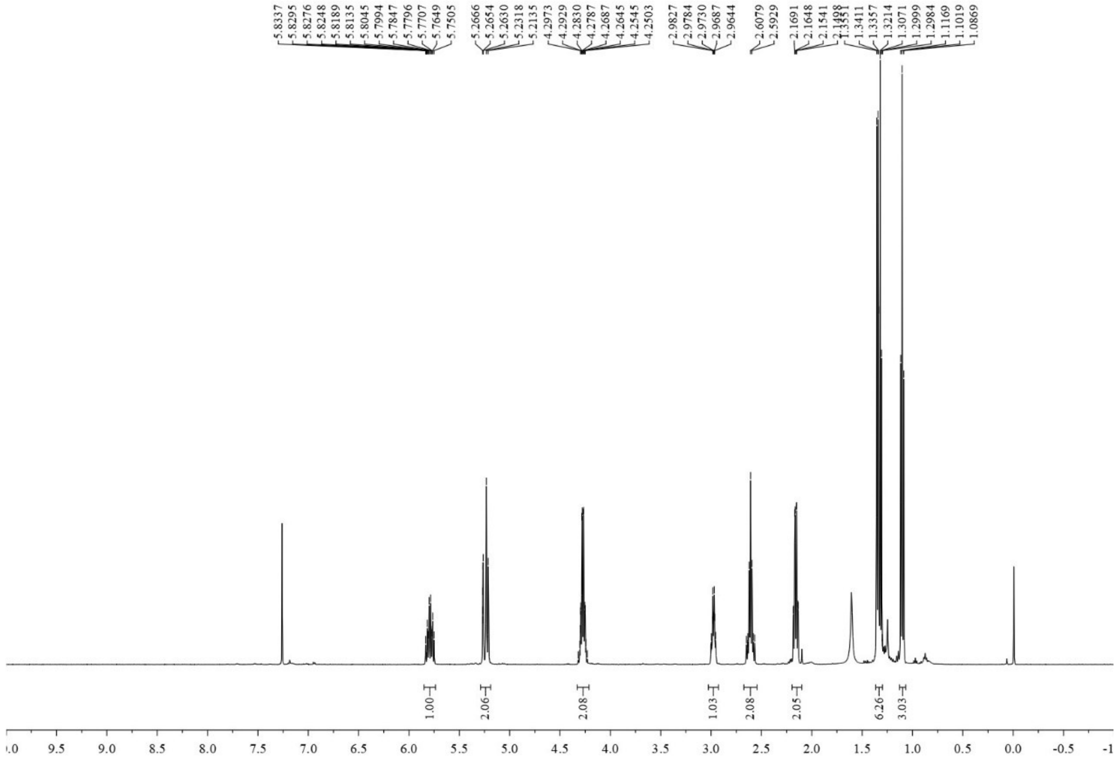

^{13}C NMR of (*R,S*)**-19 (125 MHz, CDCl$_3$)**

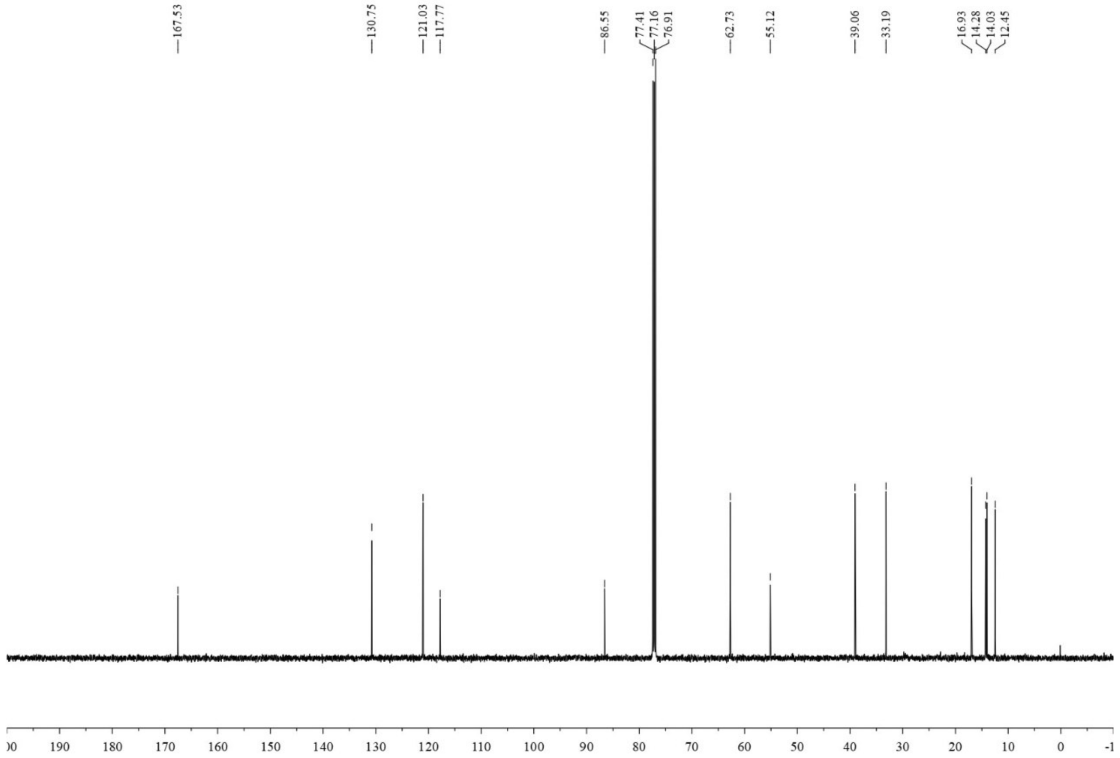

^{1}H NMR of (*S,R*)-**19 (500 MHz, CDCl$_3$)**

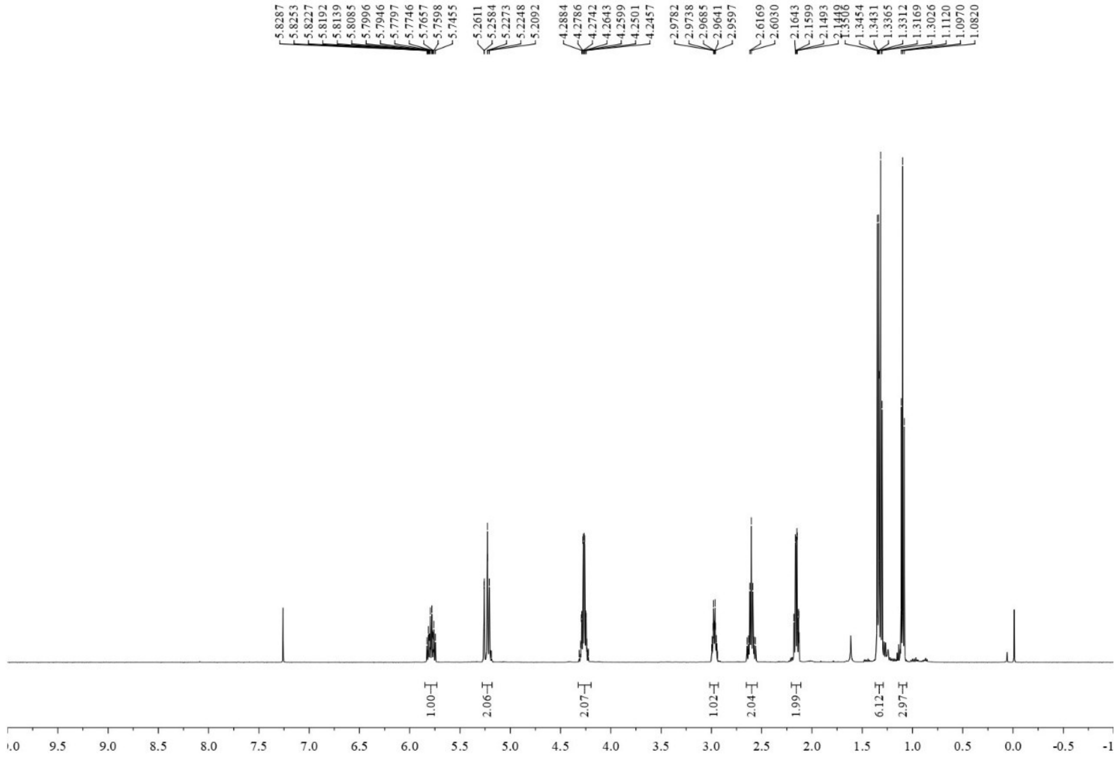

^{13}C NMR of (*S,R*)-**19 (125 MHz, CDCl$_3$)**

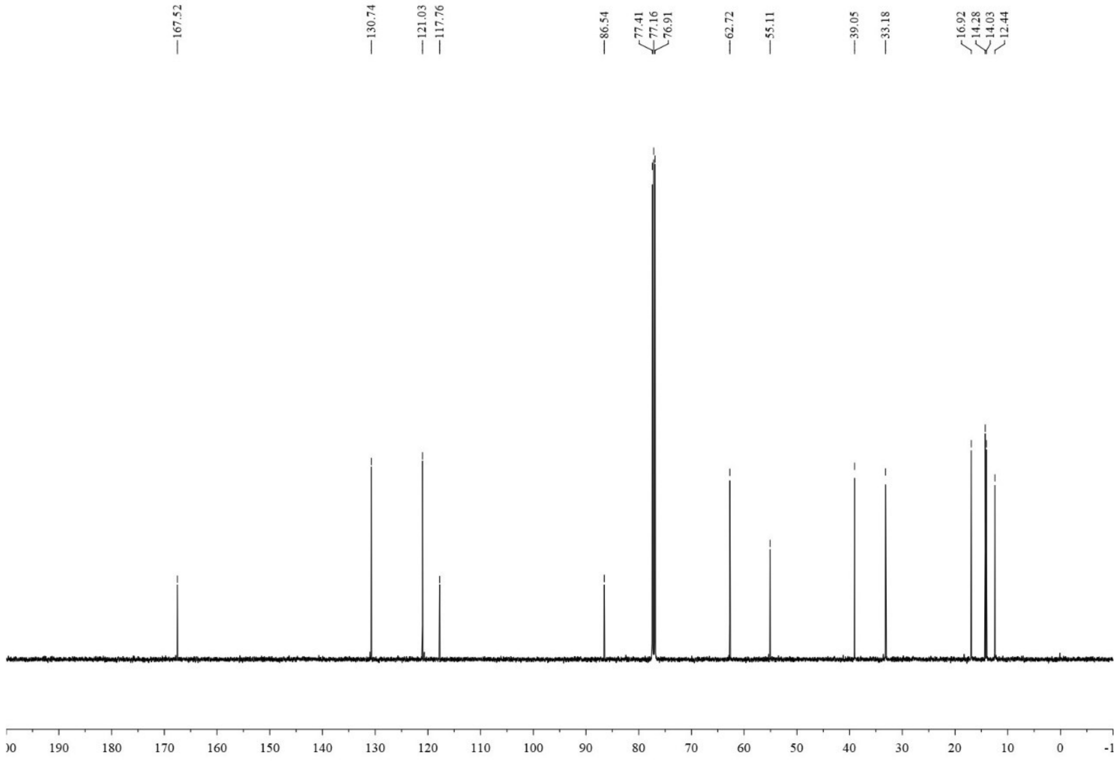

^{1}H NMR of (*R,R*)-**19 (500 MHz, CDCl$_3$)**

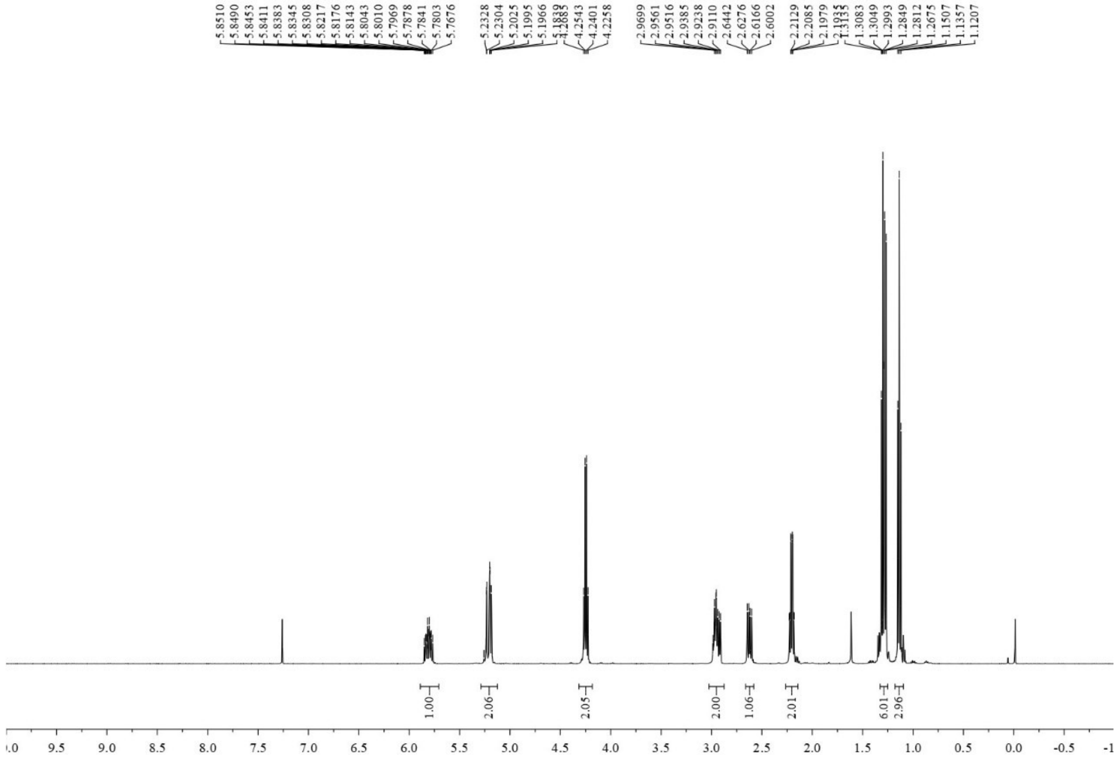

^{13}C NMR of (*R,R*)-**19 (125 MHz, CDCl$_3$)**

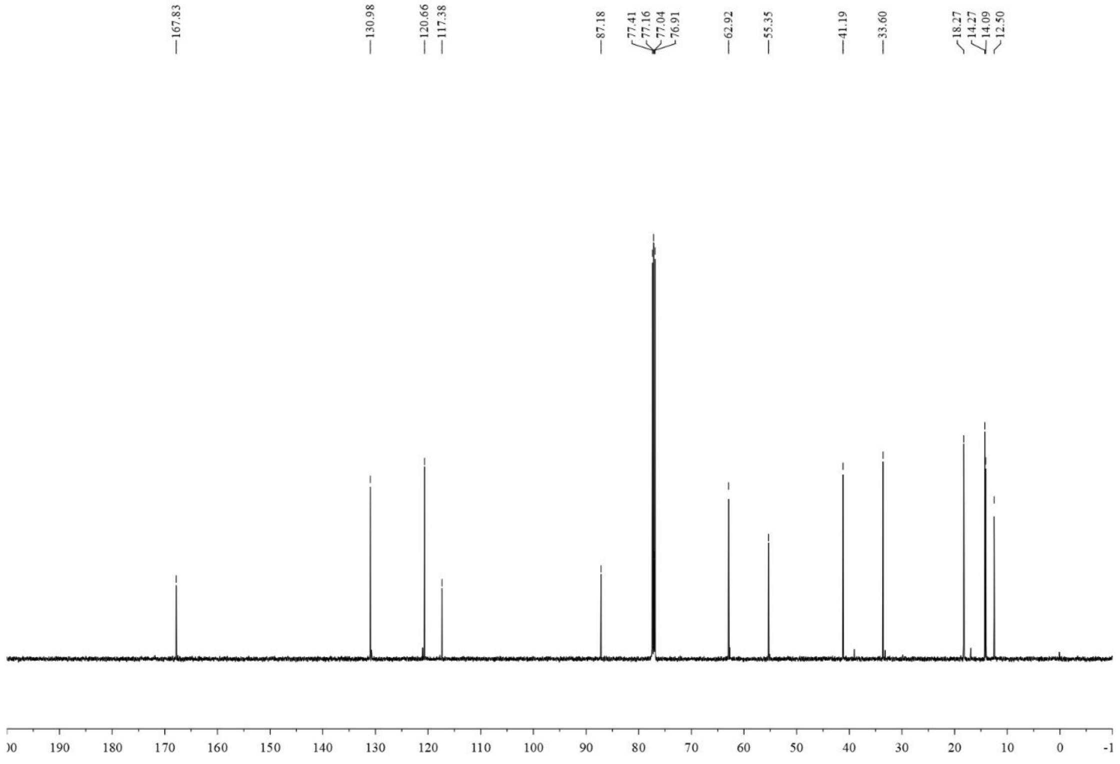

^{1}H NMR of (*S,S*)-**19 (500 MHz, CDCl$_3$)**

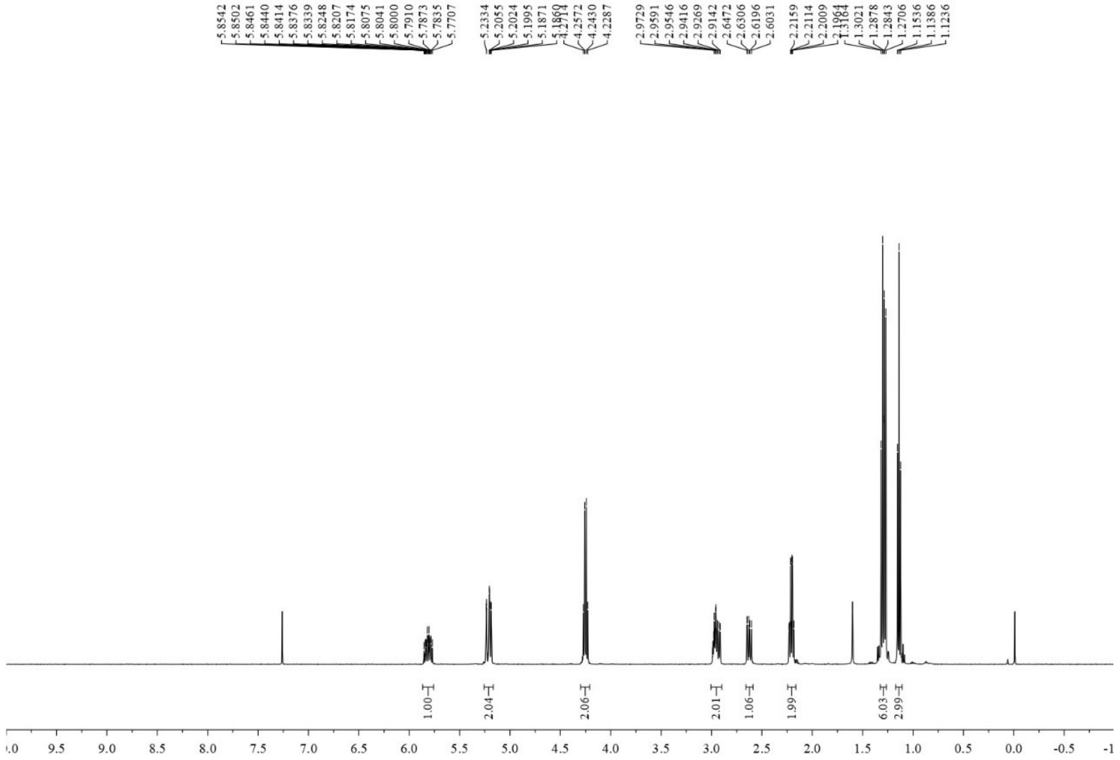

^{13}C NMR of (*S,S*)-**19 (125 MHz, CDCl$_3$)**

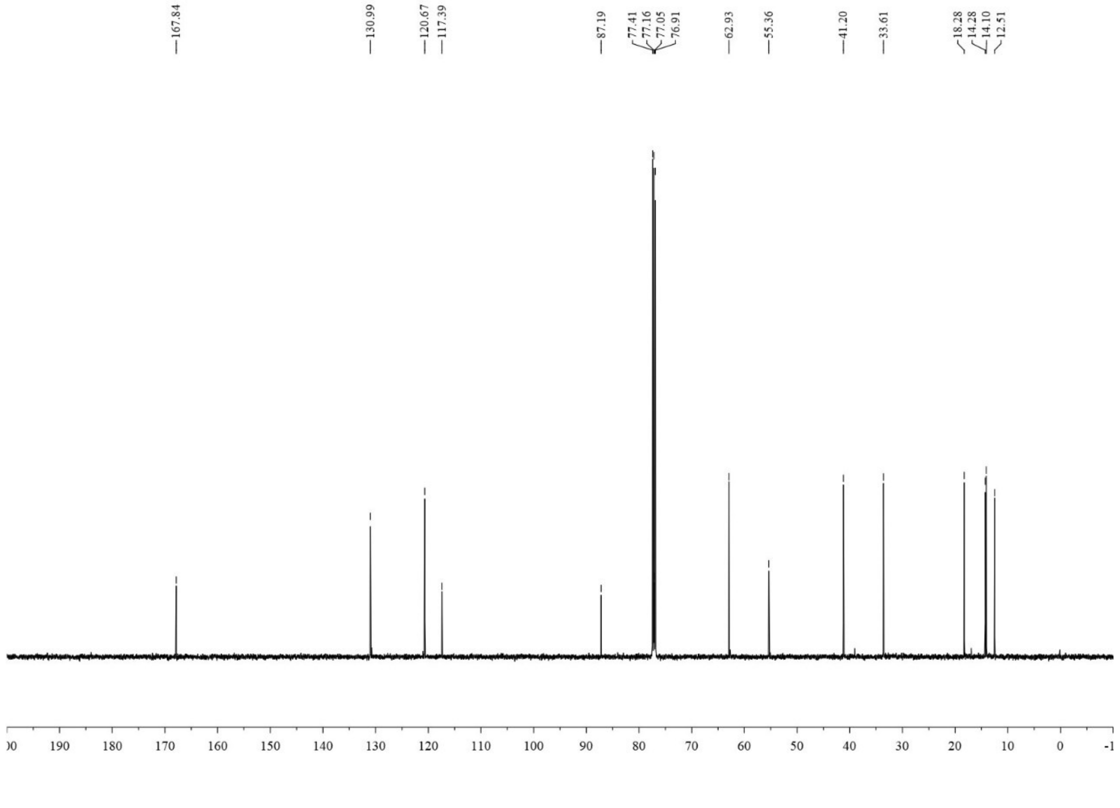

^{1}H NMR of (*R,S*)**-20 (500 MHz, CDCl$_3$)**

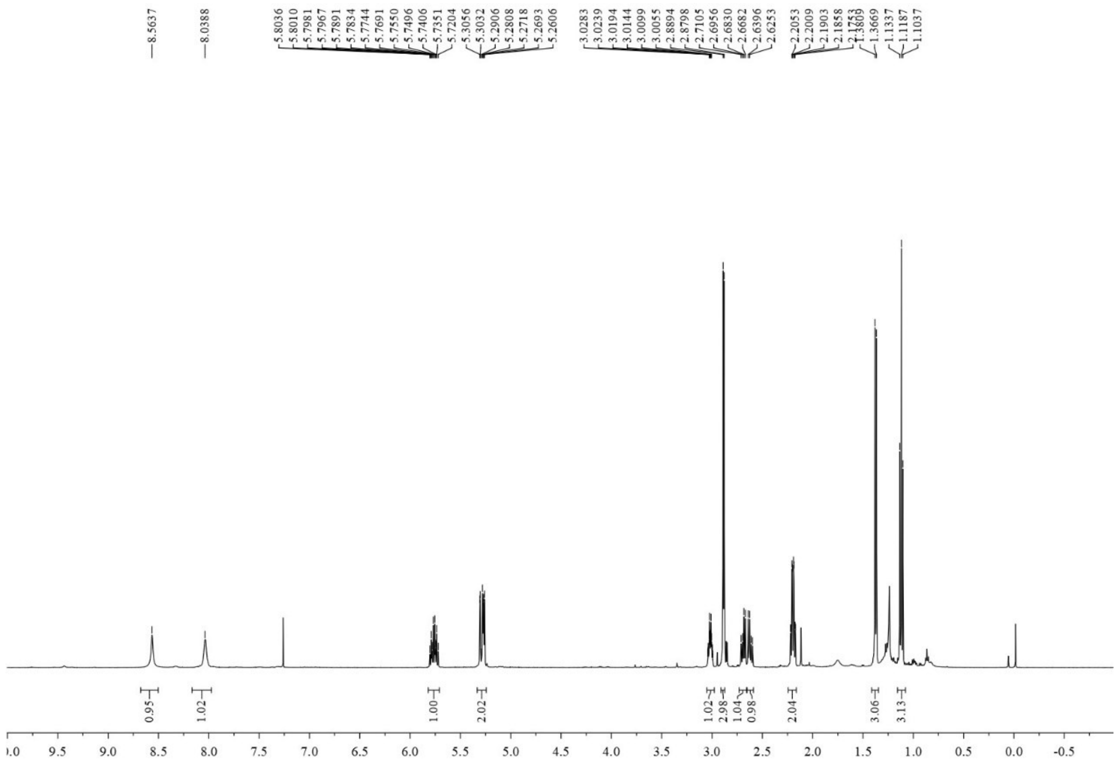

^{13}C NMR of (*R,S*)**-20 (125 MHz, CDCl$_3$)**

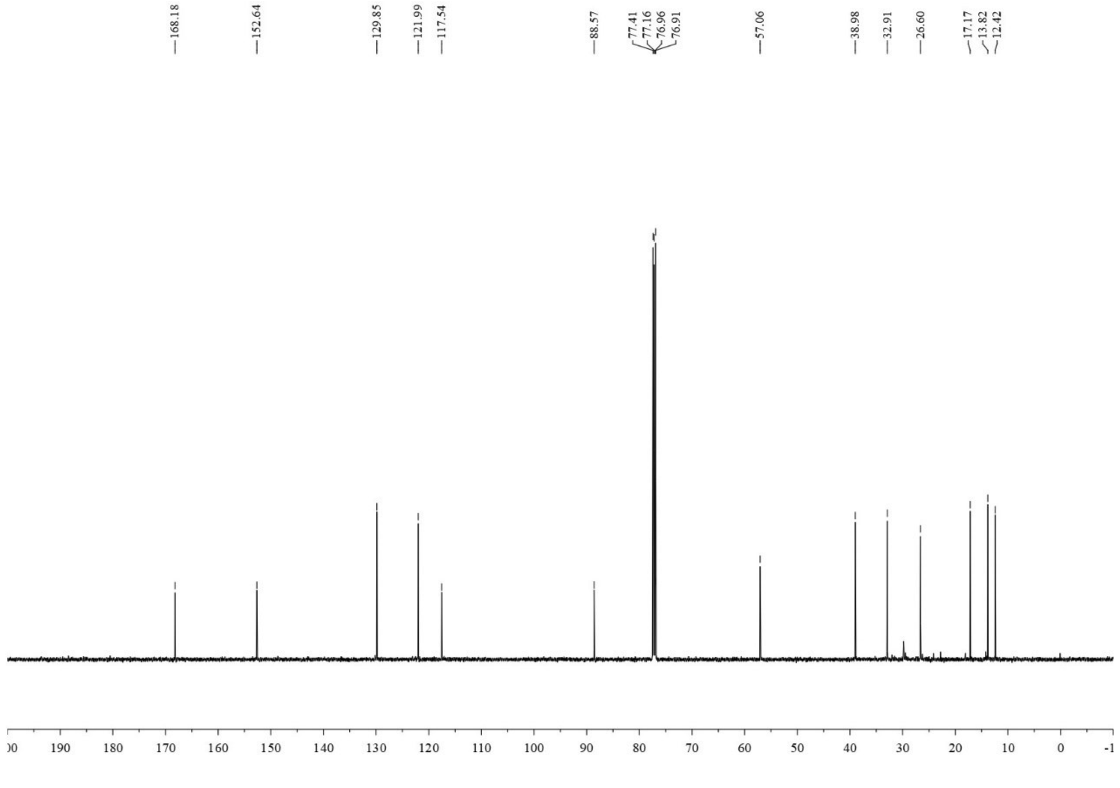

^{1}H NMR of (*S,R*)**-20 (500 MHz, CDCl$_3$)**

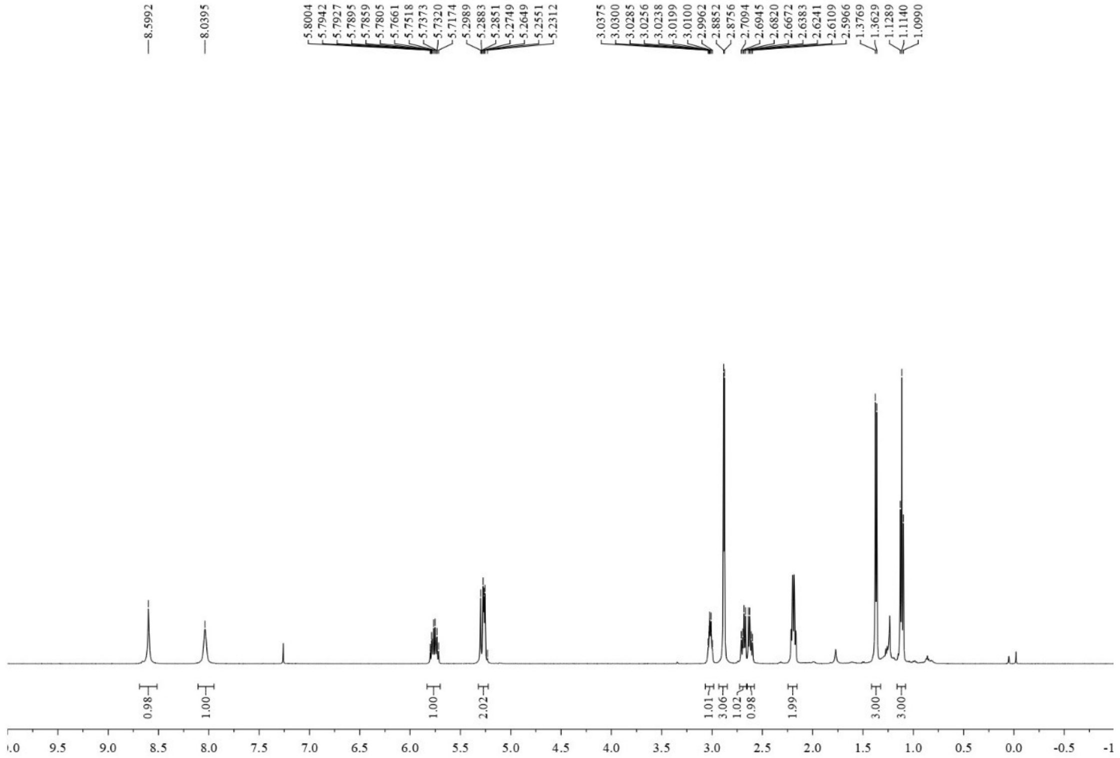

^{13}C NMR of (*S,R*)**-20 (125 MHz, CDCl$_3$)**

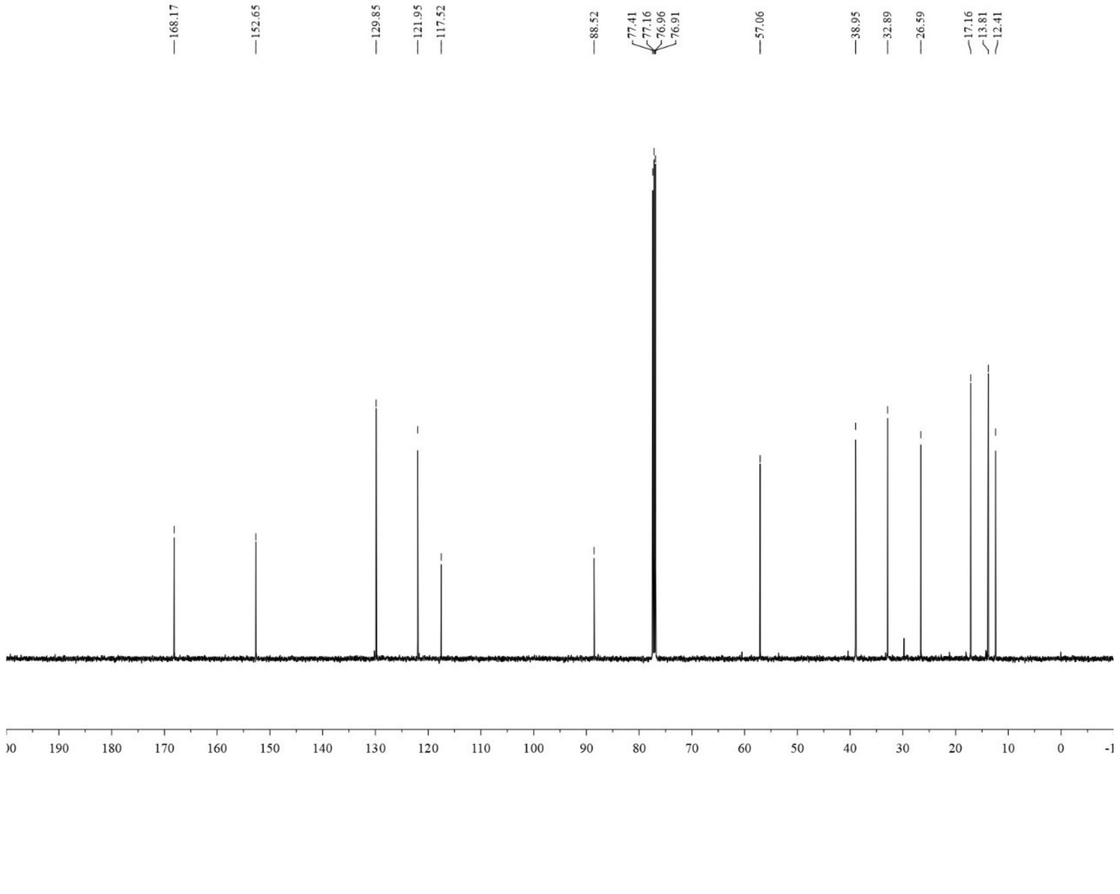

^{1}H NMR of (*R,R*)-**20 (500 MHz, CDCl$_3$)**

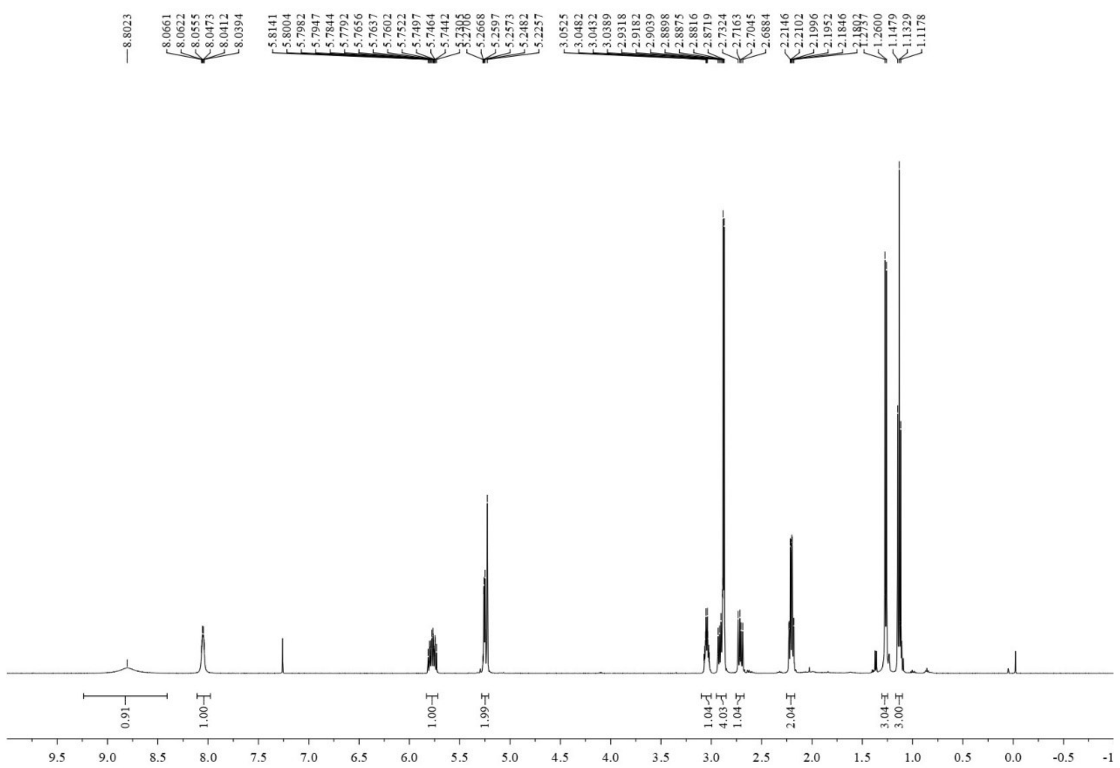

^{13}C NMR of (*R,R*)-**20 (125 MHz, CDCl$_3$)**

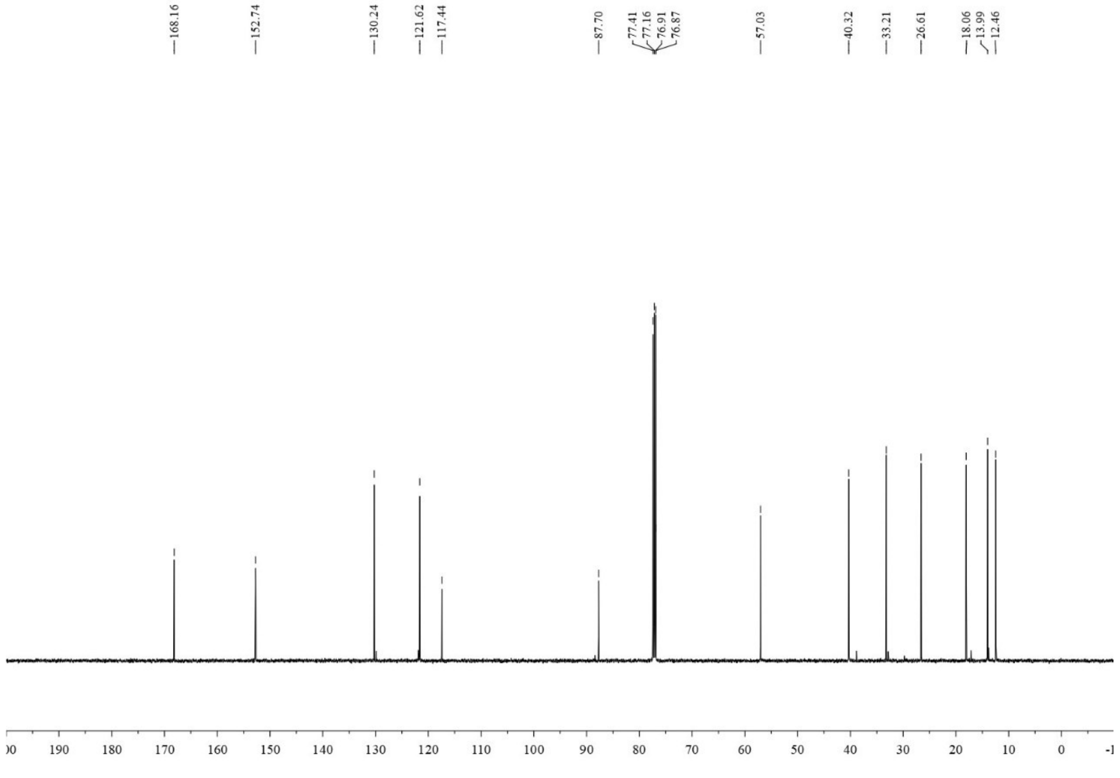

^{1}H NMR of (*S,S*)**-20 (500 MHz, $CDCl_3$)**

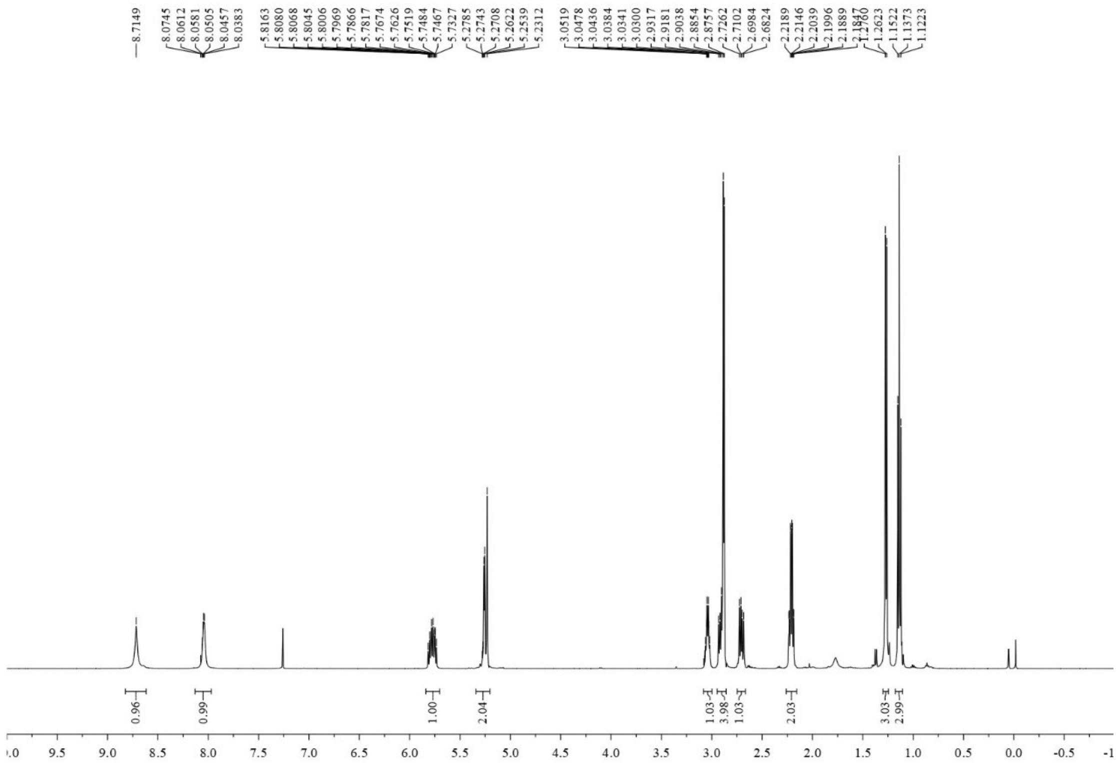

^{13}C NMR of (*S,S*)**-20 (125 MHz, $CDCl_3$)**

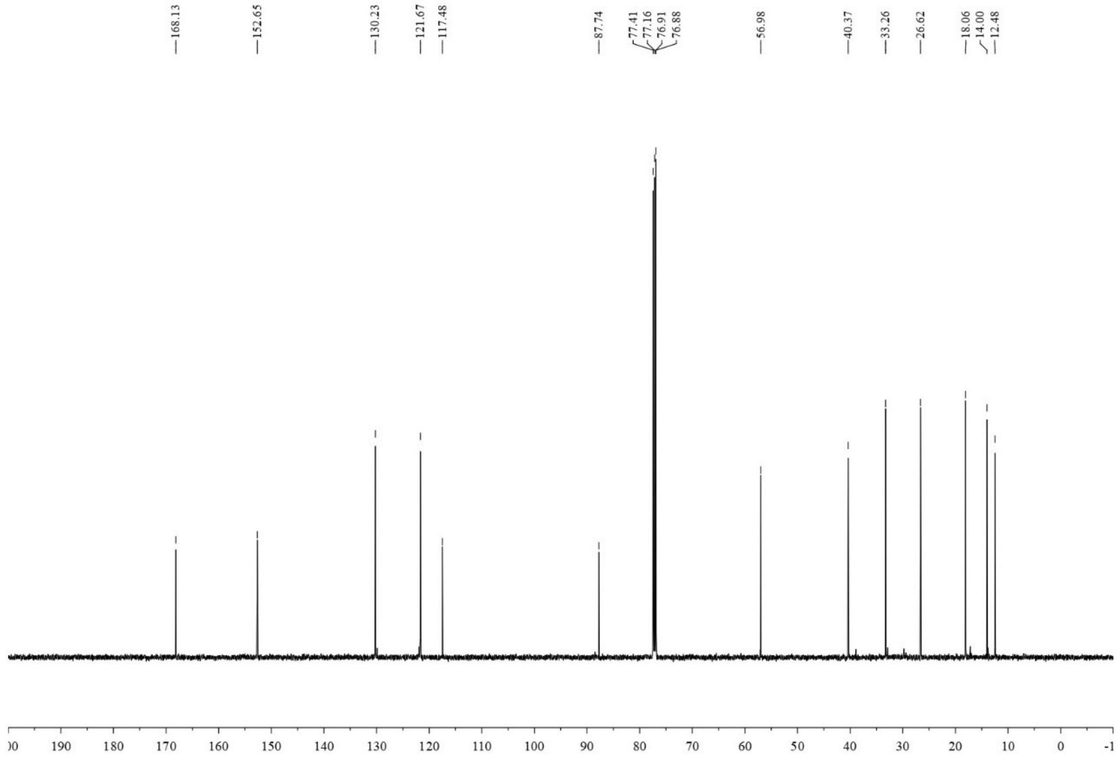

^{1}H NMR of (*S,S*)-**21 (500 MHz, CDCl$_3$)**

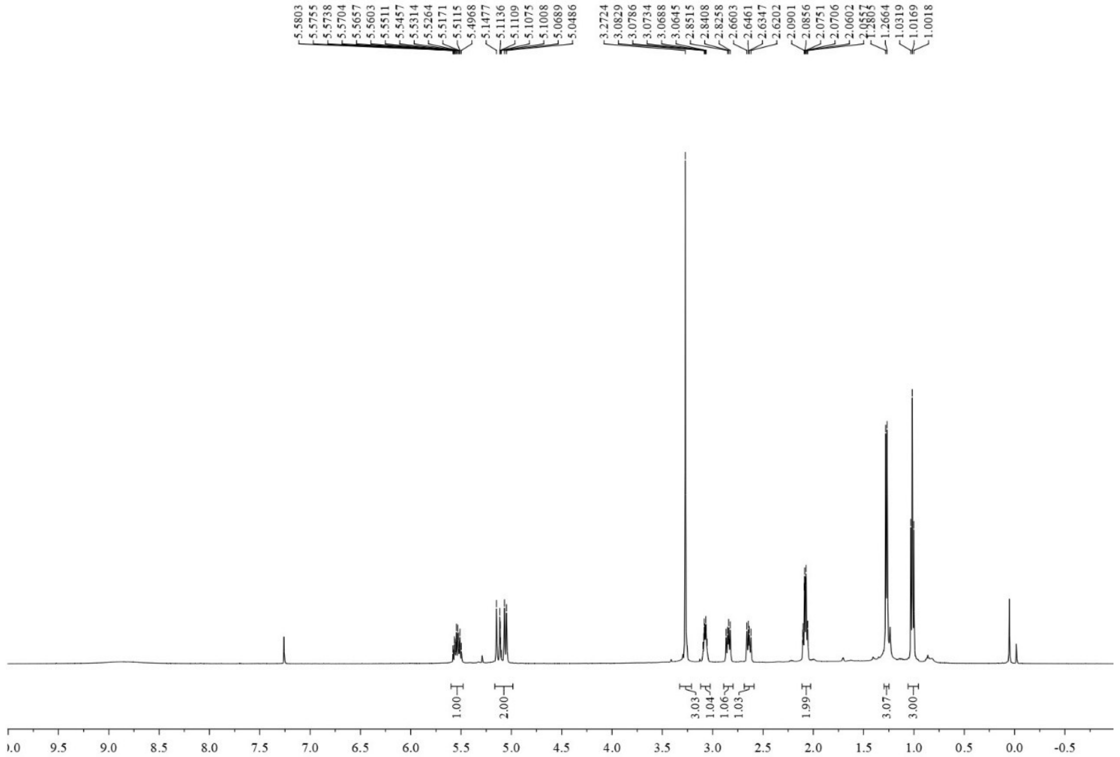

^{13}C NMR of (*S,S*)-**21 (125 MHz, CDCl$_3$)**

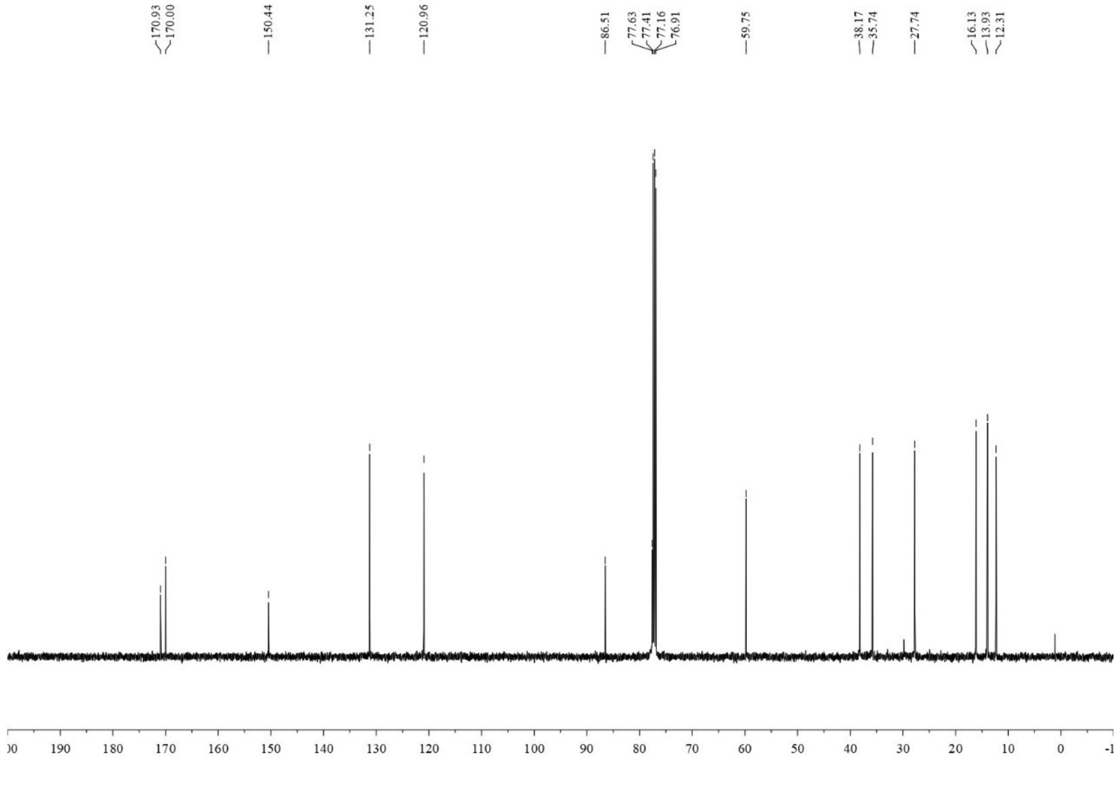

^{1}H NMR of (*R,R*)**-21 (500 MHz, CDCl$_3$)**

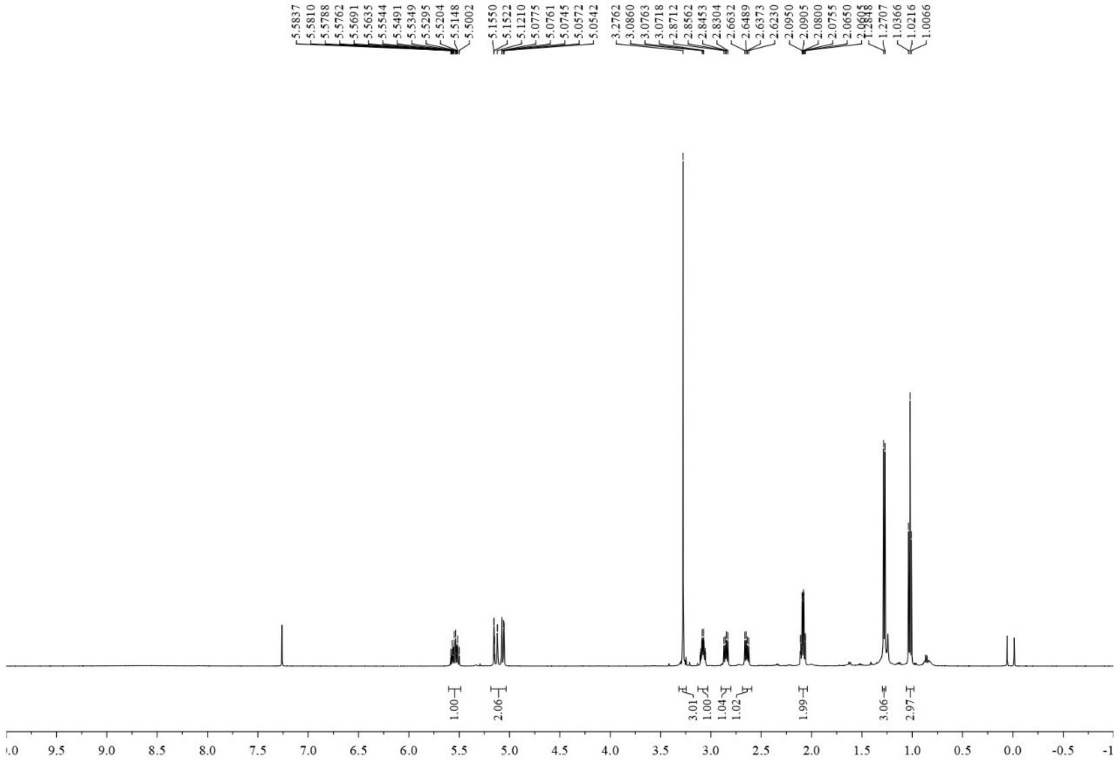

^{13}C NMR of (*R,R*)**-21 (125 MHz, CDCl$_3$)**

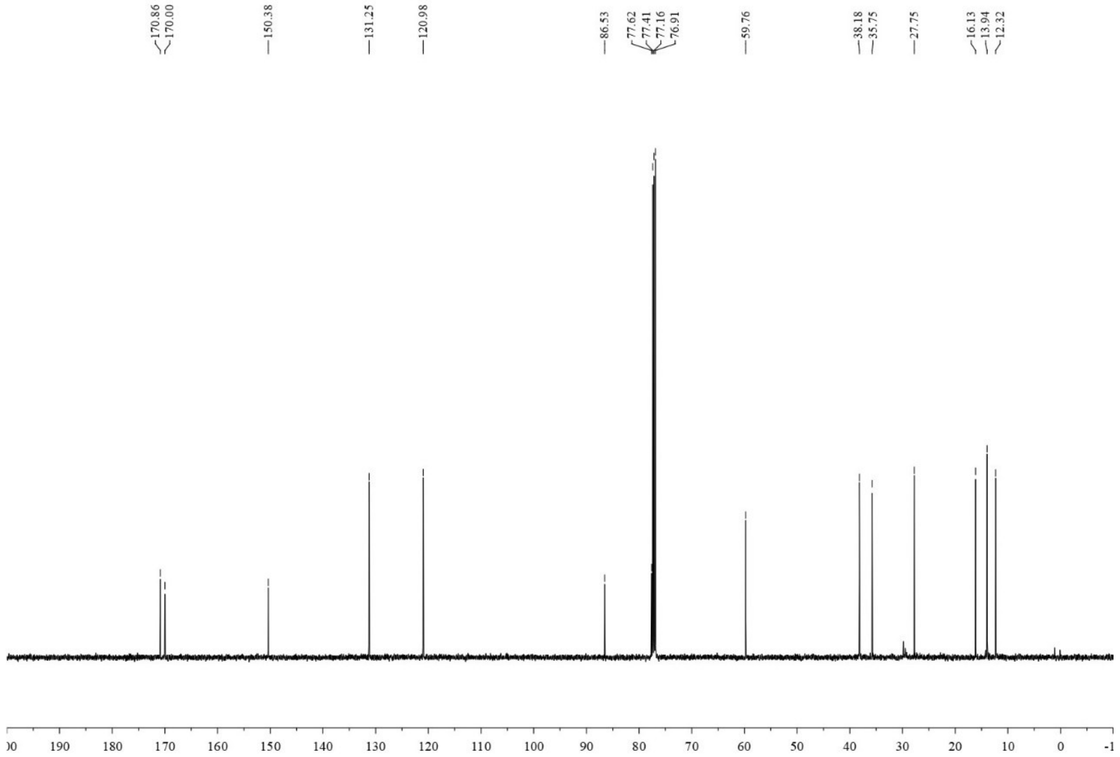

^{1}H NMR of (*S,R*)-21 (500 MHz, $CDCl_3$)

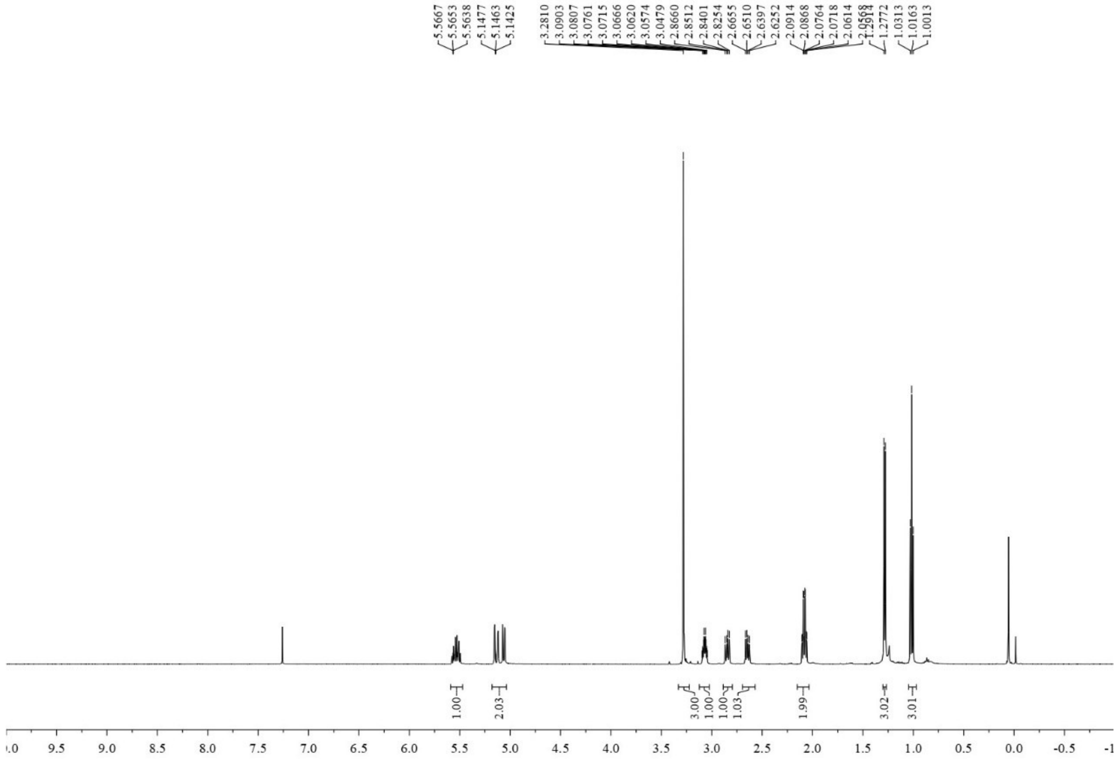

^{13}C NMR of (*S,R*)-21 (125 MHz, $CDCl_3$)

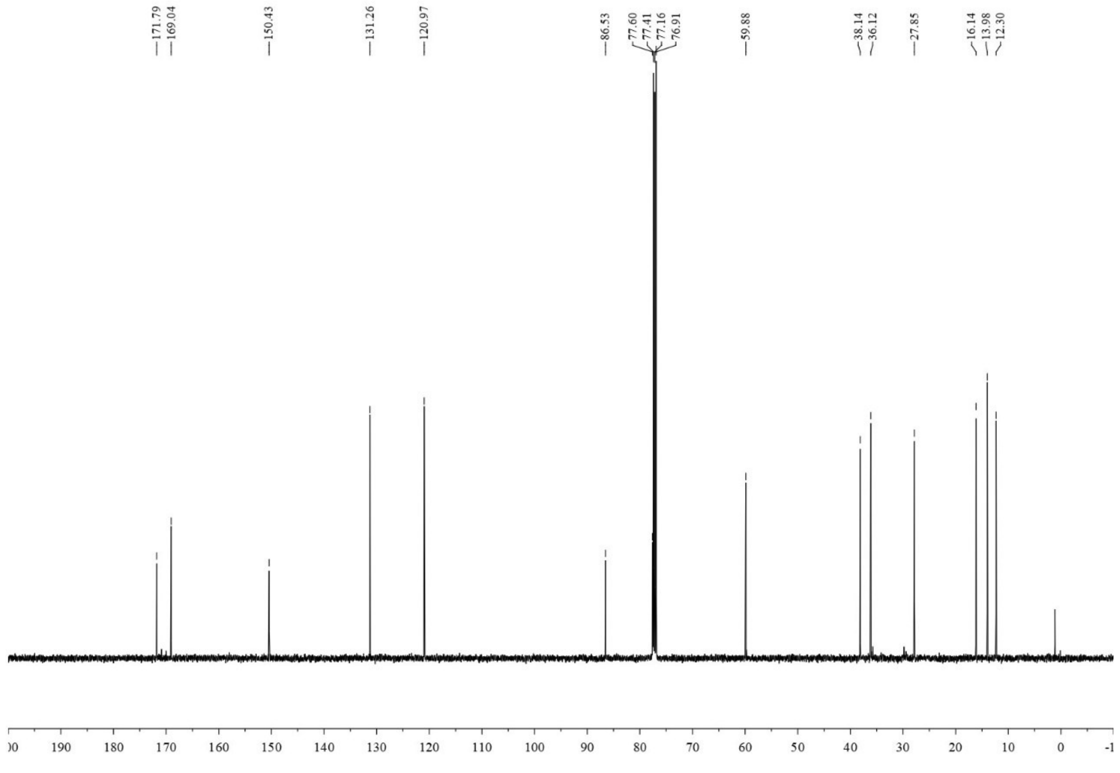

^{1}H NMR of (*R,S*)-**21 (500 MHz, CDCl$_3$)**

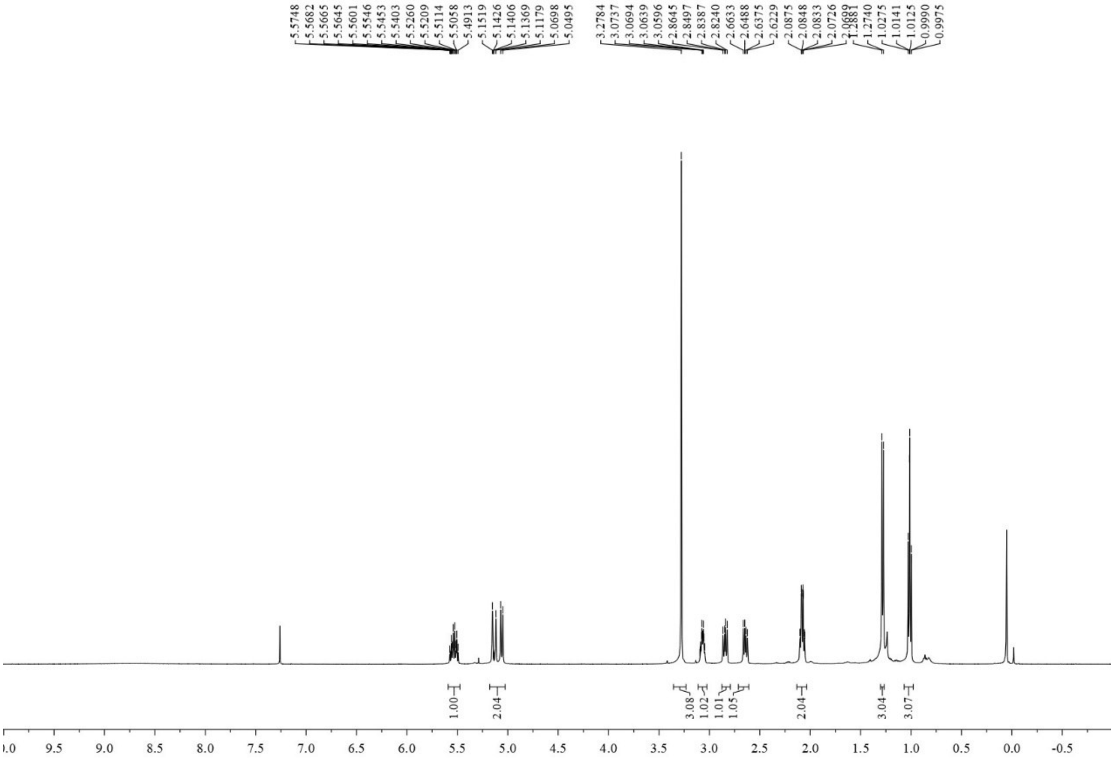

^{13}C NMR of (*R,S*)-**21 (125 MHz, CDCl$_3$)**

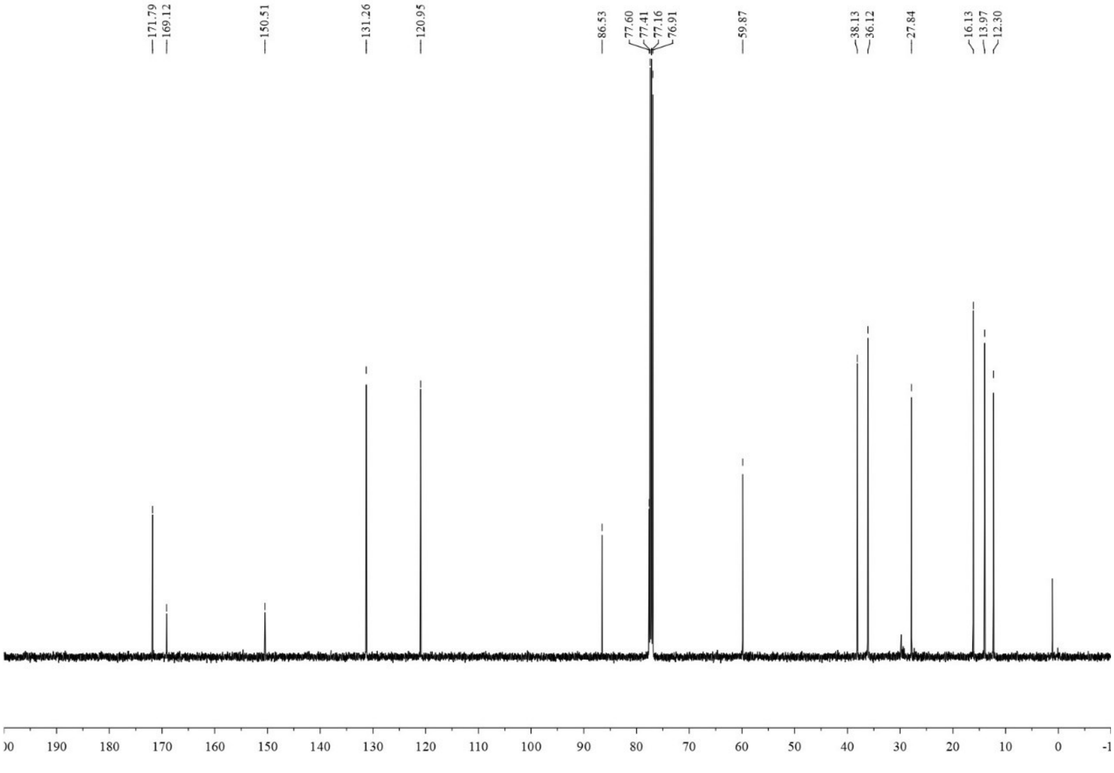

7. HPLC Traces

Rac-**3a**

SAMPLE INFORMATION

Sample Name:	cxh-10-25-4-rac-IC-1%-0.5ML	Acquired By:	System
Sample Type:	Unknown	Sample Set Name:	0710
Vial:	81	Acq. Method Set:	1% 05ML
Injection #:	1	Processing Method:	3a rac
Injection Volume:	10.00 ul	Channel Name:	254.0nm
Run Time:	35.0 Minutes	Proc. Chnl. Descr.:	2998 PDA 254.0 nm (2998
Date Acquired:	7/10/2023 5:01:03 PM CST		
Date Processed:	8/1/2023 11:05:08 AM CST		

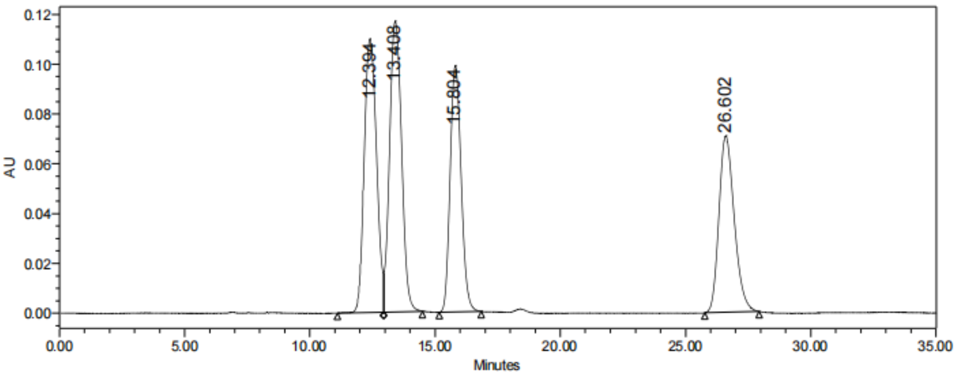

	RT	Area	% Area	Height
1	12.394	3758369	27.31	109828
2	13.408	3933860	28.58	116790
3	15.804	3038399	22.08	98967
4	26.602	3032262	22.03	70905

(*S*,*R*)-**3a**

SAMPLE INFORMATION

Sample Name:	cxh-10-86-2-asy-IC-1%-0.5ML	Acquired By:	System
Sample Type:	Unknown	Sample Set Name:	0710
Vial:	82	Acq. Method Set:	1% 05ML
Injection #:	1	Processing Method:	SR 3a
Injection Volume:	10.00 ul	Channel Name:	254.0nm
Run Time:	35.0 Minutes	Proc. Chnl. Descr.:	2998 PDA 254.0 nm (2998
Date Acquired:	7/10/2023 4:25:18 PM CST		
Date Processed:	8/1/2023 11:12:27 AM CST		

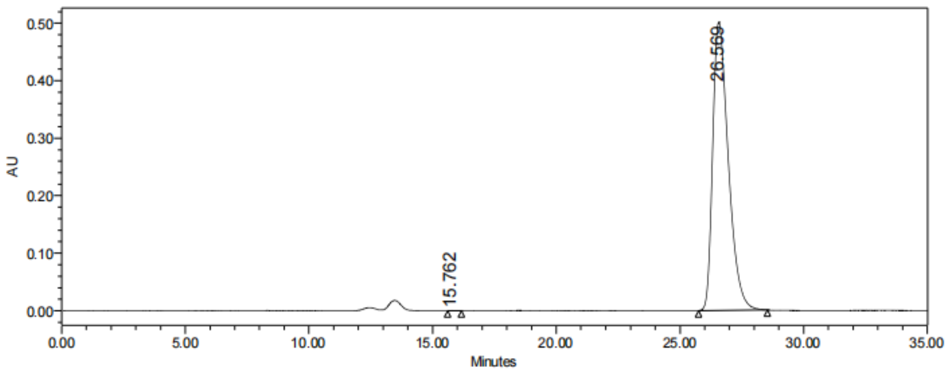

	RT	Area	% Area	Height
1	15.762	2592	0.01	152
2	26.569	22116675	99.99	500993

Rac-**3a**

SAMPLE INFORMATION

Sample Name:	cxh-10-25-4-rac-IC-1%-0.5ML	Acquired By:	System
Sample Type:	Unknown	Sample Set Name:	0710
Vial:	81	Acq. Method Set:	1% 05ML
Injection #:	1	Processing Method:	3a rac
Injection Volume:	10.00 ul	Channel Name:	254.0nm
Run Time:	35.0 Minutes	Proc. Chnl. Descr.:	2998 PDA 254.0 nm (2998
Date Acquired:	7/10/2023 5:01:03 PM CST		
Date Processed:	8/1/2023 11:05:08 AM CST		

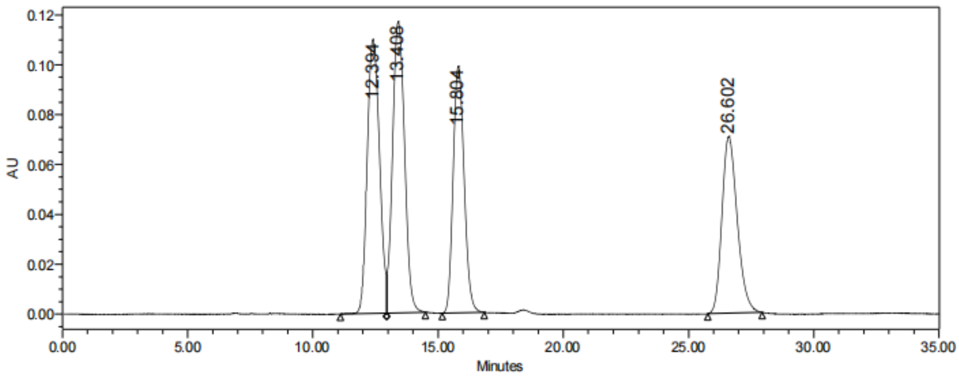

	RT	Area	% Area	Height
1	12.394	3758369	27.31	109828
2	13.408	3933860	28.58	116790
3	15.804	3038399	22.08	98967
4	26.602	3032262	22.03	70905

(*R*,*S*)-**3a**

SAMPLE INFORMATION

Sample Name:	cxh-10-96-2-asy-IC-1%-0.5ML	Acquired By:	System
Sample Type:	Unknown	Sample Set Name:	0710
Vial:	83	Acq. Method Set:	1% 05ML
Injection #:	1	Processing Method:	RS 3a
Injection Volume:	10.00 ul	Channel Name:	254.0nm
Run Time:	35.0 Minutes	Proc. Chnl. Descr.:	2998 PDA 254.0 nm (2998
Date Acquired:	7/10/2023 5:36:43 PM CST		
Date Processed:	8/1/2023 11:14:57 AM CST		

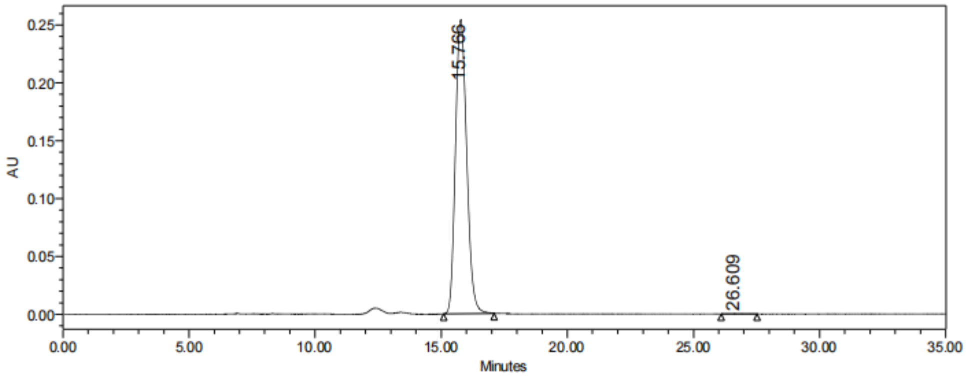

	RT	Area	% Area	Height
1	15.766	7925177	99.95	253592
2	26.609	4296	0.05	145

Rac-**3a**

SAMPLE INFORMATION

Sample Name:	cxh-10-25-4-rac-IC-1%-0.5ML	Acquired By:	System
Sample Type:	Unknown	Sample Set Name:	0710
Vial:	81	Acq. Method Set:	1% 05ML
Injection #:	1	Processing Method:	3a rac
Injection Volume:	10.00 ul	Channel Name:	254.0nm
Run Time:	35.0 Minutes	Proc. Chnl. Descr.:	2998 PDA 254.0 nm (2998
Date Acquired:	7/10/2023 5:01:03 PM CST		
Date Processed:	8/1/2023 11:05:08 AM CST		

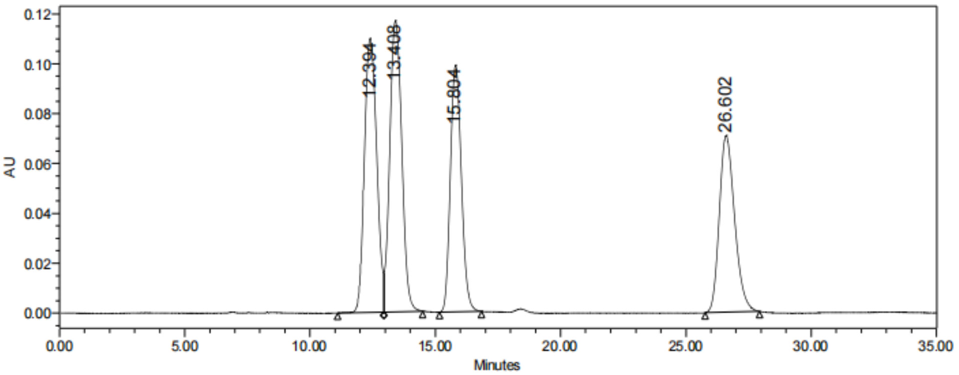

	RT	Area	% Area	Height
1	12.394	3758369	27.31	109828
2	13.408	3933860	28.58	116790
3	15.804	3038399	22.08	98967
4	26.602	3032262	22.03	70905

(*R*,*R*)-**3a**

SAMPLE INFORMATION

Sample Name:	cxh-11-30-4-asy-IC-1%-0.5ML	Acquired By:	System
Sample Type:	Unknown	Sample Set Name:	0710
Vial:	85	Acq. Method Set:	1% 05ML
Injection #:	1	Processing Method:	RR 3a
Injection Volume:	10.00 ul	Channel Name:	254.0nm
Run Time:	35.0 Minutes	Proc. Chnl. Descr.:	2998 PDA 254.0 nm (2998
Date Acquired:	7/10/2023 6:48:07 PM CST		
Date Processed:	8/1/2023 11:18:41 AM CST		

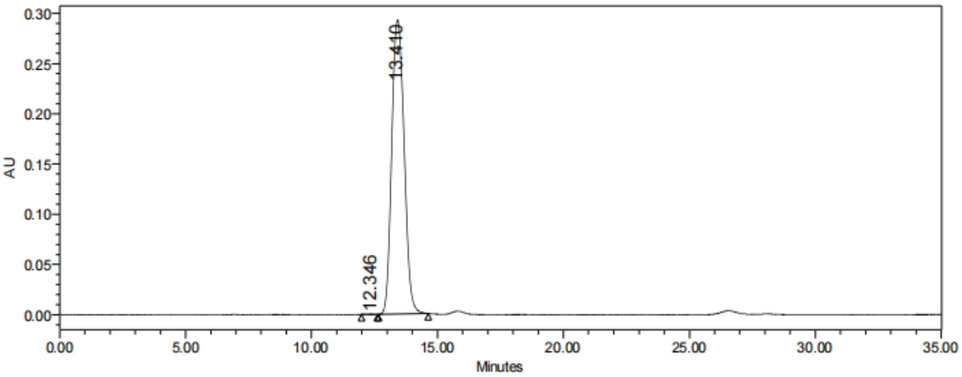

	RT	Area	% Area	Height
1	12.346	1594	0.02	110
2	13.410	10095026	99.98	292280

Rac-**3a**

SAMPLE INFORMATION

Sample Name:	cxh-10-25-4-rac-IC-1%-0.5ML	Acquired By:	System
Sample Type:	Unknown	Sample Set Name:	0710
Vial:	81	Acq. Method Set:	1% 05ML
Injection #:	1	Processing Method:	3a rac
Injection Volume:	10.00 ul	Channel Name:	254.0nm
Run Time:	35.0 Minutes	Proc. Chnl. Descr.:	2998 PDA 254.0 nm (2998
Date Acquired:	7/10/2023 5:01:03 PM CST		
Date Processed:	8/1/2023 11:05:08 AM CST		

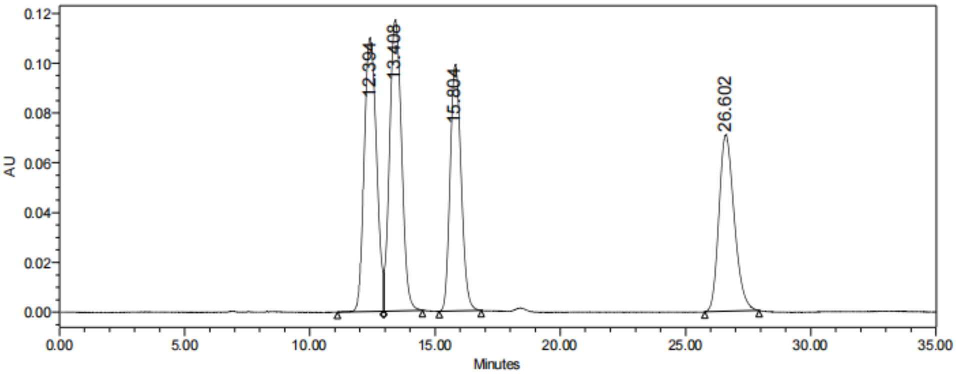

	RT	Area	% Area	Height
1	12.394	3758369	27.31	109828
2	13.408	3933860	28.58	116790
3	15.804	3038399	22.08	98967
4	26.602	3032262	22.03	70905

(*S*,*S*)-**3a**

SAMPLE INFORMATION

Sample Name:	cxh-11-30-1-asy-IC-1%-0.5ML	Acquired By:	System
Sample Type:	Unknown	Sample Set Name:	0710
Vial:	84	Acq. Method Set:	1% 05ML
Injection #:	1	Processing Method:	SS 3a
Injection Volume:	10.00 ul	Channel Name:	254.0nm
Run Time:	35.0 Minutes	Proc. Chnl. Descr.:	2998 PDA 254.0 nm (2998
Date Acquired:	7/10/2023 6:12:24 PM CST		
Date Processed:	8/1/2023 11:17:31 AM CST		

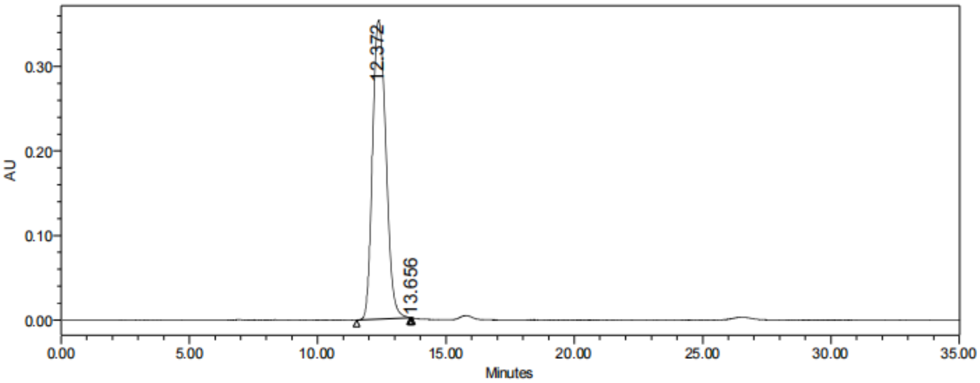

	RT	Area	% Area	Height
1	12.372	12824160	100.00	353289
2	13.656	69	0.00	-42

Rac-**3b**

SAMPLE INFORMATION

Sample Name:	cxh-10-88-2-rac-IE-1%-0.5ML	Acquired By:	System
Sample Type:	Control	Sample Set Name:	
Vial:	104	Acq. Method Set:	1% 05ML
Injection #:	1	Processing Method:	3b rac
Injection Volume:	10.00 ul	Channel Name:	254.0nm
Run Time:	30.0 Minutes	Proc. Chnl. Descr.:	2998 PDA 254.0 nm (2998
Date Acquired:	10/28/2022 10:25:22 PM CST		
Date Processed:	7/31/2023 9:25:33 PM CST		

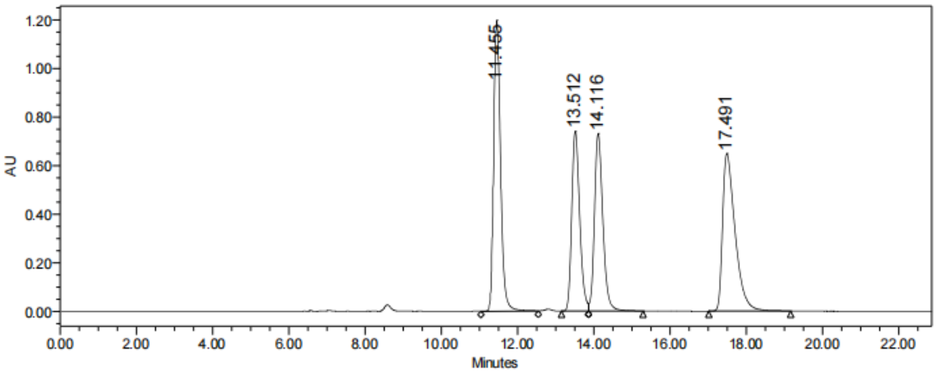

	RT	Area	% Area	Height
1	11.455	14771751	28.56	1197266
2	13.512	10773543	20.83	740435
3	14.116	11261682	21.78	730040
4	17.491	14908318	28.83	648535

Asy-**3b**

SAMPLE INFORMATION

Sample Name:	cxh-10-88-2-asy-IE-1%-0.5ML	Acquired By:	System
Sample Type:	Control	Sample Set Name:	
Vial:	19	Acq. Method Set:	1% 05ML
Injection #:	1	Processing Method:	3b asy
Injection Volume:	10.00 ul	Channel Name:	254.0nm
Run Time:	25.0 Minutes	Proc. Chnl. Descr.:	2998 PDA 254.0 nm (2998
Date Acquired:	10/28/2022 10:51:00 PM CST		
Date Processed:	7/31/2023 9:28:29 PM CST		

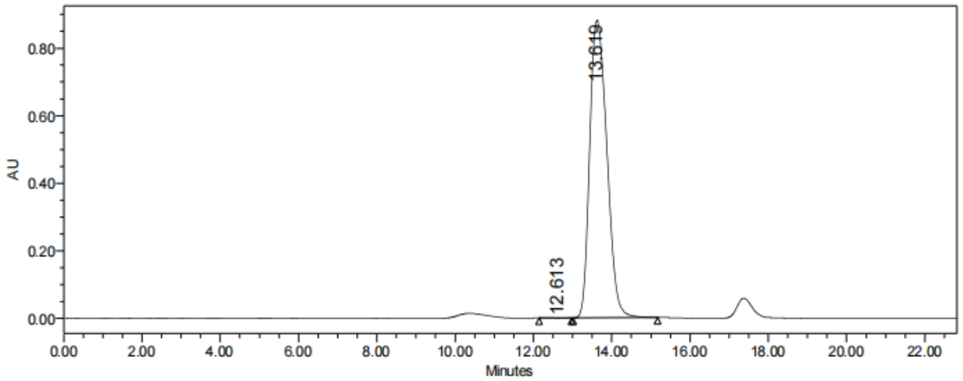

	RT	Area	% Area	Height
1	12.613	8726	0.03	-386
2	13.619	26992883	99.97	880328

Rac-**3c**

SAMPLE INFORMATION

Sample Name:	cxh-10-88-4-IC-rac-1%-0.5ML	Acquired By:	System
Sample Type:	Control	Sample Set Name:	
Vial:	17	Acq. Method Set:	1% 05ML
Injection #:	1	Processing Method:	3c rac
Injection Volume:	10.00 ul	Channel Name:	254.0nm
Run Time:	50.0 Minutes	Proc. Chnl. Descr.:	2998 PDA 254.0 nm (2998
Date Acquired:	10/24/2022 2:13:35 PM CST		
Date Processed:	7/31/2023 9:16:33 PM CST		

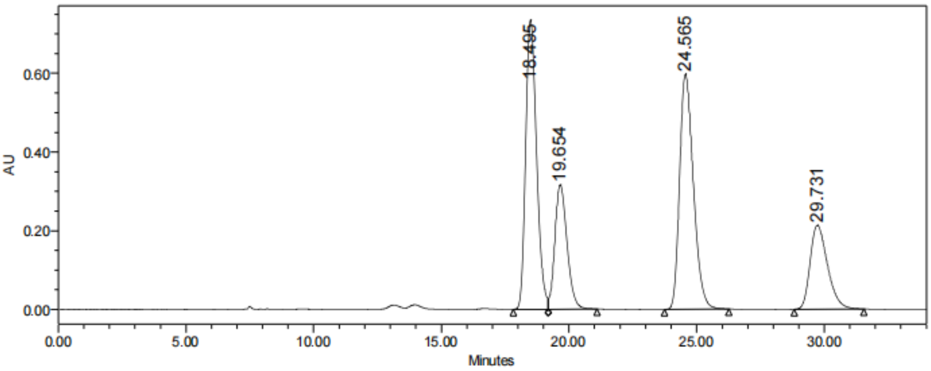

	RT	Area	% Area	Height
1	18.495	21892184	33.91	734630
2	19.654	10398927	16.11	315859
3	24.565	22154197	34.32	597560
4	29.731	10111531	15.66	213465

Asy-**3c**

SAMPLE INFORMATION

Sample Name:	cxh-10-88-4-IC-asy-1%-0.5ML	Acquired By:	System
Sample Type:	Unknown	Sample Set Name:	10261
Vial:	115	Acq. Method Set:	1% 05ML
Injection #:	1	Processing Method:	3c asy
Injection Volume:	10.00 ul	Channel Name:	254.0nm
Run Time:	38.0 Minutes	Proc. Chnl. Descr.:	2998 PDA 254.0 nm (2998
Date Acquired:	10/26/2022 4:05:52 PM CST		
Date Processed:	7/31/2023 9:19:59 PM CST		

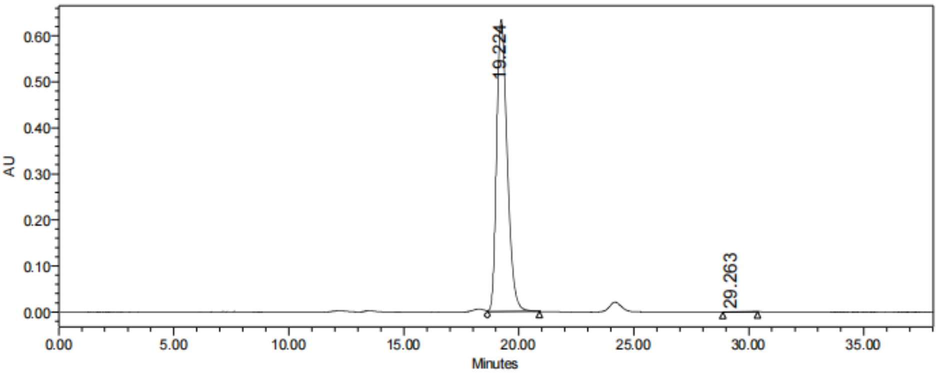

	RT	Area	% Area	Height
1	19.224	20325986	99.98	633459
2	29.263	3631	0.02	-130

Rac-**3d**

SAMPLE INFORMATION

Sample Name:	cxh-10-87-1-rac-IC-1%-0.5ML	Acquired By:	System
Sample Type:	Unknown	Sample Set Name:	10294
Vial:	44	Acq. Method Set:	1% 05ML
Injection #:	1	Processing Method:	3d rac
Injection Volume:	10.00 ul	Channel Name:	254.0nm
Run Time:	35.0 Minutes	Proc. Chnl. Descr.:	2998 PDA 254.0 nm (2998
Date Acquired:	10/29/2022 1:14:59 PM CST		
Date Processed:	7/31/2023 8:40:32 PM CST		

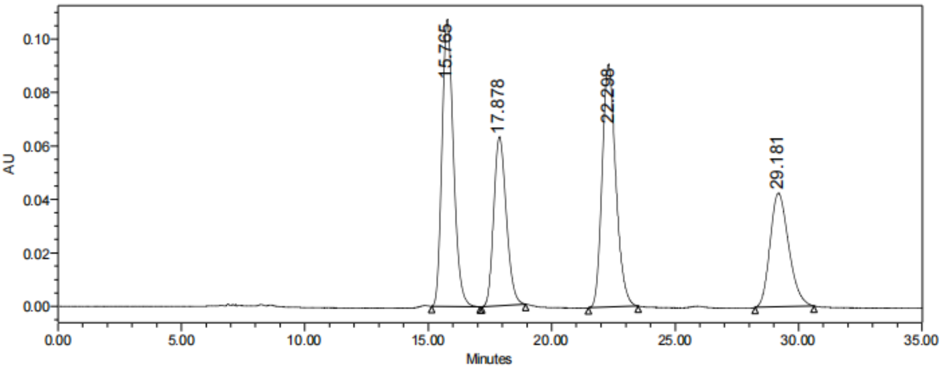

	RT	Area	% Area	Height
1	15.765	3483819	30.41	107215
2	17.878	2244684	19.60	63174
3	22.298	3483553	30.41	90841
4	29.181	2242541	19.58	42540

Asy-**3d**

SAMPLE INFORMATION

Sample Name:	cxh-10-87-1-asy-IC-1%-0.5ML	Acquired By:	System
Sample Type:	Unknown	Sample Set Name:	10293
Vial:	44	Acq. Method Set:	1% 05ML
Injection #:	1	Processing Method:	3d asy
Injection Volume:	10.00 ul	Channel Name:	254.0nm
Run Time:	35.0 Minutes	Proc. Chnl. Descr.:	2998 PDA 254.0 nm (2998
Date Acquired:	10/29/2022 12:02:53 PM CST		
Date Processed:	7/31/2023 8:42:48 PM CST		

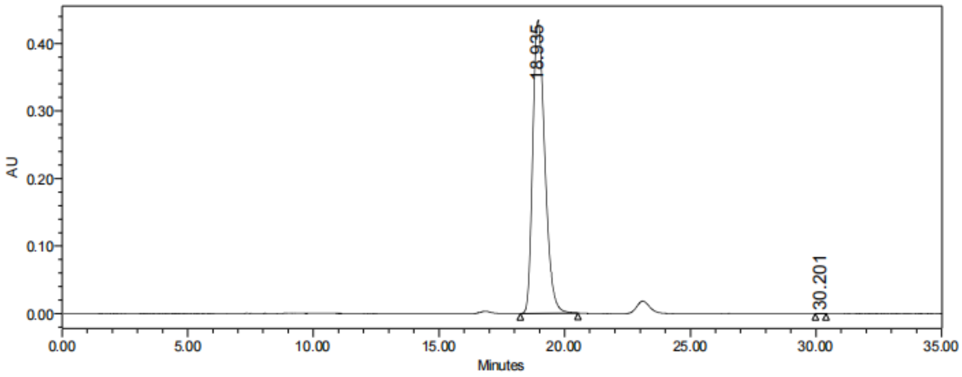

	RT	Area	% Area	Height
1	18.935	14577784	100.00	433667
2	30.201	284	0.00	-30

Rac-**3e**

SAMPLE INFORMATION

Sample Name:	cxh-10-88-5-IC-rac-1%-0.5ML	Acquired By:	System
Sample Type:	Control	Sample Set Name:	
Vial:	18	Acq. Method Set:	1% 05ML
Injection #:	1	Processing Method:	3e rac
Injection Volume:	10.00 ul	Channel Name:	254.0nm
Run Time:	50.0 Minutes	Proc. Chnl. Descr.:	2998 PDA 254.0 nm (2998
Date Acquired:	10/24/2022 2:48:48 PM CST		
Date Processed:	7/31/2023 9:51:53 PM CST		

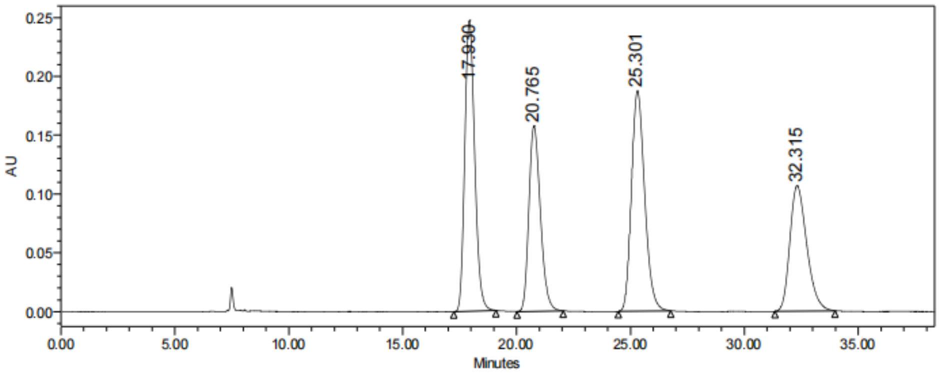

	RT	Area	% Area	Height
1	17.930	7526960	28.79	247227
2	20.765	5556904	21.26	157427
3	25.301	7522230	28.77	187210
4	32.315	5535643	21.18	106550

Asy-**3e**

SAMPLE INFORMATION

Sample Name:	cxh-10-88-5-asy-IC-1%-0.5ML	Acquired By:	System
Sample Type:	Control	Sample Set Name:	
Vial:	73	Acq. Method Set:	1% 05ML
Injection #:	1	Processing Method:	3e asy
Injection Volume:	10.00 ul	Channel Name:	254.0nm
Run Time:	40.0 Minutes	Proc. Chnl. Descr.:	2998 PDA 254.0 nm (2998
Date Acquired:	10/26/2022 9:45:03 PM CST		
Date Processed:	7/31/2023 9:57:11 PM CST		

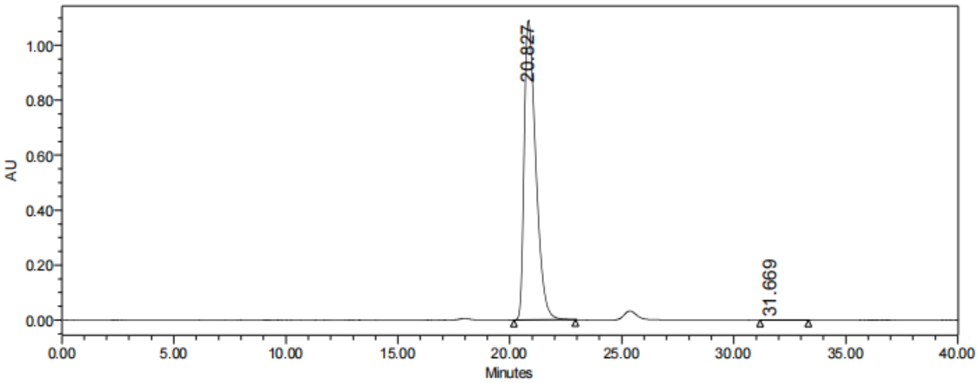

	RT	Area	% Area	Height
1	20.827	39409687	99.96	1088635
2	31.669	15379	0.04	-224

Rac-**3f**

SAMPLE INFORMATION

Sample Name:	cxh-10-87-2-IC-rac-1%-0.5ML	Acquired By:	System
Sample Type:	Unknown	Sample Set Name:	10252
Vial:	112	Acq. Method Set:	1% 05ML
Injection #:	1	Processing Method:	3f rac
Injection Volume:	10.00 ul	Channel Name:	254.0nm
Run Time:	35.0 Minutes	Proc. Chnl. Descr.:	2998 PDA 254.0 nm (2998
Date Acquired:	10/25/2022 6:48:28 PM CST		
Date Processed:	7/31/2023 8:46:26 PM CST		

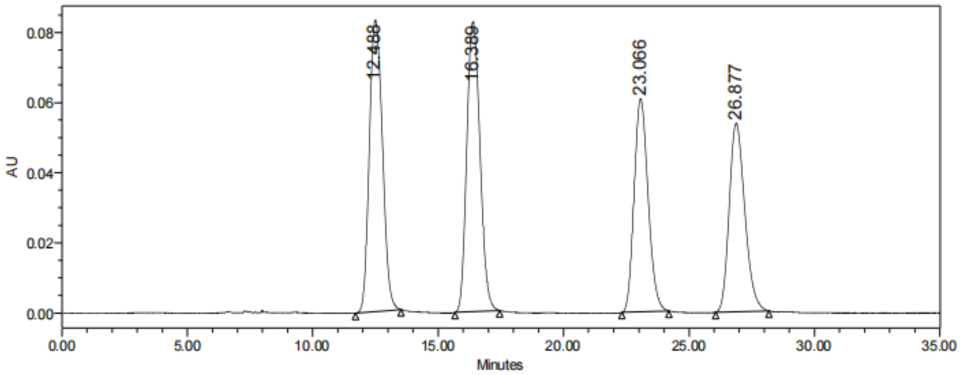

	RT	Area	% Area	Height
1	12.488	3026024	28.17	83010
2	16.389	3036528	28.26	82584
3	23.066	2341322	21.79	60748
4	26.877	2339601	21.78	53721

Asy-**3f**

SAMPLE INFORMATION

Sample Name:	cxh-10-87-2-IC-asy-1%-0.5ML	Acquired By:	System
Sample Type:	Unknown	Sample Set Name:	10251
Vial:	88	Acq. Method Set:	1% 05ML
Injection #:	1	Processing Method:	3f asy
Injection Volume:	10.00 ul	Channel Name:	254.0nm
Run Time:	35.0 Minutes	Proc. Chnl. Descr.:	2998 PDA 254.0 nm (2998
Date Acquired:	10/25/2022 5:36:26 PM CST		
Date Processed:	7/31/2023 8:47:48 PM CST		

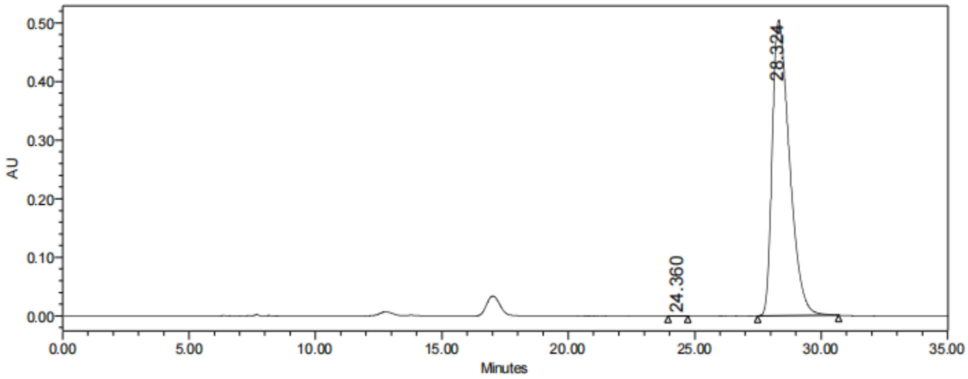

	RT	Area	% Area	Height
1	24.360	5795	0.02	222
2	28.324	23725524	99.98	503796

Rac-**3g**

SAMPLE INFORMATION

Sample Name:	cxh-10-87-3-IC-rac-1%-0.5ML	Acquired By:	System
Sample Type:	Unknown	Sample Set Name:	10252
Vial:	113	Acq. Method Set:	1% 05ML
Injection #:	1	Processing Method:	3g rac
Injection Volume:	10.00 ul	Channel Name:	280.0nm
Run Time:	35.0 Minutes	Proc. Chnl. Descr.:	2998 PDA 280.0 nm (2998
Date Acquired:	10/25/2022 7:24:24 PM CST		
Date Processed:	2/1/2024 11:28:47 AM CST		

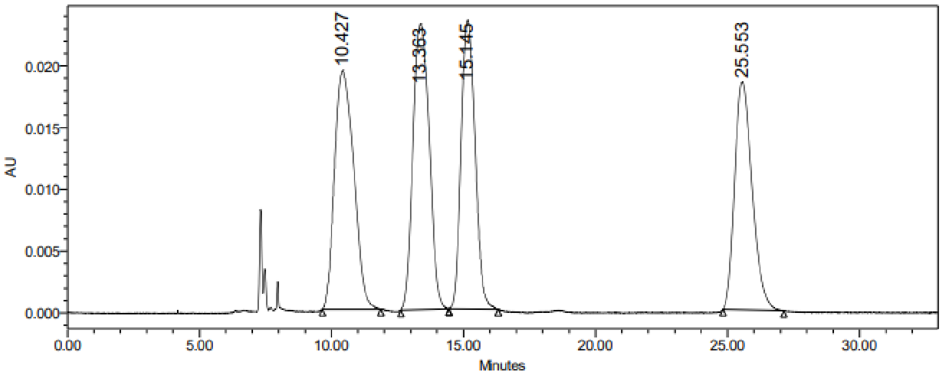

	RT	Area	% Area	Height
1	10.427	982530	26.64	19338
2	13.363	989854	26.84	23107
3	15.145	862396	23.38	23375
4	25.553	853752	23.15	18462

Asy-**3g**

SAMPLE INFORMATION

Sample Name:	cxh-10-87-3-IC-asy-1%-0.5ML	Acquired By:	System
Sample Type:	Unknown	Sample Set Name:	10251
Vial:	89	Acq. Method Set:	1% 05ML
Injection #:	1	Processing Method:	3g asy
Injection Volume:	10.00 ul	Channel Name:	280.0nm
Run Time:	35.0 Minutes	Proc. Chnl. Descr.:	2998 PDA 280.0 nm (2998
Date Acquired:	10/25/2022 6:12:27 PM CST		
Date Processed:	2/1/2024 11:31:53 AM CST		

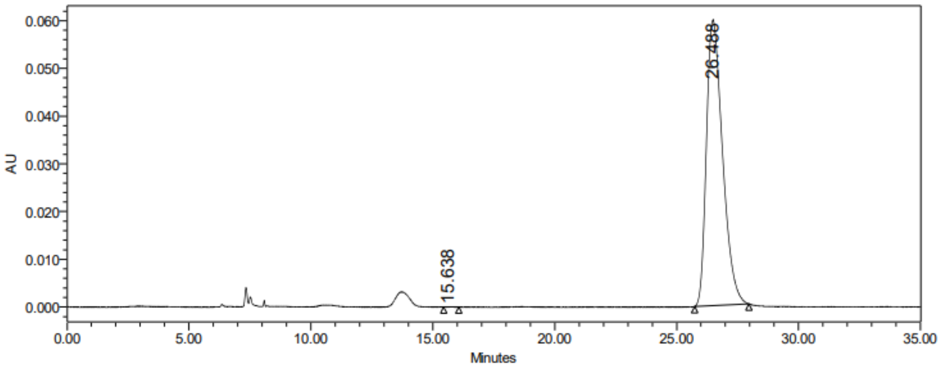

	RT	Area	% Area	Height
1	15.638	526	0.02	45
2	26.488	2746492	99.98	59827

Rac-**3h**

SAMPLE INFORMATION

Sample Name:	cxh-10-87-4-rac-IE-1%-0.5ML	Acquired By:	System
Sample Type:	Unknown	Sample Set Name:	10254
Vial:	27	Acq. Method Set:	1% 05ML
Injection #:	1	Processing Method:	3h
Injection Volume:	10.00 ul	Channel Name:	280.0nm
Run Time:	30.0 Minutes	Proc. Chnl. Descr.:	2998 PDA 280.0 nm (2998
Date Acquired:	10/25/2022 9:09:51 PM CST		
Date Processed:	2/1/2024 11:14:55 AM CST		

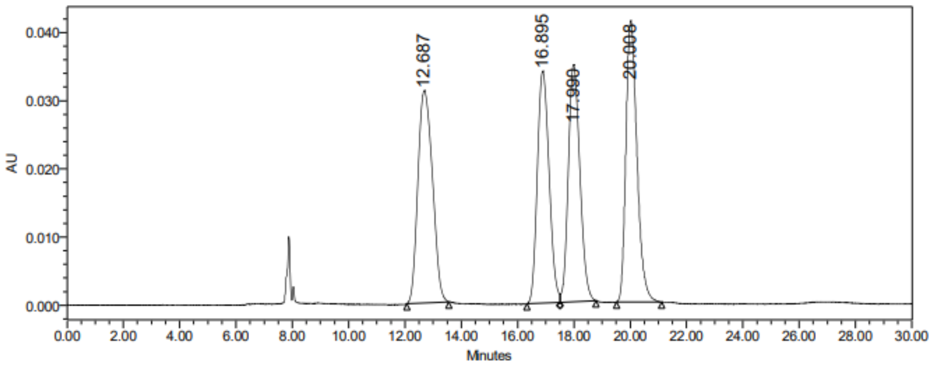

	RT	Area	% Area	Height
1	12.687	1119179	26.52	31130
2	16.895	986058	23.37	34007
3	17.990	991508	23.49	34742
4	20.008	1123467	26.62	41224

Asy-**3h**

SAMPLE INFORMATION

Sample Name:	cxh-10-87-4-asy-IE-1%-0.5ML	Acquired By:	System
Sample Type:	Unknown	Sample Set Name:	10253
Vial:	2	Acq. Method Set:	1% 05ML
Injection #:	1	Processing Method:	3h asy
Injection Volume:	10.00 ul	Channel Name:	280.0nm
Run Time:	30.0 Minutes	Proc. Chnl. Descr.:	2998 PDA 280.0 nm (2998
Date Acquired:	10/25/2022 8:12:25 PM CST		
Date Processed:	2/1/2024 11:14:11 AM CST		

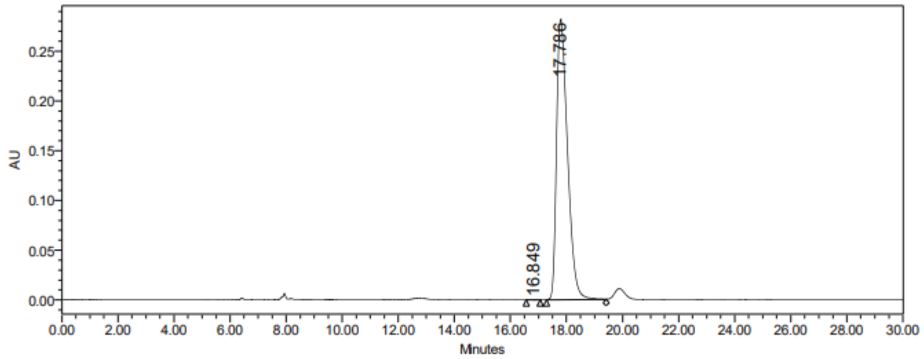

	RT	Area	% Area	Height
1	16.849	1223	0.02	80
2	17.786	7715906	99.98	281581

Rac-**3i**

SAMPLE INFORMATION

Sample Name:	cxh-10-87-5-AD-rac-1%	Acquired By:	System
Sample Type:	Unknown	Sample Set Name:	10255
Vial:	69	Acq. Method Set:	1%
Injection #:	1	Processing Method:	3i rac
Injection Volume:	10.00 ul	Channel Name:	280.0nm
Run Time:	23.0 Minutes	Proc. Chnl. Descr.:	2998 PDA 280.0 nm (2998
Date Acquired:	10/25/2022 10:37:43 PM CST		
Date Processed:	2/1/2024 11:17:31 AM CST		

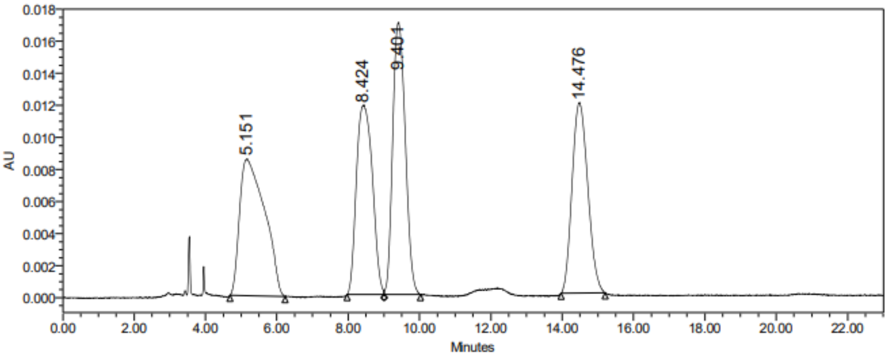

	RT	Area	% Area	Height
1	5.151	416083	26.64	8519
2	8.424	367823	23.55	11811
3	9.401	415112	26.57	16957
4	14.476	363080	23.24	11867

Asy-**3i**

SAMPLE INFORMATION

Sample Name:	cxh-10-87-5-AD-asy-1%	Acquired By:	System
Sample Type:	Unknown	Sample Set Name:	10255
Vial:	68	Acq. Method Set:	1%
Injection #:	1	Processing Method:	3i asy
Injection Volume:	10.00 ul	Channel Name:	280.0nm
Run Time:	23.0 Minutes	Proc. Chnl. Descr.:	2998 PDA 280.0 nm (2998
Date Acquired:	10/25/2022 10:14:03 PM CST		
Date Processed:	2/1/2024 11:19:55 AM CST		

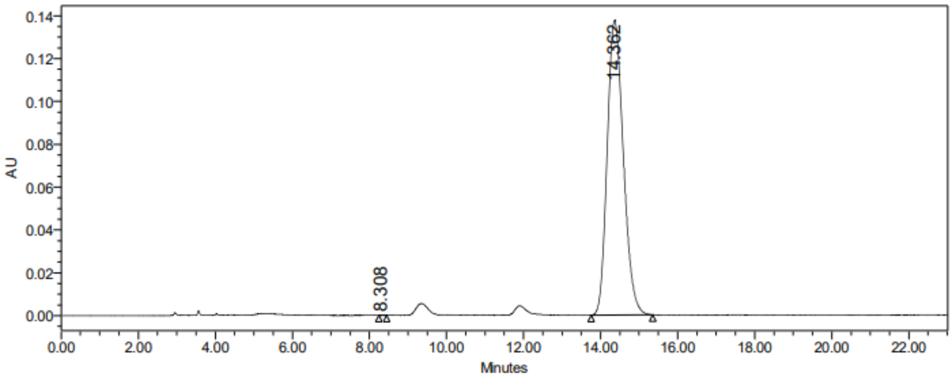

	RT	Area	% Area	Height
1	8.308	151	0.00	-39
2	14.362	4019522	100.00	137565

Rac-**3j**

SAMPLE INFORMATION

Sample Name:	cxh-10-87-6-rac-IE-1%-0.5ML	Acquired By:	System
Sample Type:	Unknown	Sample Set Name:	10254
Vial:	28	Acq. Method Set:	1% 05ML
Injection #:	1	Processing Method:	3j rac
Injection Volume:	10.00 ul	Channel Name:	280.0nm
Run Time:	25.0 Minutes	Proc. Chnl. Descr.:	2998 PDA 280.0 nm (2998
Date Acquired:	10/25/2022 9:40:32 PM CST		
Date Processed:	2/1/2024 11:35:16 AM CST		

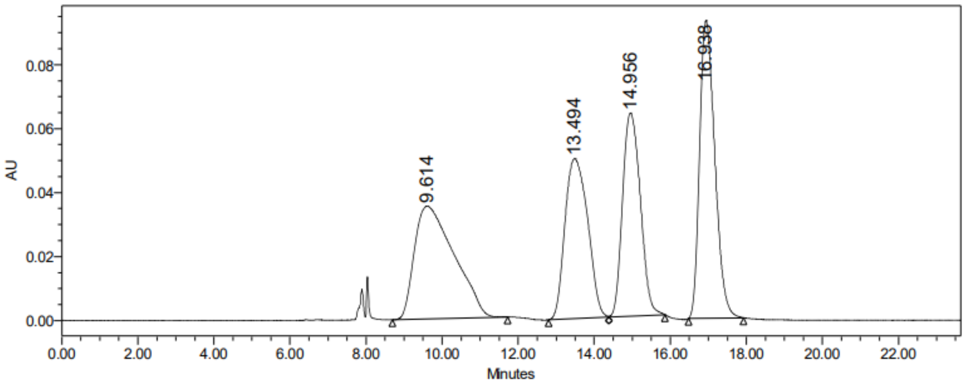

	RT	Area	% Area	Height
1	9.614	2547228	27.55	35224
2	13.494	2070441	22.40	49958
3	14.956	2062267	22.31	63543
4	16.938	2564488	27.74	93044

Asy-**3j**

SAMPLE INFORMATION

Sample Name:	cxh-10-87-6-asy-IE-1%-0.5ML	Acquired By:	System
Sample Type:	Unknown	Sample Set Name:	10253
Vial:	3	Acq. Method Set:	1% 05ML
Injection #:	1	Processing Method:	3j asy
Injection Volume:	10.00 ul	Channel Name:	280.0nm
Run Time:	30.0 Minutes	Proc. Chnl. Descr.:	2998 PDA 280.0 nm (2998
Date Acquired:	10/25/2022 8:43:08 PM CST		
Date Processed:	2/1/2024 11:36:04 AM CST		

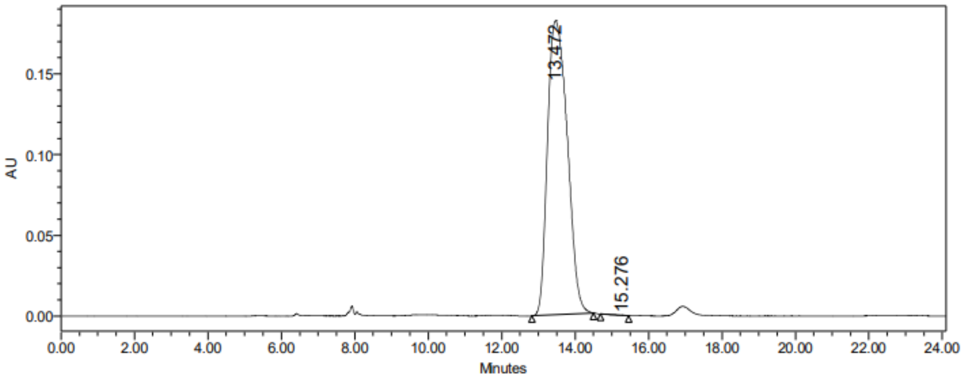

	RT	Area	% Area	Height
1	13.472	6771069	99.96	182127
2	15.276	2553	0.04	-113

Rac-**3k**

SAMPLE INFORMATION

Sample Name:	cxh-10-88-1-rac-IC-2%	Acquired By:	System
Sample Type:	Unknown	Sample Set Name:	10294
Vial:	46	Acq. Method Set:	2%
Injection #:	1	Processing Method:	881rac
Injection Volume:	10.00 ul	Channel Name:	280.0nm
Run Time:	28.0 Minutes	Proc. Chnl. Descr.:	2998 PDA 280.0 nm (2998
Date Acquired:	10/29/2022 1:55:49 PM CST		
Date Processed:	2/1/2024 10:59:34 AM CST		

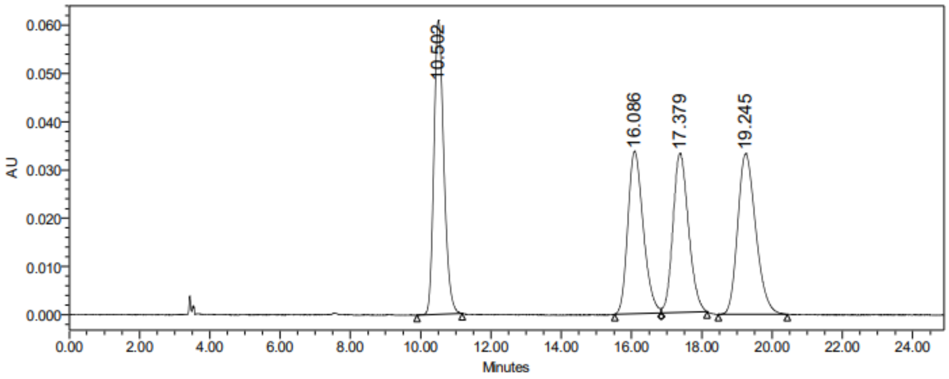

	RT	Area	% Area	Height
1	10.502	1185947	26.83	60900
2	16.086	1025684	23.20	33649
3	17.379	1020940	23.10	32995
4	19.245	1187814	26.87	33292

Asy-**3k**

SAMPLE INFORMATION

Sample Name:	cxh-10-88-1-asy-IC-2%	Acquired By:	System
Sample Type:	Unknown	Sample Set Name:	10293
Vial:	46	Acq. Method Set:	2%
Injection #:	1	Processing Method:	3k asy
Injection Volume:	10.00 ul	Channel Name:	280.0nm
Run Time:	28.0 Minutes	Proc. Chnl. Descr.:	2998 PDA 280.0 nm (2998
Date Acquired:	10/29/2022 12:43:48 PM CST		
Date Processed:	2/1/2024 11:05:36 AM CST		

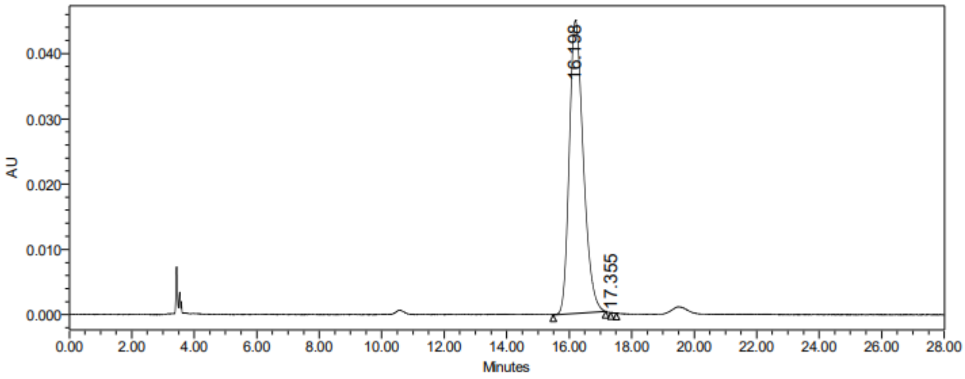

	RT	Area	% Area	Height
1	16.198	1394279	99.99	44892
2	17.355	130	0.01	37

Rac-**3l**

SAMPLE INFORMATION

Sample Name:	cxh-10-88-6-IF-rac-1%-0.5ML	Acquired By:	System
Sample Type:	Control	Sample Set Name:	
Vial:	5	Acq. Method Set:	1% 05ML
Injection #:	2	Processing Method:	3l rac
Injection Volume:	10.00 ul	Channel Name:	254.0nm
Run Time:	50.0 Minutes	Proc. Chnl. Descr.:	2998 PDA 254.0 nm (2998
Date Acquired:	10/25/2022 12:15:35 AM CST		
Date Processed:	7/31/2023 9:59:12 PM CST		

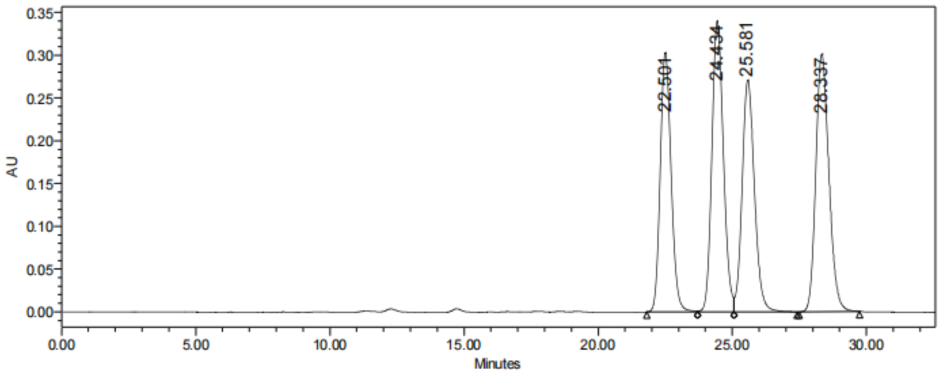

	RT	Area	% Area	Height
1	22.501	8584830	22.75	303154
2	24.434	10188334	27.00	339939
3	25.581	8717768	23.11	271041
4	28.337	10236891	27.13	301217

Asy-**3l**

SAMPLE INFORMATION

Sample Name:	cxh-10-88-6-asy-IF-1%-0.5ML	Acquired By:	System
Sample Type:	Control	Sample Set Name:	
Vial:	37	Acq. Method Set:	1% 05ML
Injection #:	1	Processing Method:	3l asy
Injection Volume:	10.00 ul	Channel Name:	254.0nm
Run Time:	50.0 Minutes	Proc. Chnl. Descr.:	2998 PDA 254.0 nm (2998
Date Acquired:	10/26/2022 7:04:54 PM CST		
Date Processed:	7/31/2023 10:01:04 PM CST		

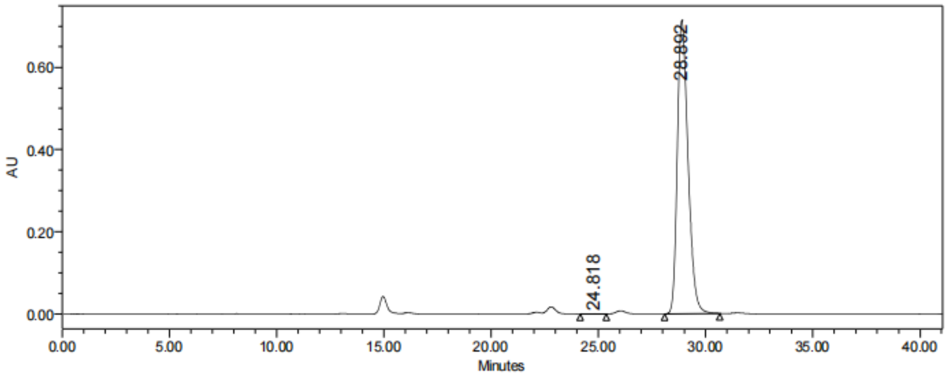

	RT	Area	% Area	Height
1	24.818	9445	0.04	332
2	28.892	24871785	99.96	713596

Rac-**3m**

SAMPLE INFORMATION

Sample Name:	cxh-10-88-3-AD-rac-1%	Acquired By:	System
Sample Type:	Control	Sample Set Name:	
Vial:	7	Acq. Method Set:	1%
Injection #:	1	Processing Method:	3m rac
Injection Volume:	10.00 ul	Channel Name:	254.0nm
Run Time:	50.0 Minutes	Proc. Chnl. Descr.:	2998 PDA 254.0 nm (2998
Date Acquired:	10/25/2022 11:29:27 AM CST		
Date Processed:	7/31/2023 9:46:11 PM CST		

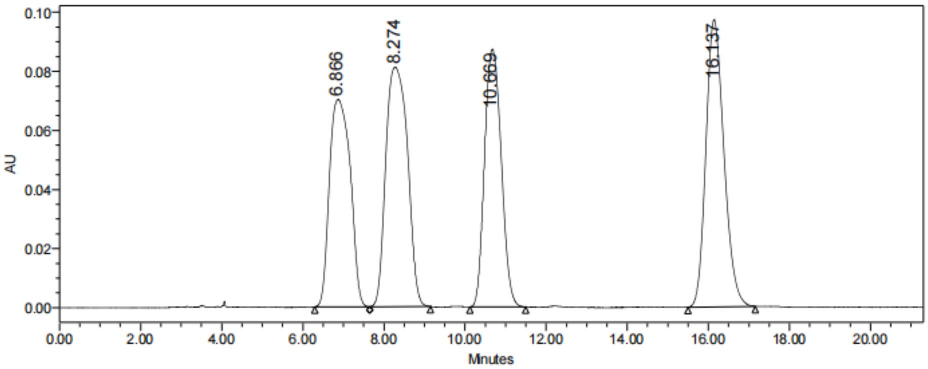

	RT	Area	% Area	Height
1	6.866	2499738	22.61	70179
2	8.274	3040655	27.50	81066
3	10.669	2493582	22.55	87107
4	16.137	3022857	27.34	97120

Asy-**3m**

SAMPLE INFORMATION

Sample Name:	cxh-10-88-3-asy-AD-1%	Acquired By:	System
Sample Type:	Control	Sample Set Name:	
Vial:	12	Acq. Method Set:	1%
Injection #:	1	Processing Method:	3m asy
Injection Volume:	10.00 ul	Channel Name:	254.0nm
Run Time:	20.0 Minutes	Proc. Chnl. Descr.:	2998 PDA 254.0 nm (2998
Date Acquired:	10/26/2022 6:30:46 PM CST		
Date Processed:	7/31/2023 9:49:02 PM CST		

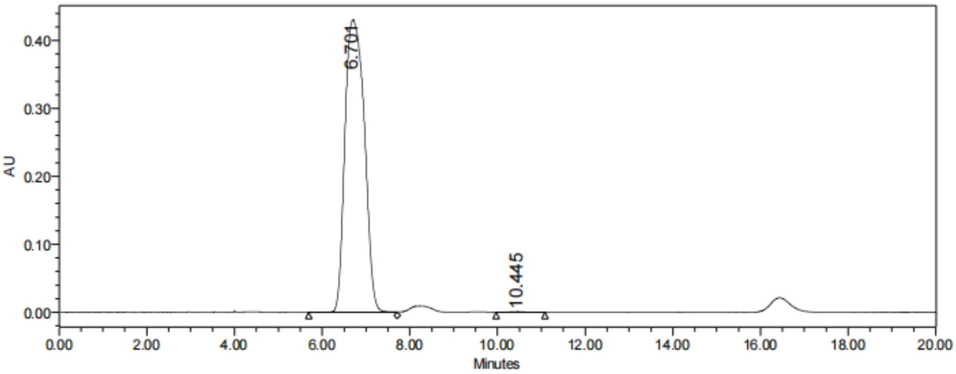

	RT	Area	% Area	Height
1	6.701	13157107	99.92	429749
2	10.445	10982	0.08	403

Rac-**3n**

SAMPLE INFORMATION

Sample Name:	cxh-12-1-1-rac-IC-1%-0.5ML	Acquired By:	System
Sample Type:	Unknown	Sample Set Name	
Vial:	94	Acq. Method Set:	1% 05ml
Injection #:	1	Processing Method	0423
Injection Volume:	10.00 ul	Channel Name:	254.0nm
Run Time:	50.0 Minutes	Proc. Chnl. Descr.:	2998 PDA 254.0 nm (2998
Date Acquired:	4/16/2024 13:35:44 CST		
Date Processed:	4/23/2024 16:08:03 CST		

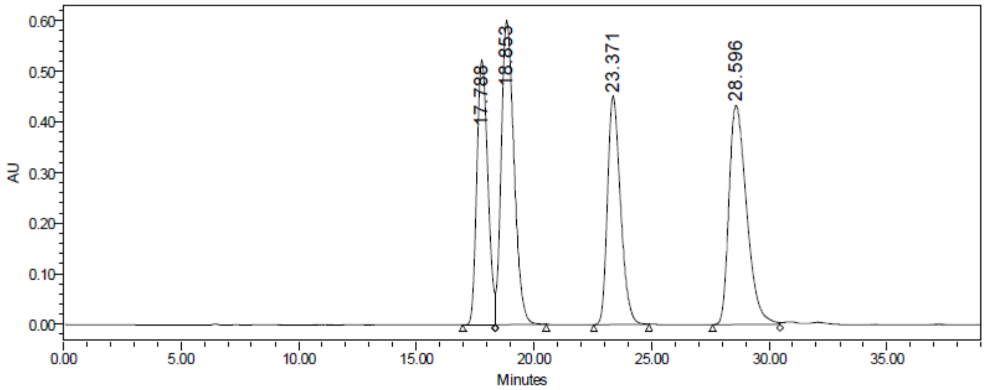

	RT	Area	% Area	Height
1	17.788	17085437	21.51	522469
2	18.853	22574838	28.42	600155
3	23.371	17345680	21.84	450863
4	28.596	22420927	28.23	433551

Asy-**3n**

SAMPLE INFORMATION

Sample Name:	cxh-12-1-1-asy-IC-1%-0.5ML	Acquired By:	System
Sample Type:	Unknown	Sample Set Name	
Vial:	115	Acq. Method Set:	1% 05ml
Injection #:	1	Processing Method	052
Injection Volume:	10.00 ul	Channel Name:	254.0nm
Run Time:	38.0 Minutes	Proc. Chnl. Descr.:	2998 PDA 254.0 nm (2998
Date Acquired:	4/16/2024 14:20:54 CST		
Date Processed:	4/23/2024 17:14:55 CST		

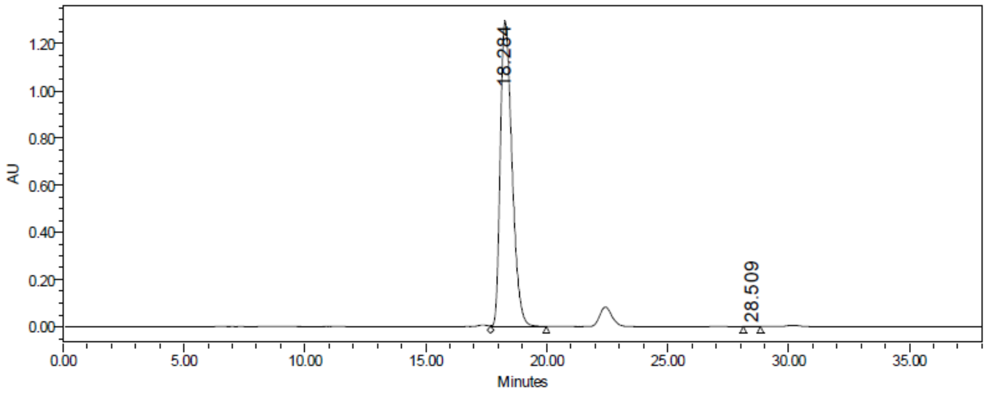

	RT	Area	% Area	Height
1	18.284	43612405	99.92	1294046
2	28.509	36744	0.08	1518

Rac-**3o**

SAMPLE INFORMATION

Sample Name:	cxh-12-1-6-rac-OJ-5%	Acquired By:	System
Sample Type:	Unknown	Sample Set Name	0417
Vial:	66	Acq. Method Set:	5%210
Injection #:	1	Processing Method	0853
Injection Volume:	10.00 ul	Channel Name:	260.0nm
Run Time:	32.0 Minutes	Proc. Chnl. Descr.:	2998 PDA 260.0 nm (2998
Date Acquired:	4/17/2024 9:16:14 CST		
Date Processed:	4/23/2024 16:12:33 CST		

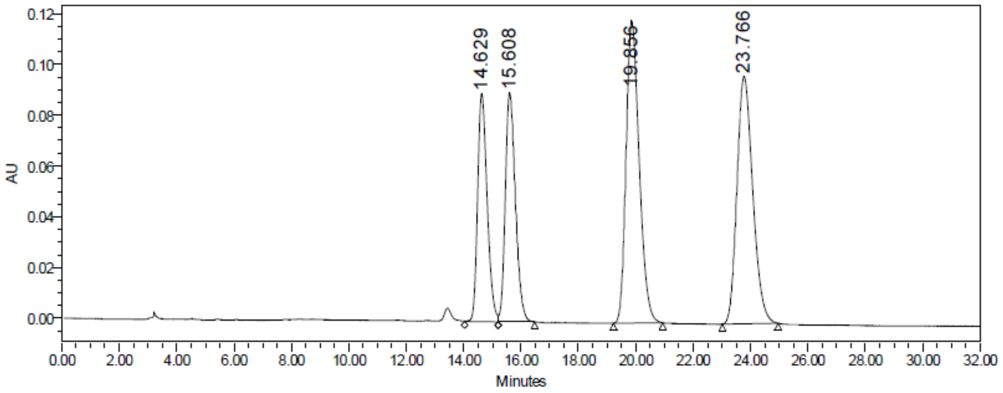

	RT	Area	% Area	Height
1	14.629	2089855	17.80	89868
2	15.608	2181461	18.59	90214
3	19.856	3751894	31.96	118879
4	23.766	3714372	31.65	97501

Asy-**3o**

SAMPLE INFORMATION

Sample Name:	cxh-12-1-6-asy-OJ-5%	Acquired By:	System
Sample Type:	Unknown	Sample Set Name	0417
Vial:	67	Acq. Method Set:	5%210
Injection #:	1	Processing Method	123
Injection Volume:	10.00 ul	Channel Name:	254.0nm
Run Time:	32.0 Minutes	Proc. Chnl. Descr.:	2998 PDA 254.0 nm (2998
Date Acquired:	4/17/2024 9:49:10 CST		
Date Processed:	4/23/2024 17:21:25 CST		

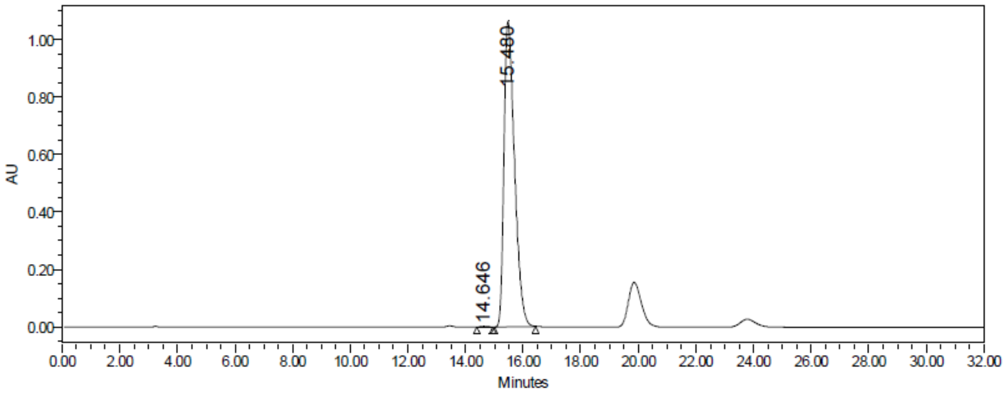

	RT	Area	% Area	Height
1	14.646	34606	0.13	1932
2	15.480	26799321	99.87	1062589

Rac-**3p**

SAMPLE INFORMATION

Sample Name:	cxh-10-90-3-rac-IC-1%-0.5ML	Acquired By:	System
Sample Type:	Control	Sample Set Name:	
Vial:	43	Acq. Method Set:	1% 05ML
Injection #:	2	Processing Method:	3n rac
Injection Volume:	10.00 ul	Channel Name:	254.0nm
Run Time:	35.0 Minutes	Proc. Chnl. Descr.:	2998 PDA 254.0 nm (2998
Date Acquired:	10/29/2022 9:25:24 AM CST		
Date Processed:	8/1/2023 8:57:41 AM CST		

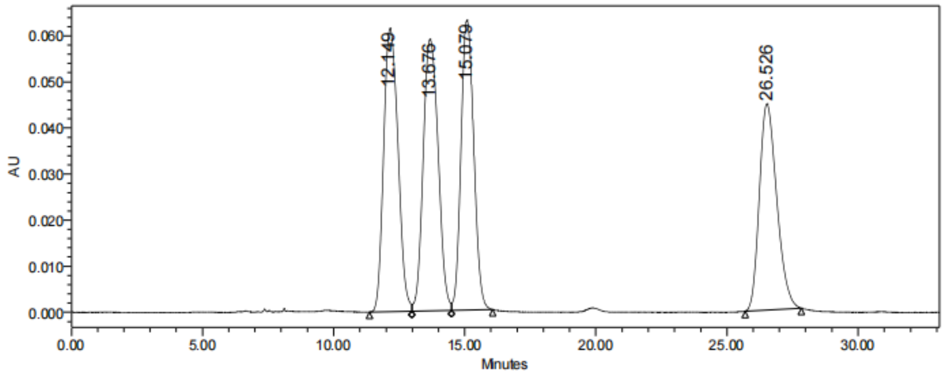

	RT	Area	% Area	Height
1	12.149	2331840	26.34	61491
2	13.676	2333088	26.35	58946
3	15.079	2131113	24.07	62905
4	26.526	2057968	23.24	44704

Asy-**3p**

SAMPLE INFORMATION

Sample Name:	cxh-10-90-3-asy-IC-1%-0.5ML	Acquired By:	System
Sample Type:	Unknown	Sample Set Name:	10306
Vial:	76	Acq. Method Set:	1% 05ML
Injection #:	1	Processing Method:	3n asy
Injection Volume:	10.00 ul	Channel Name:	254.0nm
Run Time:	35.0 Minutes	Proc. Chnl. Descr.:	2998 PDA 254.0 nm (2998
Date Acquired:	10/31/2022 12:06:48 AM CST		
Date Processed:	8/1/2023 8:58:58 AM CST		

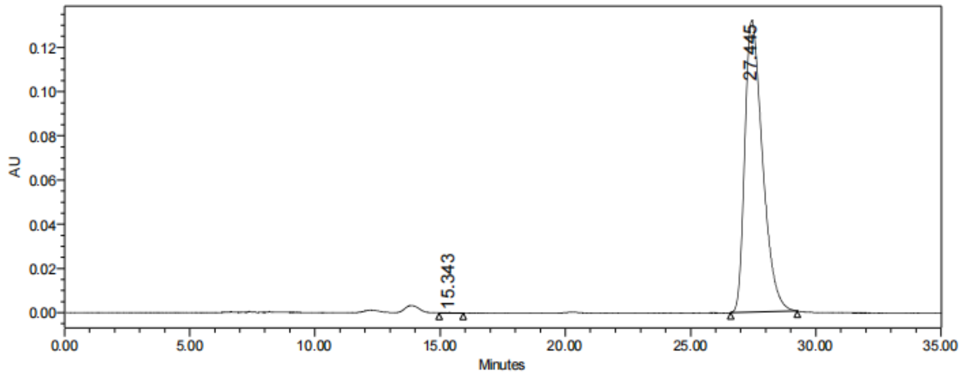

	RT	Area	% Area	Height
1	15.343	953	0.01	44
2	27.445	6532558	99.99	131959

Rac-**3q**

SAMPLE INFORMATION

Sample Name:	cxh-10-90-4-rac-IG-1%-0.5ML	Acquired By:	System
Sample Type:	Control	Sample Set Name:	
Vial:	48	Acq. Method Set:	1% 05ML
Injection #:	1	Processing Method:	3o rac
Injection Volume:	10.00 ul	Channel Name:	254.0nm
Run Time:	60.0 Minutes	Proc. Chnl. Descr.:	2998 PDA 254.0 nm (2998
Date Acquired:	10/29/2022 4:35:40 PM CST		
Date Processed:	8/1/2023 9:09:37 AM CST		

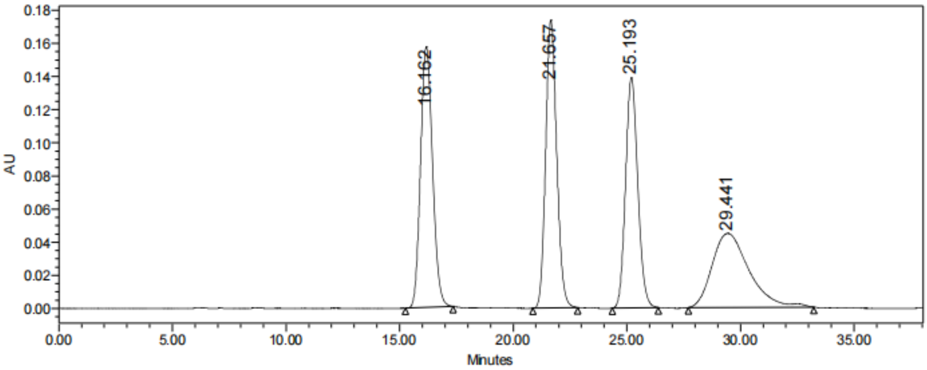

	RT	Area	% Area	Height
1	16.162	5744819	26.39	157374
2	21.657	5811026	26.69	173680
3	25.193	5158274	23.70	139006
4	29.441	5055216	23.22	44734

Asy-**3q**

SAMPLE INFORMATION

Sample Name:	cxh-10-90-4-asy-IG-1%-0.5ML	Acquired By:	System
Sample Type:	Unknown	Sample Set Name:	10303
Vial:	33	Acq. Method Set:	1% 05ML
Injection #:	1	Processing Method:	3o asy
Injection Volume:	10.00 ul	Channel Name:	254.0nm
Run Time:	40.0 Minutes	Proc. Chnl. Descr.:	2998 PDA 254.0 nm (2998
Date Acquired:	10/30/2022 7:06:02 PM CST		
Date Processed:	8/1/2023 9:11:26 AM CST		

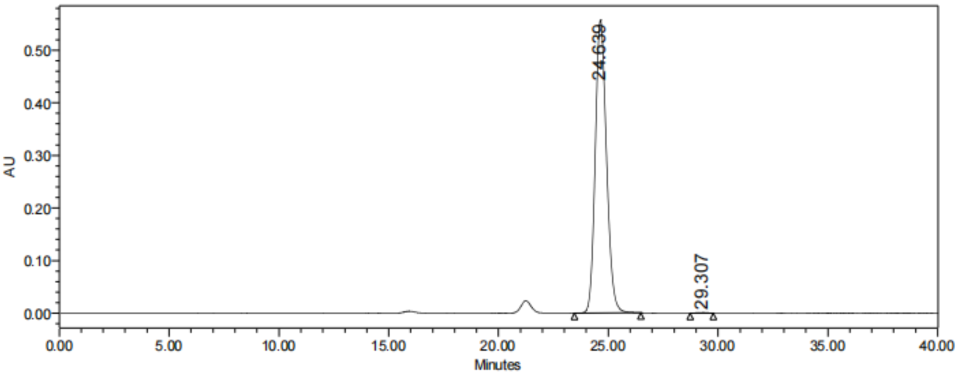

	RT	Area	% Area	Height
1	24.639	19930331	99.89	556768
2	29.307	21472	0.11	653

Rac-**3r**

SAMPLE INFORMATION

Sample Name:	cxh-10-90-5-rac-IG-1%-0.5ML	Acquired By:	System
Sample Type:	Unknown	Sample Set Name:	10305
Vial:	61	Acq. Method Set:	1% 05ML
Injection #:	1	Processing Method:	3p rac
Injection Volume:	10.00 ul	Channel Name:	254.0nm
Run Time:	40.0 Minutes	Proc. Chnl. Descr.:	2998 PDA 254.0 nm (2998
Date Acquired:	10/30/2022 9:46:42 PM CST		
Date Processed:	8/1/2023 9:13:23 AM CST		

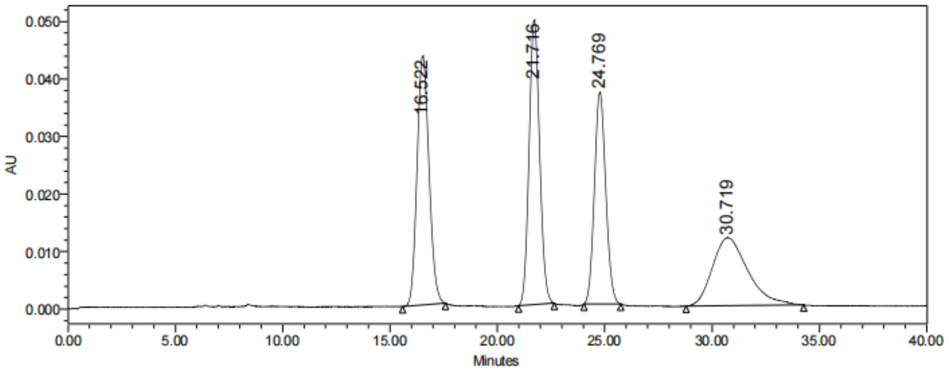

	RT	Area	% Area	Height
1	16.522	1670225	27.57	43258
2	21.716	1682342	27.77	49460
3	24.769	1347780	22.25	36840
4	30.719	1357072	22.40	11836

Asy-**3r**

SAMPLE INFORMATION

Sample Name:	cxh-10-90-5-asy-IG-1%-0.5ML	Acquired By:	System
Sample Type:	Unknown	Sample Set Name:	10303
Vial:	34	Acq. Method Set:	1% 05ML
Injection #:	1	Processing Method:	3p asy
Injection Volume:	10.00 ul	Channel Name:	254.0nm
Run Time:	40.0 Minutes	Proc. Chnl. Descr.:	2998 PDA 254.0 nm (2998
Date Acquired:	10/30/2022 7:46:43 PM CST		
Date Processed:	8/1/2023 9:15:43 AM CST		

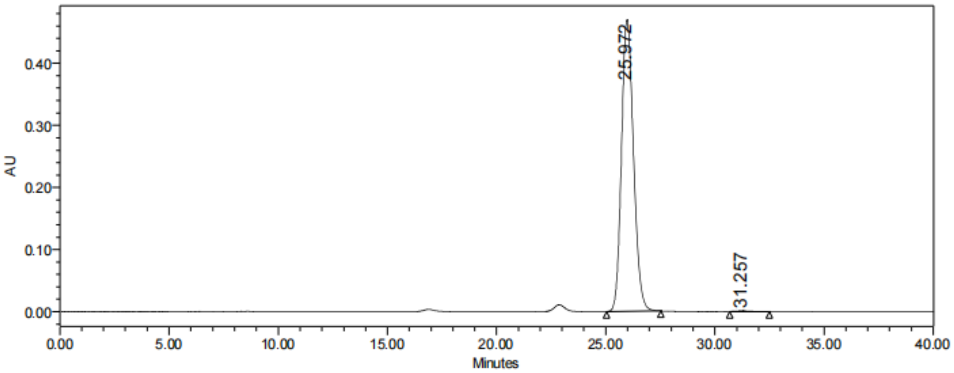

	RT	Area	% Area	Height
1	25.972	17684463	99.80	468717
2	31.257	36068	0.20	916

Rac-**3s**

SAMPLE INFORMATION

Sample Name:	cxh-10-90-1-rac-IE-1%-0.5ML	Acquired By:	System
Sample Type:	Control	Sample Set Name:	
Vial:	45	Acq. Method Set:	1% 05ML
Injection #:	1	Processing Method:	3q rac
Injection Volume:	10.00 ul	Channel Name:	254.0nm
Run Time:	50.0 Minutes	Proc. Chnl. Descr.:	2998 PDA 254.0 nm (2998
Date Acquired:	10/28/2022 11:14:57 PM CST		
Date Processed:	8/1/2023 9:01:23 AM CST		

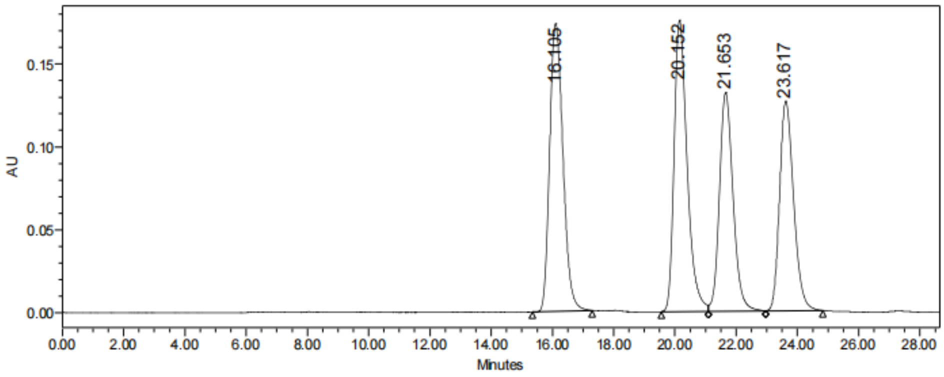

	RT	Area	% Area	Height
1	16.105	5204717	28.14	173661
2	20.152	5184742	28.03	175659
3	21.653	4098625	22.16	131918
4	23.617	4010700	21.68	126324

Asy-**3s**

SAMPLE INFORMATION

Sample Name:	cxh-10-90-1-asy-IE-1%-0.5ML	Acquired By:	System
Sample Type:	Control	Sample Set Name:	
Vial:	31	Acq. Method Set:	1% 05ML
Injection #:	1	Processing Method:	3q asy
Injection Volume:	10.00 ul	Channel Name:	254.0nm
Run Time:	60.0 Minutes	Proc. Chnl. Descr.:	2998 PDA 254.0 nm (2998
Date Acquired:	10/30/2022 5:46:06 PM CST		
Date Processed:	8/1/2023 9:02:46 AM CST		

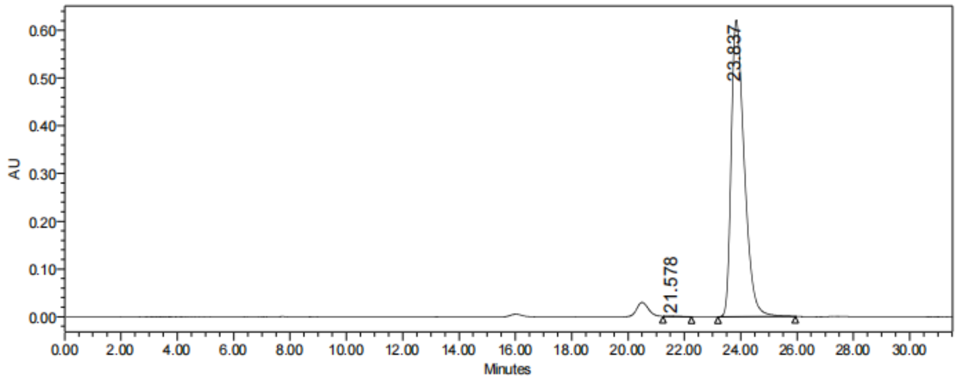

	RT	Area	% Area	Height
1	21.578	5805	0.03	282
2	23.837	20065041	99.97	620423

Rac-**3t**

SAMPLE INFORMATION

Sample Name:	cxh-10-92-2-rac-IC-2%	Acquired By:	System
Sample Type:	Control	Sample Set Name:	
Vial:	119	Acq. Method Set:	2%
Injection #:	1	Processing Method:	3r rac
Injection Volume:	10.00 ul	Channel Name:	254.0nm
Run Time:	60.0 Minutes	Proc. Chnl. Descr.:	2998 PDA 254.0 nm (2998
Date Acquired:	11/2/2022 8:19:43 AM CST		
Date Processed:	8/1/2023 9:47:46 AM CST		

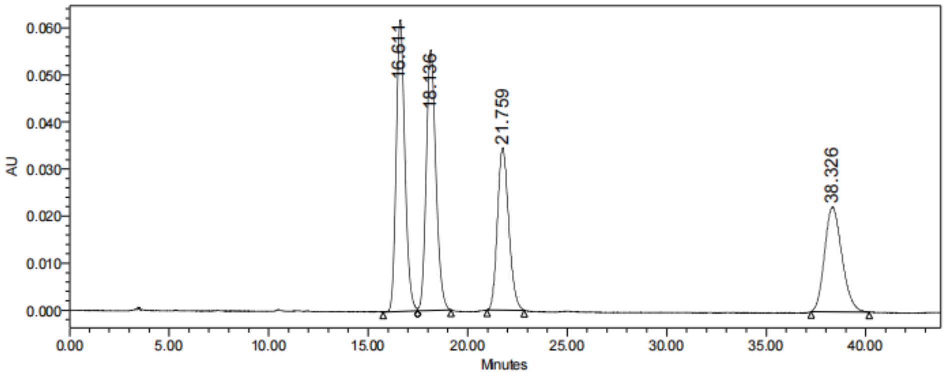

	RT	Area	% Area	Height
1	16.611	1870620	28.99	61879
2	18.136	1863696	28.89	55364
3	21.759	1360748	21.09	34437
4	38.326	1356539	21.03	22246

Asy-**3t**

SAMPLE INFORMATION

Sample Name:	cxh-10-92-2-asy-IC-2%	Acquired By:	System
Sample Type:	Unknown	Sample Set Name:	110103
Vial:	82	Acq. Method Set:	2%
Injection #:	1	Processing Method:	3r asy
Injection Volume:	10.00 ul	Channel Name:	254.0nm
Run Time:	45.0 Minutes	Proc. Chnl. Descr.:	2998 PDA 254.0 nm (2998
Date Acquired:	11/2/2022 1:51:30 AM CST		
Date Processed:	8/1/2023 9:49:10 AM CST		

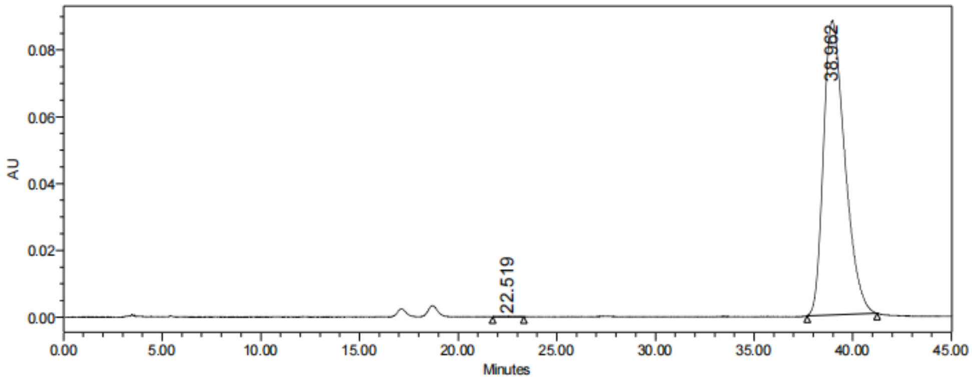

	RT	Area	% Area	Height
1	22.519	1813	0.03	67
2	38.962	6644080	99.97	88044

Rac-**3u**

SAMPLE INFORMATION

Sample Name:	cxh-10-90-2-rac-IC-1%	Acquired By:	System
Sample Type:	Unknown	Sample Set Name:	10306
Vial:	81	Acq. Method Set:	1%
Injection #:	1	Processing Method:	3s rac
Injection Volume:	10.00 ul	Channel Name:	254.0nm
Run Time:	35.0 Minutes	Proc. Chnl. Descr.:	2998 PDA 254.0 nm (2998
Date Acquired:	10/31/2022 3:20:44 AM CST		
Date Processed:	8/1/2023 9:05:27 AM CST		

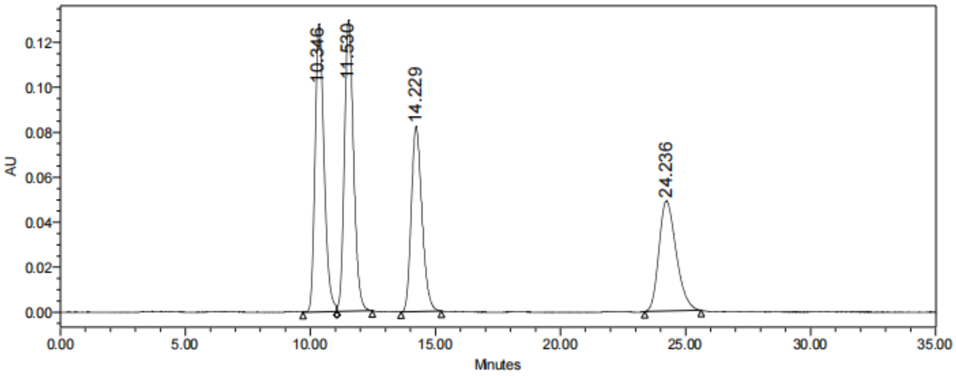

	RT	Area	% Area	Height
1	10.346	3221202	28.54	128001
2	11.530	3276353	29.03	129442
3	14.229	2421907	21.46	82252
4	24.236	2367780	20.98	49033

Asy-**3u**

SAMPLE INFORMATION

Sample Name:	cxh-10-90-2-asy-IC-1%	Acquired By:	System
Sample Type:	Unknown	Sample Set Name:	10306
Vial:	78	Acq. Method Set:	1%
Injection #:	1	Processing Method:	3s asy
Injection Volume:	10.00 ul	Channel Name:	254.0nm
Run Time:	35.0 Minutes	Proc. Chnl. Descr.:	2998 PDA 254.0 nm (2998
Date Acquired:	10/31/2022 1:23:24 AM CST		
Date Processed:	8/1/2023 9:06:28 AM CST		

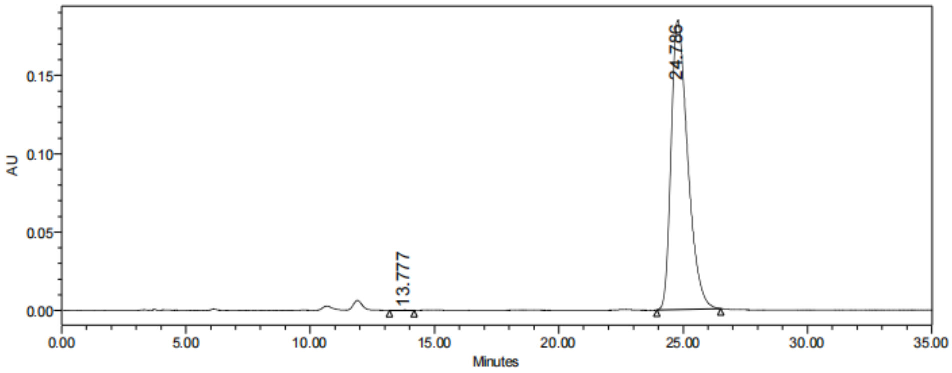

	RT	Area	% Area	Height
1	13.777	615	0.01	39
2	24.786	8579691	99.99	184538

Rac-**3v**

SAMPLE INFORMATION

Sample Name:	cxh-10-90-6-rac-IG-1%-0.5ML	Acquired By:	System
Sample Type:	Control	Sample Set Name:	
Vial:	62	Acq. Method Set:	1% 05ML
Injection #:	2	Processing Method:	3u rac
Injection Volume:	10.00 ul	Channel Name:	254.0nm
Run Time:	60.0 Minutes	Proc. Chnl. Descr.:	2998 PDA 254.0 nm (2998
Date Acquired:	10/30/2022 11:14:22 PM CST		
Date Processed:	8/1/2023 9:17:53 AM CST		

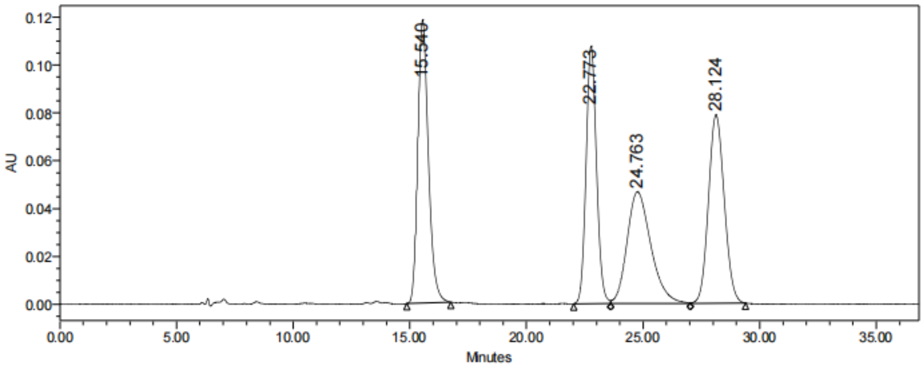

	RT	Area	% Area	Height
1	15.540	3681659	26.14	118291
2	22.773	3355659	23.82	107612
3	24.763	3322054	23.59	46626
4	28.124	3725705	26.45	78722

Asy-**3v**

SAMPLE INFORMATION

Sample Name:	cxh-10-90-6-asy-IG-1%-0.5ML	Acquired By:	System
Sample Type:	Unknown	Sample Set Name:	10303
Vial:	35	Acq. Method Set:	1% 05ML
Injection #:	1	Processing Method:	3u asy
Injection Volume:	10.00 ul	Channel Name:	254.0nm
Run Time:	60.0 Minutes	Proc. Chnl. Descr.:	2998 PDA 254.0 nm (2998
Date Acquired:	10/30/2022 8:27:25 PM CST		
Date Processed:	8/1/2023 9:25:24 AM CST		

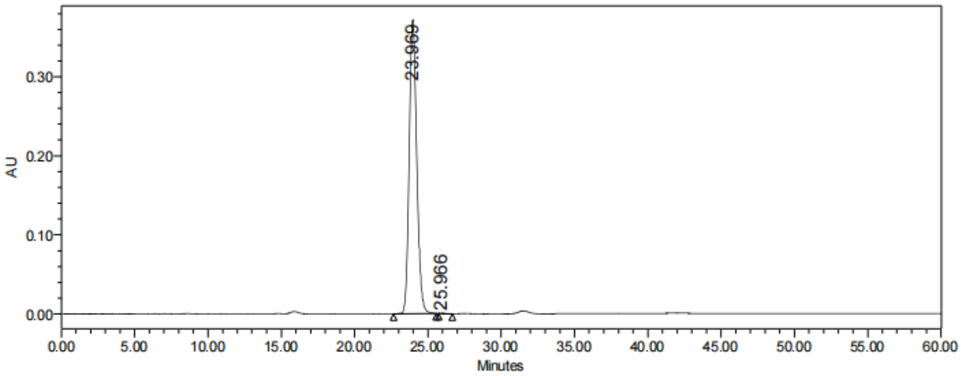

	RT	Area	% Area	Height
1	23.969	13278745	99.98	370873
2	25.966	2091	0.02	-77

Rac-**3w**

SAMPLE INFORMATION

Sample Name:	cxh-10-91-2-rac-IE-1%-0.5ML	Acquired By:	System
Sample Type:	Unknown	Sample Set Name:	10291
Vial:	47	Acq. Method Set:	1% 05ML
Injection #:	1	Processing Method:	3t rac
Injection Volume:	10.00 ul	Channel Name:	254.0nm
Run Time:	30.0 Minutes	Proc. Chnl. Descr.:	2998 PDA 254.0 nm (2998
Date Acquired:	10/29/2022 2:09:33 AM CST		
Date Processed:	8/1/2023 9:36:01 AM CST		

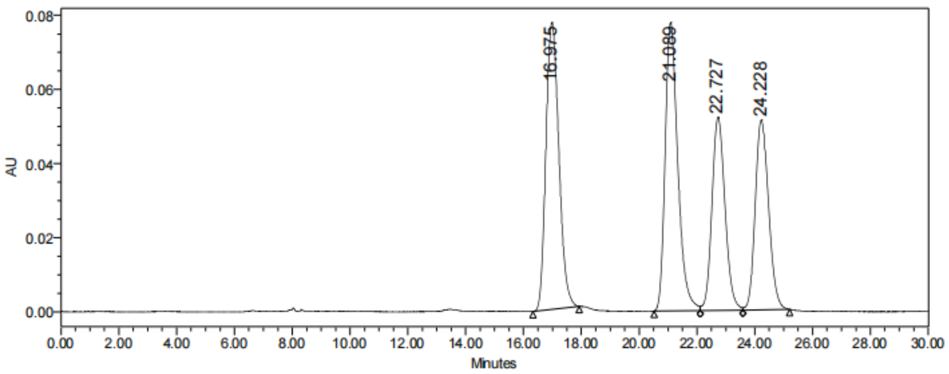

	RT	Area	% Area	Height
1	16.975	2387738	29.73	77369
2	21.089	2369349	29.50	77820
3	22.727	1648093	20.52	52121
4	24.228	1625195	20.24	51246

Asy-**3w**

SAMPLE INFORMATION

Sample Name:	cxh-10-91-2-asy-IE-1%-0.5ML	Acquired By:	System
Sample Type:	Control	Sample Set Name:	
Vial:	32	Acq. Method Set:	1% 05ML
Injection #:	1	Processing Method:	3t asy
Injection Volume:	10.00 ul	Channel Name:	254.0nm
Run Time:	40.0 Minutes	Proc. Chnl. Descr.:	2998 PDA 254.0 nm (2998
Date Acquired:	10/30/2022 6:19:27 PM CST		
Date Processed:	8/1/2023 9:37:18 AM CST		

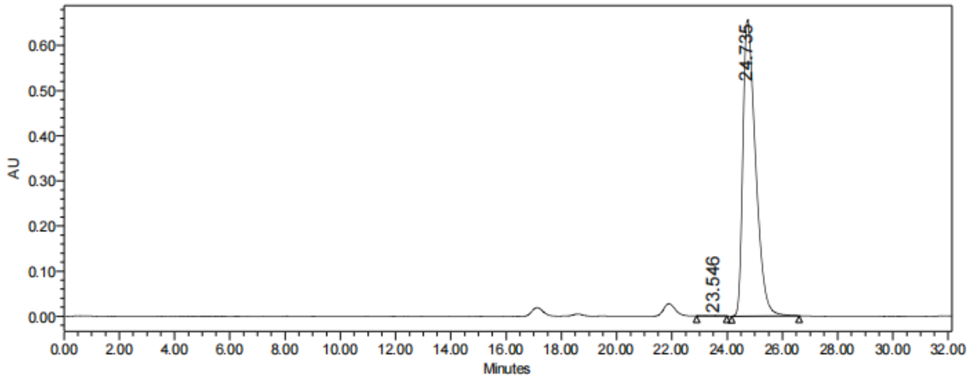

	RT	Area	% Area	Height
1	23.546	25810	0.12	665
2	24.735	21032844	99.88	655644

Rac-**3x**

SAMPLE INFORMATION

Sample Name:	cxh-10-91-1-rac-IC-1%-0.5ML	Acquired By:	System
Sample Type:	Unknown	Sample Set Name:	1031
Vial:	92	Acq. Method Set:	1% 05ML
Injection #:	1	Processing Method:	3v rac
Injection Volume:	10.00 ul	Channel Name:	254.0nm
Run Time:	35.0 Minutes	Proc. Chnl. Descr.:	2998 PDA 254.0 nm (2998
Date Acquired:	10/31/2022 9:00:20 AM CST		
Date Processed:	8/1/2023 9:30:56 AM CST		

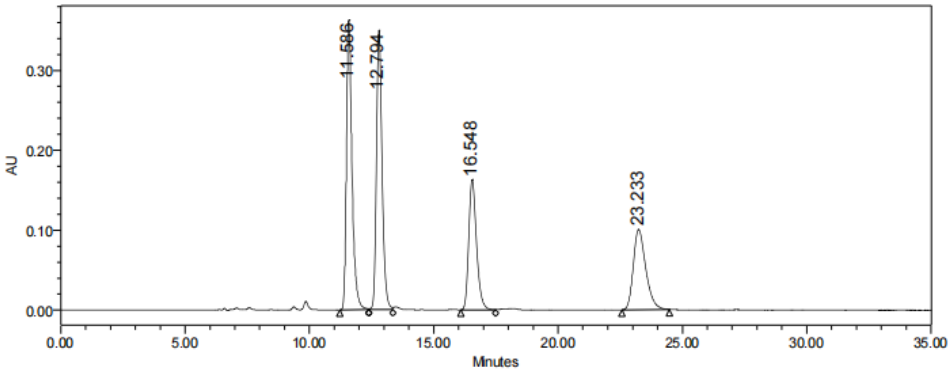

	RT	Area	% Area	Height
1	11.586	5605144	30.79	362663
2	12.794	5533715	30.40	349820
3	16.548	3568287	19.60	163061
4	23.233	3495072	19.20	100607

Asy-**3x**

SAMPLE INFORMATION

Sample Name:	cxh-10-91-1-asy-IC-1%-0.5ML	Acquired By:	System
Sample Type:	Unknown	Sample Set Name:	10312
Vial:	99	Acq. Method Set:	1% 05ML
Injection #:	1	Processing Method:	3v asy
Injection Volume:	10.00 ul	Channel Name:	254.0nm
Run Time:	35.0 Minutes	Proc. Chnl. Descr.:	2998 PDA 254.0 nm (2998
Date Acquired:	10/31/2022 10:21:25 AM CST		
Date Processed:	8/1/2023 9:33:46 AM CST		

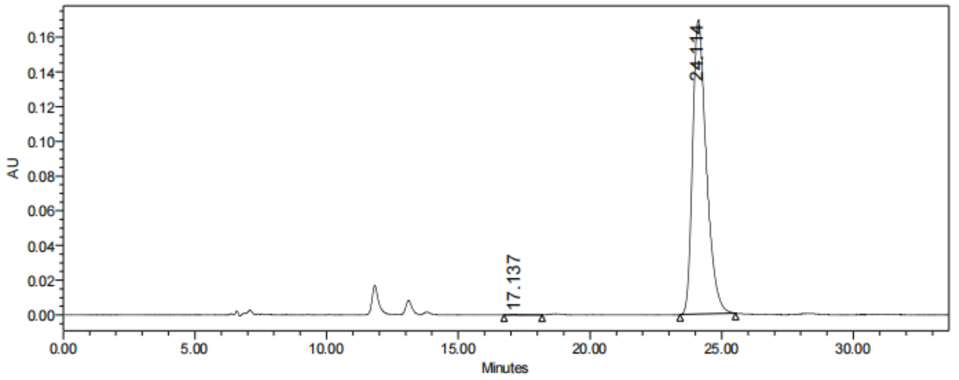

	RT	Area	% Area	Height
1	17.137	3758	0.06	171
2	24.114	6111010	99.94	169247

Rac-**3y**

SAMPLE INFORMATION

Sample Name:	cxh-10-92-1-rac-Ic-1%-0.5ML	Acquired By:	System
Sample Type:	Control	Sample Set Name:	
Vial:	4	Acq. Method Set:	1% 05ML
Injection #:	1	Processing Method:	3w rac
Injection Volume:	10.00 ul	Channel Name:	254.0nm
Run Time:	60.0 Minutes	Proc. Chnl. Descr.:	2998 PDA 254.0 nm (2998
Date Acquired:	11/1/2022 11:49:17 AM CST		
Date Processed:	8/1/2023 9:46:34 AM CST		

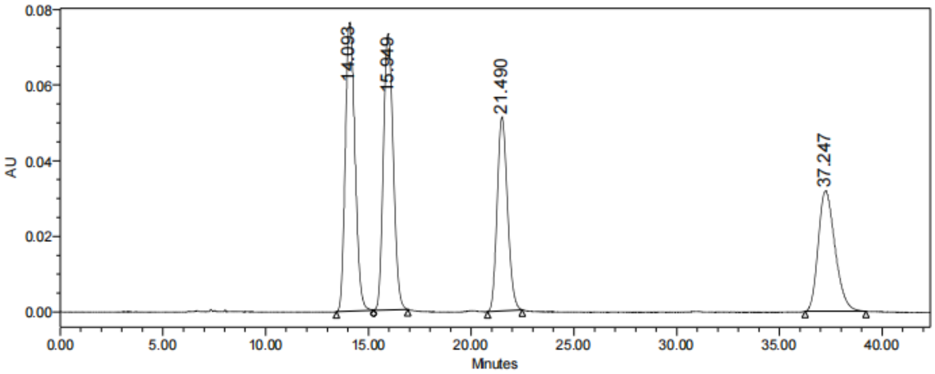

	RT	Area	% Area	Height
1	14.093	2472296	29.01	76297
2	15.949	2450291	28.75	73014
3	21.490	1800195	21.12	51184
4	37.247	1799156	21.11	31809

Asy-**3y**

SAMPLE INFORMATION

Sample Name:	cxh-10-92-1-asy-IC-1%-0.5ML	Acquired By:	System
Sample Type:	Unknown	Sample Set Name:	110103
Vial:	81	Acq. Method Set:	1% 05ML
Injection #:	1	Processing Method:	3w asy
Injection Volume:	10.00 ul	Channel Name:	254.0nm
Run Time:	45.0 Minutes	Proc. Chnl. Descr.:	2998 PDA 254.0 nm (2998
Date Acquired:	11/2/2022 1:00:37 AM CST		
Date Processed:	8/1/2023 9:45:23 AM CST		

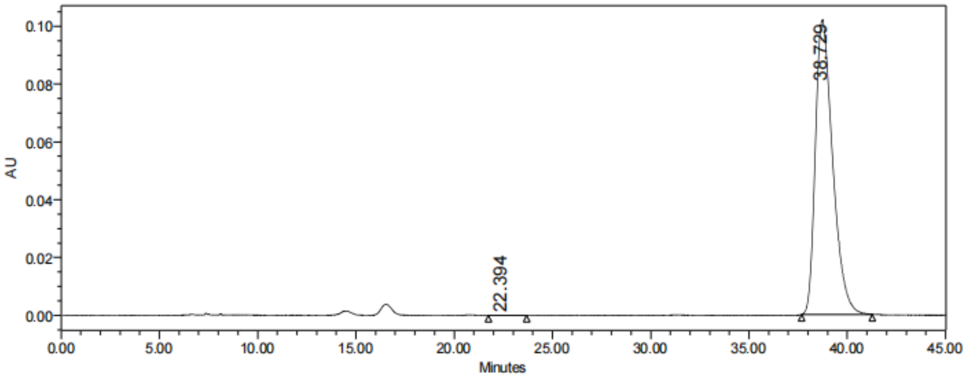

	RT	Area	% Area	Height
1	22.394	1776	0.03	52
2	38.729	6279533	99.97	101844

Rac-**3z**

SAMPLE INFORMATION

Sample Name:	cxh-10-94-1-rac-IC-1%-0.5ML	Acquired By:	System
Sample Type:	Control	Sample Set Name:	
Vial:	18	Acq. Method Set:	1% 05ML
Injection #:	1	Processing Method:	3x rac
Injection Volume:	10.00 ul	Channel Name:	254.0nm
Run Time:	40.0 Minutes	Proc. Chnl. Descr.:	2998 PDA 254.0 nm (2998
Date Acquired:	11/11/2022 8:53:44 AM CST		
Date Processed:	8/1/2023 10:05:56 AM CST		

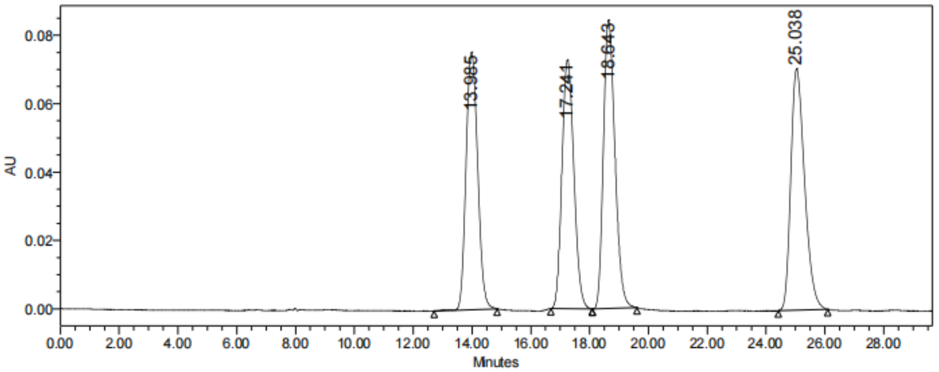

	RT	Area	% Area	Height
1	13.985	2077515	23.76	75270
2	17.241	2049248	23.44	72791
3	18.643	2303909	26.35	84343
4	25.038	2311740	26.44	70602

Asy-**3z**

SAMPLE INFORMATION

Sample Name:	cxh-10-94-1-asy-IC-1%-0.5ML	Acquired By:	System
Sample Type:	Control	Sample Set Name:	
Vial:	19	Acq. Method Set:	1% 05ML
Injection #:	1	Processing Method:	3x asy
Injection Volume:	10.00 ul	Channel Name:	254.0nm
Run Time:	35.0 Minutes	Proc. Chnl. Descr.:	2998 PDA 254.0 nm (2998
Date Acquired:	11/11/2022 9:24:38 AM CST		
Date Processed:	8/1/2023 10:05:47 AM CST		

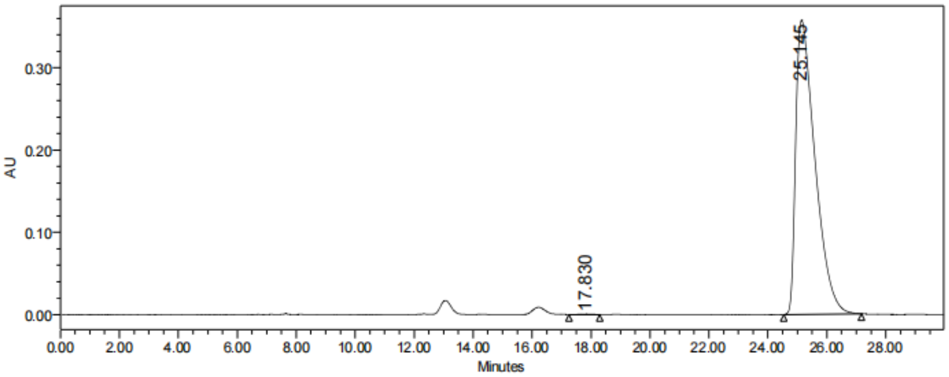

	RT	Area	% Area	Height
1	17.830	11358	0.07	415
2	25.145	16312408	99.93	357090

Rac-**3aa**

SAMPLE INFORMATION

Sample Name:	cxh-10-93-5-rac-IC-1%	Acquired By:	System
Sample Type:	Unknown	Sample Set Name:	0711
Vial:	86	Acq. Method Set:	1%
Injection #:	1	Processing Method:	3y rac
Injection Volume:	10.00 ul	Channel Name:	254.0nm
Run Time:	17.0 Minutes	Proc. Chnl. Descr.:	2998 PDA 254.0 nm (2998
Date Acquired:	7/10/2023 10:46:42 PM CST		
Date Processed:	8/1/2023 11:26:03 AM CST		

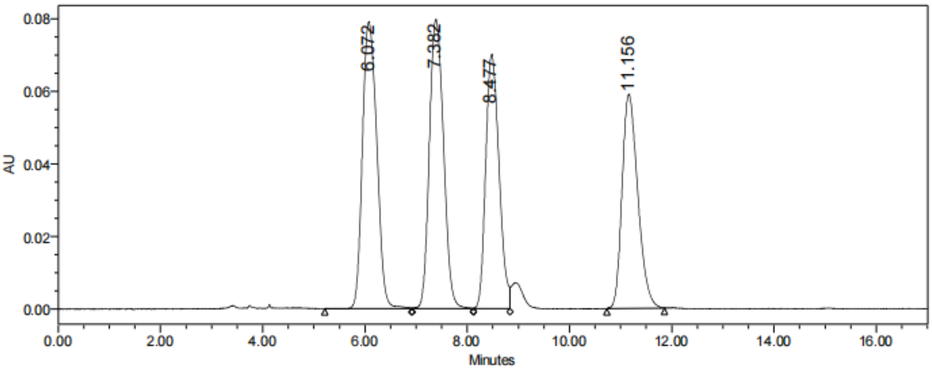

	RT	Area	% Area	Height
1	6.072	1547984	27.63	79008
2	7.382	1536855	27.43	79564
3	8.477	1261701	22.52	69952
4	11.156	1255455	22.41	58918

(*S*,*R*)-**3aa**

SAMPLE INFORMATION

Sample Name:	cxh-10-93-5-asy-IC-1%	Acquired By:	System
Sample Type:	Unknown	Sample Set Name:	0711
Vial:	87	Acq. Method Set:	1%
Injection #:	1	Processing Method:	SR 3y
Injection Volume:	10.00 ul	Channel Name:	254.0nm
Run Time:	17.0 Minutes	Proc. Chnl. Descr.:	2998 PDA 254.0 nm (2998
Date Acquired:	7/10/2023 11:04:25 PM CST		
Date Processed:	8/1/2023 11:27:59 AM CST		

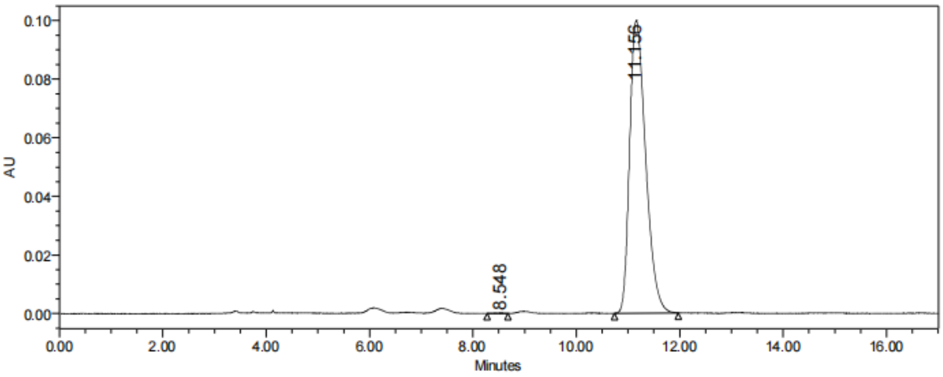

	RT	Area	% Area	Height
1	8.548	755	0.04	70
2	11.156	2136184	99.96	99891

Rac-**3aa**

SAMPLE INFORMATION			
Sample Name:	cxh-10-93-5-rac-IC-1%	Acquired By:	System
Sample Type:	Unknown	Sample Set Name:	0711
Vial:	86	Acq. Method Set:	1%
Injection #:	1	Processing Method:	3y rac
Injection Volume:	10.00 ul	Channel Name:	254.0nm
Run Time:	17.0 Minutes	Proc. Chnl. Descr.:	2998 PDA 254.0 nm (2998
Date Acquired:	7/10/2023 10:46:42 PM CST		
Date Processed:	8/1/2023 11:26:03 AM CST		

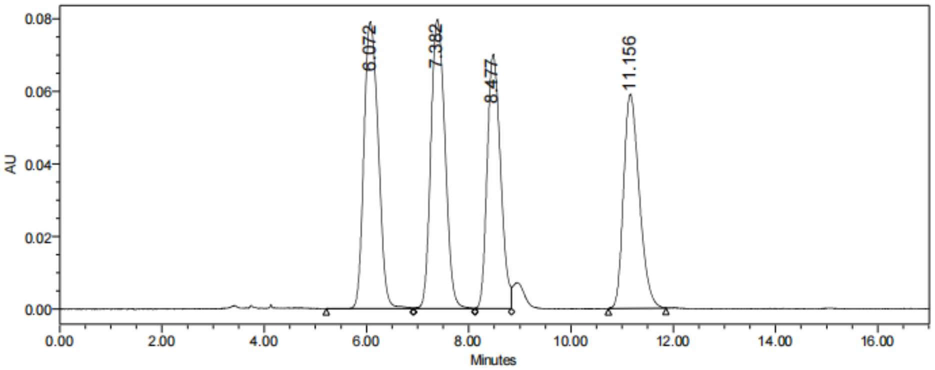

	RT	Area	% Area	Height
1	6.072	1547984	27.63	79008
2	7.382	1536855	27.43	79564
3	8.477	1261701	22.52	69952
4	11.156	1255455	22.41	58918

(*R*,*S*)-**3aa**

SAMPLE INFORMATION			
Sample Name:	cxh-10-96-5-asy-IC-1%	Acquired By:	System
Sample Type:	Unknown	Sample Set Name:	0711
Vial:	88	Acq. Method Set:	1%
Injection #:	1	Processing Method:	RS 3y
Injection Volume:	10.00 ul	Channel Name:	254.0nm
Run Time:	17.0 Minutes	Proc. Chnl. Descr.:	2998 PDA 254.0 nm (2998
Date Acquired:	7/10/2023 11:22:08 PM CST		
Date Processed:	8/1/2023 11:29:44 AM CST		

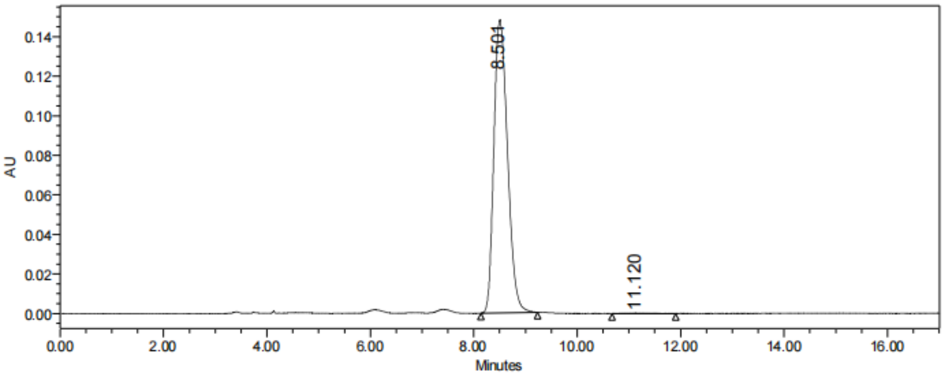

	RT	Area	% Area	Height
1	8.501	2697919	99.81	147991
2	11.120	5032	0.19	142

Rac-**3aa**

SAMPLE INFORMATION			
Sample Name:	cxh-10-93-5-rac-IC-1%	Acquired By:	System
Sample Type:	Unknown	Sample Set Name:	0711
Vial:	86	Acq. Method Set:	1%
Injection #:	1	Processing Method:	3y rac
Injection Volume:	10.00 ul	Channel Name:	254.0nm
Run Time:	17.0 Minutes	Proc. Chnl. Descr.:	2998 PDA 254.0 nm (2998
Date Acquired:	7/10/2023 10:46:42 PM CST		
Date Processed:	8/1/2023 11:26:03 AM CST		

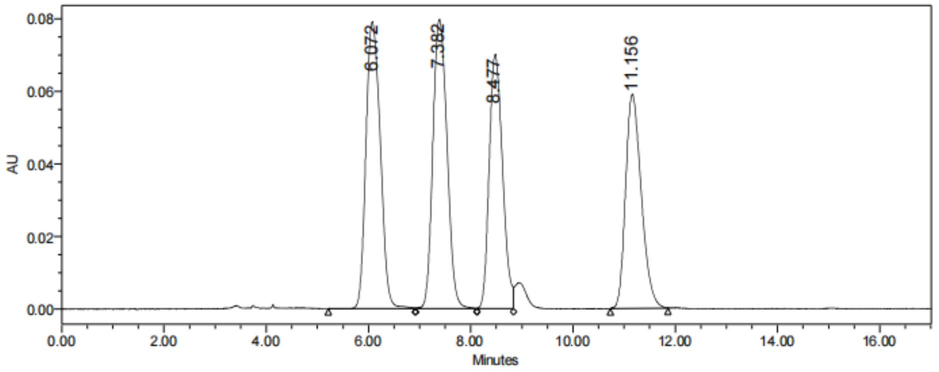

	RT	Area	% Area	Height
1	6.072	1547984	27.63	79008
2	7.382	1536855	27.43	79564
3	8.477	1261701	22.52	69952
4	11.156	1255455	22.41	58918

(*R*,*R*)-**3aa**

SAMPLE INFORMATION			
Sample Name:	cxh-11-30-2-asy-IC-1%	Acquired By:	System
Sample Type:	Unknown	Sample Set Name:	0711
Vial:	89	Acq. Method Set:	1%
Injection #:	1	Processing Method:	RR 3y
Injection Volume:	10.00 ul	Channel Name:	254.0nm
Run Time:	17.0 Minutes	Proc. Chnl. Descr.:	2998 PDA 254.0 nm (2998
Date Acquired:	7/10/2023 11:39:52 PM CST		
Date Processed:	8/1/2023 11:32:04 AM CST		

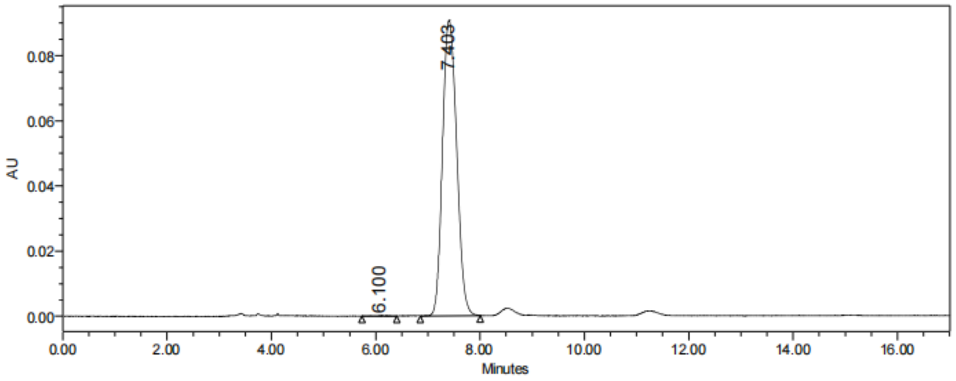

	RT	Area	% Area	Height
1	6.100	931	0.05	70
2	7.403	1717343	99.95	90697

Rac-**3aa**

SAMPLE INFORMATION			
Sample Name:	cxh-10-93-5-rac-IC-1%	Acquired By:	System
Sample Type:	Unknown	Sample Set Name:	0711
Vial:	86	Acq. Method Set:	1%
Injection #:	1	Processing Method:	3y rac
Injection Volume:	10.00 ul	Channel Name:	254.0nm
Run Time:	17.0 Minutes	Proc. Chnl. Descr.:	2998 PDA 254.0 nm (2998
Date Acquired:	7/10/2023 10:46:42 PM CST		
Date Processed:	8/1/2023 11:26:03 AM CST		

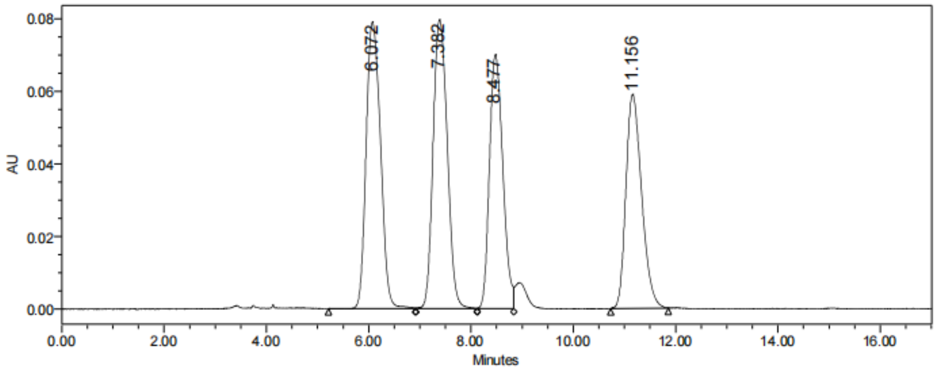

	RT	Area	% Area	Height
1	6.072	1547984	27.63	79008
2	7.382	1536855	27.43	79564
3	8.477	1261701	22.52	69952
4	11.156	1255455	22.41	58918

(*S*,*S*)-**3aa**

SAMPLE INFORMATION			
Sample Name:	cxh-11-30-3-asy-IC-1%	Acquired By:	System
Sample Type:	Unknown	Sample Set Name:	0711
Vial:	90	Acq. Method Set:	1%
Injection #:	1	Processing Method:	SS 3y
Injection Volume:	10.00 ul	Channel Name:	254.0nm
Run Time:	17.0 Minutes	Proc. Chnl. Descr.:	2998 PDA 254.0 nm (2998
Date Acquired:	7/10/2023 11:57:34 PM CST		
Date Processed:	8/1/2023 11:34:29 AM CST		

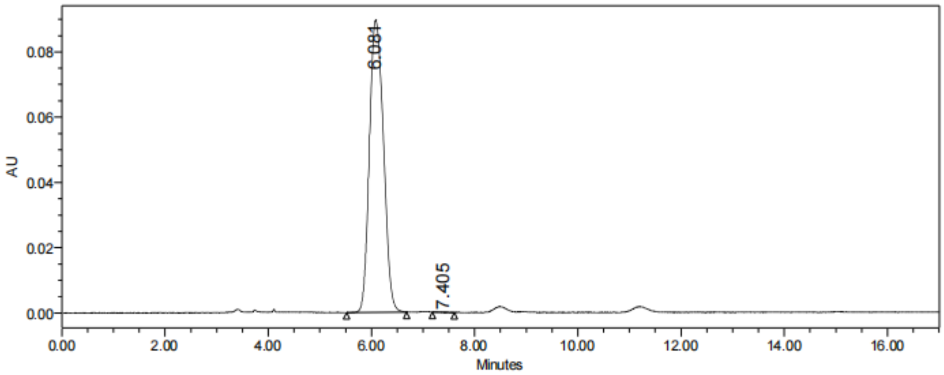

	RT	Area	% Area	Height
1	6.081	1698455	99.95	89550
2	7.405	896	0.05	-88

Rac-**3ab**

SAMPLE INFORMATION

Sample Name:	cxh-10-94-3-rac-IC-1%-0.5ML	Acquired By:	System
Sample Type:	Control	Sample Set Name:	
Vial:	80	Acq. Method Set:	1% 05ML
Injection #:	1	Processing Method:	3z rac
Injection Volume:	10.00 ul	Channel Name:	254.0nm
Run Time:	50.0 Minutes	Proc. Chnl. Descr.:	2998 PDA 254.0 nm (2998
Date Acquired:	11/15/2022 11:21:35 AM CST		
Date Processed:	8/1/2023 10:17:18 AM CST		

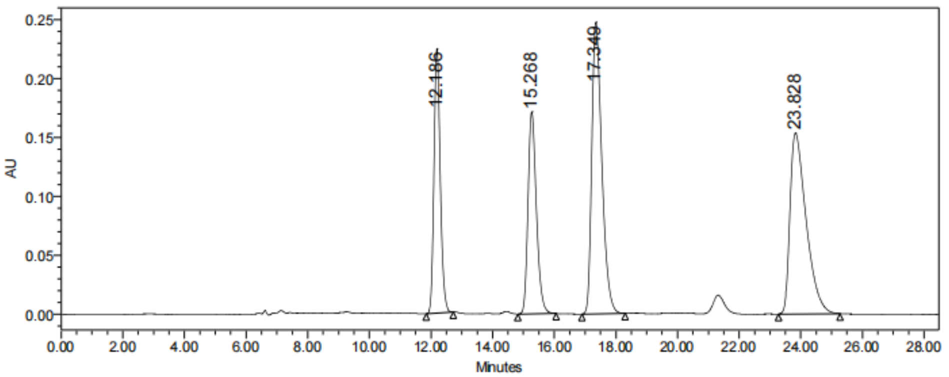

	RT	Area	% Area	Height
1	12.186	3189743	18.06	224384
2	15.268	3253936	18.42	171350
3	17.349	5614642	31.79	247314
4	23.828	5602395	31.72	153424

Asy-**3ab**

SAMPLE INFORMATION

Sample Name:	cxh-10-94-3-asy-IC-1%-0.5ML	Acquired By:	System
Sample Type:	Unknown	Sample Set Name:	1115
Vial:	83	Acq. Method Set:	1% 05ML
Injection #:	1	Processing Method:	3z asy
Injection Volume:	10.00 ul	Channel Name:	254.0nm
Run Time:	30.0 Minutes	Proc. Chnl. Descr.:	2998 PDA 254.0 nm (2998
Date Acquired:	11/15/2022 12:49:15 PM CST		
Date Processed:	8/1/2023 10:18:58 AM CST		

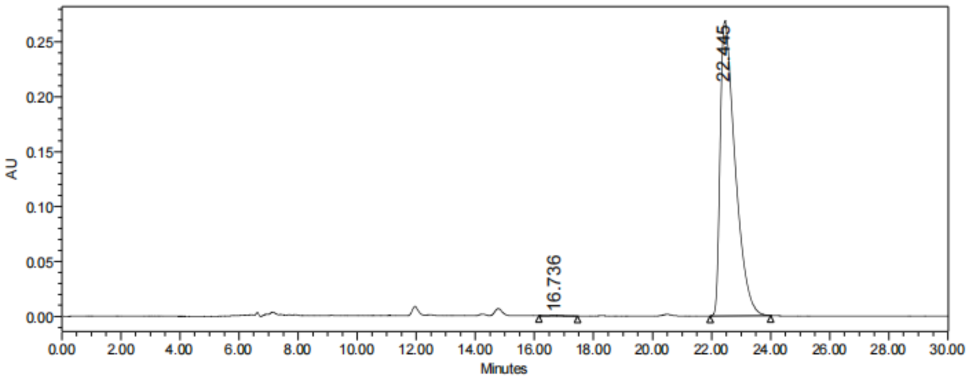

	RT	Area	% Area	Height
1	16.736	10827	0.11	489
2	22.445	9491662	99.89	268530

Rac-**3ac**

SAMPLE INFORMATION

Sample Name:	cxh-10-94-5-rac-IC-1%-0.5ML	Acquired By:	System
Sample Type:	Control	Sample Set Name:	
Vial:	46	Acq. Method Set:	1% 05ML
Injection #:	1	Processing Method:	3aa rac
Injection Volume:	10.00 ul	Channel Name:	254.0nm
Run Time:	50.0 Minutes	Proc. Chnl. Descr.:	2998 PDA 254.0 nm (2998
Date Acquired:	11/16/2022 12:59:34 PM CST		
Date Processed:	8/1/2023 10:22:01 AM CST		

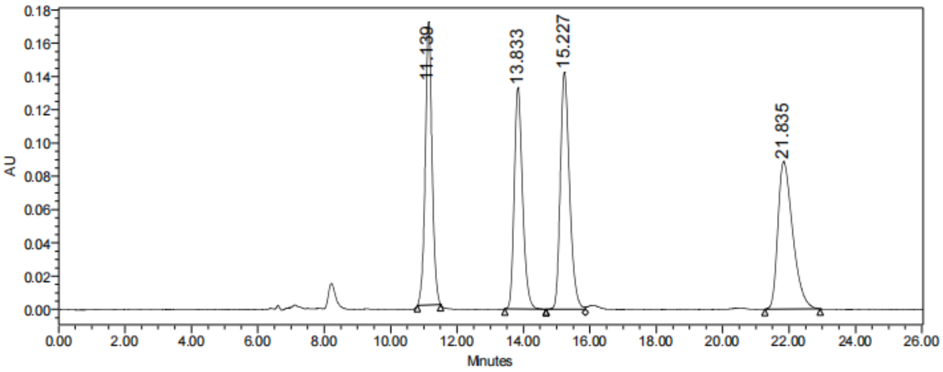

	RT	Area	% Area	Height
1	11.139	2407498	23.49	170126
2	13.833	2322217	22.66	133152
3	15.227	2758186	26.91	142437
4	21.835	2761535	26.94	88598

Asy-**3ac**

SAMPLE INFORMATION

Sample Name:	cxh-10-94-5-asy-IC-1%-0.5ML	Acquired By:	System
Sample Type:	Control	Sample Set Name:	
Vial:	46	Acq. Method Set:	1% 05ML
Injection #:	2	Processing Method:	3aa asy
Injection Volume:	10.00 ul	Channel Name:	254.0nm
Run Time:	28.0 Minutes	Proc. Chnl. Descr.:	2998 PDA 254.0 nm (2998
Date Acquired:	11/16/2022 1:26:33 PM CST		
Date Processed:	8/1/2023 10:23:27 AM CST		

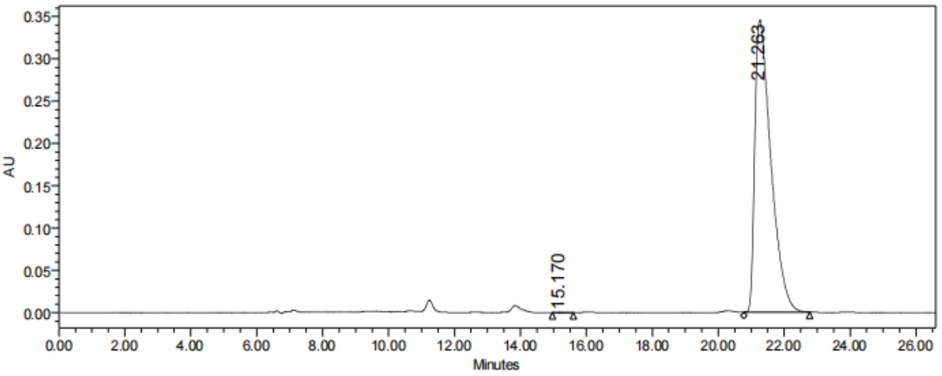

	RT	Area	% Area	Height
1	15.170	2278	0.02	165
2	21.263	11657177	99.98	344946

Rac-**3ad**

SAMPLE INFORMATION

Sample Name:	cxh-10-95-1-rac-IC-1%-0.5ML	Acquired By:	System
Sample Type:	Control	Sample Set Name:	
Vial:	20	Acq. Method Set:	1% 05ML
Injection #:	1	Processing Method:	3ab rac
Injection Volume:	10.00 ul	Channel Name:	254.0nm
Run Time:	50.0 Minutes	Proc. Chnl. Descr.:	2998 PDA 254.0 nm (2998
Date Acquired:	11/11/2022 9:55:41 AM CST		
Date Processed:	8/1/2023 10:08:05 AM CST		

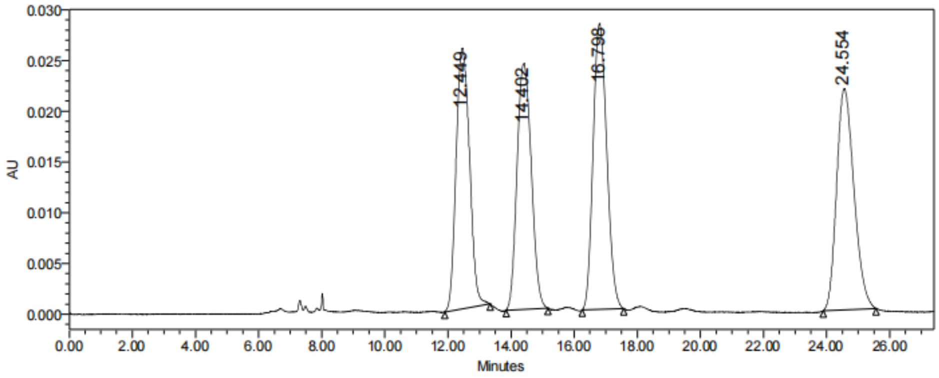

	RT	Area	% Area	Height
1	12.449	761400	23.75	25642
2	14.402	760638	23.73	24232
3	16.798	843992	26.33	28131
4	24.554	839300	26.18	21781

Asy-**3ad**

SAMPLE INFORMATION

Sample Name:	cxh-10-95-1-asy-IC-1%-0.5ML	Acquired By:	System
Sample Type:	Unknown	Sample Set Name:	0
Vial:	21	Acq. Method Set:	1% 05ML
Injection #:	1	Processing Method:	3ab asy
Injection Volume:	10.00 ul	Channel Name:	254.0nm
Run Time:	36.0 Minutes	Proc. Chnl. Descr.:	2998 PDA 254.0 nm (2998
Date Acquired:	11/11/2022 10:24:08 AM CST		
Date Processed:	8/1/2023 10:10:20 AM CST		

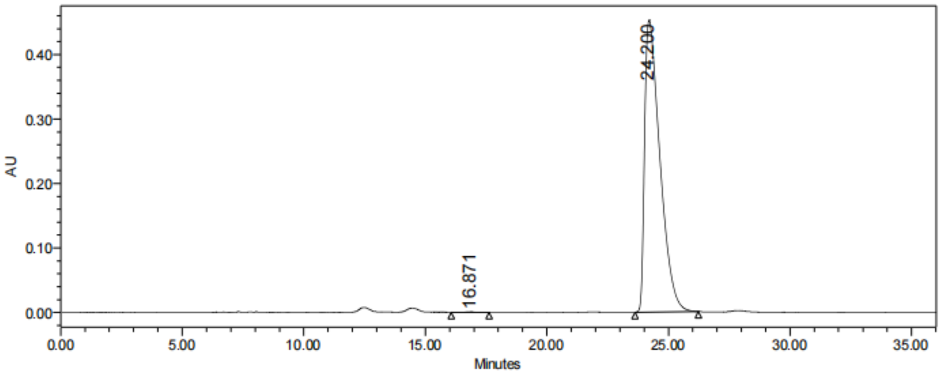

	RT	Area	% Area	Height
1	16.871	8711	0.04	283
2	24.200	19937131	99.96	452679

Rac-**3ae**

SAMPLE INFORMATION

Sample Name:	cxh-10-95-2-rac-IG-1%-0.5ML	Acquired By:	System
Sample Type:	Control	Sample Set Name:	
Vial:	6	Acq. Method Set:	1% 05ML
Injection #:	1	Processing Method:	3ac rac
Injection Volume:	10.00 ul	Channel Name:	254.0nm
Run Time:	50.0 Minutes	Proc. Chnl. Descr.:	2998 PDA 254.0 nm (2998
Date Acquired:	11/12/2022 3:06:50 PM CST		
Date Processed:	8/1/2023 10:31:14 AM CST		

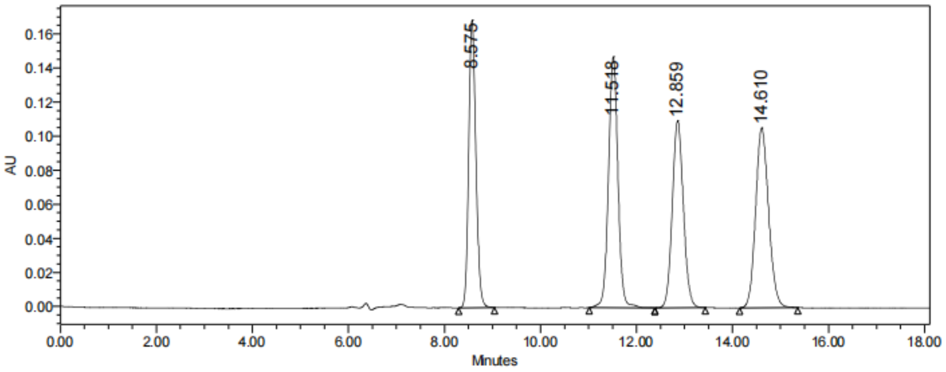

	RT	Area	% Area	Height
1	8.575	1758424	23.41	168812
2	11.518	2011871	26.79	147380
3	12.859	1756199	23.38	109745
4	14.610	1984485	26.42	105634

Asy-**3ae**

SAMPLE INFORMATION

Sample Name:	cxh-10-95-2-asy-IG-1%-0.5ML	Acquired By:	System
Sample Type:	Control	Sample Set Name:	
Vial:	7	Acq. Method Set:	1% 05ML
Injection #:	1	Processing Method:	3ac asy
Injection Volume:	10.00 ul	Channel Name:	254.0nm
Run Time:	20.0 Minutes	Proc. Chnl. Descr.:	2998 PDA 254.0 nm (2998
Date Acquired:	11/12/2022 3:29:18 PM CST		
Date Processed:	8/1/2023 10:32:41 AM CST		

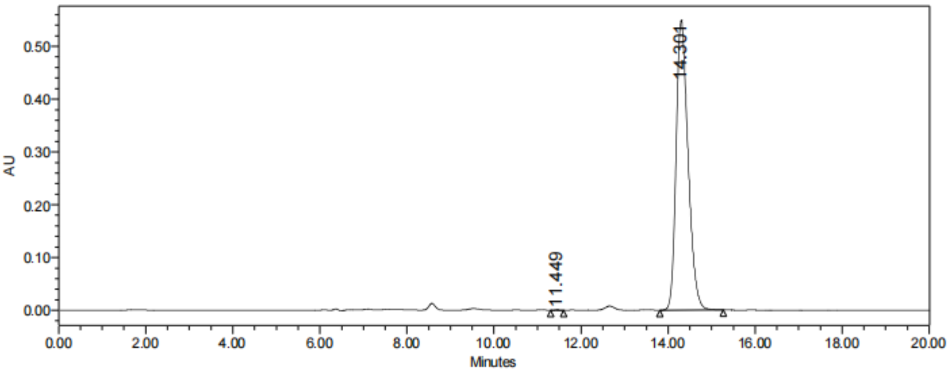

	RT	Area	% Area	Height
1	11.449	1626	0.02	168
2	14.301	10419907	99.98	548793

Rac-**3af**

SAMPLE INFORMATION			
Sample Name:	cxh-10-95-4-rac-IF-1%-0.5ML	Acquired By:	System
Sample Type:	Unknown	Sample Set Name:	1112
Vial:	9	Acq. Method Set:	1% 05ML
Injection #:	1	Processing Method:	3ad rac
Injection Volume:	10.00 ul	Channel Name:	254.0nm
Run Time:	30.0 Minutes	Proc. Chnl. Descr.:	2998 PDA 254.0 nm (2998
Date Acquired:	11/12/2022 9:39:21 AM CST		
Date Processed:	8/1/2023 10:38:55 AM CST		

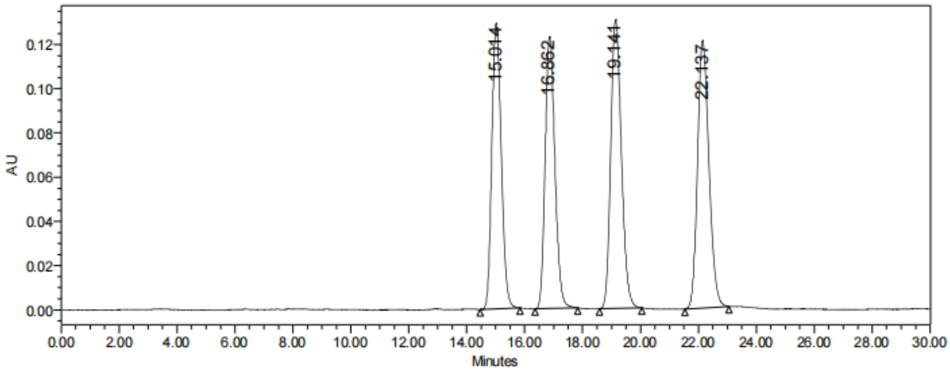

	RT	Area	% Area	Height
1	15.014	2878611	23.29	129159
2	16.862	2866644	23.19	123056
3	19.141	3314159	26.81	130455
4	22.137	3302338	26.71	120834

Asy-**3af**

SAMPLE INFORMATION			
Sample Name:	cxh-10-95-4-asy-IF-1%-0.5ML	Acquired By:	System
Sample Type:	Control	Sample Set Name:	
Vial:	8	Acq. Method Set:	1% 05ML
Injection #:	1	Processing Method:	3ad asy
Injection Volume:	10.00 ul	Channel Name:	254.0nm
Run Time:	30.0 Minutes	Proc. Chnl. Descr.:	2998 PDA 254.0 nm (2998
Date Acquired:	11/12/2022 9:06:13 AM CST		
Date Processed:	8/1/2023 10:39:54 AM CST		

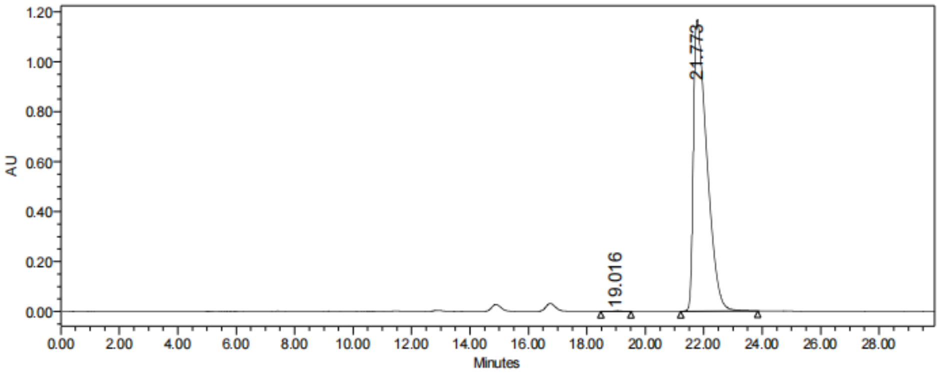

	RT	Area	% Area	Height
1	19.016	15802	0.04	791
2	21.773	36599516	99.96	1165866

Rac-**3ag**

SAMPLE INFORMATION

Sample Name:	cxh-10-94-2-rac-IC-1%-0.5ML	Acquired By:	System
Sample Type:	Control	Sample Set Name:	
Vial:	79	Acq. Method Set:	1% 05ML
Injection #:	1	Processing Method:	3ae rac1
Injection Volume:	10.00 ul	Channel Name:	254.0nm
Run Time:	50.0 Minutes	Proc. Chnl. Descr.:	2998 PDA 254.0 nm (2998
Date Acquired:	11/15/2022 4:03:47 PM CST		
Date Processed:	8/1/2023 10:12:09 AM CST		

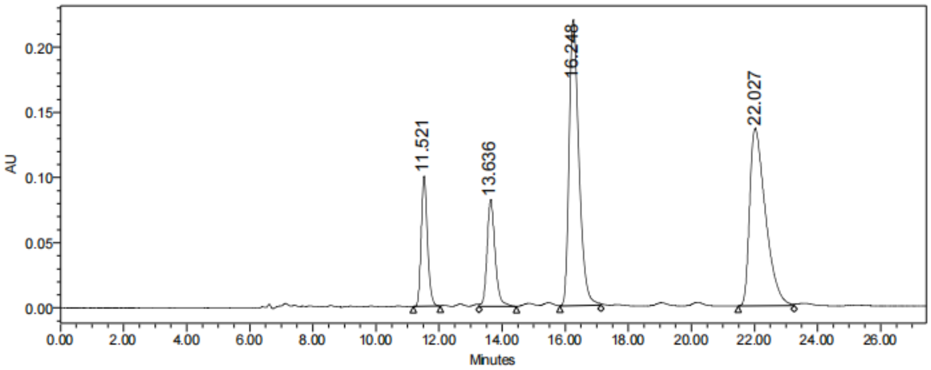

	RT	Area	% Area	Height
1	11.521	1350288	11.01	99592
2	13.636	1423909	11.61	81563
3	16.248	4761804	38.83	219019
4	22.027	4726118	38.54	135995

Asy-**3ag**

SAMPLE INFORMATION

Sample Name:	cxh-10-94-2-asy-IC-1%-0.5ML	Acquired By:	System
Sample Type:	Control	Sample Set Name:	
Vial:	82	Acq. Method Set:	1% 05ML
Injection #:	1	Processing Method:	3ae rac
Injection Volume:	10.00 ul	Channel Name:	254.0nm
Run Time:	50.0 Minutes	Proc. Chnl. Descr.:	2998 PDA 254.0 nm (2998
Date Acquired:	11/15/2022 3:35:57 PM CST		
Date Processed:	8/1/2023 10:16:17 AM CST		

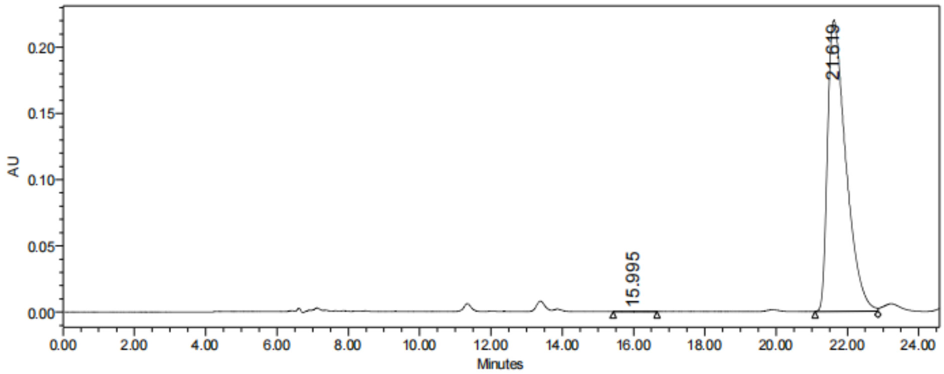

	RT	Area	% Area	Height
1	15.995	2432	0.03	94
2	21.619	7809820	99.97	219806

Rac-**3ah**

SAMPLE INFORMATION			
Sample Name:	cxh-10-95-3-rac-IF-1%-0.5ML	Acquired By:	System
Sample Type:	Control	Sample Set Name:	
Vial:	9	Acq. Method Set:	1% 05ML
Injection #:	1	Processing Method:	3af rac
Injection Volume:	10.00 ul	Channel Name:	254.0nm
Run Time:	60.0 Minutes	Proc. Chnl. Descr.:	2998 PDA 254.0 nm (2998
Date Acquired:	11/12/2022 1:21:43 PM CST		
Date Processed:	8/1/2023 10:34:27 AM CST		

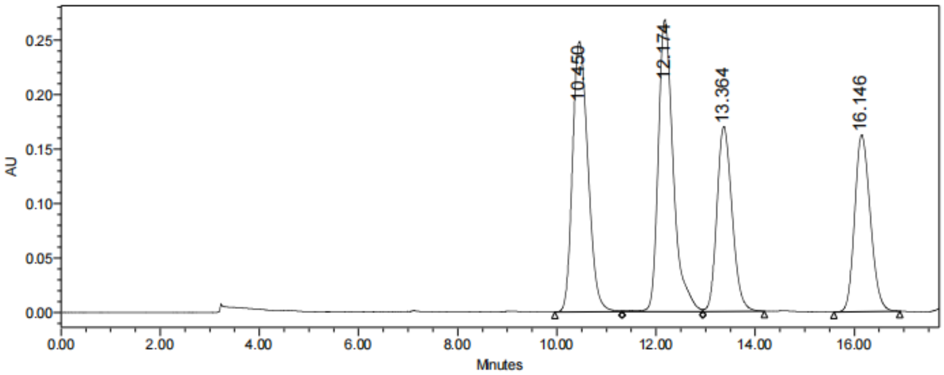

	RT	Area	% Area	Height
1	10.450	5373989	29.47	247732
2	12.174	5668474	31.09	267241
3	13.364	3624525	19.88	169234
4	16.146	3568062	19.57	161830

Asy-**3ah**

SAMPLE INFORMATION			
Sample Name:	cxh-10-95-3-asy-IF-1%-0.5ML	Acquired By:	System
Sample Type:	Control	Sample Set Name:	
Vial:	10	Acq. Method Set:	1% 05ML
Injection #:	1	Processing Method:	3af asy
Injection Volume:	10.00 ul	Channel Name:	254.0nm
Run Time:	25.0 Minutes	Proc. Chnl. Descr.:	2998 PDA 254.0 nm (2998
Date Acquired:	11/12/2022 1:45:01 PM CST		
Date Processed:	8/1/2023 10:35:33 AM CST		

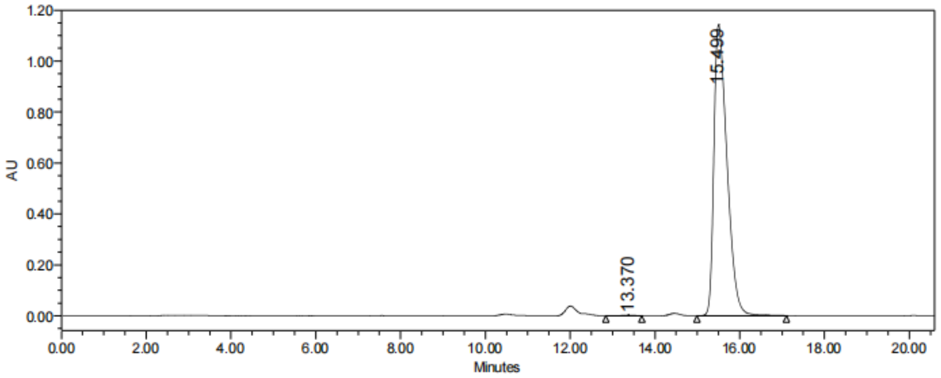

	RT	Area	% Area	Height
1	13.370	40136	0.16	2281
2	15.499	24606790	99.84	1142339

Rac-**3ai**

SAMPLE INFORMATION

Sample Name:	cxh-10-92-3-rac-IE-1%-0.5ML	Acquired By:	System
Sample Type:	Control	Sample Set Name:	
Vial:	5	Acq. Method Set:	1% 05ML
Injection #:	1	Processing Method:	3ag rac
Injection Volume:	10.00 ul	Channel Name:	254.0nm
Run Time:	60.0 Minutes	Proc. Chnl. Descr.:	2998 PDA 254.0 nm (2998
Date Acquired:	11/1/2022 2:26:58 PM CST		
Date Processed:	8/1/2023 9:50:37 AM CST		

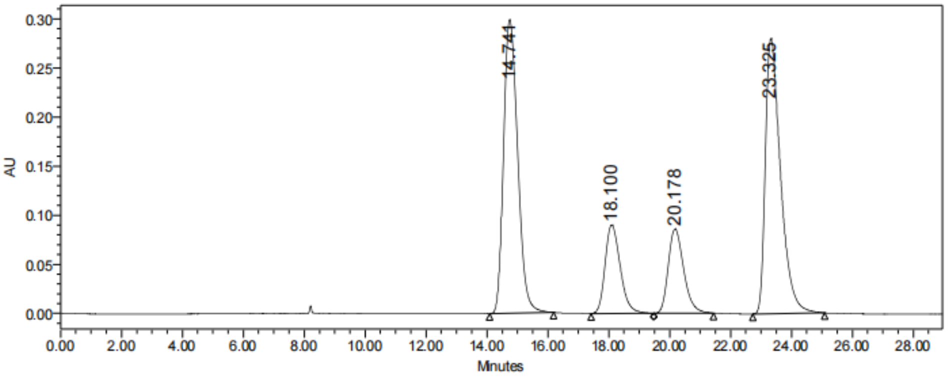

	RT	Area	% Area	Height
1	14.741	9621109	37.88	298742
2	18.100	3131025	12.33	90299
3	20.178	3041236	11.97	86018
4	23.325	9607123	37.82	279990

Asy-**3ai**

SAMPLE INFORMATION

Sample Name:	cxh-10-92-3-asy-IE-1%-0.5ML	Acquired By:	System
Sample Type:	Control	Sample Set Name:	
Vial:	9	Acq. Method Set:	1% 05ML
Injection #:	1	Processing Method:	3ag asy
Injection Volume:	10.00 ul	Channel Name:	254.0nm
Run Time:	30.0 Minutes	Proc. Chnl. Descr.:	2998 PDA 254.0 nm (2998
Date Acquired:	11/2/2022 9:18:54 AM CST		
Date Processed:	8/1/2023 9:55:54 AM CST		

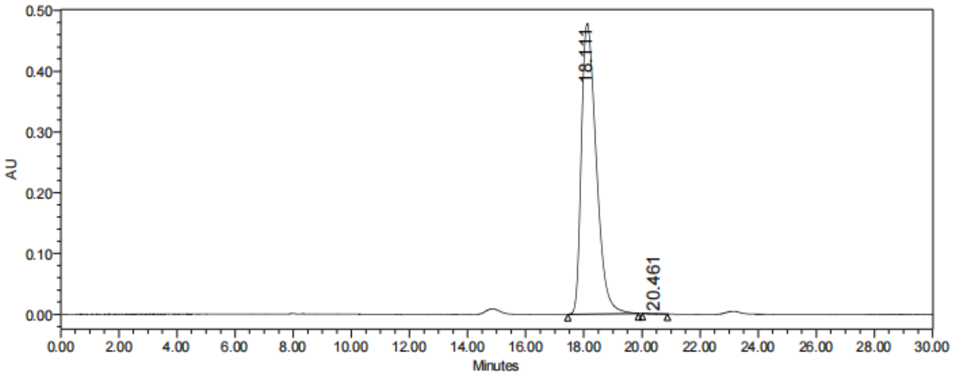

	RT	Area	% Area	Height
1	18.111	17105124	99.96	477610
2	20.461	6799	0.04	-232

Rac-**3aj**

SAMPLE INFORMATION

Sample Name:	cxh-10-92-4-rac-IG-1%-0.5ML	Acquired By:	System
Sample Type:	Control	Sample Set Name:	
Vial:	115	Acq. Method Set:	1% 05ML
Injection #:	1	Processing Method:	3ah rac
Injection Volume:	10.00 ul	Channel Name:	254.0nm
Run Time:	60.0 Minutes	Proc. Chnl. Descr.:	2998 PDA 254.0 nm (2998
Date Acquired:	11/3/2022 11:34:53 PM CST		
Date Processed:	8/1/2023 9:57:43 AM CST		

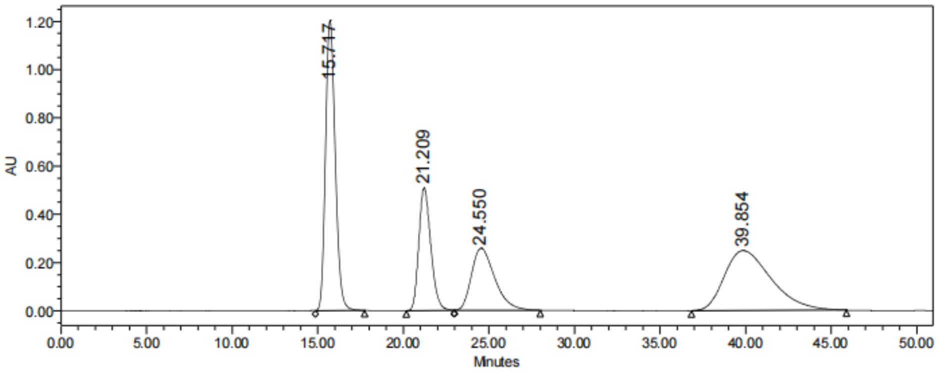

	RT	Area	% Area	Height
1	15.717	47656013	33.08	1203041
2	21.209	24669030	17.12	508943
3	24.550	24406977	16.94	257967
4	39.854	47345450	32.86	248125

Asy-**3aj**

SAMPLE INFORMATION

Sample Name:	cxh-10-92-4-asy-IG-1%-0.5ML	Acquired By:	System
Sample Type:	Control	Sample Set Name:	
Vial:	116	Acq. Method Set:	1% 05ML
Injection #:	1	Processing Method:	3ah asy
Injection Volume:	10.00 ul	Channel Name:	254.0nm
Run Time:	60.0 Minutes	Proc. Chnl. Descr.:	2998 PDA 254.0 nm (2998
Date Acquired:	11/3/2022 10:42:35 PM CST		
Date Processed:	8/1/2023 9:59:57 AM CST		

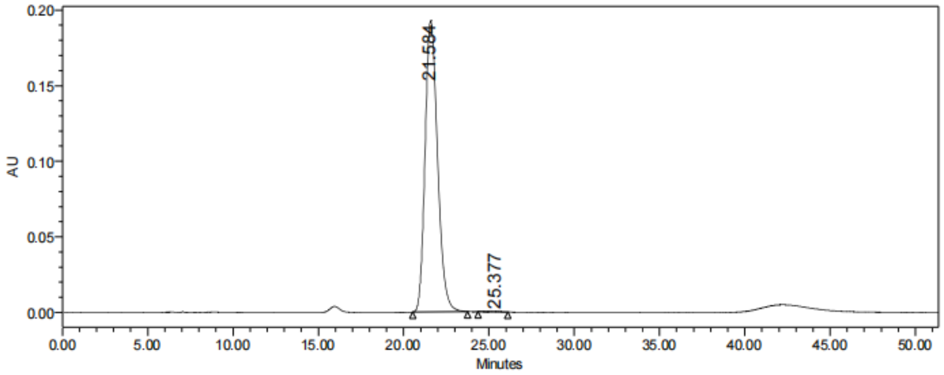

	RT	Area	% Area	Height
1	21.584	9694432	99.90	192694
2	25.377	9524	0.10	226

Rac-**3ak**

S A M P L E   I N F O R M A T I O N

Sample Name:	cxh-10-91-5-rac-IE-1%-0.5ML	Acquired By:	System
Sample Type:	Control	Sample Set Name:	
Vial:	66	Acq. Method Set:	1% 05ML
Injection #:	1	Processing Method:	3ai rac
Injection Volume:	10.00 ul	Channel Name:	254.0nm
Run Time:	60.0 Minutes	Proc. Chnl. Descr.:	2998 PDA 254.0 nm (2998
Date Acquired:	10/31/2022 8:21:39 PM CST		
Date Processed:	8/1/2023 10:46:53 AM CST		

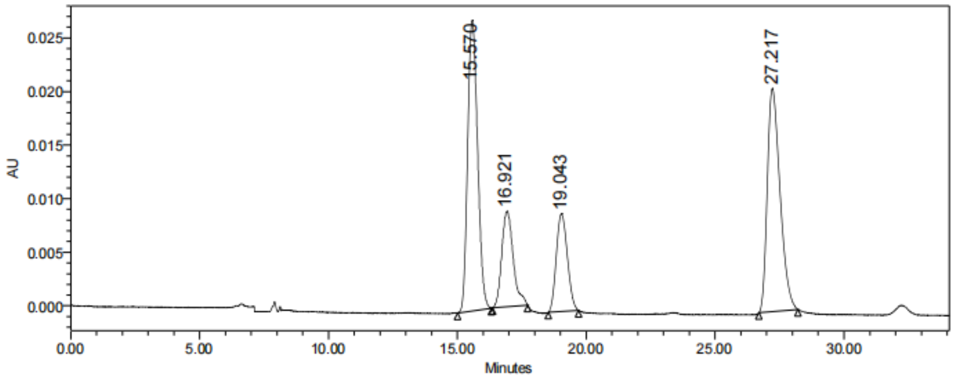

	RT	Area	% Area	Height
1	15.570	729812	36.62	27092
2	16.921	278518	13.97	8908
3	19.043	270985	13.60	9133
4	27.217	713717	35.81	20824

Asy-**3ak**

S A M P L E   I N F O R M A T I O N

Sample Name:	cxh-10-91-5-asy-IE-1%-0.5ML	Acquired By:	System
Sample Type:	Control	Sample Set Name:	
Vial:	82	Acq. Method Set:	1% 05ML
Injection #:	1	Processing Method:	3ai asy
Injection Volume:	10.00 ul	Channel Name:	254.0nm
Run Time:	40.0 Minutes	Proc. Chnl. Descr.:	2998 PDA 254.0 nm (2998
Date Acquired:	10/31/2022 8:57:28 PM CST		
Date Processed:	8/1/2023 10:48:30 AM CST		

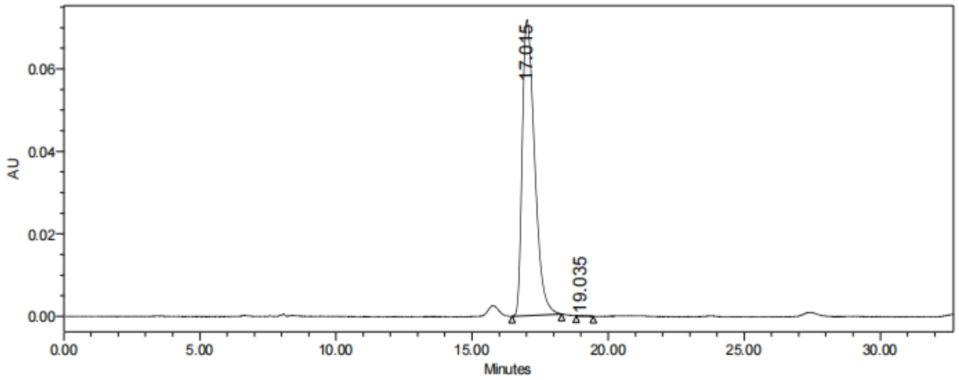

	RT	Area	% Area	Height
1	17.015	2277513	99.98	71592
2	19.035	568	0.02	-39

Rac-**3al**

SAMPLE INFORMATION

Sample Name:	cxh-10-95-5-IC-1%-0.5ML	Acquired By:	System
Sample Type:	Control	Sample Set Name:	
Vial:	56	Acq. Method Set:	1% 05ML
Injection #:	1	Processing Method:	3aj rac
Injection Volume:	10.00 ul	Channel Name:	254.0nm
Run Time:	60.0 Minutes	Proc. Chnl. Descr.:	2998 PDA 254.0 nm (2998
Date Acquired:	11/12/2022 7:47:11 PM CST		
Date Processed:	8/1/2023 10:41:44 AM CST		

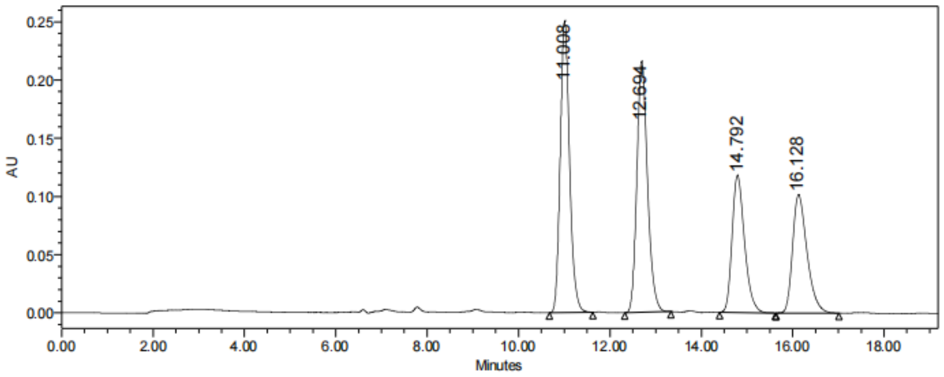

	RT	Area	% Area	Height
1	11.008	3494084	30.46	250501
2	12.694	3497792	30.49	215656
3	14.792	2236787	19.50	117696
4	16.128	2243131	19.55	101511

Asy-**3al**

SAMPLE INFORMATION

Sample Name:	cxh-10-95-5-asy-IC-1%-0.5ML	Acquired By:	System
Sample Type:	Control	Sample Set Name:	
Vial:	57	Acq. Method Set:	1% 05ML
Injection #:	1	Processing Method:	3aj asy
Injection Volume:	10.00 ul	Channel Name:	254.0nm
Run Time:	25.0 Minutes	Proc. Chnl. Descr.:	2998 PDA 254.0 nm (2998
Date Acquired:	11/12/2022 8:10:49 PM CST		
Date Processed:	8/1/2023 10:43:57 AM CST		

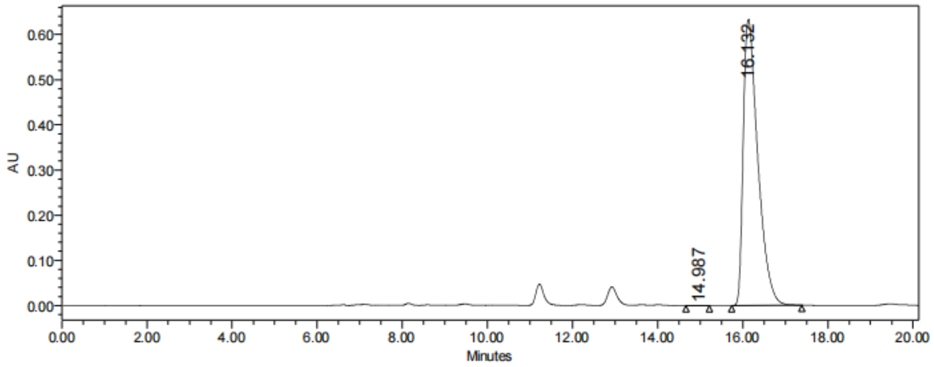

	RT	Area	% Area	Height
1	14.987	2770	0.02	193
2	16.132	14996895	99.98	631993

Rac-**3am**

SAMPLE INFORMATION

Sample Name:	cxh-12-1-7-rac-IA-1%	Acquired By:	System
Sample Type:	Unknown	Sample Set Name	0417
Vial:	82	Acq. Method Set:	1%
Injection #:	1	Processing Method	ssda
Injection Volume:	10.00 ul	Channel Name:	254.0nm
Run Time:	50.0 Minutes	Proc. Chnl. Descr.:	2998 PDA 254.0 nm (2998
Date Acquired:	4/17/2024 17:40:54 CST		
Date Processed:	4/23/2024 16:15:23 CST		

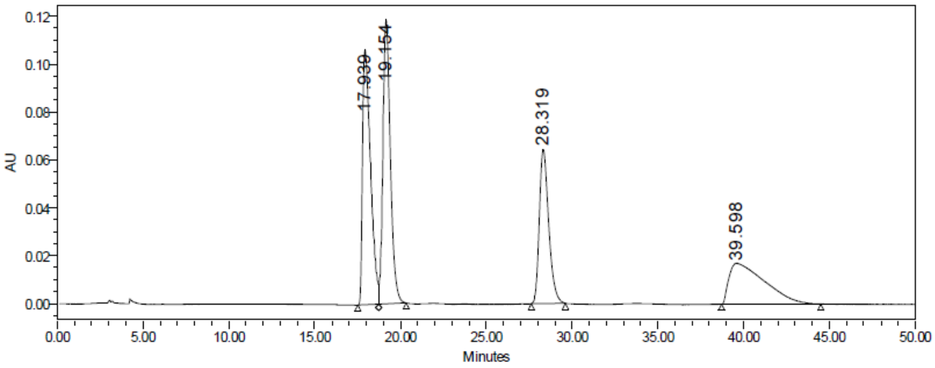

	RT	Area	% Area	Height
1	17.939	3448780	28.95	106420
2	19.154	3553317	29.82	118506
3	28.319	2456664	20.62	64396
4	39.598	2455676	20.61	17107

Asy-**3am**

SAMPLE INFORMATION

Sample Name:	cxh-12-1-7-asy-IA-1%	Acquired By:	System
Sample Type:	Unknown	Sample Set Name	0417
Vial:	83	Acq. Method Set:	1%
Injection #:	1	Processing Method	3d2
Injection Volume:	10.00 ul	Channel Name:	254.0nm
Run Time:	50.0 Minutes	Proc. Chnl. Descr.:	2998 PDA 254.0 nm (2998
Date Acquired:	4/17/2024 18:31:50 CST		
Date Processed:	4/23/2024 16:19:41 CST		

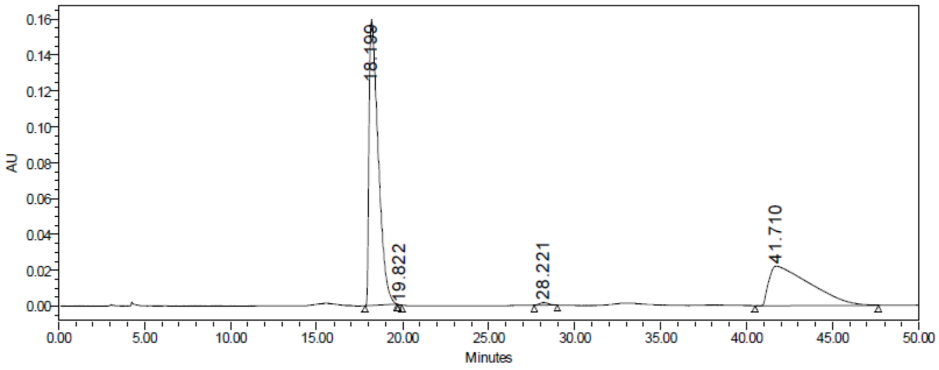

	RT	Area	% Area	Height
1	18.199	5857066	61.32	159039
2	19.822	636	0.01	-104
3	28.221	52903	0.55	1507
4	41.710	3641028	38.12	21983

Rac-**3an** (major conformational)

SAMPLE INFORMATION

Sample Name:	cxh-12-1-3-up-rac-IG-2%	Acquired By:	System
Sample Type:	Unknown	Sample Set Name	
Vial:	85	Acq. Method Set:	2%
Injection #:	2	Processing Method	3k2
Injection Volume:	10.00 ul	Channel Name:	254.0nm
Run Time:	50.0 Minutes	Proc. Chnl. Descr.:	2998 PDA 254.0 nm (2998
Date Acquired:	4/18/2024 9:02:10 CST		
Date Processed:	4/23/2024 16:24:24 CST		

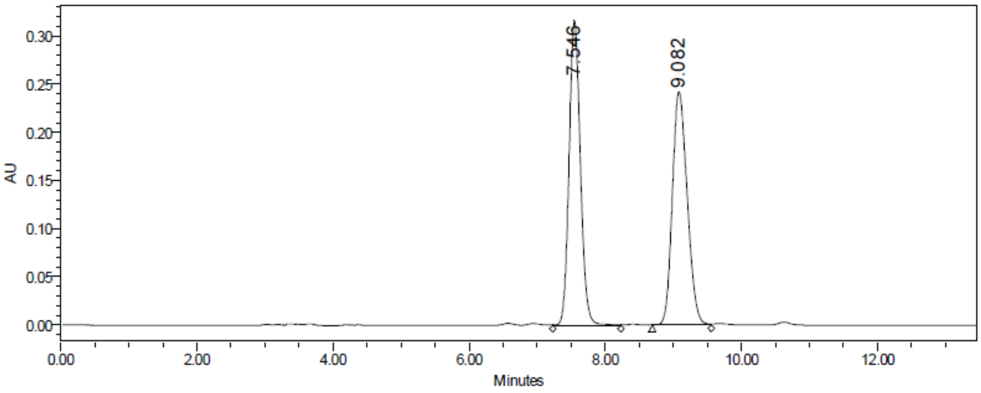

	RT	Area	% Area	Height
1	7.546	3649641	49.97	316046
2	9.082	3654658	50.03	242274

Asy-**3an** (major conformational)

SAMPLE INFORMATION

Sample Name:	cxh-12-1-3-up-asy-IG-2%	Acquired By:	System
Sample Type:	Unknown	Sample Set Name	
Vial:	86	Acq. Method Set:	2%
Injection #:	1	Processing Method	3z rac
Injection Volume:	10.00 ul	Channel Name:	254.0nm
Run Time:	15.0 Minutes	Proc. Chnl. Descr.:	2998 PDA 254.0 nm (2998
Date Acquired:	4/18/2024 9:16:37 CST		
Date Processed:	4/23/2024 16:24:41 CST		

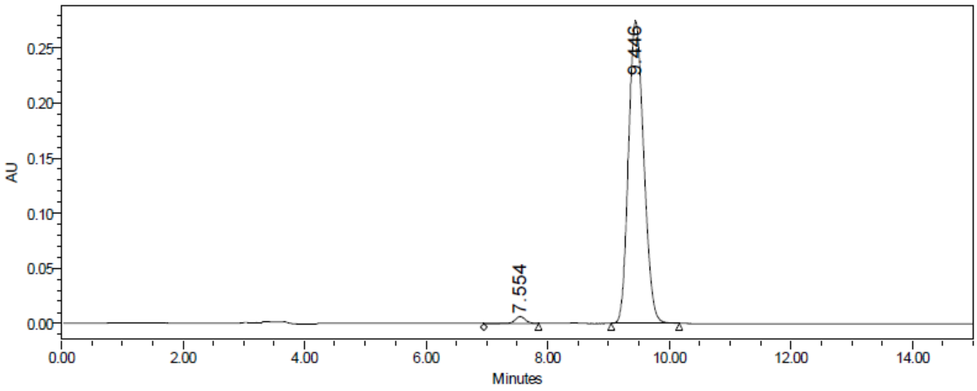

	RT	Area	% Area	Height
1	7.554	80619	1.65	6252
2	9.446	4815897	98.35	274560

Rac-**3an** (minor conformational)

SAMPLE INFORMATION

Sample Name:	cxh-12-1-3-down-rac-IG-1%-0.5ML	Acquired By:	System
Sample Type:	Control	Sample Set Name:	
Vial:	98	Acq. Method Set:	1% 05ML
Injection #:	1	Processing Method:	021677
Injection Volume:	10.00 ul	Channel Name:	254.0nm
Run Time:	50.0 Minutes	Proc. Chnl. Descr.:	2998 PDA 254.0 nm (2998
Date Acquired:	4/18/2024 12:59:13 PM CST		
Date Processed:	4/23/2024 4:28:26 PM CST		

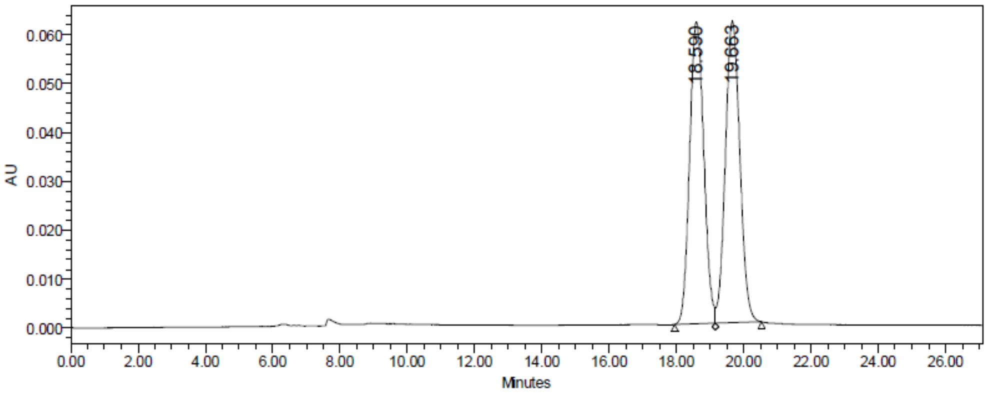

	RT	Area	% Area	Height
1	18.590	1818123	49.61	61690
2	19.663	1846644	50.39	61628

Asy-**3an** (minor conformational)

SAMPLE INFORMATION

Sample Name:	cxh-12-1-3-down-asy-IG-1%-0.5ML	Acquired By:	System
Sample Type:	Control	Sample Set Name:	
Vial:	20	Acq. Method Set:	1% 05ML
Injection #:	1	Processing Method:	021644
Injection Volume:	10.00 ul	Channel Name:	254.0nm
Run Time:	28.0 Minutes	Proc. Chnl. Descr.:	2998 PDA 254.0 nm (2998
Date Acquired:	4/18/2024 1:27:23 PM CST		
Date Processed:	4/23/2024 4:29:26 PM CST		

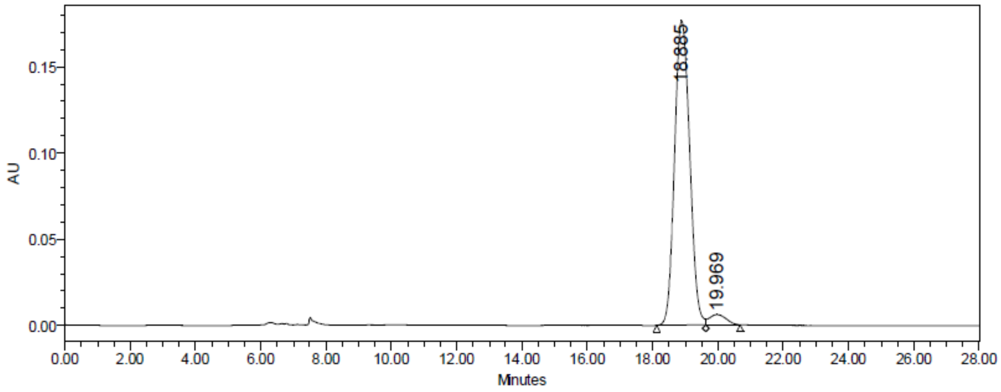

	RT	Area	% Area	Height
1	18.885	5710344	96.37	176632
2	19.969	215041	3.63	6054

Rac-**7a**

SAMPLE INFORMATION

Sample Name:	cxh-10-78-6-rac-AD-1%	Acquired By:	System
Sample Type:	Unknown	Sample Set Name:	09132
Vial:	6	Acq. Method Set:	1%
Injection #:	1	Processing Method:	7a rac
Injection Volume:	10.00 ul	Channel Name:	254.0nm
Run Time:	20.0 Minutes	Proc. Chnl. Descr.:	2998 PDA 254.0 nm (2998
Date Acquired:	9/13/2022 1:15:23 PM CST		
Date Processed:	7/31/2023 7:35:20 PM CST		

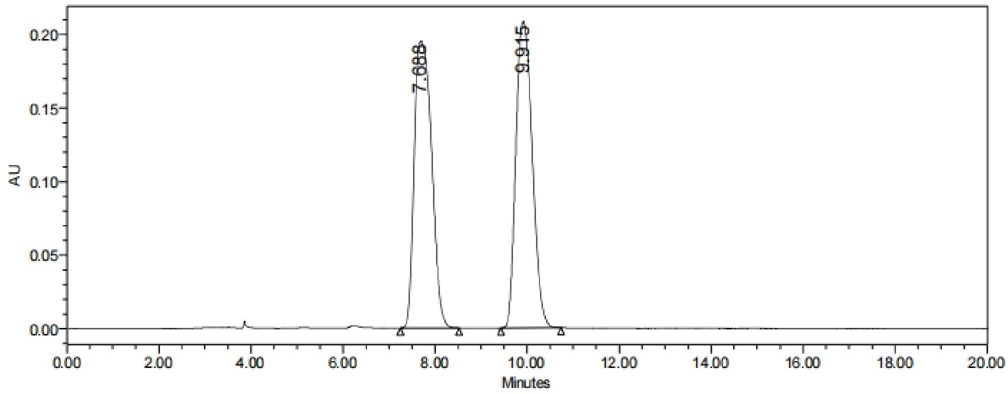

	RT	Area	% Area	Height
1	7.688	5155328	50.13	195011
2	9.915	5129505	49.87	208384

Asy-**7a**

SAMPLE INFORMATION

Sample Name:	cxh-10-78-6-asy-AD-1%	Acquired By:	System
Sample Type:	Unknown	Sample Set Name:	09133
Vial:	98	Acq. Method Set:	1%
Injection #:	1	Processing Method:	7a asy
Injection Volume:	10.00 ul	Channel Name:	254.0nm
Run Time:	16.0 Minutes	Proc. Chnl. Descr.:	2998 PDA 254.0 nm (2998
Date Acquired:	9/13/2022 7:16:09 PM CST		
Date Processed:	7/31/2023 7:36:00 PM CST		

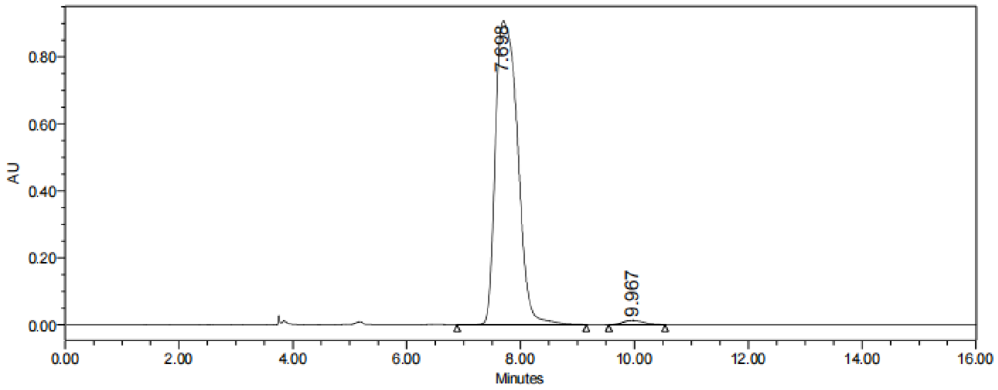

	RT	Area	% Area	Height
1	7.698	24440617	98.70	906105
2	9.967	321184	1.30	12719

Rac-**7b**

SAMPLE INFORMATION

Sample Name:	cxh-10-78-3-rac-AD-1%	Acquired By:	System
Sample Type:	Unknown	Sample Set Name:	
Vial:	2	Acq. Method Set:	1%
Injection #:	2	Processing Method	78 3 rac
Injection Volume:	10.00 ul	Channel Name:	254.0nm
Run Time:	40.0 Minutes	Proc. Chnl. Descr.:	2998 PDA 254.0 nm (2998
Date Acquired:	9/7/2022 11:06:51 CST		
Date Processed:	7/31/2023 16:17:27 CST		

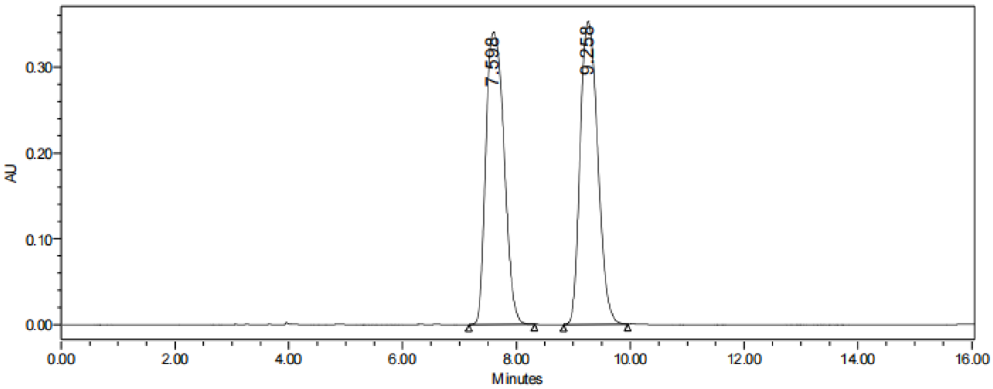

	RT	Area	% Area	Height
1	7.598	7635044	50.07	340103
2	9.258	7613362	49.93	352579

Asy-**7b**

SAMPLE INFORMATION

Sample Name:	cxh-10-78-3-AD-1%	Acquired By:	System
Sample Type:	Control	Sample Set Name:	
Vial:	72	Acq. Method Set:	1%
Injection #:	1	Processing Method:	7b asy
Injection Volume:	10.00 ul	Channel Name:	254.0nm
Run Time:	25.0 Minutes	Proc. Chnl. Descr.:	2998 PDA 254.0 nm (2998
Date Acquired:	9/8/2022 10:57:27 AM CST		
Date Processed:	7/31/2023 6:13:08 PM CST		

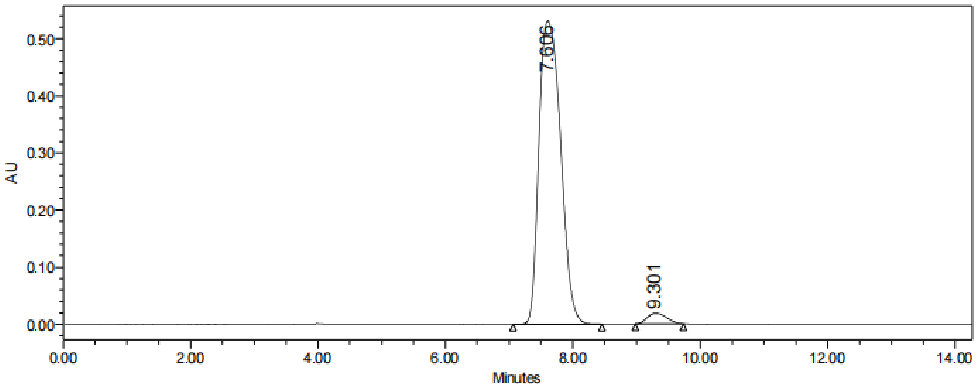

	RT	Area	% Area	Height
1	7.606	12601406	96.94	530702
2	9.301	397992	3.06	18611

Rac-**7c**

SAMPLE INFORMATION

Sample Name:	cxh-10-78-4-rac-AD-1%	Acquired By:	System
Sample Type:	Unknown	Sample Set Name:	09132
Vial:	4	Acq. Method Set:	1%
Injection #:	1	Processing Method:	7c rac
Injection Volume:	10.00 ul	Channel Name:	254.0nm
Run Time:	20.0 Minutes	Proc. Chnl. Descr.:	2998 PDA 254.0 nm (2998
Date Acquired:	9/13/2022 12:34:02 PM CST		
Date Processed:	7/31/2023 7:32:40 PM CST		

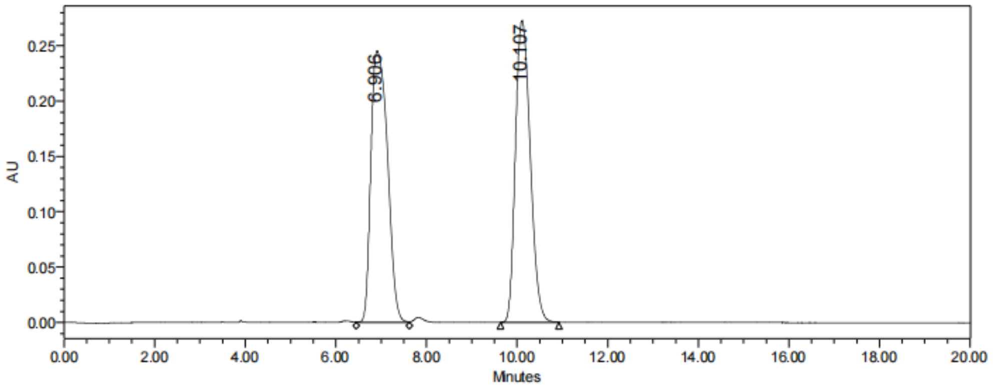

	RT	Area	% Area	Height
1	6.906	6201654	50.01	244933
2	10.107	6199360	49.99	272272

Asy-**7c**

SAMPLE INFORMATION

Sample Name:	cxh-10-78-4-asy-AD-1%	Acquired By:	System
Sample Type:	Unknown	Sample Set Name:	09133
Vial:	97	Acq. Method Set:	1%
Injection #:	1	Processing Method:	7c asy
Injection Volume:	10.00 ul	Channel Name:	254.0nm
Run Time:	16.0 Minutes	Proc. Chnl. Descr.:	2998 PDA 254.0 nm (2998
Date Acquired:	9/13/2022 6:59:26 PM CST		
Date Processed:	7/31/2023 7:33:17 PM CST		

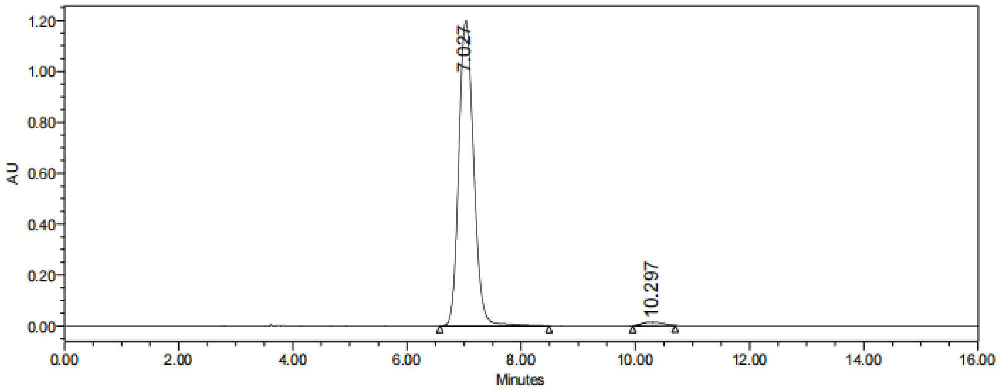

	RT	Area	% Area	Height
1	7.027	21684510	98.48	1196535
2	10.297	334891	1.52	13350

Rac-**7d**

SAMPLE INFORMATION

Sample Name:	cxh-10-78-5-rac-AD-1%	Acquired By:	System
Sample Type:	Unknown	Sample Set Name:	09133
Vial:	99	Acq. Method Set:	1%
Injection #:	1	Processing Method:	7d rac
Injection Volume:	10.00 ul	Channel Name:	254.0nm
Run Time:	20.0 Minutes	Proc. Chnl. Descr.:	2998 PDA 254.0 nm (2998
Date Acquired:	9/13/2022 7:32:53 PM CST		
Date Processed:	7/31/2023 7:37:36 PM CST		

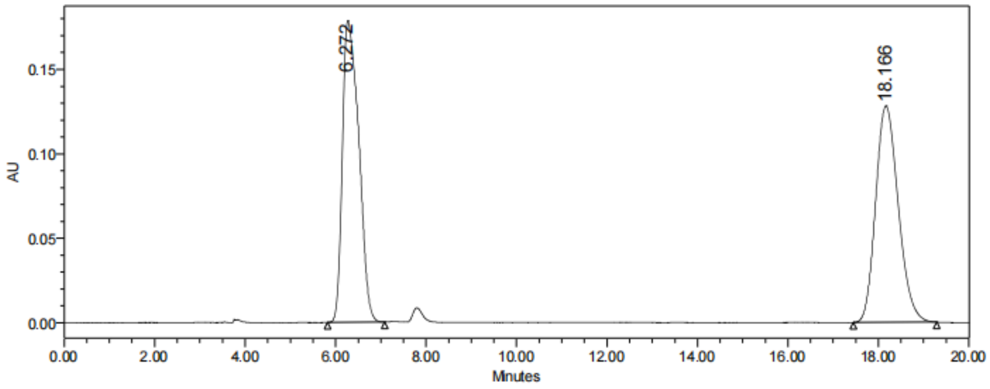

	RT	Area	% Area	Height
1	6.272	4486167	50.13	178534
2	18.166	4462470	49.87	128256

Asy-**7d**

SAMPLE INFORMATION

Sample Name:	cxh-10-78-5-asy-AD-1%	Acquired By:	System
Sample Type:	Control	Sample Set Name:	
Vial:	12	Acq. Method Set:	1%
Injection #:	1	Processing Method:	7d asy
Injection Volume:	10.00 ul	Channel Name:	254.0nm
Run Time:	25.0 Minutes	Proc. Chnl. Descr.:	2998 PDA 254.0 nm (2998
Date Acquired:	9/13/2022 8:54:22 PM CST		
Date Processed:	7/31/2023 7:38:35 PM CST		

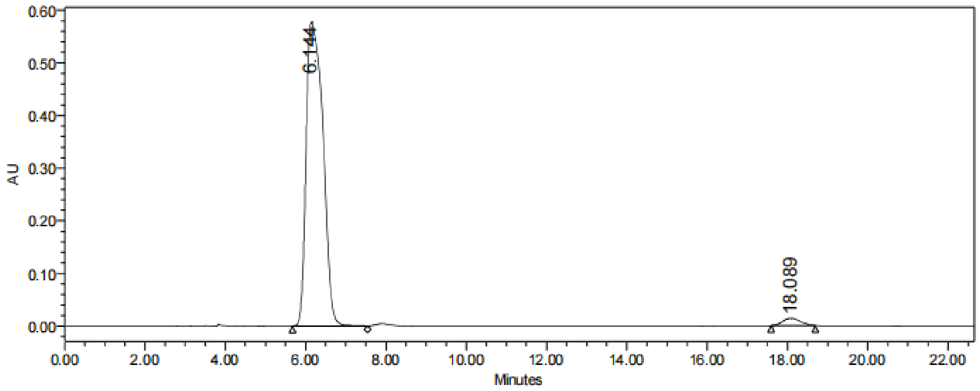

	RT	Area	% Area	Height
1	6.144	16781473	97.45	576574
2	18.089	439751	2.55	14340

Rac-**7e**

SAMPLE INFORMATION

Sample Name:	cxh-10-79-2-rac-AD-1%	Acquired By:	System
Sample Type:	Unknown	Sample Set Name:	0919
Vial:	12	Acq. Method Set:	1%
Injection #:	1	Processing Method	7e rac
Injection Volume:	10.00 ul	Channel Name:	254.0nm
Run Time:	20.0 Minutes	Proc. Chnl. Descr.:	2998 PDA 254.0 nm (2998
Date Acquired:	9/19/2022 19:18:19 CST		
Date Processed:	7/31/2023 16:34:15 CST		

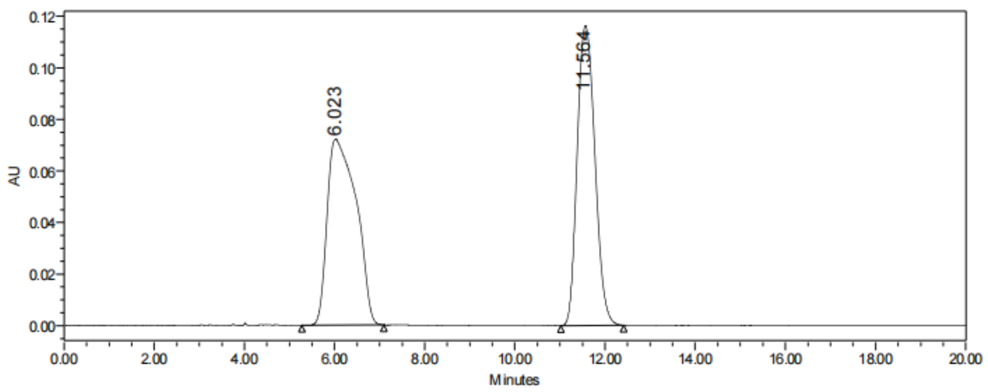

	RT	Area	% Area	Height
1	6.023	3212973	50.09	72104
2	11.564	3202041	49.91	116068

Asy-**7e**

SAMPLE INFORMATION

Sample Name:	cxh-10-79-2-asy-AD-1%	Acquired By:	System
Sample Type:	Unknown	Sample Set Name:	09193
Vial:	42	Acq. Method Set:	1%
Injection #:	1	Processing Method	7e asy
Injection Volume:	10.00 ul	Channel Name:	254.0nm
Run Time:	18.0 Minutes	Proc. Chnl. Descr.:	2998 PDA 254.0 nm (2998
Date Acquired:	9/19/2022 22:33:14 CST		
Date Processed:	7/31/2023 16:38:17 CST		

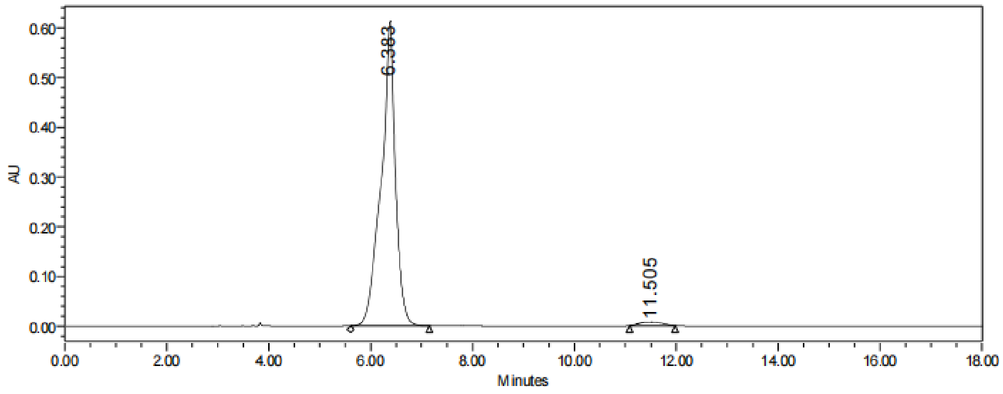

	RT	Area	% Area	Height
1	6.383	11935756	98.05	611937
2	11.505	237829	1.95	7570

Rac-**7f**

SAMPLE INFORMATION			
Sample Name:	cxh-10-79-3-rac-IA-1%	Acquired By:	System
Sample Type:	Control	Sample Set Name:	
Vial:	8	Acq. Method Set:	1%
Injection #:	3	Processing Method:	7f rac
Injection Volume:	10.00 ul	Channel Name:	254.0nm
Run Time:	25.0 Minutes	Proc. Chnl. Descr.:	2998 PDA 254.0 nm (2998
Date Acquired:	9/20/2022 11:57:48 AM CST		
Date Processed:	7/31/2023 7:41:11 PM CST		

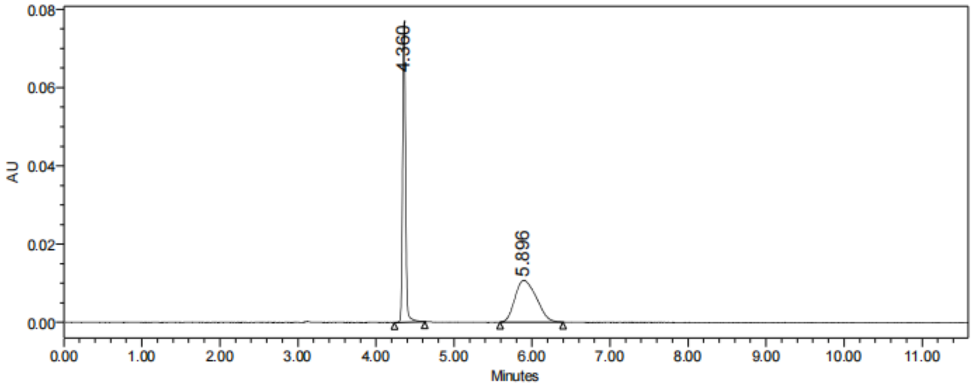

	RT	Area	% Area	Height
1	4.360	205900	50.24	76896
2	5.896	203927	49.76	10673

Asy-**7f**

SAMPLE INFORMATION			
Sample Name:	cxh-10-79-3-asy-IA-1%	Acquired By:	System
Sample Type:	Control	Sample Set Name:	
Vial:	39	Acq. Method Set:	1%
Injection #:	2	Processing Method:	7f asy
Injection Volume:	10.00 ul	Channel Name:	254.0nm
Run Time:	10.0 Minutes	Proc. Chnl. Descr.:	2998 PDA 254.0 nm (2998
Date Acquired:	9/20/2022 12:27:32 PM CST		
Date Processed:	7/31/2023 7:41:57 PM CST		

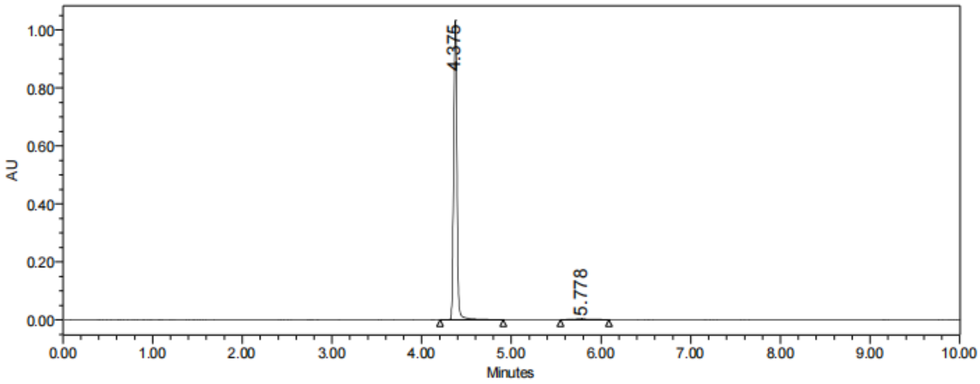

	RT	Area	% Area	Height
1	4.375	2637156	98.58	1032233
2	5.778	37988	1.42	2095

Rac-**7g**

SAMPLE INFORMATION

Sample Name:	cxh-10-79-1-rac-AD-5%	Acquired By:	System
Sample Type:	Unknown	Sample Set Name	
Vial:	85	Acq. Method Set:	5%210
Injection #:	1	Processing Method	7g rac
Injection Volume:	10.00 ul	Channel Name:	254.0nm
Run Time:	40.0 Minutes	Proc. Chnl. Descr.:	2998 PDA 254.0 nm (2998
Date Acquired:	9/19/2022 18:50:56 CST		
Date Processed:	7/31/2023 16:28:34 CST		

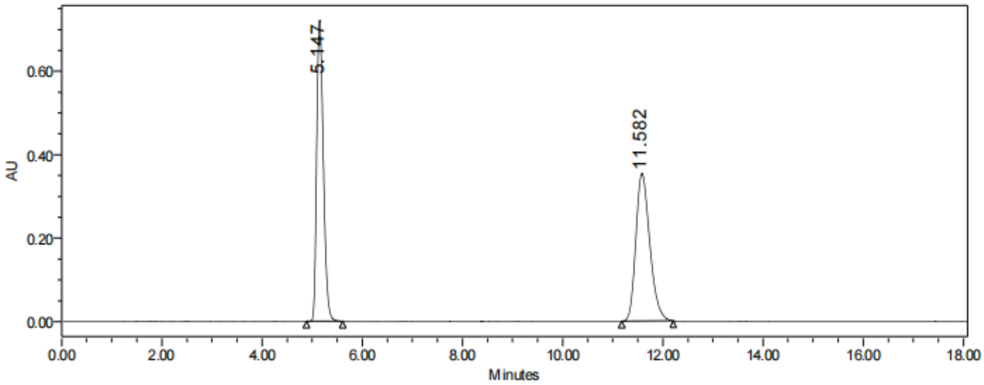

	RT	Area	% Area	Height
1	5.147	6729094	49.33	720848
2	11.582	6910565	50.67	354108

Asy-**7g**

SAMPLE INFORMATION

Sample Name:	cxh-10-79-1-asy-AD-5%	Acquired By:	System
Sample Type:	Unknown	Sample Set Name:	09192
Vial:	41	Acq. Method Set:	5%210
Injection #:	1	Processing Method	7g asy
Injection Volume:	10.00 ul	Channel Name:	260.0nm
Run Time:	15.0 Minutes	Proc. Chnl. Descr.:	2998 PDA 260.0 nm (2998
Date Acquired:	9/19/2022 21:36:46 CST		
Date Processed:	7/31/2023 16:32:09 CST		

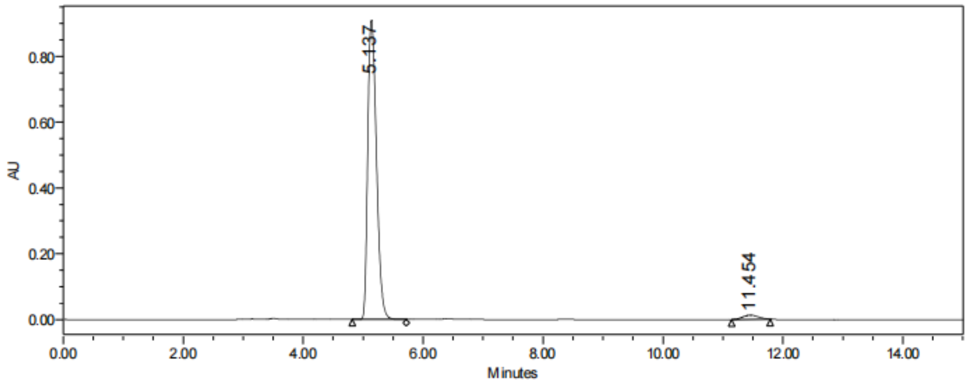

	RT	Area	% Area	Height
1	5.137	9245658	97.53	907738
2	11.454	234175	2.47	13095

Rac-**7h**

SAMPLE INFORMATION

Sample Name:	cxh-10-79-5-rac-AD-1%	Acquired By:	System
Sample Type:	Unknown	Sample Set Name:	0919
Vial:	14	Acq. Method Set:	1%
Injection #:	1	Processing Method	7h rac
Injection Volume:	10.00 ul	Channel Name:	254.0nm
Run Time:	20.0 Minutes	Proc. Chnl. Descr.:	2998 PDA 254.0 nm (2998
Date Acquired:	9/19/2022 19:59:44 CST		
Date Processed:	7/31/2023 16:44:24 CST		

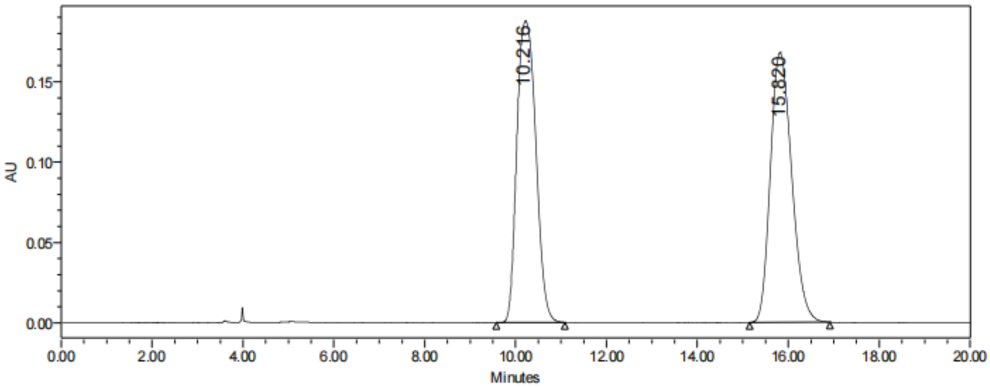

	RT	Area	% Area	Height
1	10.216	5456422	50.07	187412
2	15.820	5440851	49.93	167805

Asy-**7h**

SAMPLE INFORMATION

Sample Name:	cxh-10-79-5-asy-AD-1%	Acquired By:	System
Sample Type:	Unknown	Sample Set Name:	09193
Vial:	43	Acq. Method Set:	1%
Injection #:	1	Processing Method	7h asy
Injection Volume:	10.00 ul	Channel Name:	254.0nm
Run Time:	20.0 Minutes	Proc. Chnl. Descr.:	2998 PDA 254.0 nm (2998
Date Acquired:	9/19/2022 22:51:55 CST		
Date Processed:	7/31/2023 16:45:14 CST		

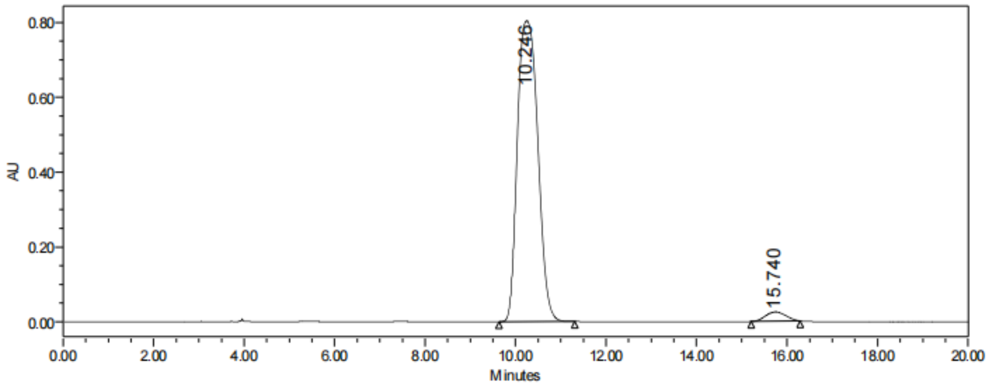

	RT	Area	% Area	Height
1	10.246	25344290	97.05	803608
2	15.740	770201	2.95	24461

Rac-**7i**

SAMPLE INFORMATION

Sample Name:	cxh-10-80-1-rac-AD-1%	Acquired By:	System
Sample Type:	Unknown	Sample Set Name:	0919
Vial:	15	Acq. Method Set:	1%
Injection #:	1	Processing Method	7i rac
Injection Volume:	10.00 ul	Channel Name:	254.0nm
Run Time:	20.0 Minutes	Proc. Chnl. Descr.:	2998 PDA 254.0 nm (2998
Date Acquired:	9/19/2022 20:20:25 CST		
Date Processed:	7/31/2023 16:48:28 CST		

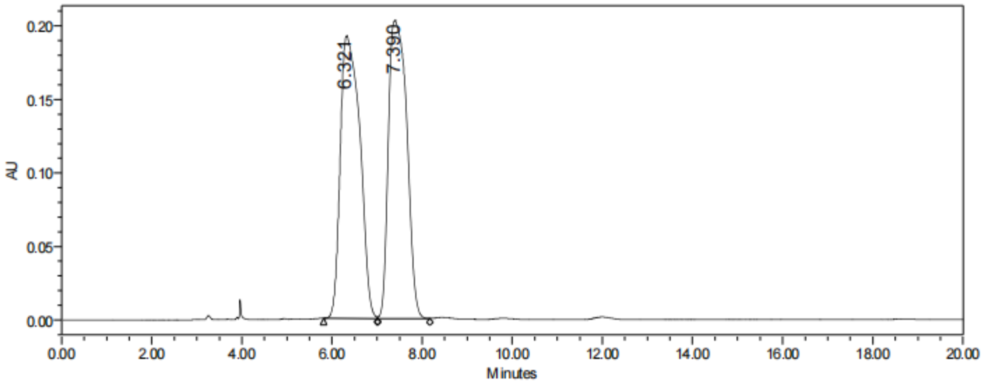

	RT	Area	% Area	Height
1	6.321	5772143	50.29	192219
2	7.390	5705095	49.71	202762

Asy-**7i**

SAMPLE INFORMATION

Sample Name:	cxh-10-80-1-asy-AD-1%	Acquired By:	System
Sample Type:	Unknown	Sample Set Name:	09193
Vial:	44	Acq. Method Set:	1%
Injection #:	1	Processing Method	7i asy
Injection Volume:	10.00 ul	Channel Name:	254.0nm@1
Run Time:	12.0 Minutes	Proc. Chnl. Descr.:	2998 PDA 254.0 nm (2998
Date Acquired:	9/19/2022 23:12:38 CST		
Date Processed:	7/31/2023 16:51:15 CST		

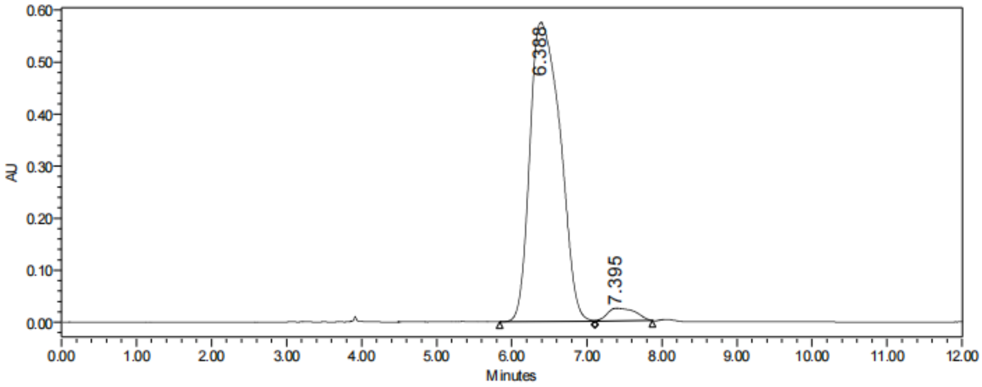

	RT	Area	% Area	Height
1	6.388	16112609	96.19	574387
2	7.395	638691	3.81	24291

Rac-**7j**

SAMPLE INFORMATION

Sample Name:	cxh-10-80-2-rac-AD-1%	Acquired By:	System
Sample Type:	Unknown	Sample Set Name:	0919
Vial:	16	Acq. Method Set:	1%
Injection #:	1	Processing Method	7j rac
Injection Volume:	10.00 ul	Channel Name:	254.0nm
Run Time:	20.0 Minutes	Proc. Chnl. Descr.:	2998 PDA 254.0 nm (2998
Date Acquired:	9/19/2022 20:41:08 CST		
Date Processed:	7/31/2023 16:52:27 CST		

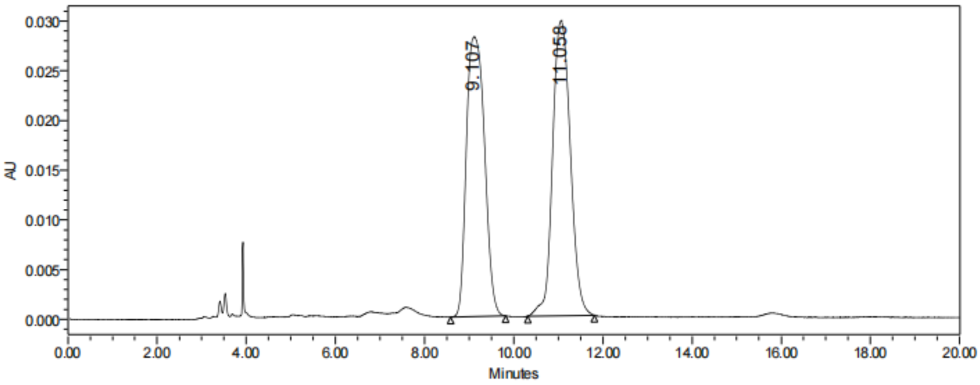

	RT	Area	% Area	Height
1	9.107	797852	49.27	28135
2	11.058	821441	50.73	29664

Asy-**7j**

SAMPLE INFORMATION

Sample Name:	cxh-10-80-2-asy-AD-1%	Acquired By:	System
Sample Type:	Unknown	Sample Set Name:	09193
Vial:	45	Acq. Method Set:	1%
Injection #:	1	Processing Method	7j asy
Injection Volume:	10.00 ul	Channel Name:	254.0nm
Run Time:	16.0 Minutes	Proc. Chnl. Descr.:	2998 PDA 254.0 nm (2998
Date Acquired:	9/19/2022 23:25:20 CST		
Date Processed:	7/31/2023 16:52:03 CST		

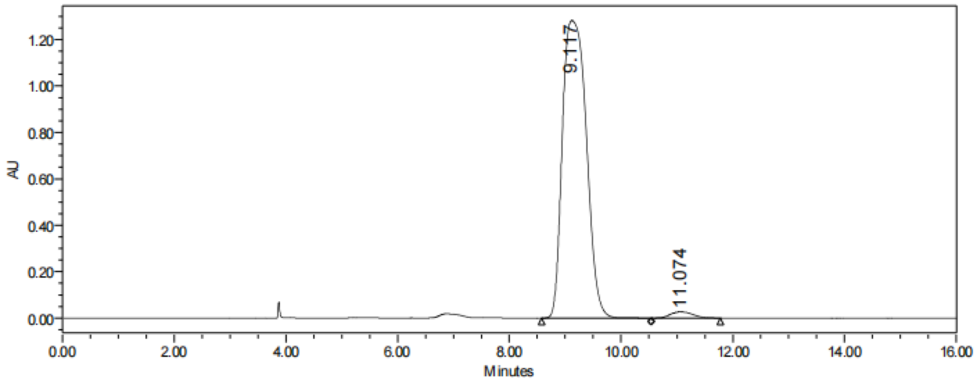

	RT	Area	% Area	Height
1	9.117	38379566	98.10	1280831
2	11.074	743075	1.90	26305

Rac-**7k**

SAMPLE INFORMATION

Sample Name:	cxh-10-53-3-rac-AD-2%	Acquired By:	System
Sample Type:	Control	Sample Set Name:	
Vial:	71	Acq. Method Set:	2%
Injection #:	2	Processing Method:	7k rac
Injection Volume:	5.00 ul	Channel Name:	254.0nm
Run Time:	60.0 Minutes	Proc. Chnl. Descr.:	2998 PDA 254.0 nm (2998
Date Acquired:	6/23/2022 11:00:04 AM CST		
Date Processed:	7/31/2023 8:29:07 PM CST		

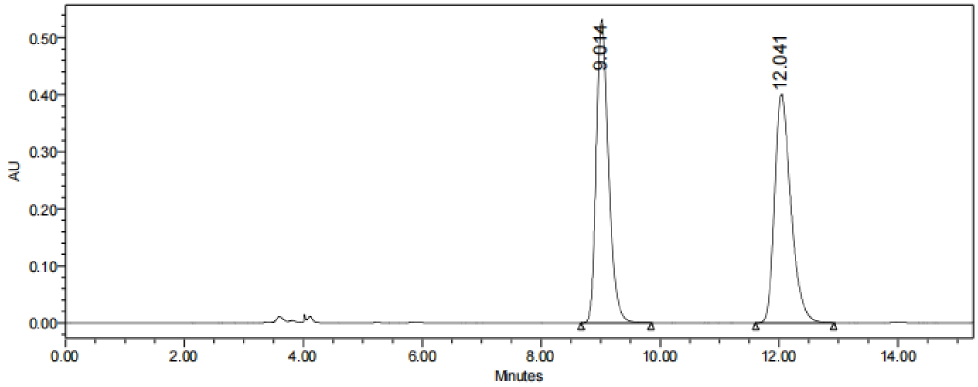

	RT	Area	% Area	Height
1	9.014	7603492	49.88	531079
2	12.041	7641601	50.12	399996

Asy-**7k**

SAMPLE INFORMATION

Sample Name:	cxh-10-72-2-AD-2%	Acquired By:	System
Sample Type:	Unknown	Sample Set Name:	0822
Vial:	6	Acq. Method Set:	2%
Injection #:	1	Processing Method	7k rac
Injection Volume:	10.00 ul	Channel Name:	254.0nm
Run Time:	14.0 Minutes	Proc. Chnl. Descr.:	2998 PDA 254.0 nm (2998
Date Acquired:	8/22/2022 21:35:28 CST		
Date Processed:	7/31/2023 20:17:31 CST		

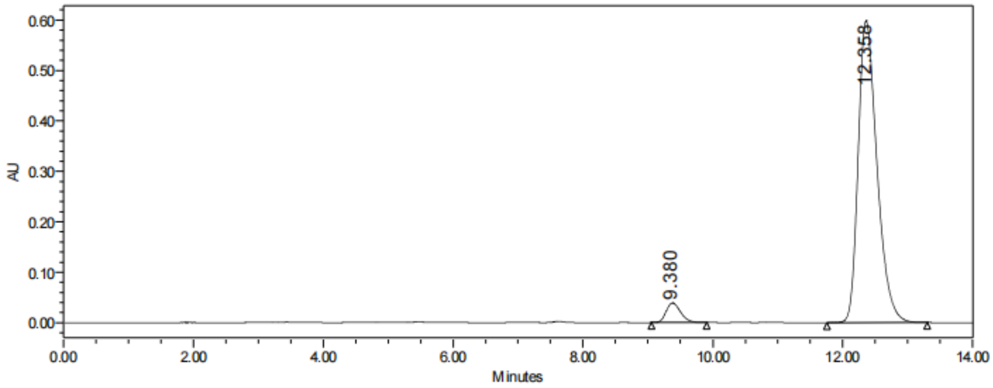

	RT	Area	% Area	Height
1	9.380	607056	4.77	39059
2	12.358	12127324	95.23	598456

Rac-**7l**

SAMPLE INFORMATION

Sample Name:	cxh-10-72-5-AD-rac-2%	Acquired By:	System
Sample Type:	Control	Sample Set Name:	
Vial:	1	Acq. Method Set:	2%
Injection #:	1	Processing Method:	7l rac
Injection Volume:	10.00 ul	Channel Name:	254.0nm
Run Time:	50.0 Minutes	Proc. Chnl. Descr.:	2998 PDA 254.0 nm (2998
Date Acquired:	8/22/2022 10:33:32 AM CST		
Date Processed:	7/31/2023 7:45:05 PM CST		

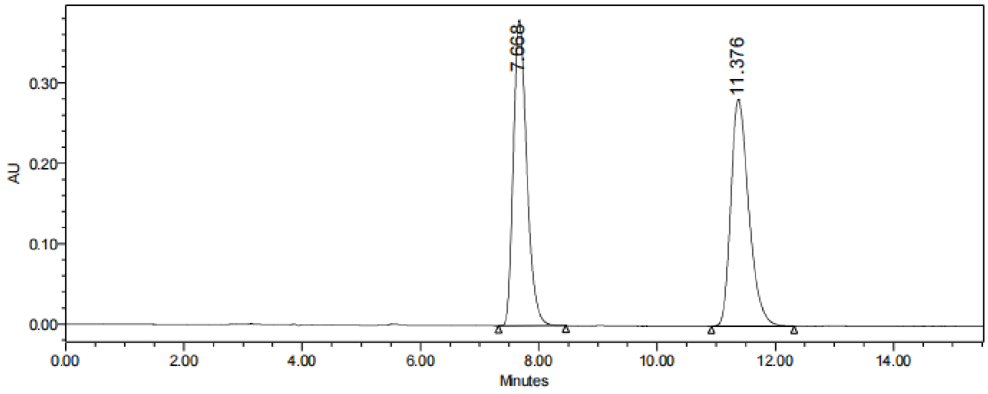

	RT	Area	% Area	Height
1	7.668	5920155	49.95	379664
2	11.376	5932844	50.05	281503

Asy-**7l**

SAMPLE INFORMATION

Sample Name:	cxh-10-72-7-AD-2%	Acquired By:	System
Sample Type:	Unknown	Sample Set Name:	0822
Vial:	50	Acq. Method Set:	2%
Injection #:	1	Processing Method:	7l asy
Injection Volume:	10.00 ul	Channel Name:	254.0nm
Run Time:	15.0 Minutes	Proc. Chnl. Descr.:	2998 PDA 254.0 nm (2998
Date Acquired:	8/22/2022 11:28:16 AM CST		
Date Processed:	7/31/2023 7:47:45 PM CST		

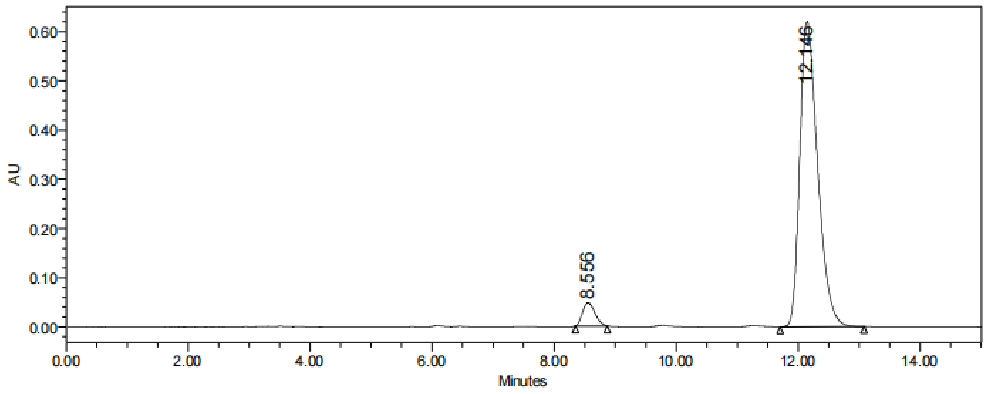

	RT	Area	% Area	Height
1	8.556	663307	5.00	47123
2	12.146	12593091	95.00	619704

Rac-**7m**

SAMPLE INFORMATION

Sample Name:	cxh-11-8-1-rac-AD-1%	Acquired By:	System
Sample Type:	Control	Sample Set Name:	
Vial:	118	Acq. Method Set:	1%
Injection #:	1	Processing Method:	7m rac
Injection Volume:	10.00 ul	Channel Name:	254.0nm
Run Time:	40.0 Minutes	Proc. Chnl. Descr.:	2998 PDA 254.0 nm (2998
Date Acquired:	2/1/2023 1:42:09 PM CST		
Date Processed:	7/31/2023 8:09:43 PM CST		

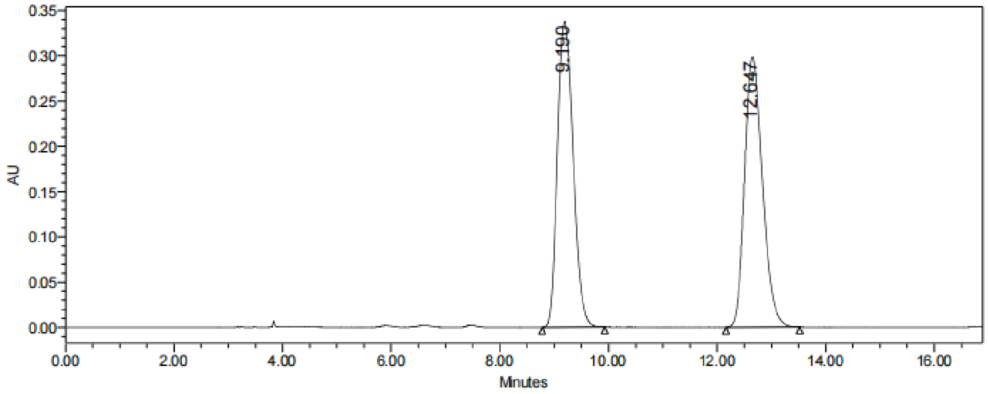

	RT	Area	% Area	Height
1	9.190	6840323	49.93	337162
2	12.647	6859455	50.07	298263

Asy-**7m**

SAMPLE INFORMATION

Sample Name:	cxh-11-10-3-asy-AD-1%	Acquired By:	System
Sample Type:	Unknown	Sample Set Name:	0207
Vial:	45	Acq. Method Set:	1%
Injection #:	1	Processing Method:	7m asy
Injection Volume:	10.00 ul	Channel Name:	254.0nm
Run Time:	17.0 Minutes	Proc. Chnl. Descr.:	2998 PDA 254.0 nm (2998
Date Acquired:	2/7/2023 1:21:27 PM CST		
Date Processed:	7/31/2023 8:07:51 PM CST		

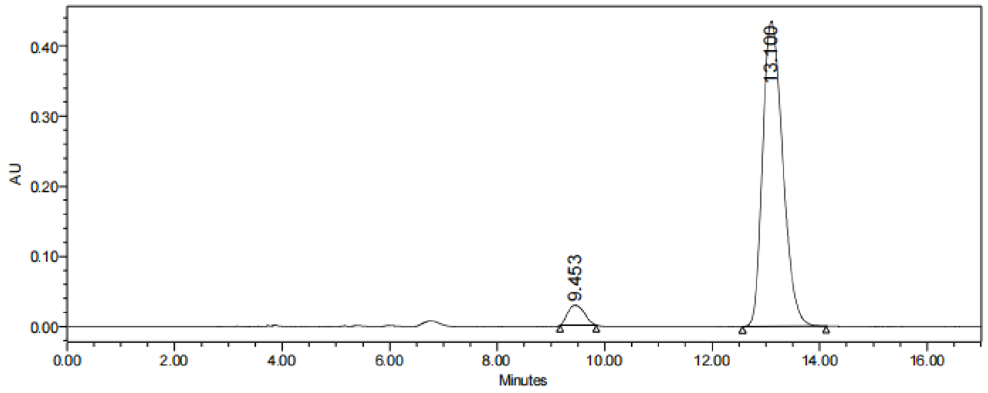

	RT	Area	% Area	Height
1	9.453	610707	5.11	28092
2	13.100	11349897	94.89	434846

Rac-**7n**

SAMPLE INFORMATION

Sample Name:	cxh-10-82-3-rac-AD-1%	Acquired By:	System
Sample Type:	Unknown	Sample Set Name:	
Vial:	36	Acq. Method Set:	1%
Injection #:	1	Processing Method	7n rac
Injection Volume:	10.00 ul	Channel Name:	254.0nm
Run Time:	40.0 Minutes	Proc. Chnl. Descr.:	2998 PDA 254.0 nm (2998
Date Acquired:	9/27/2022 12:12:02 CST		
Date Processed:	7/31/2023 17:25:10 CST		

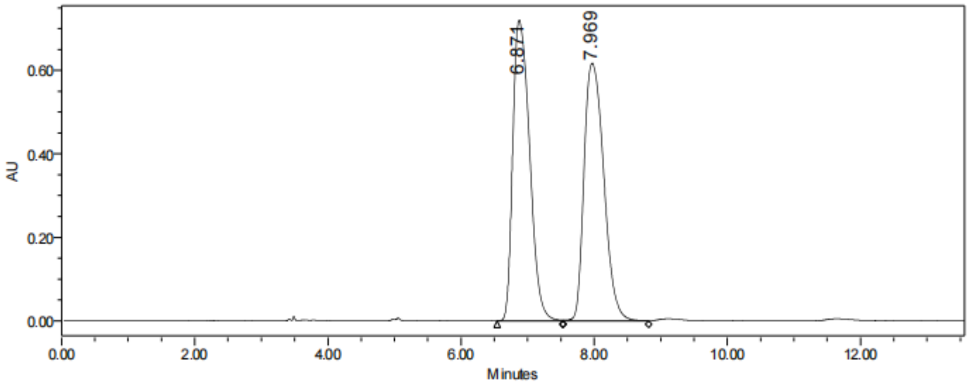

	RT	Area	% Area	Height
1	6.871	12683825	49.80	719144
2	7.969	12787484	50.20	616797

Asy-**7n**

SAMPLE INFORMATION

Sample Name:	cxh-10-82-3-asy-AD-1%	Acquired By:	System
Sample Type:	Unknown	Sample Set Name:	0927
Vial:	102	Acq. Method Set:	1%
Injection #:	1	Processing Method	7n asy
Injection Volume:	10.00 ul	Channel Name:	254.0nm
Run Time:	12.0 Minutes	Proc. Chnl. Descr.:	2998 PDA 254.0 nm (2998
Date Acquired:	9/27/2022 13:46:44 CST		
Date Processed:	7/31/2023 17:25:57 CST		

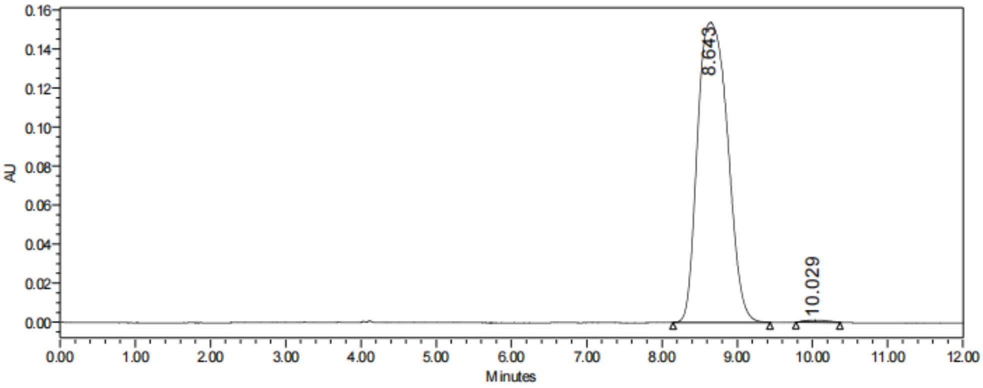

	RT	Area	% Area	Height
1	8.643	4272553	99.47	153452
2	10.029	22779	0.53	1071

Rac-**7o**

SAMPLE INFORMATION

Sample Name:	cxh-10-84-2-rac-AD-1%	Acquired By:	System
Sample Type:	Control	Sample Set Name:	
Vial:	82	Acq. Method Set:	1%
Injection #:	1	Processing Method:	7o rac
Injection Volume:	10.00 ul	Channel Name:	254.0nm
Run Time:	30.0 Minutes	Proc. Chnl. Descr.:	2998 PDA 254.0 nm (2998
Date Acquired:	10/8/2022 12:36:09 PM CST		
Date Processed:	7/31/2023 7:57:38 PM CST		

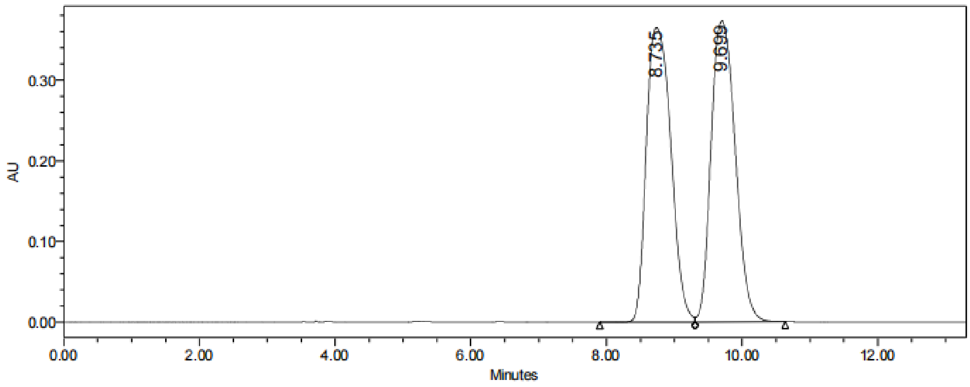

	RT	Area	% Area	Height
1	8.735	9165734	49.89	364749
2	9.699	9207038	50.11	372892

Asy-**7o**

SAMPLE INFORMATION

Sample Name:	cxh-10-84-2-asy-AD-1%	Acquired By:	System
Sample Type:	Unknown	Sample Set Name:	1008
Vial:	81	Acq. Method Set:	1%
Injection #:	1	Processing Method:	7o asy
Injection Volume:	10.00 ul	Channel Name:	254.0nm
Run Time:	15.0 Minutes	Proc. Chnl. Descr.:	2998 PDA 254.0 nm (2998
Date Acquired:	10/8/2022 12:50:36 PM CST		
Date Processed:	7/31/2023 7:57:51 PM CST		

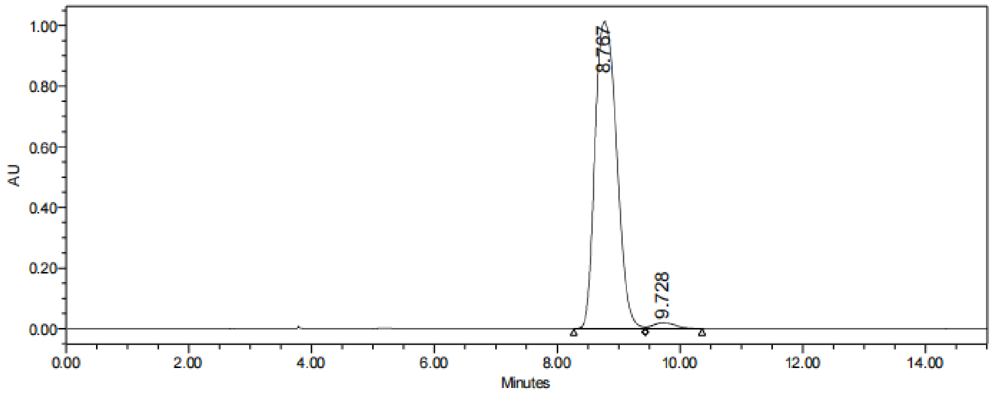

	RT	Area	% Area	Height
1	8.767	24741878	98.12	1012754
2	9.728	474995	1.88	19171

Rac-**7p**

SAMPLE INFORMATION

Sample Name:	cxh-10-83-1-rac-AD-1%	Acquired By:	System
Sample Type:	Unknown	Sample Set Name:	
Vial:	20	Acq. Method Set:	1%
Injection #:	1	Processing Method	7p rac
Injection Volume:	10.00 ul	Channel Name:	254.0nm
Run Time:	40.0 Minutes	Proc. Chnl. Descr.:	2998 PDA 254.0 nm (2998
Date Acquired:	9/30/2022 11:00:36 CST		
Date Processed:	7/31/2023 17:36:52 CST		

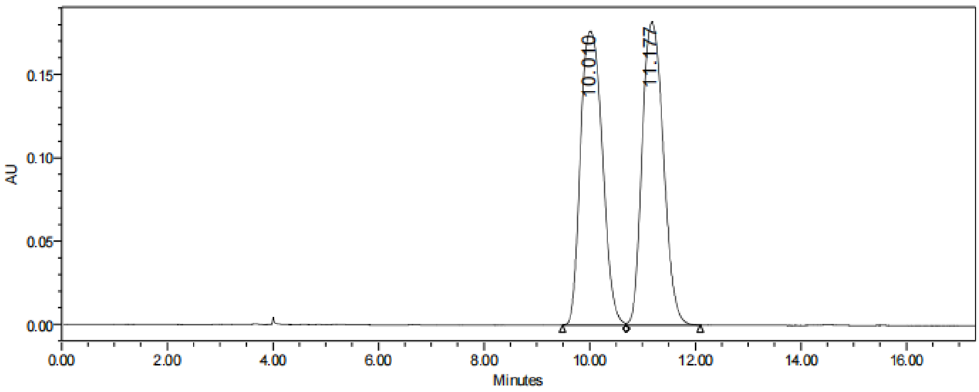

	RT	Area	% Area	Height
1	10.010	5124951	49.99	176425
2	11.177	5126344	50.01	181941

Asy-**7p**

SAMPLE INFORMATION

Sample Name:	cxh-10-83-1-asy-AD-1%	Acquired By:	System
Sample Type:	Unknown	Sample Set Name:	0930
Vial:	41	Acq. Method Set:	1%
Injection #:	1	Processing Method	7p asy
Injection Volume:	10.00 ul	Channel Name:	254.0nm
Run Time:	15.0 Minutes	Proc. Chnl. Descr.:	2998 PDA 254.0 nm (2998
Date Acquired:	9/30/2022 11:51:38 CST		
Date Processed:	7/31/2023 17:37:08 CST		

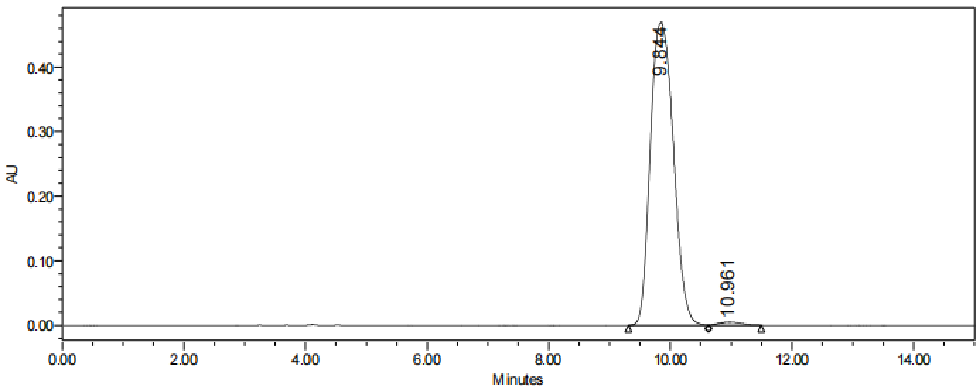

	RT	Area	% Area	Height
1	9.844	12360434	99.01	468719
2	10.961	123196	0.99	4784

Rac-**7q**

SAMPLE INFORMATION

Sample Name:	cxh-10-82-1-rac-AD-1%	Acquired By:	System
Sample Type:	Unknown	Sample Set Name:	
Vial:	3	Acq. Method Set:	1%
Injection #:	1	Processing Method	7q rac
Injection Volume:	10.00 ul	Channel Name:	254.0nm
Run Time:	40.0 Minutes	Proc. Chnl. Descr.:	2998 PDA 254.0 nm (2998
Date Acquired:	9/27/2022 11:57:53 CST		
Date Processed:	7/31/2023 17:16:50 CST		

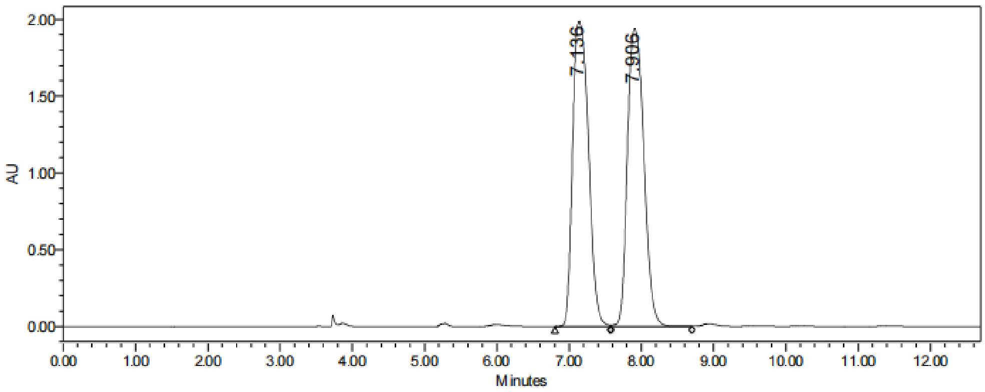

	RT	Area	% Area	Height
1	7.136	30258959	49.74	1985705
2	7.906	30575524	50.26	1940087

Asy-**7q**

SAMPLE INFORMATION

Sample Name:	cxh-10-82-1-asy-AD-1%	Acquired By:	System
Sample Type:	Unknown	Sample Set Name:	0927
Vial:	101	Acq. Method Set:	1%
Injection #:	1	Processing Method	7q asy
Injection Volume:	10.00 ul	Channel Name:	254.0nm
Run Time:	12.0 Minutes	Proc. Chnl. Descr.:	2998 PDA 254.0 nm (2998
Date Acquired:	9/27/2022 13:34:02 CST		
Date Processed:	7/31/2023 17:16:32 CST		

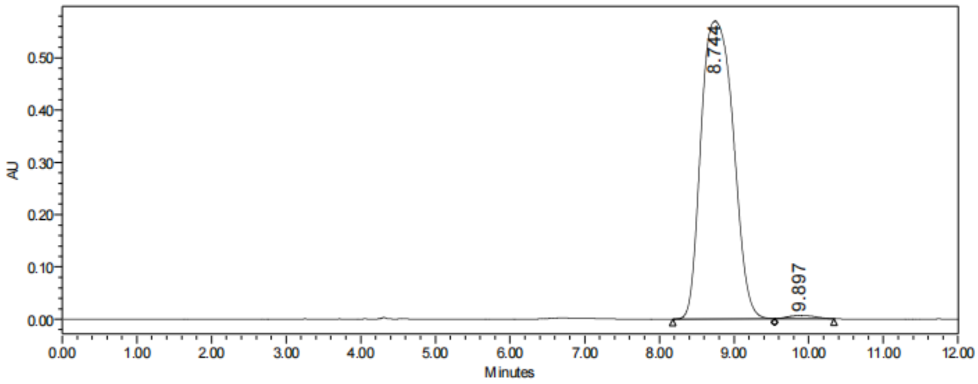

	RT	Area	% Area	Height
1	8.744	17103959	99.03	569661
2	9.897	167489	0.97	6482

Rac-**7r**

SAMPLE INFORMATION

Sample Name:	cxh-10-82-4-rac-AD-3%	Acquired By:	System
Sample Type:	Unknown	Sample Set Name:	
Vial:	90	Acq. Method Set:	3%210
Injection #:	2	Processing Method	7r rac
Injection Volume:	10.00 ul	Channel Name:	254.0nm
Run Time:	40.0 Minutes	Proc. Chnl. Descr.:	2998 PDA 254.0 nm (2998
Date Acquired:	9/27/2022 13:14:56 CST		
Date Processed:	7/31/2023 17:28:28 CST		

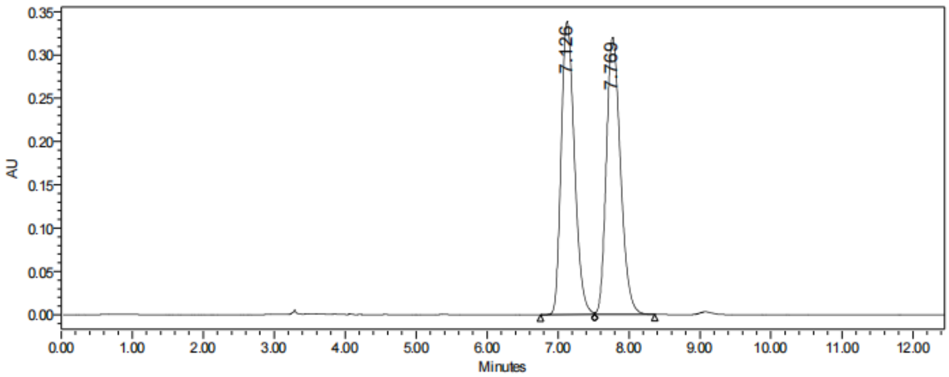

	RT	Area	% Area	Height
1	7.126	4416188	50.01	338360
2	7.769	4415155	49.99	319669

Asy-**7r**

SAMPLE INFORMATION

Sample Name:	cxh-10-82-4-asy-AD-3%	Acquired By:	System
Sample Type:	Unknown	Sample Set Name:	0927
Vial:	105	Acq. Method Set:	3%210
Injection #:	1	Processing Method	7r asy
Injection Volume:	10.00 ul	Channel Name:	254.0nm
Run Time:	12.0 Minutes	Proc. Chnl. Descr.:	2998 PDA 254.0 nm (2998
Date Acquired:	9/27/2022 14:35:55 CST		
Date Processed:	7/31/2023 17:29:37 CST		

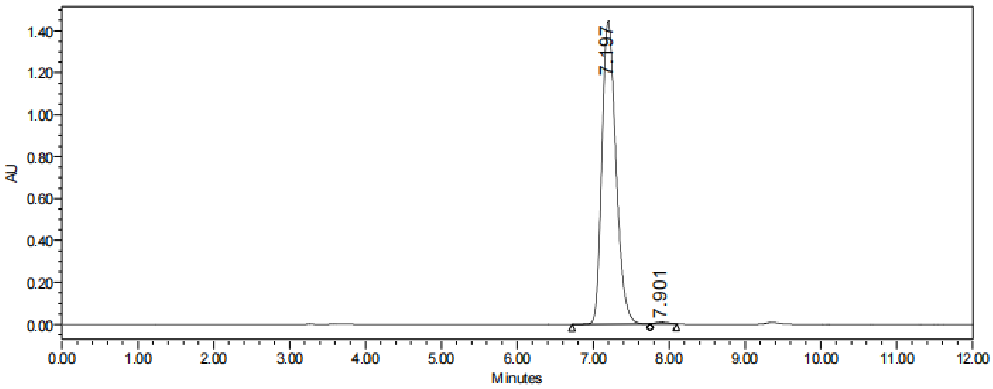

	RT	Area	% Area	Height
1	7.197	18527017	99.48	1442317
2	7.901	97385	0.52	8428

Rac-**7s**

SAMPLE INFORMATION

Sample Name:	cxh-10-82-5-rac-AD-1%	Acquired By:	System
Sample Type:	Unknown	Sample Set Name:	
Vial:	61	Acq. Method Set:	1%
Injection #:	1	Processing Method	7s rac
Injection Volume:	10.00 ul	Channel Name:	254.0nm
Run Time:	40.0 Minutes	Proc. Chnl. Descr.:	2998 PDA 254.0 nm (2998
Date Acquired:	9/27/2022 12:26:53 CST		
Date Processed:	7/31/2023 17:31:20 CST		

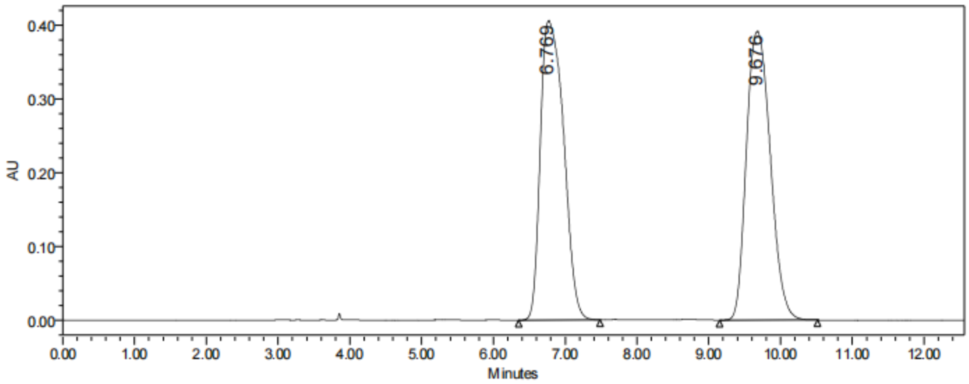

	RT	Area	% Area	Height
1	6.769	9092795	49.89	405537
2	9.676	9131578	50.11	391278

Asy-**7s**

SAMPLE INFORMATION

Sample Name:	cxh-10-82-5-asy-AD-1%	Acquired By:	System
Sample Type:	Unknown	Sample Set Name:	0927
Vial:	103	Acq. Method Set:	1%
Injection #:	1	Processing Method	7s asy
Injection Volume:	10.00 ul	Channel Name:	254.0nm
Run Time:	14.0 Minutes	Proc. Chnl. Descr.:	2998 PDA 254.0 nm (2998
Date Acquired:	9/27/2022 13:59:24 CST		
Date Processed:	7/31/2023 17:32:10 CST		

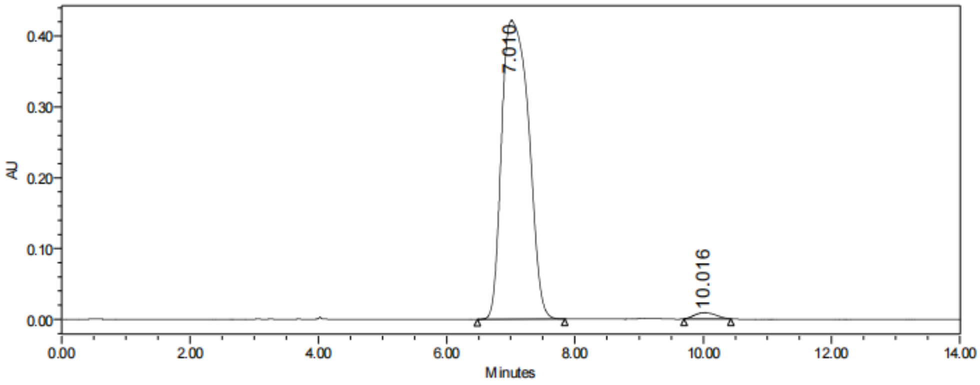

	RT	Area	% Area	Height
1	7.010	12303973	98.41	421627
2	10.016	198702	1.59	8566

Rac-**7t**

SAMPLE INFORMATION

Sample Name:	cxh-10-82-6-rac-AD-1%	Acquired By:	System
Sample Type:	Unknown	Sample Set Name:	
Vial:	62	Acq. Method Set:	1%
Injection #:	1	Processing Method	7t rac
Injection Volume:	10.00 ul	Channel Name:	254.0nm
Run Time:	40.0 Minutes	Proc. Chnl. Descr.:	2998 PDA 254.0 nm (2998
Date Acquired:	9/27/2022 12:40:08 CST		
Date Processed:	7/31/2023 17:33:39 CST		

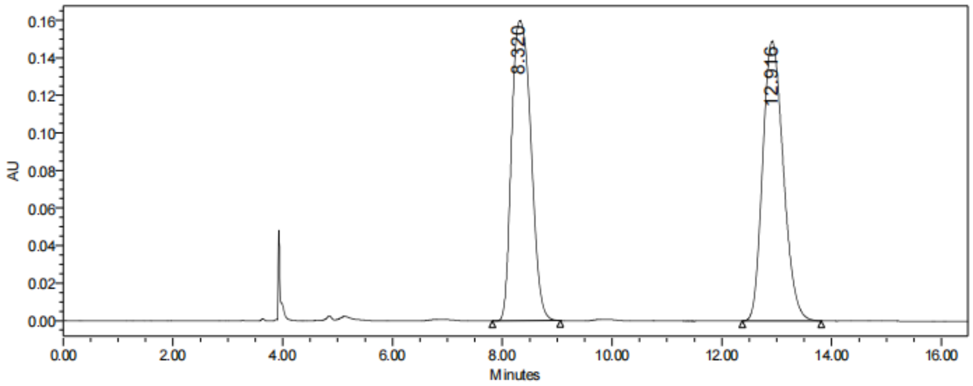

	RT	Area	% Area	Height
1	8.320	3899691	50.04	159774
2	12.916	3894171	49.96	148937

Asy-**7t**

SAMPLE INFORMATION

Sample Name:	cxh-10-82-6-asy-AD-1%	Acquired By:	System
Sample Type:	Unknown	Sample Set Name:	0927
Vial:	104	Acq. Method Set:	1%
Injection #:	1	Processing Method	7t asy
Injection Volume:	10.00 ul	Channel Name:	254.0nm
Run Time:	18.0 Minutes	Proc. Chnl. Descr.:	2998 PDA 254.0 nm (2998
Date Acquired:	9/27/2022 14:14:07 CST		
Date Processed:	7/31/2023 17:33:55 CST		

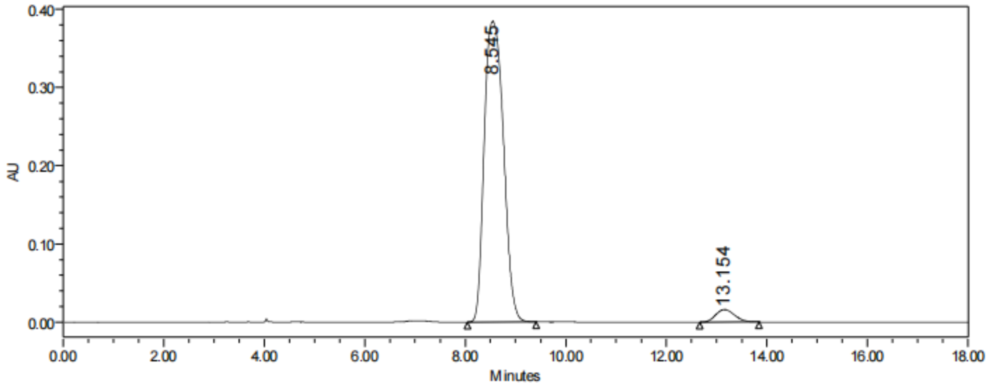

	RT	Area	% Area	Height
1	8.545	10233646	96.05	384147
2	13.154	420516	3.95	15901

Rac-**7u**

SAMPLE INFORMATION

Sample Name:	cxh-10-83-2-rac-AD-1%	Acquired By:	System
Sample Type:	Unknown	Sample Set Name:	
Vial:	21	Acq. Method Set:	1%
Injection #:	1	Processing Method	7u rac
Injection Volume:	10.00 ul	Channel Name:	254.0nm
Run Time:	40.0 Minutes	Proc. Chnl. Descr.:	2998 PDA 254.0 nm (2998
Date Acquired:	9/30/2022 11:18:38 CST		
Date Processed:	7/31/2023 17:38:45 CST		

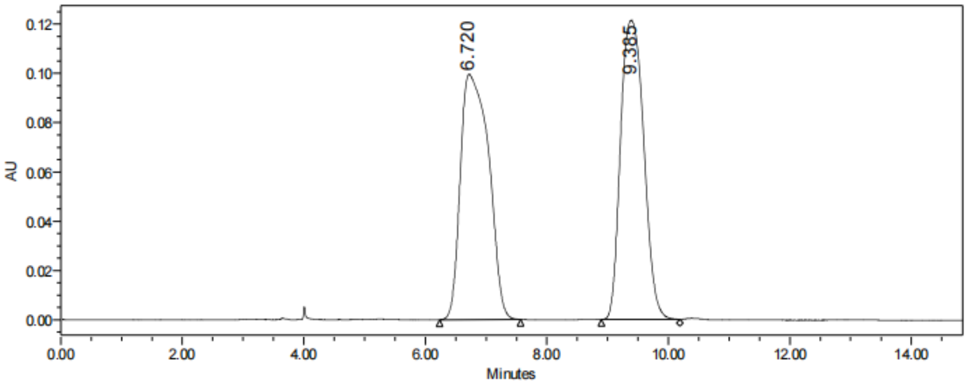

	RT	Area	% Area	Height
1	6.720	3236868	50.01	99493
2	9.385	3235432	49.99	121265

Asy-**7u**

SAMPLE INFORMATION

Sample Name:	cxh-10-83-2-asy-AD-1%	Acquired By:	System
Sample Type:	Unknown	Sample Set Name:	0930
Vial:	42	Acq. Method Set:	1%
Injection #:	1	Processing Method	7u asy
Injection Volume:	10.00 ul	Channel Name:	254.0nm
Run Time:	15.0 Minutes	Proc. Chnl. Descr.:	2998 PDA 254.0 nm (2998
Date Acquired:	9/30/2022 12:07:19 CST		
Date Processed:	7/31/2023 17:39:16 CST		

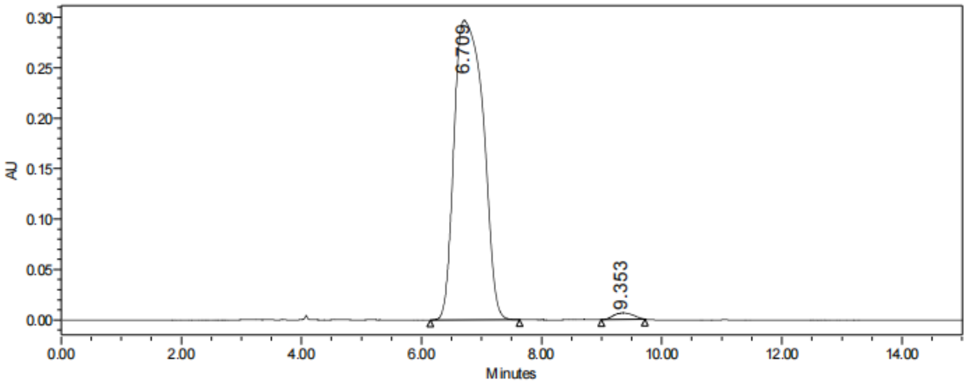

	RT	Area	% Area	Height
1	6.709	10016155	98.57	296659
2	9.353	145063	1.43	6282

Rac-**7v**

SAMPLE INFORMATION			
Sample Name:	cxh-10-83-3-RAC-AD-1%	Acquired By:	System
Sample Type:	Control	Sample Set Name:	
Vial:	58	Acq. Method Set:	1%
Injection #:	1	Processing Method:	7v rac
Injection Volume:	10.00 ul	Channel Name:	254.0nm
Run Time:	30.0 Minutes	Proc. Chnl. Descr.:	2998 PDA 254.0 nm (2998
Date Acquired:	10/6/2022 11:11:31 AM CST		
Date Processed:	7/31/2023 7:54:34 PM CST		

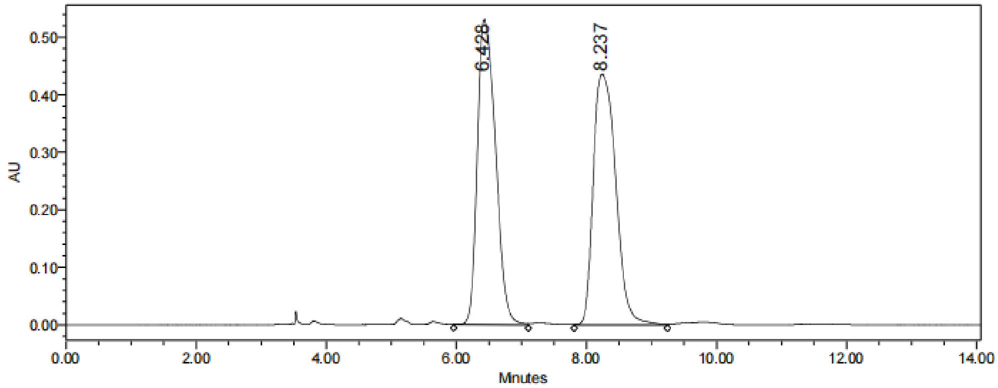

	RT	Area	% Area	Height
1	6.428	10364384	49.78	529539
2	8.237	10456945	50.22	435179

Asy-**7v**

SAMPLE INFORMATION			
Sample Name:	cxh-10-84-1-asy-AD-1%	Acquired By:	System
Sample Type:	Control	Sample Set Name:	
Vial:	65	Acq. Method Set:	1%
Injection #:	1	Processing Method:	7v asy
Injection Volume:	10.00 ul	Channel Name:	254.0nm
Run Time:	30.0 Minutes	Proc. Chnl. Descr.:	2998 PDA 254.0 nm (2998
Date Acquired:	10/8/2022 12:15:21 PM CST		
Date Processed:	7/31/2023 7:55:32 PM CST		

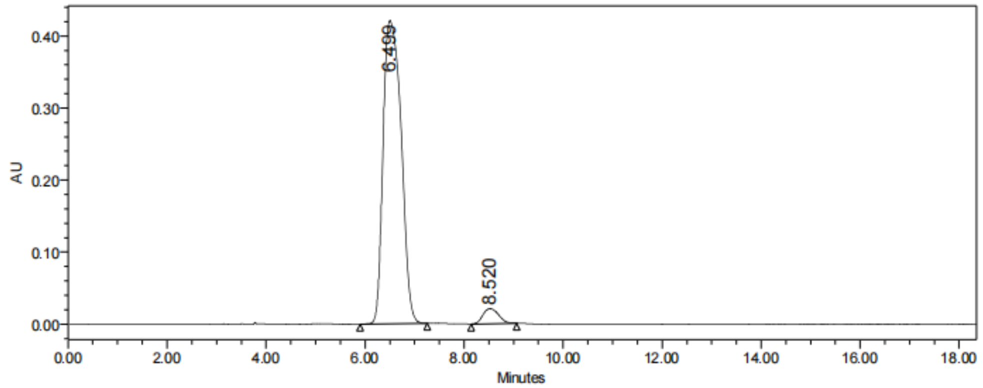

	RT	Area	% Area	Height
1	6.499	10685037	95.93	420606
2	8.520	453472	4.07	20655

Rac-**7w**

SAMPLE INFORMATION

Sample Name:	cxh-10-83-4-rac-AD-1%	Acquired By:	System
Sample Type:	Unknown	Sample Set Name	
Vial:	22	Acq. Method Set:	1%
Injection #:	1	Processing Method	7w rac
Injection Volume:	10.00 ul	Channel Name:	254.0nm
Run Time:	40.0 Minutes	Proc. Chnl. Descr.:	2998 PDA 254.0 nm (2998
Date Acquired:	9/30/2022 11:34:15 CST		
Date Processed:	7/31/2023 17:41:07 CST		

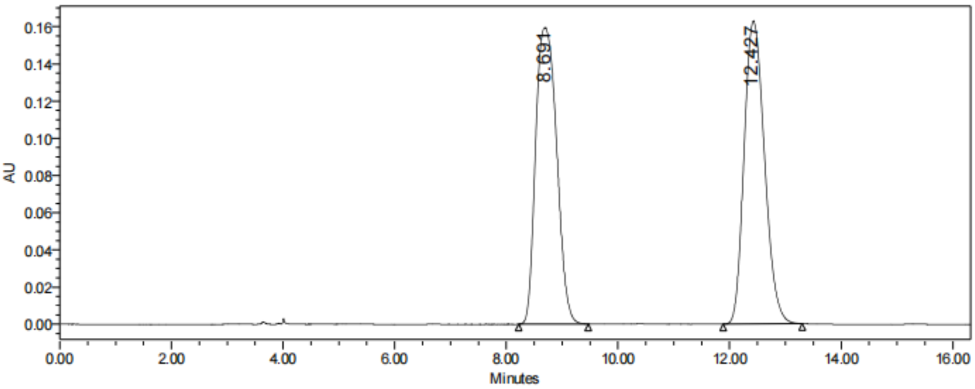

	RT	Area	% Area	Height
1	8.691	4179330	50.06	159411
2	12.427	4169685	49.94	162791

Asy-**7w**

SAMPLE INFORMATION

Sample Name:	cxh-10-83-4-asy-AD-1%	Acquired By:	System
Sample Type:	Unknown	Sample Set Name:	0930
Vial:	43	Acq. Method Set:	1%
Injection #:	1	Processing Method	7w asy
Injection Volume:	10.00 ul	Channel Name:	254.0nm
Run Time:	18.0 Minutes	Proc. Chnl. Descr.:	2998 PDA 254.0 nm (2998
Date Acquired:	9/30/2022 12:22:59 CST		
Date Processed:	7/31/2023 17:41:39 CST		

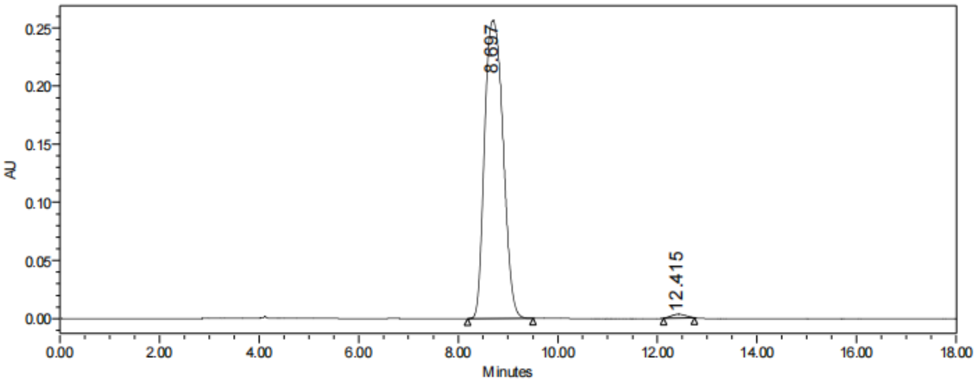

	RT	Area	% Area	Height
1	8.697	6603565	99.04	256265
2	12.415	64055	0.96	3118

Rac-**7x**

SAMPLE INFORMATION

Sample Name:	cxh-10-81-1-rac-AD-1%	Acquired By:	System
Sample Type:	Unknown	Sample Set Name:	
Vial:	66	Acq. Method Set:	1%
Injection #:	1	Processing Method	7x rac
Injection Volume:	10.00 ul	Channel Name:	270.0nm
Run Time:	40.0 Minutes	Proc. Chnl. Descr.:	2998 PDA 270.0 nm (2998
Date Acquired:	9/30/2022 15:50:12 CST		
Date Processed:	7/31/2023 17:03:13 CST		

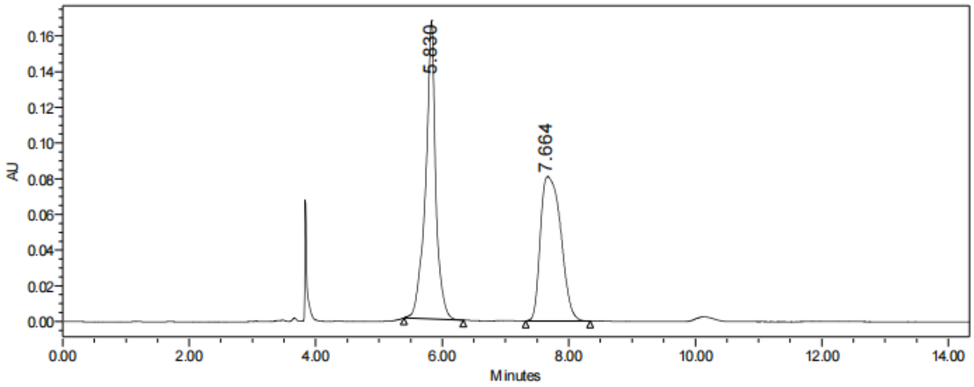

	RT	Area	% Area	Height
1	5.830	1876076	50.87	167057
2	7.664	1811853	49.13	80930

Asy-**7x**

SAMPLE INFORMATION

Sample Name:	cxh-10-81-1-asy-AD-1%	Acquired By:	System
Sample Type:	Unknown	Sample Set Name:	09301
Vial:	10	Acq. Method Set:	1%
Injection #:	1	Processing Method	7x asy
Injection Volume:	10.00 ul	Channel Name:	270.0nm
Run Time:	12.0 Minutes	Proc. Chnl. Descr.:	2998 PDA 270.0 nm (2998
Date Acquired:	9/30/2022 16:54:19 CST		
Date Processed:	7/31/2023 17:04:17 CST		

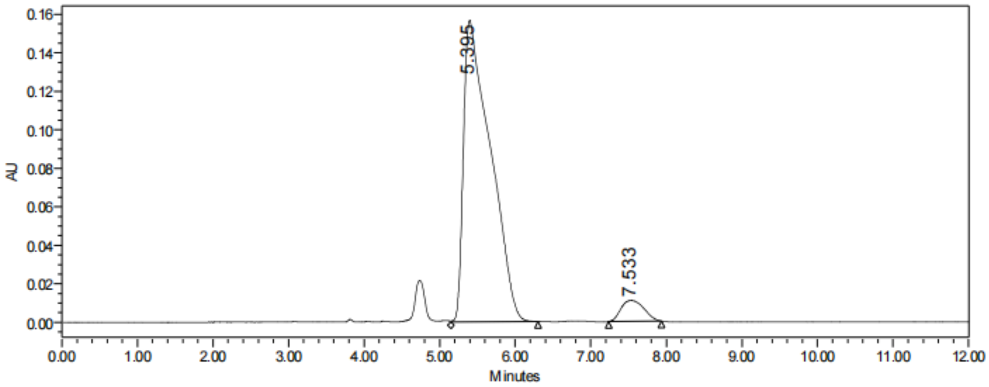

	RT	Area	% Area	Height
1	5.395	3885360	94.44	156199
2	7.533	228544	5.56	10827

Rac-**7y**

SAMPLE INFORMATION

Sample Name:	cxh-10-81-4-rac-AD-1%	Acquired By:	System
Sample Type:	Unknown	Sample Set Name:	
Vial:	92	Acq. Method Set:	1%
Injection #:	1	Processing Method	7y rac
Injection Volume:	10.00 ul	Channel Name:	254.0nm@1
Run Time:	40.0 Minutes	Proc. Chnl. Descr.:	2998 PDA 254.0 nm (2998
Date Acquired:	9/30/2022 16:05:46 CST		
Date Processed:	7/31/2023 17:09:44 CST		

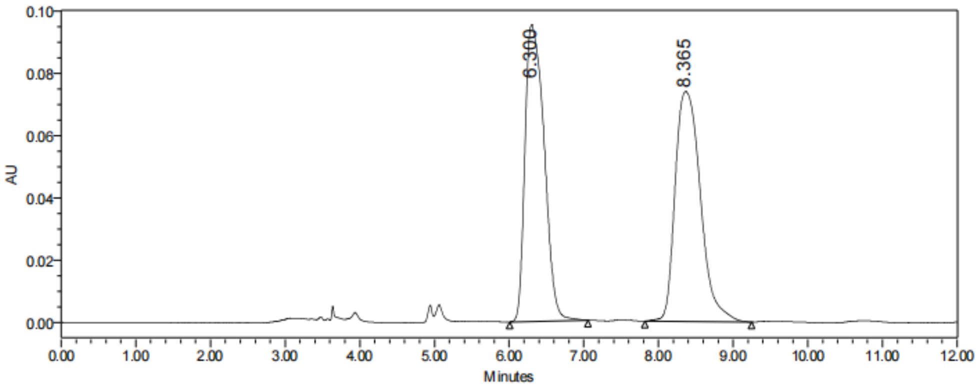

	RT	Area	% Area	Height
1	6.300	1704551	49.31	95198
2	8.365	1752326	50.69	73873

Asy-**7y**

SAMPLE INFORMATION

Sample Name:	cxh-10-81-4-asy-AD-1%	Acquired By:	System
Sample Type:	Unknown	Sample Set Name:	09301
Vial:	11	Acq. Method Set:	1%
Injection #:	1	Processing Method	7y asy
Injection Volume:	10.00 ul	Channel Name:	254.0nm
Run Time:	12.0 Minutes	Proc. Chnl. Descr.:	2998 PDA 254.0 nm (2998
Date Acquired:	9/30/2022 17:07:03 CST		
Date Processed:	7/31/2023 17:09:28 CST		

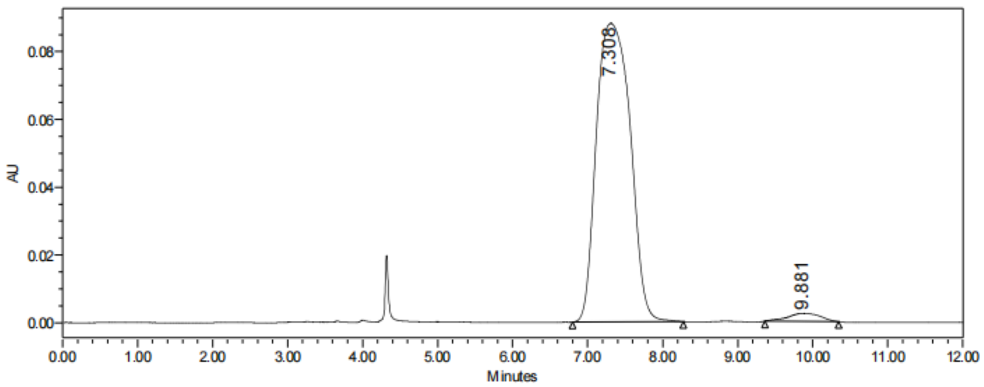

	RT	Area	% Area	Height
1	7.308	2770590	97.46	88106
2	9.881	72169	2.54	2355

Rac-**7z**

SAMPLE INFORMATION			
Sample Name:	cxh-10-81-5-rac-AD-1%	Acquired By:	System
Sample Type:	Unknown	Sample Set Name	09301
Vial:	112	Acq. Method Set:	1%
Injection #:	1	Processing Method	7z rac
Injection Volume:	10.00 ul	Channel Name:	254.0nm
Run Time:	12.0 Minutes	Proc. Chnl. Descr.:	2998 PDA 254.0 nm (2998
Date Acquired:	9/30/2022 17:20:04 CST		
Date Processed:	7/31/2023 17:13:49 CST		

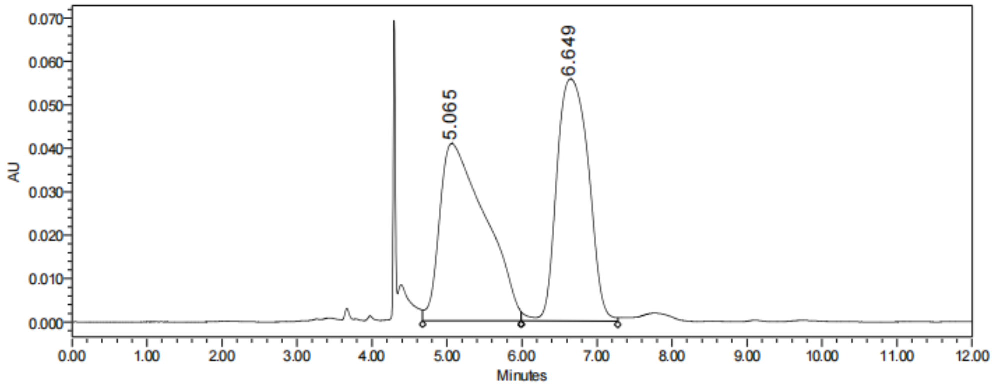

	RT	Area	% Area	Height
1	5.065	1735259	51.27	40847
2	6.649	1649341	48.73	55778

Asy-**7z**

SAMPLE INFORMATION			
Sample Name:	cxh-10-81-5-asy-AD-1%	Acquired By:	System
Sample Type:	Unknown	Sample Set Name:	09301
Vial:	12	Acq. Method Set:	1%
Injection #:	1	Processing Method	7z asy
Injection Volume:	10.00 ul	Channel Name:	254.0nm
Run Time:	12.0 Minutes	Proc. Chnl. Descr.:	2998 PDA 254.0 nm (2998
Date Acquired:	9/30/2022 17:32:58 CST		
Date Processed:	7/31/2023 17:13:18 CST		

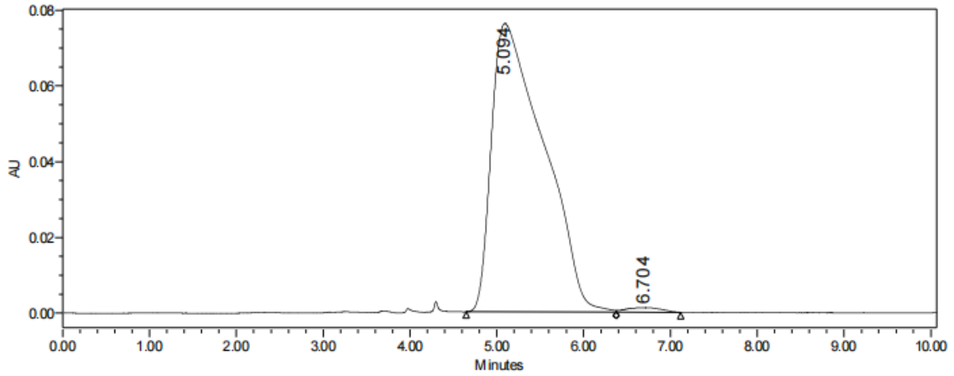

	RT	Area	% Area	Height
1	5.094	3183444	98.91	76139
2	6.704	35004	1.09	1243

Rac-**7aa**

SAMPLE INFORMATION

Sample Name:	cxh-10-84-4-rac-AD-1%	Acquired By:	System
Sample Type:	Control	Sample Set Name:	
Vial:	12	Acq. Method Set:	1%
Injection #:	2	Processing Method:	7aa rac
Injection Volume:	10.00 ul	Channel Name:	254.0nm
Run Time:	30.0 Minutes	Proc. Chnl. Descr.:	2998 PDA 254.0 nm (2998
Date Acquired:	10/9/2022 6:52:34 PM CST		
Date Processed:	7/31/2023 8:02:26 PM CST		

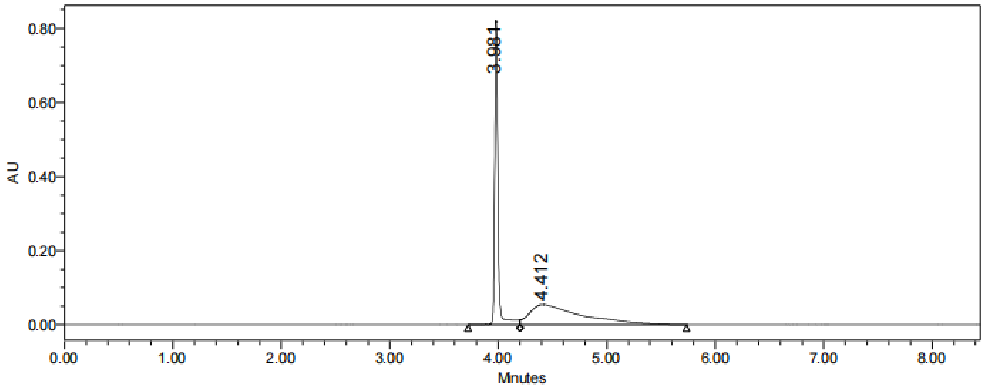

	RT	Area	% Area	Height
1	3.981	1684615	48.62	822880
2	4.412	1780180	51.38	54179

Asy-**7aa**

SAMPLE INFORMATION

Sample Name:	cxh-10-84-4-asy-AD-1%	Acquired By:	System
Sample Type:	Control	Sample Set Name:	
Vial:	30	Acq. Method Set:	1%
Injection #:	1	Processing Method:	7aa asy
Injection Volume:	10.00 ul	Channel Name:	254.0nm
Run Time:	10.0 Minutes	Proc. Chnl. Descr.:	2998 PDA 254.0 nm (2998
Date Acquired:	10/10/2022 11:27:58 AM CST		
Date Processed:	7/31/2023 8:01:41 PM CST		

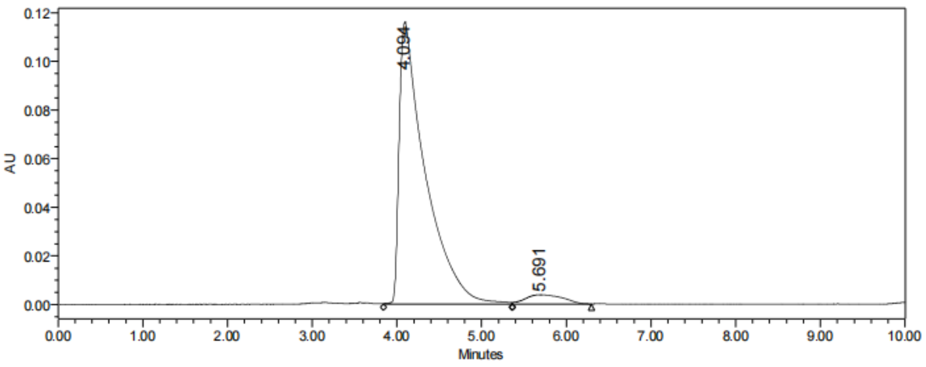

	RT	Area	% Area	Height
1	4.094	2475146	95.49	115862
2	5.691	117026	4.51	3778

Rac-**7ab**

SAMPLE INFORMATION

Sample Name:	cxh-10-97-1-rac-AD-1%	Acquired By:	System
Sample Type:	Unknown	Sample Set Name:	
Vial:	42	Acq. Method Set:	1%
Injection #:	1	Processing Method	7ab rac
Injection Volume:	10.00 ul	Channel Name:	254.0nm
Run Time:	50.0 Minutes	Proc. Chnl. Descr.:	2998 PDA 254.0 nm (2998
Date Acquired:	11/21/2022 11:57:56 CST		
Date Processed:	7/31/2023 20:10:40 CST		

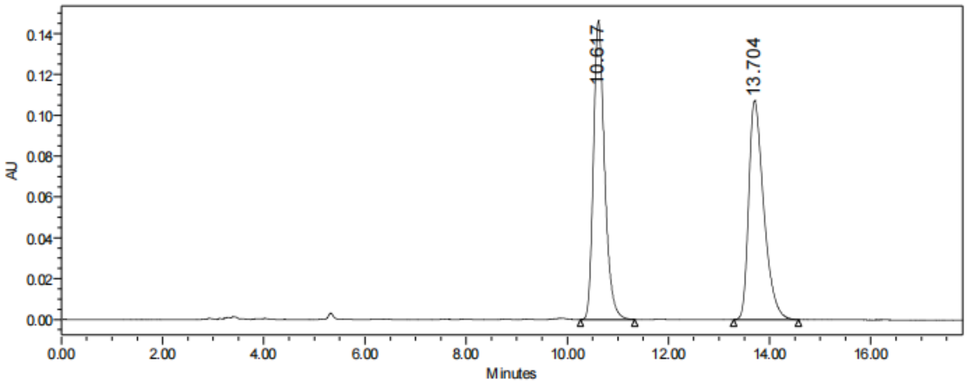

	RT	Area	% Area	Height
1	10.617	2234090	50.08	146268
2	13.704	2227208	49.92	107249

Asy-**7ab**

SAMPLE INFORMATION

Sample Name:	cxh-10-100-1-asy-AD-1%	Acquired By:	System
Sample Type:	Control	Sample Set Name:	
Vial:	85	Acq. Method Set:	1%
Injection #:	1	Processing Method:	7ab rac
Injection Volume:	10.00 ul	Channel Name:	254.0nm
Run Time:	30.0 Minutes	Proc. Chnl. Descr.:	2998 PDA 254.0 nm (2998
Date Acquired:	12/5/2022 4:03:40 PM CST		
Date Processed:	7/31/2023 8:18:45 PM CST		

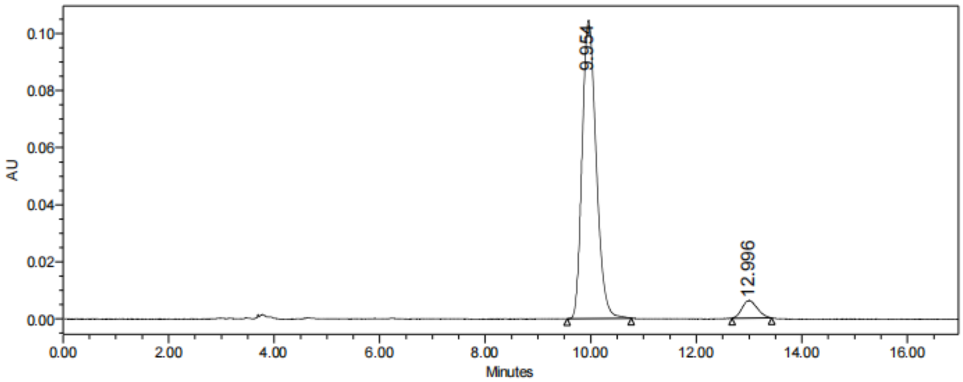

	RT	Area	% Area	Height
1	9.954	1930556	94.05	104383
2	12.996	122090	5.95	6125

Rac-**7ac**

SAMPLE INFORMATION

Sample Name:	cxh-10-97-4-rac-AD-1%	Acquired By:	System
Sample Type:	Control	Sample Set Name:	
Vial:	106	Acq. Method Set:	1%
Injection #:	2	Processing Method:	7ac rac
Injection Volume:	10.00 ul	Channel Name:	254.0nm
Run Time:	50.0 Minutes	Proc. Chnl. Descr.:	2998 PDA 254.0 nm (2998
Date Acquired:	11/26/2022 2:46:56 PM CST		
Date Processed:	7/31/2023 8:16:12 PM CST		

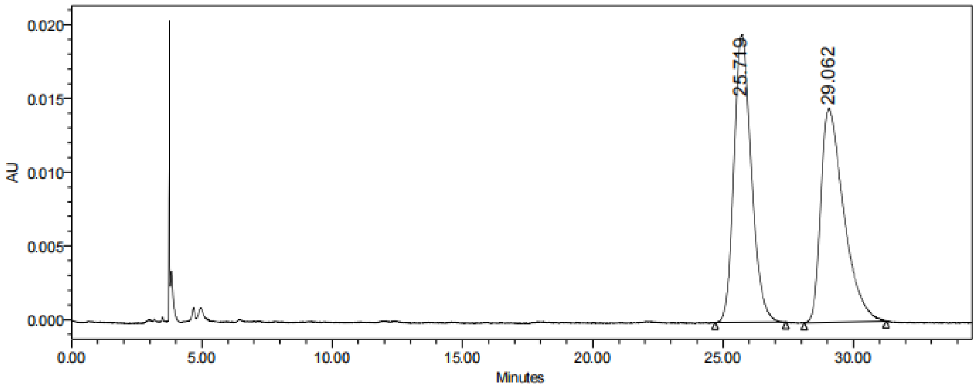

	RT	Area	% Area	Height
1	25.719	904662	50.27	19496
2	29.062	894997	49.73	14520

Asy-**7ac**

SAMPLE INFORMATION

Sample Name:	cxh-10-99-4-asy-AD-1%	Acquired By:	System
Sample Type:	Unknown	Sample Set Name:	1201
Vial:	90	Acq. Method Set:	1%
Injection #:	1	Processing Method:	7ac asy
Injection Volume:	10.00 ul	Channel Name:	254.0nm
Run Time:	40.0 Minutes	Proc. Chnl. Descr.:	2998 PDA 254.0 nm (2998
Date Acquired:	12/1/2022 12:52:49 PM CST		
Date Processed:	7/31/2023 8:14:52 PM CST		

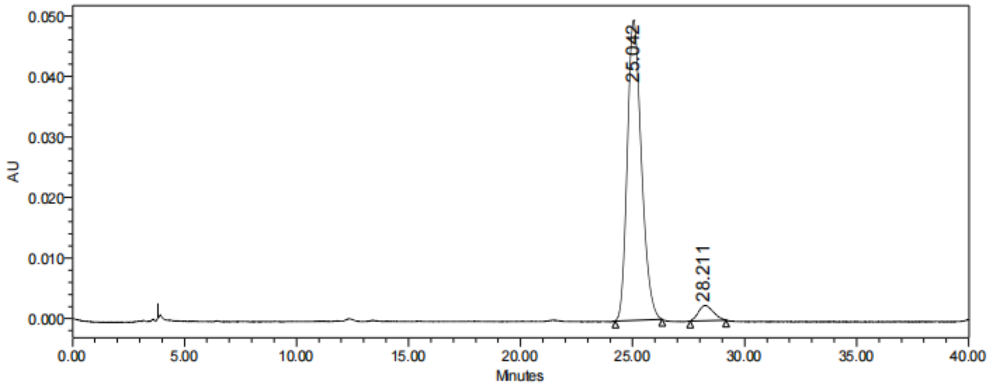

	RT	Area	% Area	Height
1	25.042	2167161	94.97	49495
2	28.211	114818	5.03	2506

Rac-**8**

SAMPLE INFORMATION

Sample Name:	cxh-11-3-2-rac-IC-1%-0.5ML	Acquired By:	System
Sample Type:	Control	Sample Set Name:	
Vial:	119	Acq. Method Set:	1% 05ML
Injection #:	4	Processing Method:	8 rac
Injection Volume:	10.00 ul	Channel Name:	254.0nm
Run Time:	50.0 Minutes	Proc. Chnl. Descr.:	2998 PDA 254.0 nm (2998
Date Acquired:	12/30/2022 2:18:51 PM CST		
Date Processed:	8/1/2023 2:19:49 PM CST		

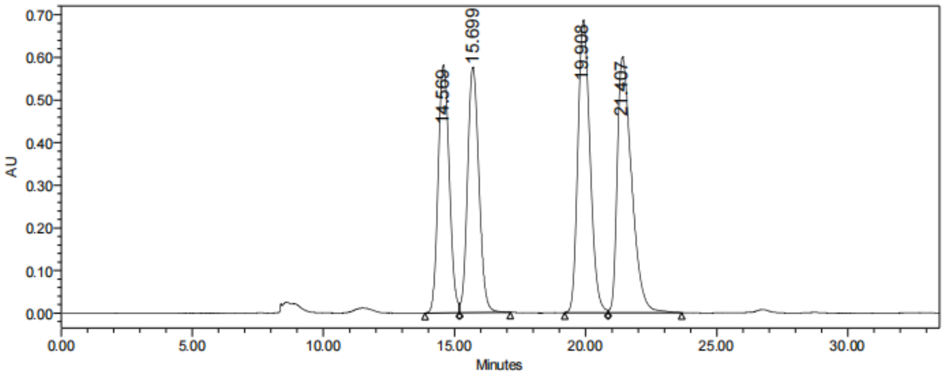

	RT	Area	% Area	Height
1	14.569	17095788	21.55	580962
2	15.699	17397744	21.93	575667
3	19.908	22260324	28.06	685310
4	21.407	22579074	28.46	599147

Asy-**8**

SAMPLE INFORMATION

Sample Name:	cxh-11-3-1-asy-IC-1%-0.5ML	Acquired By:	System
Sample Type:	Control	Sample Set Name:	
Vial:	40	Acq. Method Set:	1% 05ML
Injection #:	1	Processing Method:	8 asy
Injection Volume:	10.00 ul	Channel Name:	254.0nm
Run Time:	35.0 Minutes	Proc. Chnl. Descr.:	2998 PDA 254.0 nm (2998
Date Acquired:	12/30/2022 6:41:29 PM CST		
Date Processed:	8/1/2023 2:21:03 PM CST		

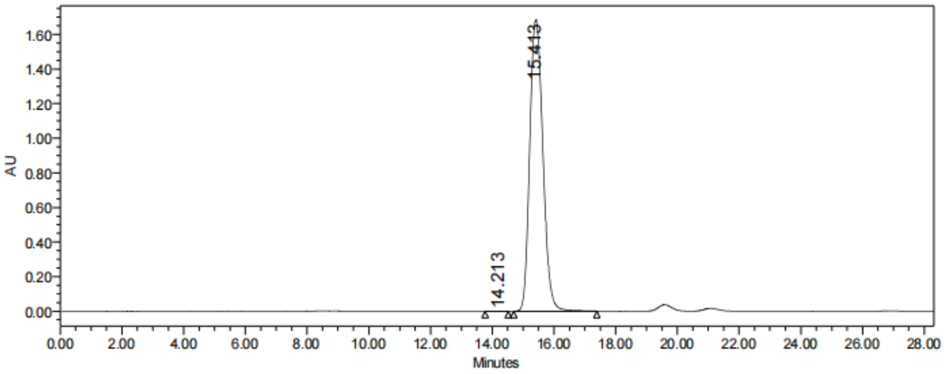

	RT	Area	% Area	Height
1	14.213	19135	0.04	743
2	15.413	50101597	99.96	1682806

Rac-**9**

SAMPLE INFORMATION

Sample Name:	cxh-11-7-3-rac-IG-1%-0.5ml	Acquired By:	System
Sample Type:	Unknown	Sample Set Name:	02083
Vial:	3	Acq. Method Set:	1% 05ML
Injection #:	1	Processing Method:	9 rac
Injection Volume:	10.00 ul	Channel Name:	254.0nm
Run Time:	52.0 Minutes	Proc. Chnl. Descr.:	2998 PDA 254.0 nm (2998
Date Acquired:	2/8/2023 11:37:08 AM CST		
Date Processed:	8/1/2023 2:34:25 PM CST		

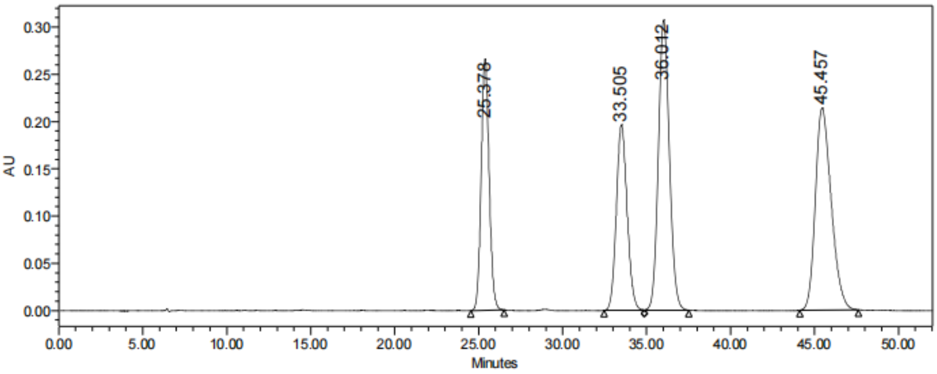

	RT	Area	% Area	Height
1	25.378	8672789	19.07	265782
2	33.505	8683906	19.10	196338
3	36.012	14085645	30.98	306873
4	45.457	14026853	30.85	213598

Asy-**9**

SAMPLE INFORMATION

Sample Name:	cxh-11-7-4-asy-IG-1%-0.5ml	Acquired By:	System
Sample Type:	Unknown	Sample Set Name:	02083
Vial:	4	Acq. Method Set:	1% 05ML
Injection #:	1	Processing Method:	9 asy
Injection Volume:	10.00 ul	Channel Name:	254.0nm
Run Time:	52.0 Minutes	Proc. Chnl. Descr.:	2998 PDA 254.0 nm (2998
Date Acquired:	2/8/2023 12:29:52 PM CST		
Date Processed:	8/1/2023 2:35:20 PM CST		

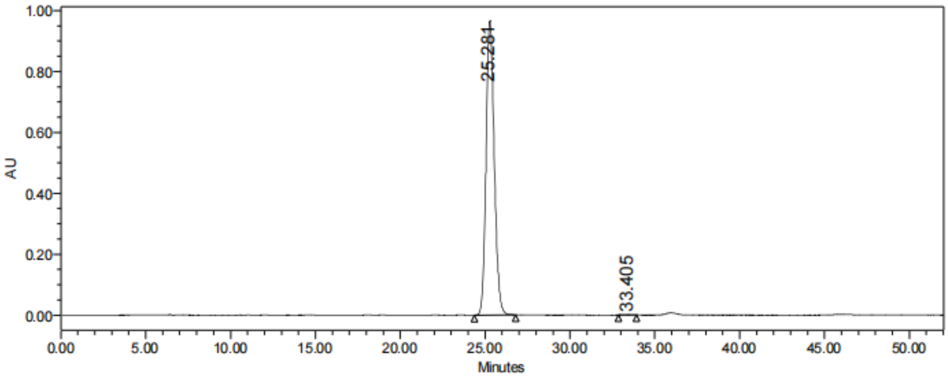

	RT	Area	% Area	Height
1	25.281	31992441	99.99	964621
2	33.405	3888	0.01	175

Rac-**10**

SAMPLE INFORMATION

Sample Name:	cxh-11-6-1-rac-AS-1%-0.5ML	Acquired By:	System
Sample Type:	Control	Sample Set Name:	
Vial:	11	Acq. Method Set:	1% 05ML
Injection #:	15	Processing Method:	10 rac
Injection Volume:	10.00 ul	Channel Name:	254.0nm
Run Time:	18.0 Minutes	Proc. Chnl. Descr.:	2998 PDA 254.0 nm (2998
Date Acquired:	1/11/2023 8:26:35 PM CST		
Date Processed:	8/1/2023 2:26:21 PM CST		

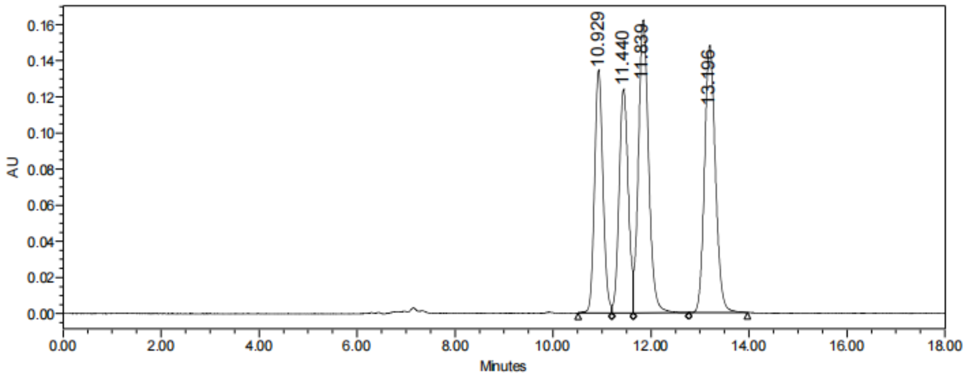

	RT	Area	% Area	Height
1	10.929	1607946	20.59	134201
2	11.440	1583121	20.28	123647
3	11.839	2332057	29.87	161833
4	13.196	2285092	29.27	147735

Asy-**10**

SAMPLE INFORMATION

Sample Name:	cxh-11-6-4-asy1-AS-1%-0.5ML	Acquired By:	System
Sample Type:	Unknown	Sample Set Name:	0111
Vial:	36	Acq. Method Set:	1% 05ML
Injection #:	1	Processing Method:	10 asy
Injection Volume:	10.00 ul	Channel Name:	254.0nm
Run Time:	20.0 Minutes	Proc. Chnl. Descr.:	2998 PDA 254.0 nm (2998
Date Acquired:	1/11/2023 9:04:47 PM CST		
Date Processed:	8/1/2023 2:28:48 PM CST		

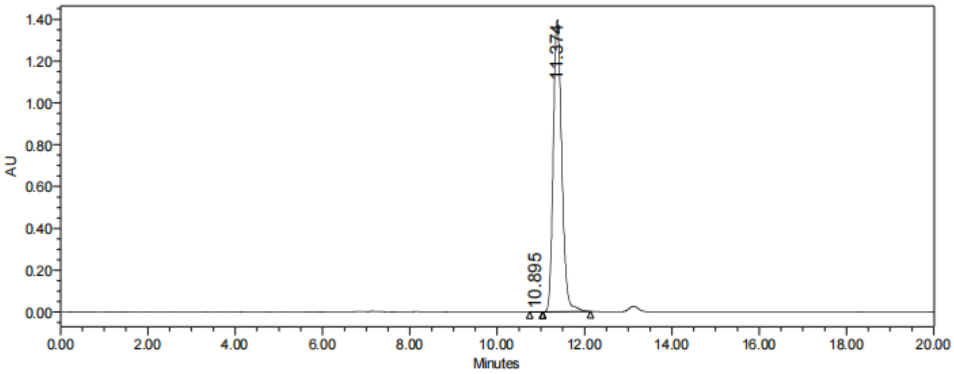

	RT	Area	% Area	Height
1	10.895	2664	0.01	330
2	11.374	18966684	99.99	1393484

Rac-**11**

SAMPLE INFORMATION

Sample Name:		Acquired By:	System
Sample Type:	Unknown	Sample Set Name:	
Vial:	4	Acq. Method Set:	2% 05ML
Injection #:	2	Processing Method	11 rac
Injection Volume:	10.00 ul	Channel Name:	254.0nm
Run Time:	80.0 Minutes	Proc. Chnl. Descr.:	2998 PDA 254.0 nm (2998
Date Acquired:	1/13/2023 18:03:55 CST		
Date Processed:	8/1/2023 14:34:26 CST		

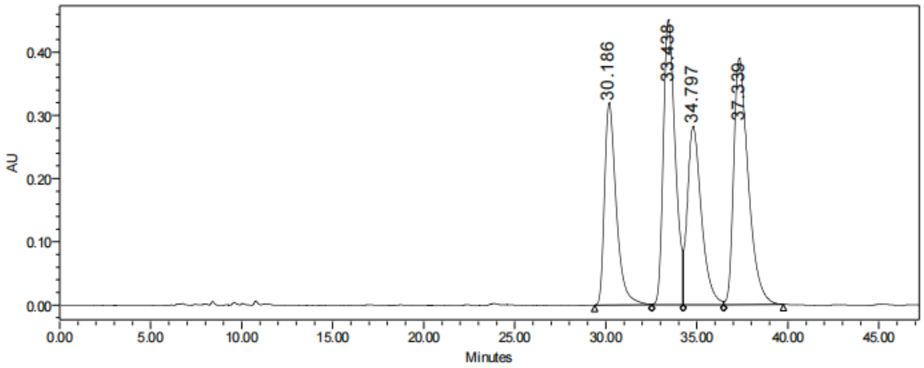

	RT	Area	% Area	Height
1	30.186	14434602	20.21	319441
2	33.438	20477294	28.68	450280
3	34.797	15051618	21.08	281639
4	37.339	21444396	30.03	389362

Asy-**11**

SAMPLE INFORMATION

Sample Name:		Acquired By:	System
Sample Type:	Unknown	Sample Set Name:	
Vial:	36	Acq. Method Set:	2% 05ML
Injection #:	1	Processing Method	11 asy
Injection Volume:	10.00 ul	Channel Name:	254.0nm
Run Time:	50.0 Minutes	Proc. Chnl. Descr.:	2998 PDA 254.0 nm (2998
Date Acquired:	1/13/2023 18:52:39 CST		
Date Processed:	8/1/2023 14:39:38 CST		

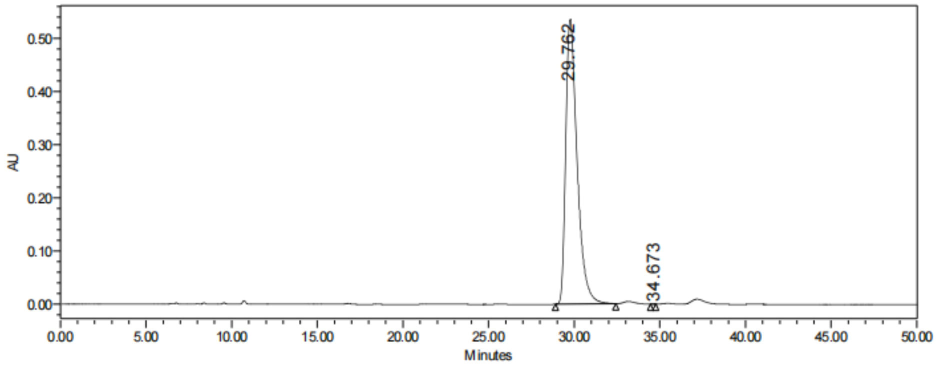

	RT	Area	% Area	Height
1	29.762	24764774	100.00	534829
2	34.673	232	0.00	-34

Rac-**12**

SAMPLE INFORMATION

Sample Name:	cxh-11-7-1-rac-OJ-1%-0.5ml	Acquired By:	System
Sample Type:	Unknown	Sample Set Name:	0113
Vial:	14	Acq. Method Set:	1% 05ML
Injection #:	1	Processing Method:	12 rac
Injection Volume:	10.00 ul	Channel Name:	254.0nm
Run Time:	45.0 Minutes	Proc. Chnl. Descr.:	2998 PDA 254.0 nm (2998
Date Acquired:	1/13/2023 11:56:25 AM CST		
Date Processed:	8/1/2023 2:31:06 PM CST		

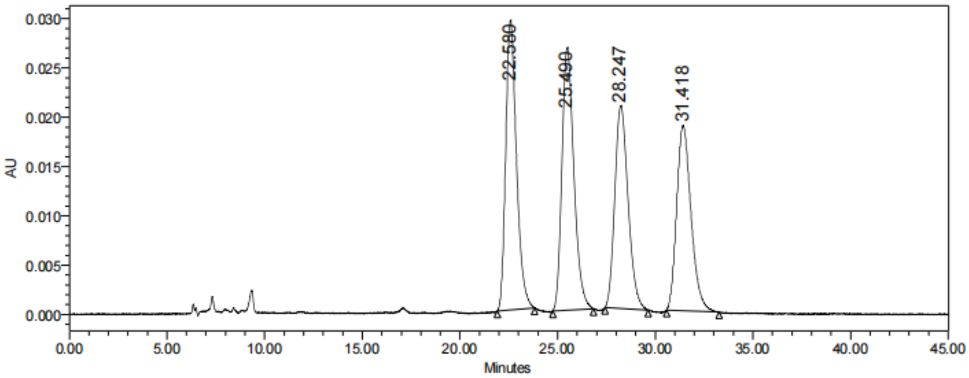

	RT	Area	% Area	Height
1	22.580	1134652	26.99	29365
2	25.490	1129267	26.86	26617
3	28.247	974131	23.17	20578
4	31.418	965769	22.97	18810

Asy-**12**

SAMPLE INFORMATION

Sample Name:	cxh-11-7-2-asy-OJ-1%-0.5ml	Acquired By:	System
Sample Type:	Unknown	Sample Set Name:	01132
Vial:	33	Acq. Method Set:	1% 05ML
Injection #:	1	Processing Method:	12 asy
Injection Volume:	10.00 ul	Channel Name:	254.0nm
Run Time:	45.0 Minutes	Proc. Chnl. Descr.:	2998 PDA 254.0 nm (2998
Date Acquired:	1/13/2023 12:47:48 PM CST		
Date Processed:	8/1/2023 2:32:31 PM CST		

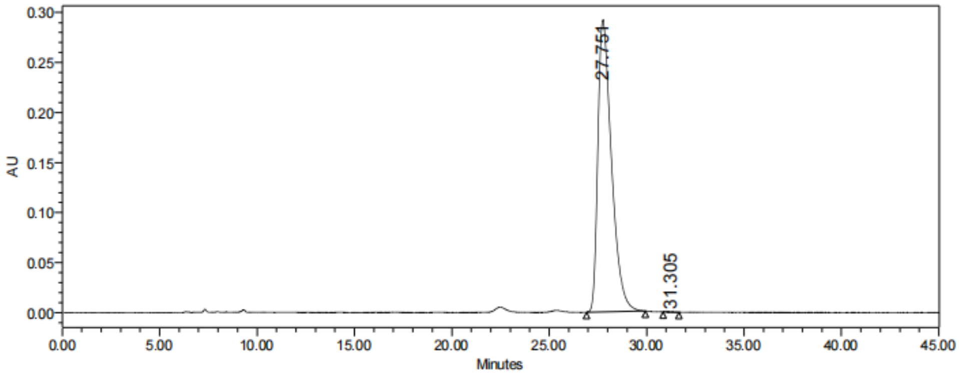

	RT	Area	% Area	Height
1	27.751	14264279	99.99	291712
2	31.305	1096	0.01	82

Rac-**13**

SAMPLE INFORMATION

Sample Name:	cxh-11-9-1-rac-IG-1%	Acquired By:	System
Sample Type:	Unknown	Sample Set Name:	0302
Vial:	106	Acq. Method Set:	1%
Injection #:	1	Processing Method:	13 rac
Injection Volume:	10.00 ul	Channel Name:	254.0nm
Run Time:	17.0 Minutes	Proc. Chnl. Descr.:	2998 PDA 254.0 nm (2998
Date Acquired:	3/2/2023 2:28:50 PM CST		
Date Processed:	8/1/2023 2:59:51 PM CST		

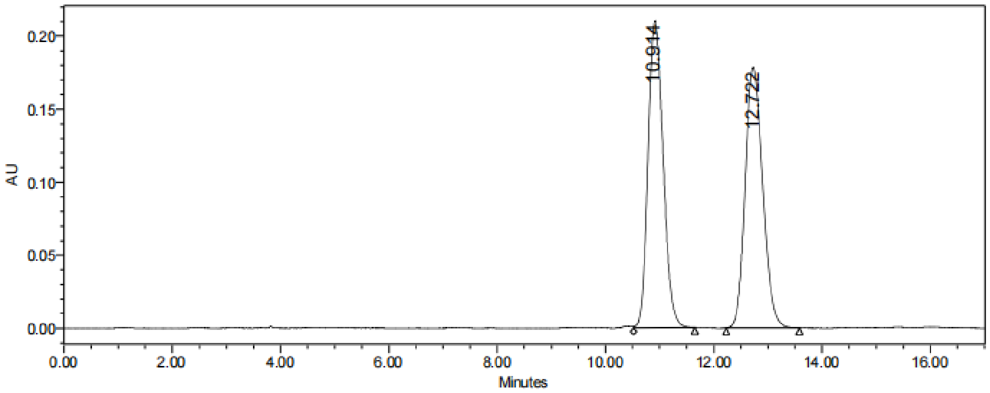

	RT	Area	% Area	Height
1	10.914	4036954	49.96	210234
2	12.722	4043926	50.04	178151

Asy-**13**

SAMPLE INFORMATION

Sample Name:	cxh-11-9-1-asy-IG-1%	Acquired By:	System
Sample Type:	Control	Sample Set Name:	
Vial:	106	Acq. Method Set:	1%
Injection #:	1	Processing Method:	13 asy
Injection Volume:	10.00 ul	Channel Name:	254.0nm
Run Time:	18.0 Minutes	Proc. Chnl. Descr.:	2998 PDA 254.0 nm (2998
Date Acquired:	3/2/2023 3:27:33 PM CST		
Date Processed:	8/1/2023 3:01:45 PM CST		

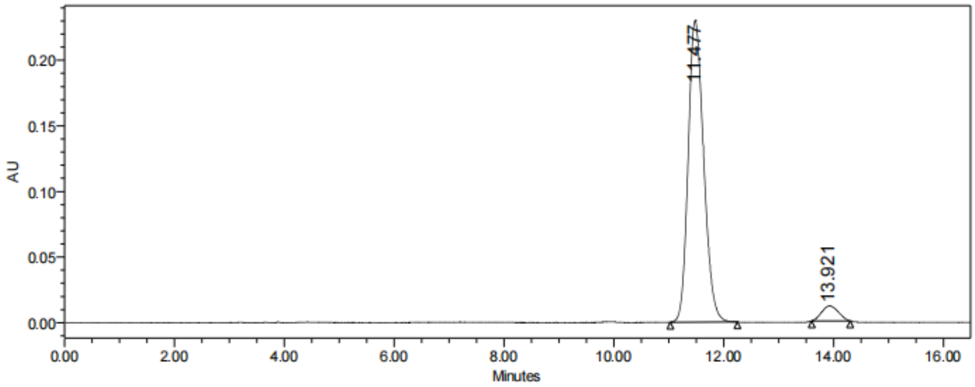

	RT	Area	% Area	Height
1	11.477	4530955	94.92	229802
2	13.921	242273	5.08	11465

Rac-**15**

SAMPLE INFORMATION

Sample Name:	cxh-11-13-1-rac-AD-1%	Acquired By:	System
Sample Type:	Unknown	Sample Set Name:	03072
Vial:	57	Acq. Method Set:	1%
Injection #:	1	Processing Method:	15 rac
Injection Volume:	10.00 ul	Channel Name:	230.0nm
Run Time:	13.0 Minutes	Proc. Chnl. Descr.:	2998 PDA 230.0 nm (2998
Date Acquired:	3/7/2023 7:08:41 PM CST		
Date Processed:	8/1/2023 3:21:03 PM CST		

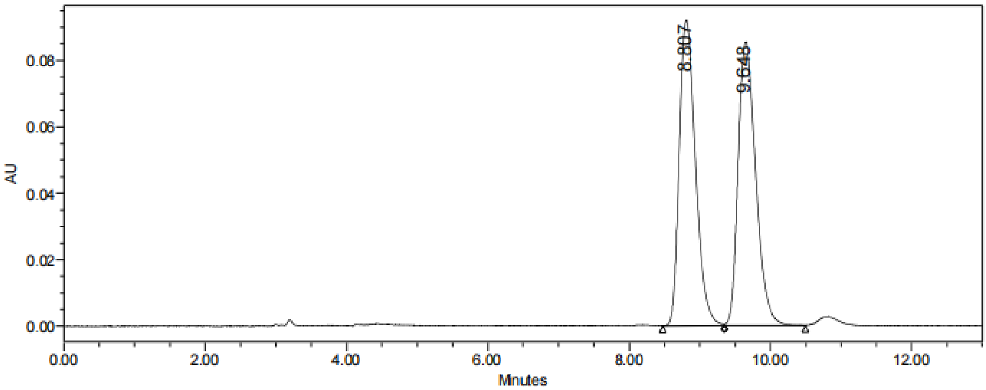

	RT	Area	% Area	Height
1	8.807	1470654	49.94	91922
2	9.648	1474098	50.06	85388

Asy-**15**

SAMPLE INFORMATION

Sample Name:	cxh-11-13-5-asy-AD-1%	Acquired By:	System
Sample Type:	Unknown	Sample Set Name:	03072
Vial:	58	Acq. Method Set:	1%
Injection #:	1	Processing Method:	15 asy
Injection Volume:	10.00 ul	Channel Name:	230.0nm
Run Time:	13.0 Minutes	Proc. Chnl. Descr.:	2998 PDA 230.0 nm (2998
Date Acquired:	3/7/2023 7:22:22 PM CST		
Date Processed:	8/1/2023 3:20:25 PM CST		

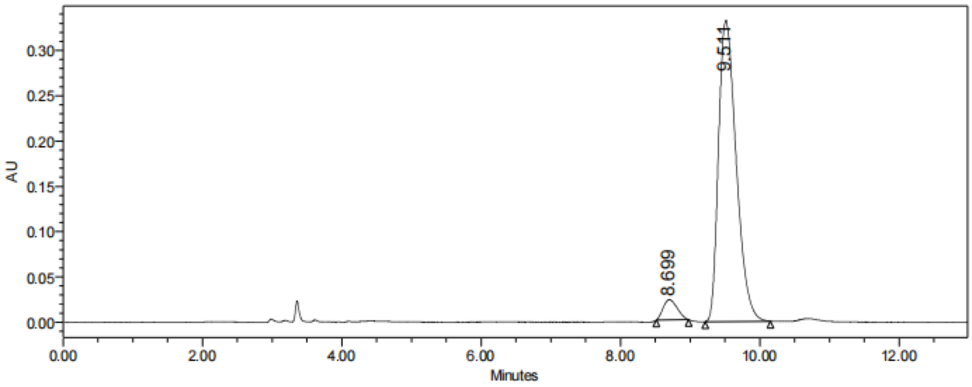

	RT	Area	% Area	Height
1	8.699	314542	5.12	22230
2	9.511	5825908	94.88	331771

Rac-**16**

SAMPLE INFORMATION

Sample Name:	cxh-11-9-2-rac-AS-1%	Acquired By:	System
Sample Type:	Control	Sample Set Name:	
Vial:	46	Acq. Method Set:	1%
Injection #:	1	Processing Method:	16 rac
Injection Volume:	10.00 ul	Channel Name:	254.0nm
Run Time:	60.0 Minutes	Proc. Chnl. Descr.:	2998 PDA 254.0 nm (2998
Date Acquired:	3/13/2023 9:11:01 PM CST		
Date Processed:	8/1/2023 3:06:22 PM CST		

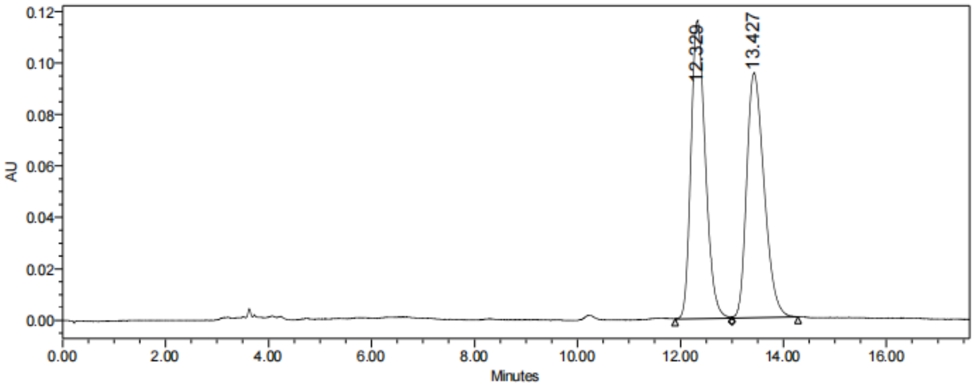

	RT	Area	% Area	Height
1	12.329	2258390	50.05	115779
2	13.427	2253760	49.95	95088

Asy-**16**

SAMPLE INFORMATION

Sample Name:	cxh-11-9-2-asy-AS-1%	Acquired By:	System
Sample Type:	Control	Sample Set Name:	
Vial:	98	Acq. Method Set:	1%
Injection #:	1	Processing Method:	16 asy
Injection Volume:	10.00 ul	Channel Name:	254.0nm
Run Time:	20.0 Minutes	Proc. Chnl. Descr.:	2998 PDA 254.0 nm (2998
Date Acquired:	3/14/2023 11:20:22 AM CST		
Date Processed:	8/1/2023 3:08:01 PM CST		

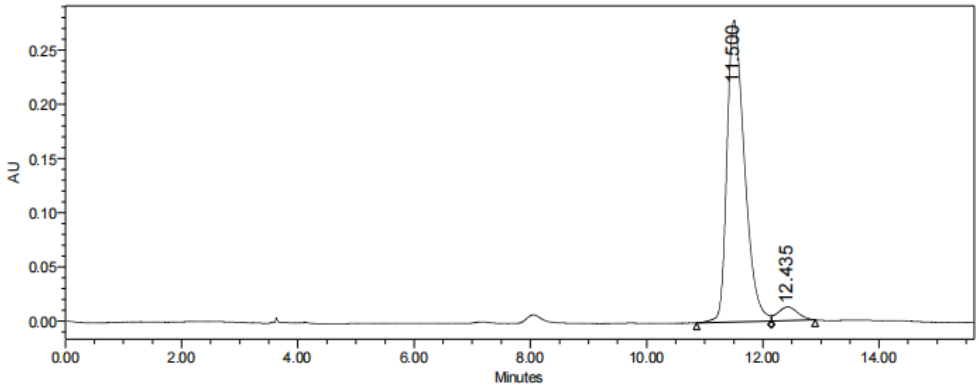

	RT	Area	% Area	Height
1	11.500	5807937	95.03	277914
2	12.435	303749	4.97	12668

Rac-**17**

SAMPLE INFORMATION

Sample Name:	cxh-11-14-1-rac-IE-1%-0.5ML	Acquired By:	System
Sample Type:	Control	Sample Set Name:	
Vial:	20	Acq. Method Set:	1% 05ML
Injection #:	2	Processing Method:	17 rac
Injection Volume:	20.00 ul	Channel Name:	220.0nm
Run Time:	50.0 Minutes	Proc. Chnl. Descr.:	2998 PDA 220.0 nm (2998
Date Acquired:	4/22/2023 2:51:34 PM CST		
Date Processed:	8/1/2023 3:27:08 PM CST		

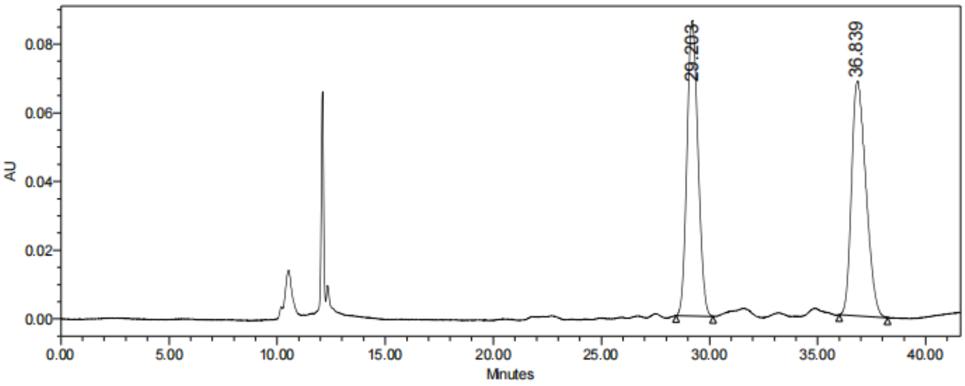

	RT	Area	% Area	Height
1	29.203	3069873	49.53	85928
2	36.839	3127986	50.47	68270

Asy-**17**

SAMPLE INFORMATION

Sample Name:	cxh-11-17-5-asy-IE-1%-0.5ML	Acquired By:	System
Sample Type:	Control	Sample Set Name:	
Vial:	21	Acq. Method Set:	1% 05ML
Injection #:	1	Processing Method:	17 asy
Injection Volume:	15.00 ul	Channel Name:	220.0nm
Run Time:	50.0 Minutes	Proc. Chnl. Descr.:	2998 PDA 220.0 nm (2998
Date Acquired:	4/22/2023 3:34:43 PM CST		
Date Processed:	8/1/2023 3:27:40 PM CST		

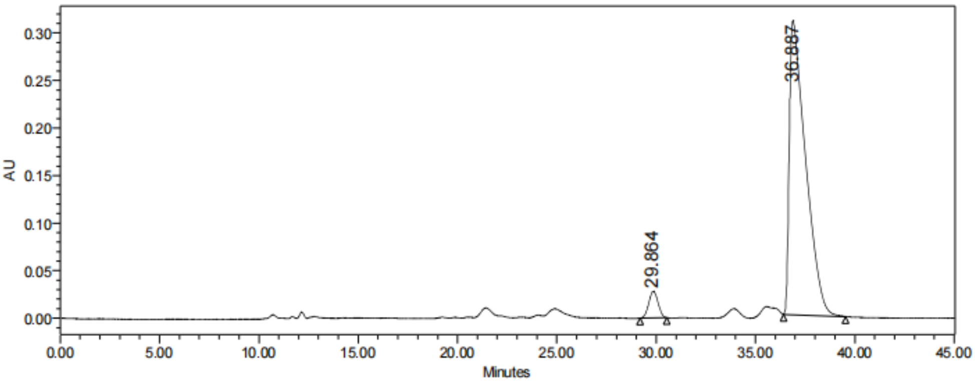

	RT	Area	% Area	Height
1	29.864	916944	4.98	28164
2	36.887	17496596	95.02	309115

Rac-**20**

SAMPLE INFORMATION

Sample Name:	cxh-11-16-4-rac-IA-2%	Acquired By:	System
Sample Type:	Unknown	Sample Set Name:	06021
Vial:	28	Acq. Method Set:	2%
Injection #:	1	Processing Method:	20 rac
Injection Volume:	10.00 ul	Channel Name:	211.0nm
Run Time:	25.0 Minutes	Proc. Chnl. Descr.:	2998 PDA 211.0 nm (2998
Date Acquired:	6/2/2023 7:02:57 PM CST		
Date Processed:	8/1/2023 3:46:34 PM CST		

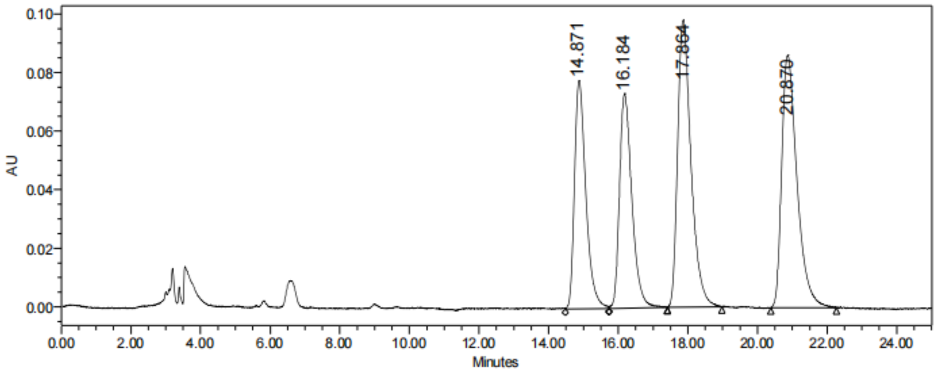

	RT	Area	% Area	Height
1	14.871	1775850	20.17	77822
2	16.184	1784344	20.27	73243
3	17.864	2603428	29.58	97978
4	20.870	2638882	29.98	86236

(*R*,*S*)-**20**

SAMPLE INFORMATION

Sample Name:	cxh-11-24-5-asy-IA-2%	Acquired By:	System
Sample Type:	Control	Sample Set Name:	
Vial:	49	Acq. Method Set:	2%
Injection #:	2	Processing Method:	RS 20
Injection Volume:	10.00 ul	Channel Name:	211.0nm
Run Time:	25.0 Minutes	Proc. Chnl. Descr.:	2998 PDA 211.0 nm (2998
Date Acquired:	6/2/2023 10:05:10 PM CST		
Date Processed:	8/1/2023 3:54:11 PM CST		

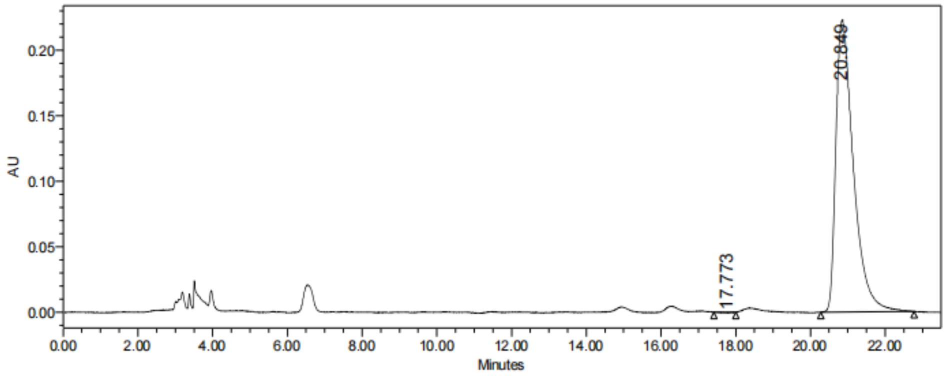

	RT	Area	% Area	Height
1	17.773	6772	0.09	-403
2	20.849	7221917	99.91	222851

Rac-**20**

SAMPLE INFORMATION			
Sample Name:	cxh-11-16-4-rac-IA-2%	Acquired By:	System
Sample Type:	Unknown	Sample Set Name:	06021
Vial:	28	Acq. Method Set:	2%
Injection #:	1	Processing Method:	20 rac
Injection Volume:	10.00 ul	Channel Name:	211.0nm
Run Time:	25.0 Minutes	Proc. Chnl. Descr.:	2998 PDA 211.0 nm (2998
Date Acquired:	6/2/2023 7:02:57 PM CST		
Date Processed:	8/1/2023 3:46:34 PM CST		

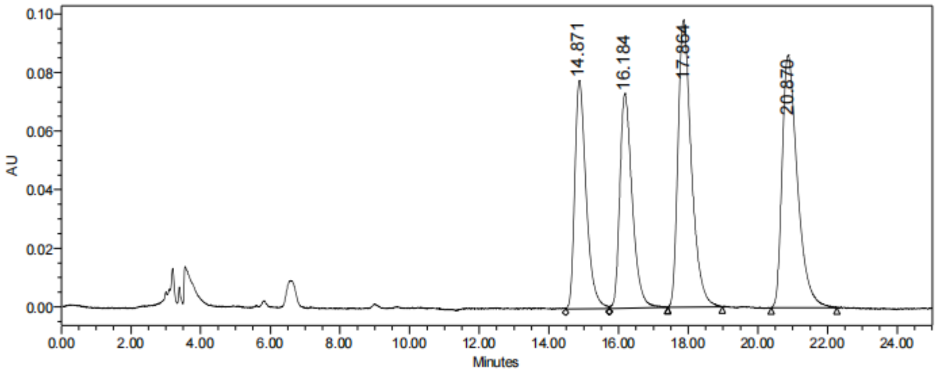

	RT	Area	% Area	Height
1	14.871	1775850	20.17	77822
2	16.184	1784344	20.27	73243
3	17.864	2603428	29.58	97978
4	20.870	2638882	29.98	86236

(*S*,*R*)-**20**

SAMPLE INFORMATION			
Sample Name:	cxh-11-19-5-asy-IA-2%	Acquired By:	System
Sample Type:	Unknown	Sample Set Name:	06021
Vial:	29	Acq. Method Set:	2%
Injection #:	1	Processing Method:	SR 20
Injection Volume:	10.00 ul	Channel Name:	211.0nm
Run Time:	25.0 Minutes	Proc. Chnl. Descr.:	2998 PDA 211.0 nm (2998
Date Acquired:	6/2/2023 7:28:40 PM CST		
Date Processed:	8/1/2023 3:53:52 PM CST		

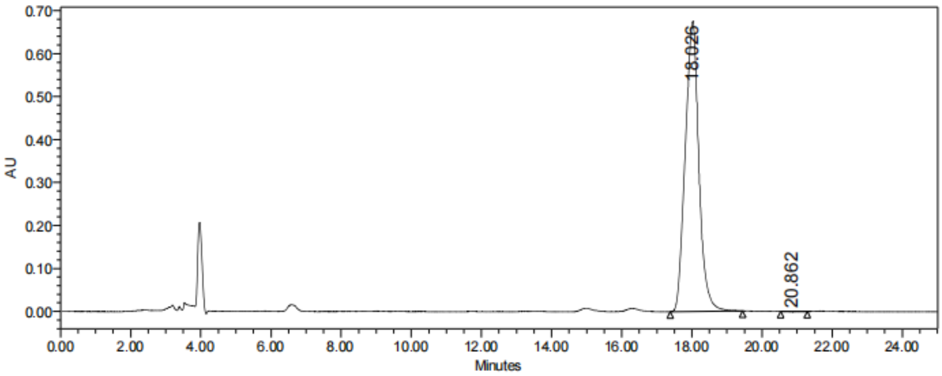

	RT	Area	% Area	Height
1	18.026	18079894	99.97	674901
2	20.862	5529	0.03	-348

Rac-**20**

SAMPLE INFORMATION

Sample Name:	cxh-11-16-4-rac-IA-2%	Acquired By:	System
Sample Type:	Unknown	Sample Set Name:	06021
Vial:	28	Acq. Method Set:	2%
Injection #:	1	Processing Method:	20 rac
Injection Volume:	10.00 ul	Channel Name:	211.0nm
Run Time:	25.0 Minutes	Proc. Chnl. Descr.:	2998 PDA 211.0 nm (2998
Date Acquired:	6/2/2023 7:02:57 PM CST		
Date Processed:	8/1/2023 3:46:34 PM CST		

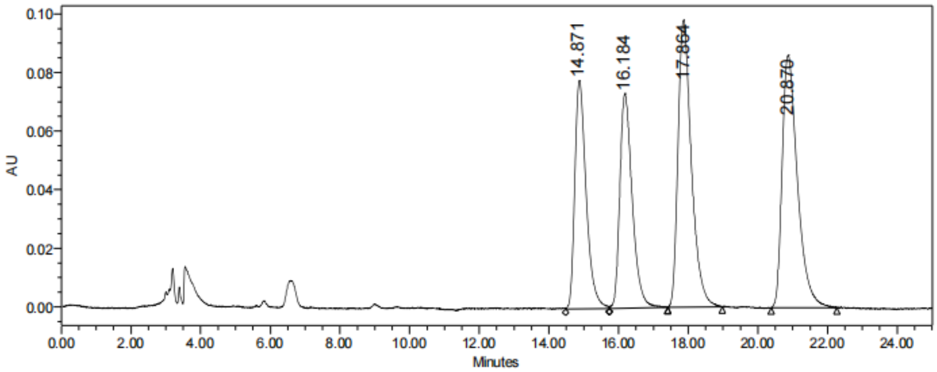

	RT	Area	% Area	Height
1	14.871	1775850	20.17	77822
2	16.184	1784344	20.27	73243
3	17.864	2603428	29.58	97978
4	20.870	2638882	29.98	86236

(*R*,*R*)-**20**

SAMPLE INFORMATION

Sample Name:	cxh-11-26-5-asy-IA-2%	Acquired By:	System
Sample Type:	Unknown	Sample Set Name:	06021
Vial:	32	Acq. Method Set:	2%
Injection #:	1	Processing Method:	RR 20
Injection Volume:	10.00 ul	Channel Name:	211.0nm
Run Time:	25.0 Minutes	Proc. Chnl. Descr.:	2998 PDA 211.0 nm (2998
Date Acquired:	6/2/2023 8:45:47 PM CST		
Date Processed:	8/1/2023 3:52:44 PM CST		

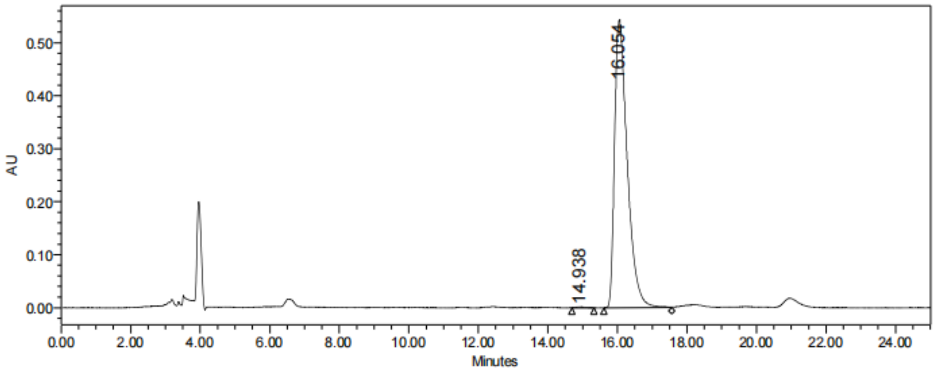

	RT	Area	% Area	Height
1	14.938	27908	0.20	1354
2	16.054	13659358	99.80	542848

Rac-**20**

SAMPLE INFORMATION

Sample Name:	cxh-11-16-4-rac-IA-2%	Acquired By:	System
Sample Type:	Unknown	Sample Set Name:	06021
Vial:	28	Acq. Method Set:	2%
Injection #:	1	Processing Method:	20 rac
Injection Volume:	10.00 ul	Channel Name:	211.0nm
Run Time:	25.0 Minutes	Proc. Chnl. Descr.:	2998 PDA 211.0 nm (2998
Date Acquired:	6/2/2023 7:02:57 PM CST		
Date Processed:	8/1/2023 3:46:34 PM CST		

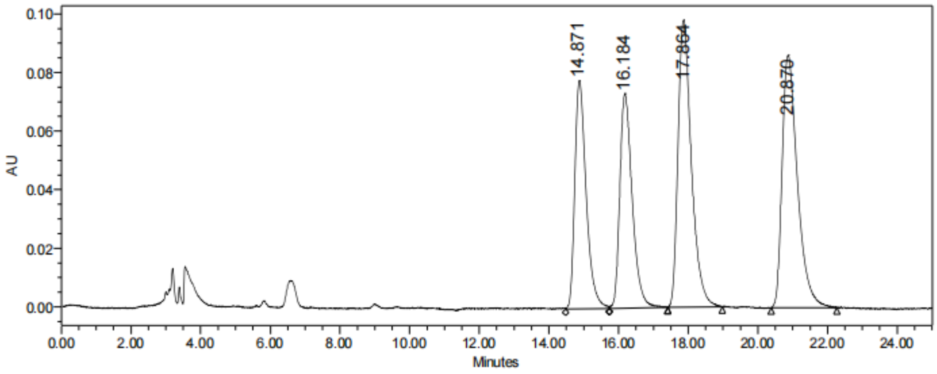

	RT	Area	% Area	Height
1	14.871	1775850	20.17	77822
2	16.184	1784344	20.27	73243
3	17.864	2603428	29.58	97978
4	20.870	2638882	29.98	86236

(*S*,*S*)-**20**

SAMPLE INFORMATION

Sample Name:	cxh-11-25-5-asy-IA-2%	Acquired By:	System
Sample Type:	Unknown	Sample Set Name:	06021
Vial:	31	Acq. Method Set:	2%
Injection #:	1	Processing Method:	SS 20
Injection Volume:	10.00 ul	Channel Name:	211.0nm
Run Time:	25.0 Minutes	Proc. Chnl. Descr.:	2998 PDA 211.0 nm (2998
Date Acquired:	6/2/2023 8:20:05 PM CST		
Date Processed:	8/1/2023 3:53:59 PM CST		

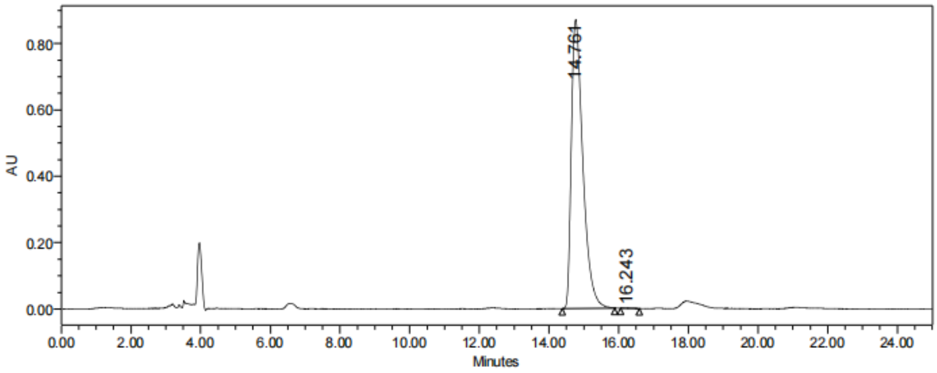

	RT	Area	% Area	Height
1	14.761	20033490	99.92	869251
2	16.243	16828	0.08	1032

Rac-**21**

SAMPLE INFORMATION

Sample Name:	cxh-11-23-1-rac-IA-3%	Acquired By:	System
Sample Type:	Unknown	Sample Set Name:	0612
Vial:	27	Acq. Method Set:	3%
Injection #:	1	Processing Method:	21 rac
Injection Volume:	10.00 ul	Channel Name:	211.0nm
Run Time:	22.0 Minutes	Proc. Chnl. Descr.:	2998 PDA 211.0 nm (2998
Date Acquired:	6/12/2023 12:04:43 PM CST		
Date Processed:	8/1/2023 4:00:09 PM CST		

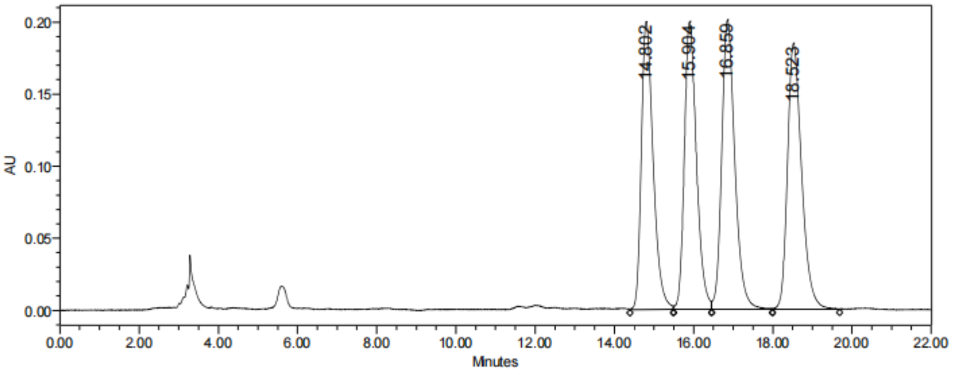

	RT	Area	% Area	Height
1	14.802	4150047	23.63	199670
2	15.904	4173142	23.76	199754
3	16.859	4641116	26.43	200912
4	18.523	4595864	26.17	184599

(*S*,*S*)-**21**

SAMPLE INFORMATION

Sample Name:	cxh-11-24-6-asy-IA-3%	Acquired By:	System
Sample Type:	Unknown	Sample Set Name:	0612
Vial:	29	Acq. Method Set:	3%
Injection #:	1	Processing Method:	RS 21
Injection Volume:	10.00 ul	Channel Name:	211.0nm
Run Time:	22.0 Minutes	Proc. Chnl. Descr.:	2998 PDA 211.0 nm (2998
Date Acquired:	6/12/2023 12:50:05 PM CST		
Date Processed:	8/1/2023 4:06:08 PM CST		

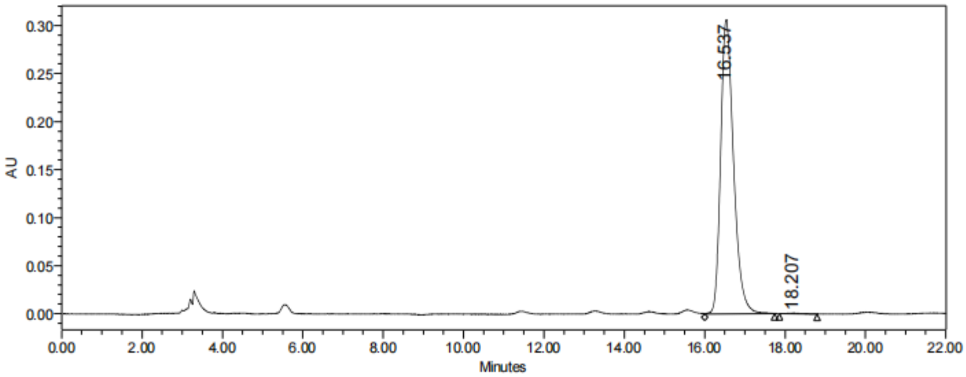

	RT	Area	% Area	Height
1	16.537	6853523	99.76	305488
2	18.207	16430	0.24	754

Rac-**21**

SAMPLE INFORMATION

Sample Name:	cxh-11-23-1-rac-IA-3%	Acquired By:	System
Sample Type:	Unknown	Sample Set Name:	0612
Vial:	27	Acq. Method Set:	3%
Injection #:	1	Processing Method:	21 rac
Injection Volume:	10.00 ul	Channel Name:	211.0nm
Run Time:	22.0 Minutes	Proc. Chnl. Descr.:	2998 PDA 211.0 nm (2998
Date Acquired:	6/12/2023 12:04:43 PM CST		
Date Processed:	8/1/2023 4:00:09 PM CST		

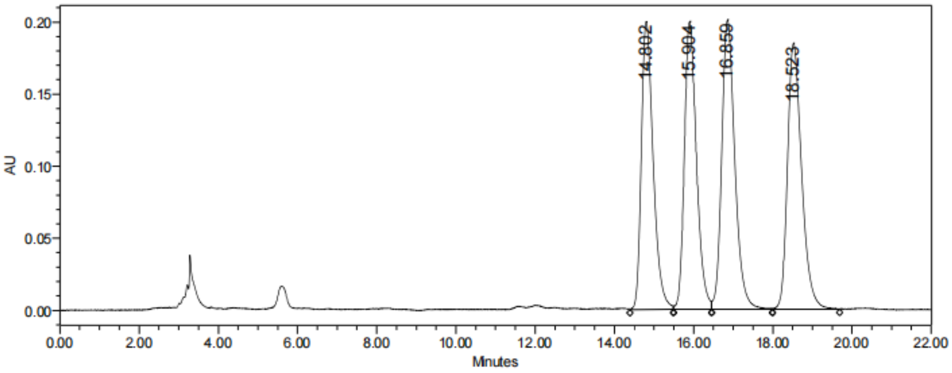

	RT	Area	% Area	Height
1	14.802	4150047	23.63	199670
2	15.904	4173142	23.76	199754
3	16.859	4641116	26.43	200912
4	18.523	4595864	26.17	184599

(*R*,*R*)-**21**

SAMPLE INFORMATION

Sample Name:	cxh-11-19-6-asy-IA-3%	Acquired By:	System
Sample Type:	Unknown	Sample Set Name:	0612
Vial:	28	Acq. Method Set:	3%
Injection #:	1	Processing Method:	SR 21
Injection Volume:	10.00 ul	Channel Name:	211.0nm
Run Time:	22.0 Minutes	Proc. Chnl. Descr.:	2998 PDA 211.0 nm (2998
Date Acquired:	6/12/2023 12:27:25 PM CST		
Date Processed:	8/1/2023 4:05:28 PM CST		

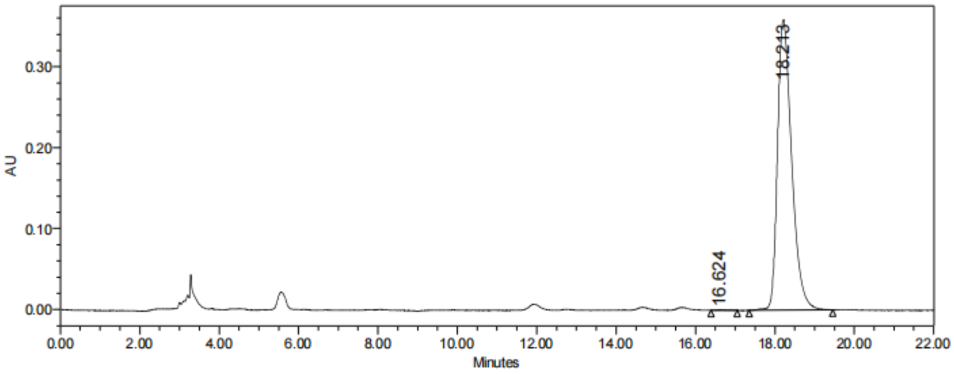

	RT	Area	% Area	Height
1	16.624	12022	0.13	664
2	18.213	8904535	99.87	358398

Rac-**21**

SAMPLE INFORMATION

Sample Name:	cxh-11-23-1-rac-IA-3%	Acquired By:	System
Sample Type:	Unknown	Sample Set Name:	0612
Vial:	27	Acq. Method Set:	3%
Injection #:	1	Processing Method:	21 rac
Injection Volume:	10.00 ul	Channel Name:	211.0nm
Run Time:	22.0 Minutes	Proc. Chnl. Descr.:	2998 PDA 211.0 nm (2998
Date Acquired:	6/12/2023 12:04:43 PM CST		
Date Processed:	8/1/2023 4:00:09 PM CST		

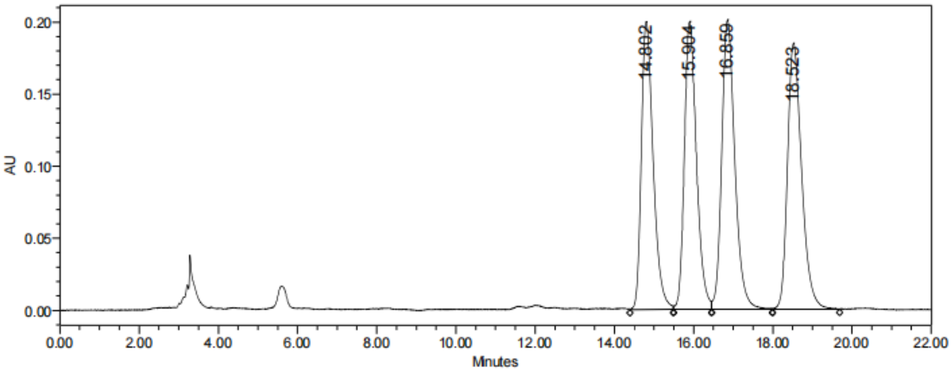

	RT	Area	% Area	Height
1	14.802	4150047	23.63	199670
2	15.904	4173142	23.76	199754
3	16.859	4641116	26.43	200912
4	18.523	4595864	26.17	184599

(*S*,*R*)-**21**

SAMPLE INFORMATION

Sample Name:	cxh-11-26-6-asy-IA-3%	Acquired By:	System
Sample Type:	Unknown	Sample Set Name:	0612
Vial:	31	Acq. Method Set:	3%
Injection #:	1	Processing Method:	RR 21
Injection Volume:	10.00 ul	Channel Name:	211.0nm
Run Time:	22.0 Minutes	Proc. Chnl. Descr.:	2998 PDA 211.0 nm (2998
Date Acquired:	6/12/2023 1:35:29 PM CST		
Date Processed:	8/1/2023 4:08:25 PM CST		

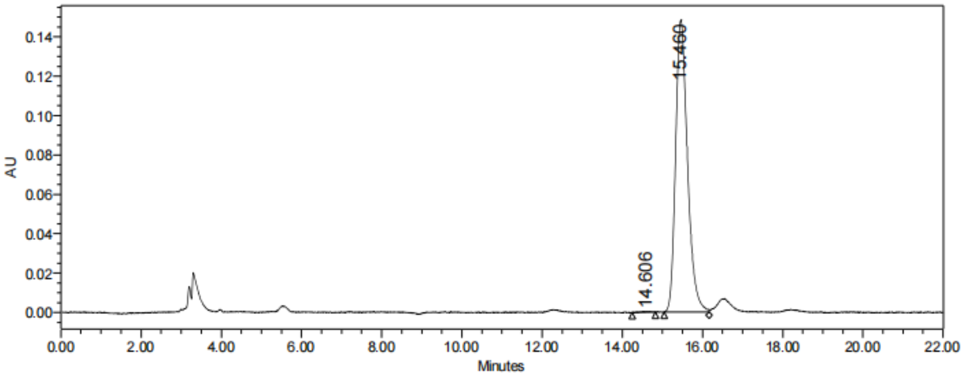

	RT	Area	% Area	Height
1	14.606	4984	0.16	442
2	15.460	3051059	99.84	148207

Rac-**21**

S A M P L E   I N F O R M A T I O N

Sample Name:	cxh-11-23-1-rac-IA-3%	Acquired By:	System
Sample Type:	Unknown	Sample Set Name:	0612
Vial:	27	Acq. Method Set:	3%
Injection #:	1	Processing Method:	21 rac
Injection Volume:	10.00 ul	Channel Name:	211.0nm
Run Time:	22.0 Minutes	Proc. Chnl. Descr.:	2998 PDA 211.0 nm (2998
Date Acquired:	6/12/2023 12:04:43 PM CST		
Date Processed:	8/1/2023 4:00:09 PM CST		

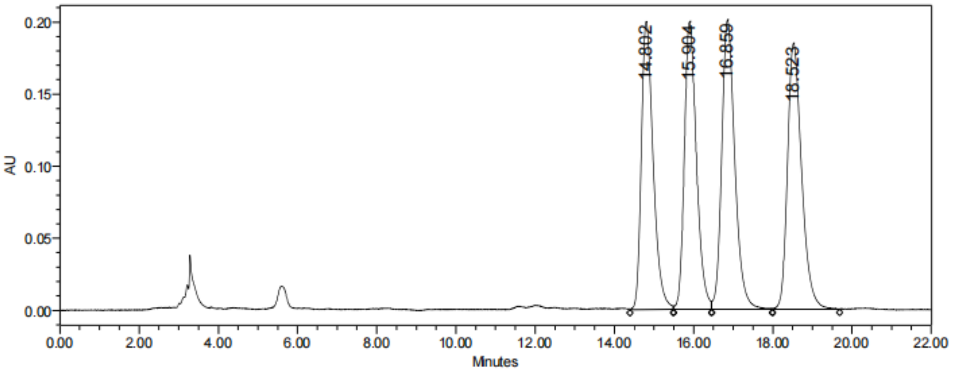

	RT	Area	% Area	Height
1	14.802	4150047	23.63	199670
2	15.904	4173142	23.76	199754
3	16.859	4641116	26.43	200912
4	18.523	4595864	26.17	184599

(*R*,*S*)-**21**

S A M P L E   I N F O R M A T I O N

Sample Name:	cxh-11-25-6-asy-IA-3%	Acquired By:	System
Sample Type:	Unknown	Sample Set Name:	0612
Vial:	30	Acq. Method Set:	3%
Injection #:	1	Processing Method:	SS 21
Injection Volume:	10.00 ul	Channel Name:	211.0nm
Run Time:	22.0 Minutes	Proc. Chnl. Descr.:	2998 PDA 211.0 nm (2998
Date Acquired:	6/12/2023 1:12:47 PM CST		
Date Processed:	8/1/2023 4:07:34 PM CST		

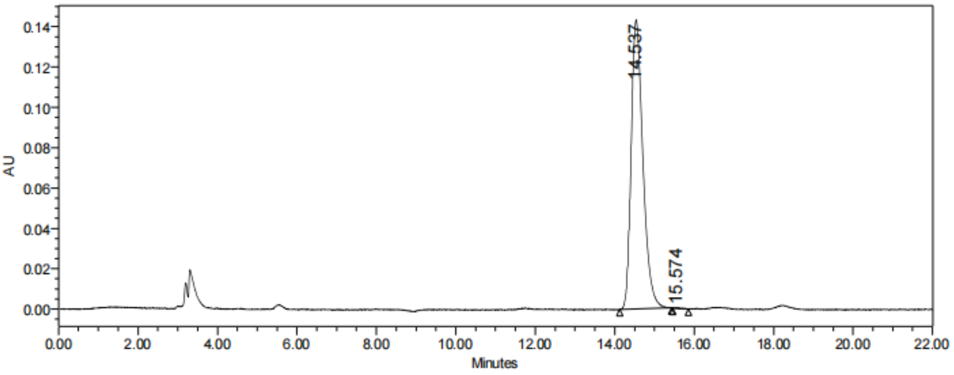

	RT	Area	% Area	Height
1	14.537	2934690	99.90	143299
2	15.574	2851	0.10	293

8. References

1. M. G. Woll, H. Qi, A. Turpoff, N. Zhang, X. Zhang, G. Chen, C. Li, S. Huang, T. Yang, Y.-C.Moon, C.-S. Lee, S. Choi, N. G. Almstead, N. A. Naryshkin, A. Dakka, J. Narasimhan, V. Gabbeta, E. Welch, X. Zhao, N. Risher, J. Sheedy, M. Weetall, G. M. Karp, Discovery and optimization of small molecule splicing modifiers of survival motor neuron 2 as a treatment for spinal muscular atrophy. *J. Med. Chem.* **2016**, *59*, 6070.
2. H. C. Shen, F.-X. Ding, S. L. Colletti, *α*-Heteroarylation of esters, lactones, amides, and lactams by nucleophilic aromatic substitution. *Org. Lett.* **2006**, *8*, 1447.
3. Z.-T. He, X. Jiang, J. F. Hartwig, Stereodivergent construction of tertiary fluorides in vicinal stereogenic pairs by allylic substitution with iridium and copper catalysts. *J. Am. Chem. Soc*. **2019**, *141*, 13066.
4. Z. Wang, A. Ying, Z. Fan, C. Hervieu, L. Zhang, Tertiary amino group in cationic gold catalyst: tethered frustrated Lewis pairs that enable ligand-controlled regiodivergent and stereoselective isomerizations of propargylic esters. *ACS Catal.* **2017**, *7*, 3676.
